# Supplementary figures and images for: Integrative analysis of the salt stress response in cyanobacteria
Source: Biol Direct. 2021 Dec 14;16:26. doi: 10.1186/s13062-021-00316-4 (PMC8670252; doi:10.1186/s13062-021-00316-4)

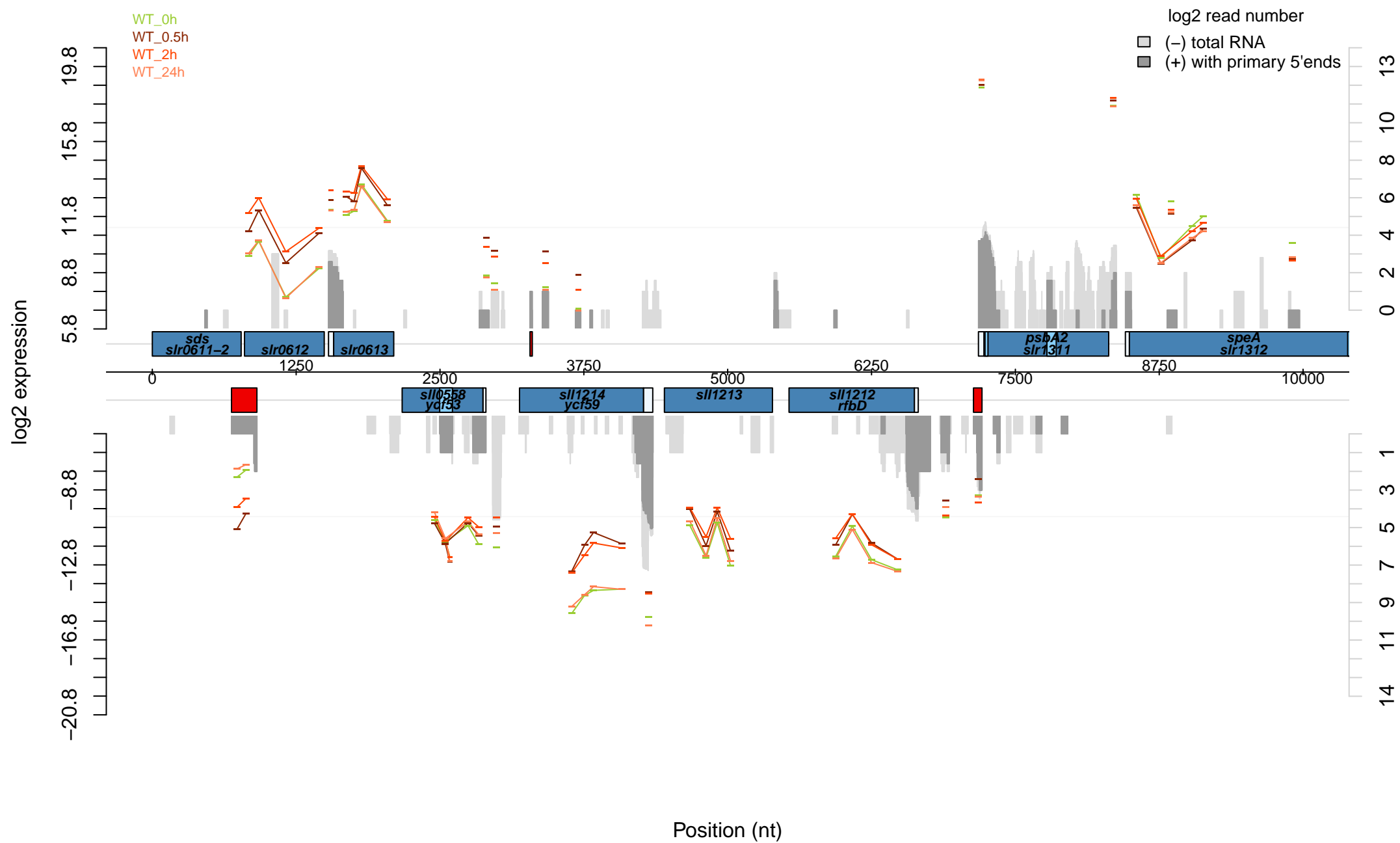

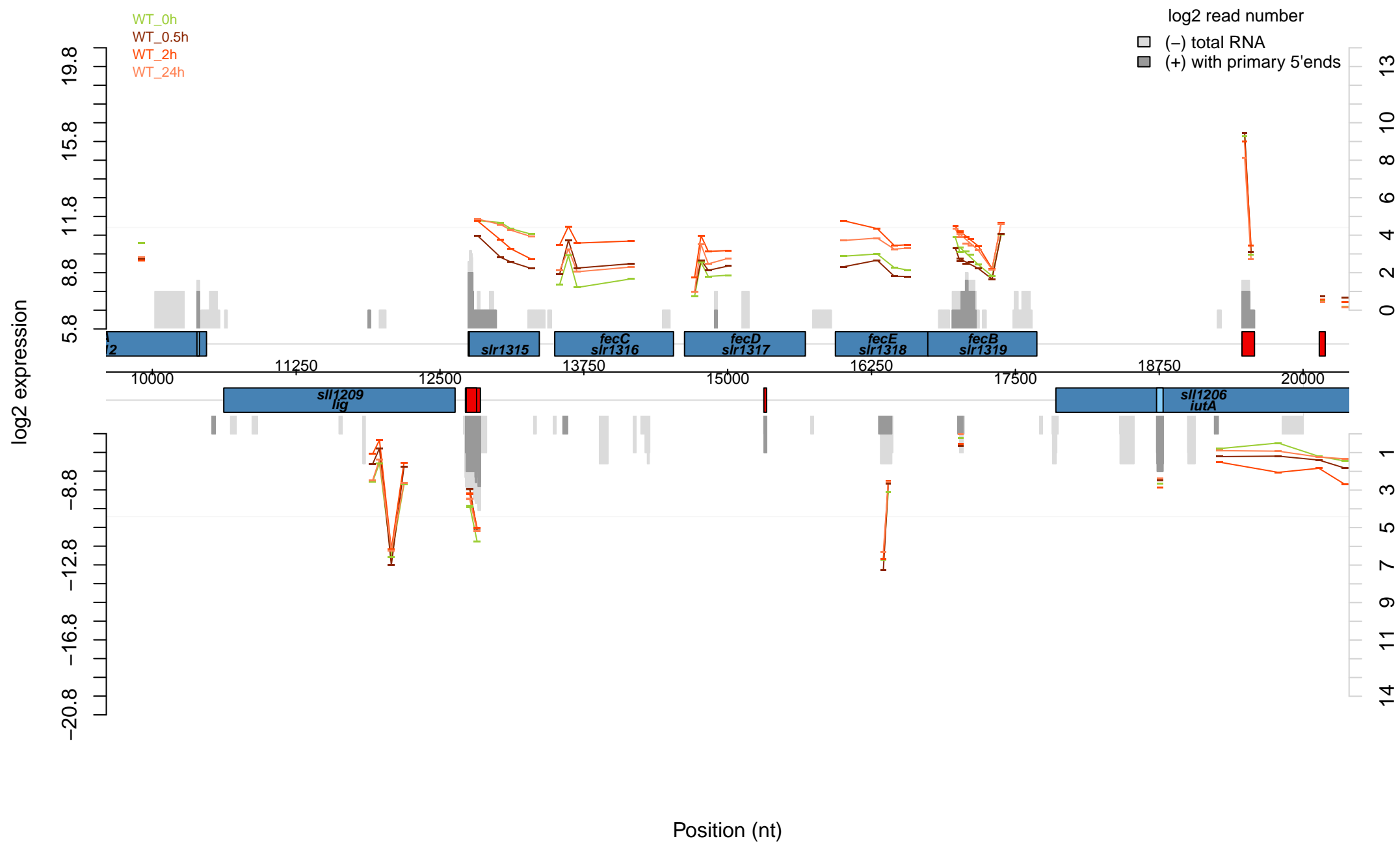

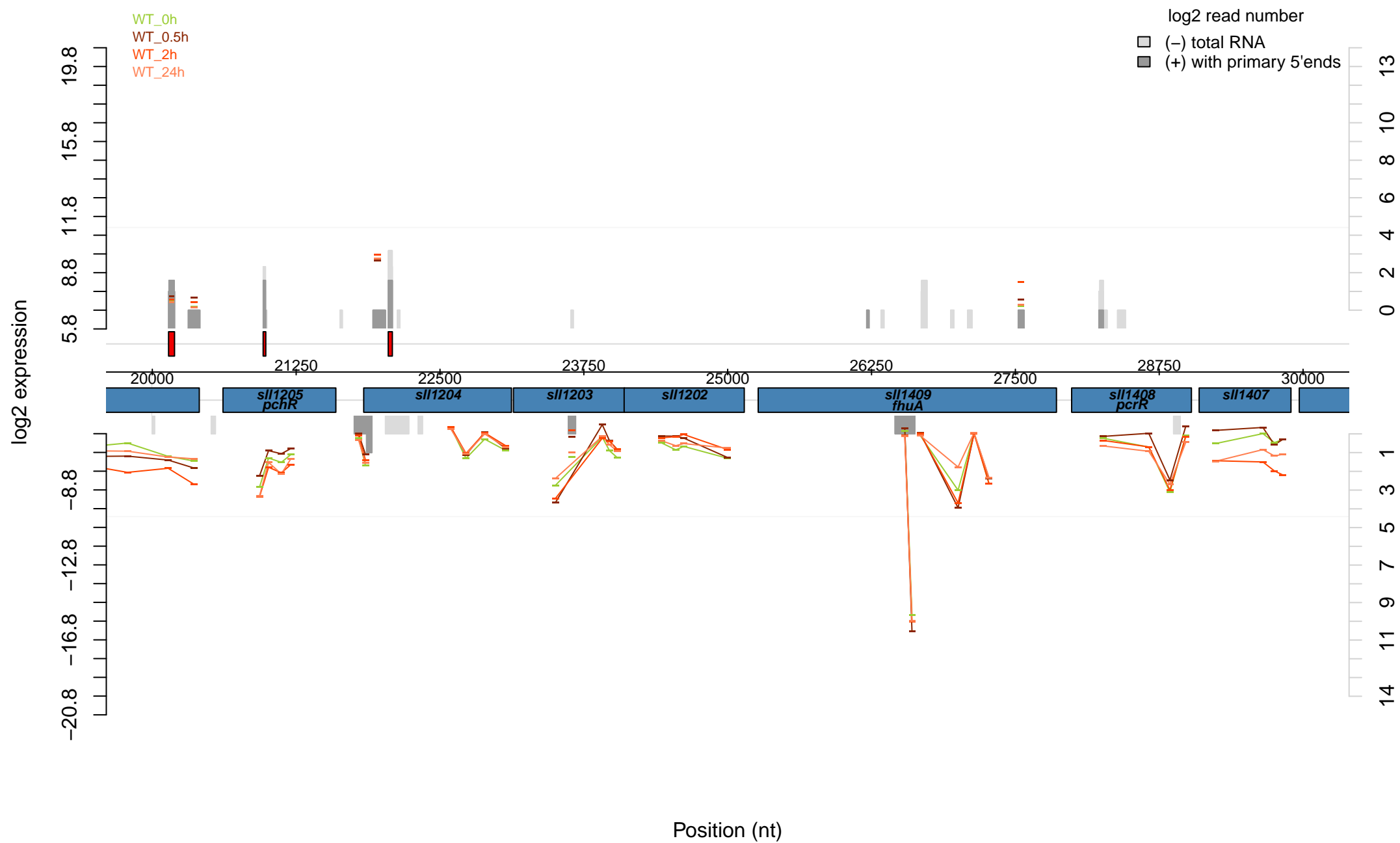

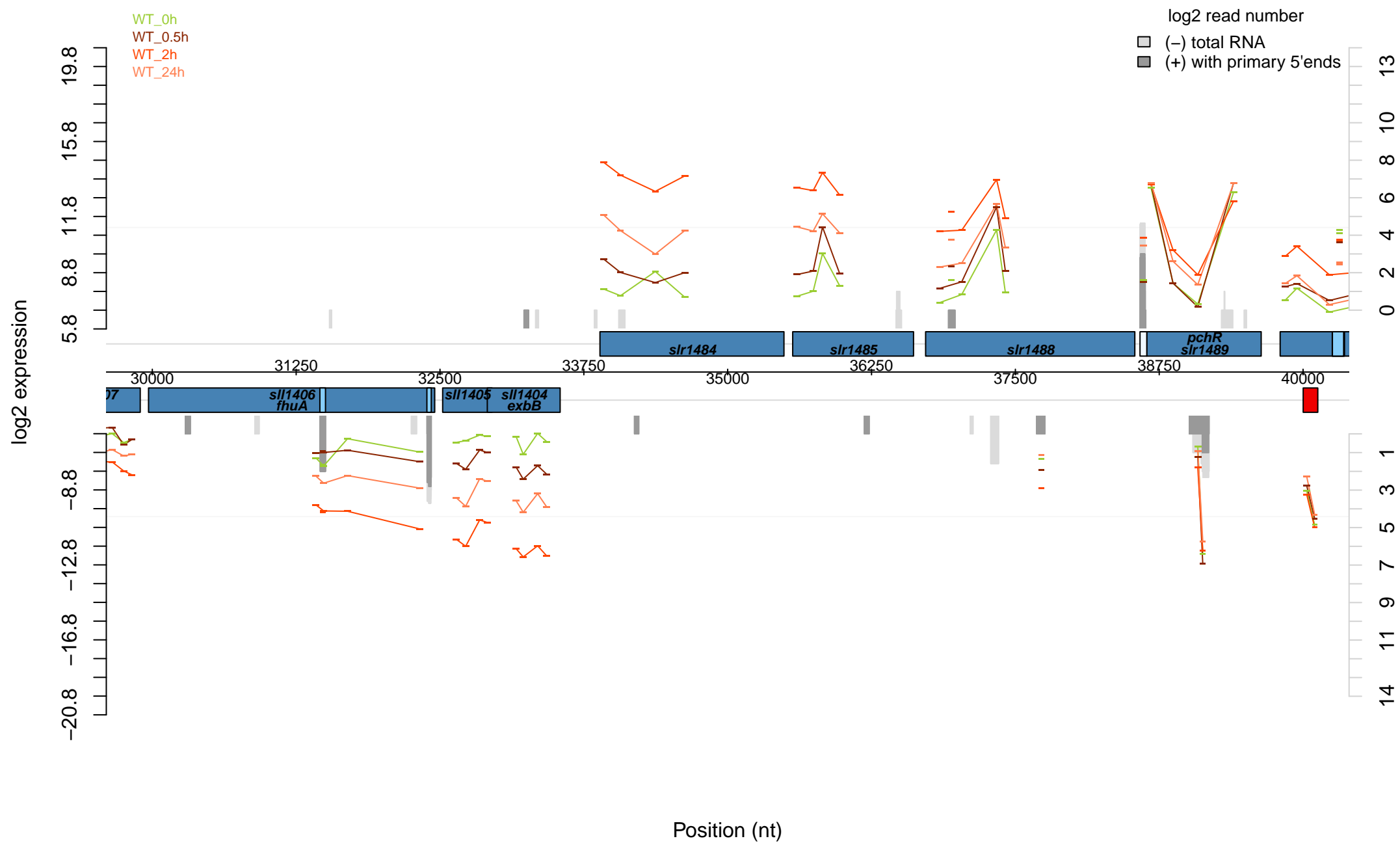

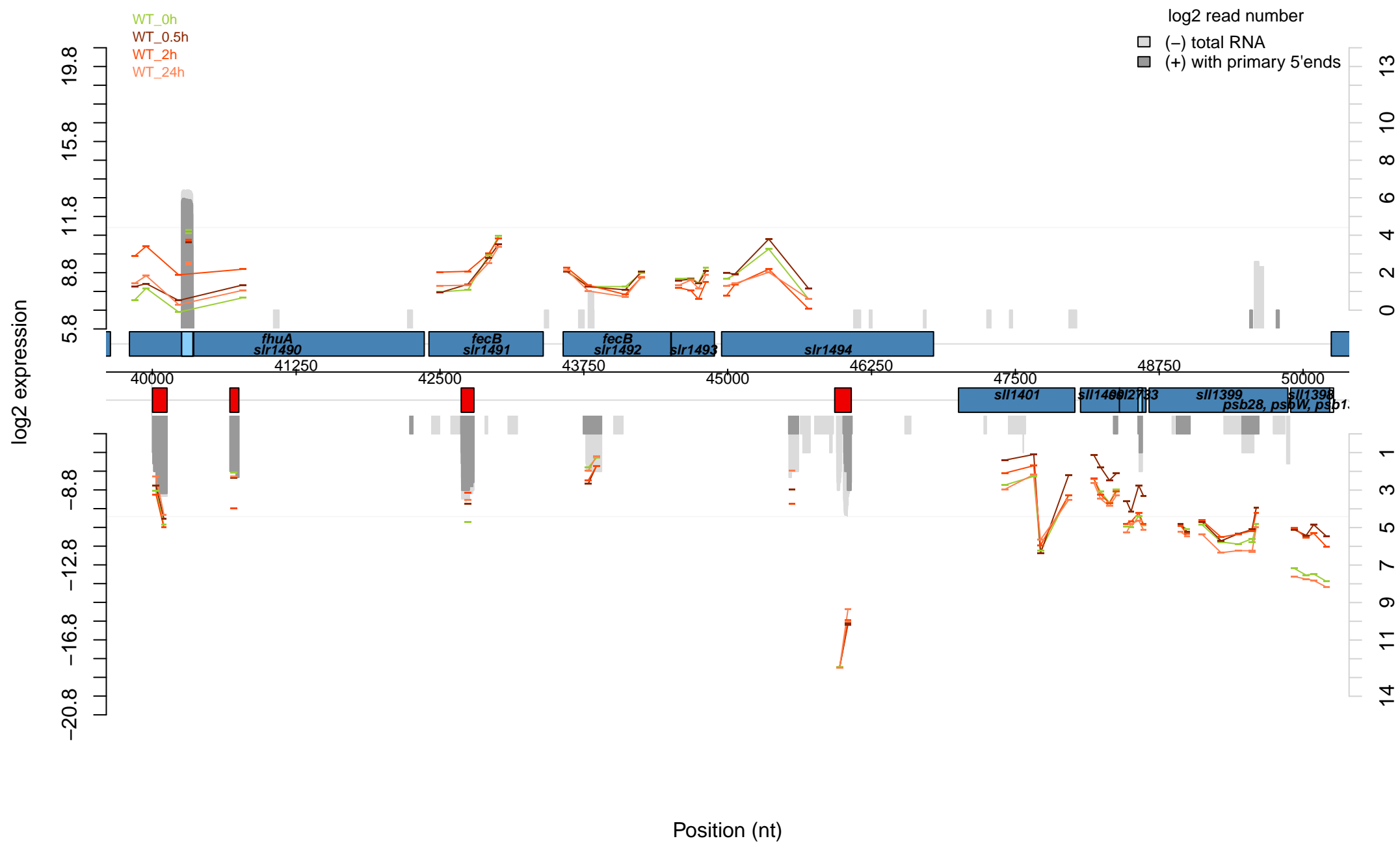

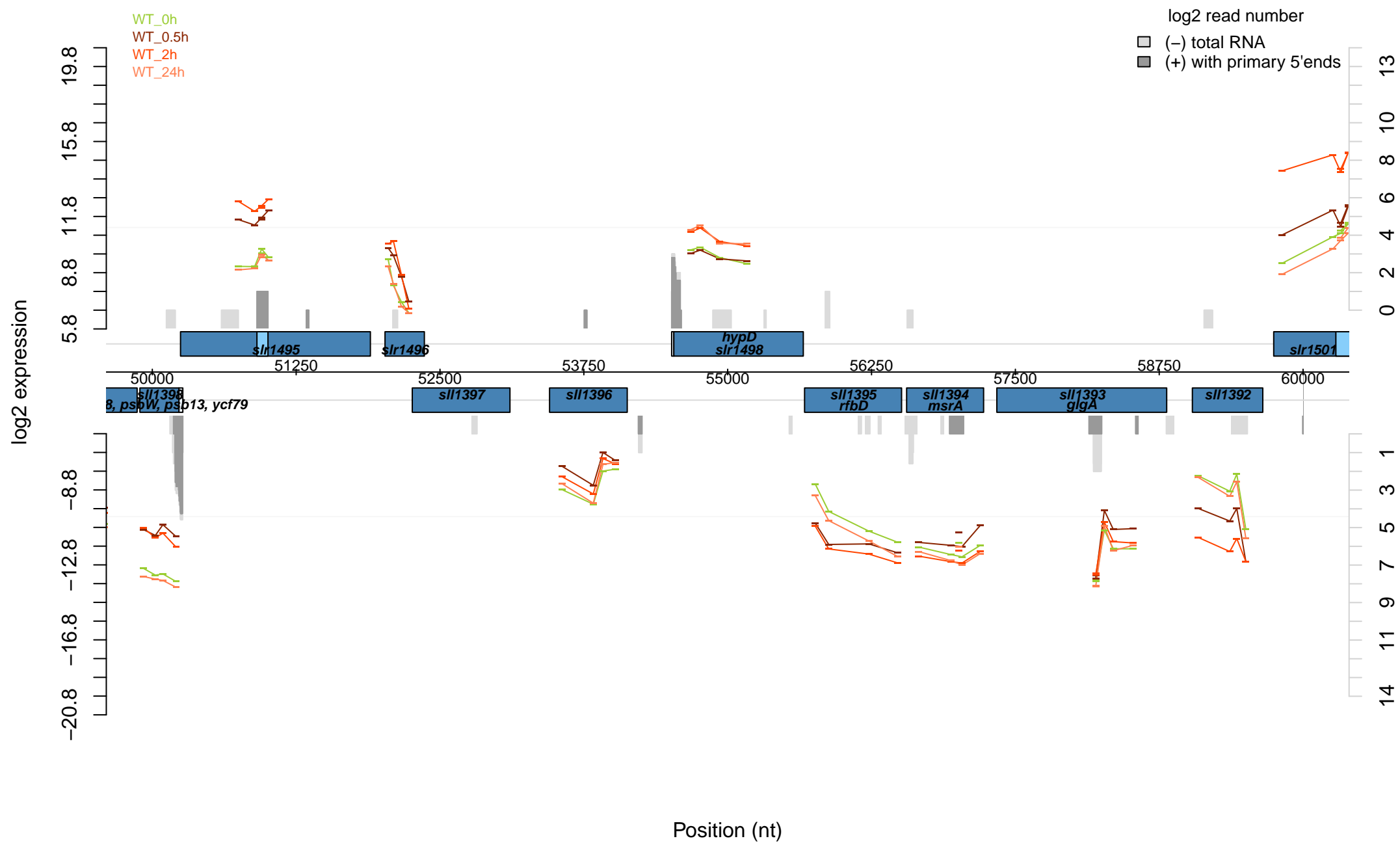

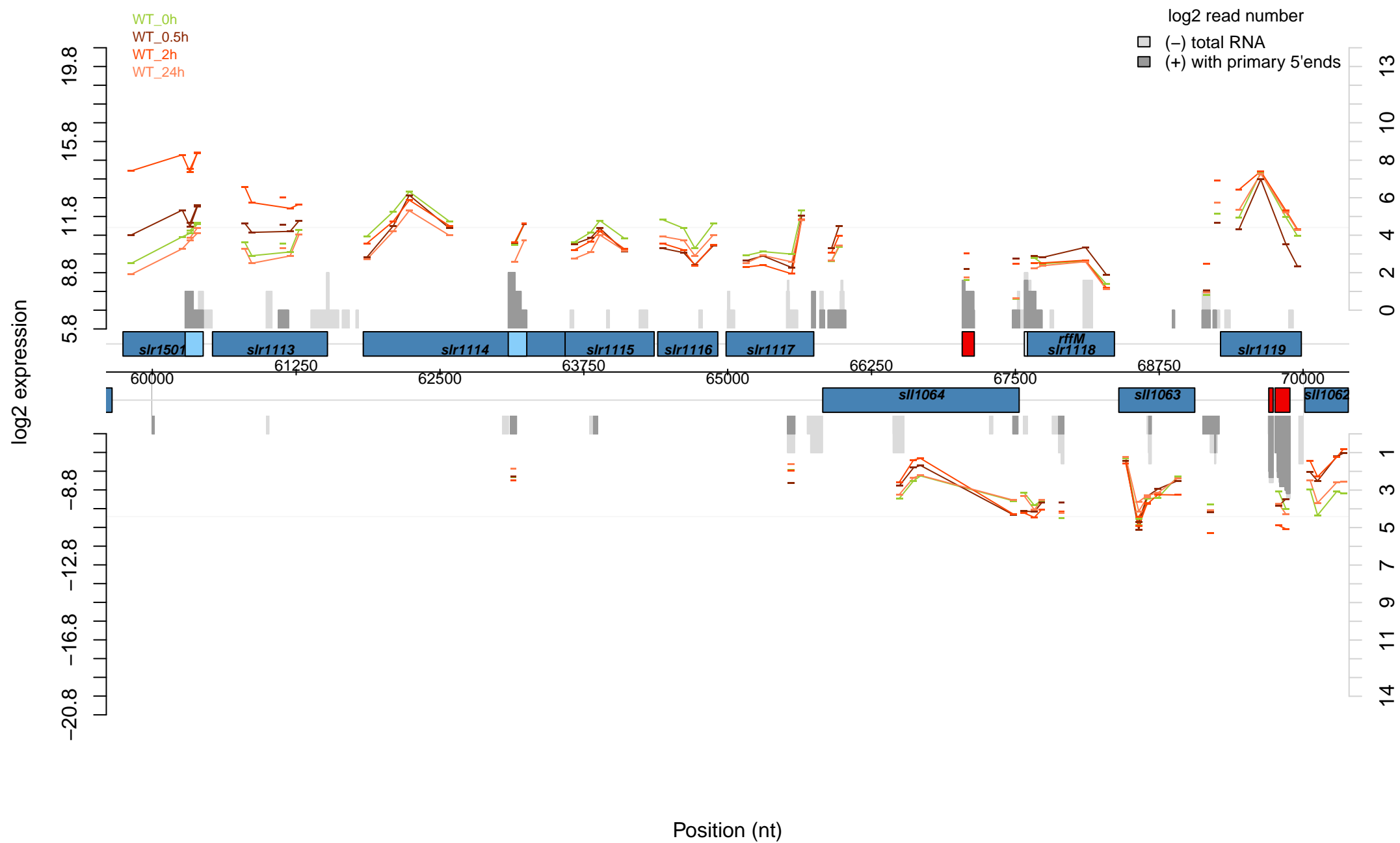

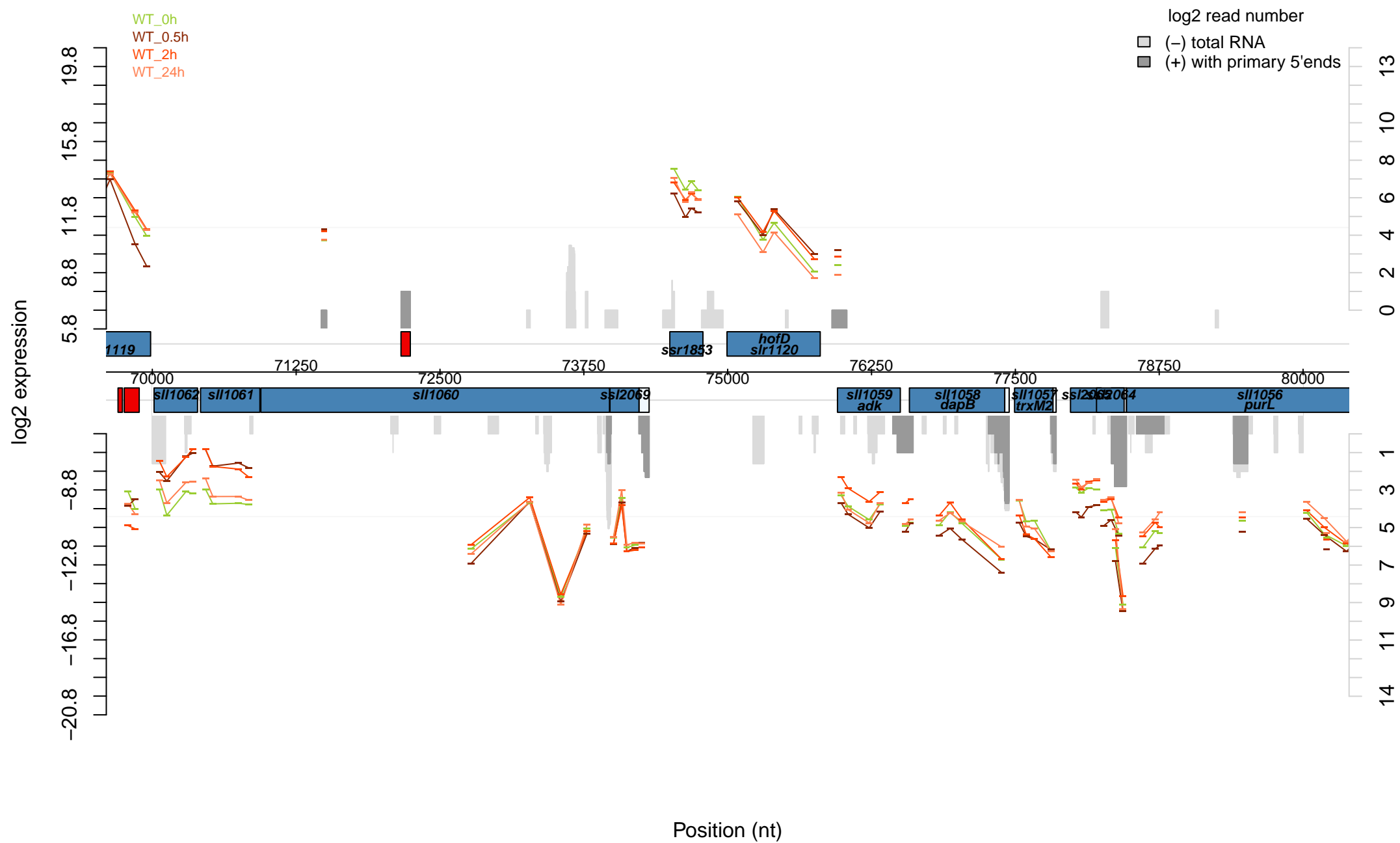

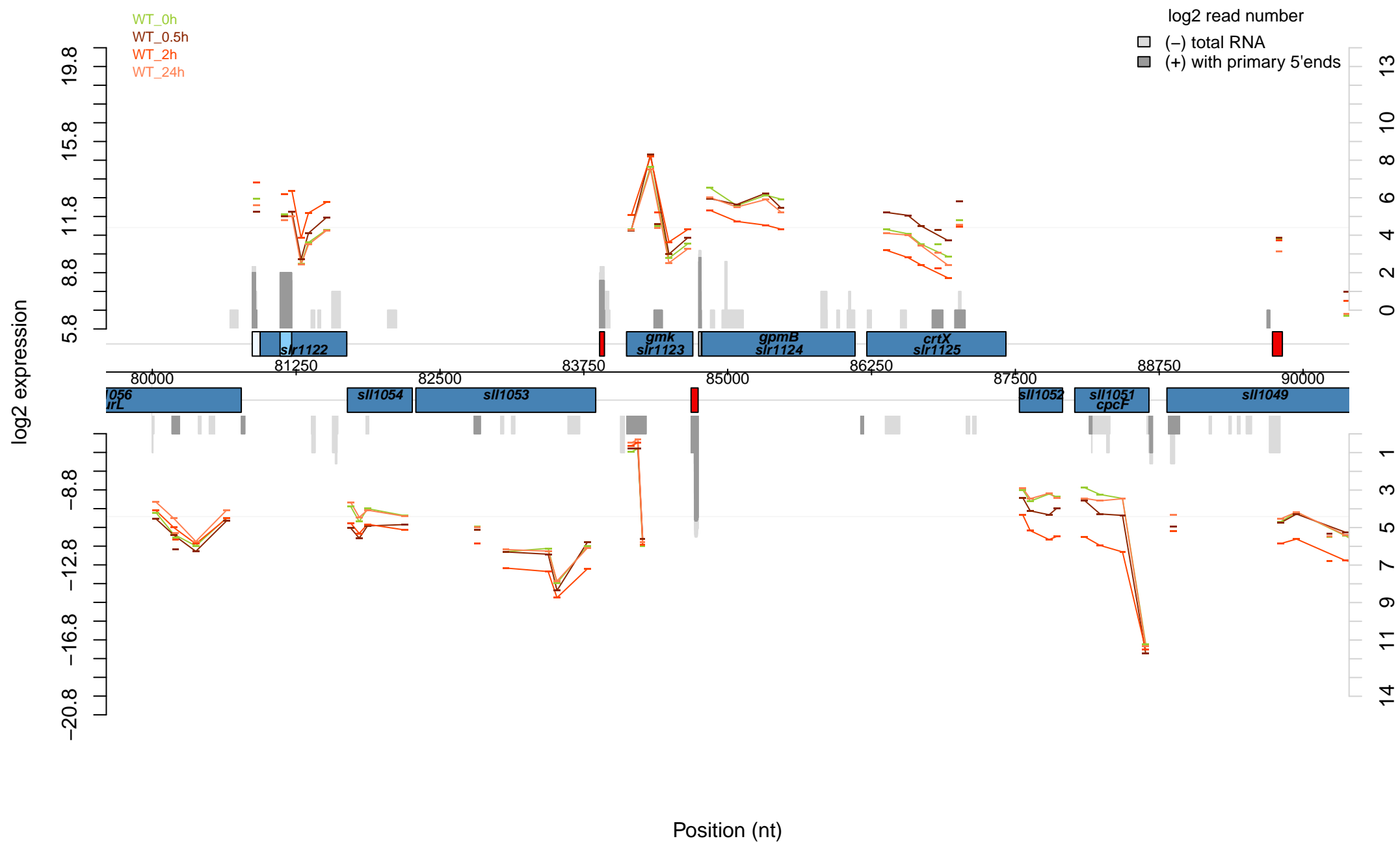

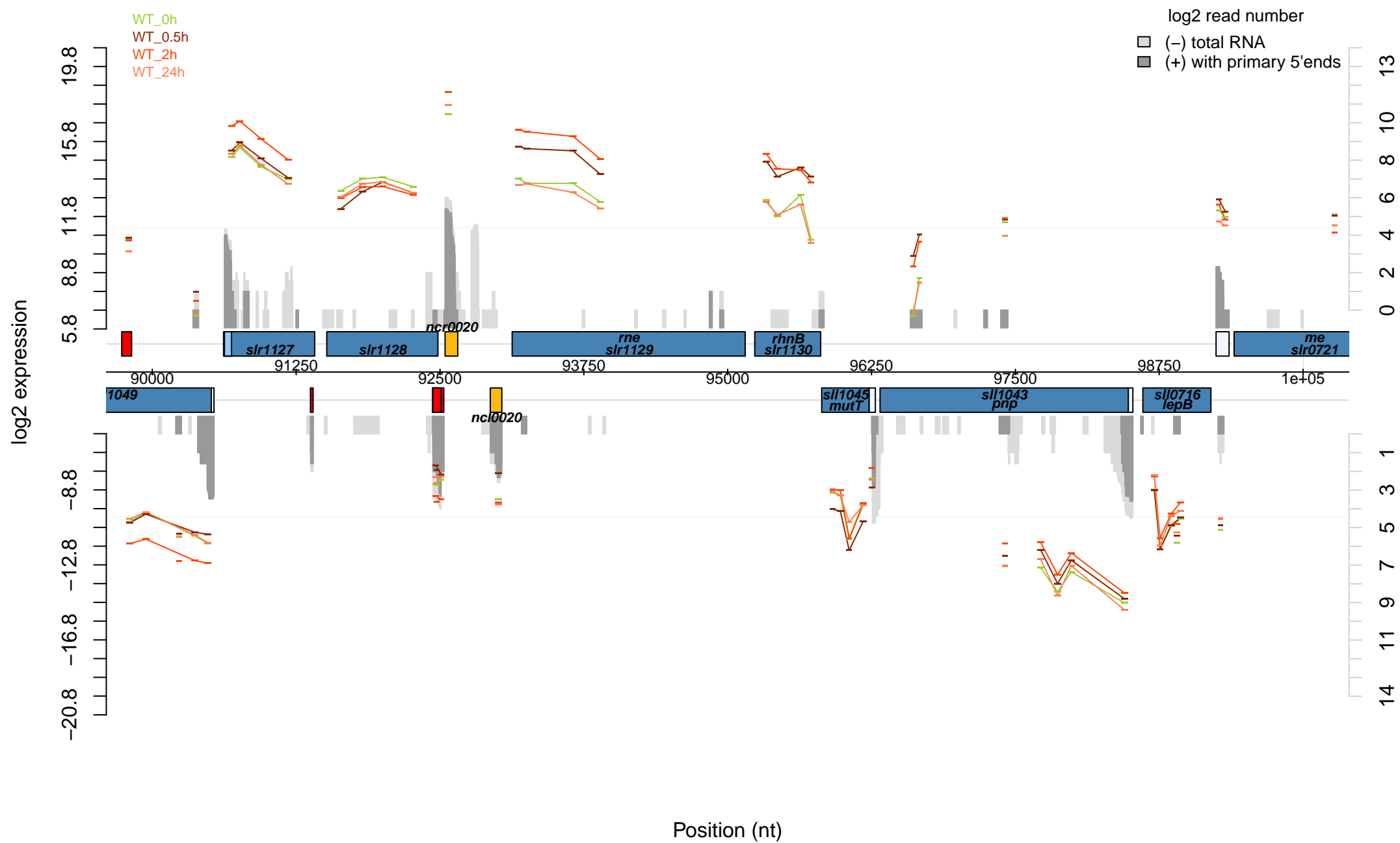

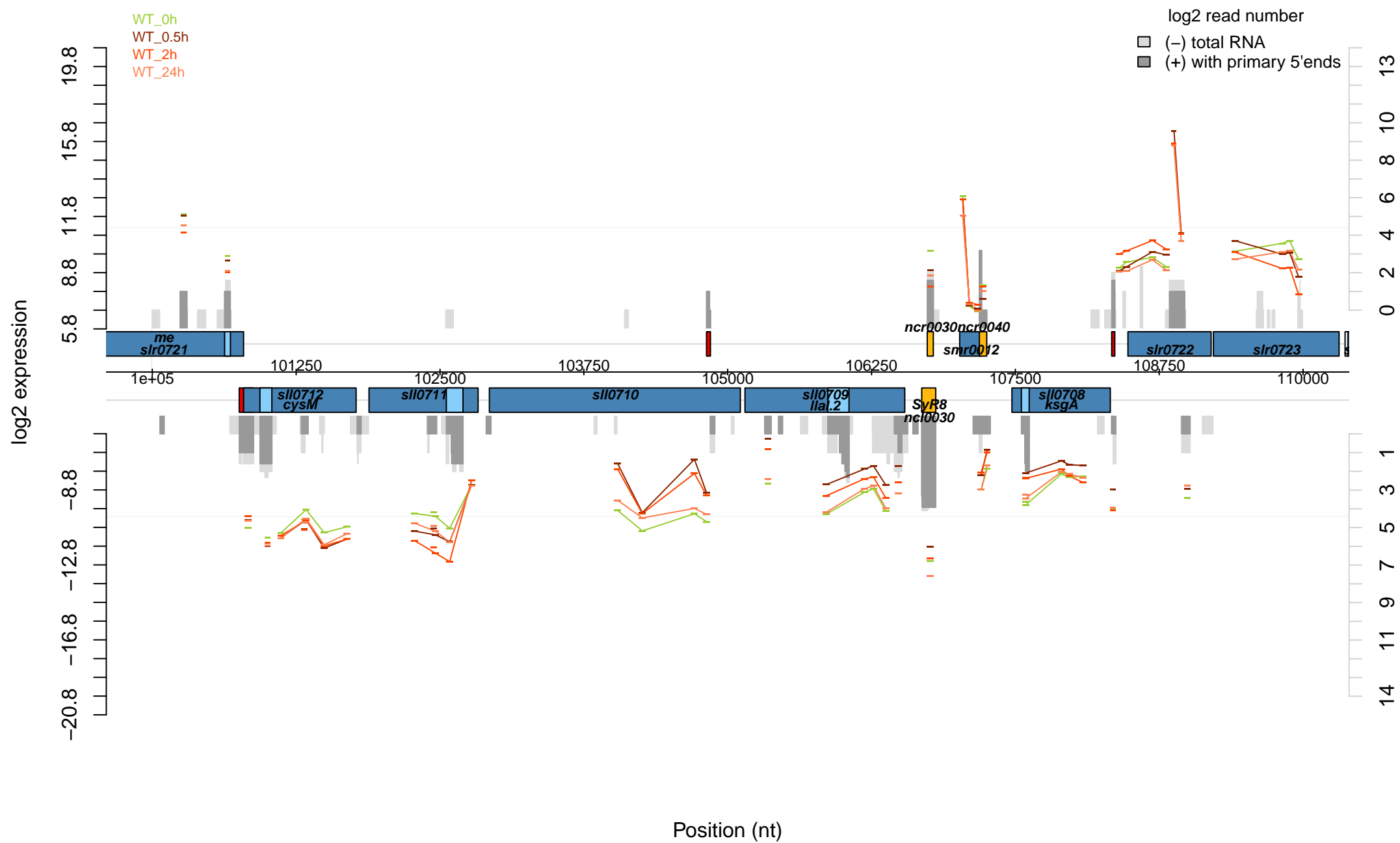

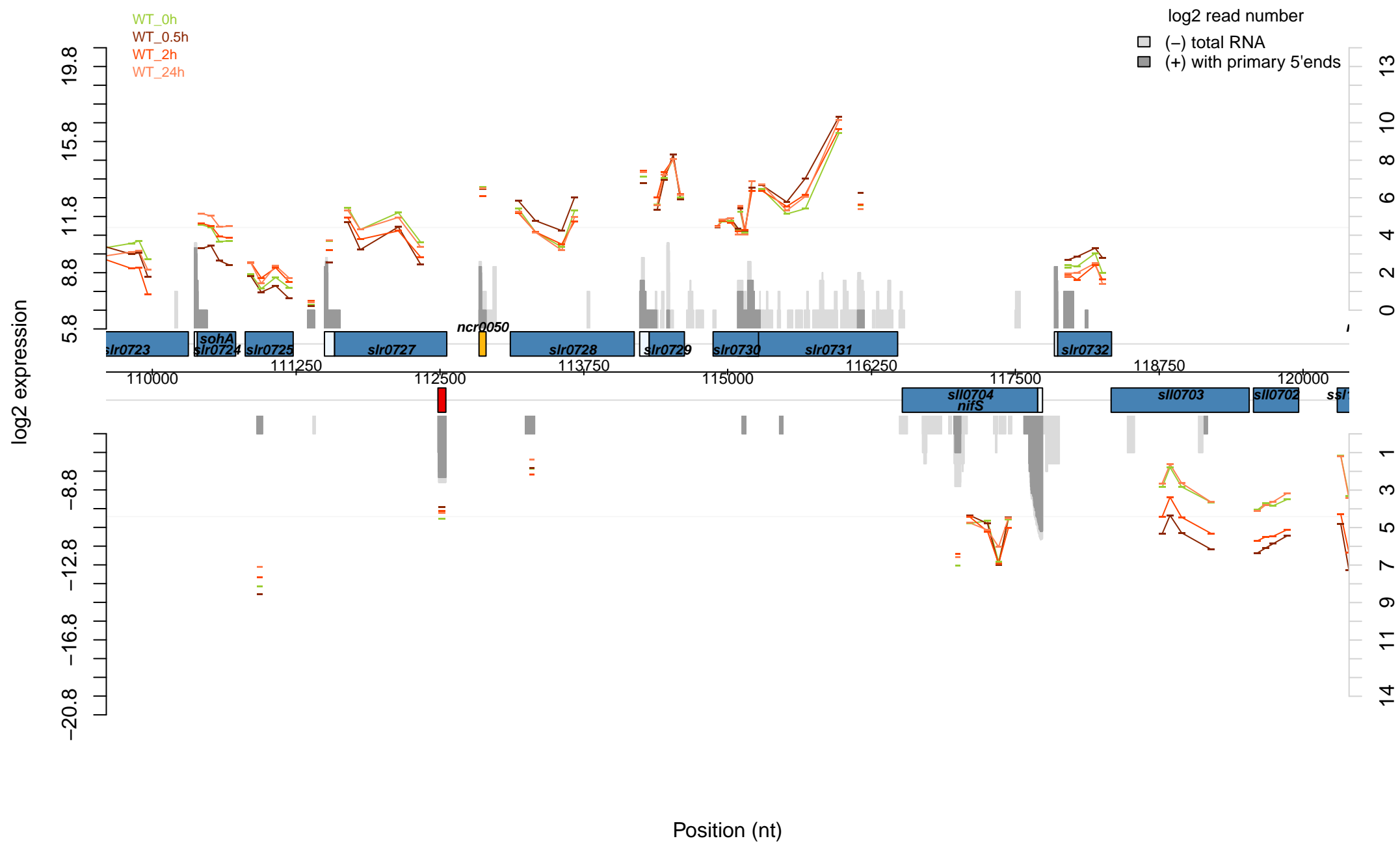

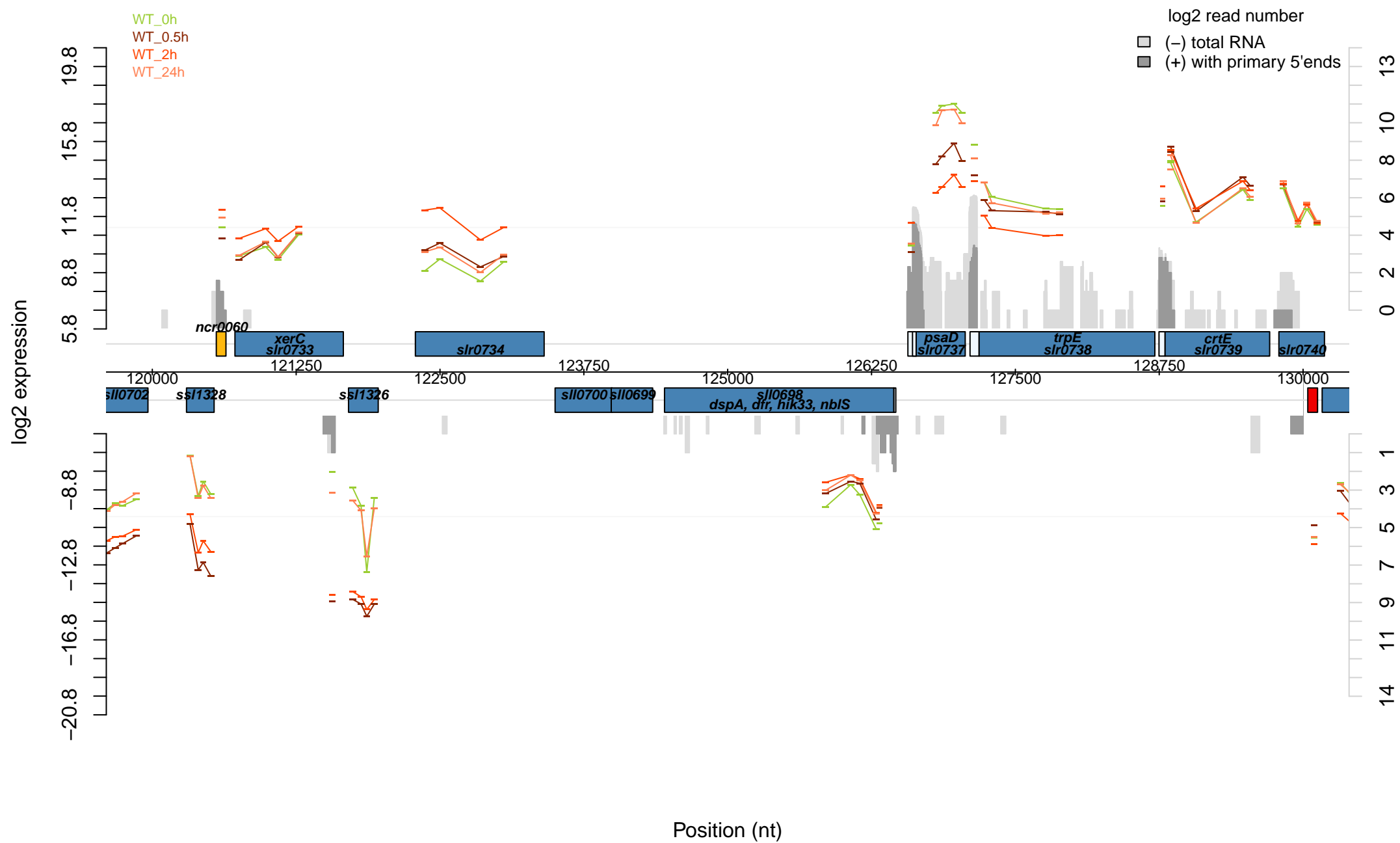

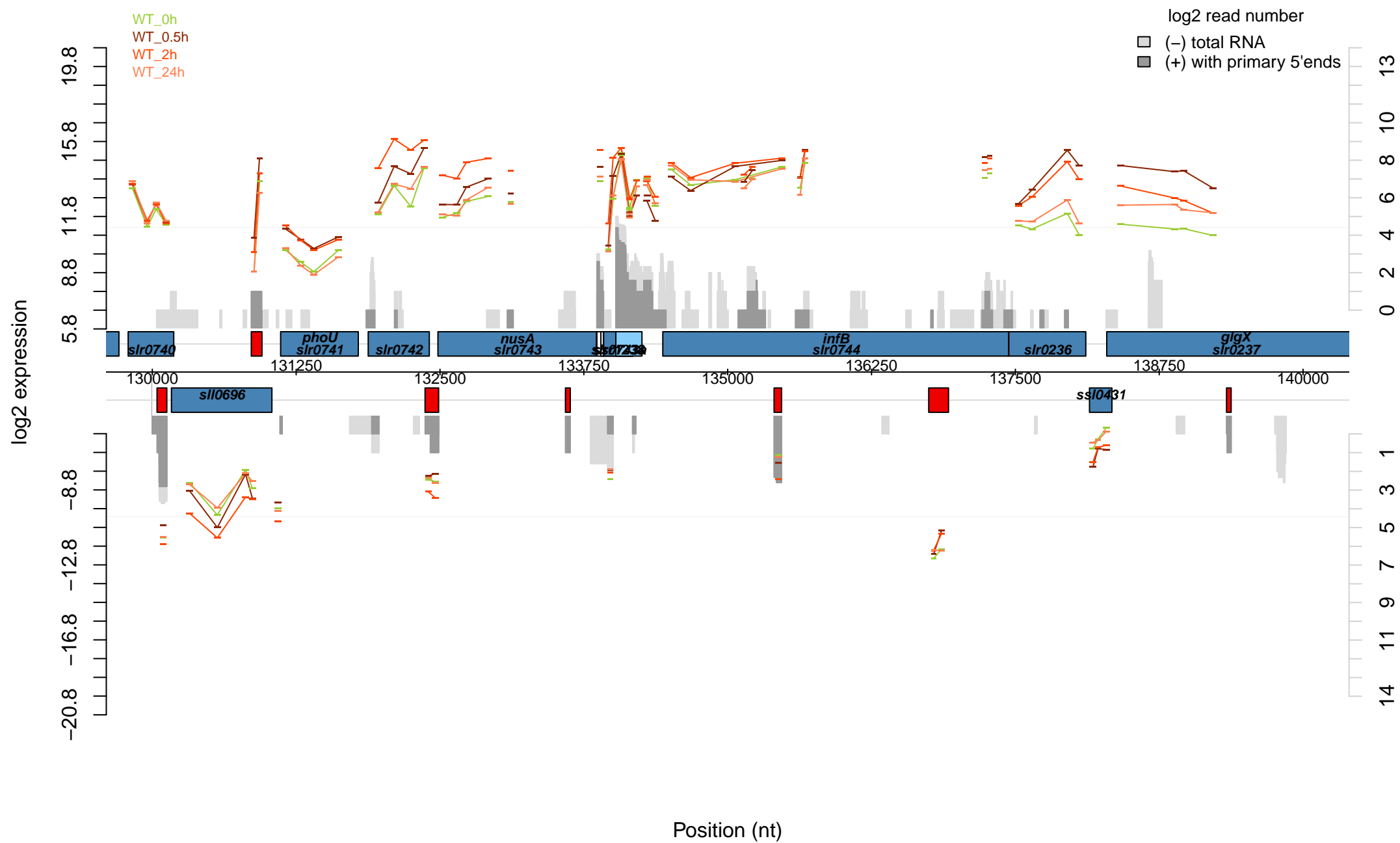

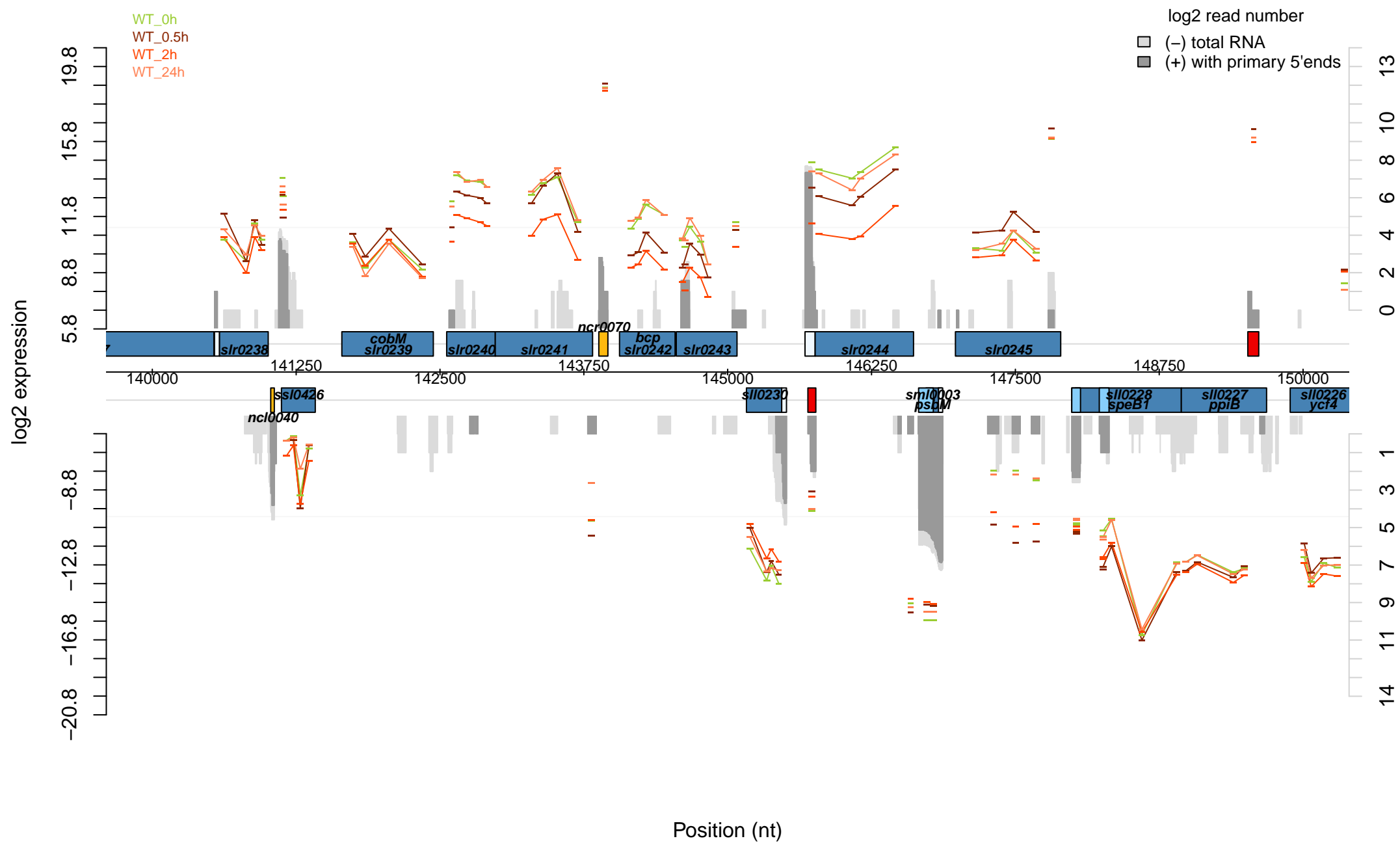

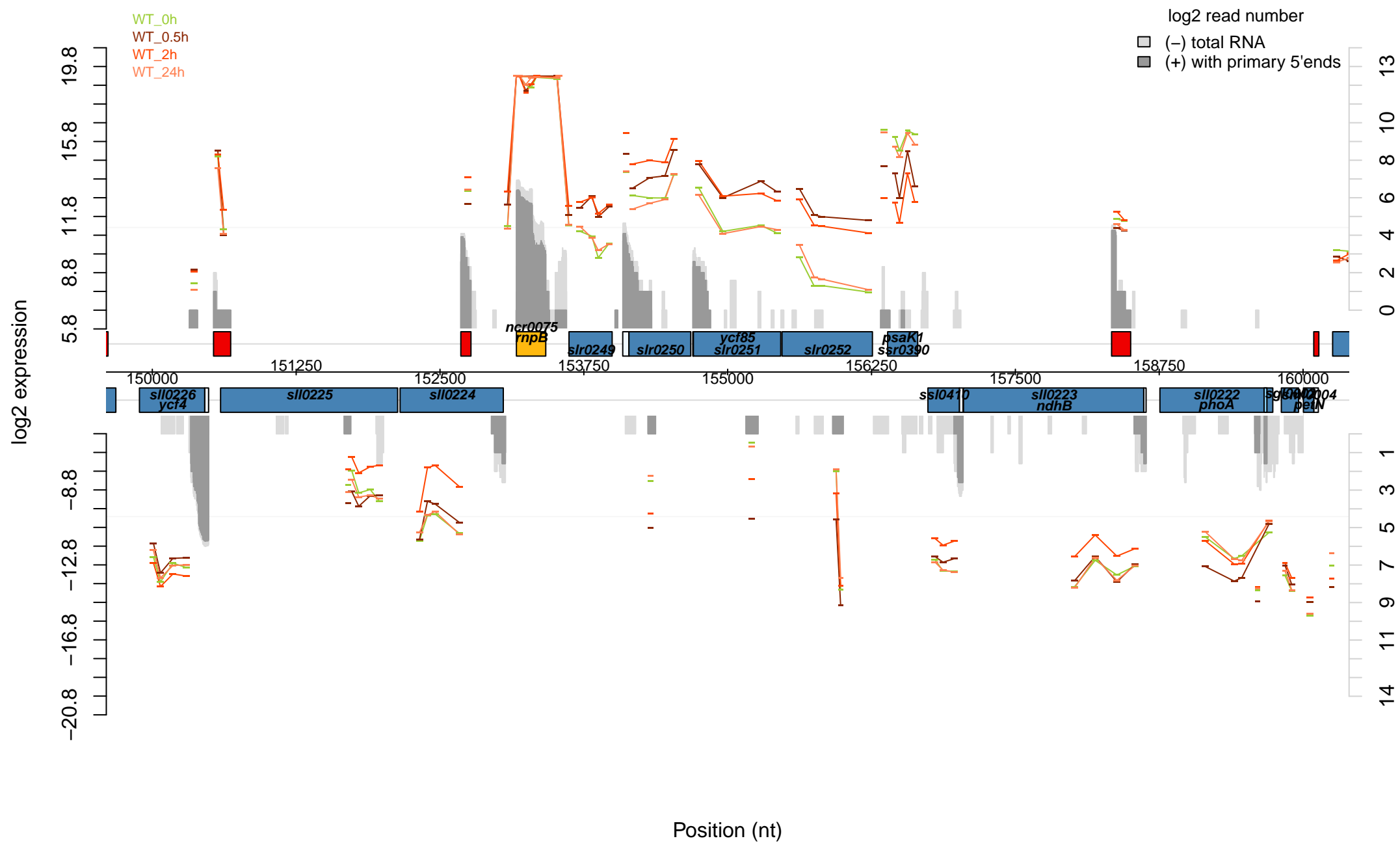

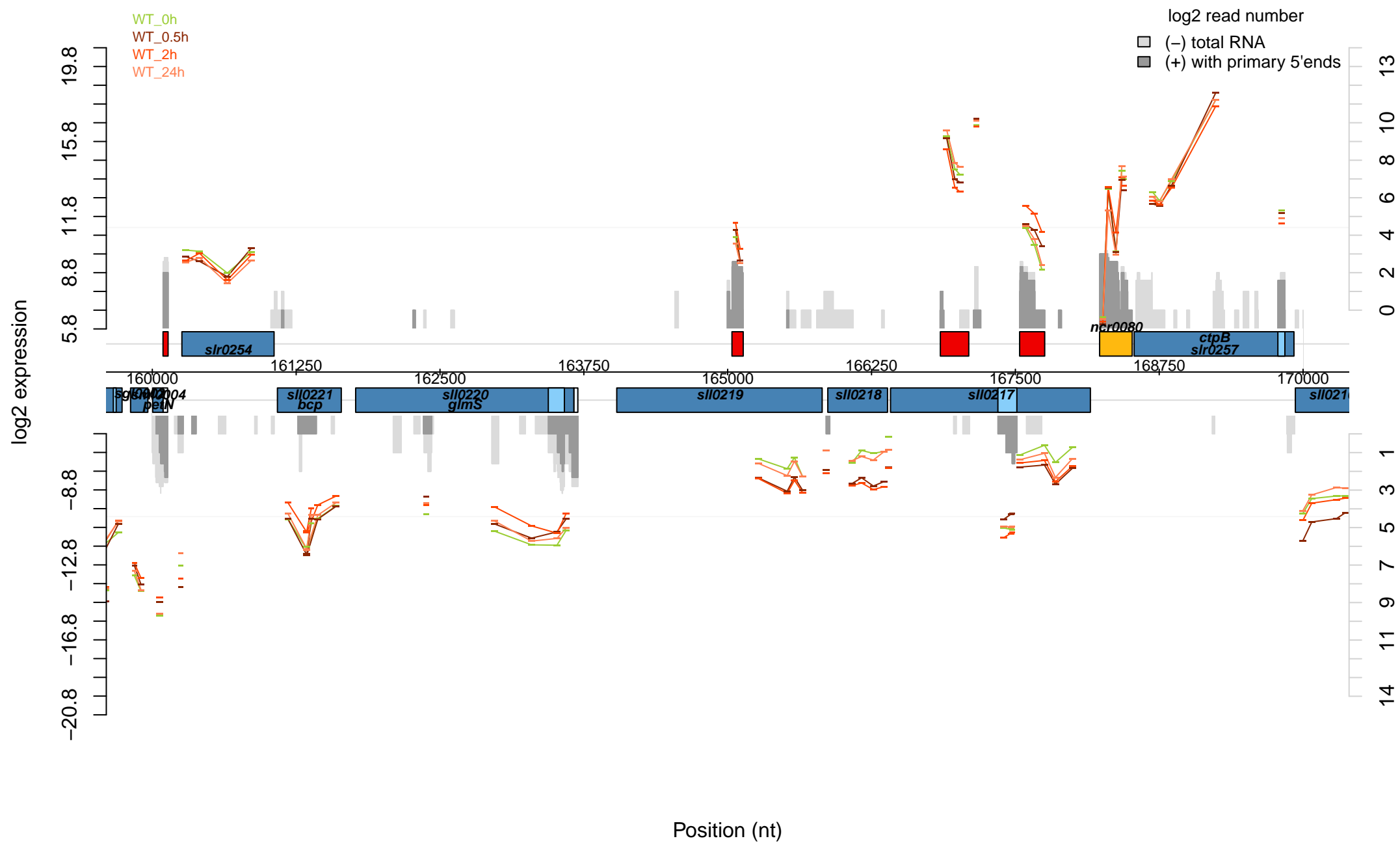

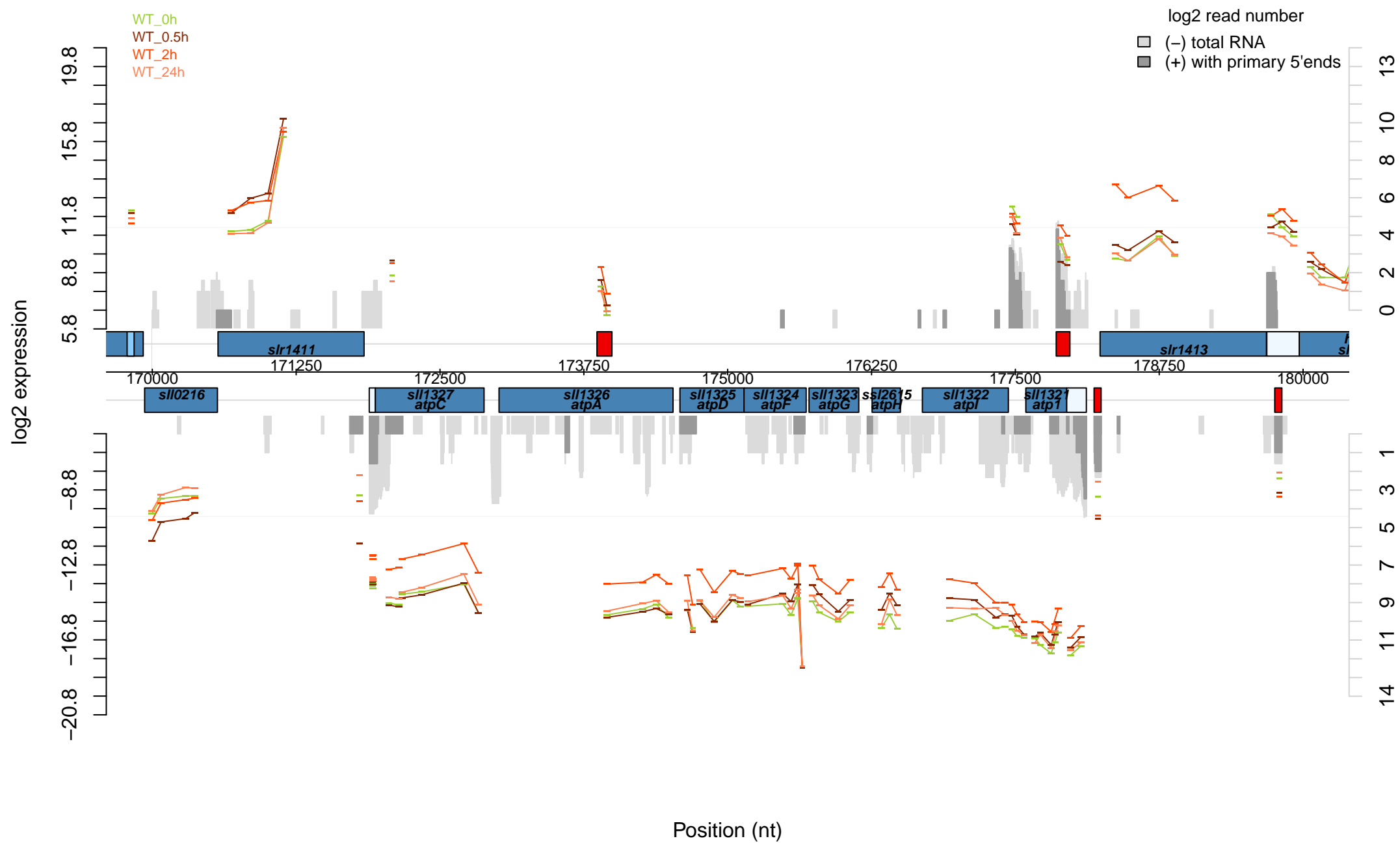

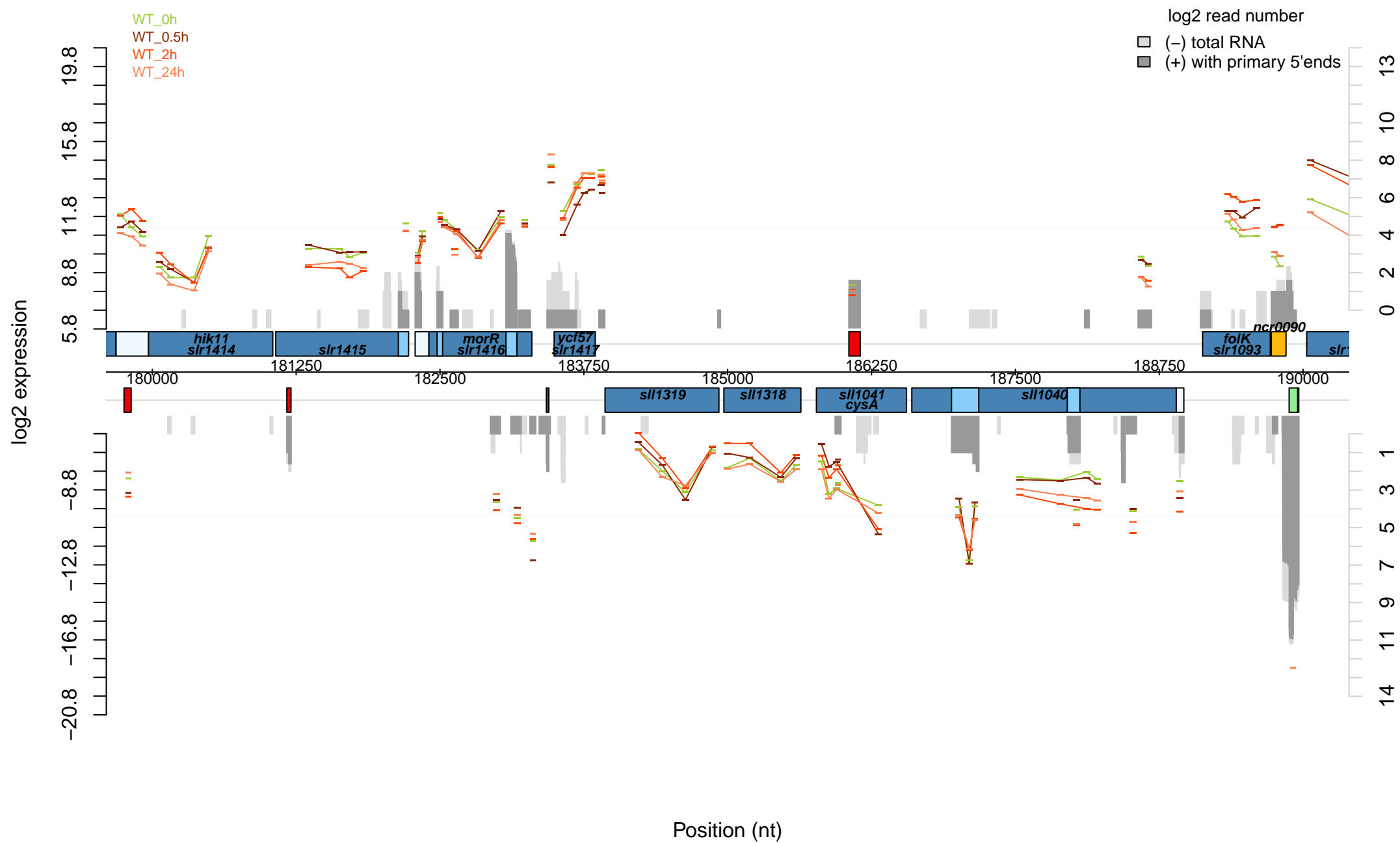

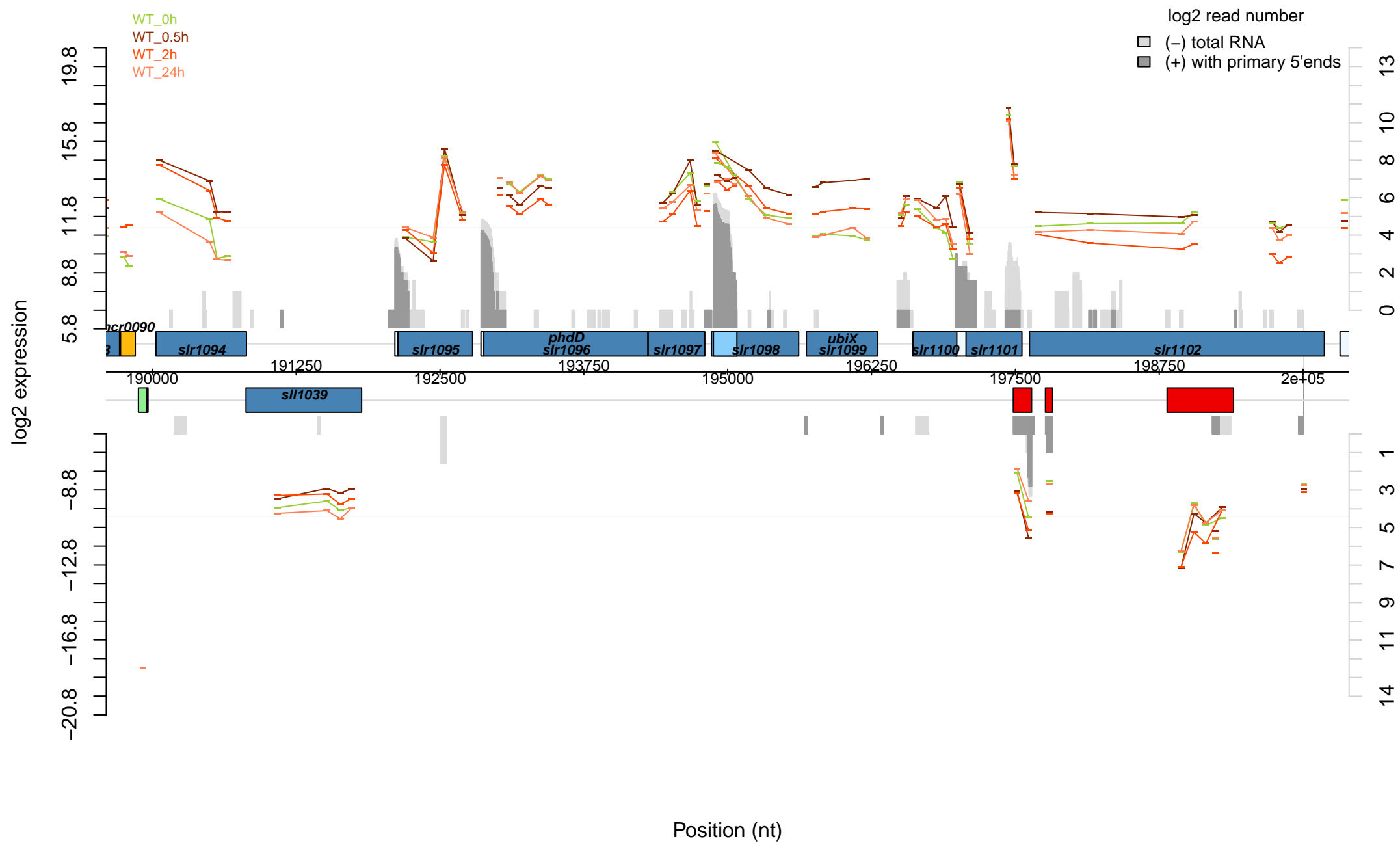

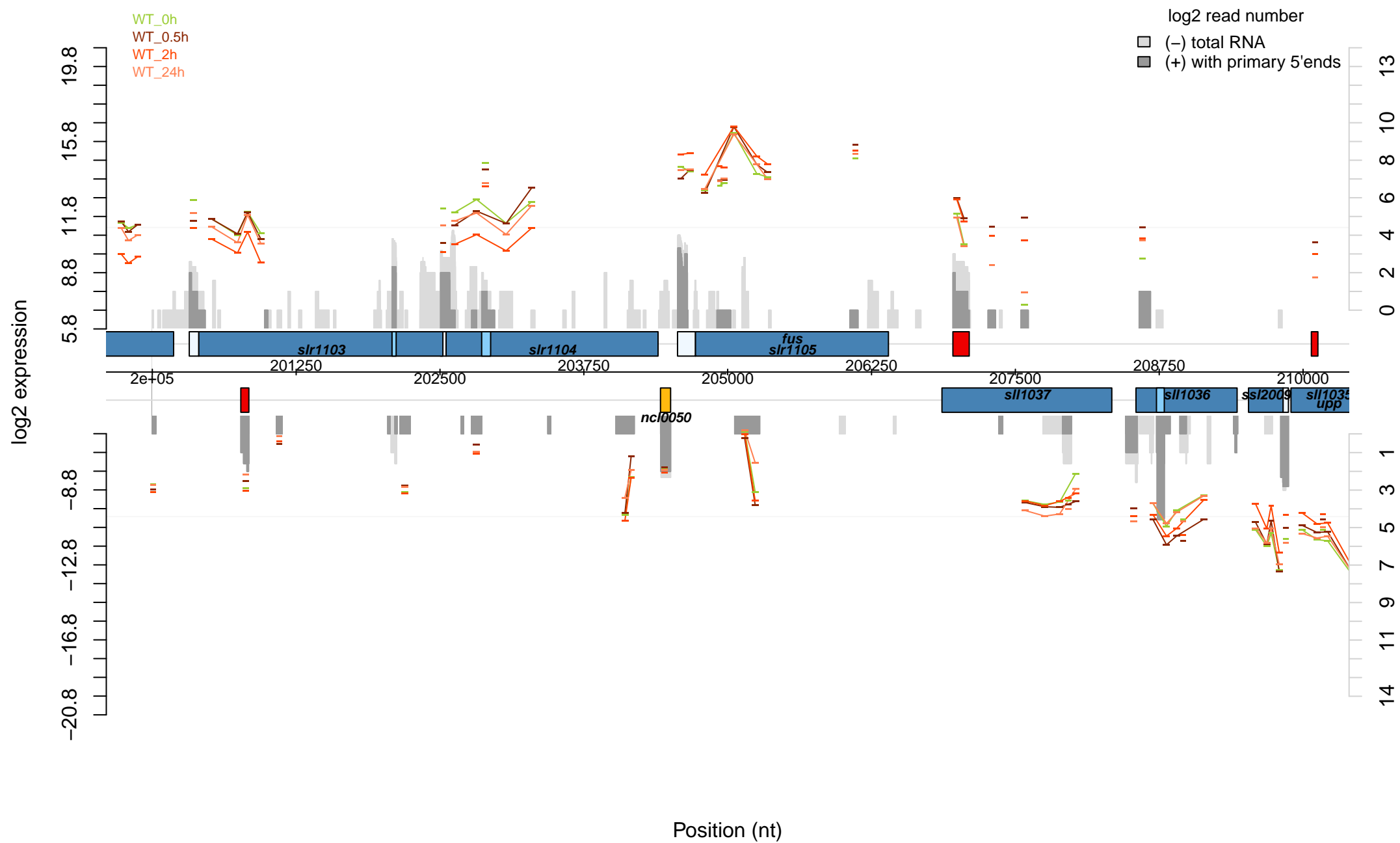

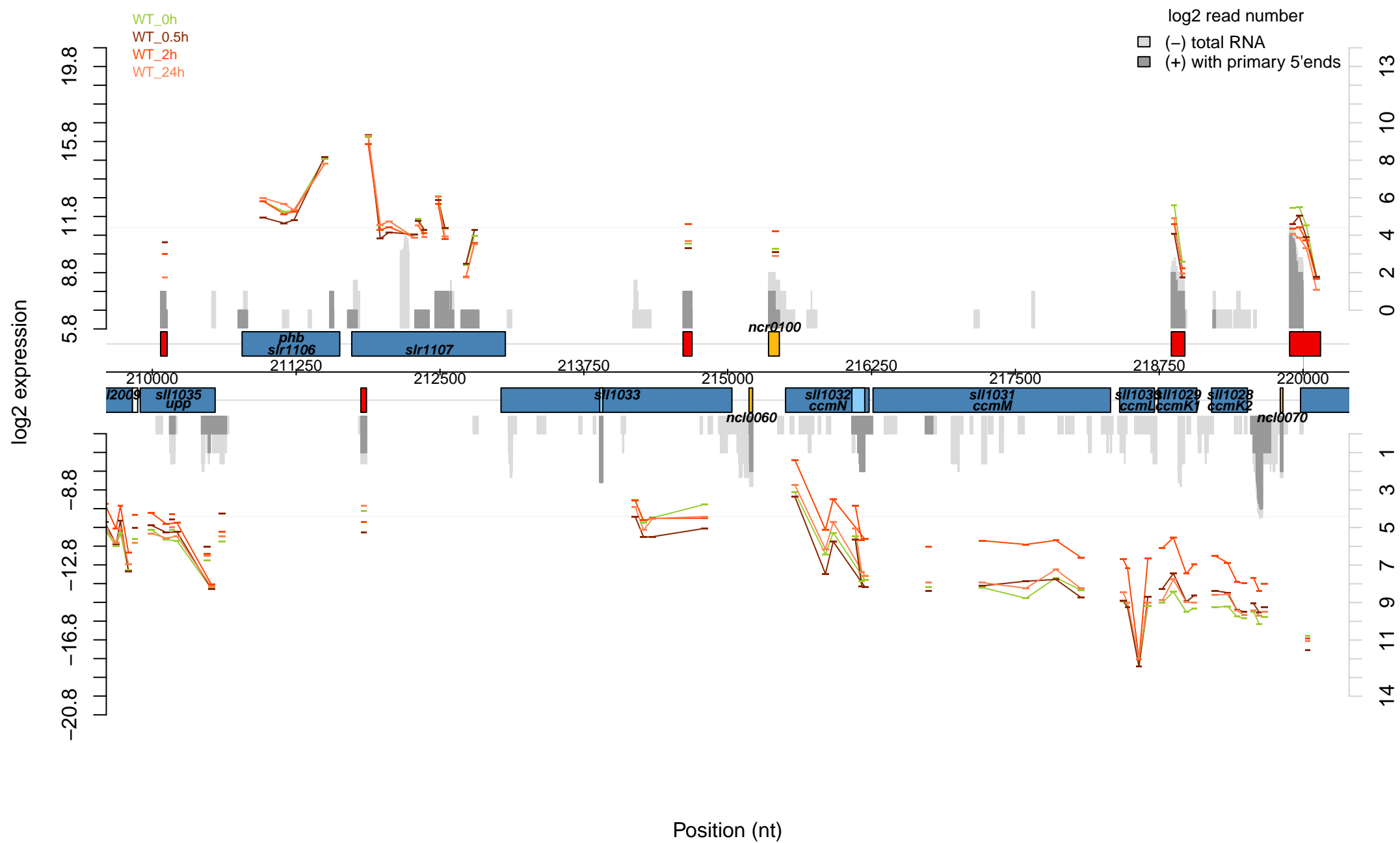

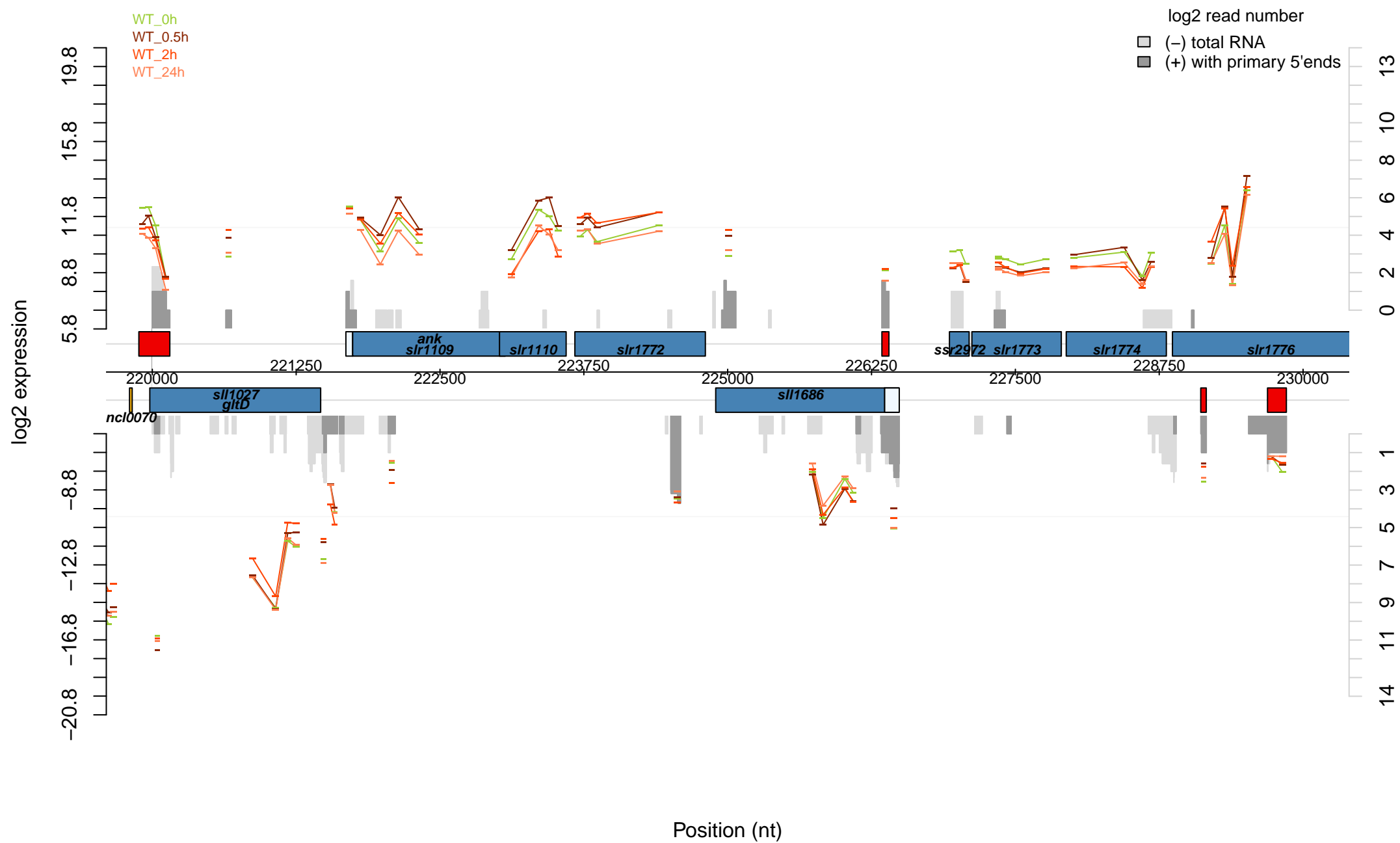

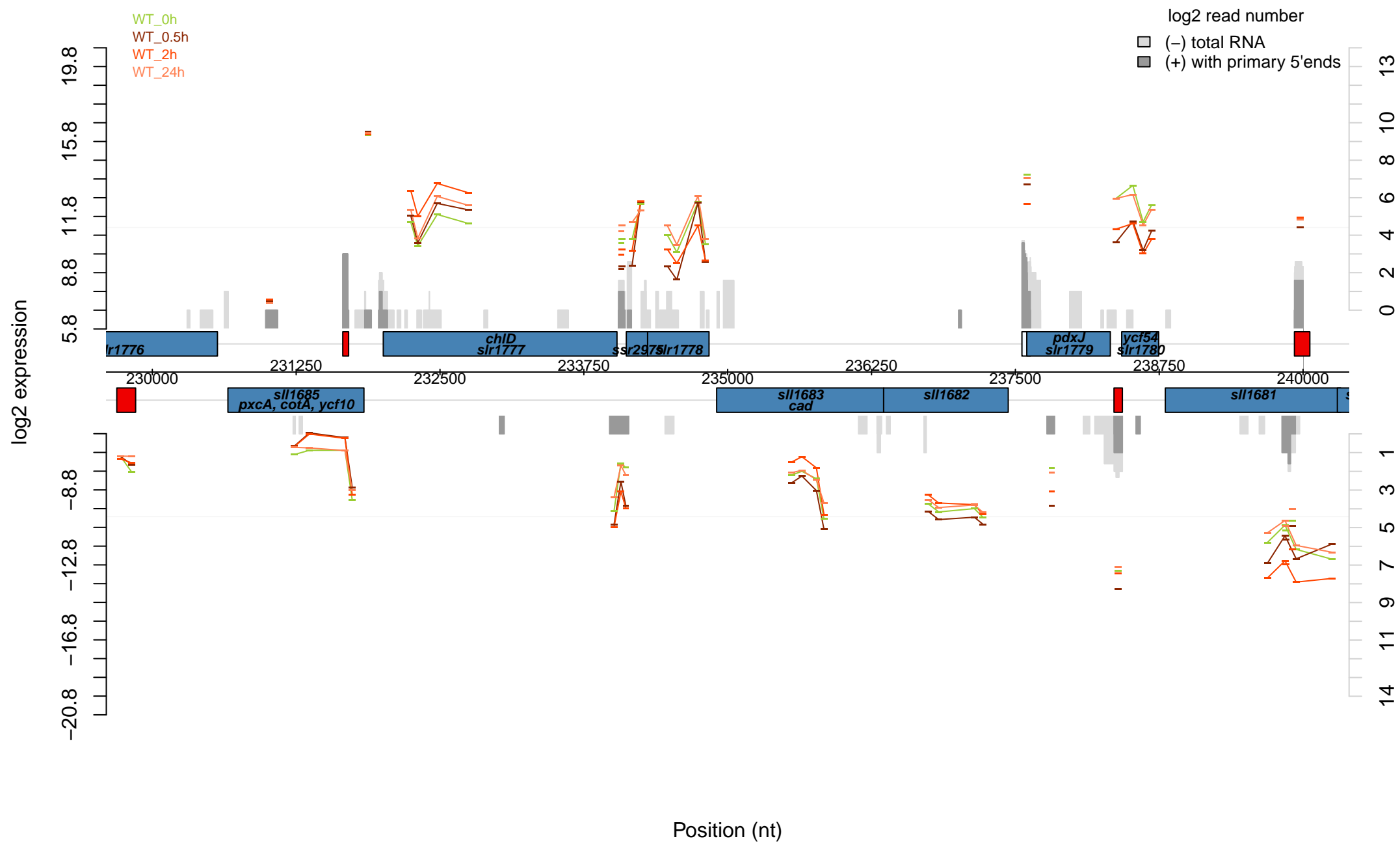

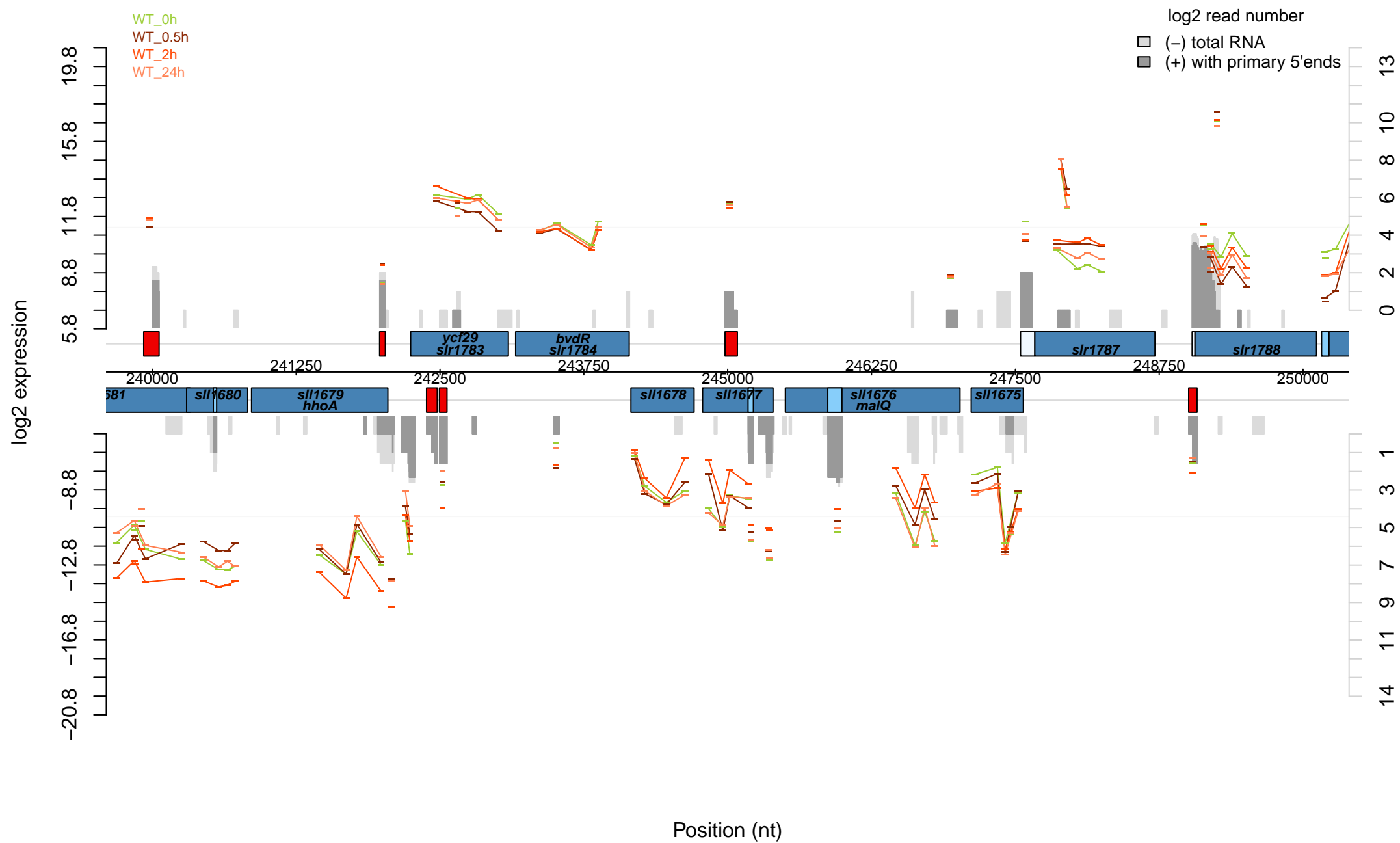

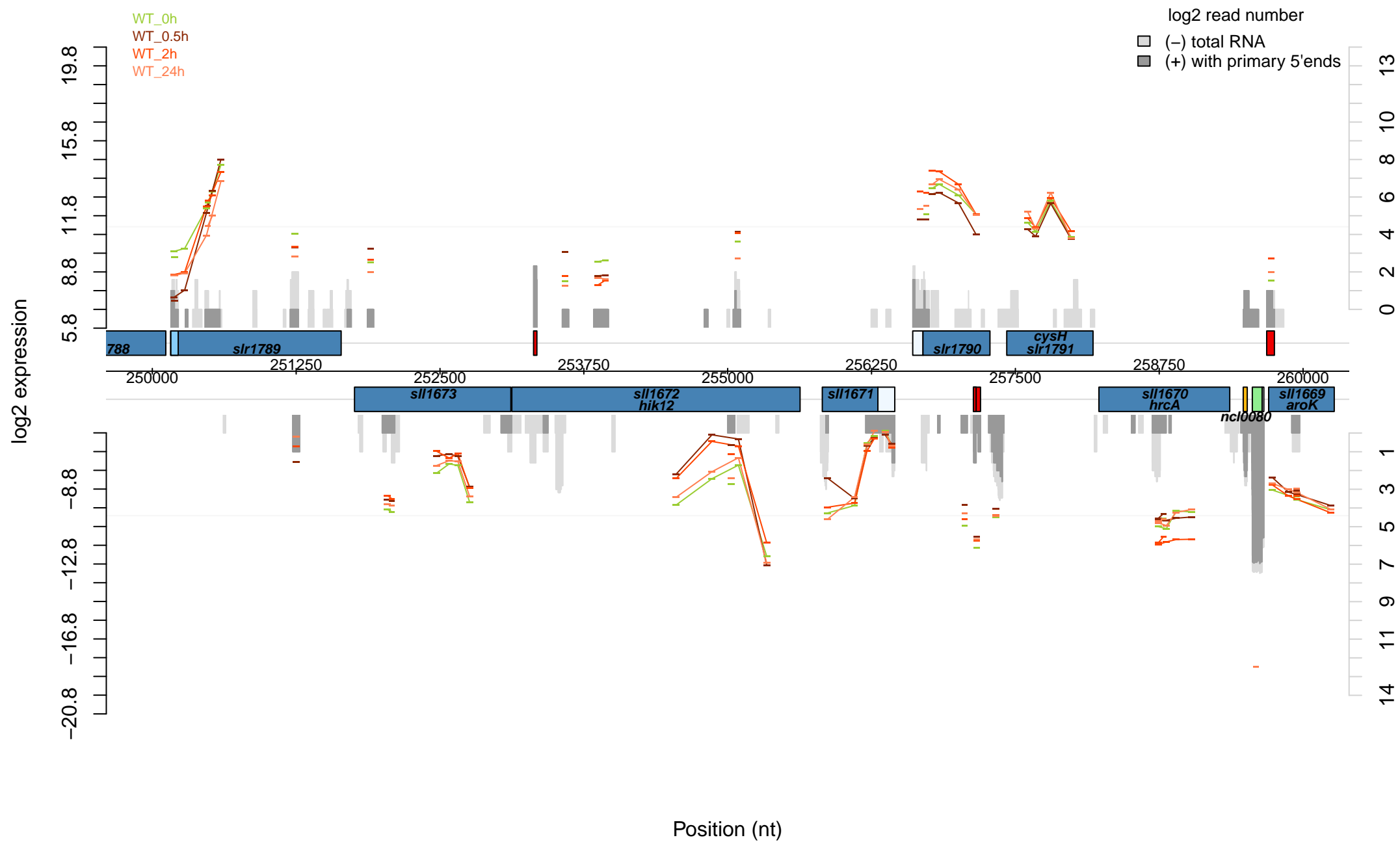

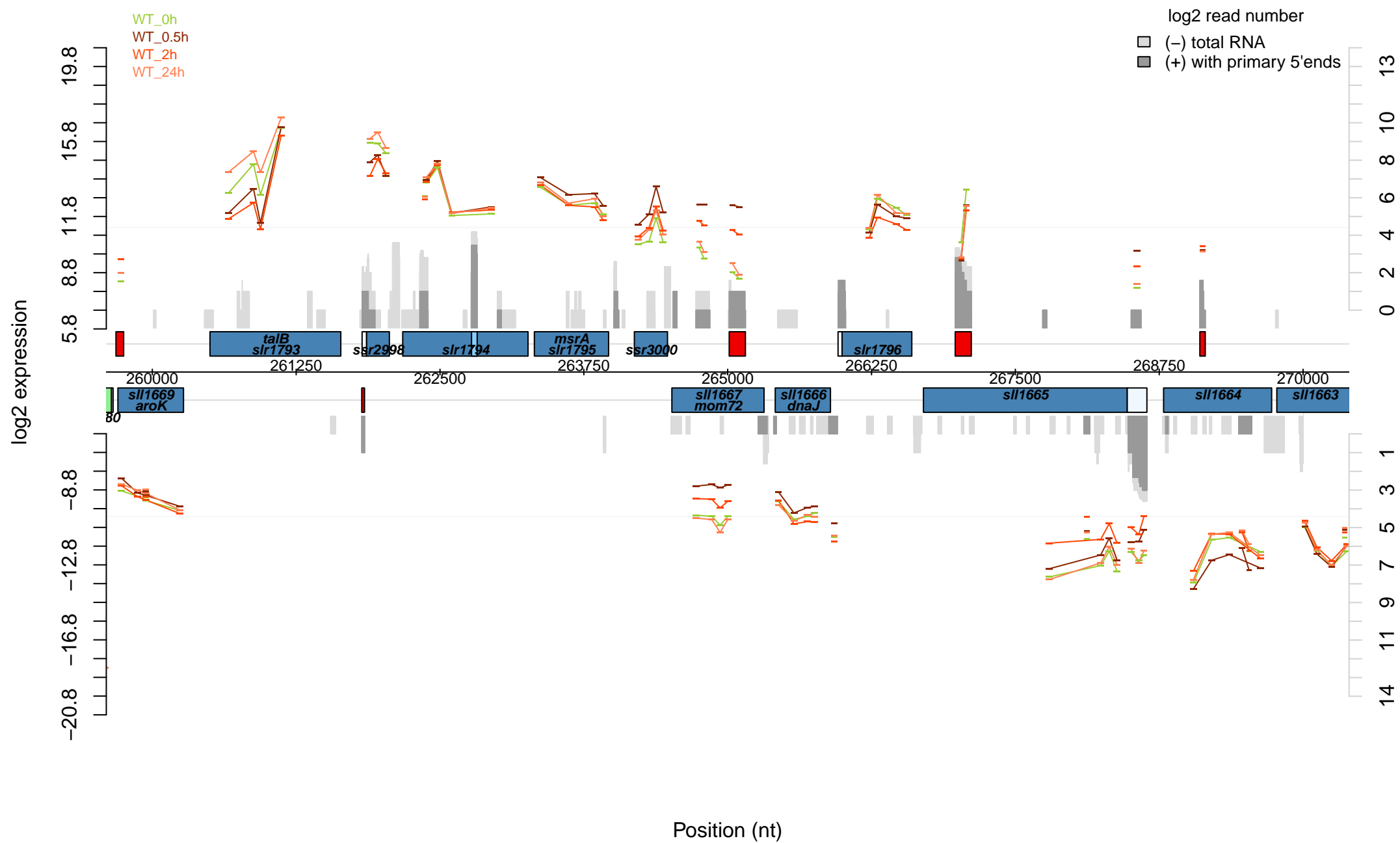

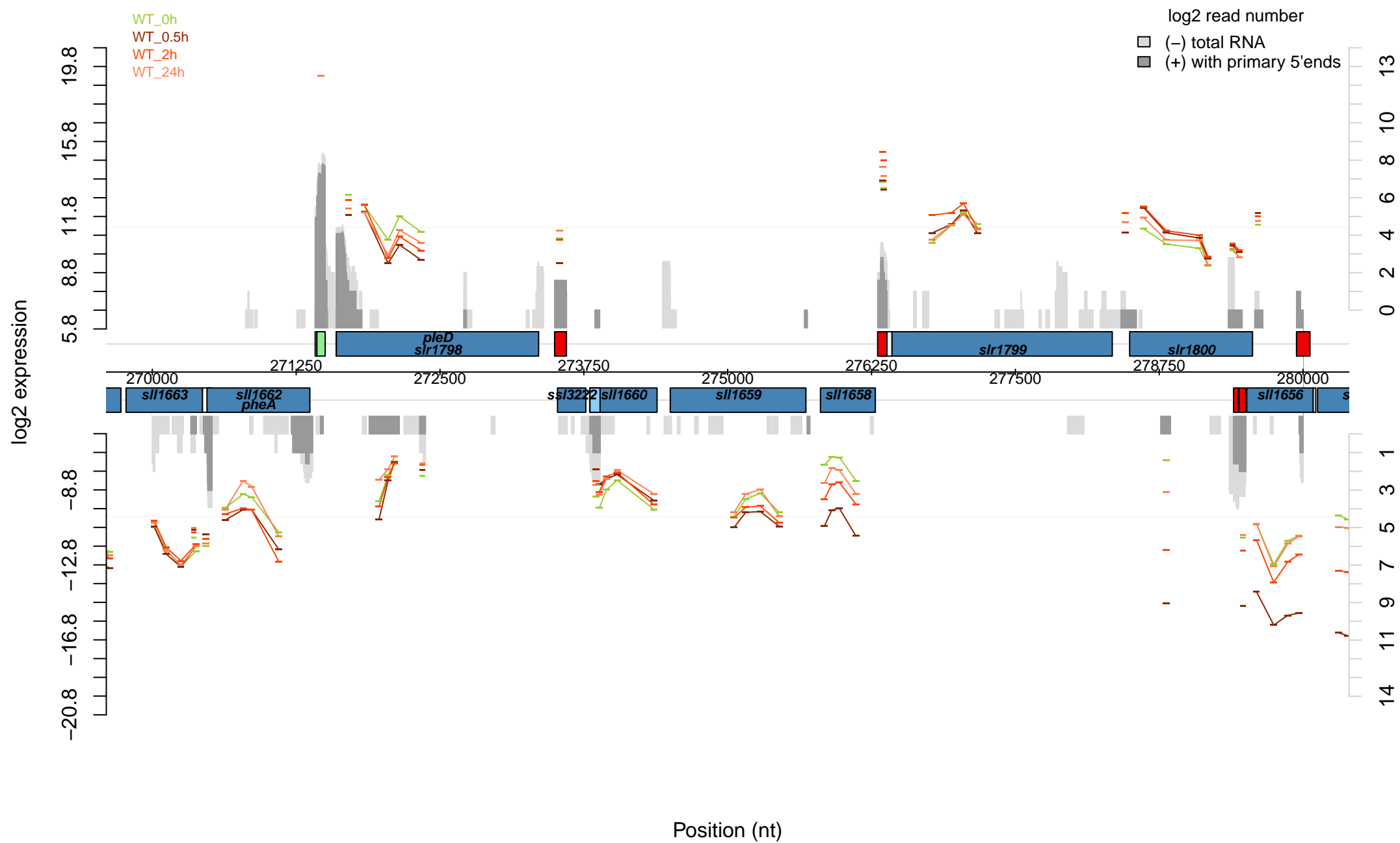

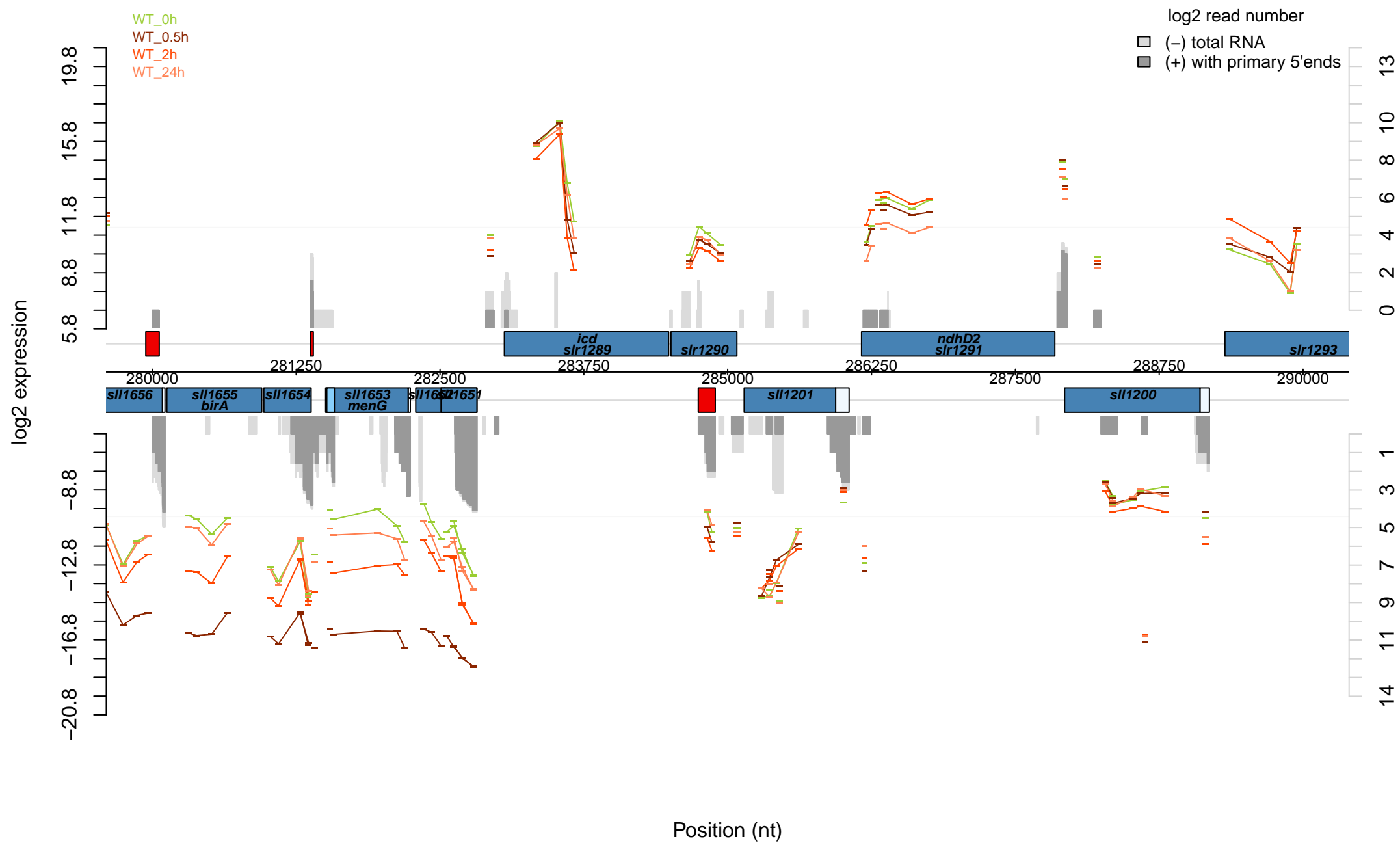

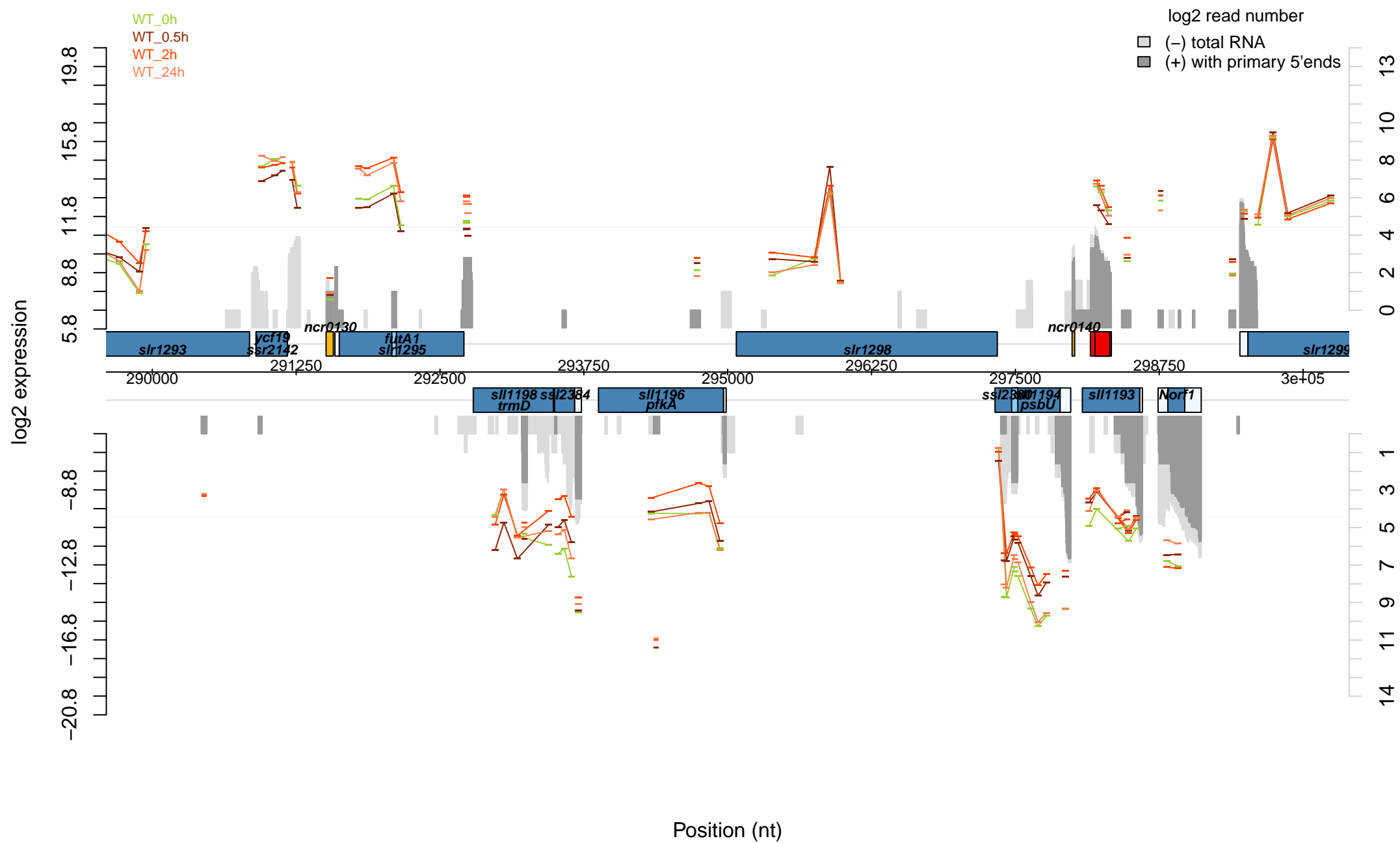

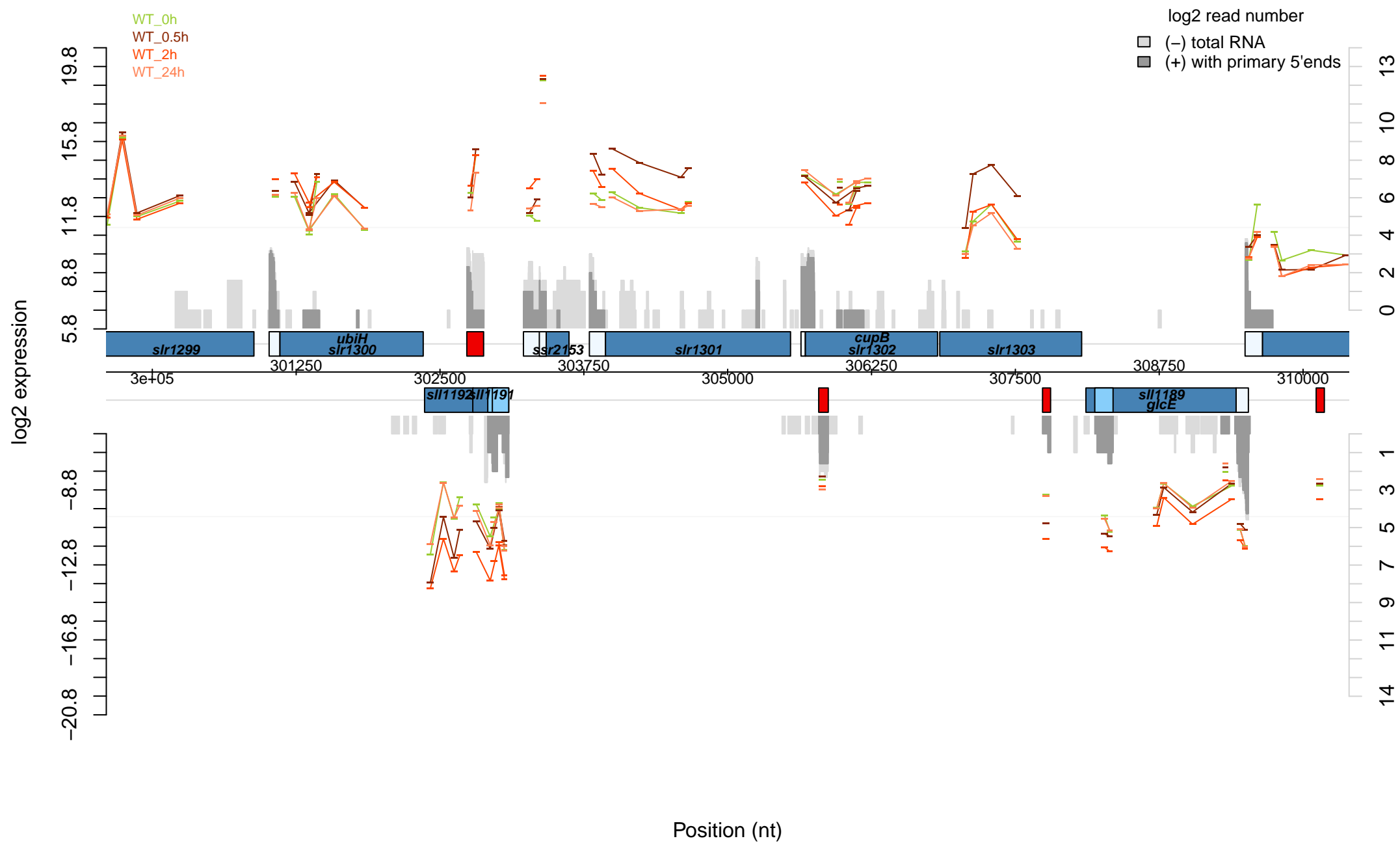

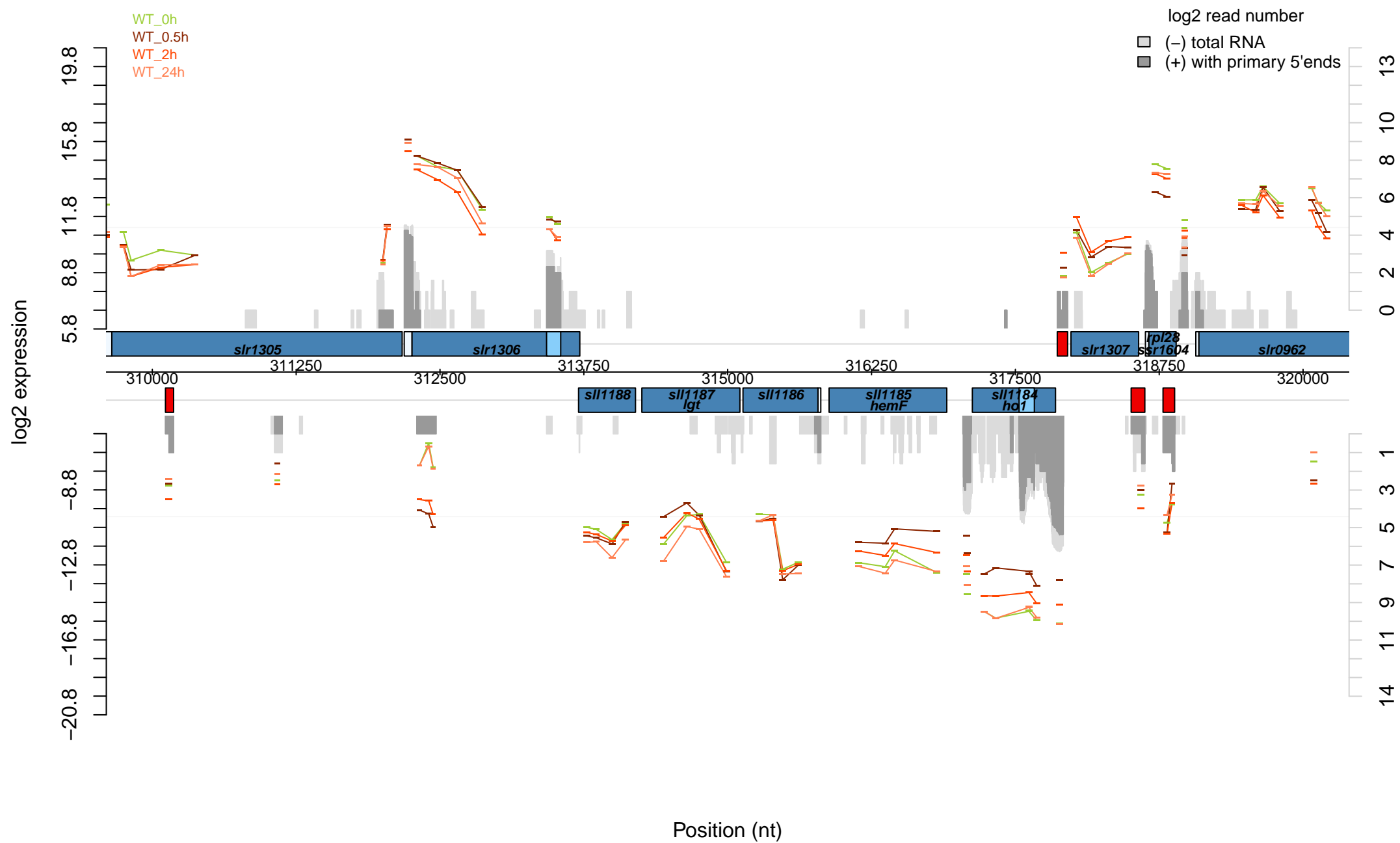

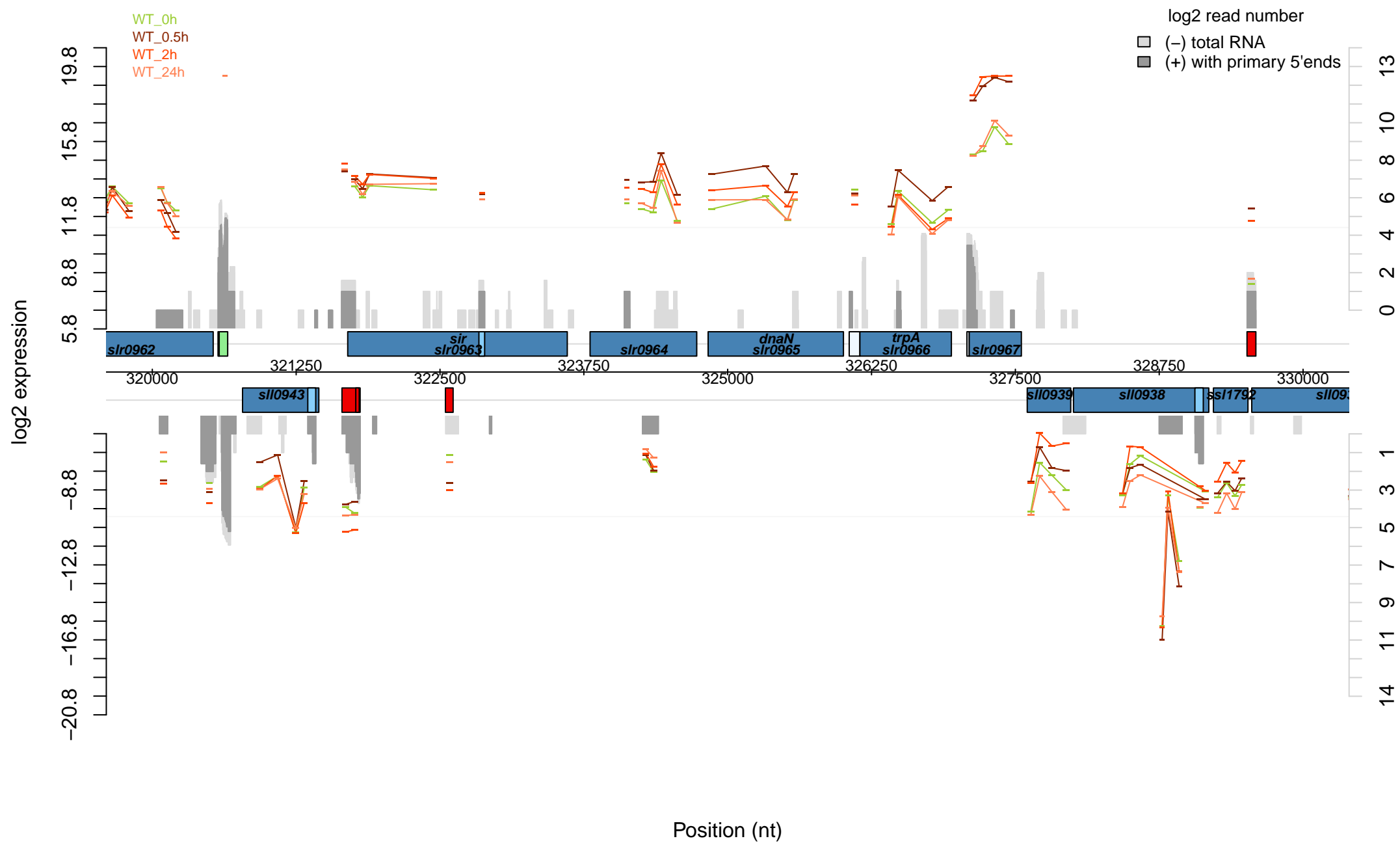

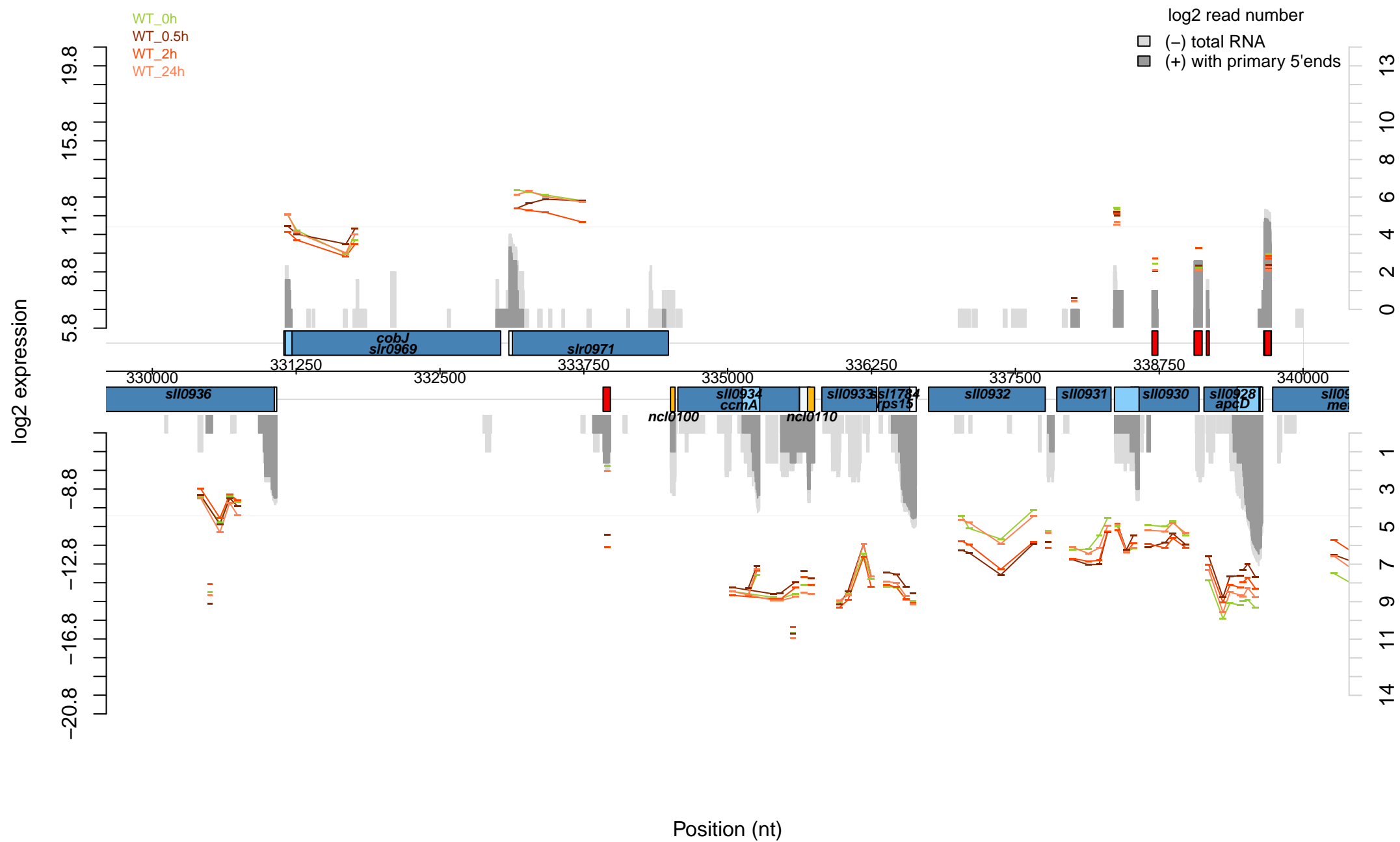

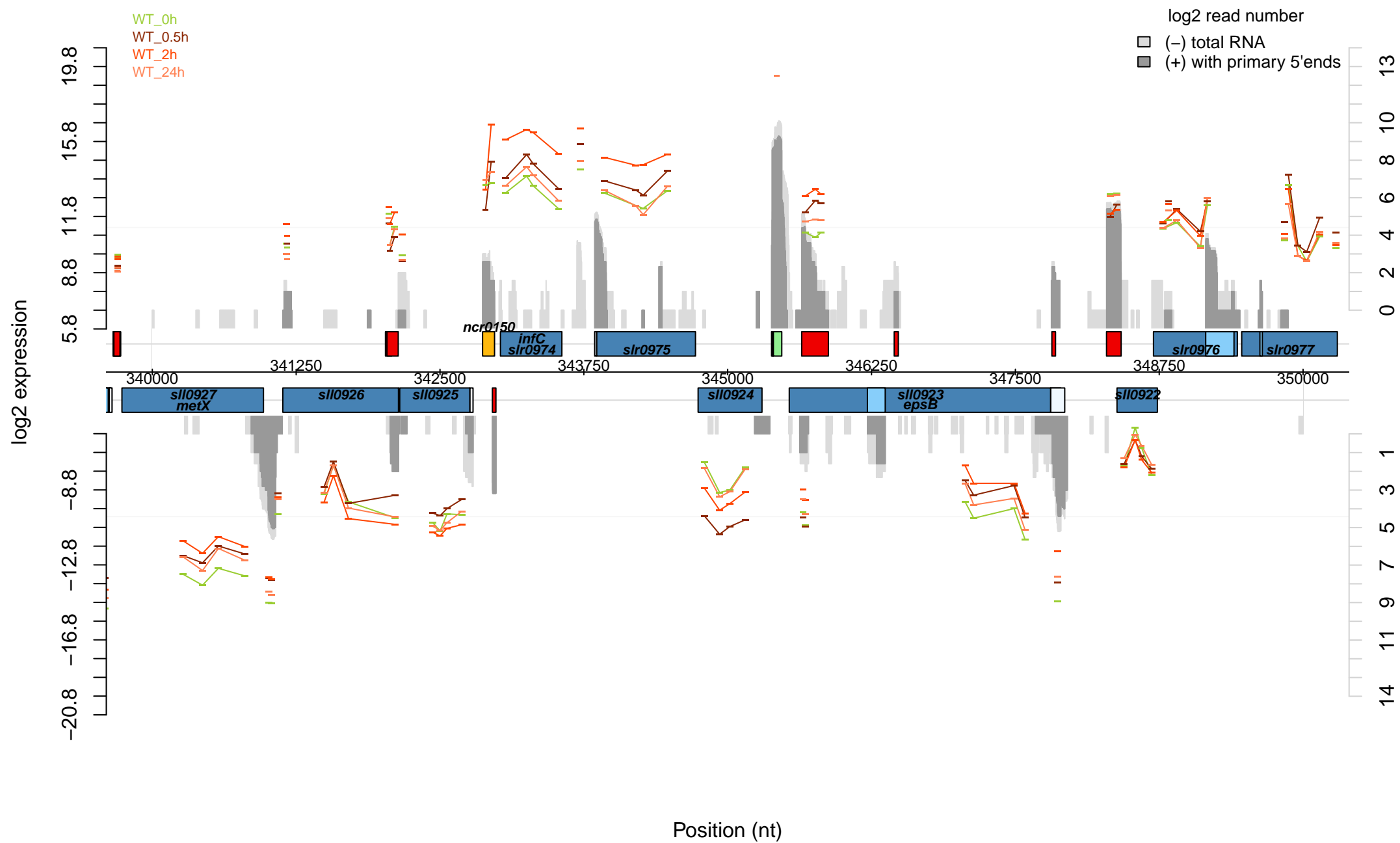

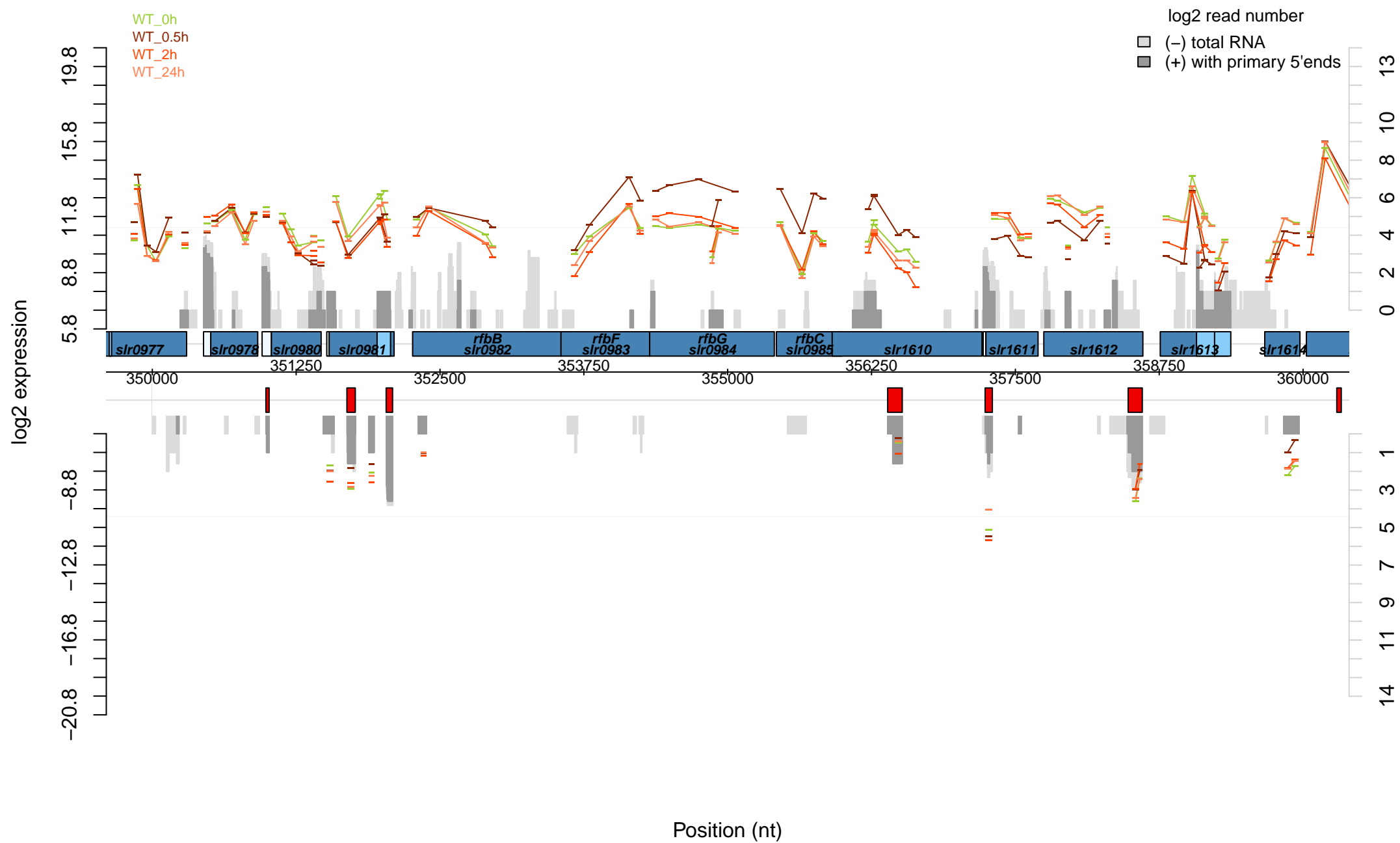

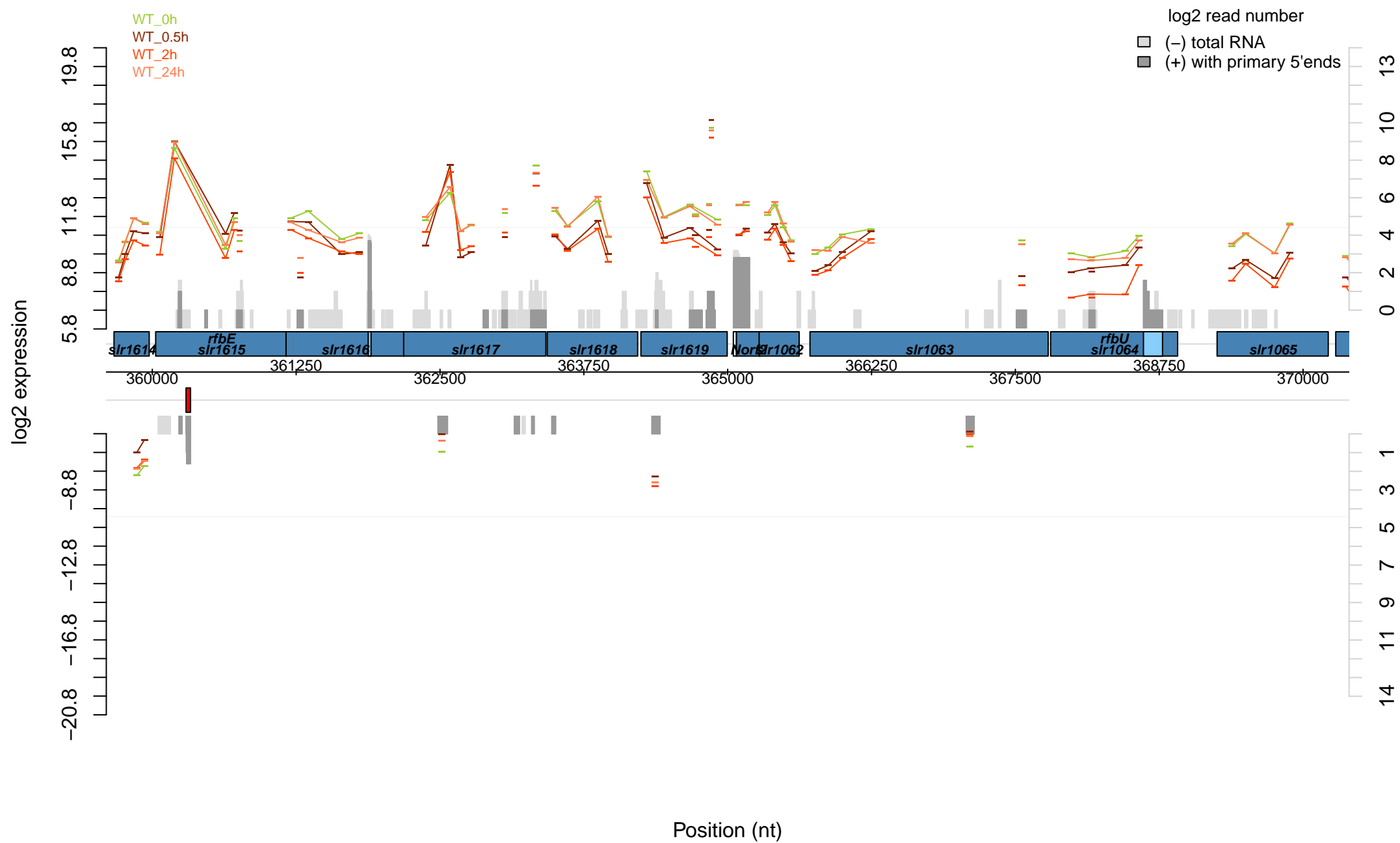

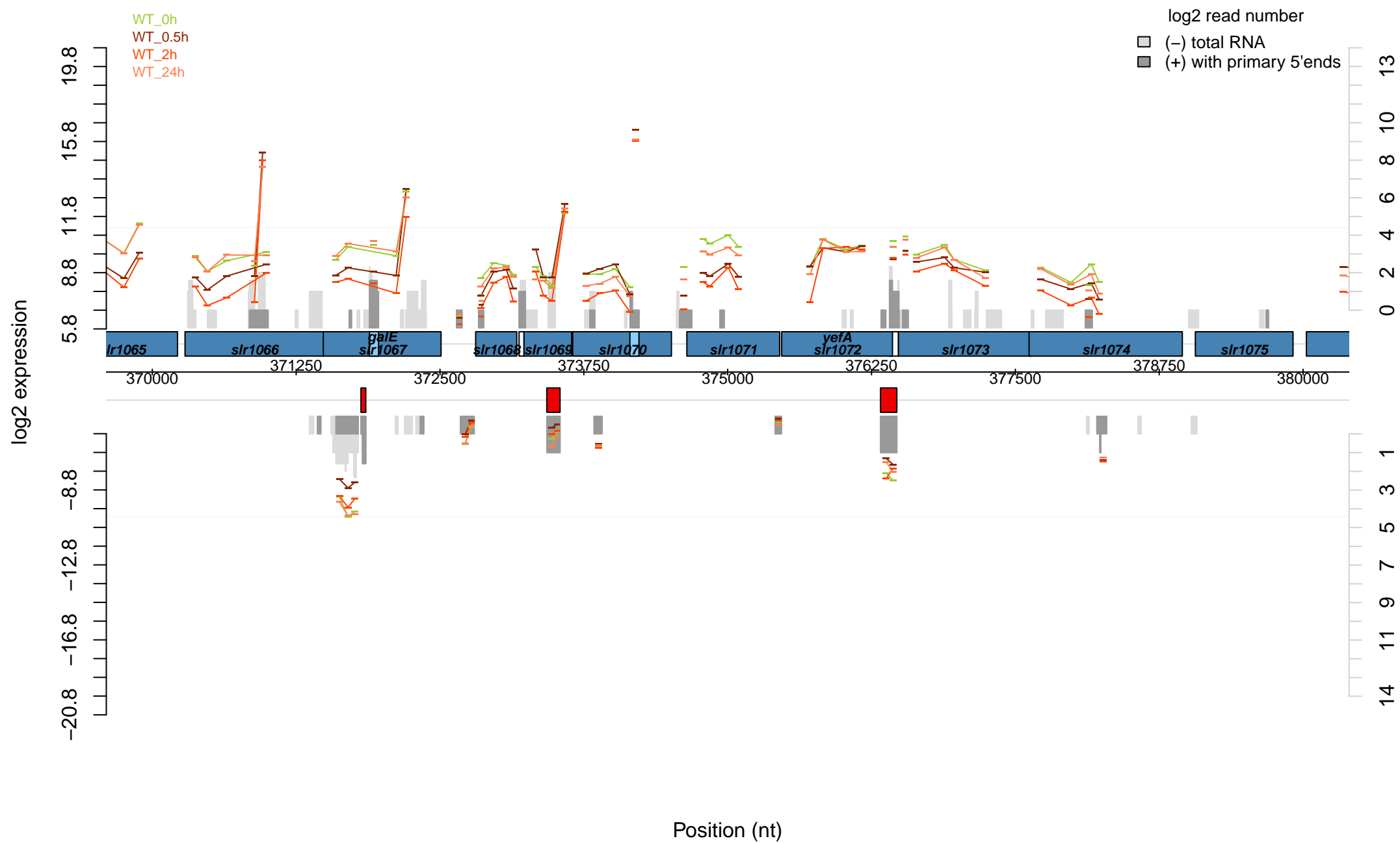

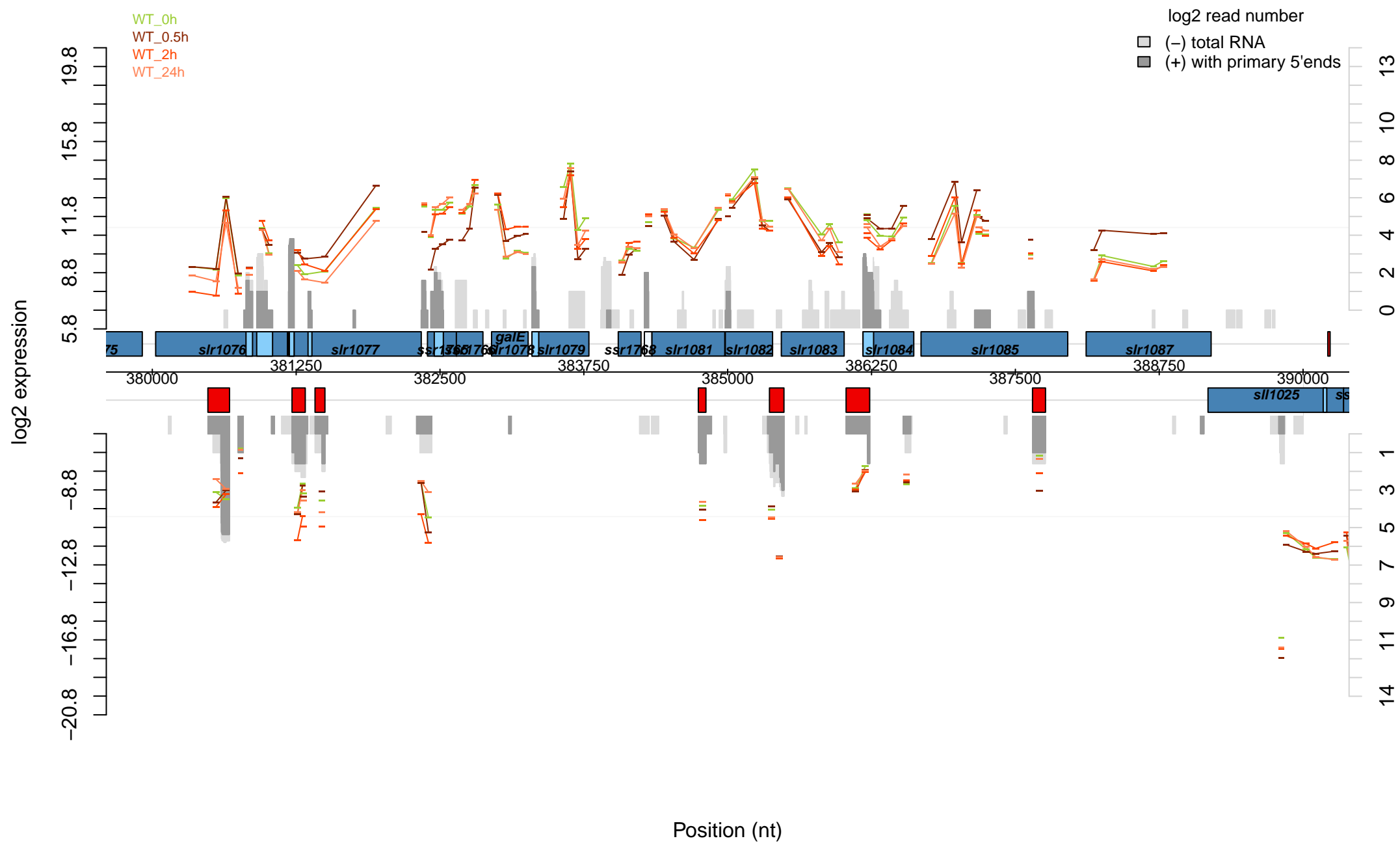

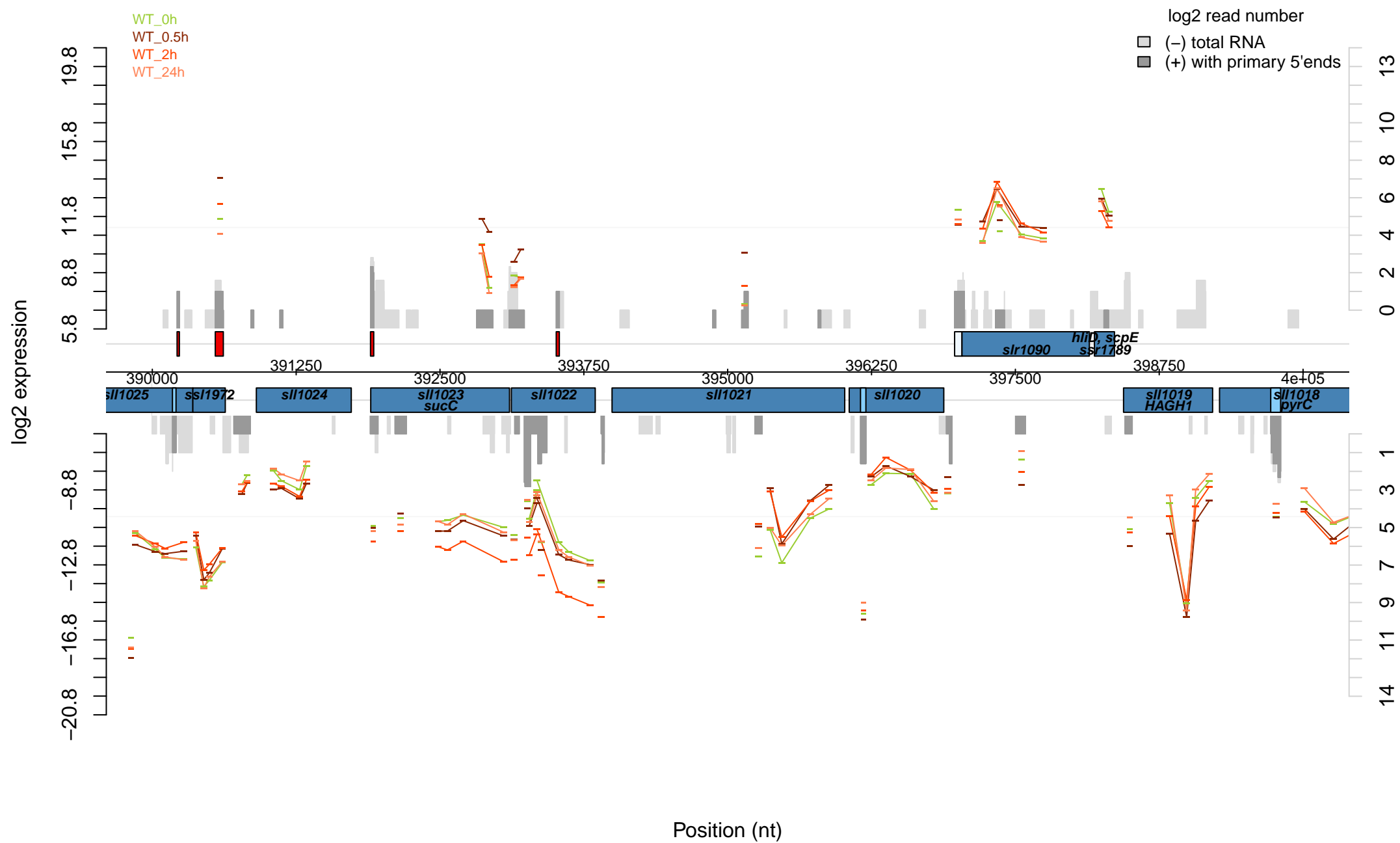

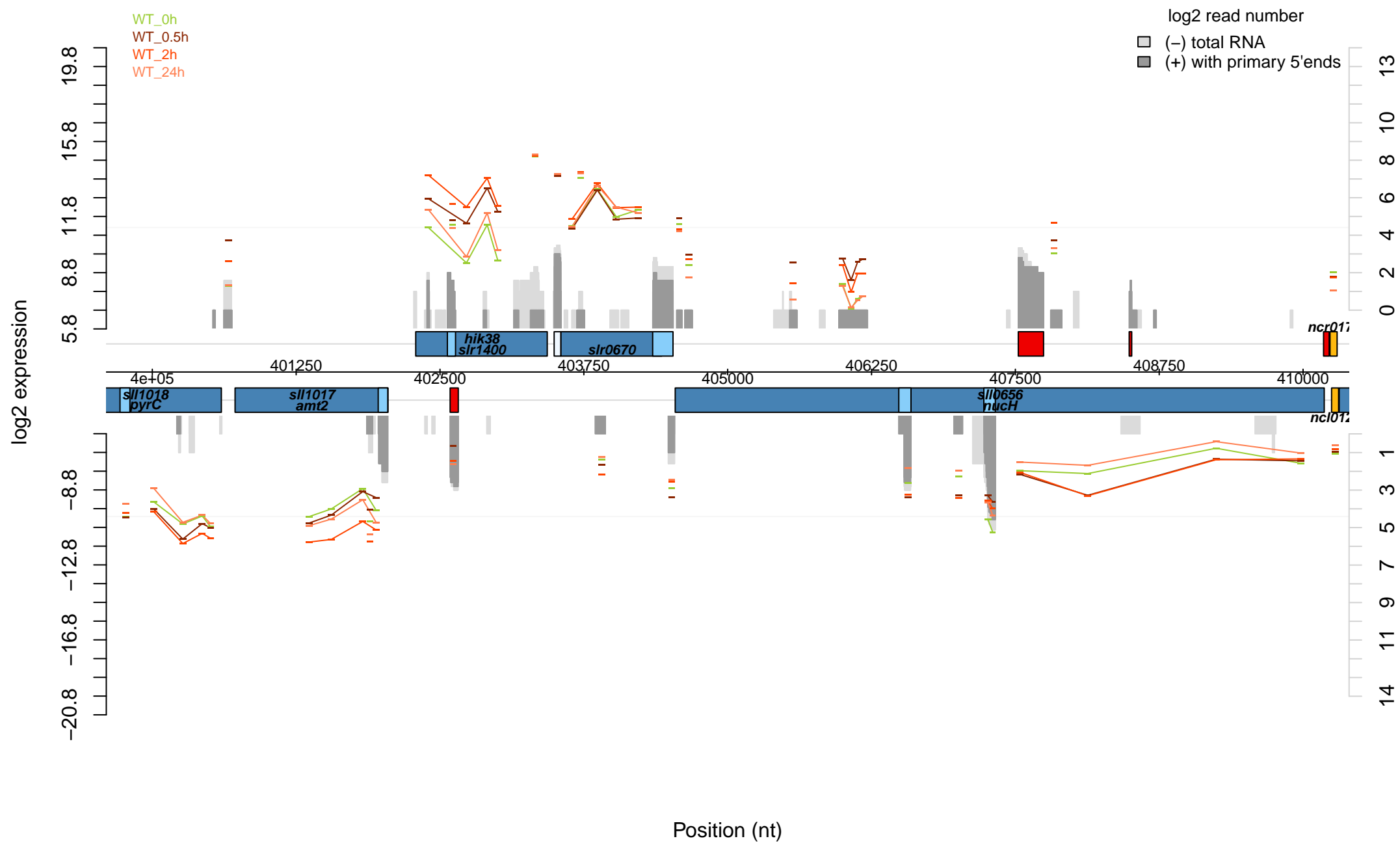

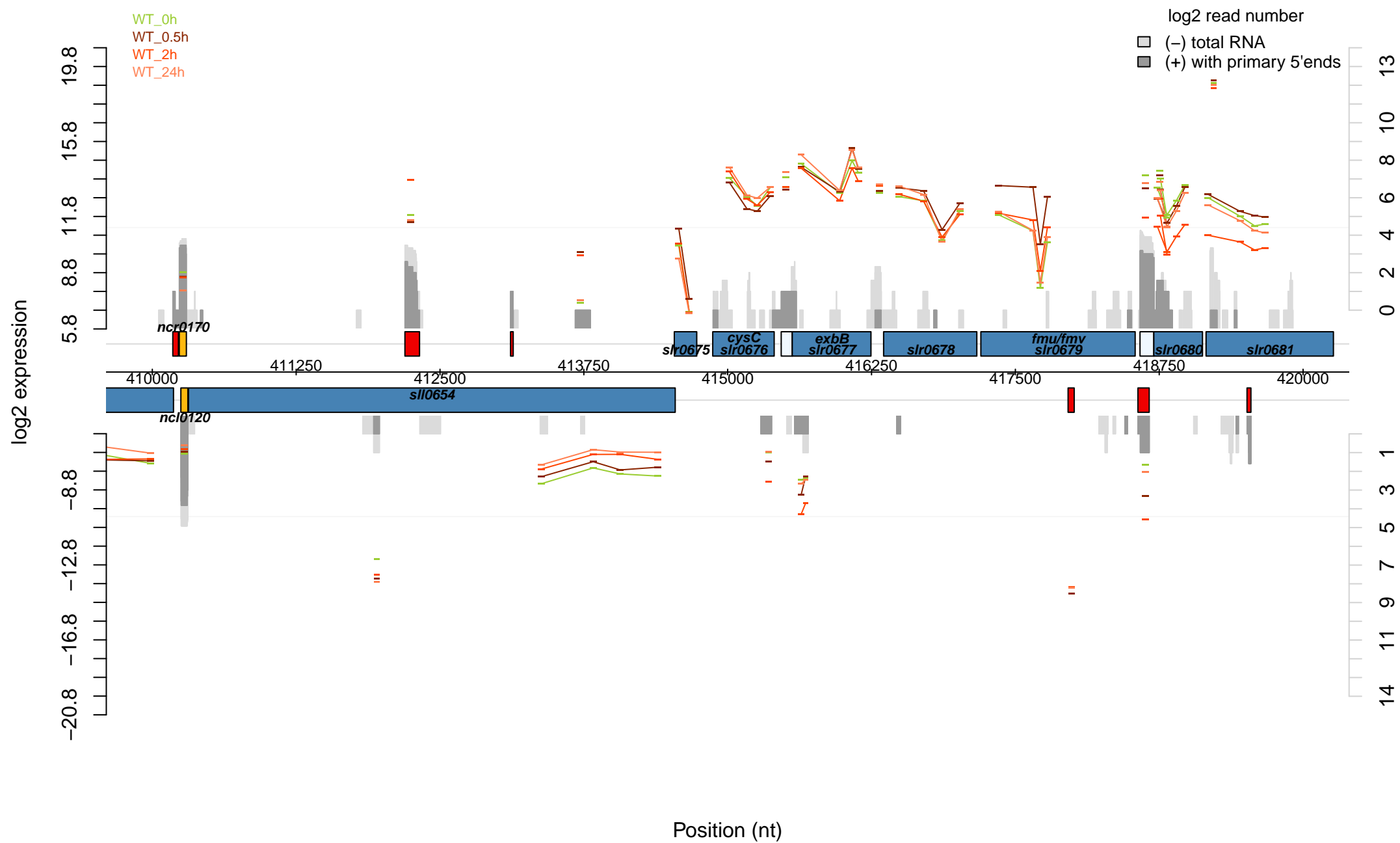

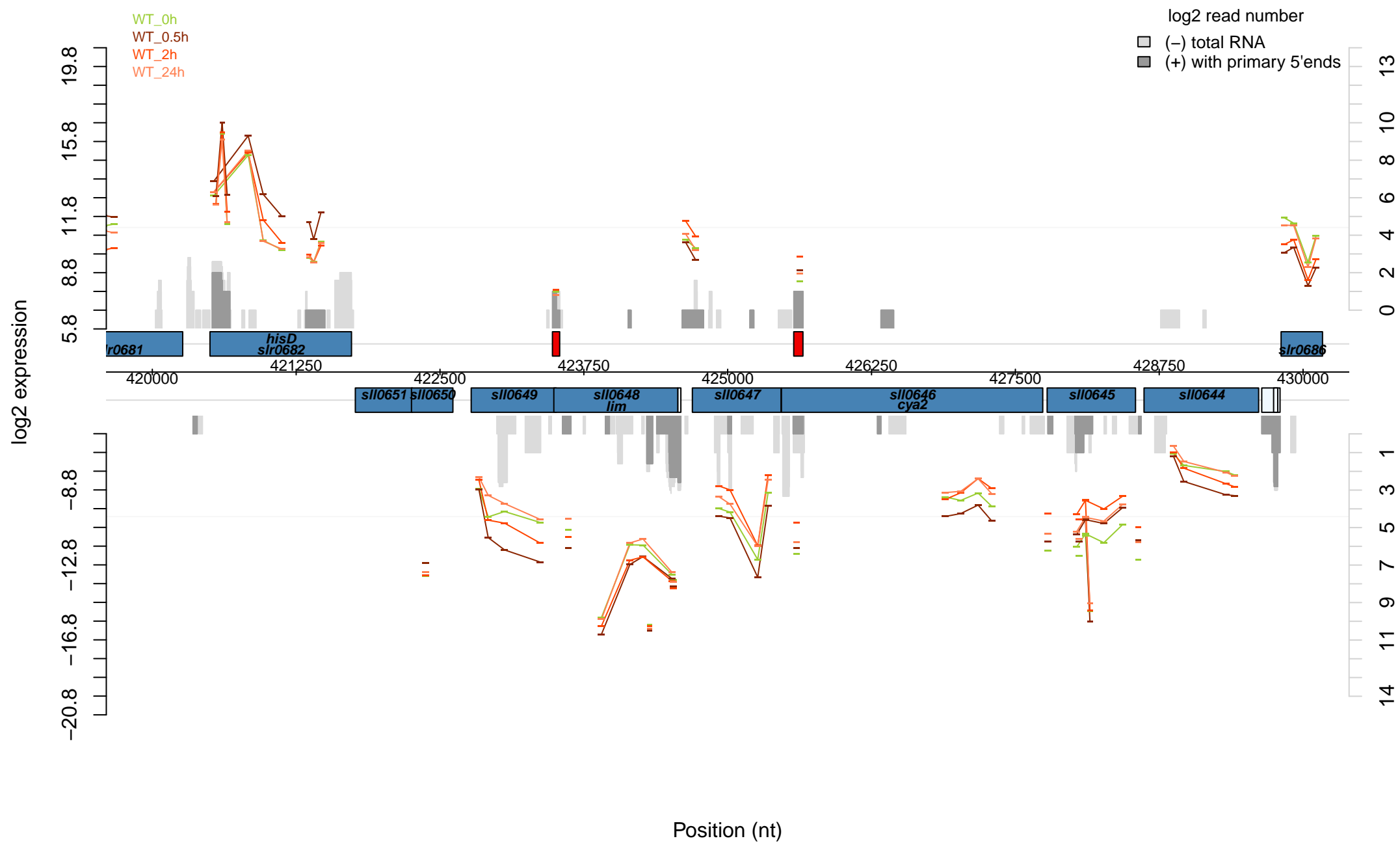

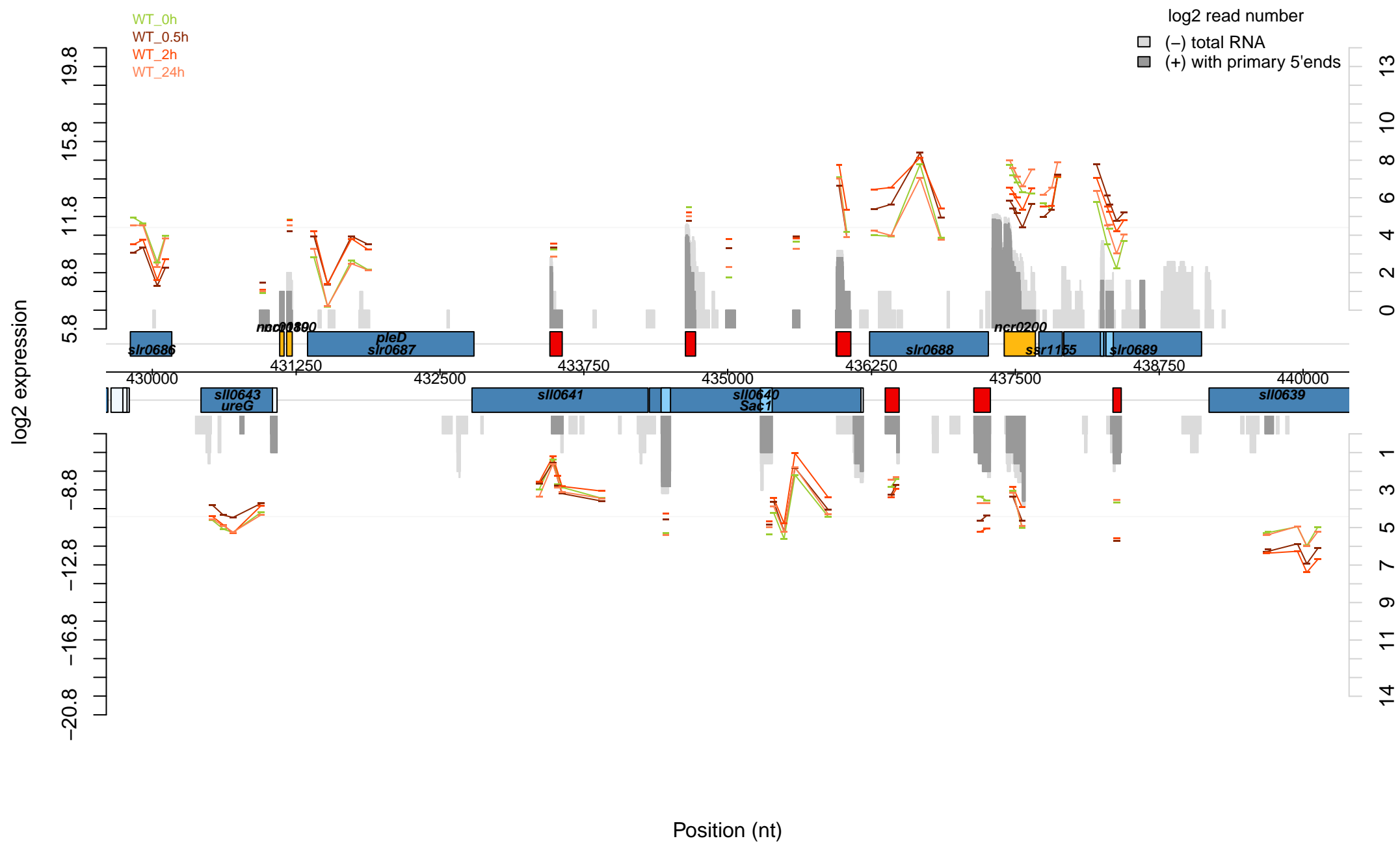

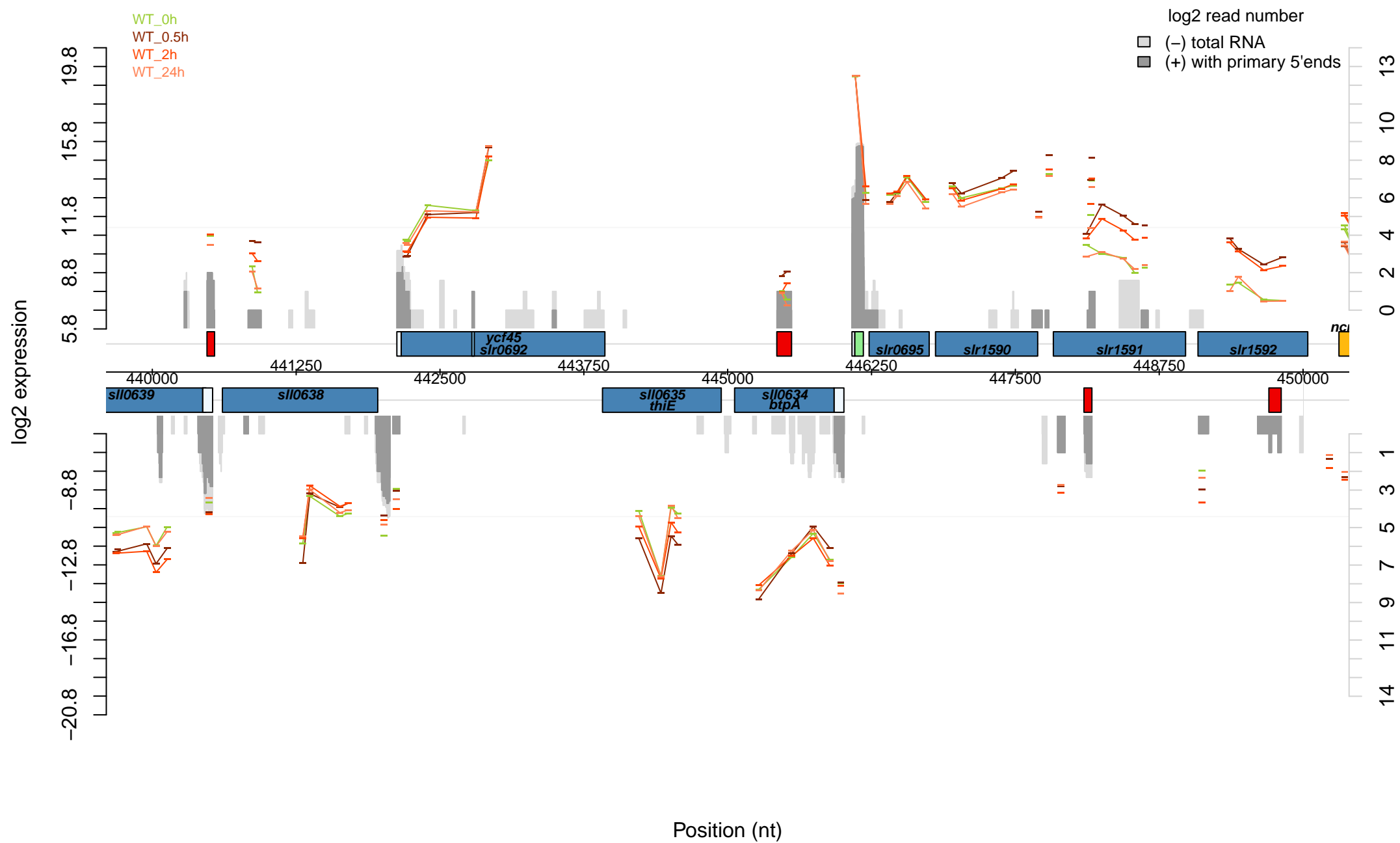

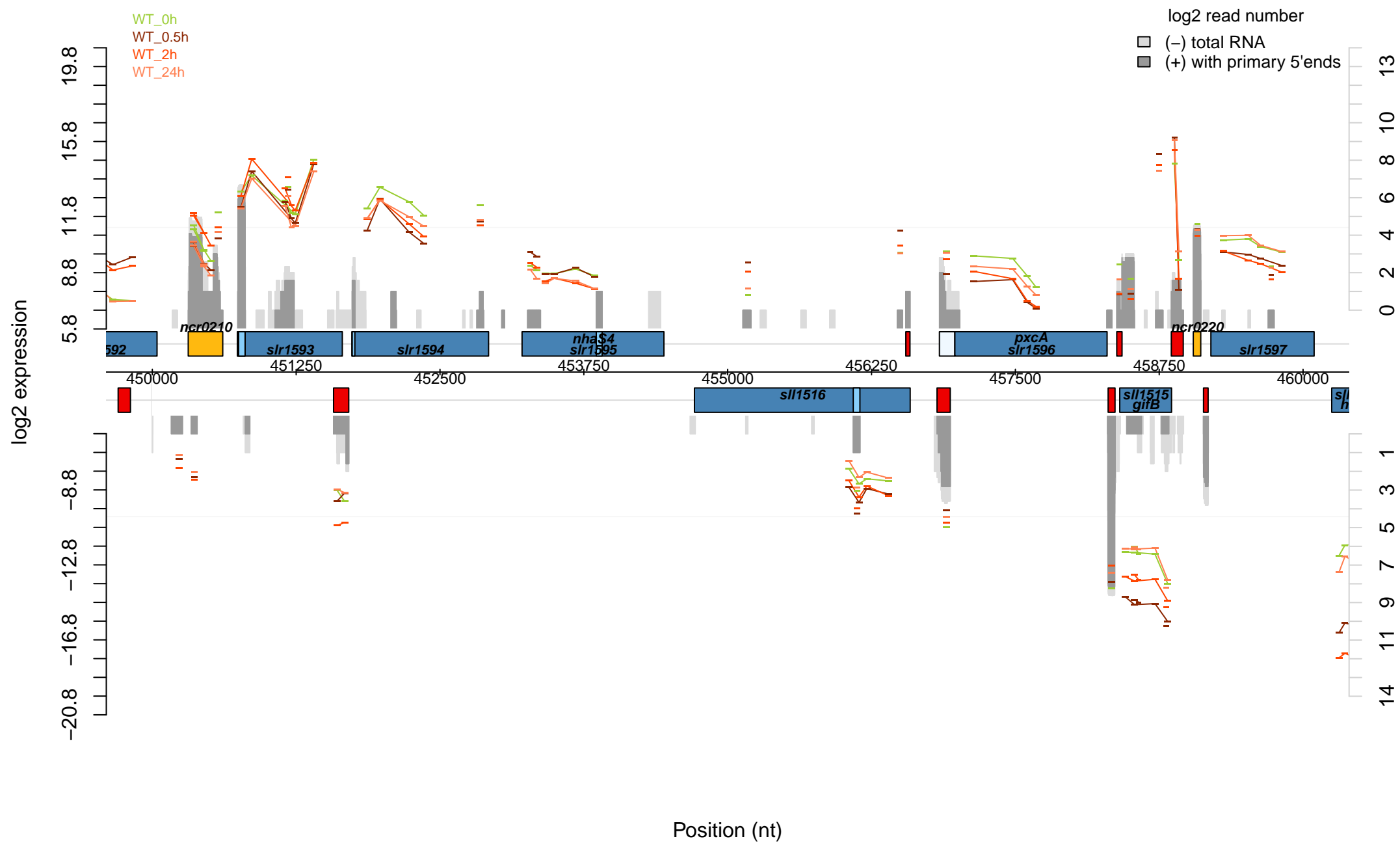

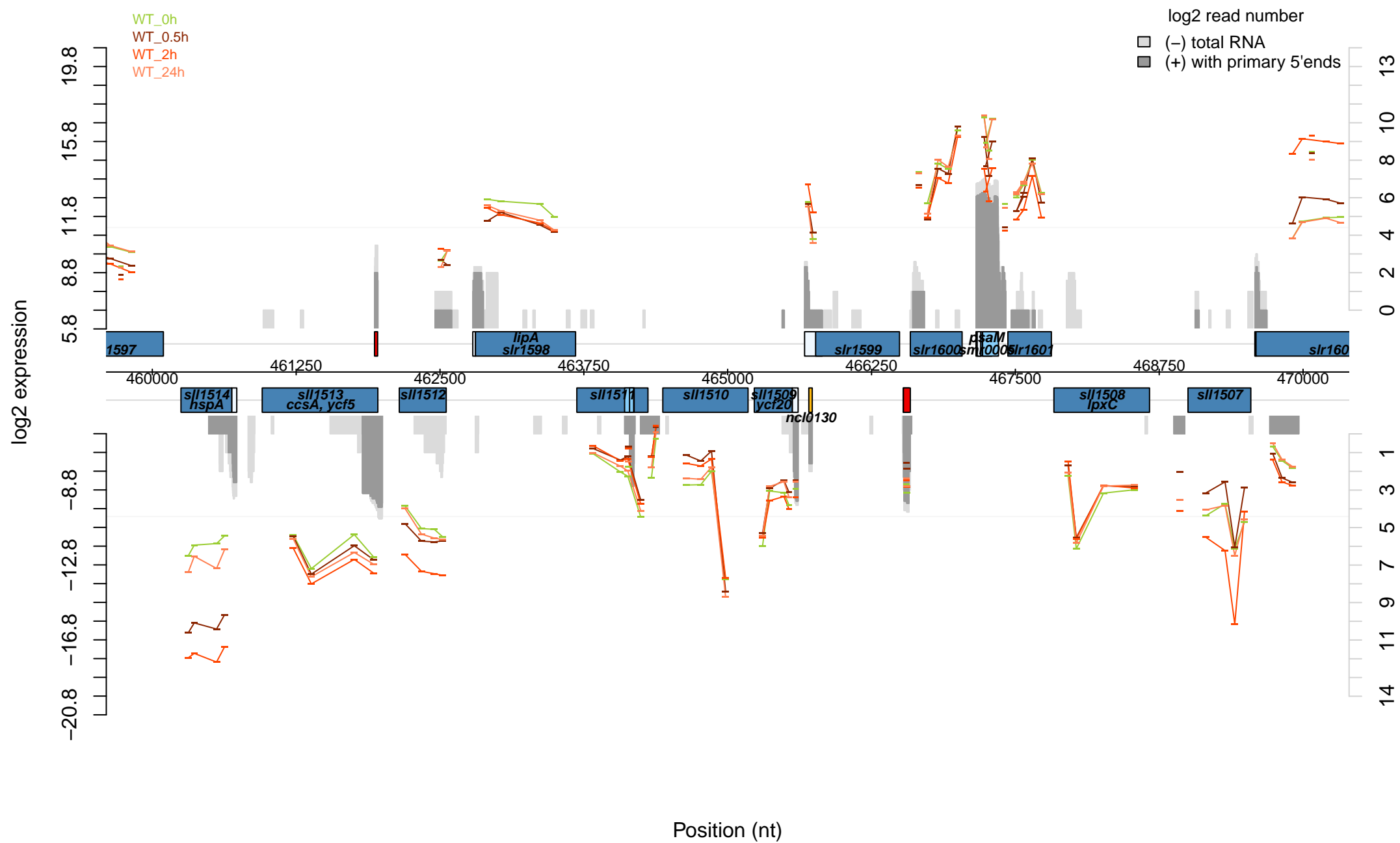

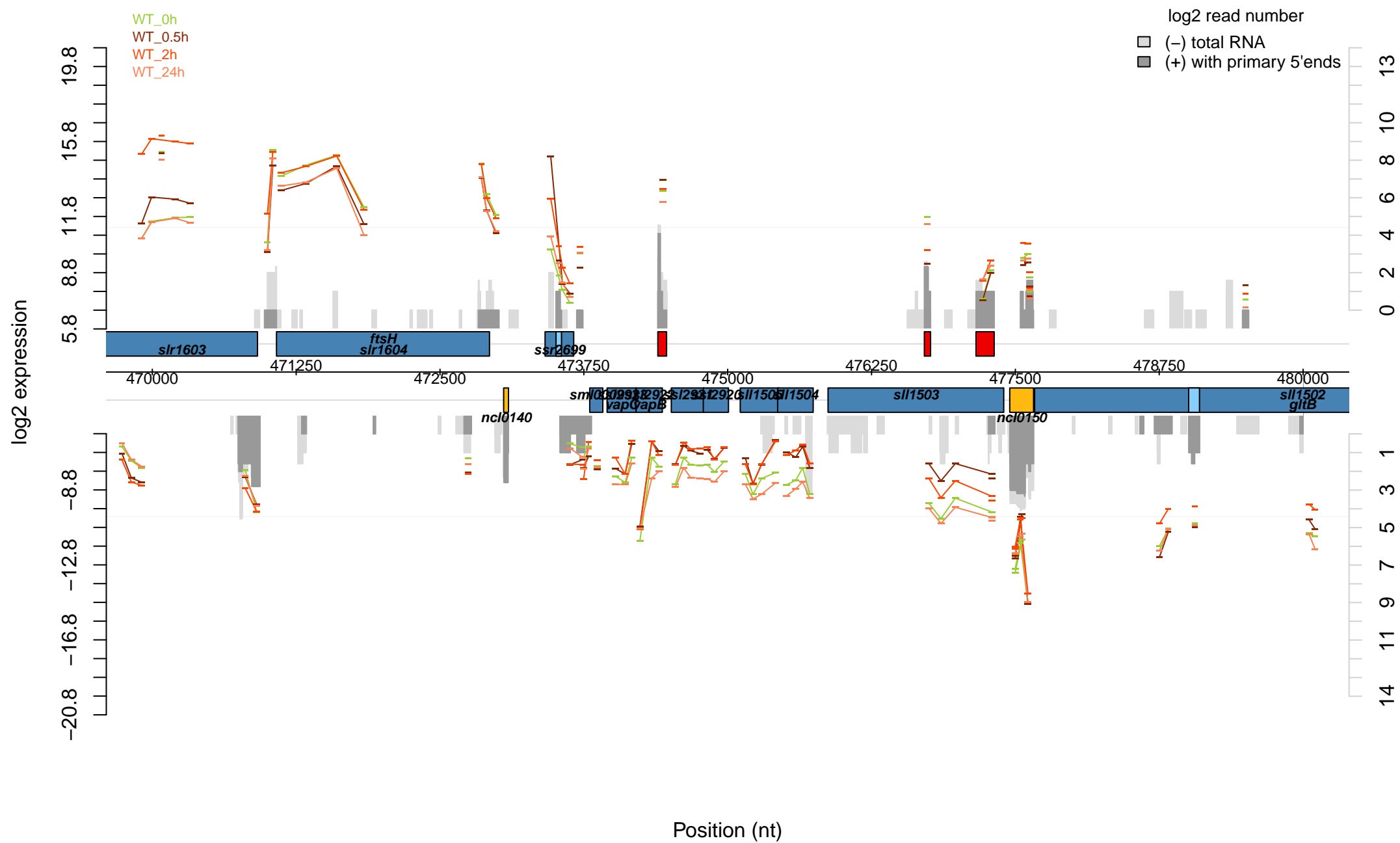

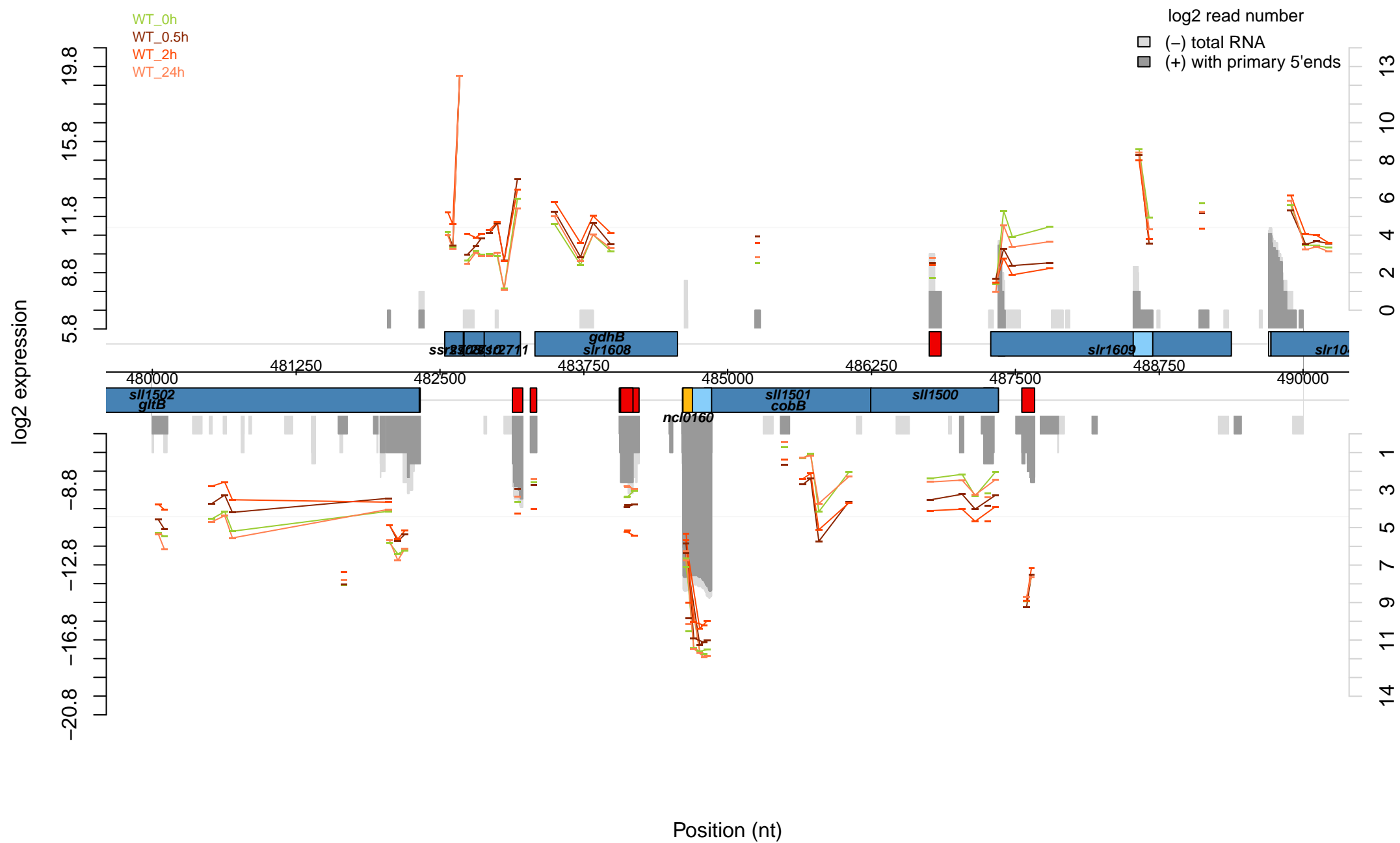

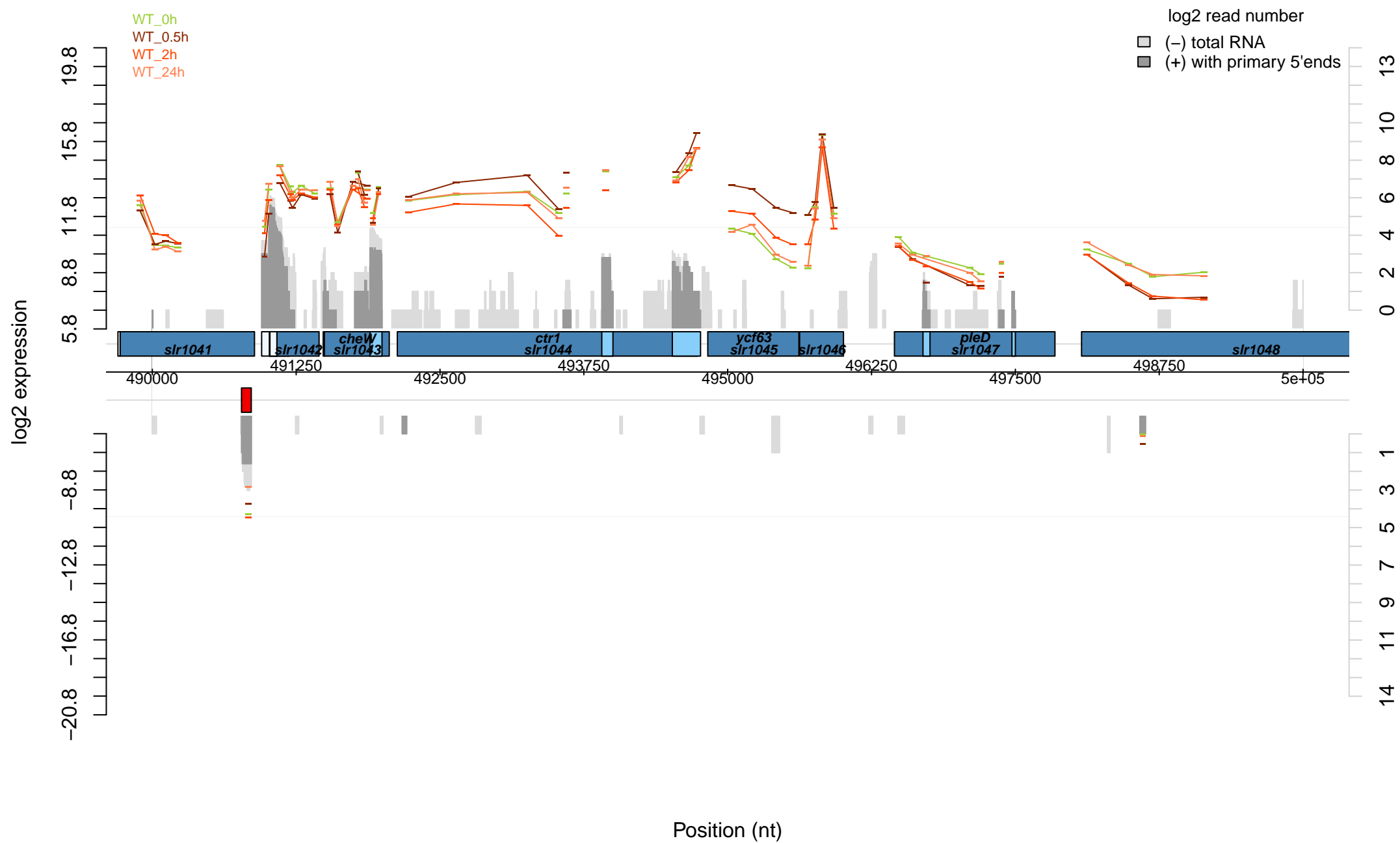

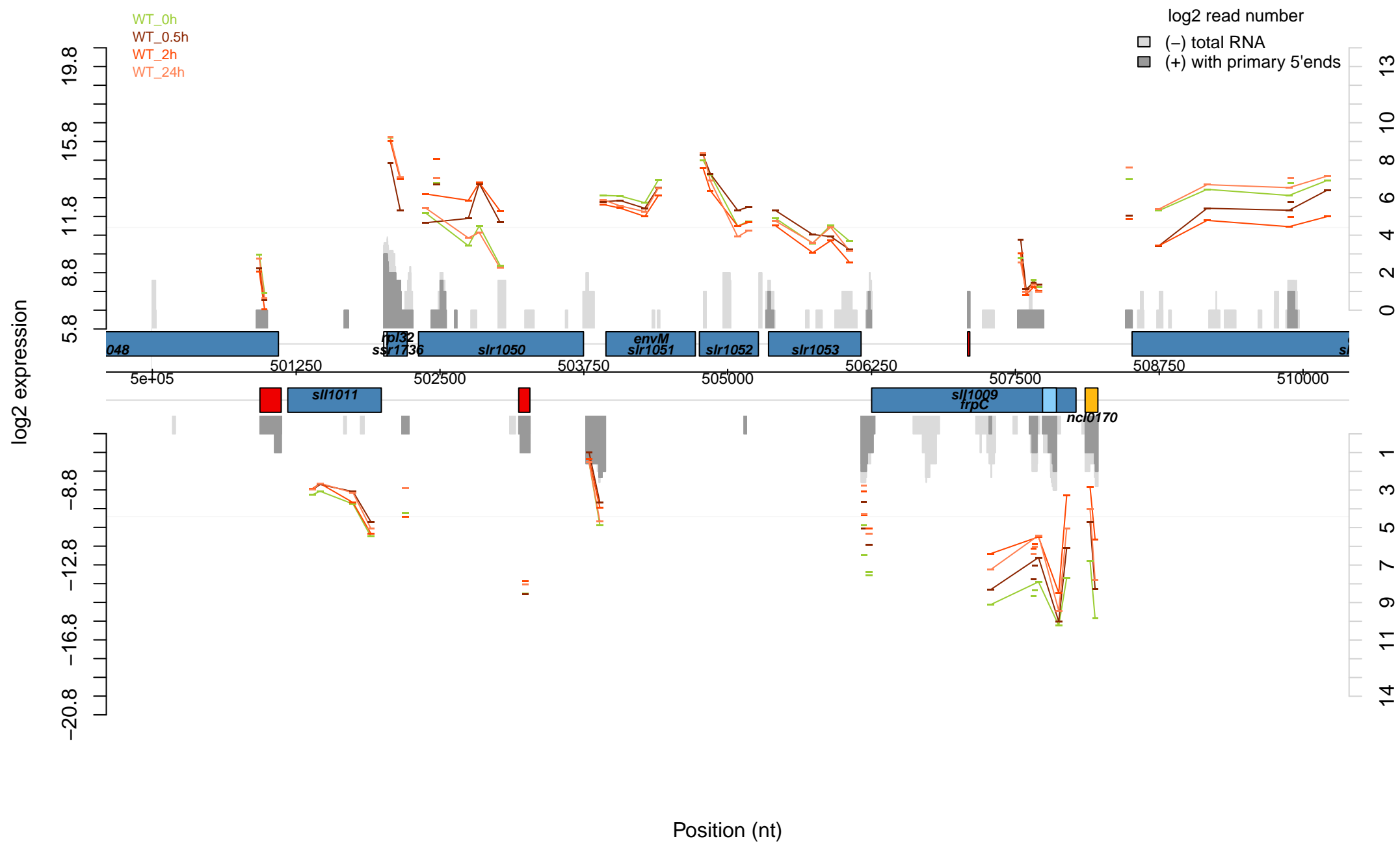

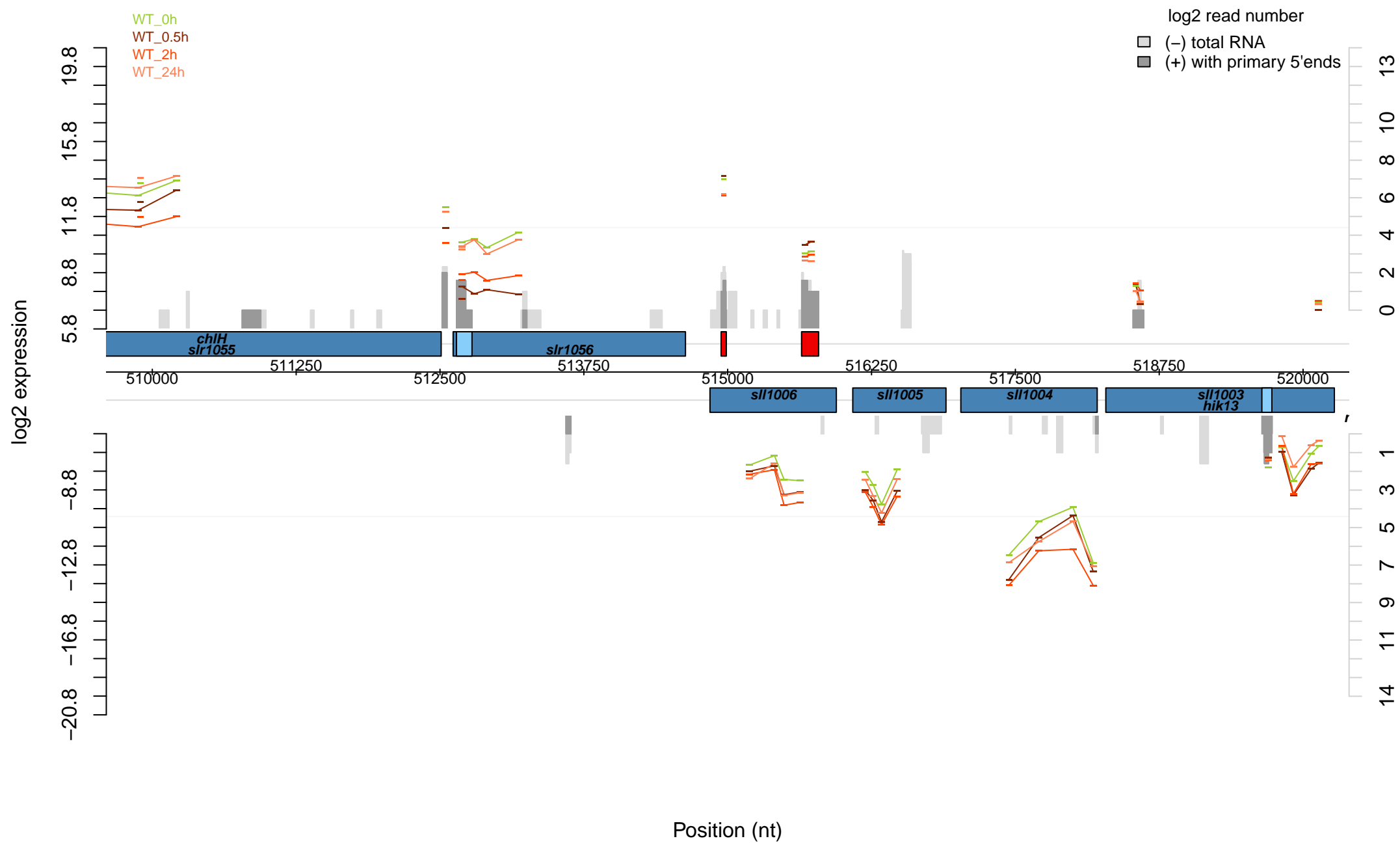

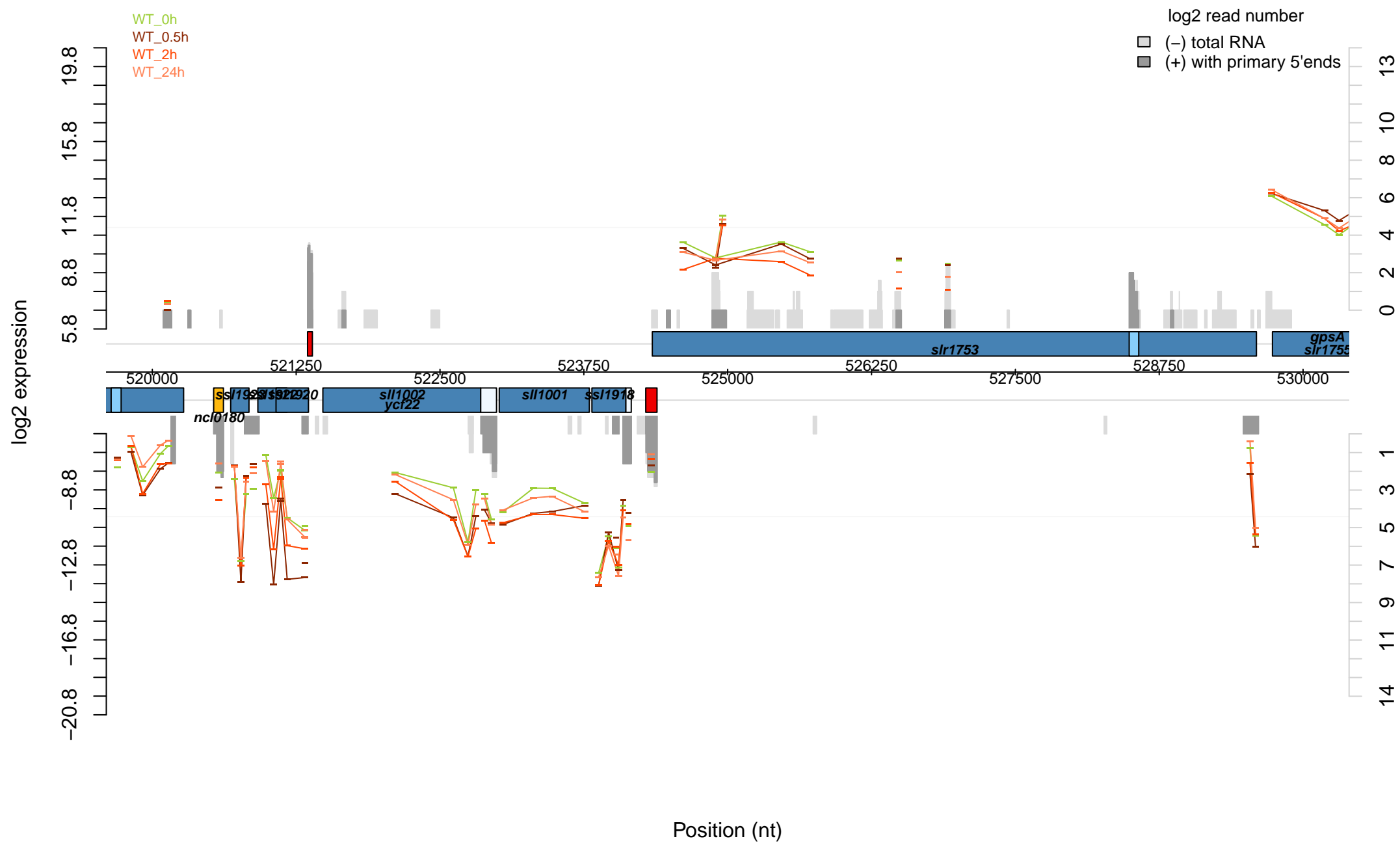

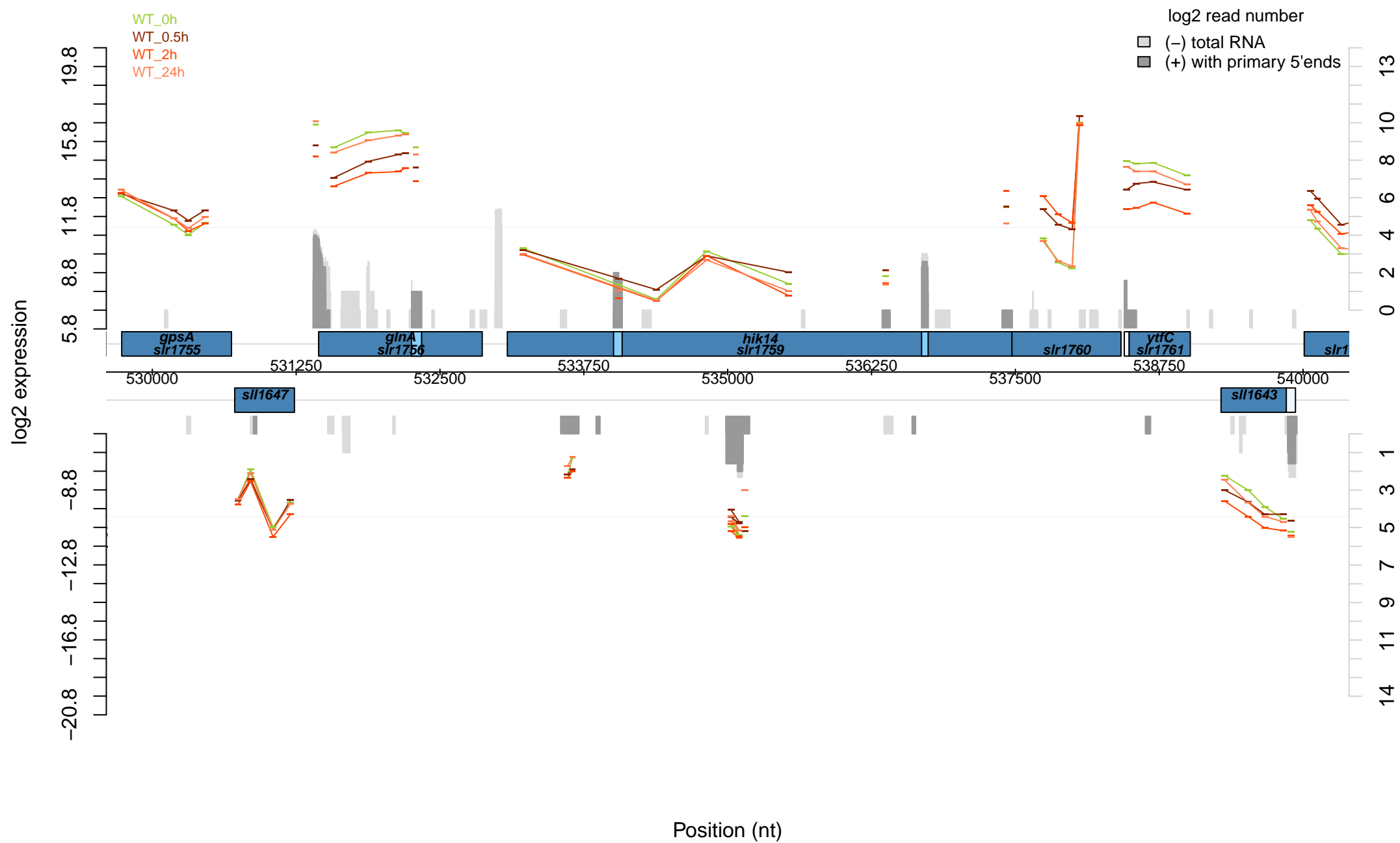

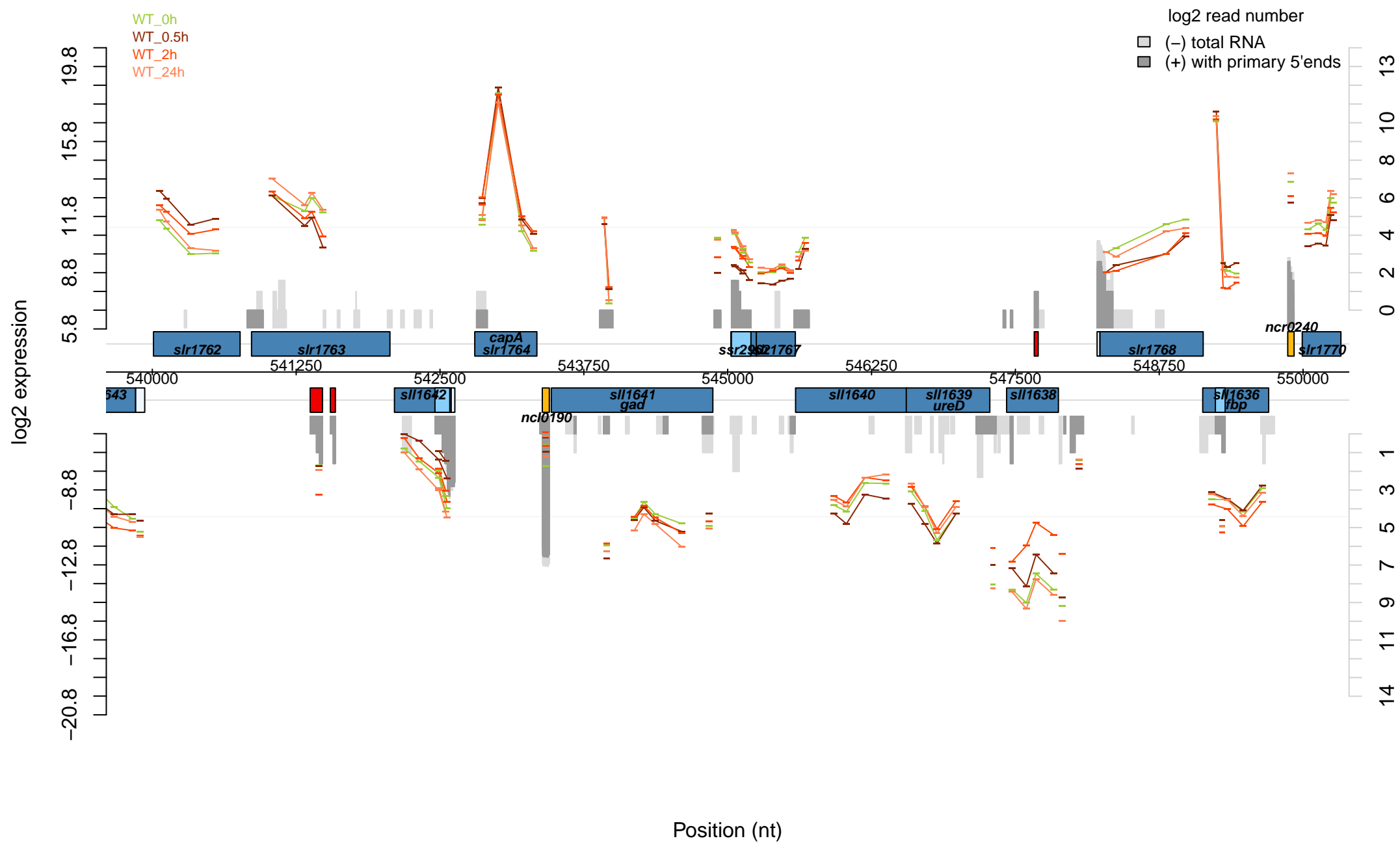

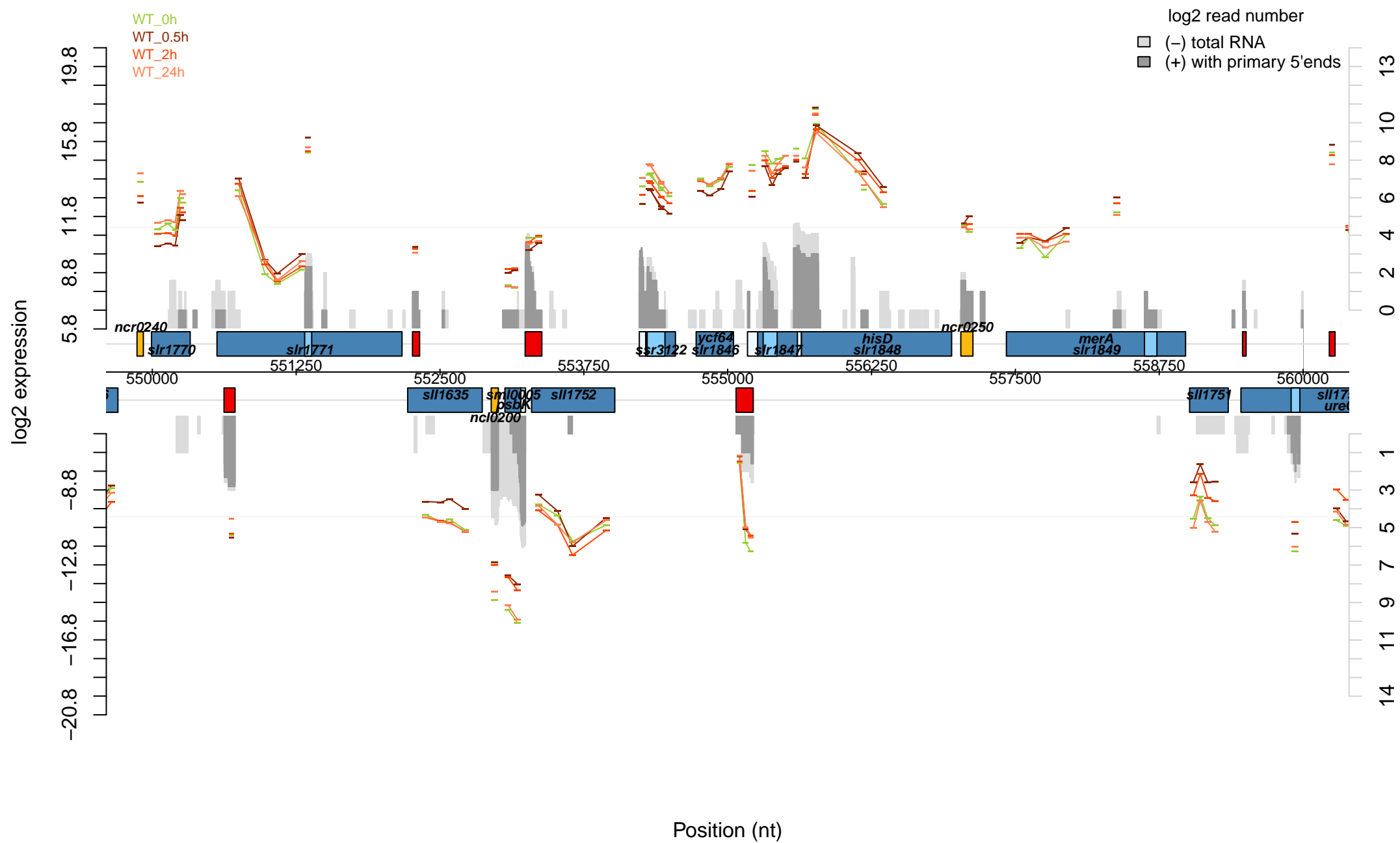

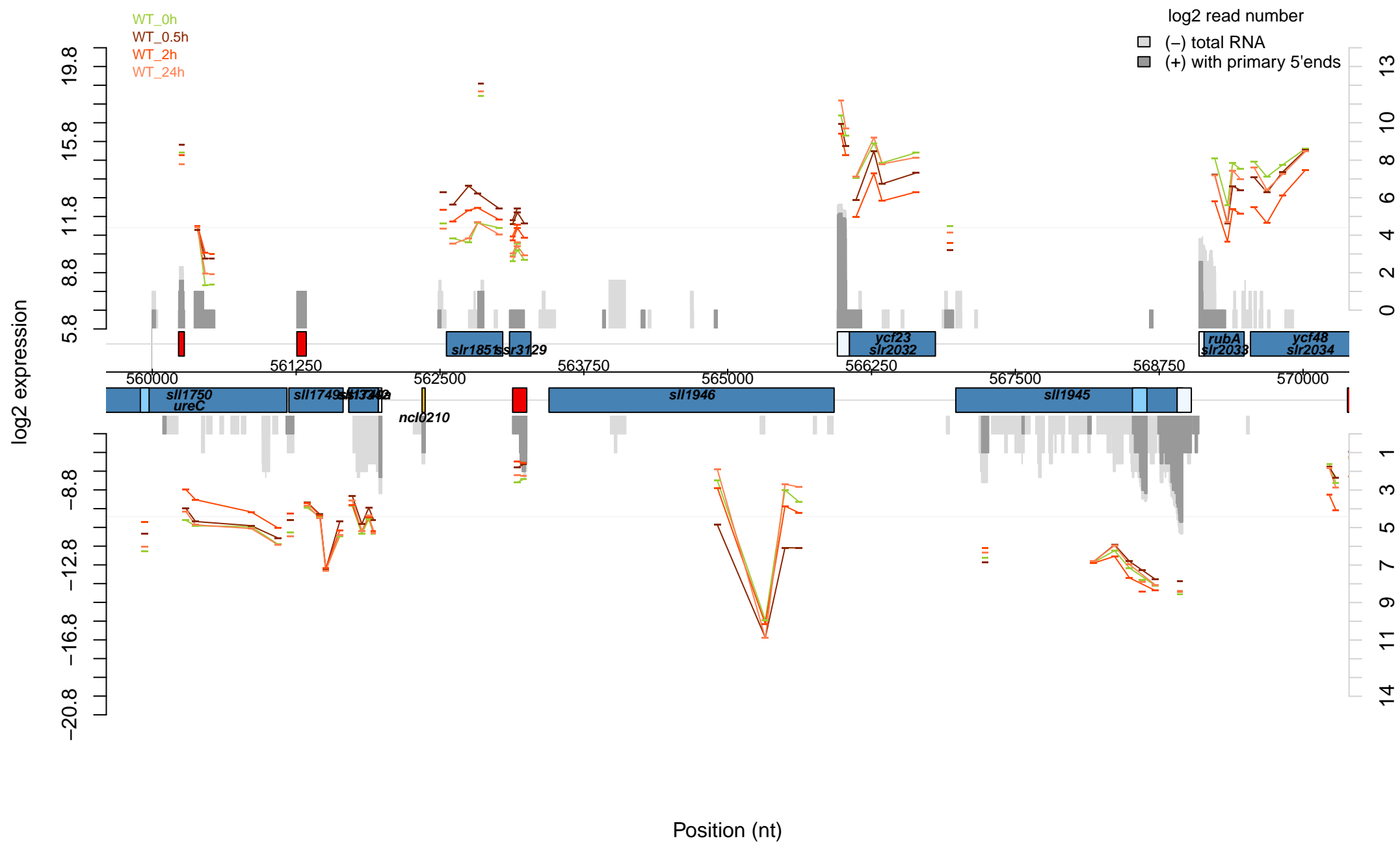

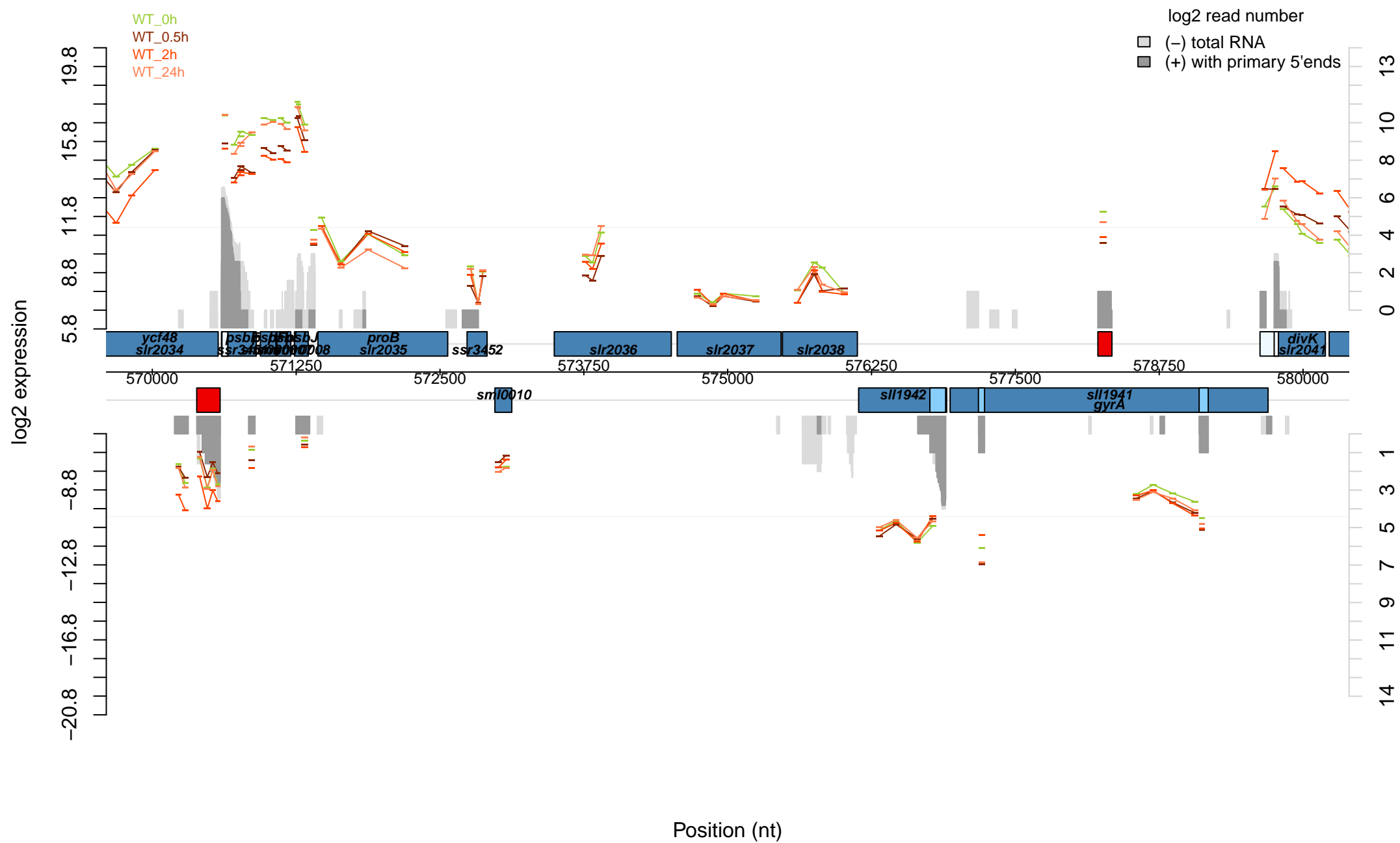

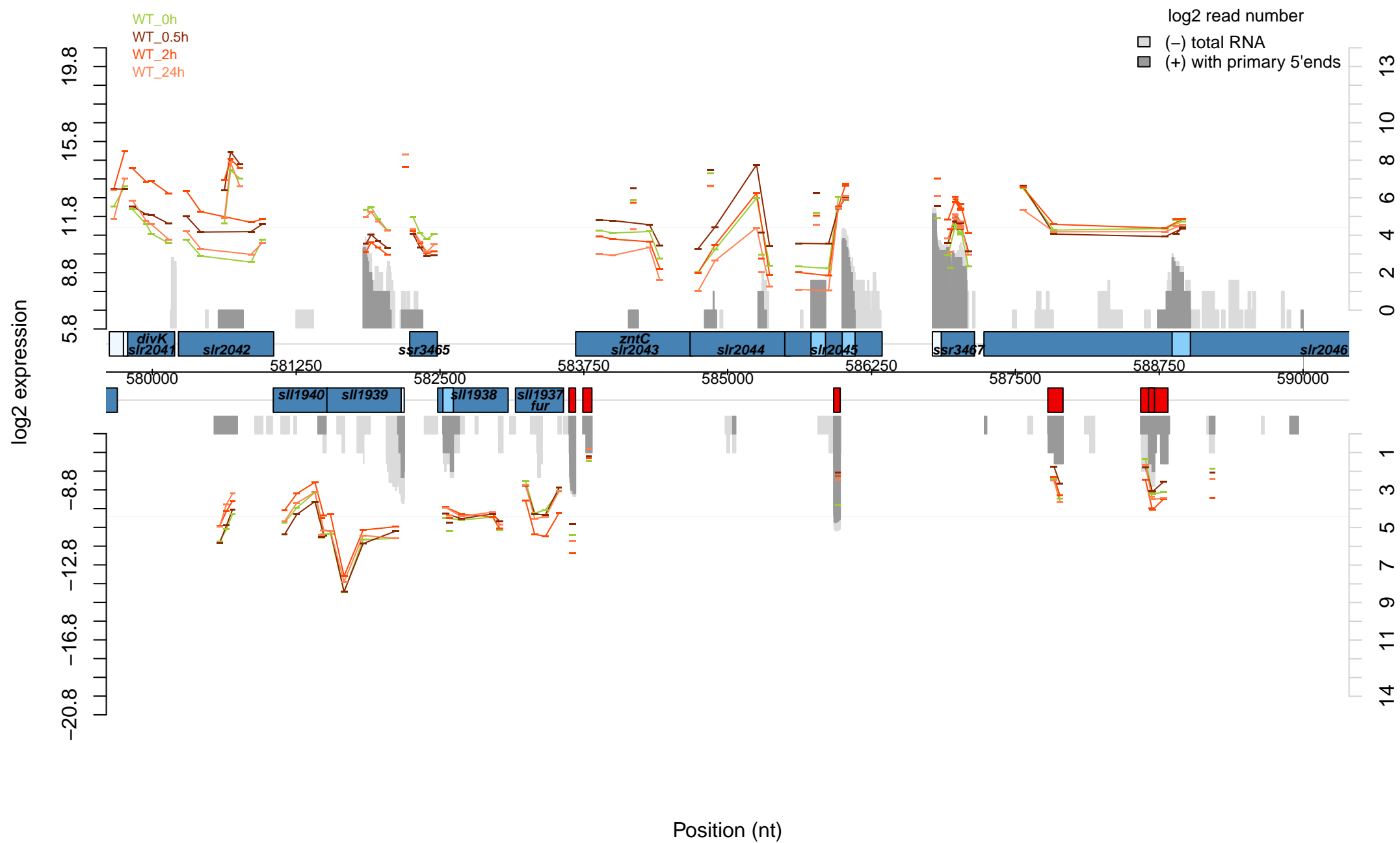

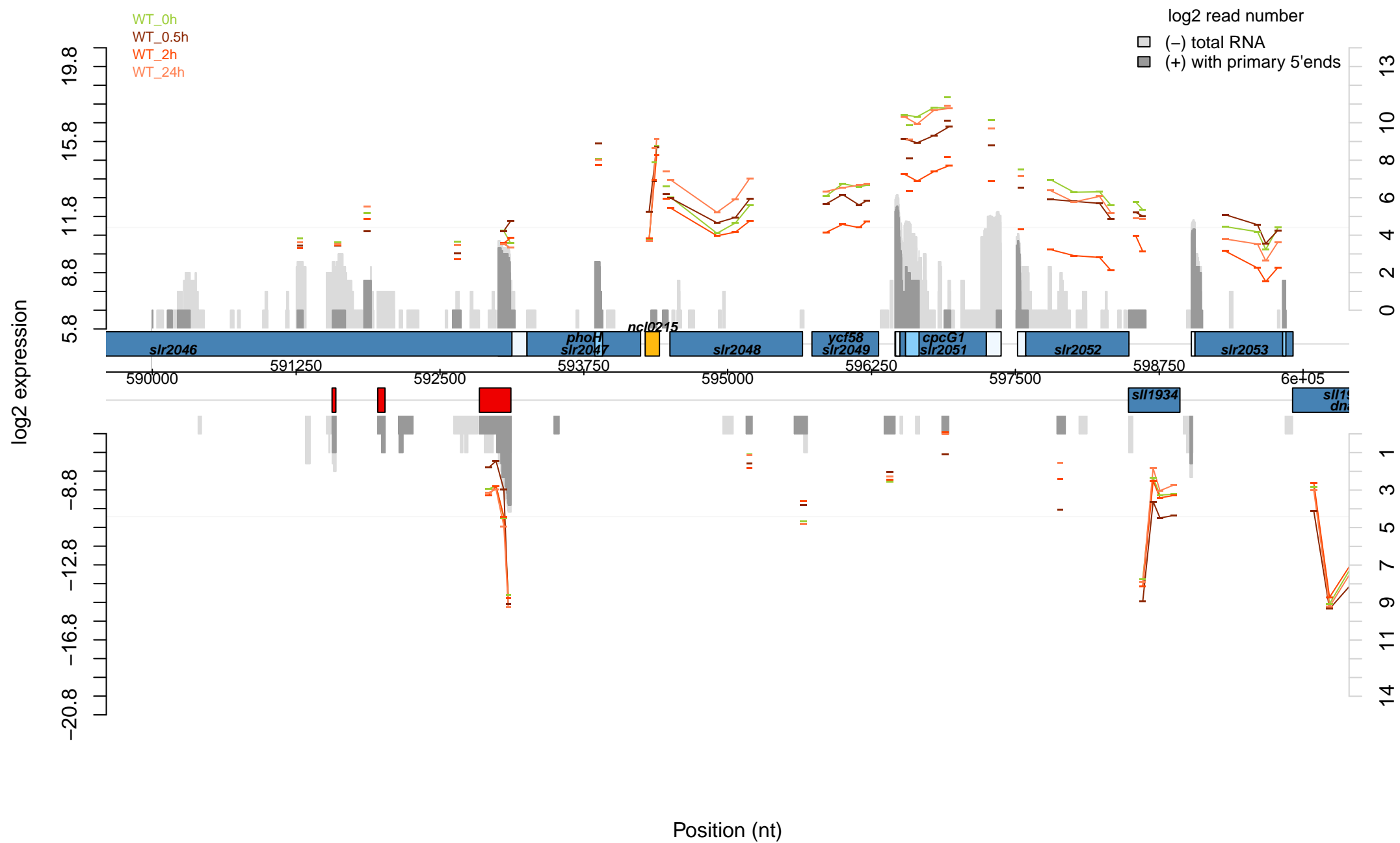

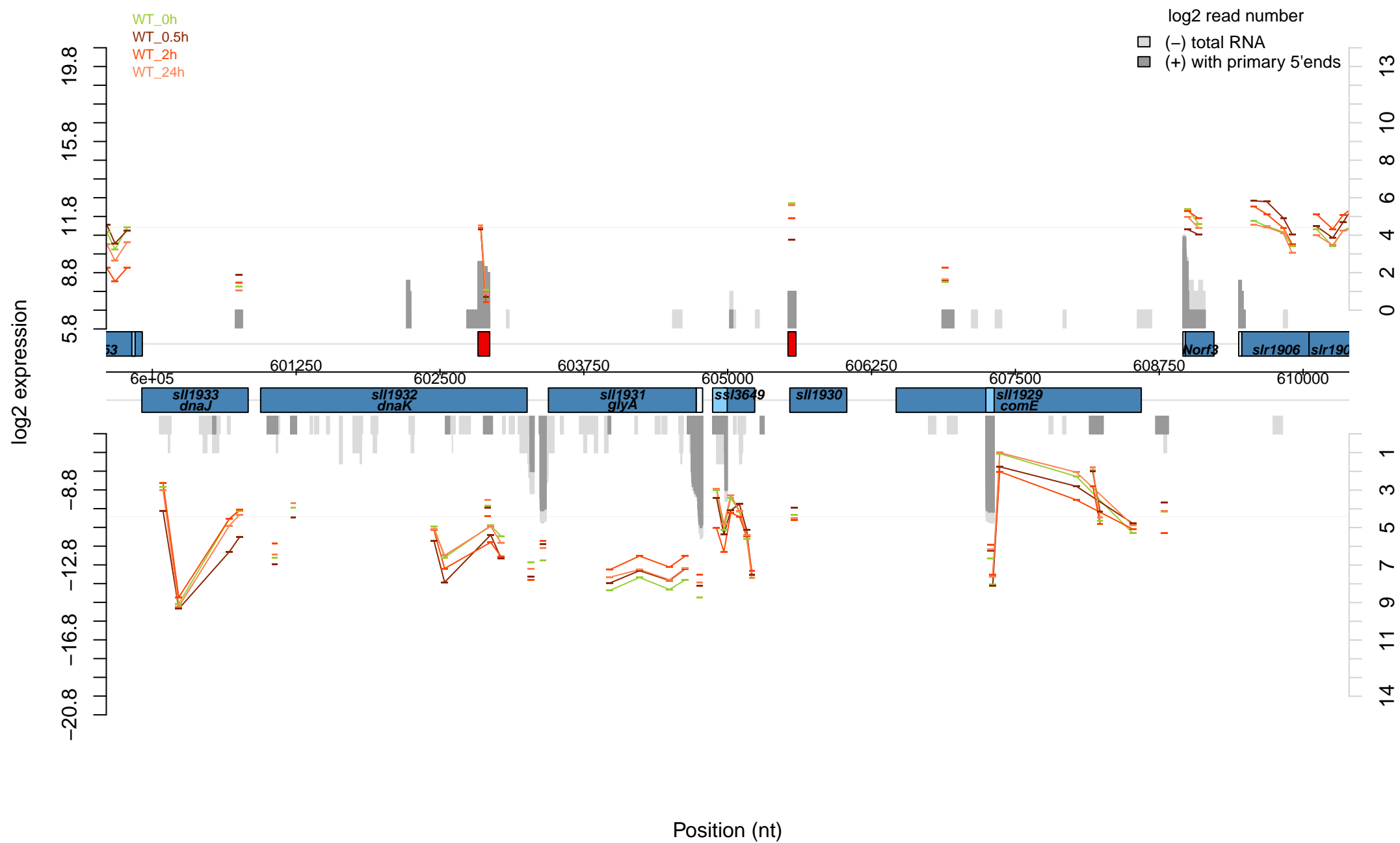

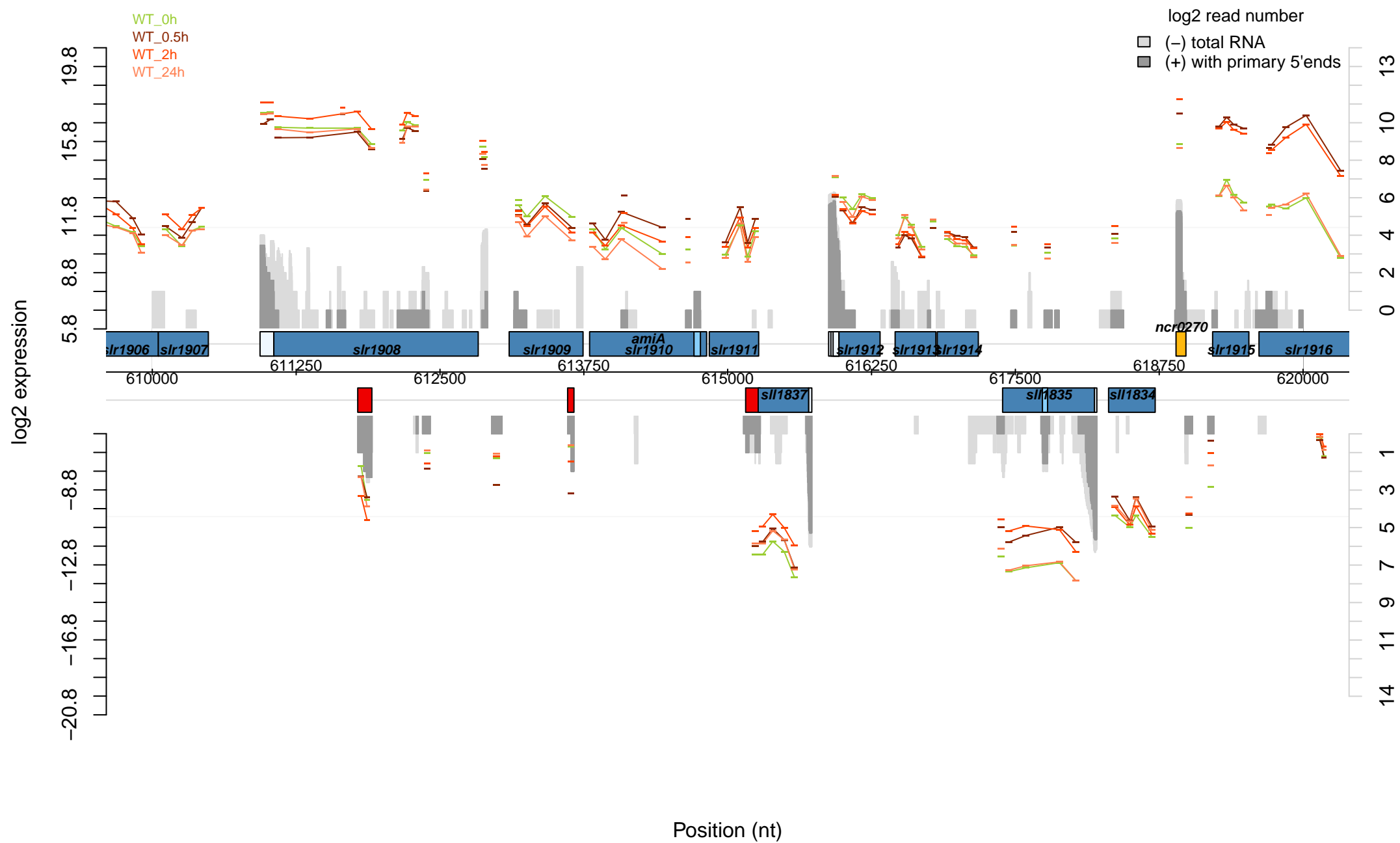

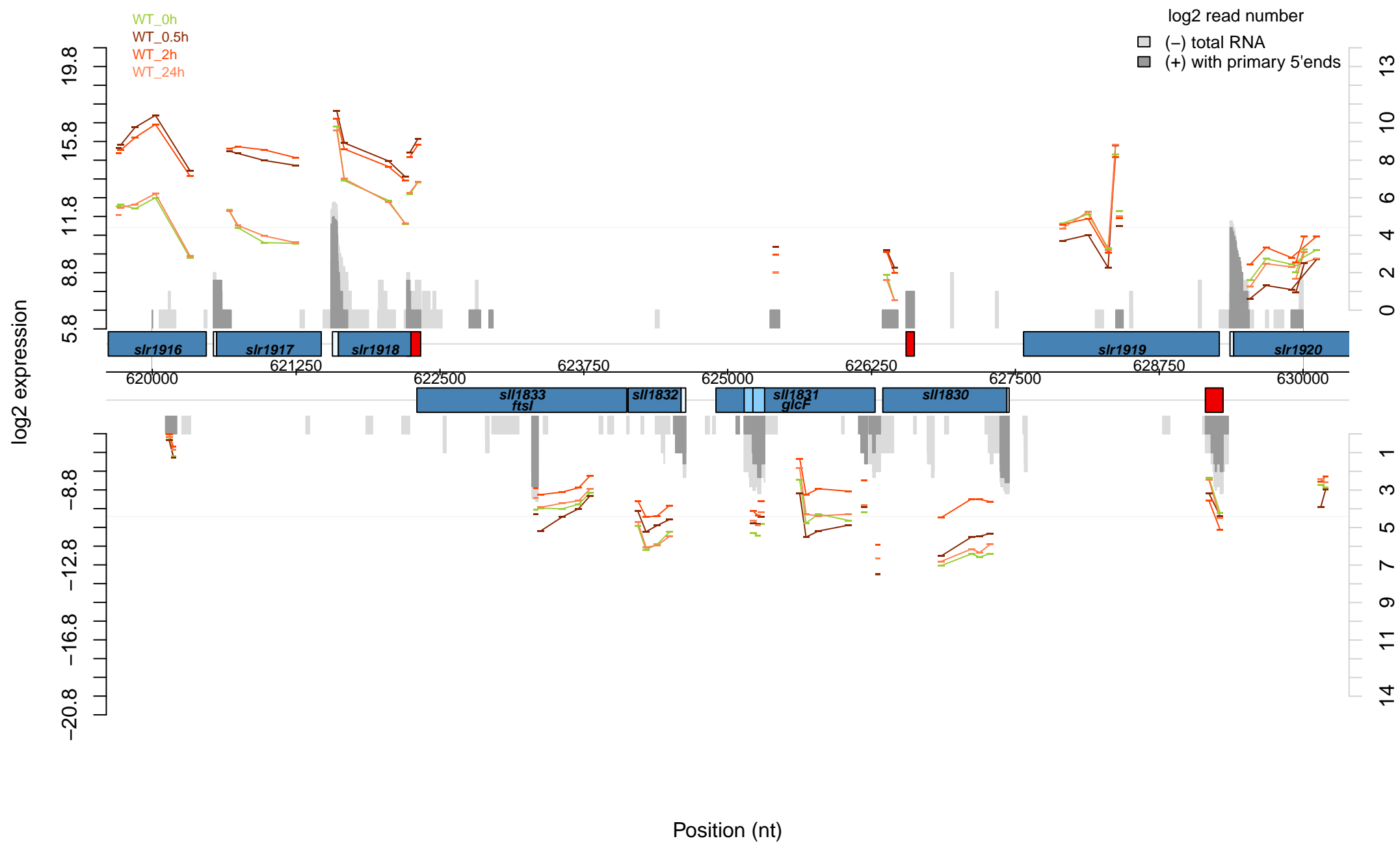

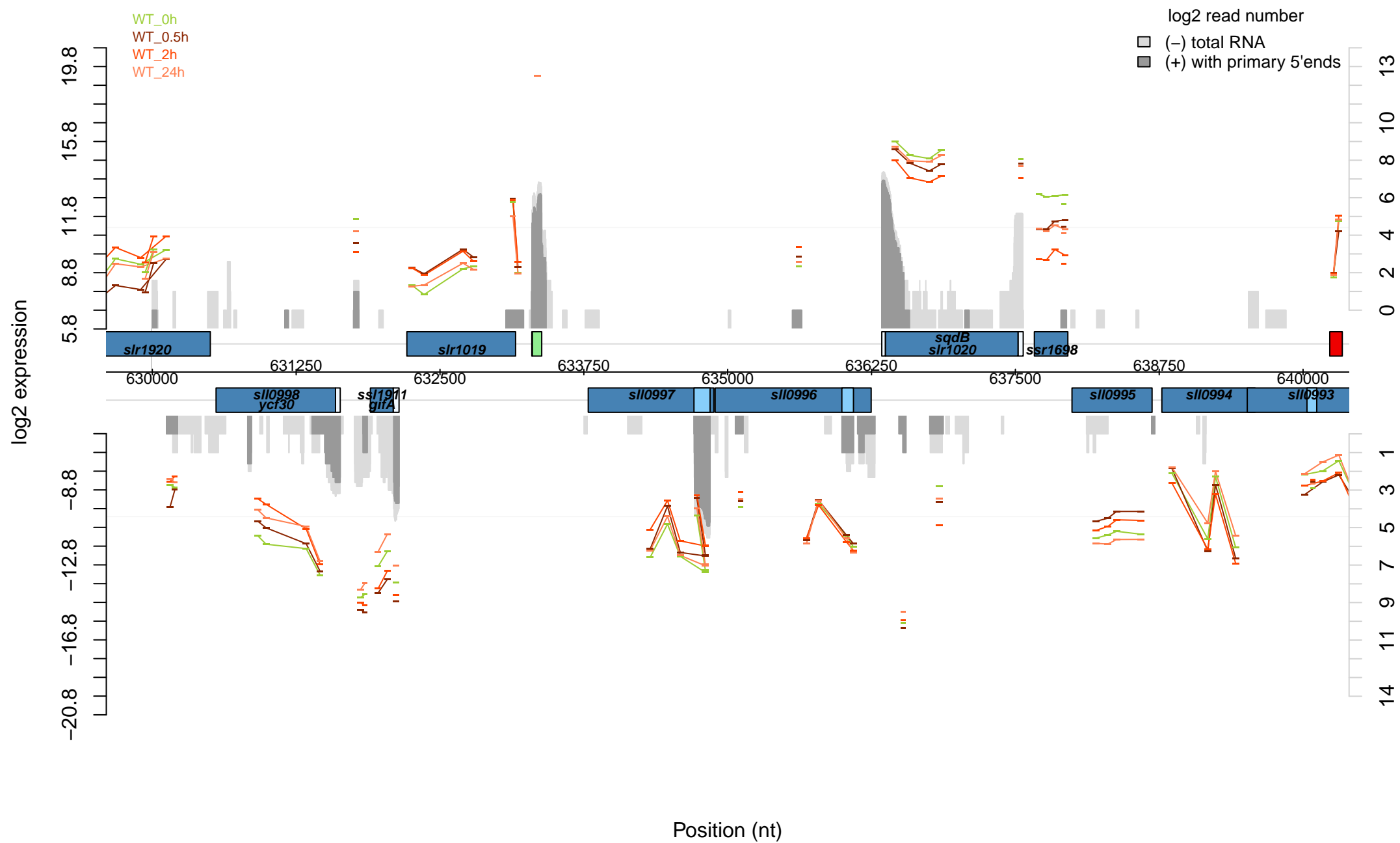

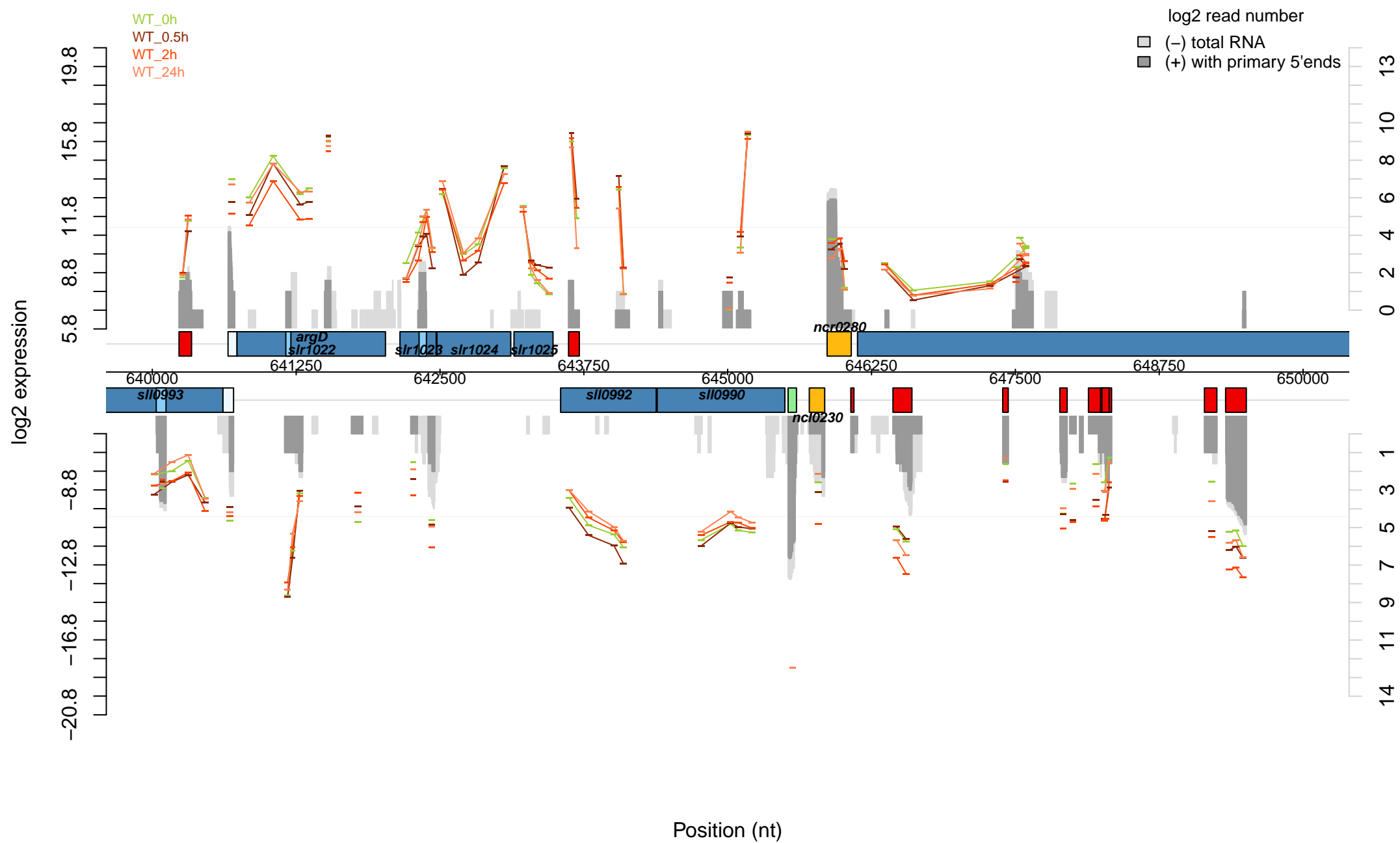

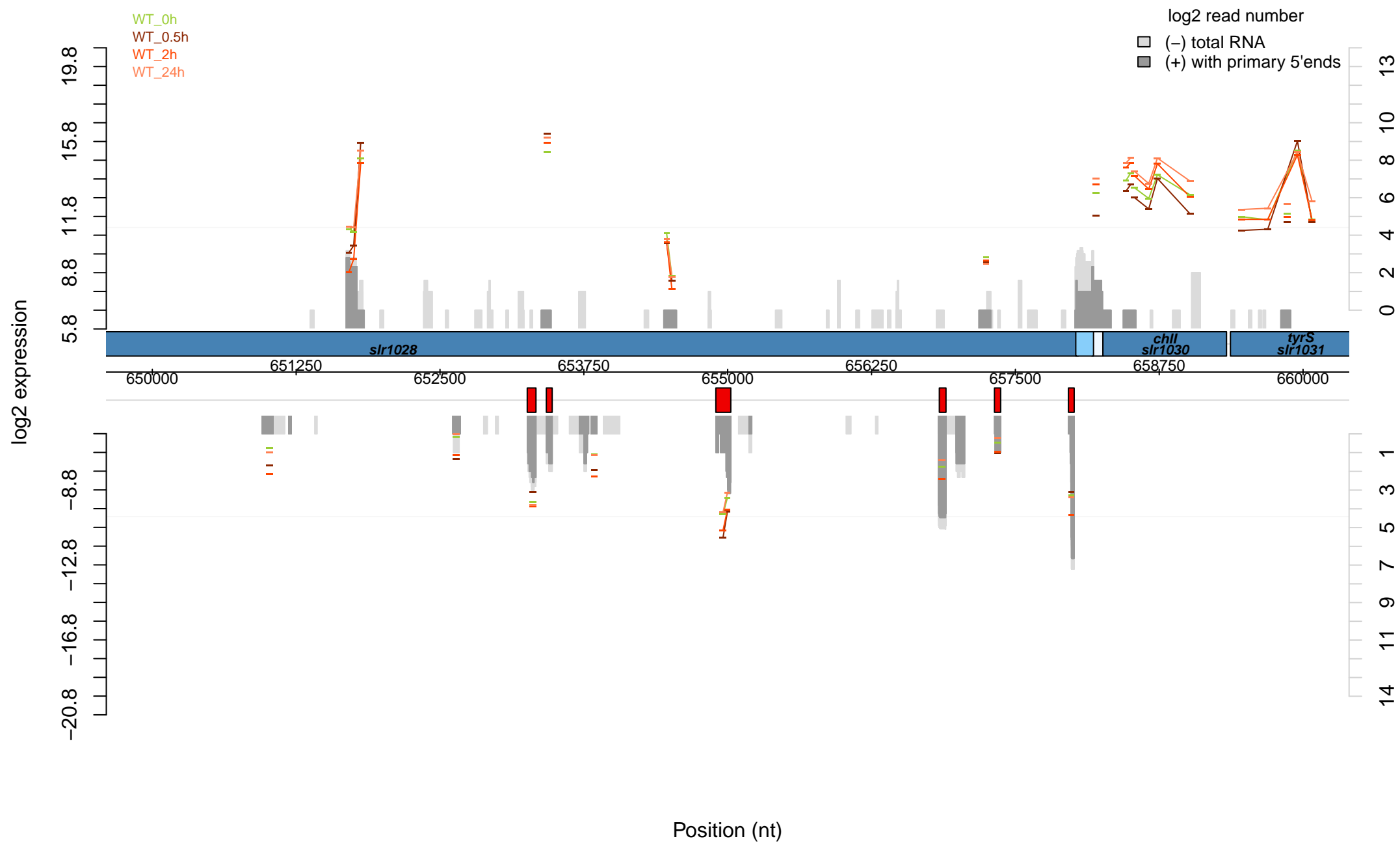

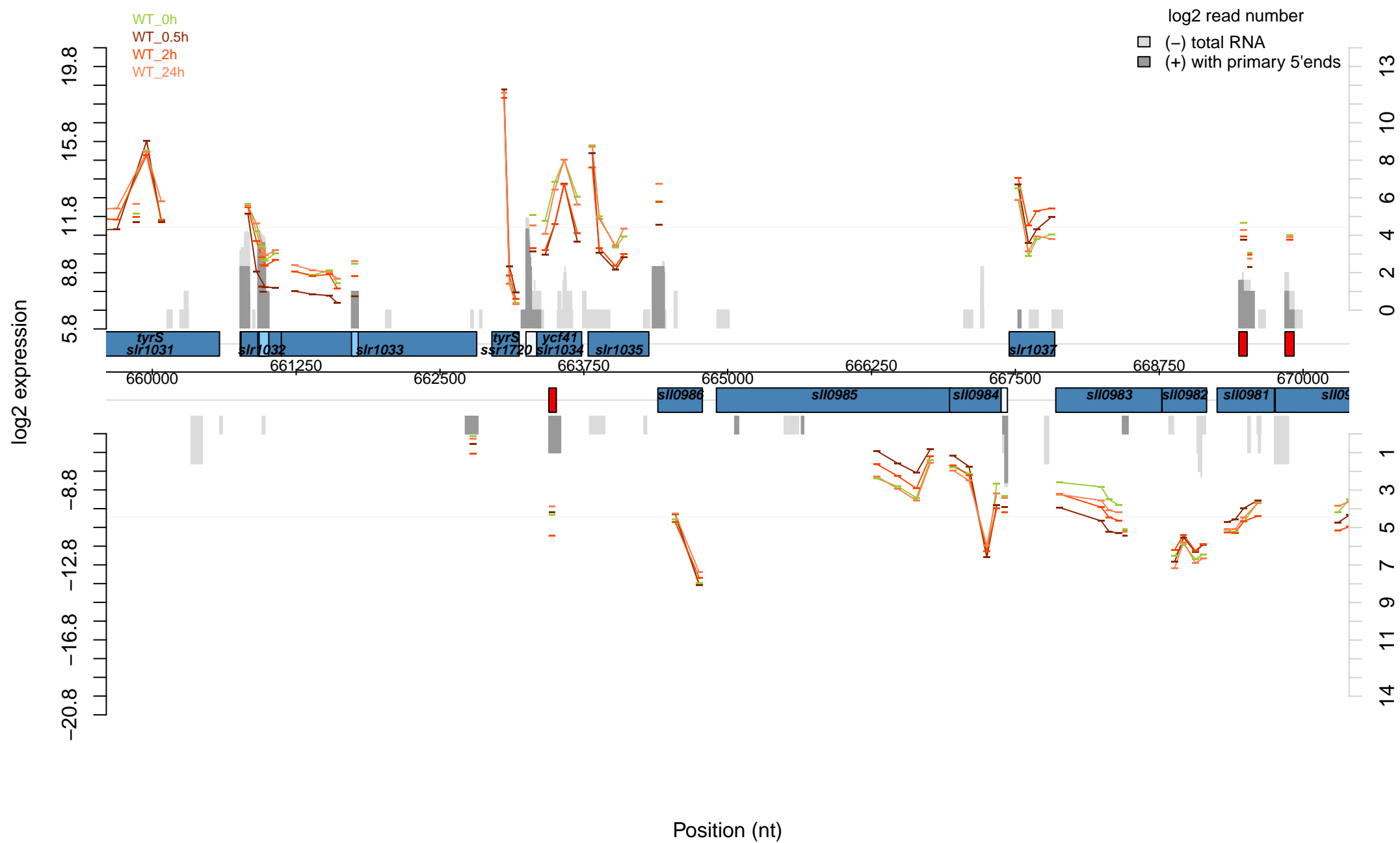

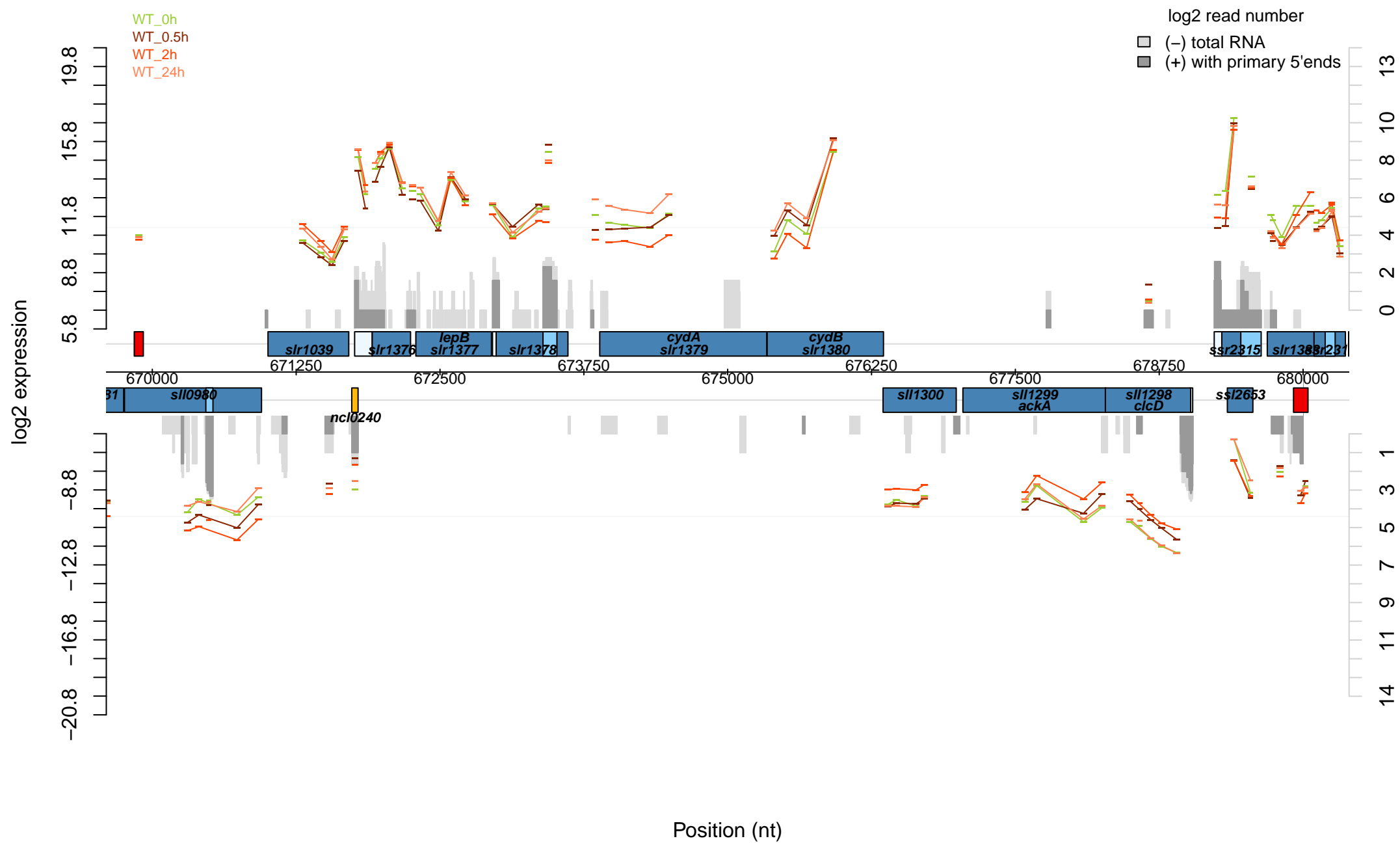

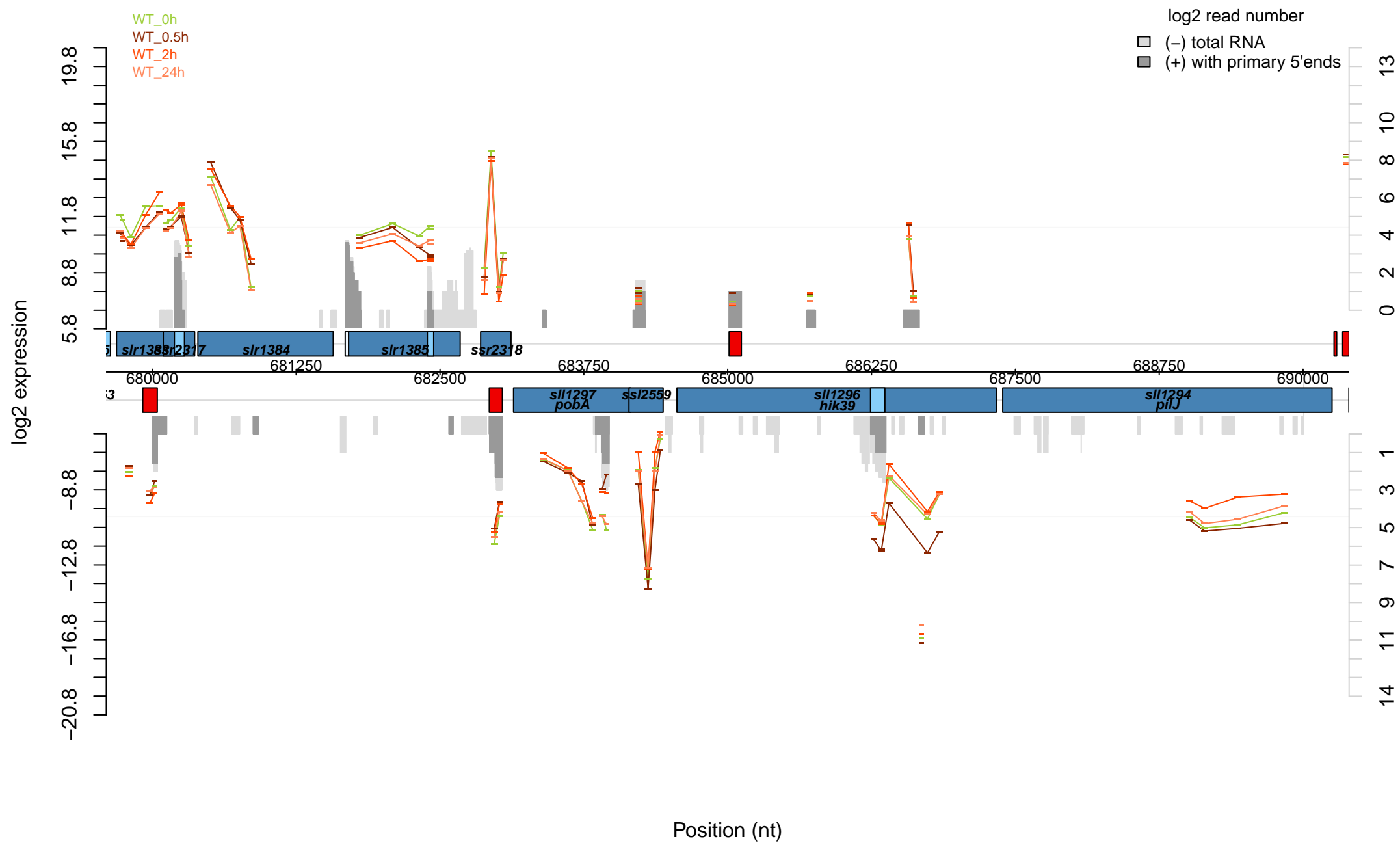

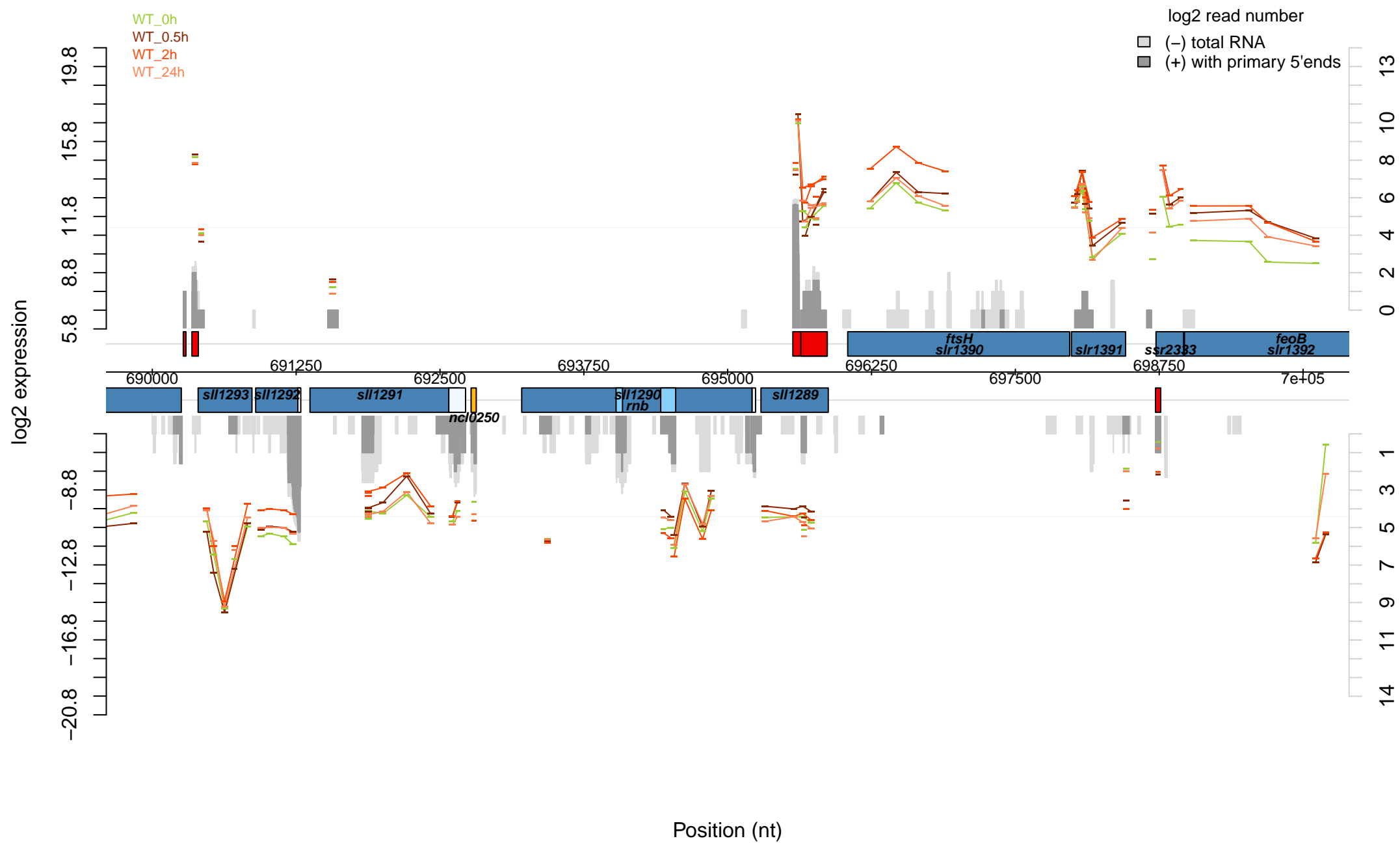

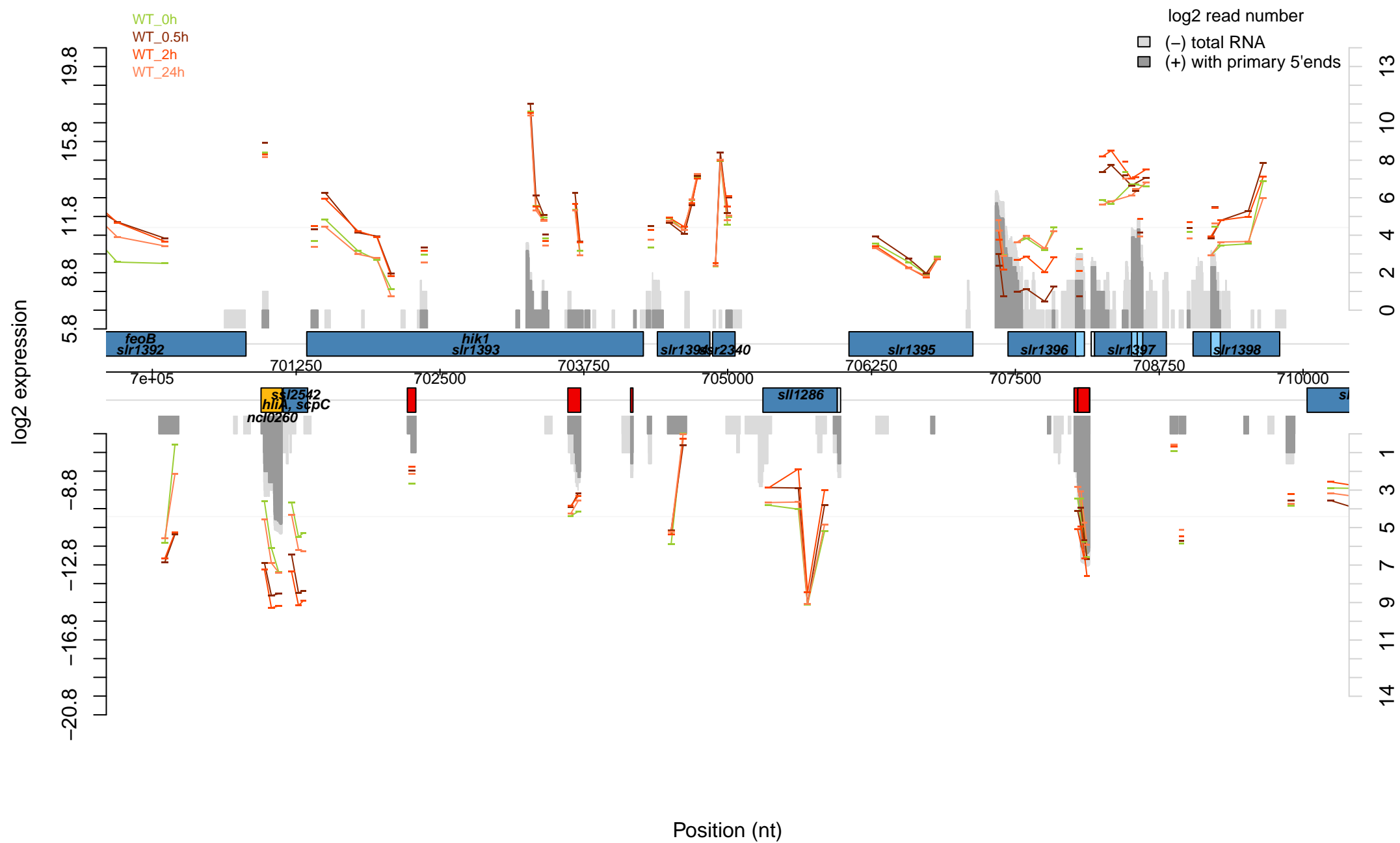

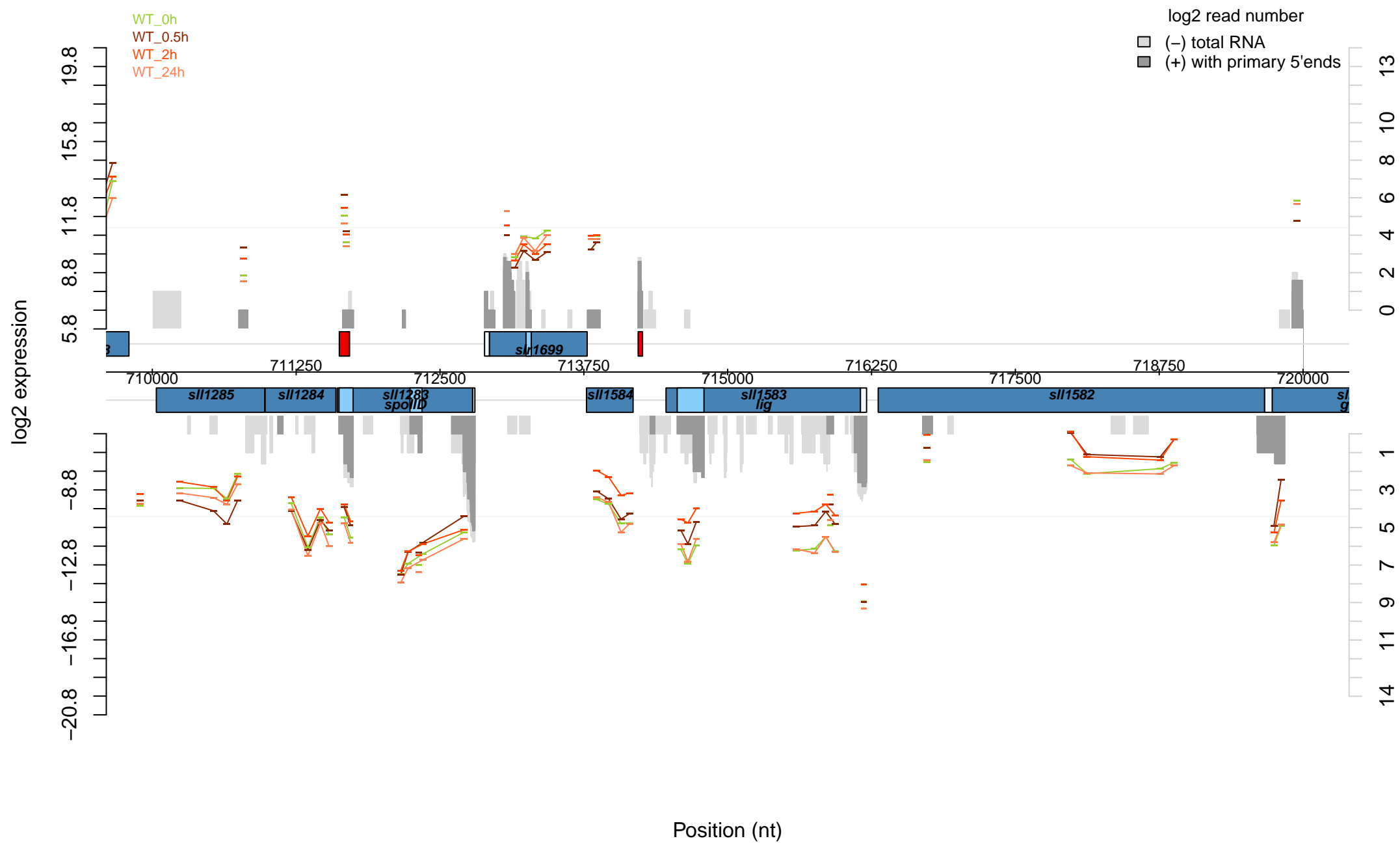

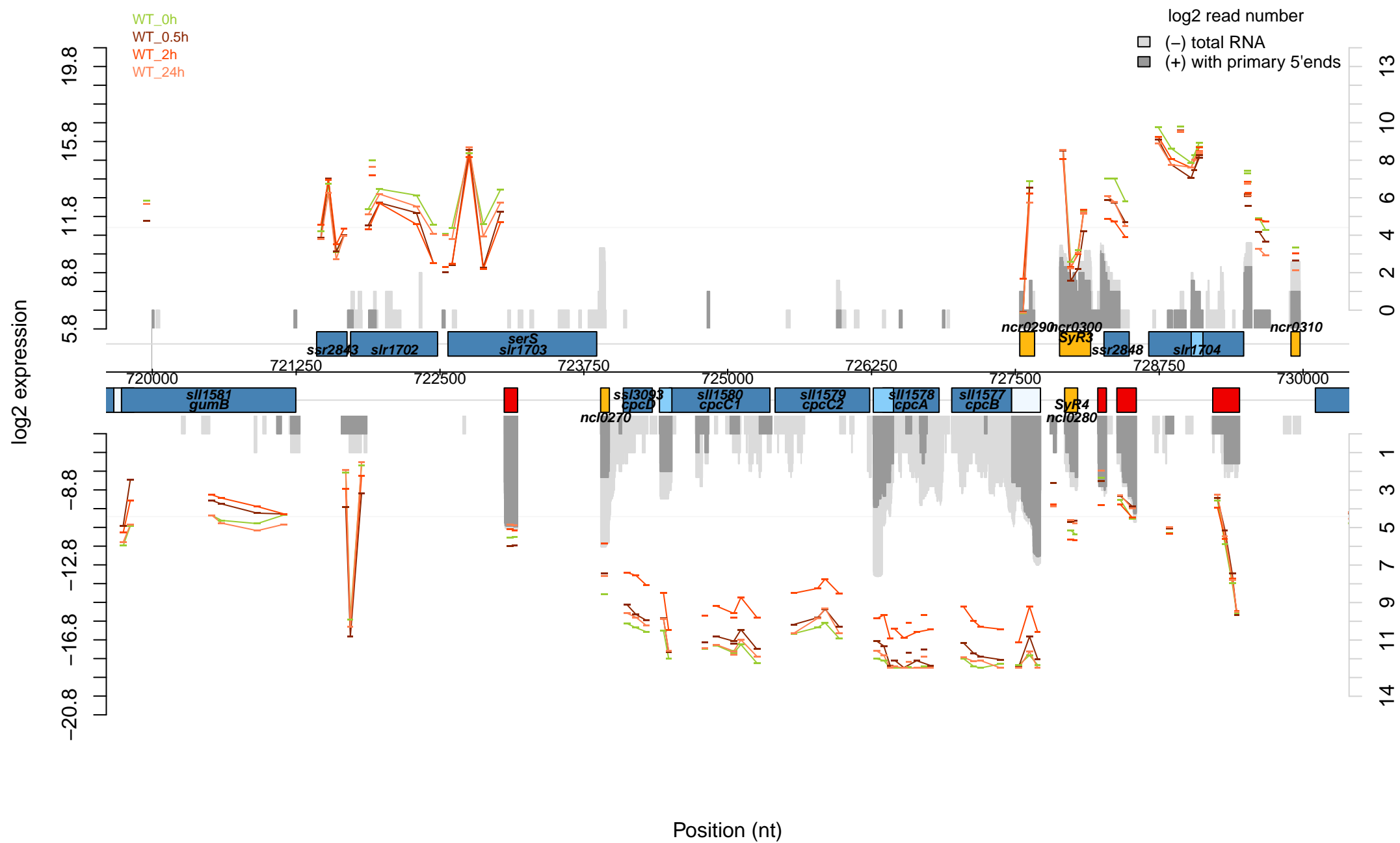

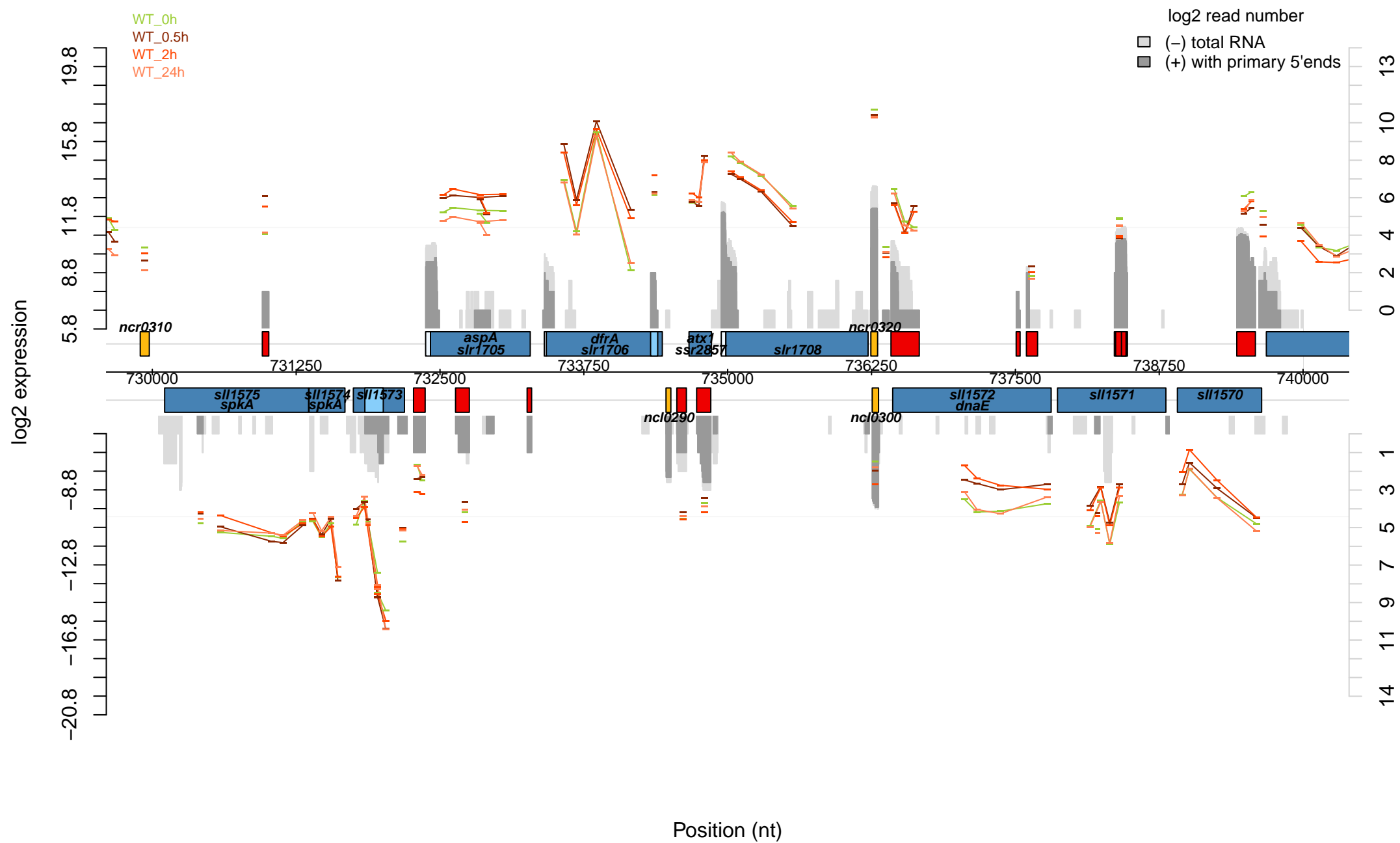

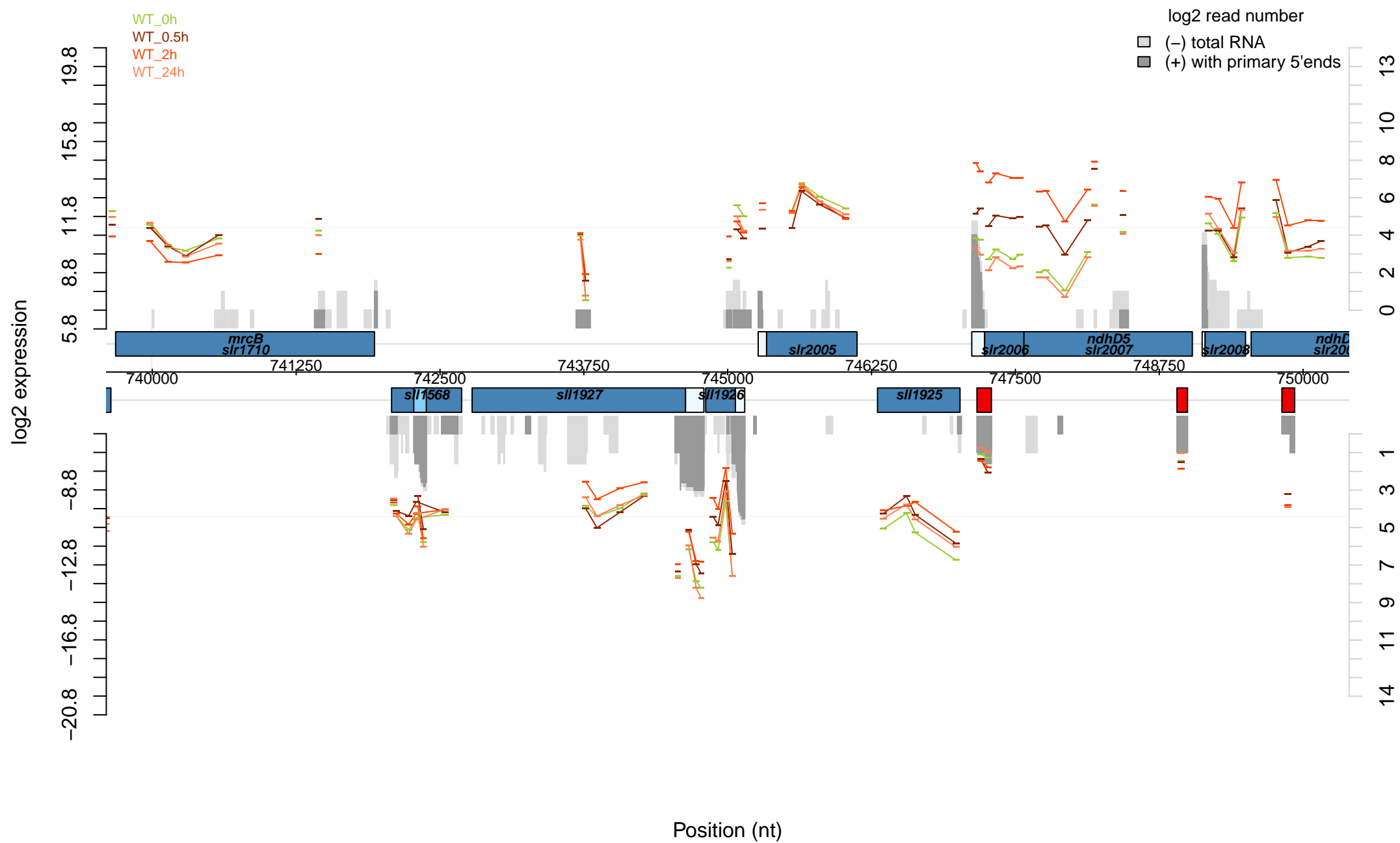

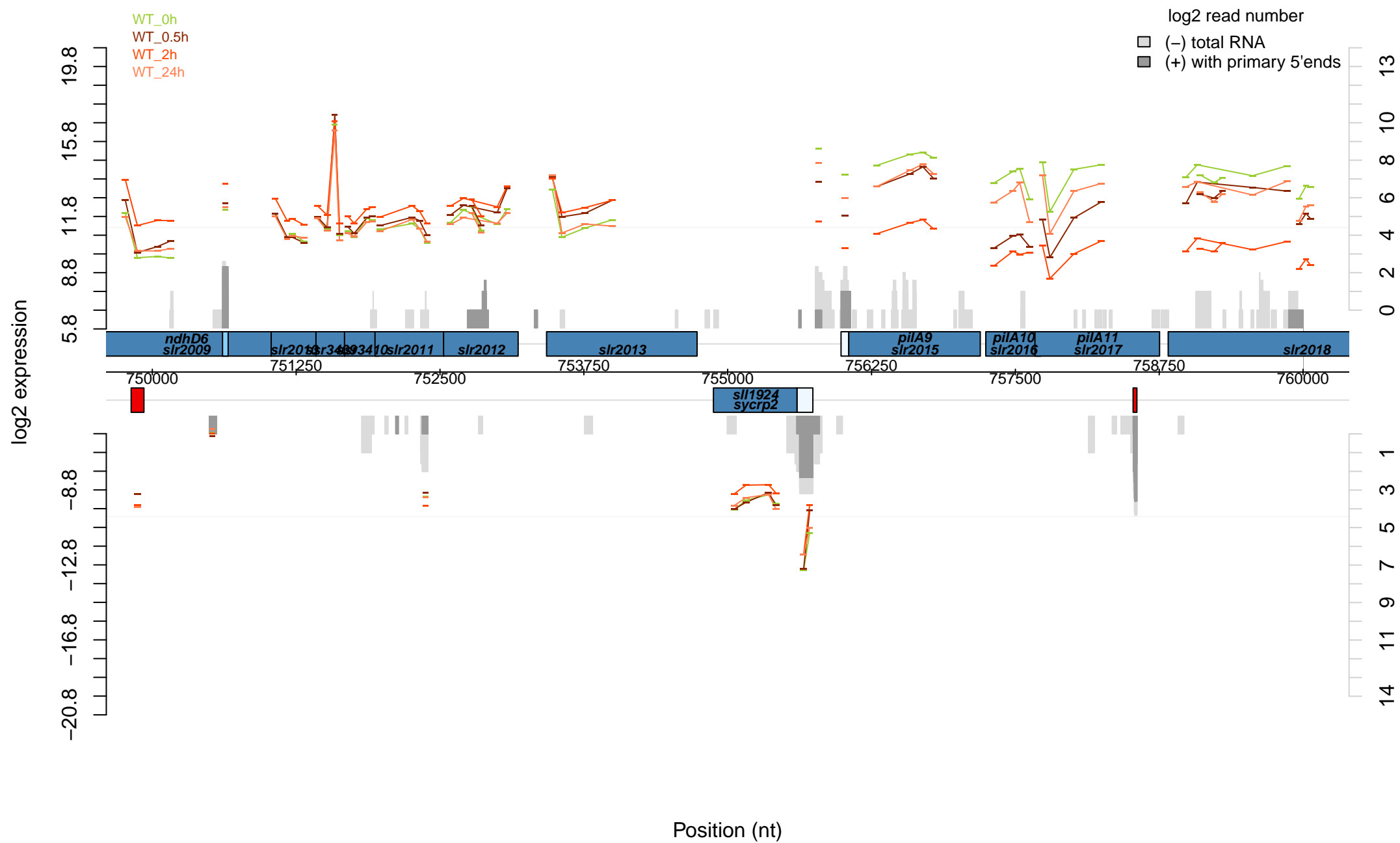

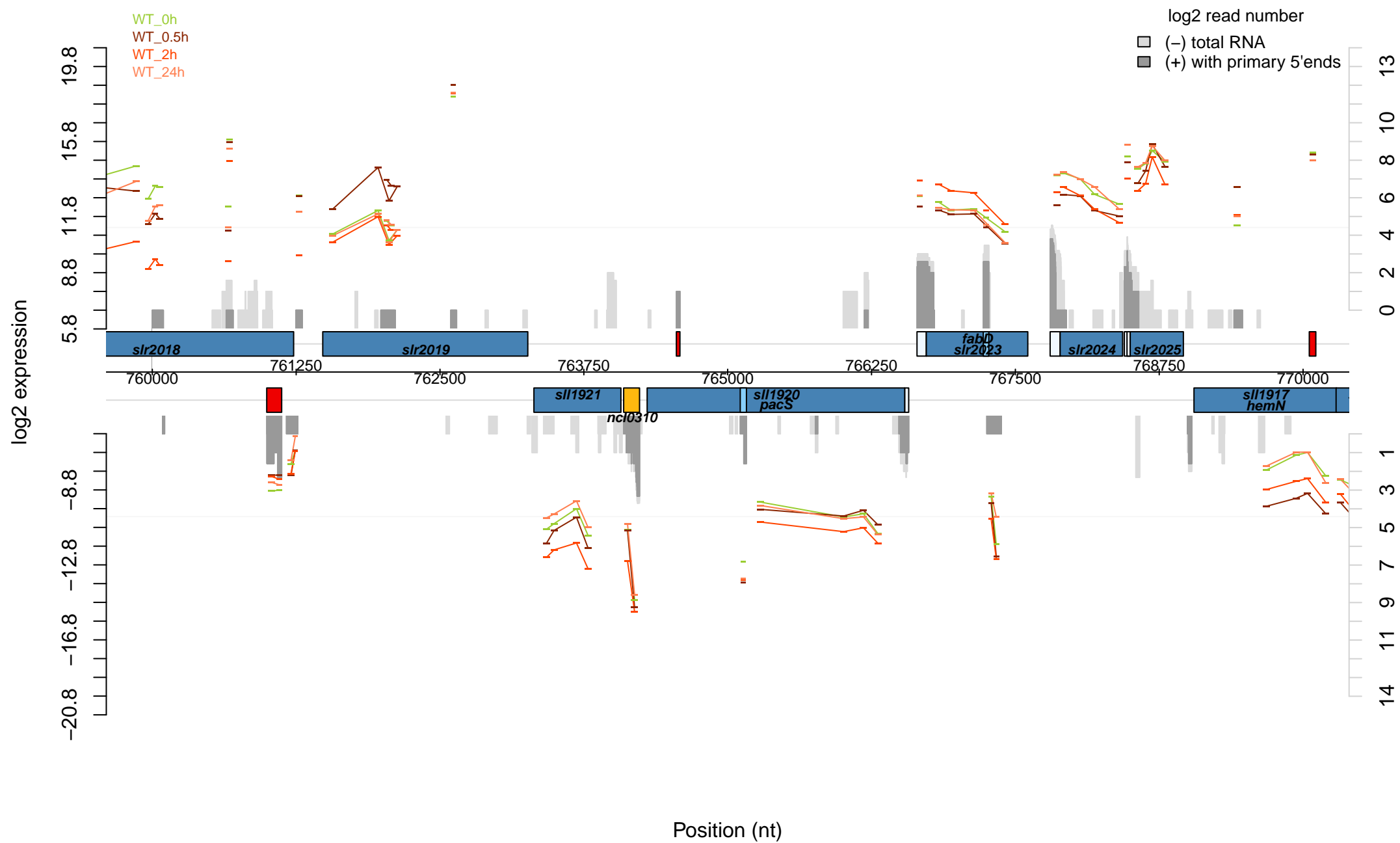

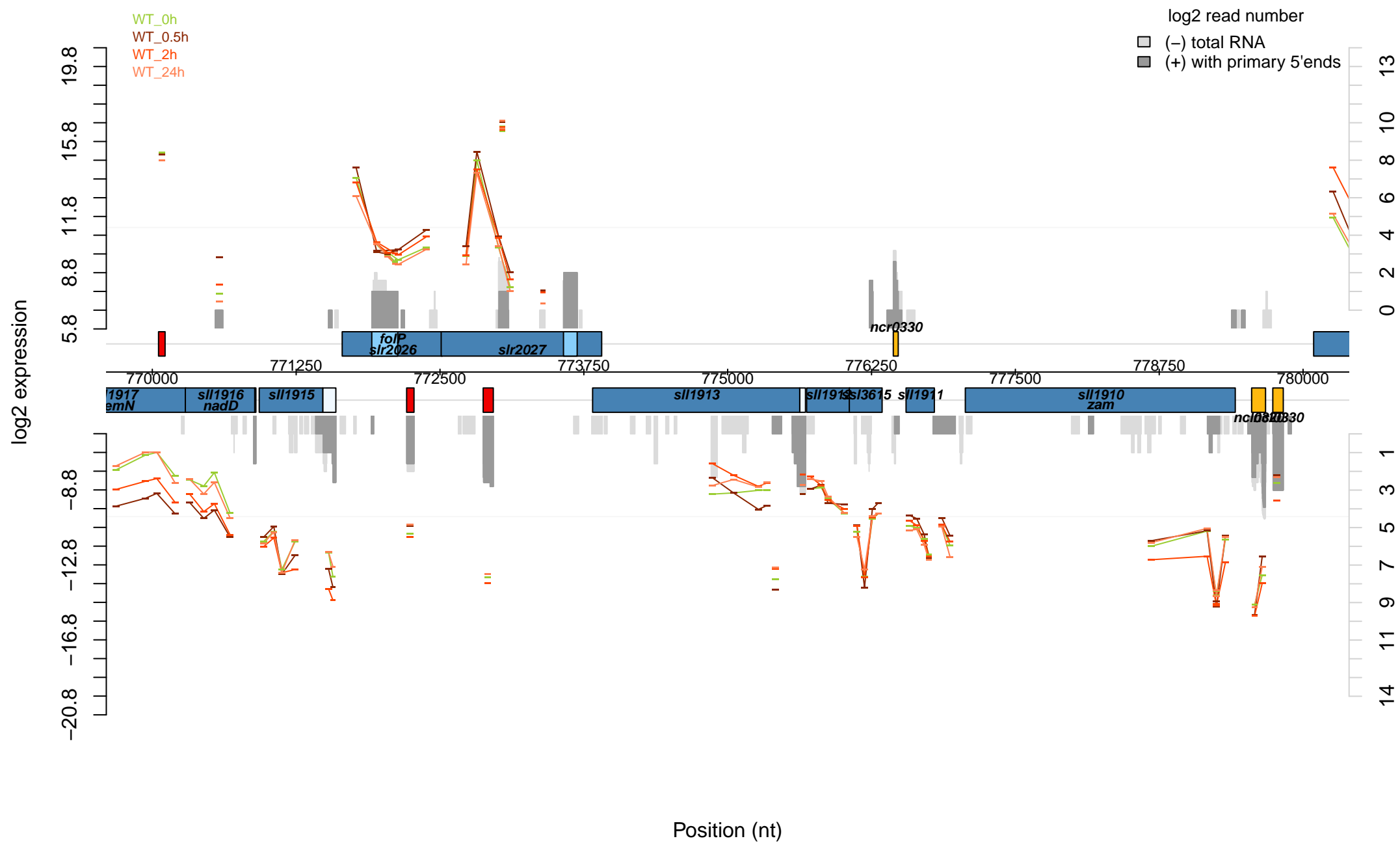

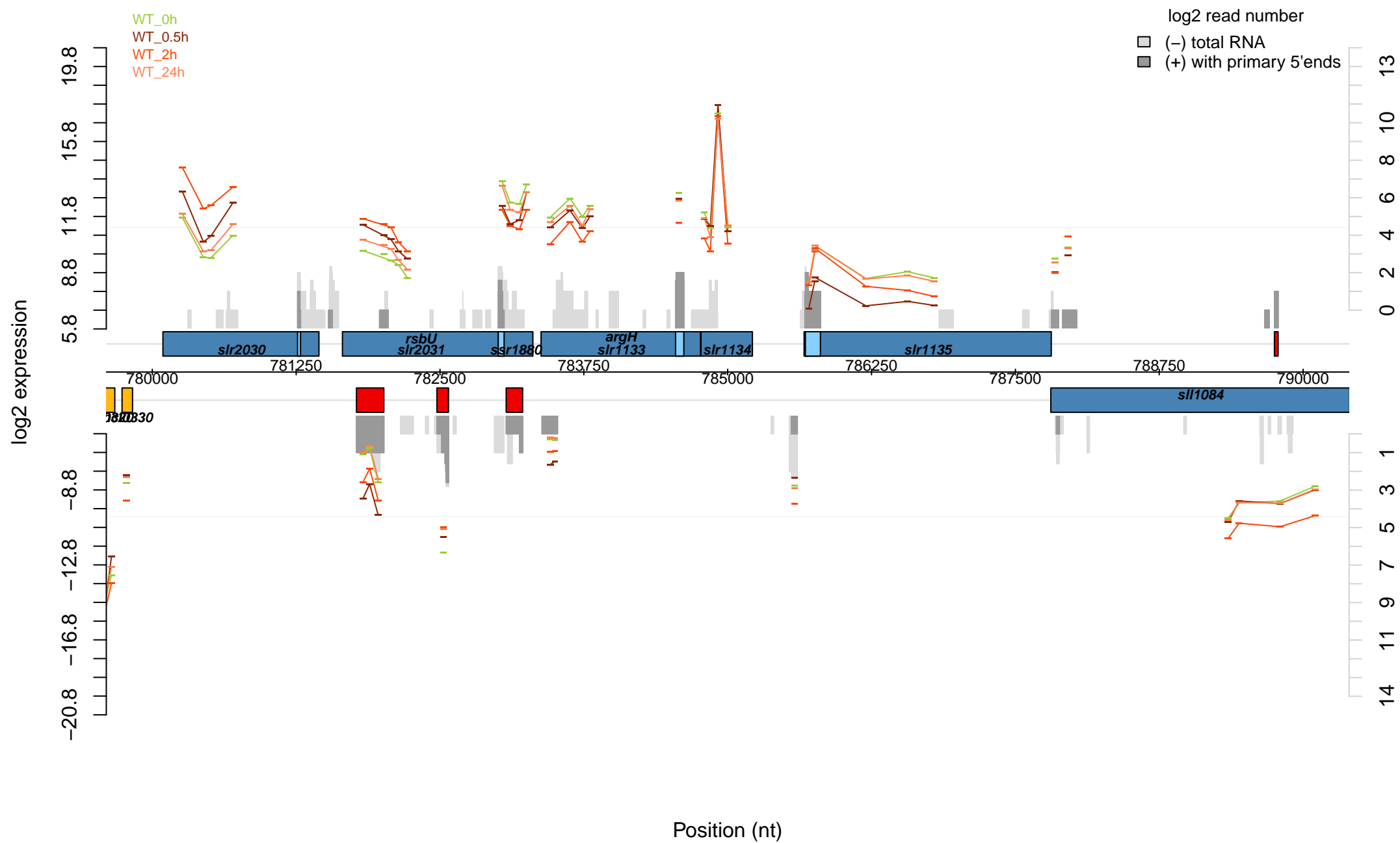

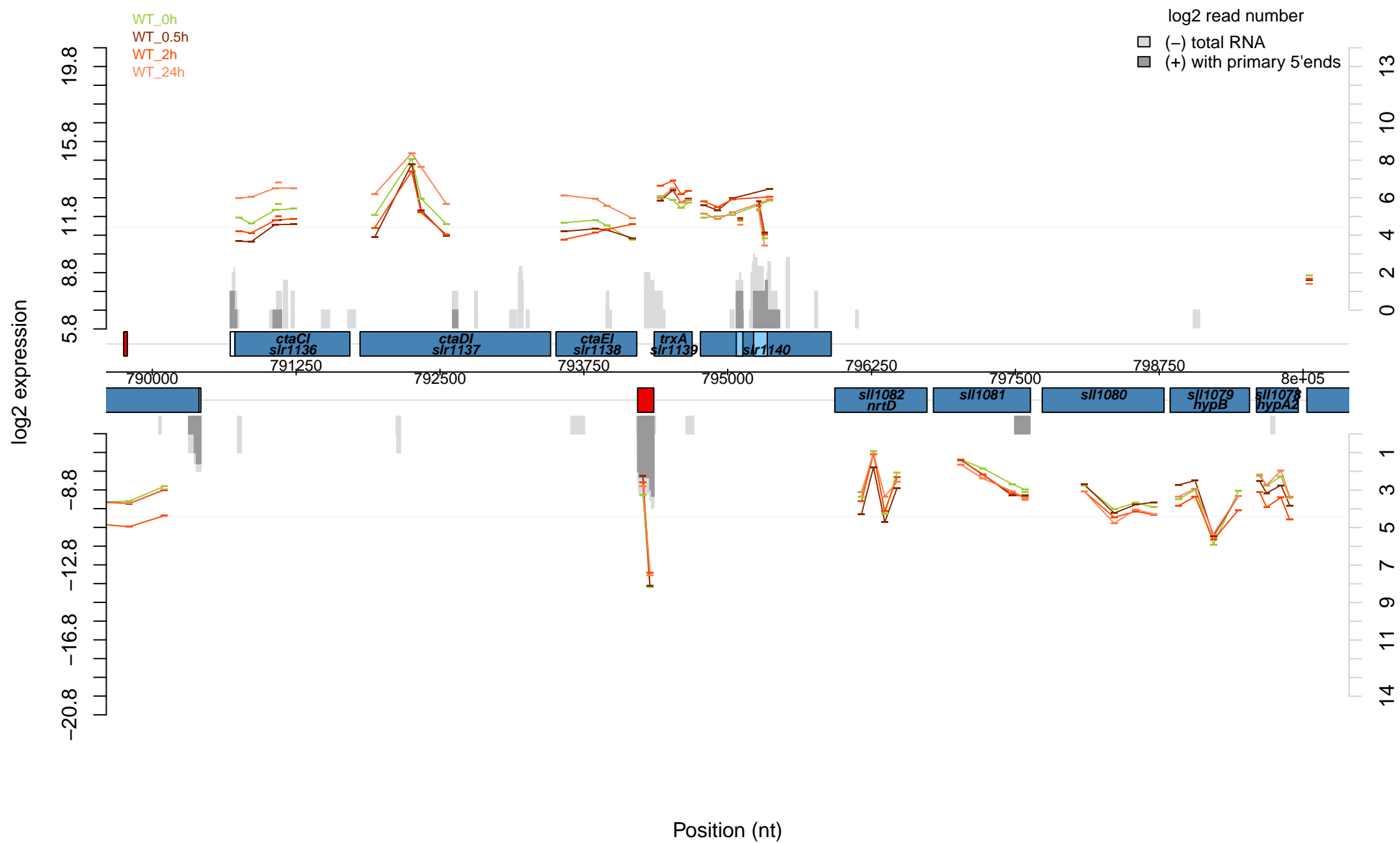

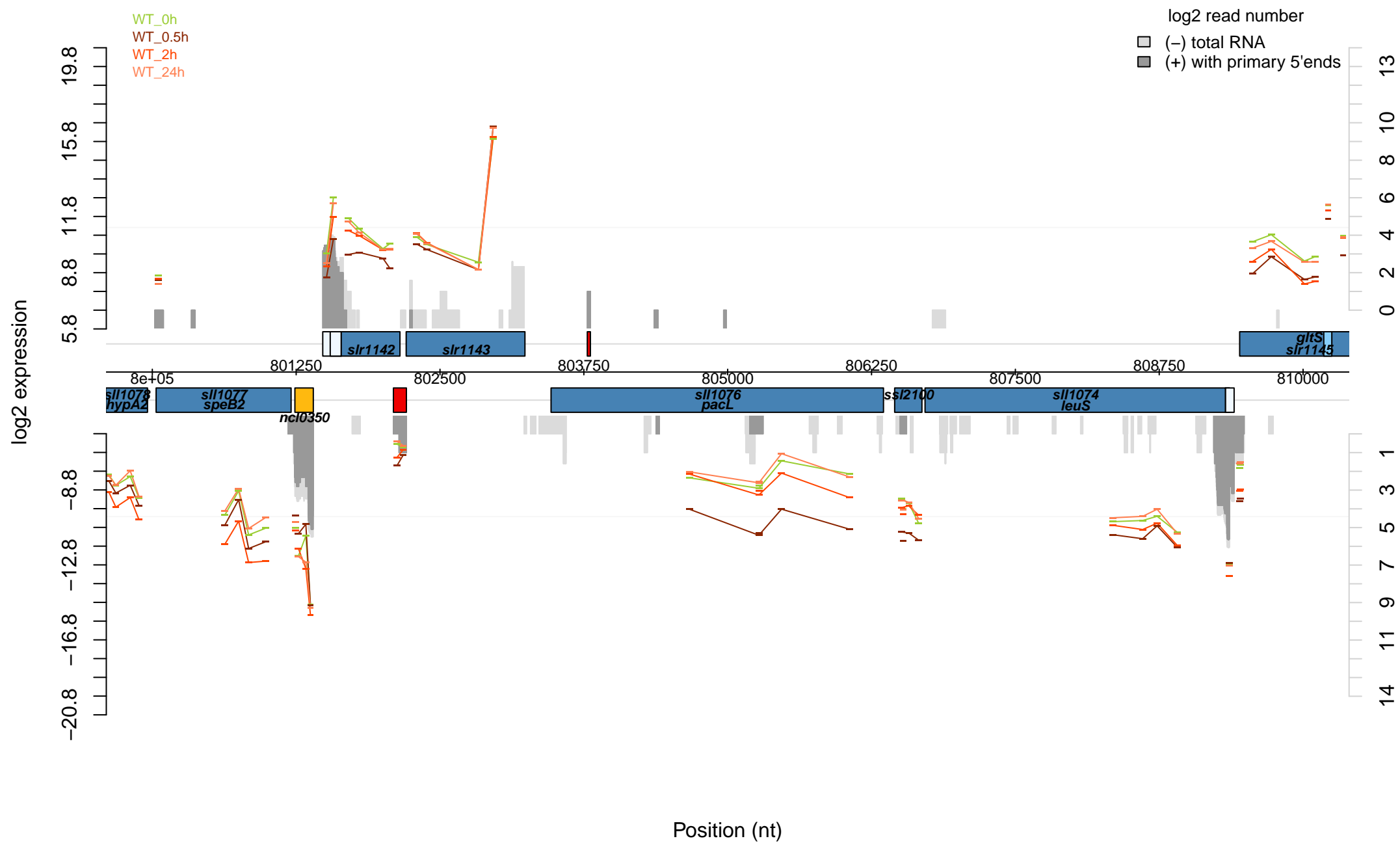

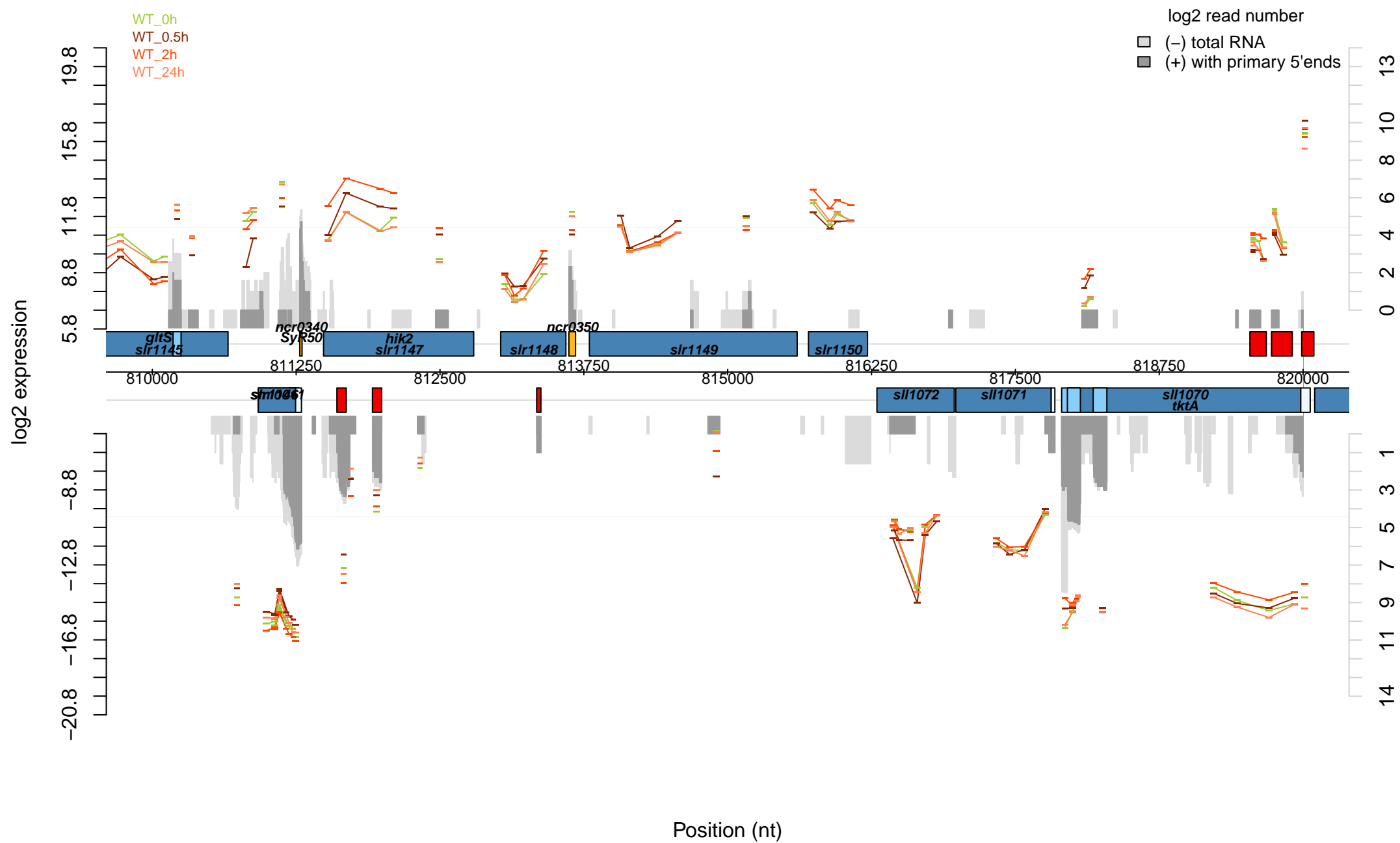

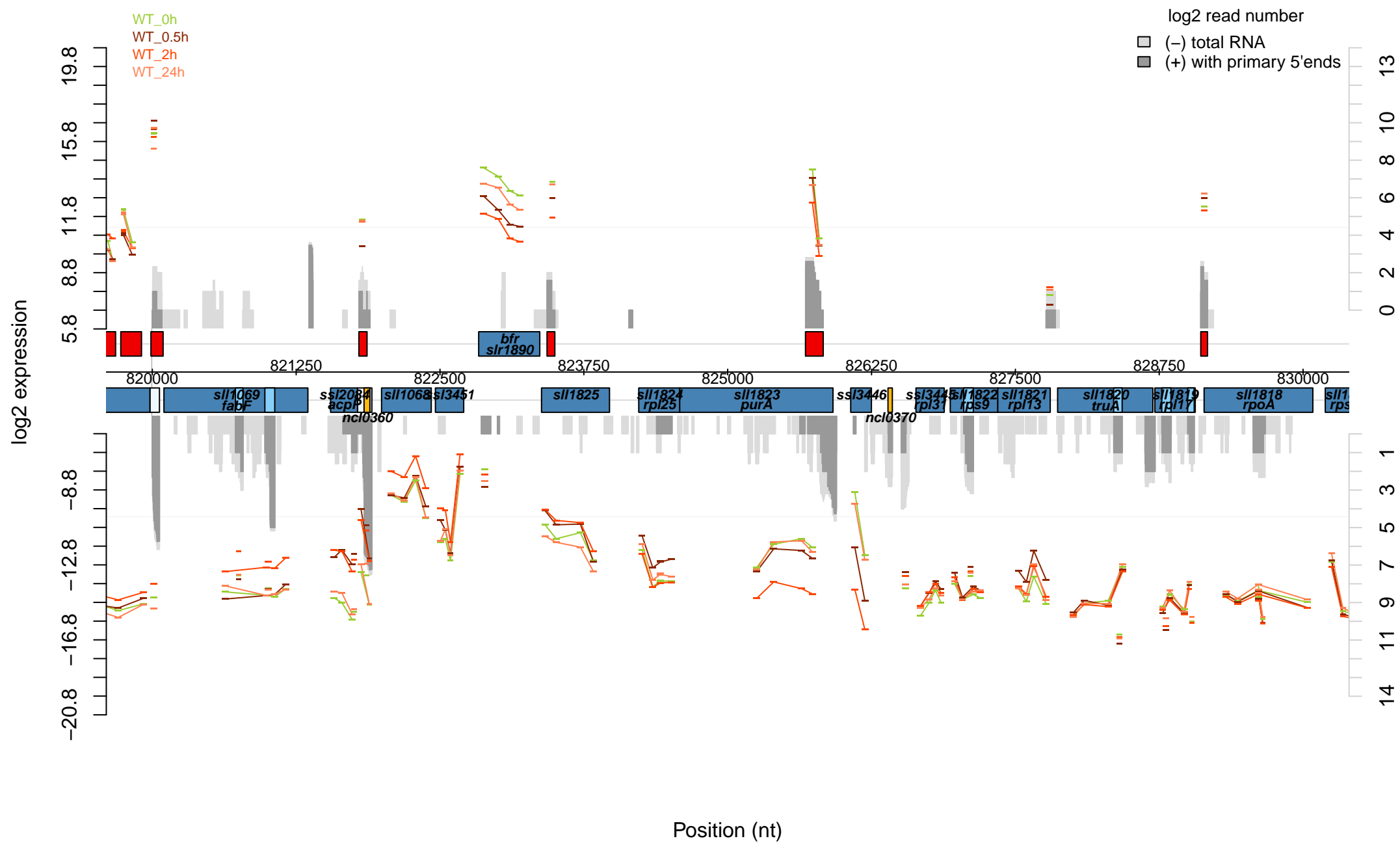

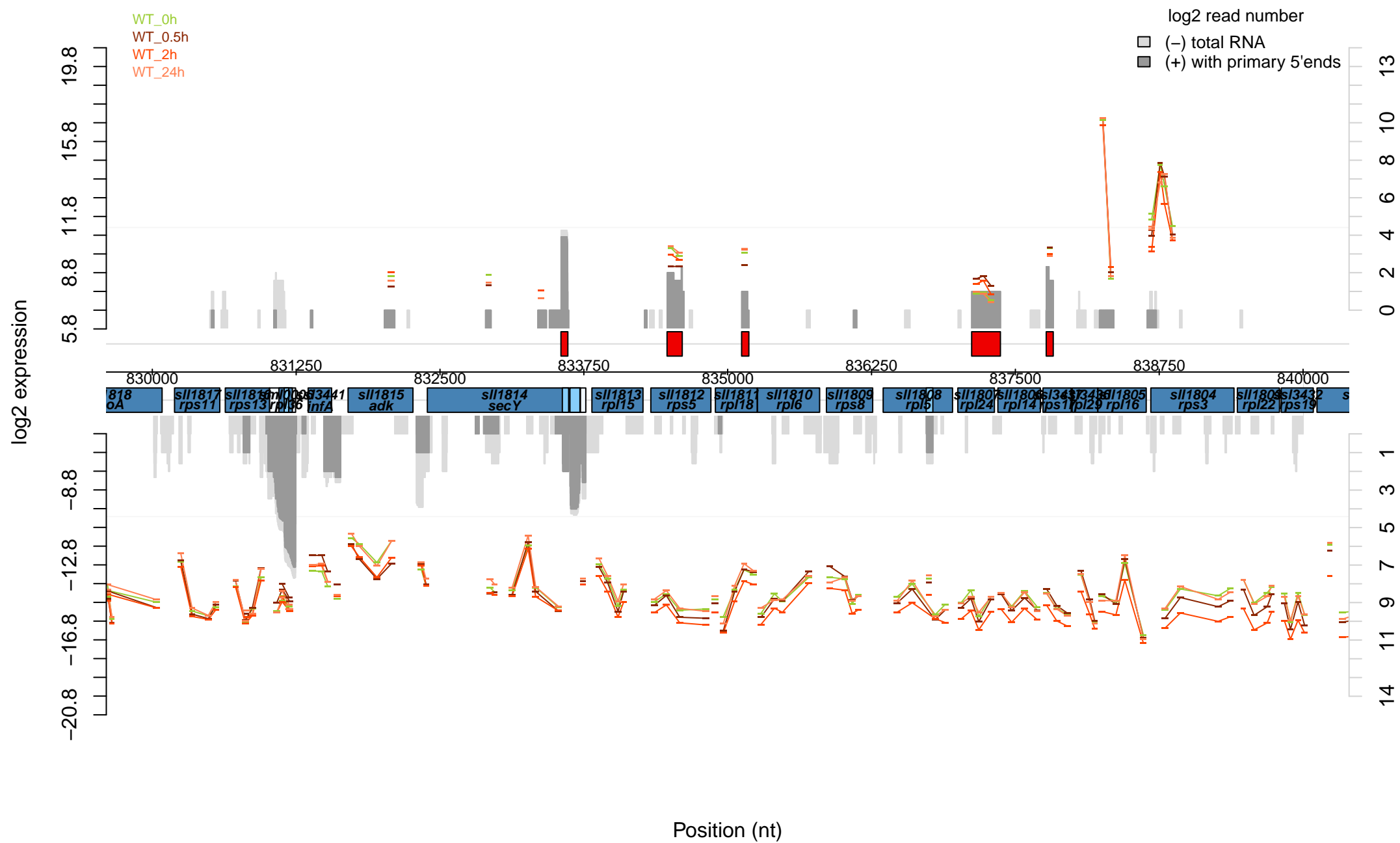

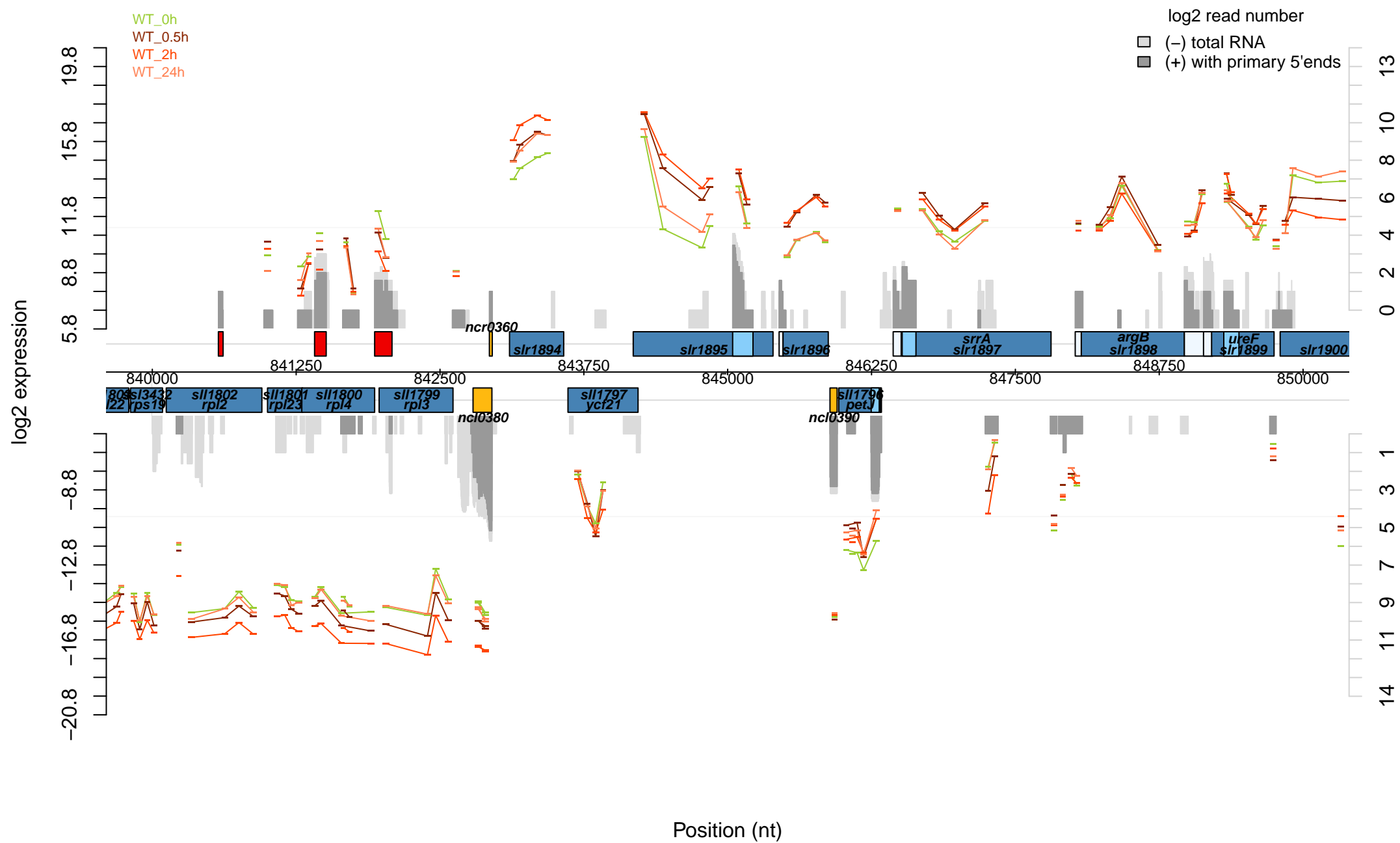

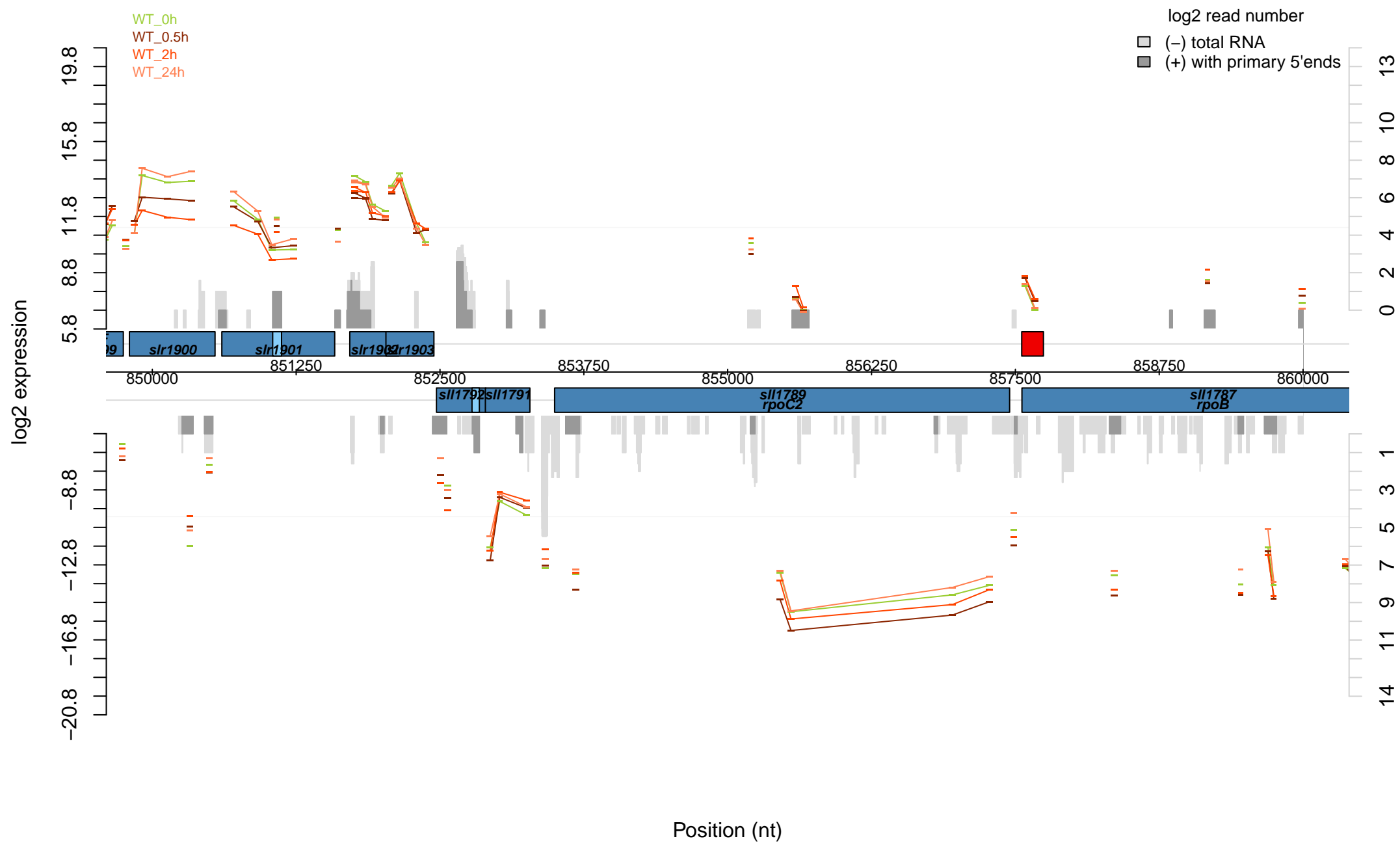

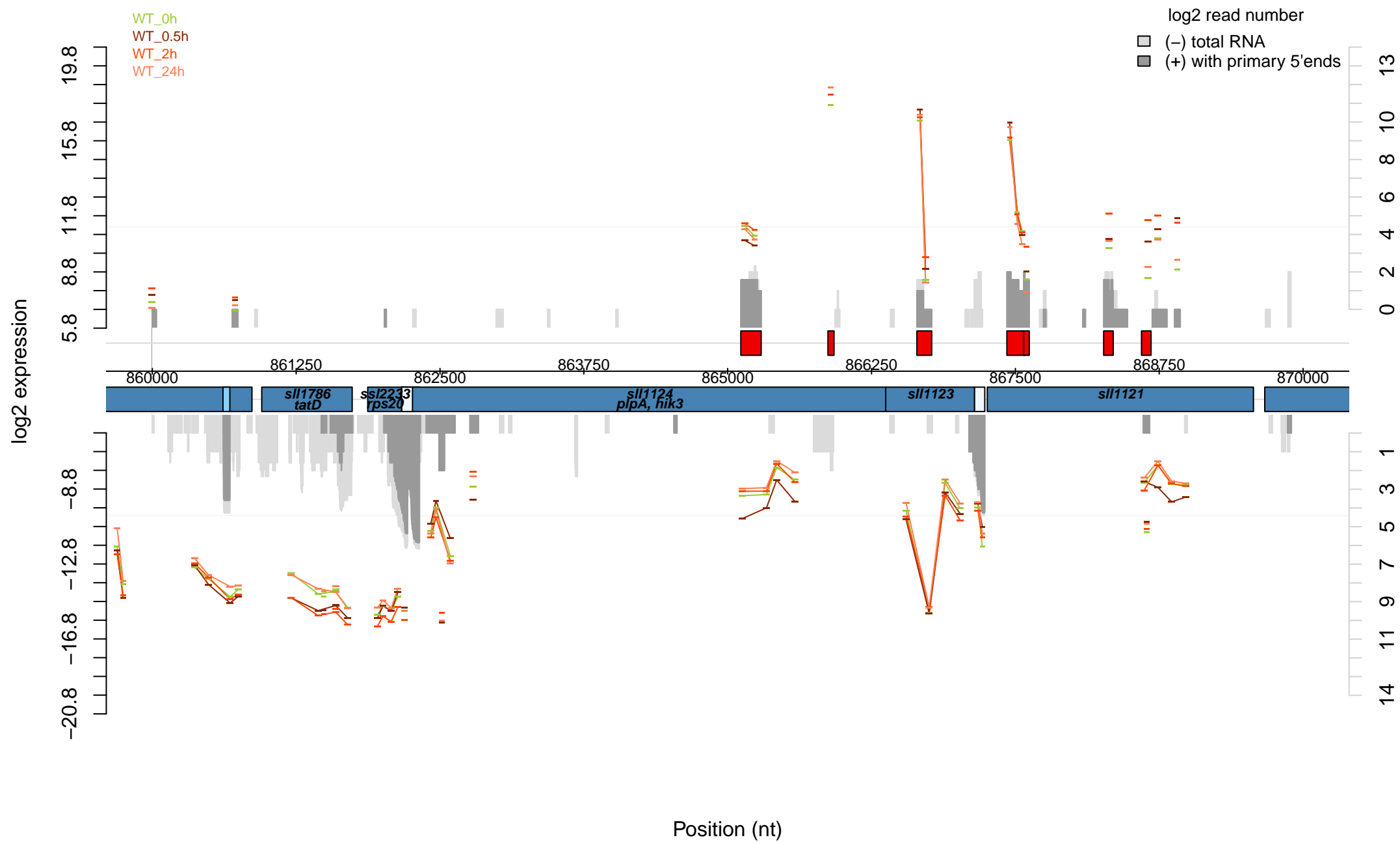

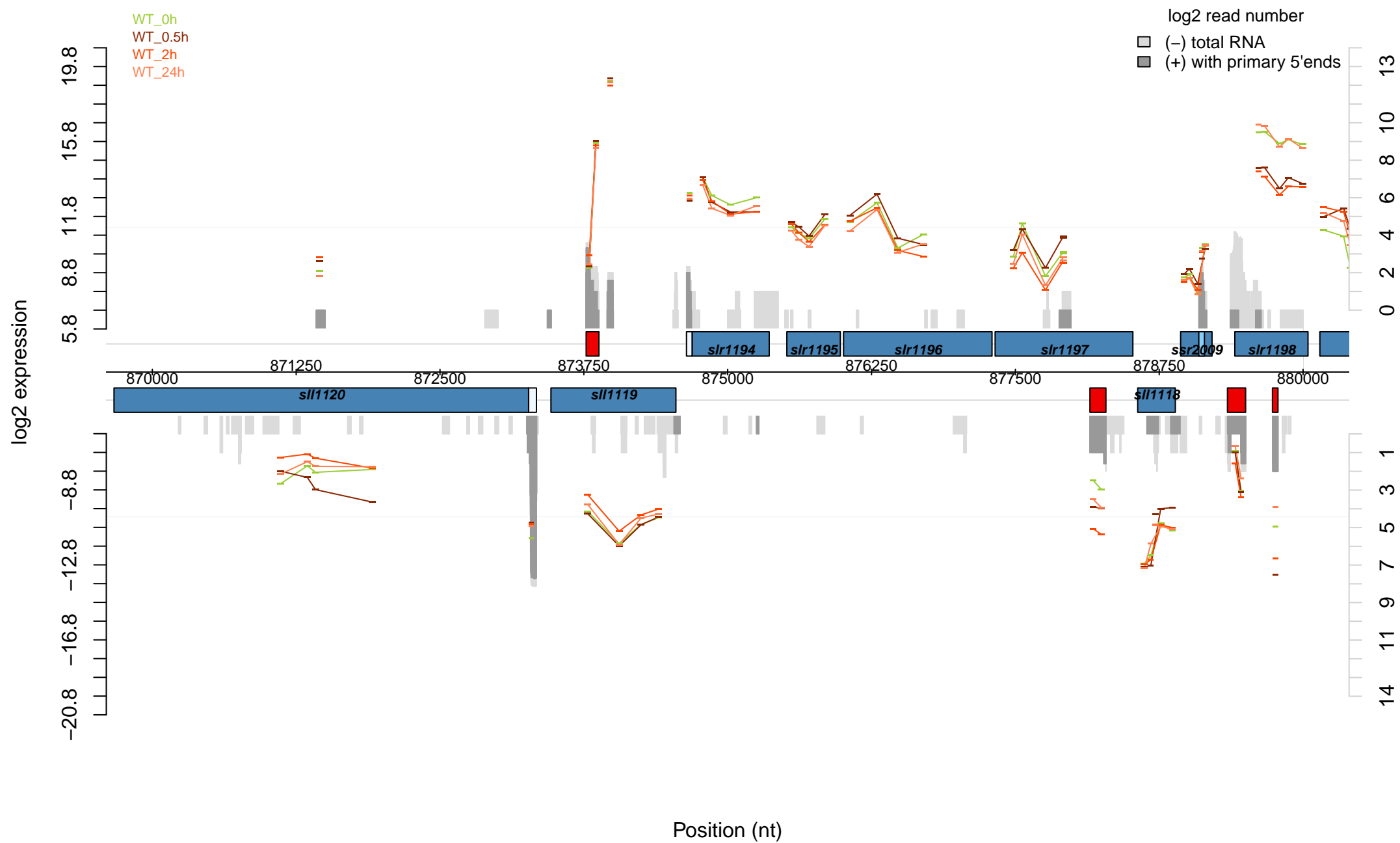

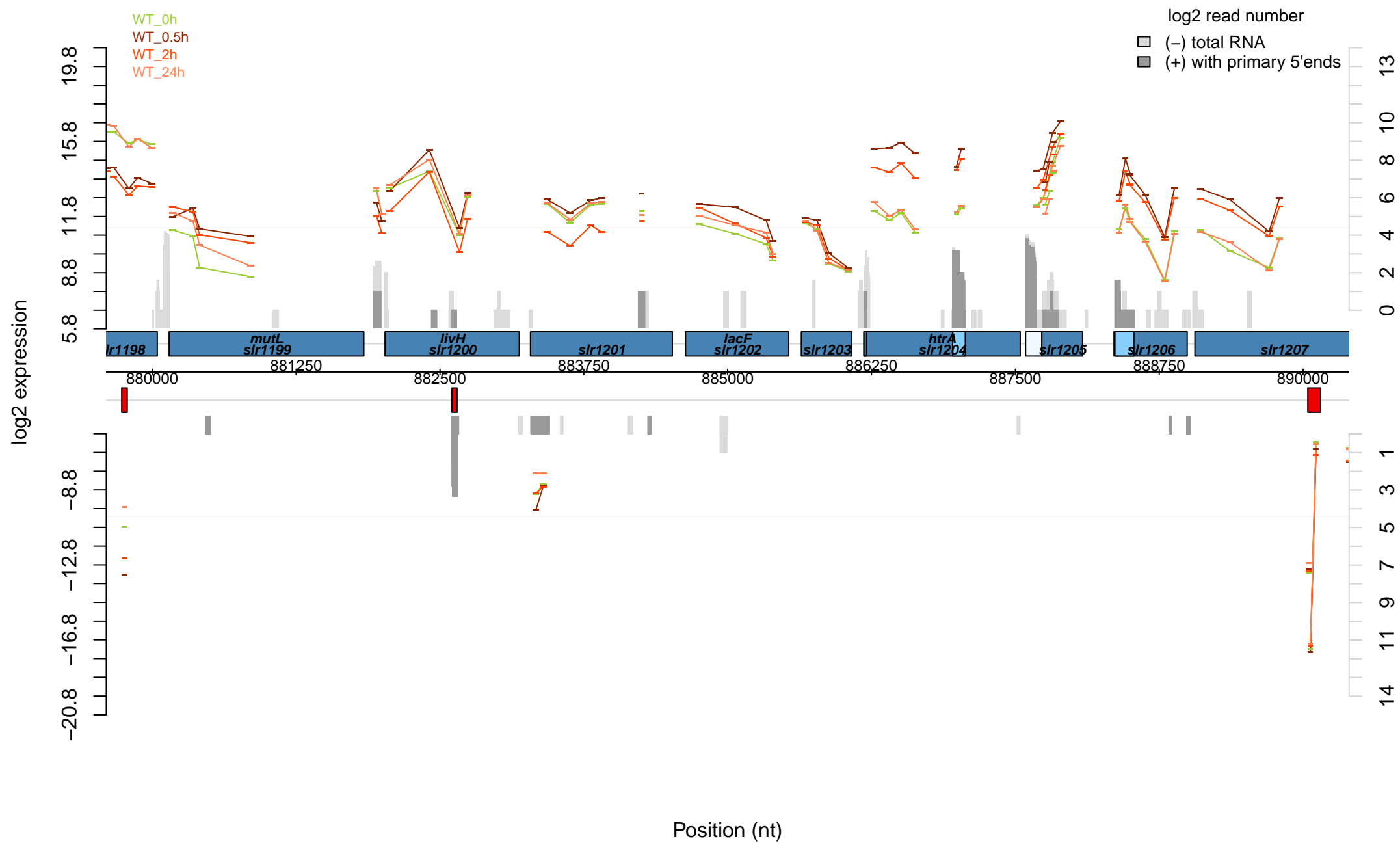

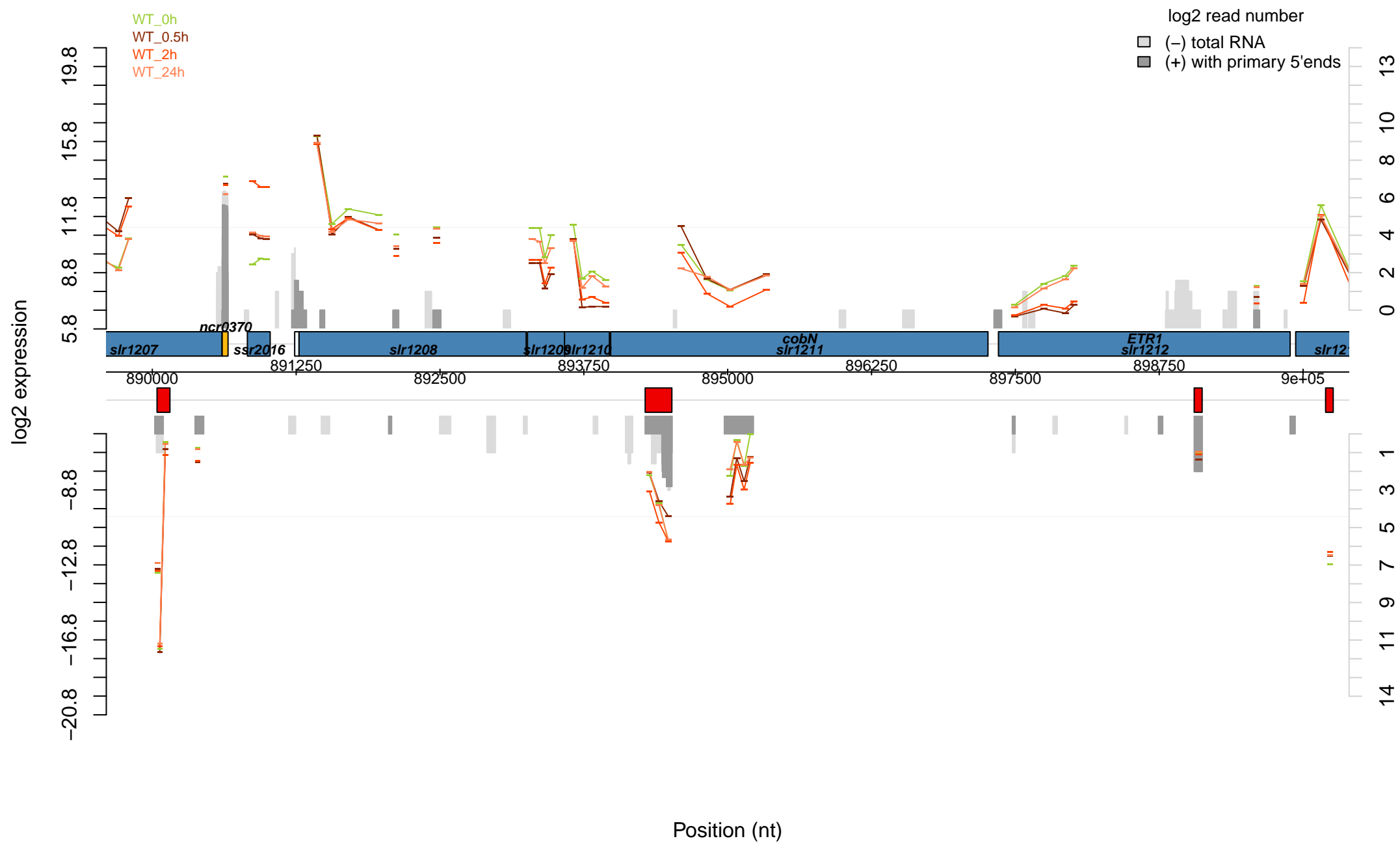

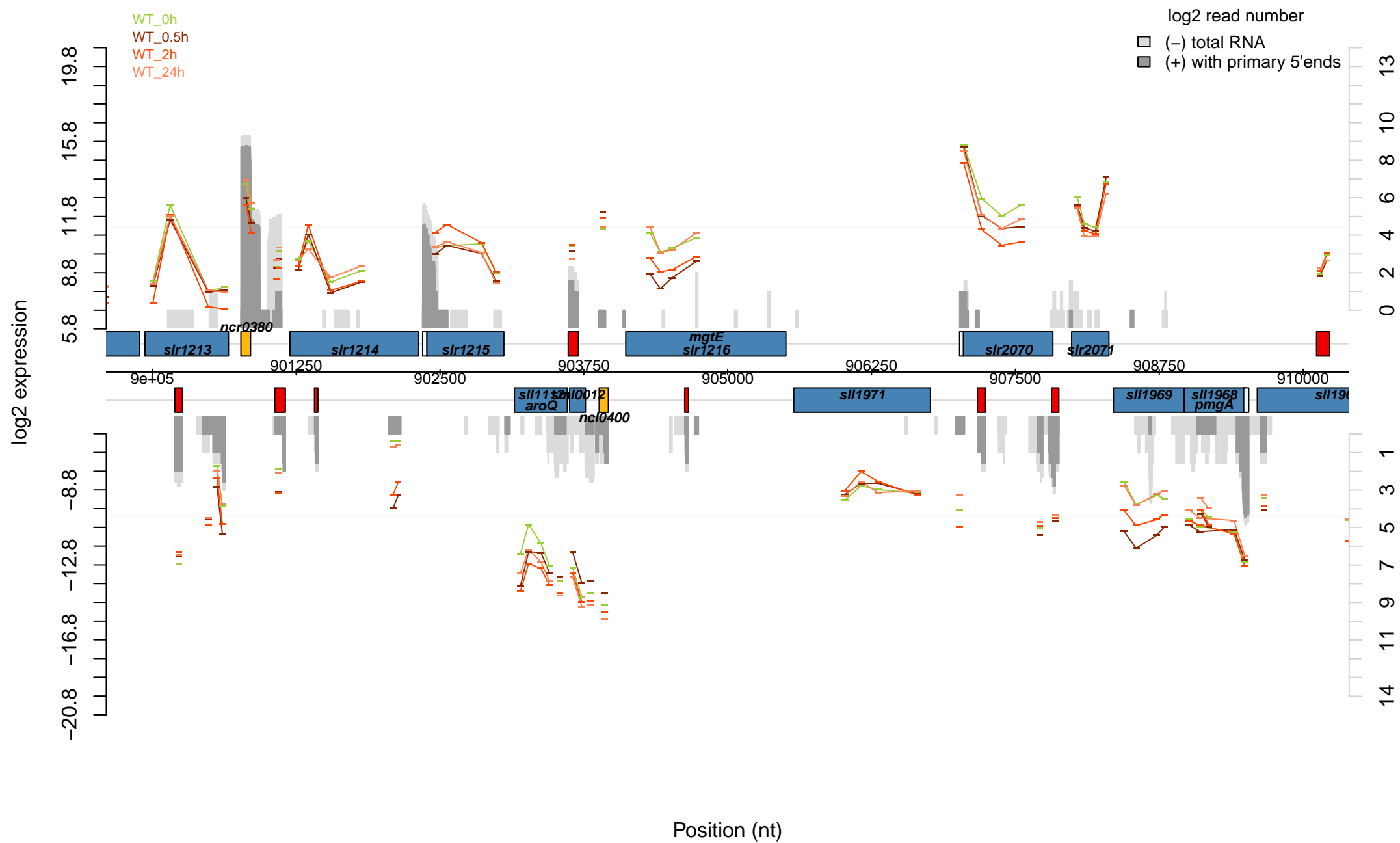

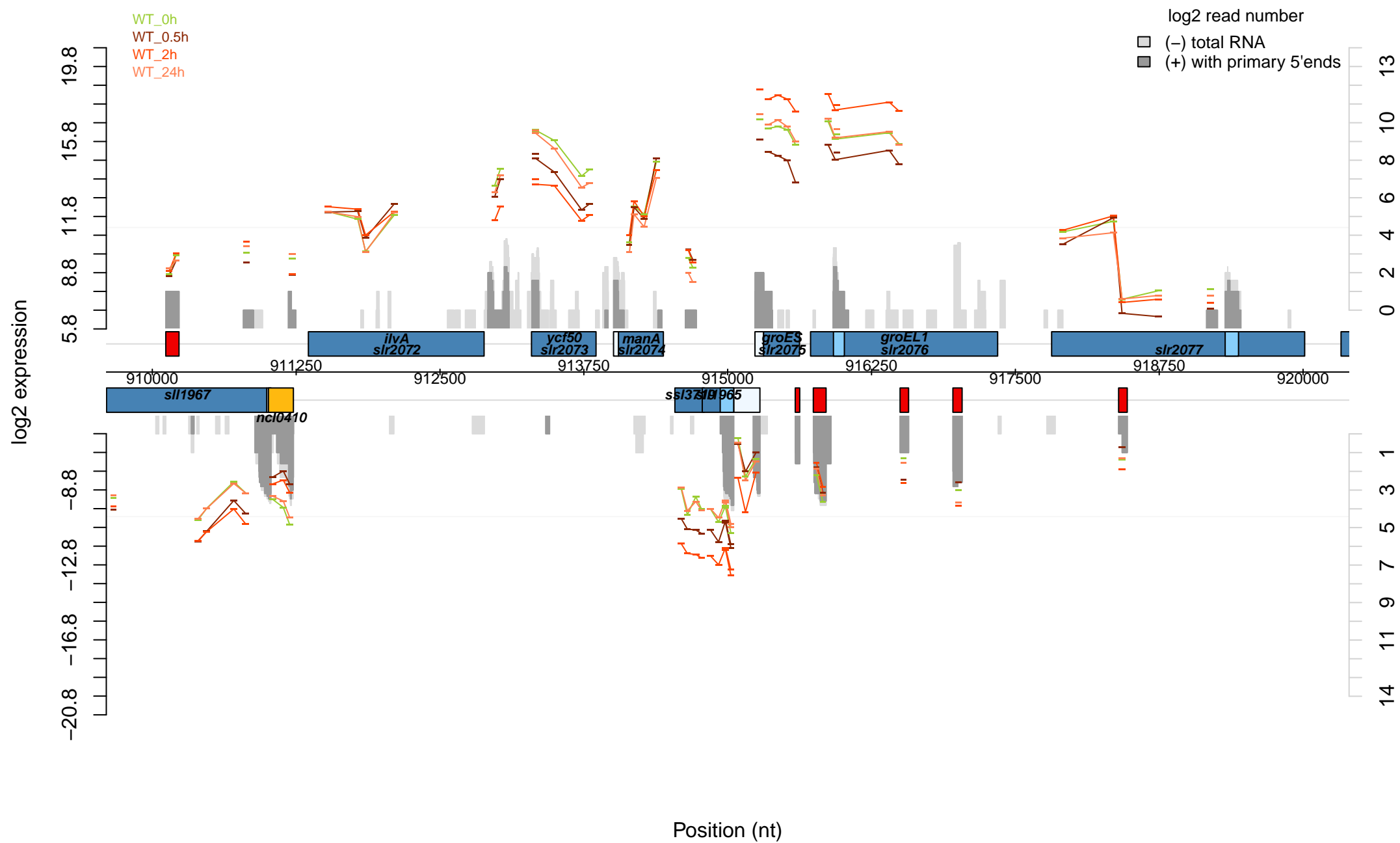

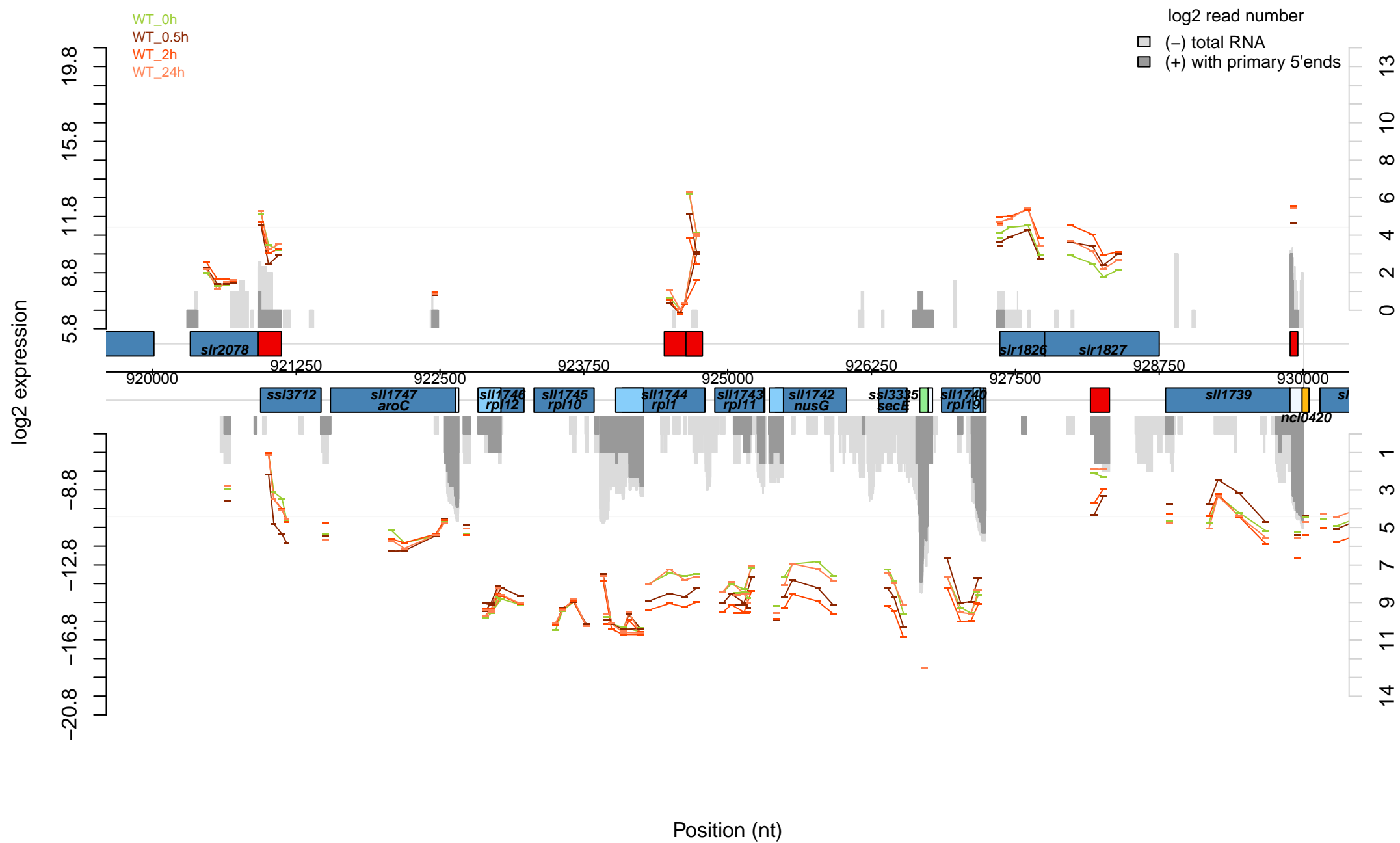

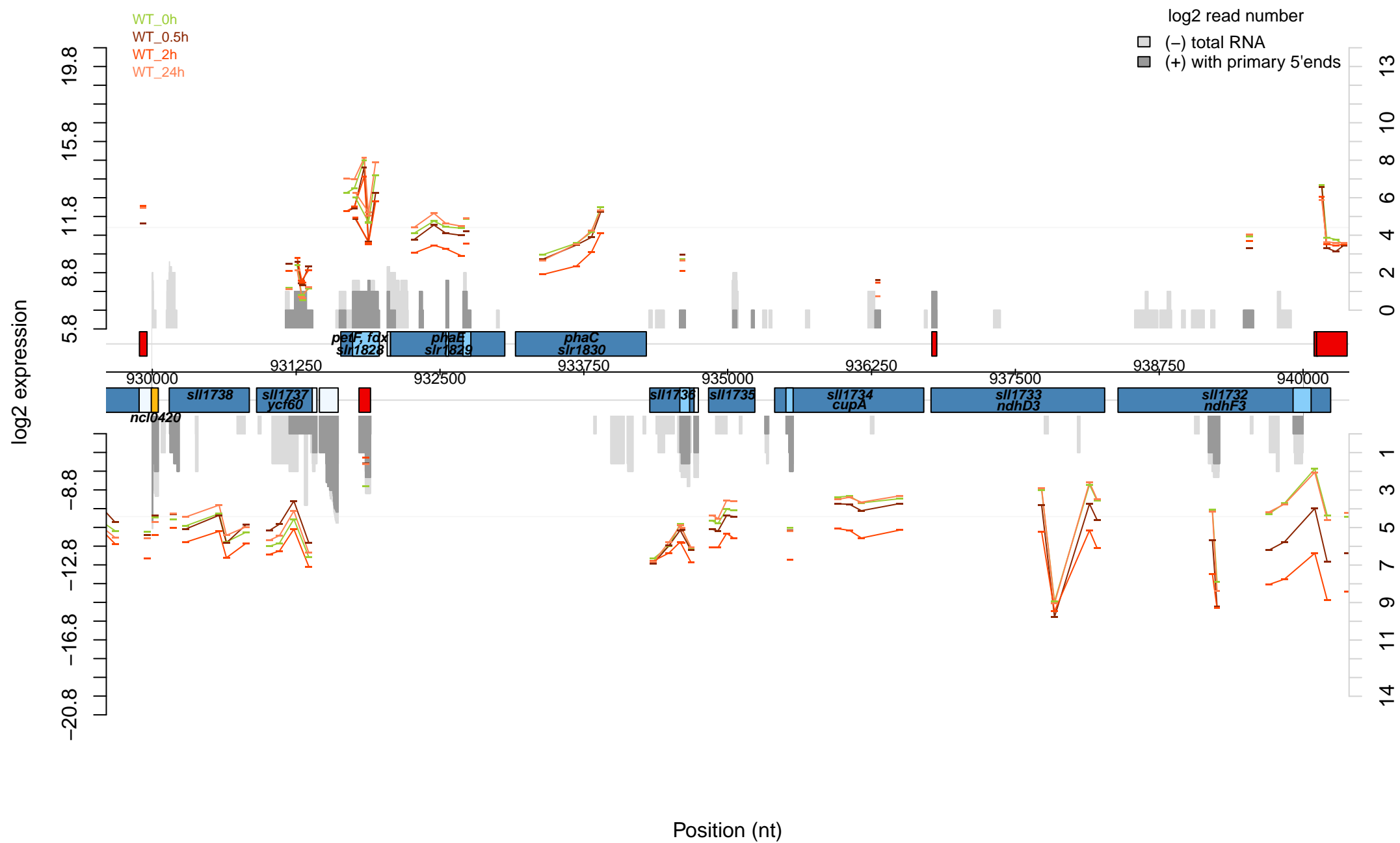

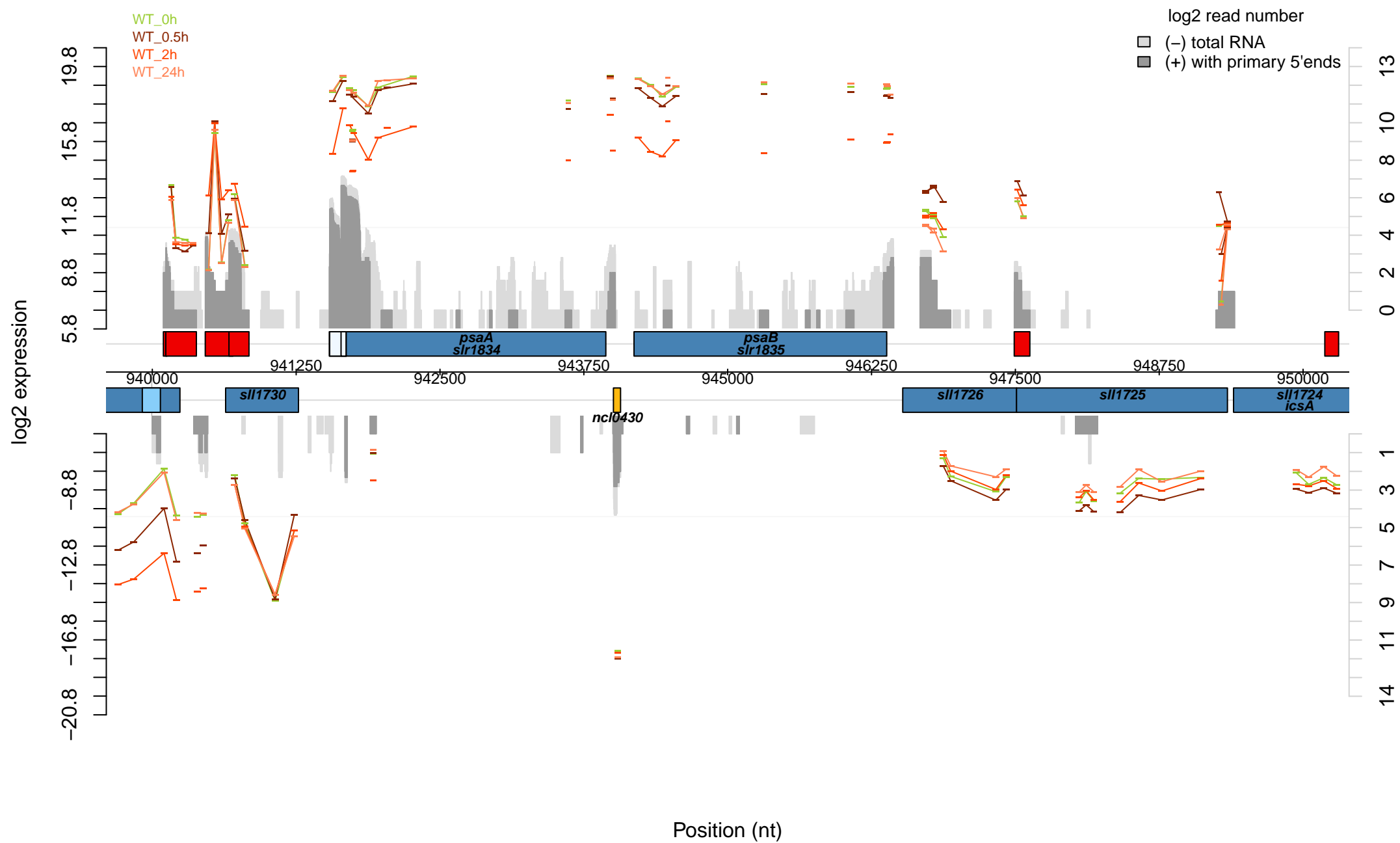

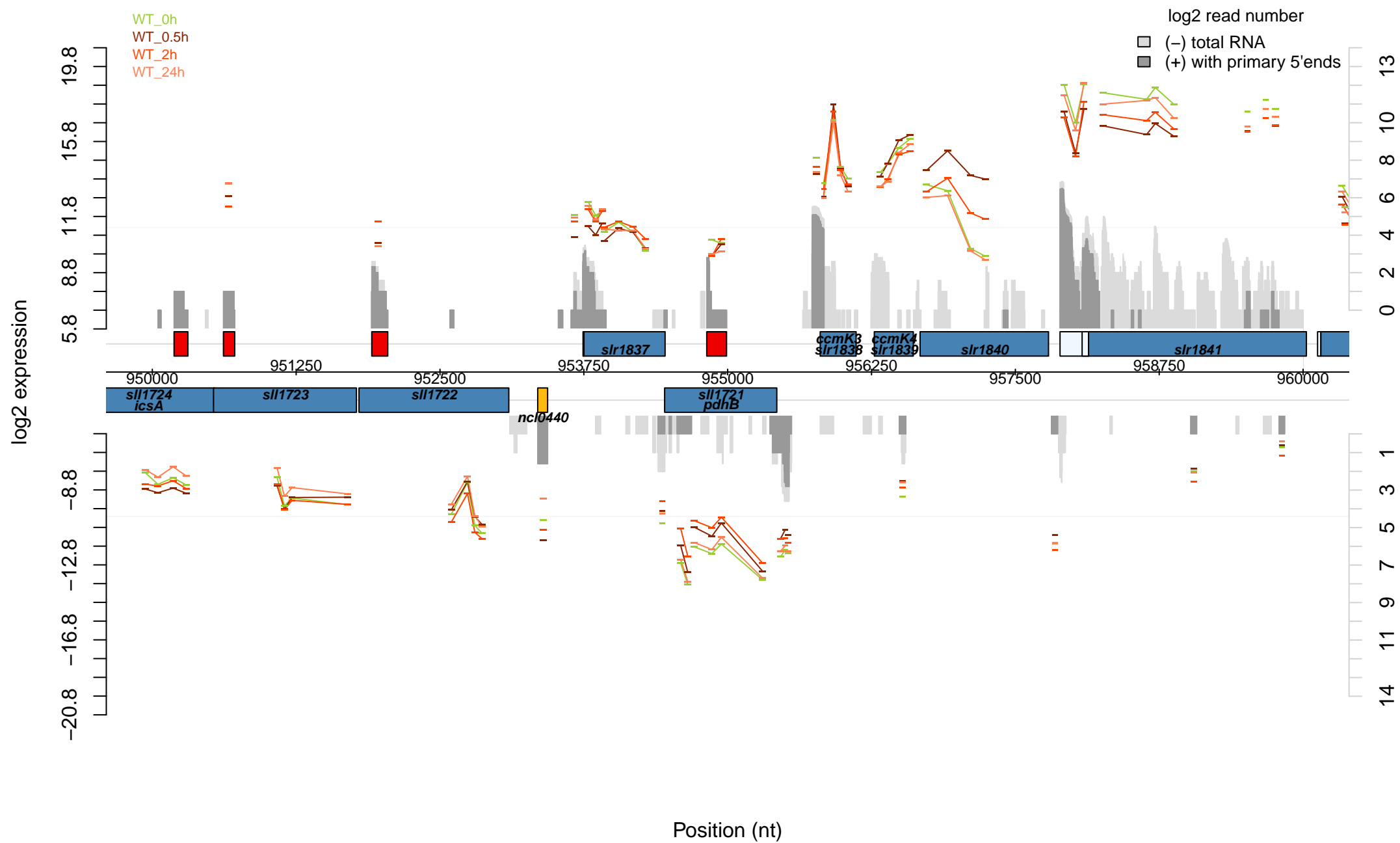

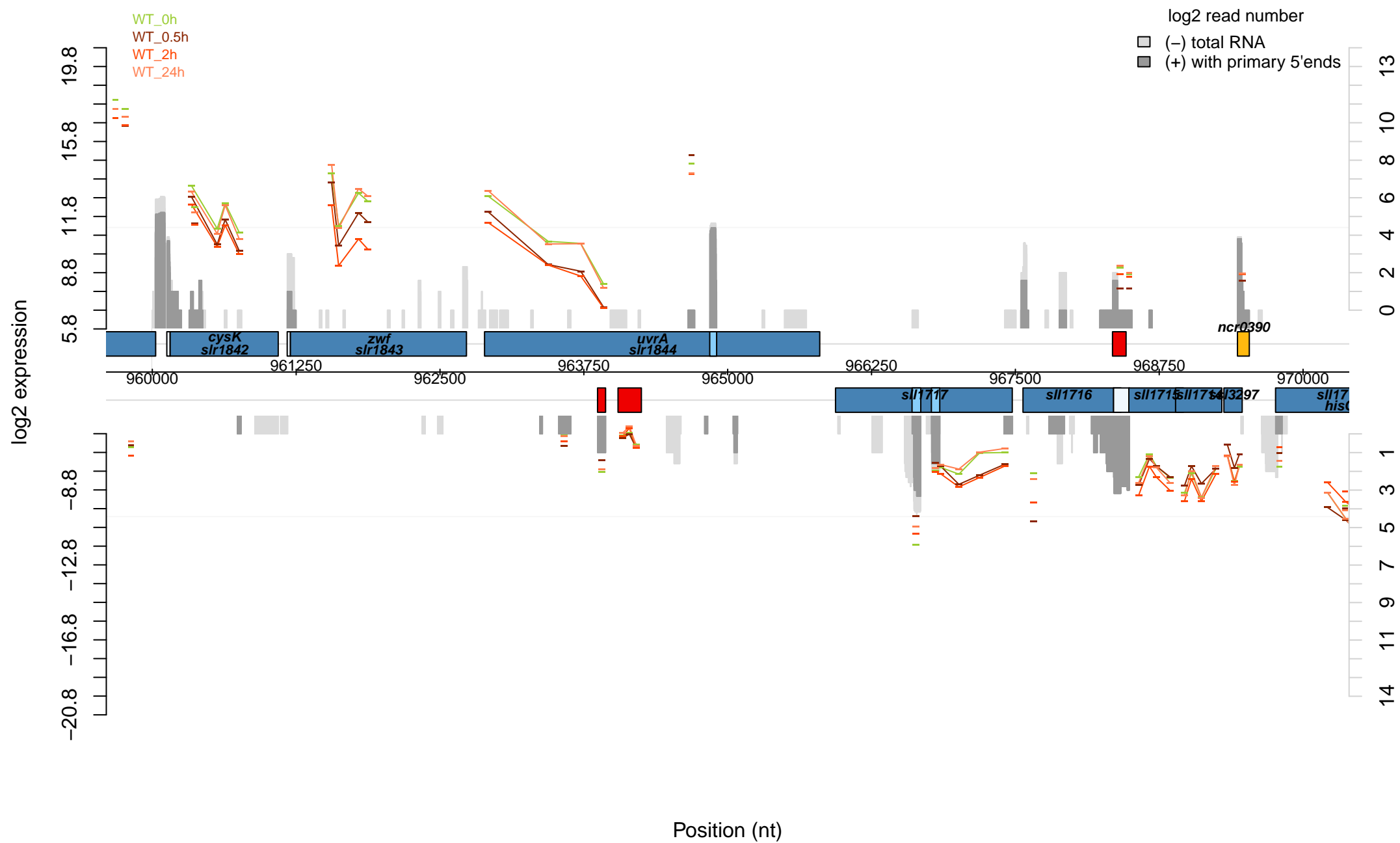

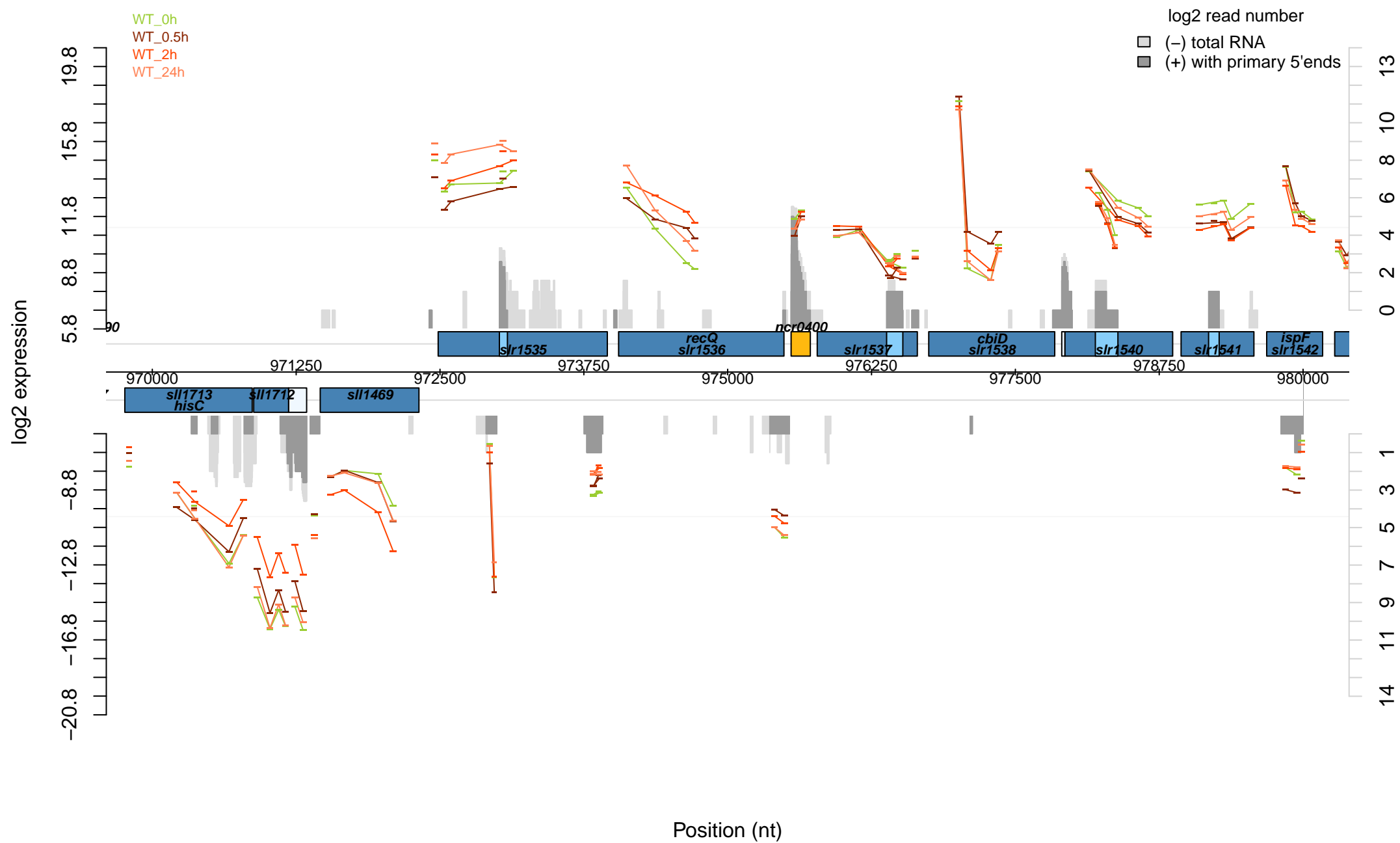

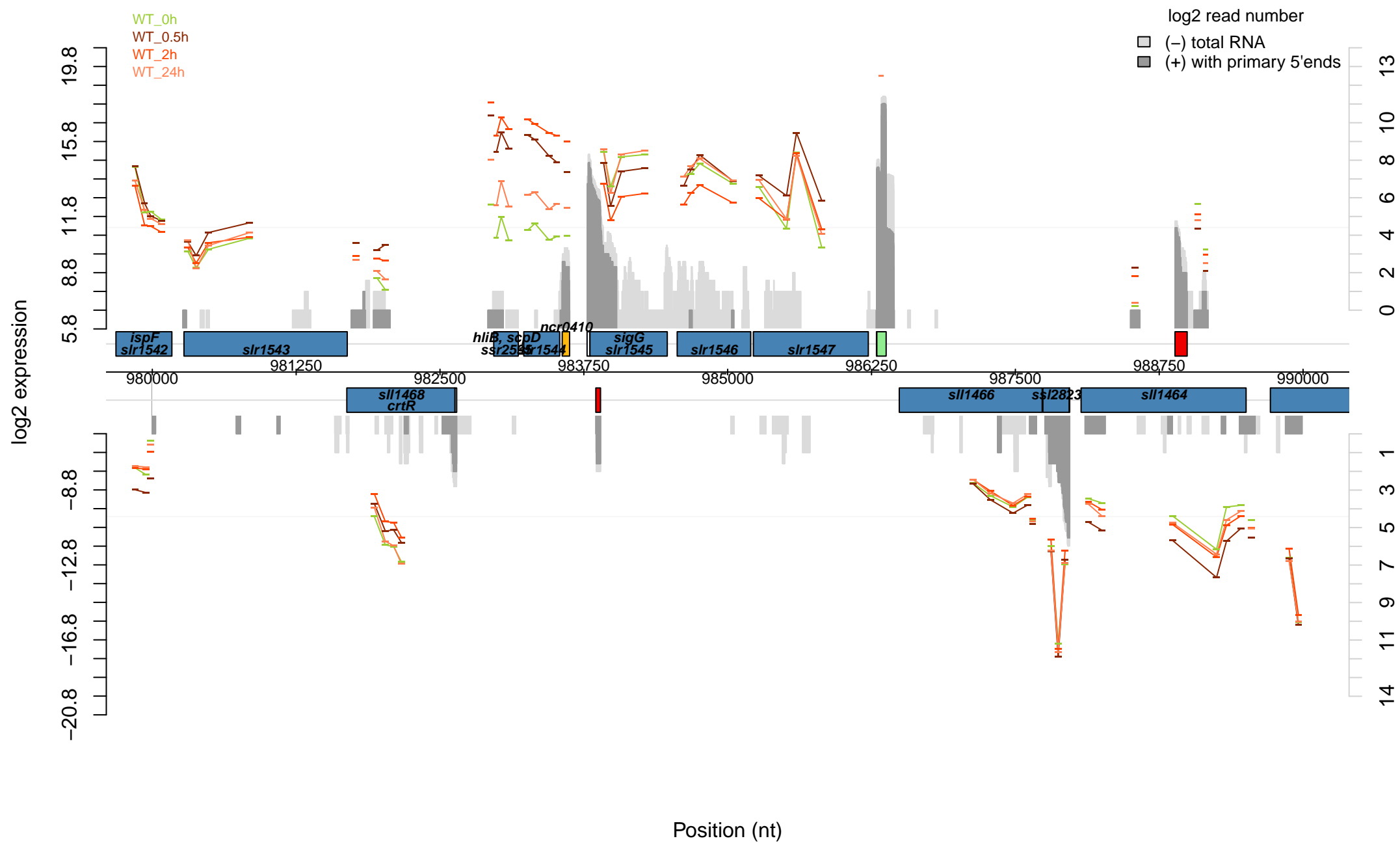

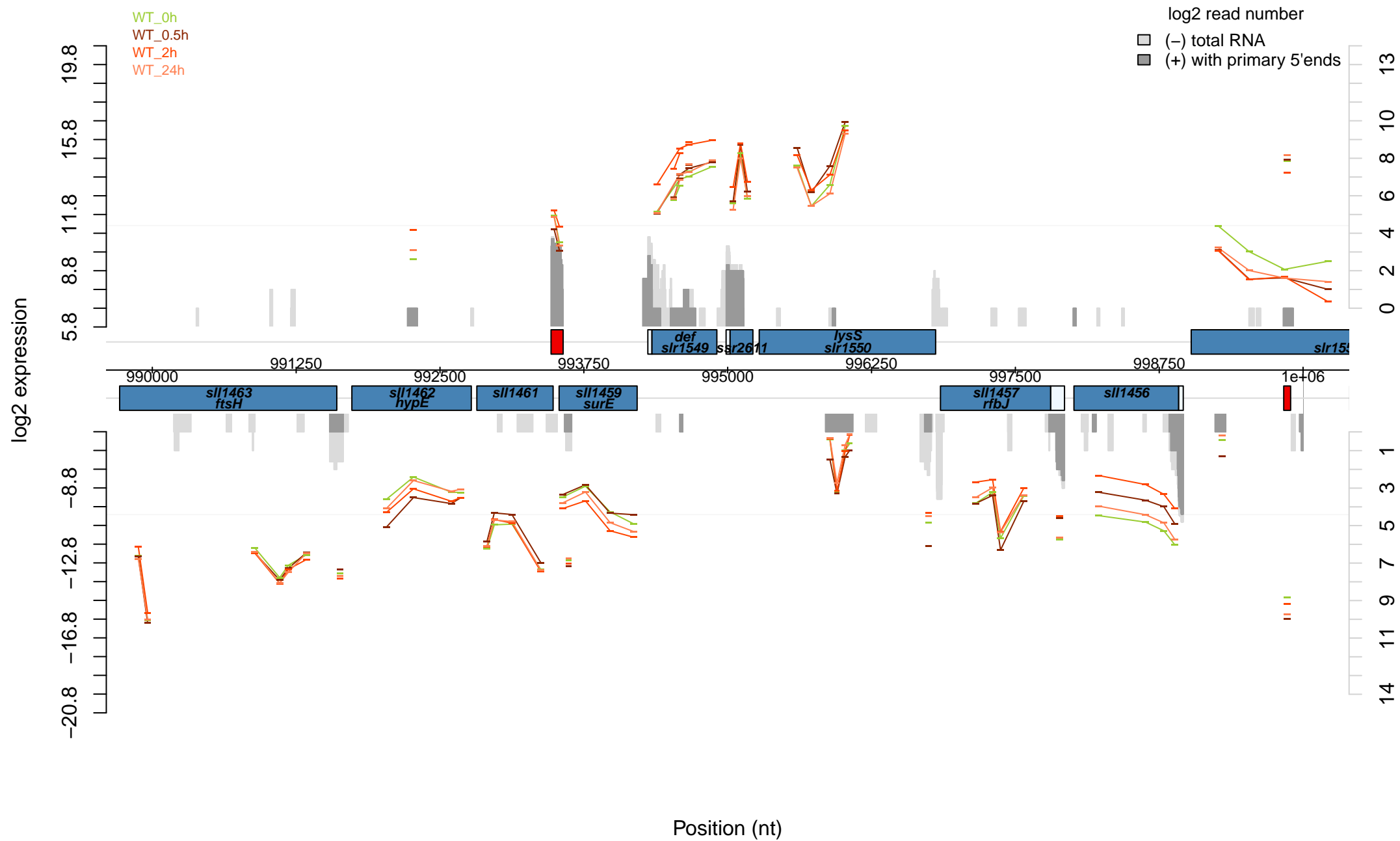

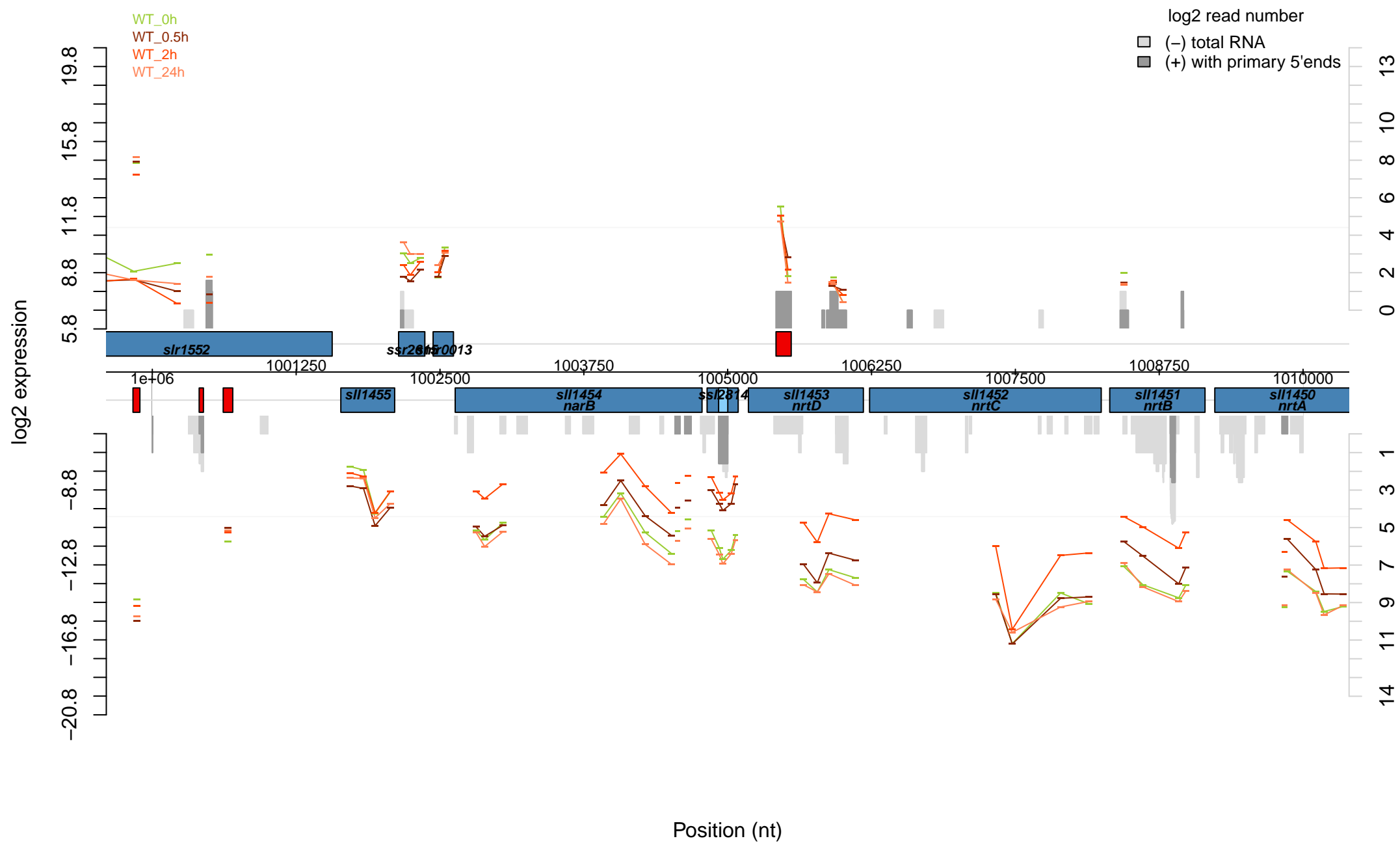

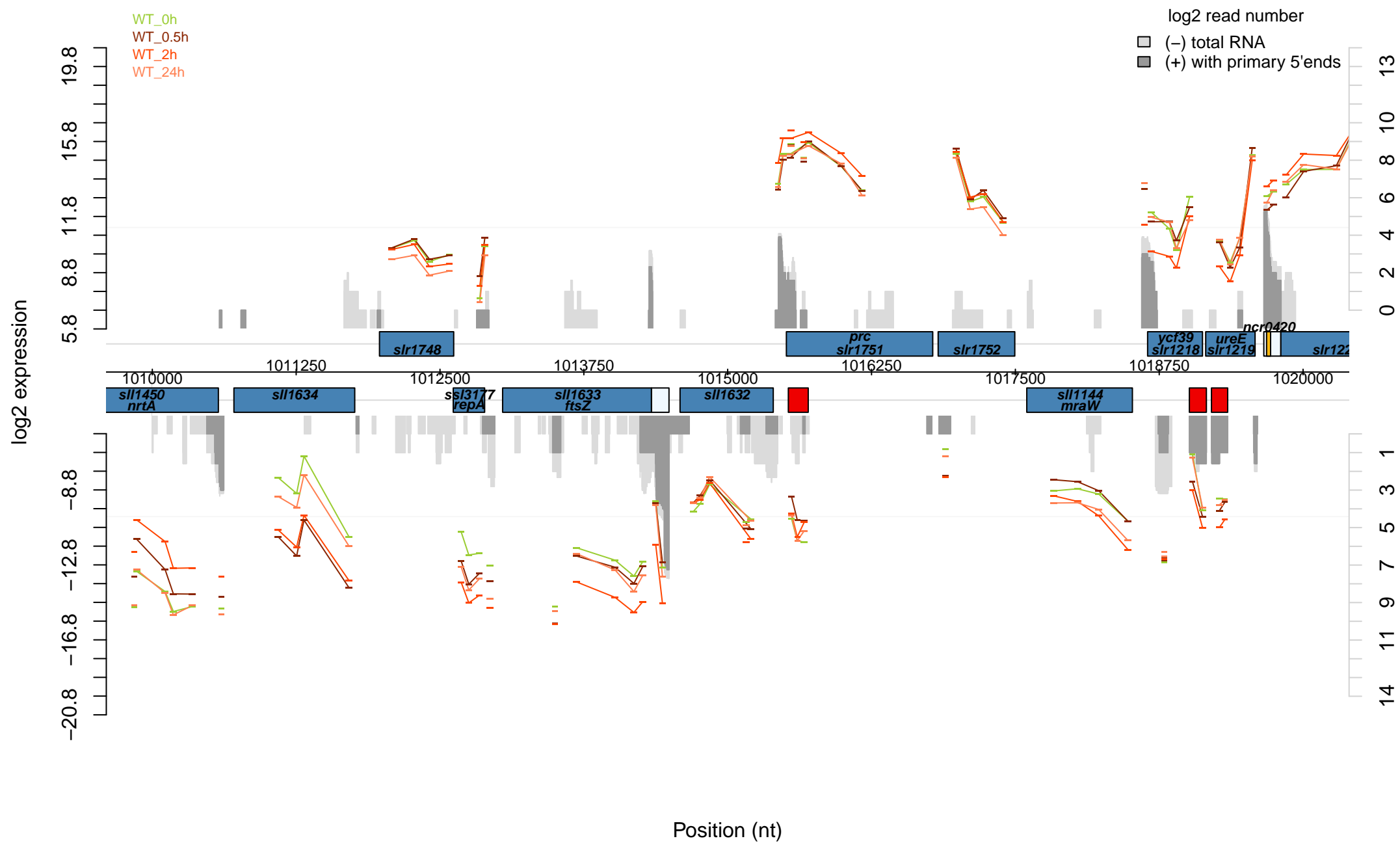

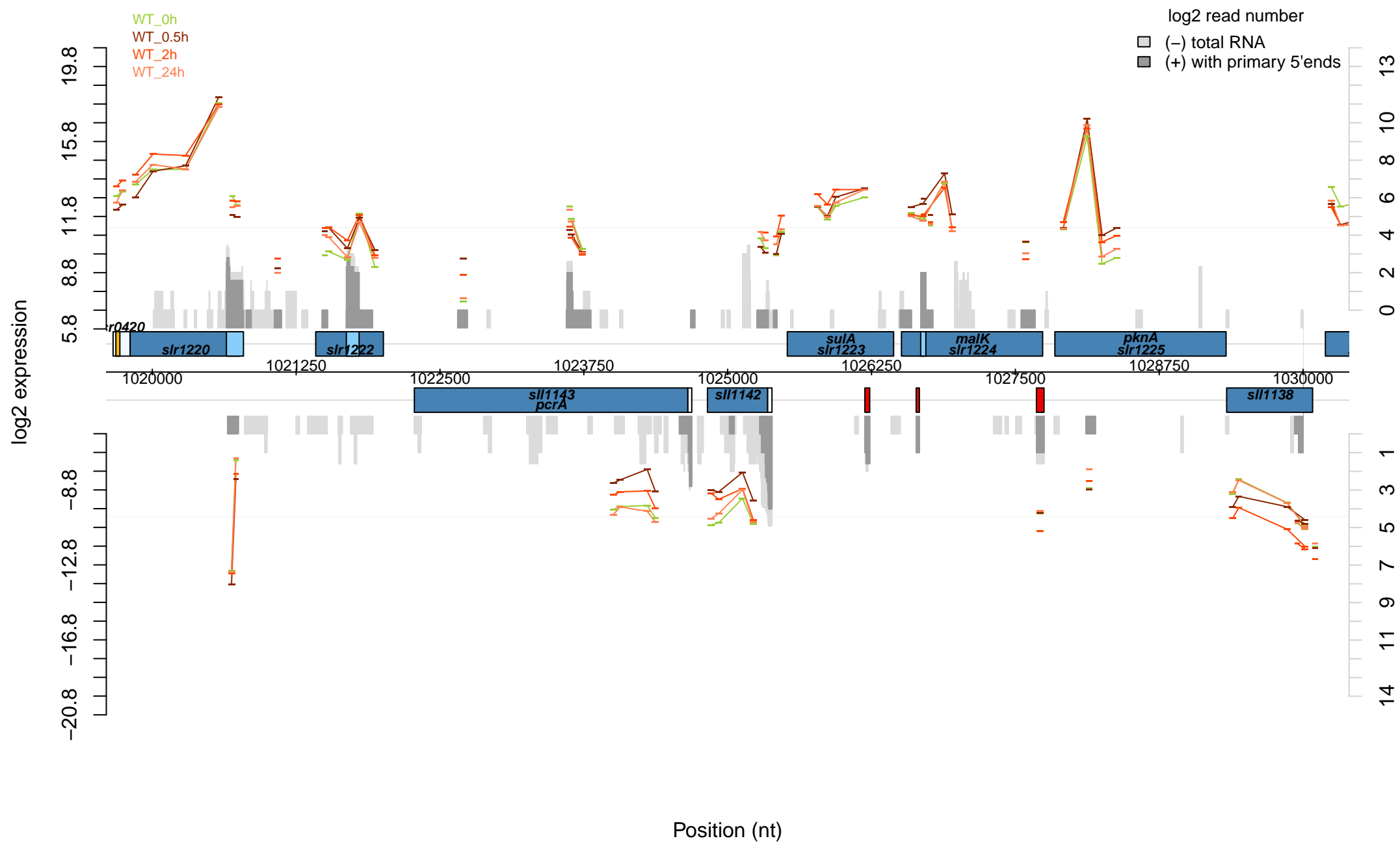

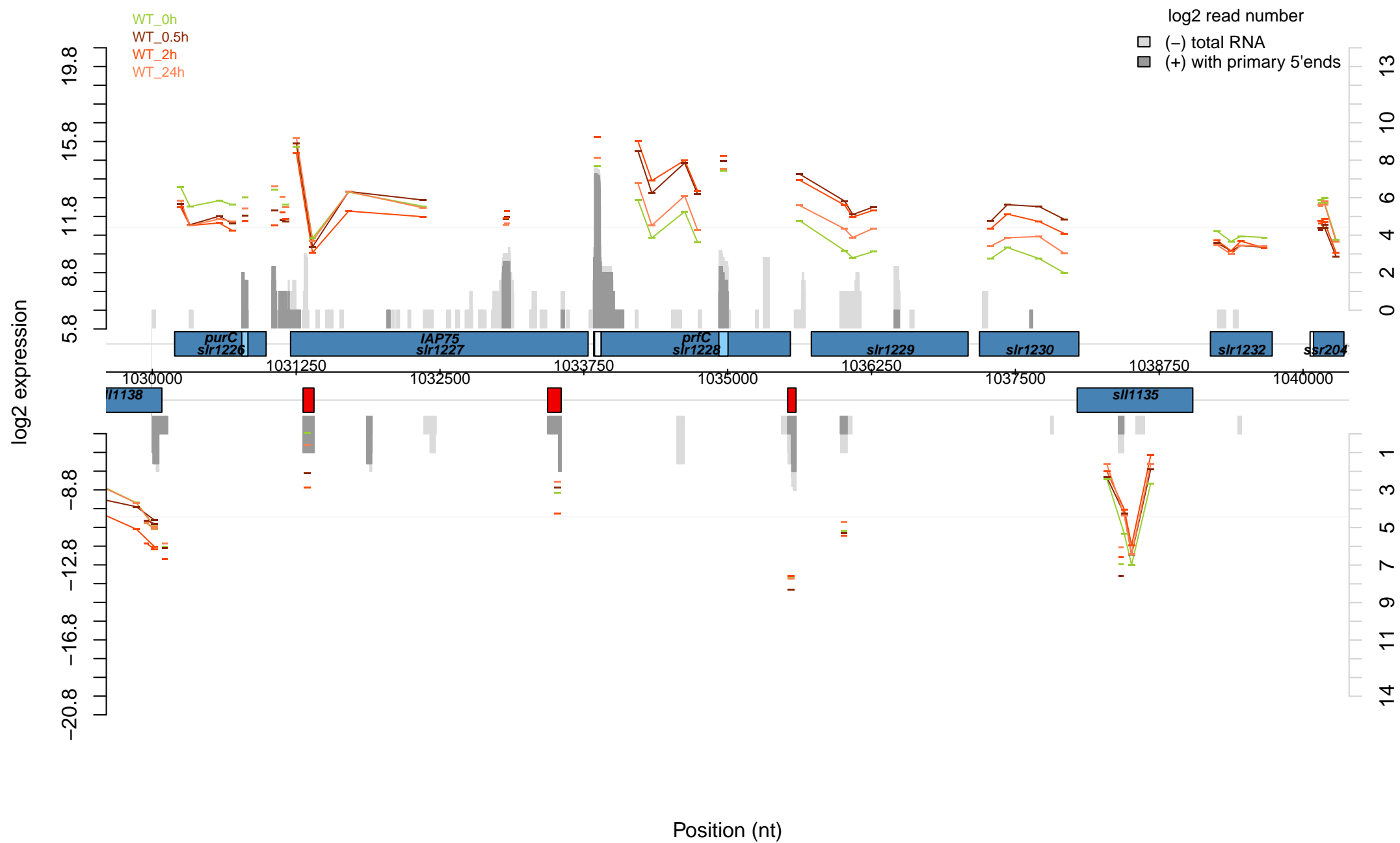

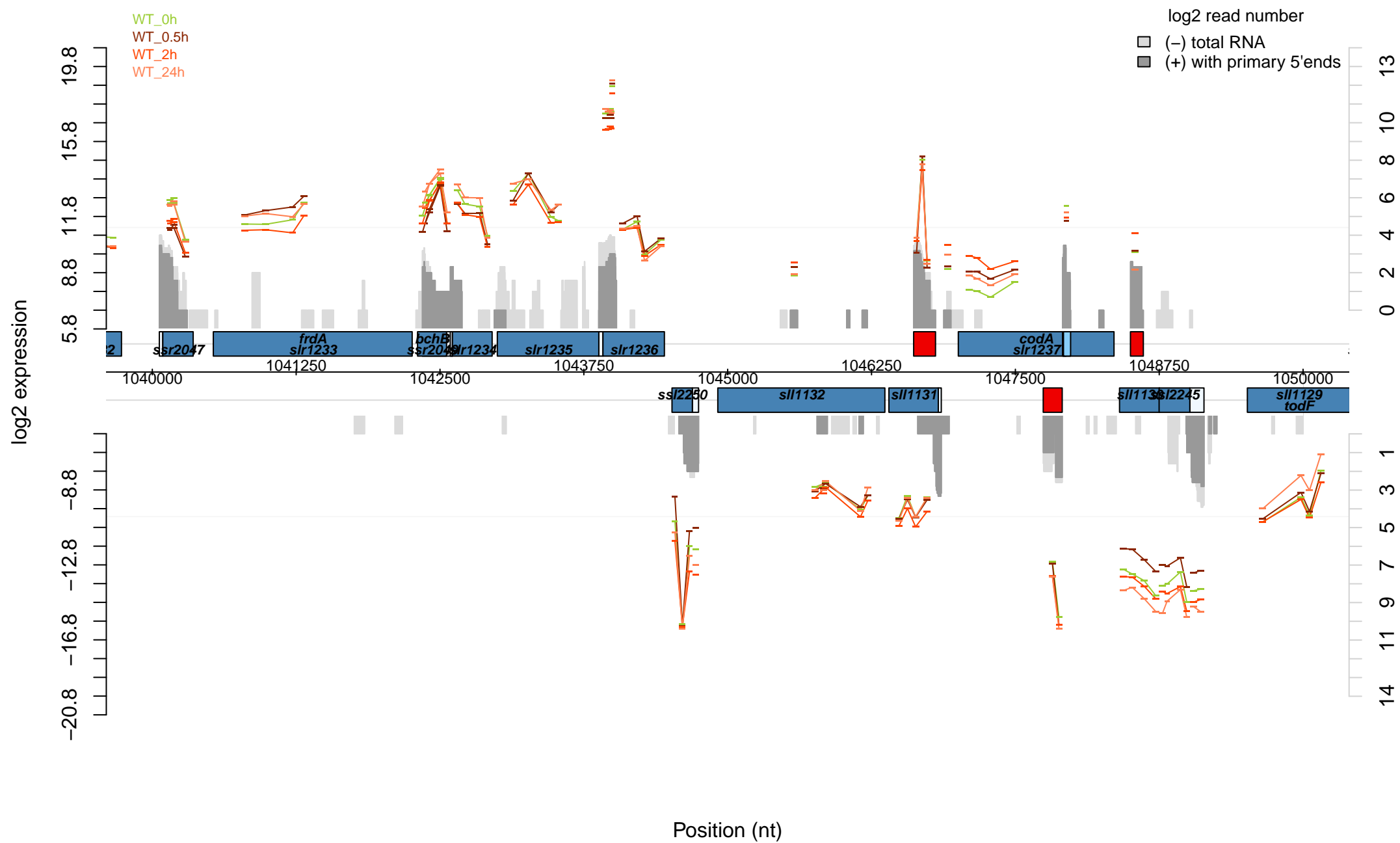

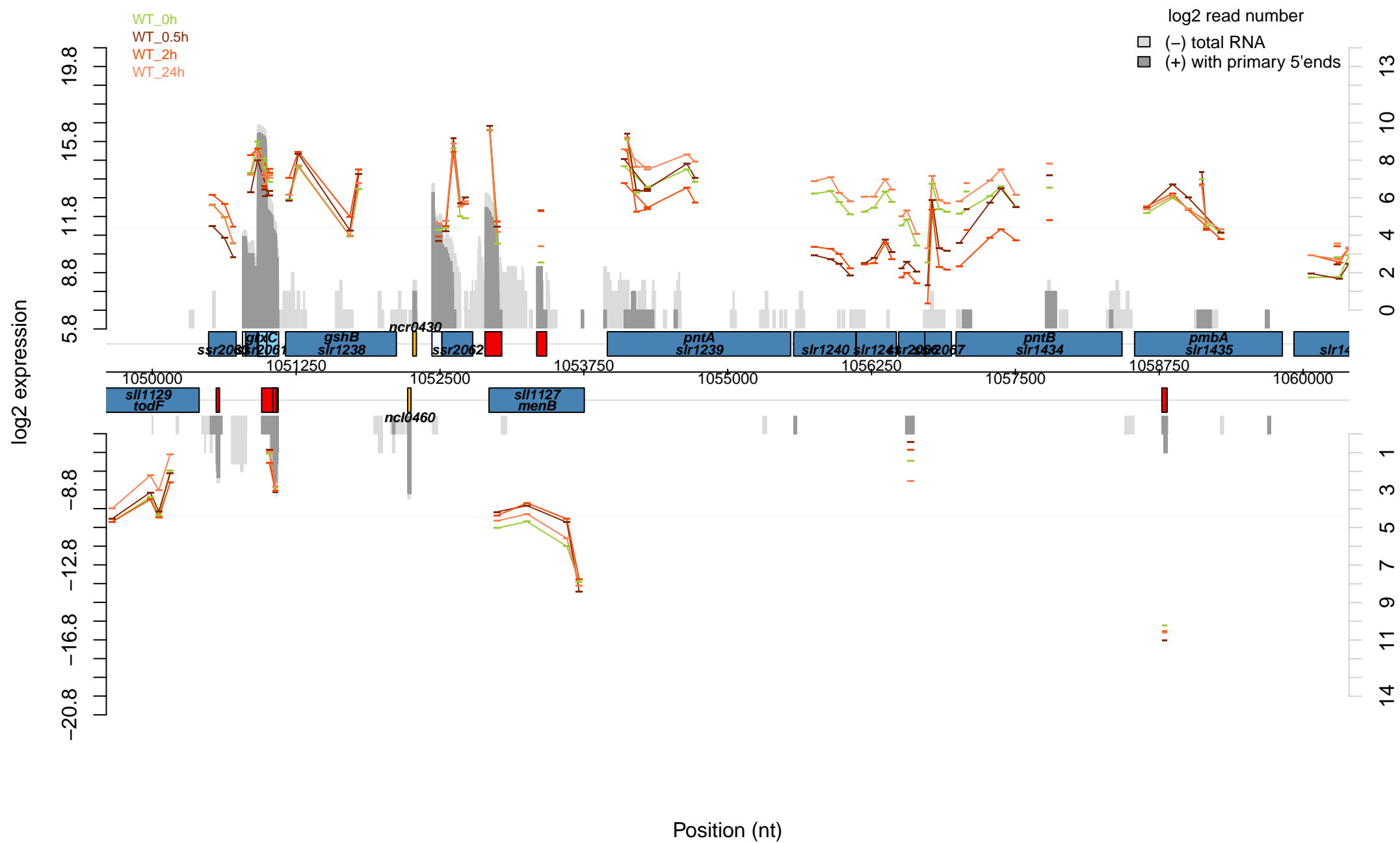

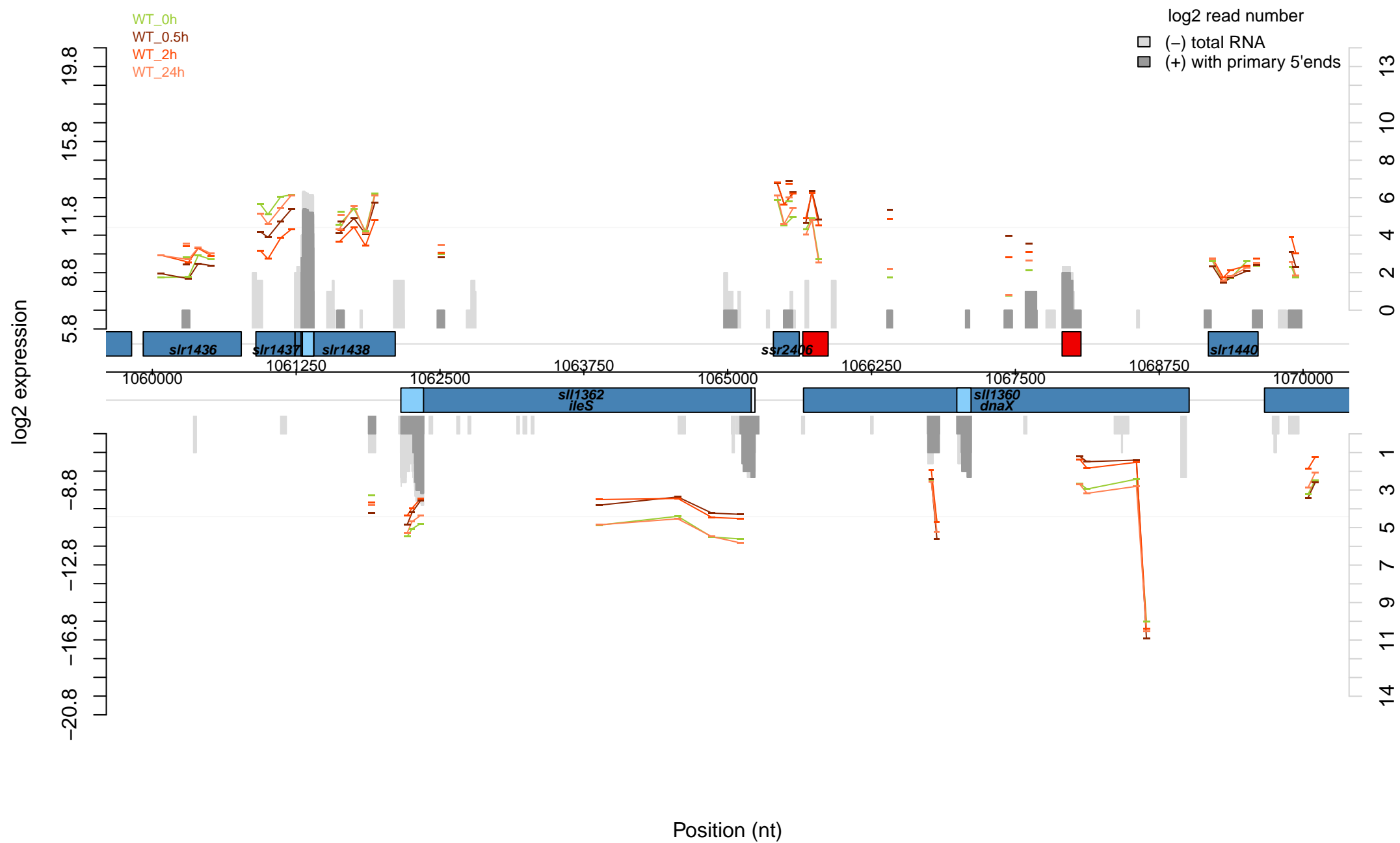

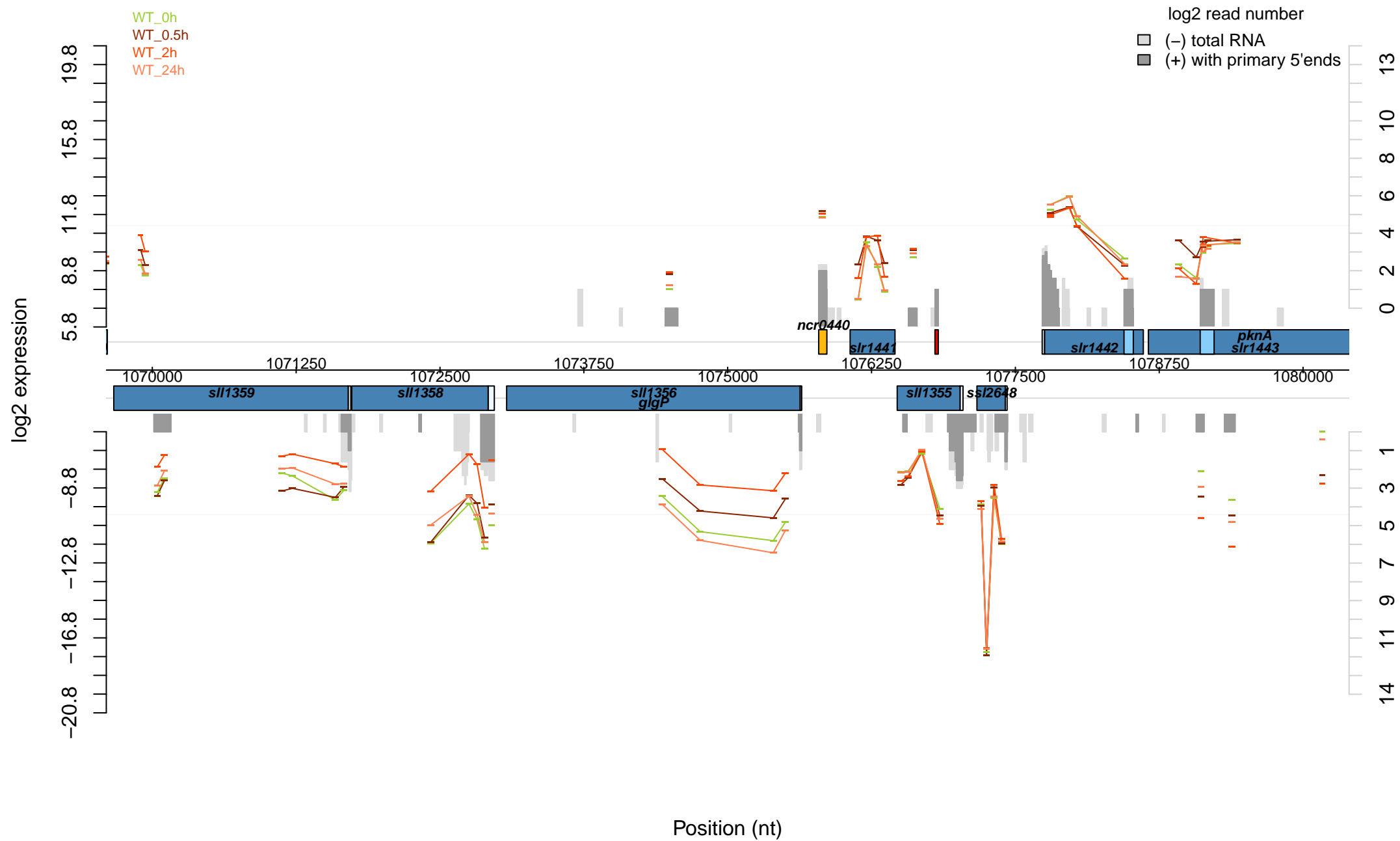

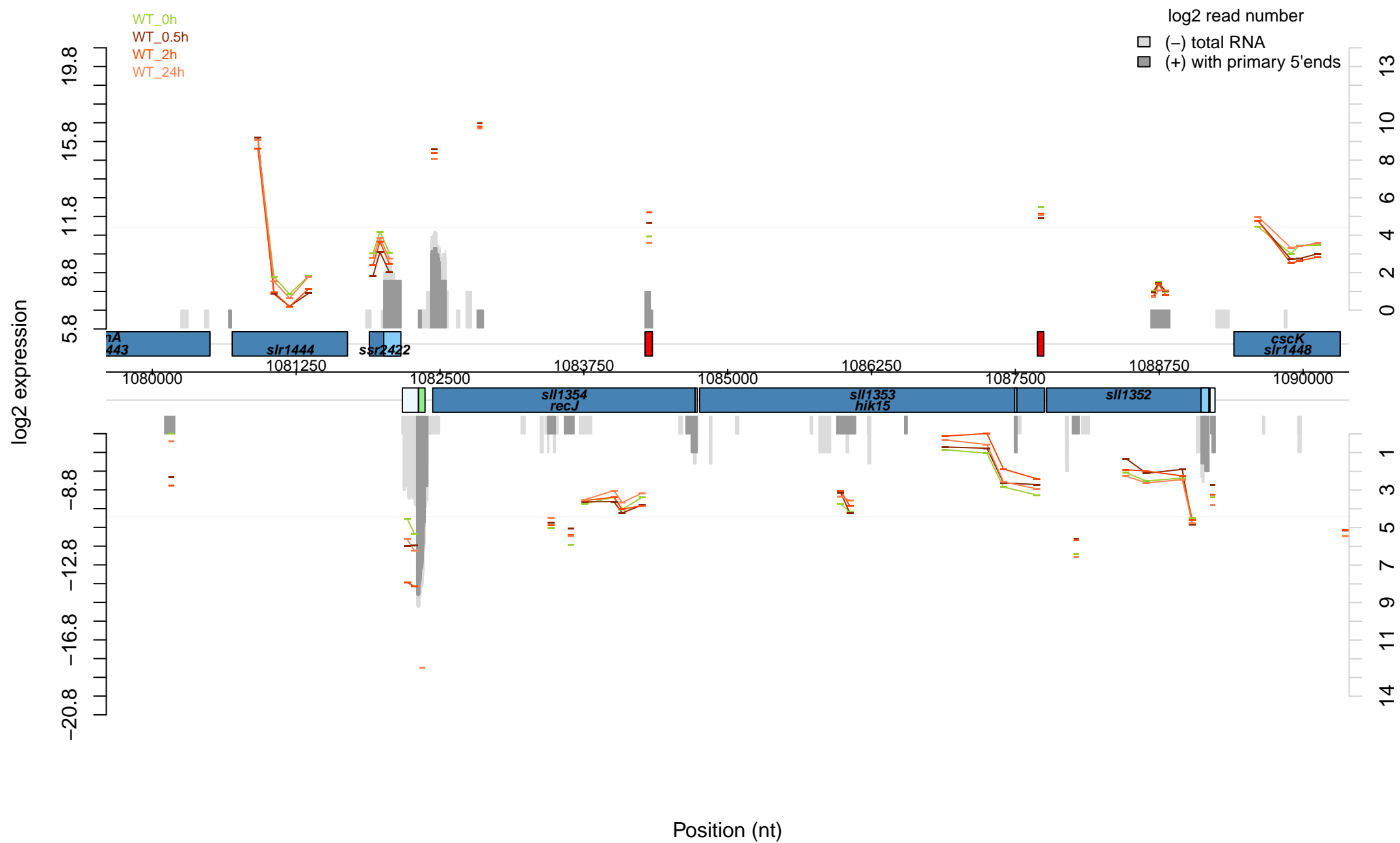

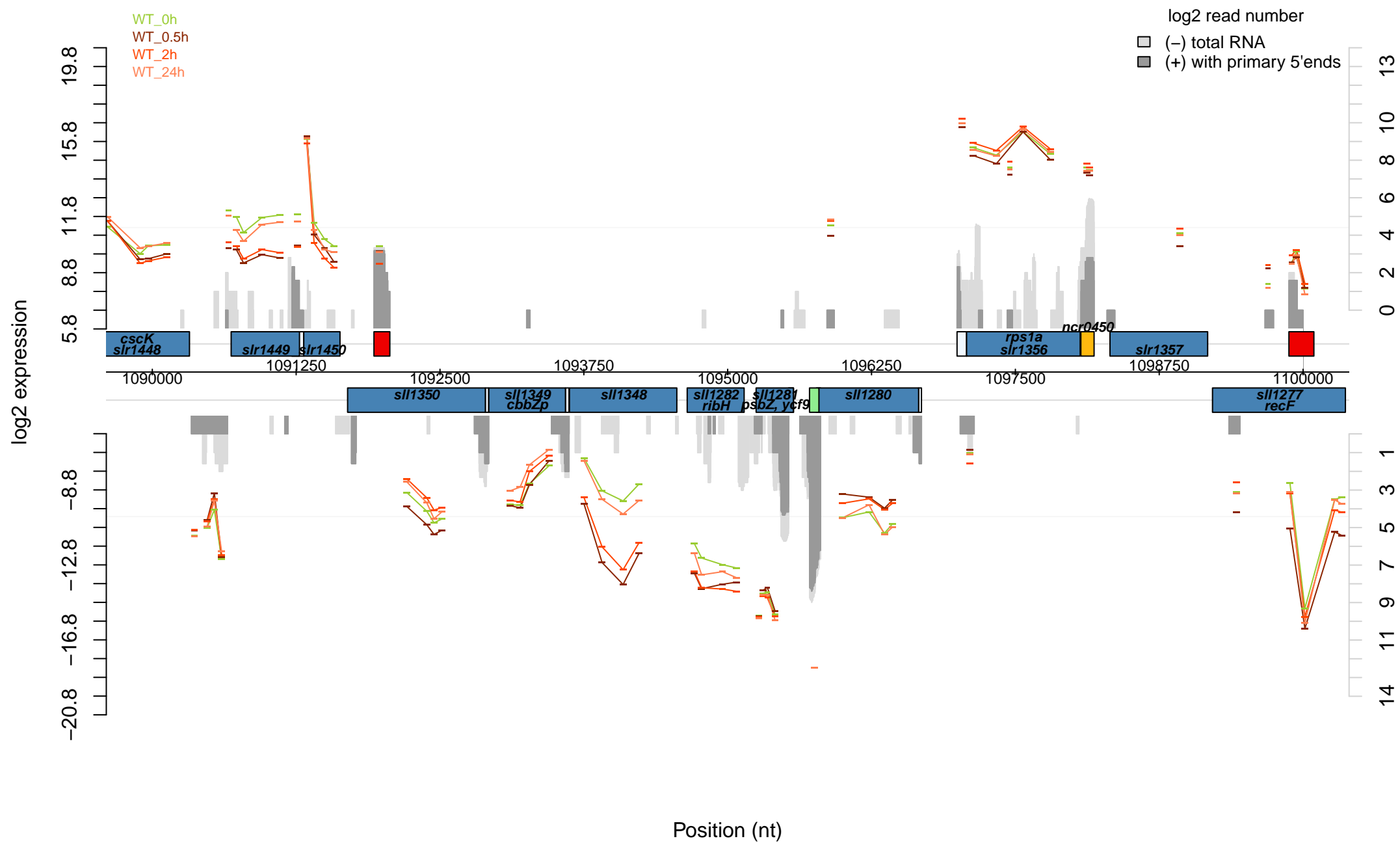

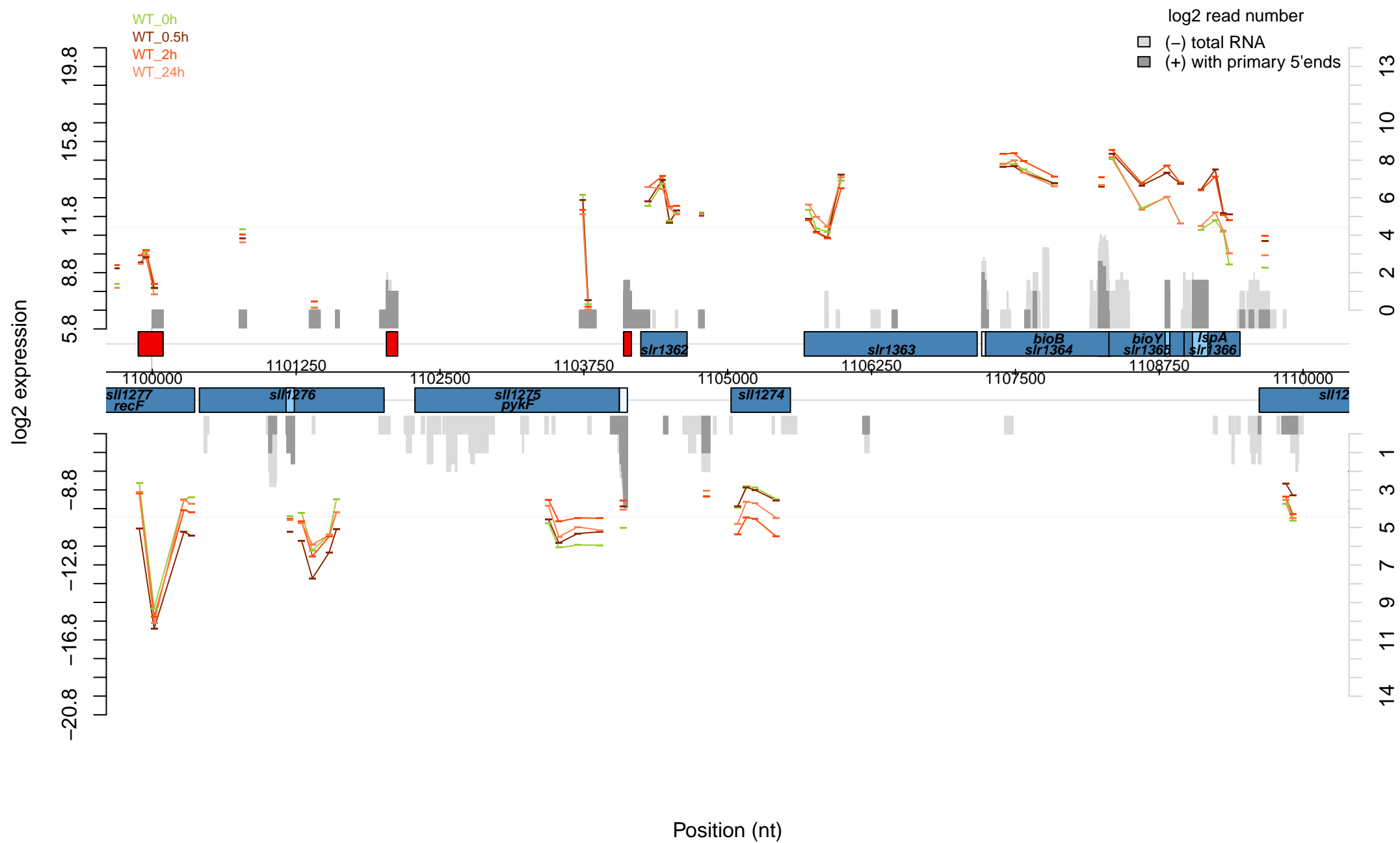

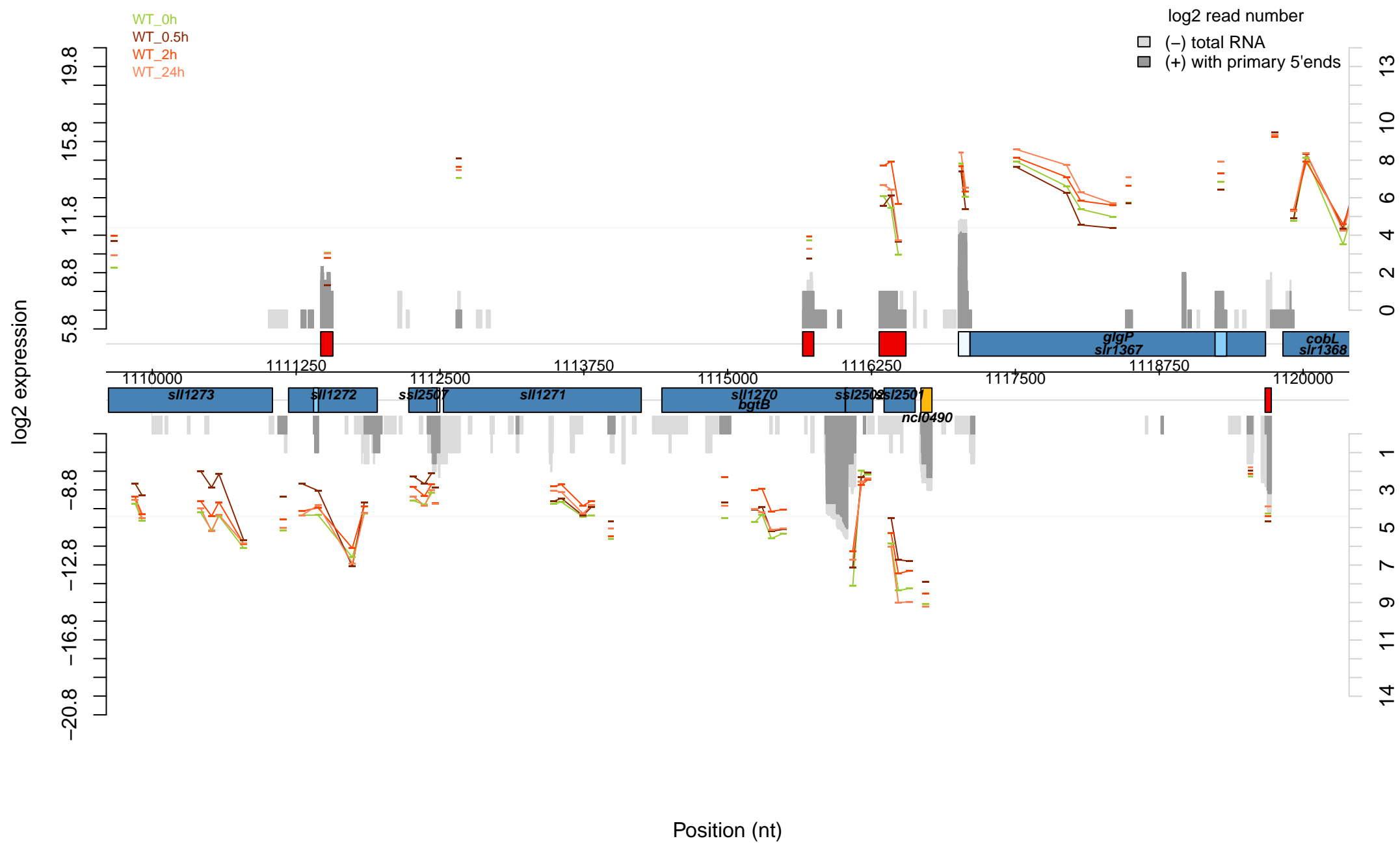

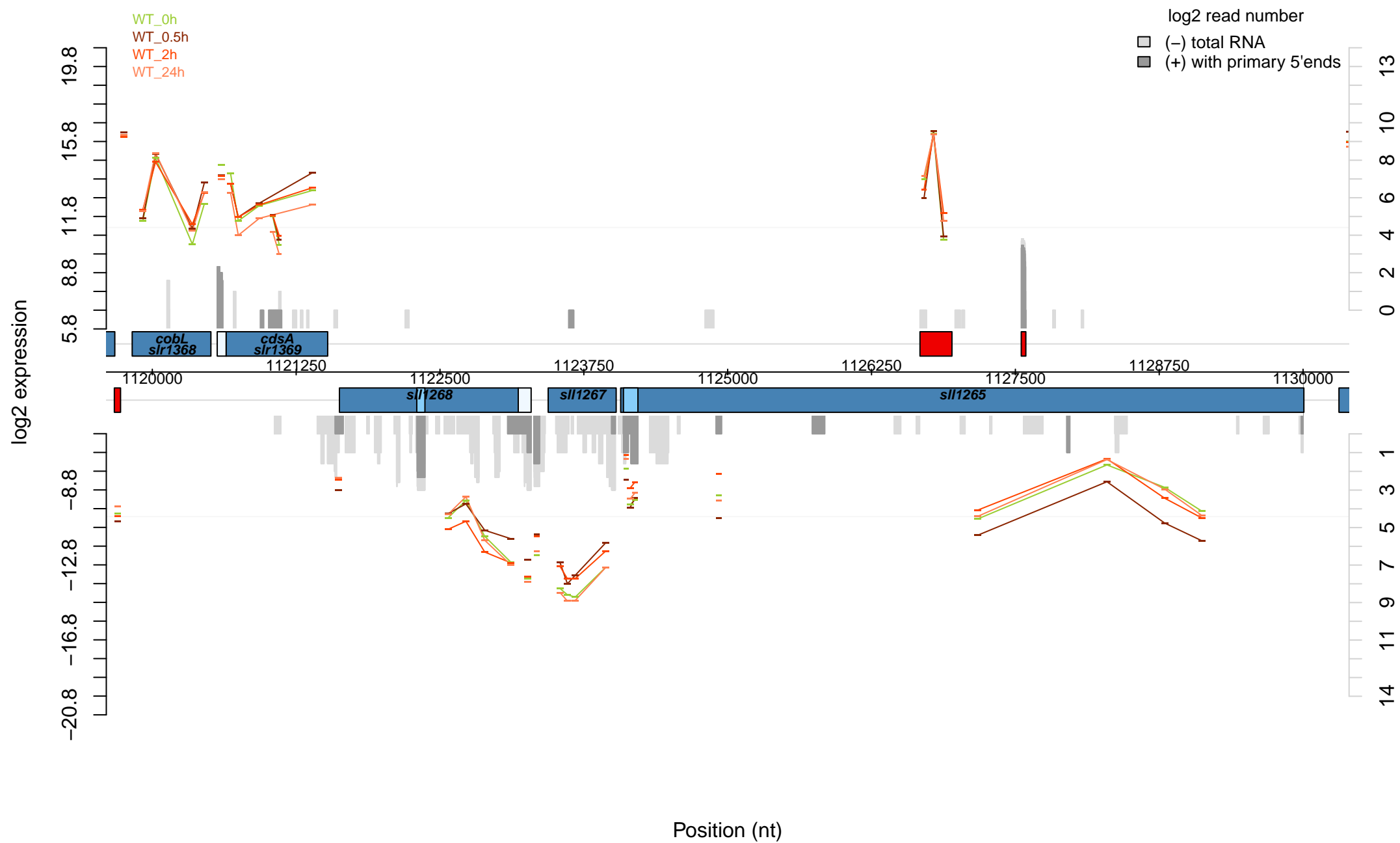

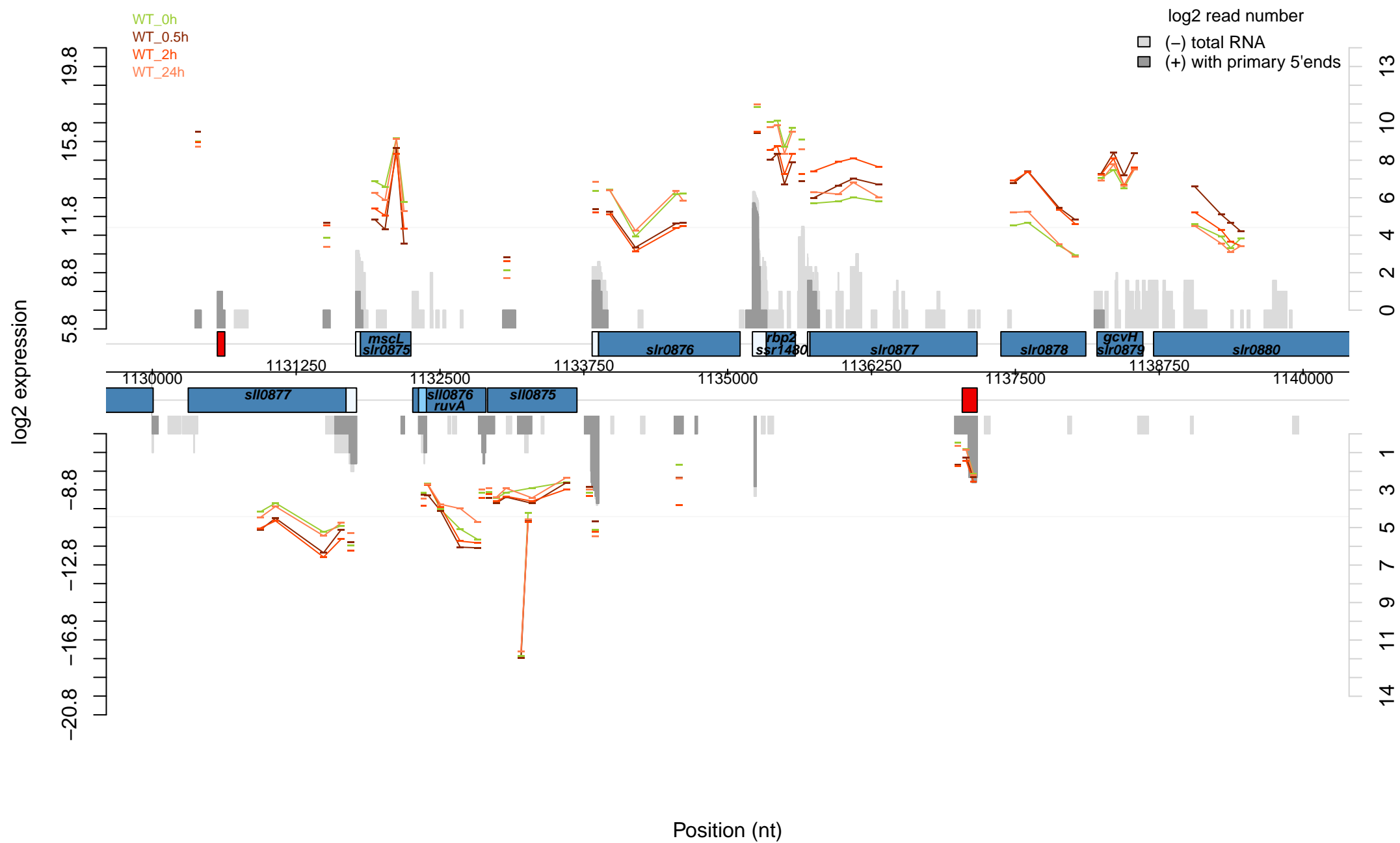

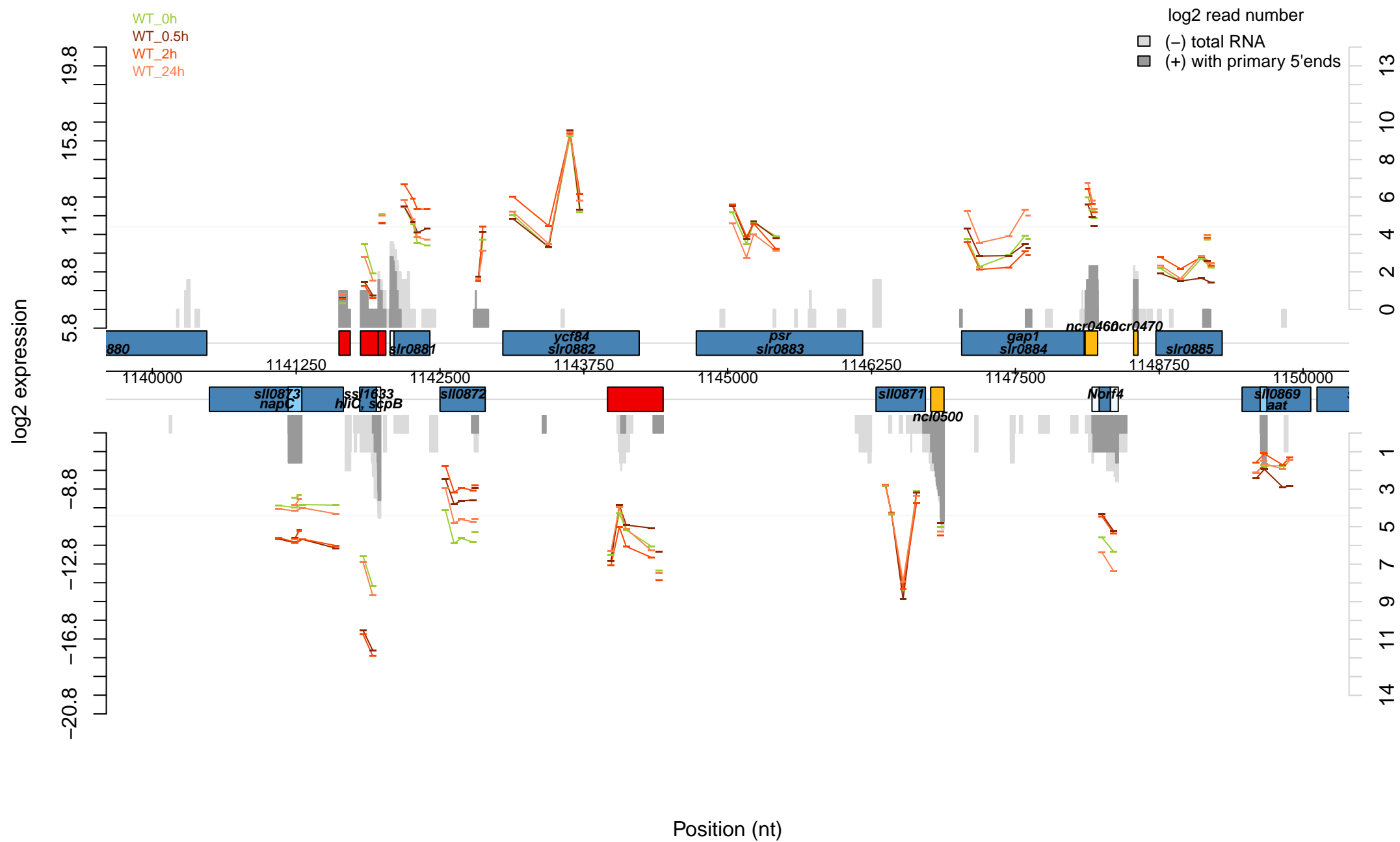

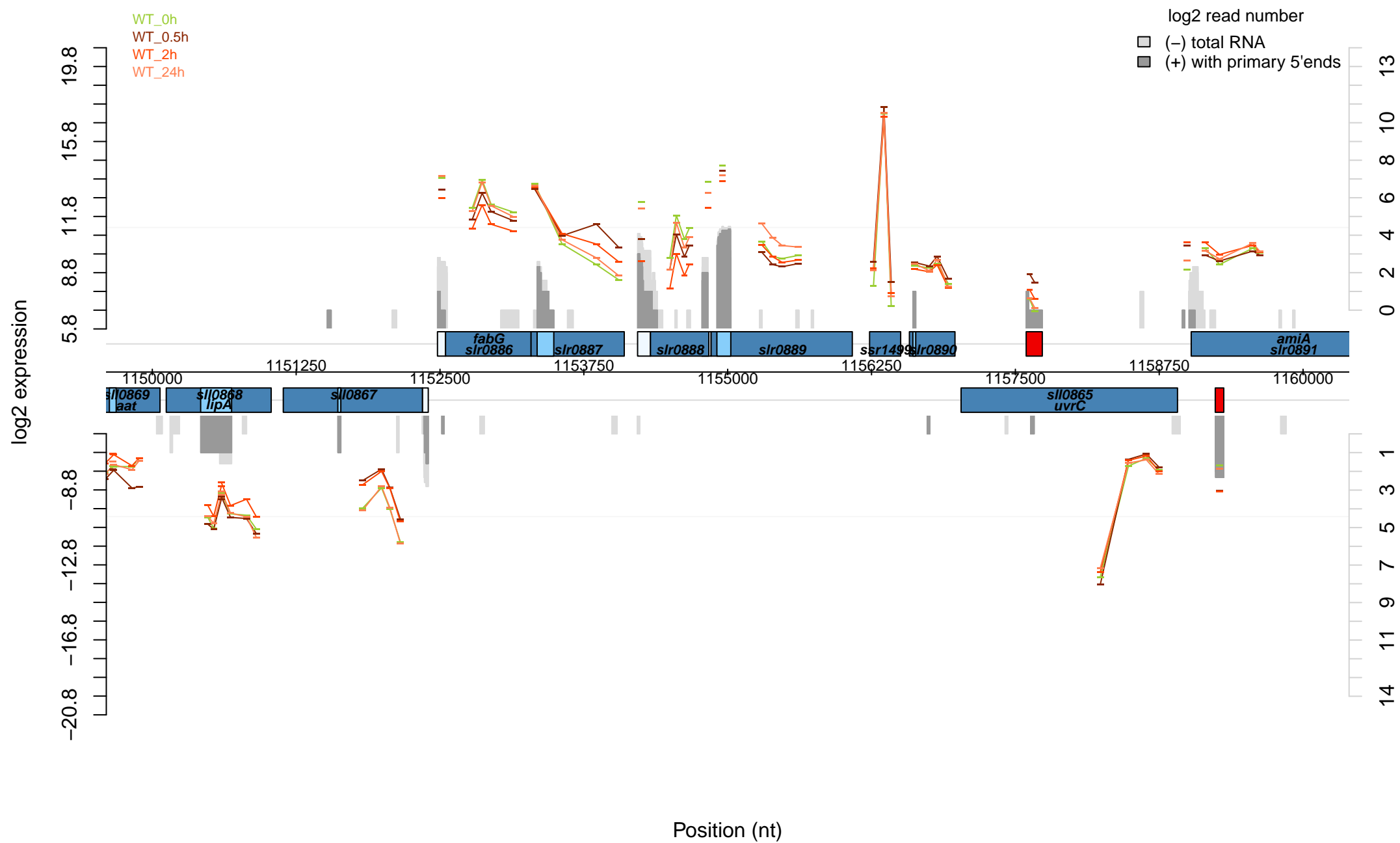

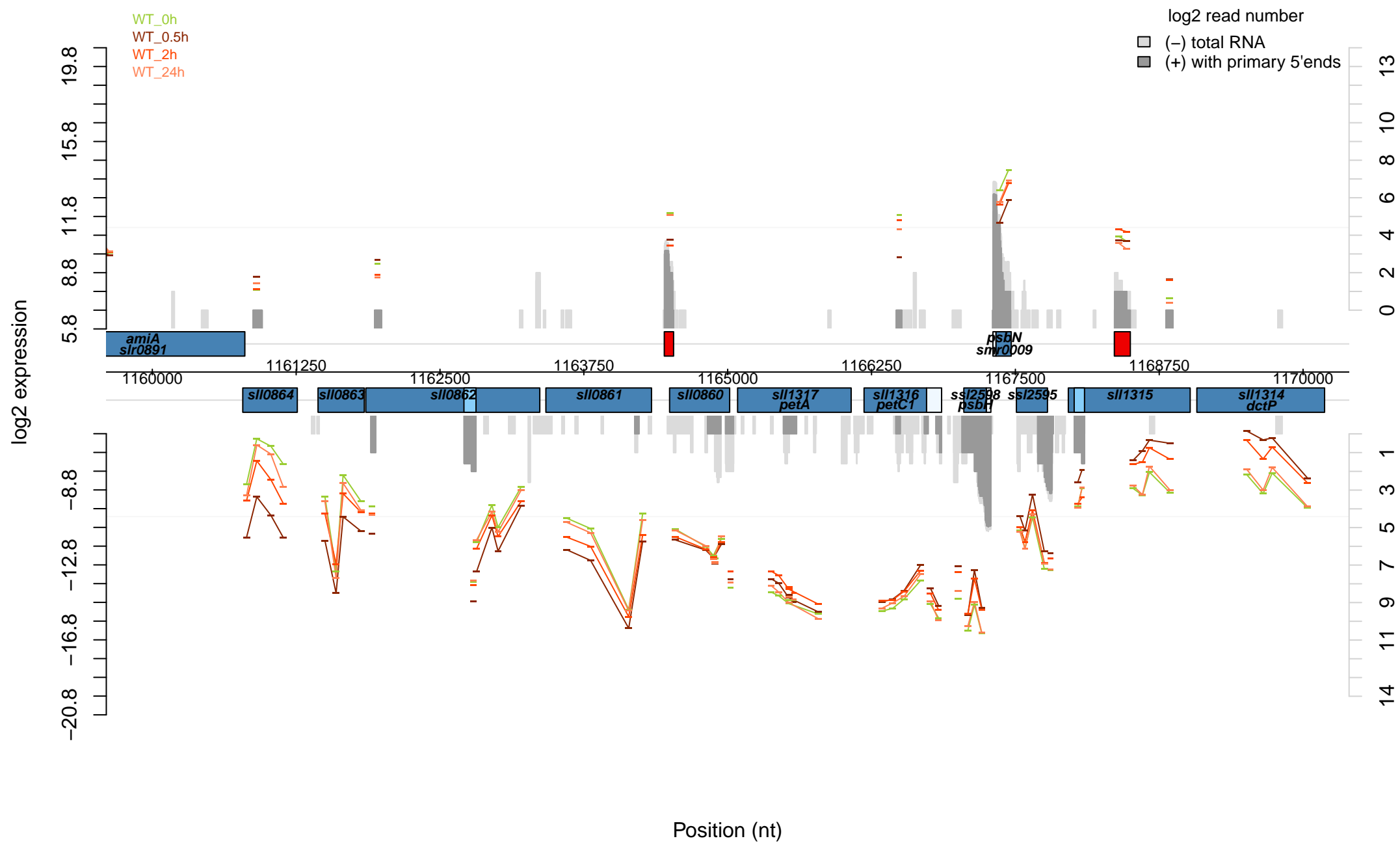

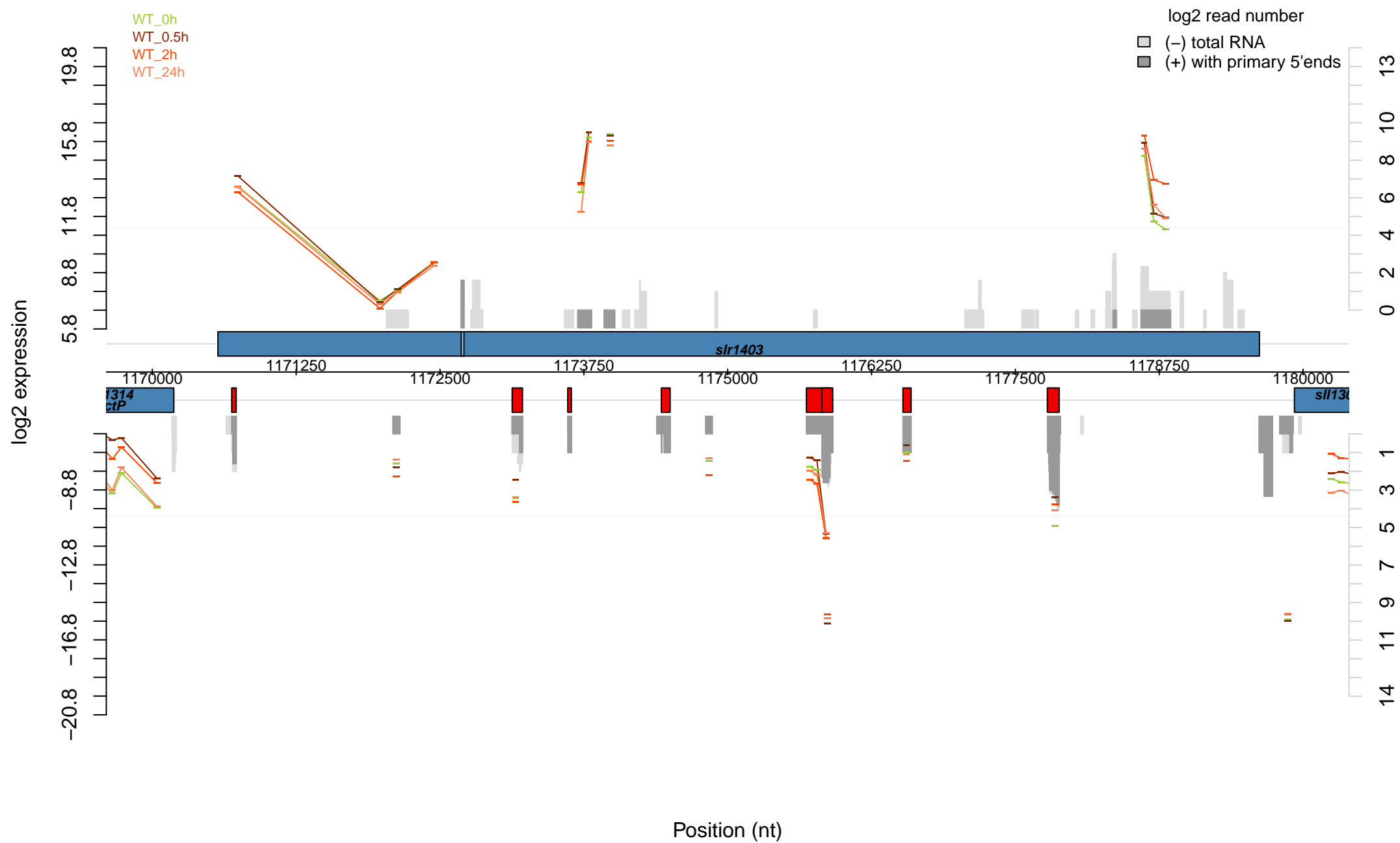

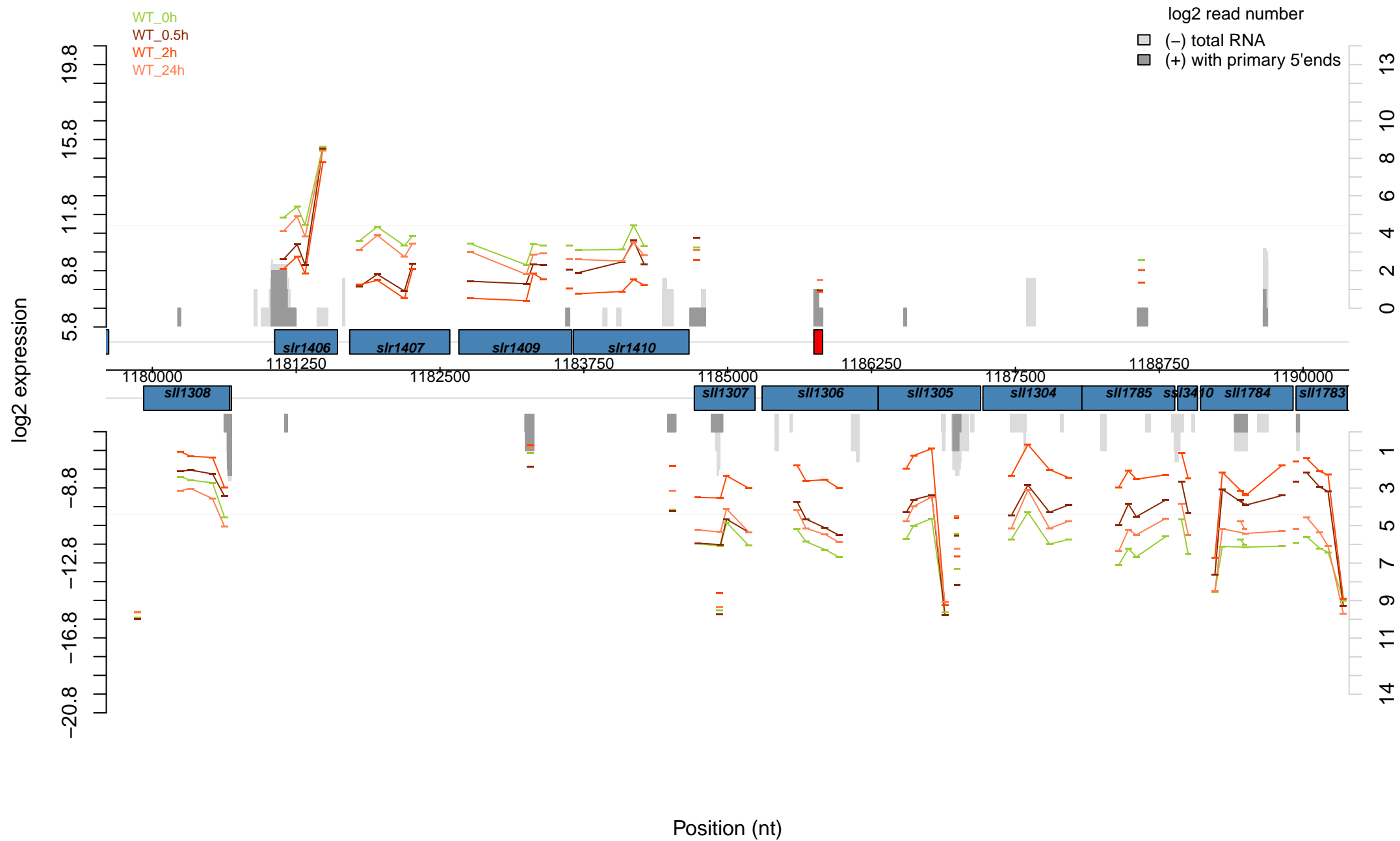

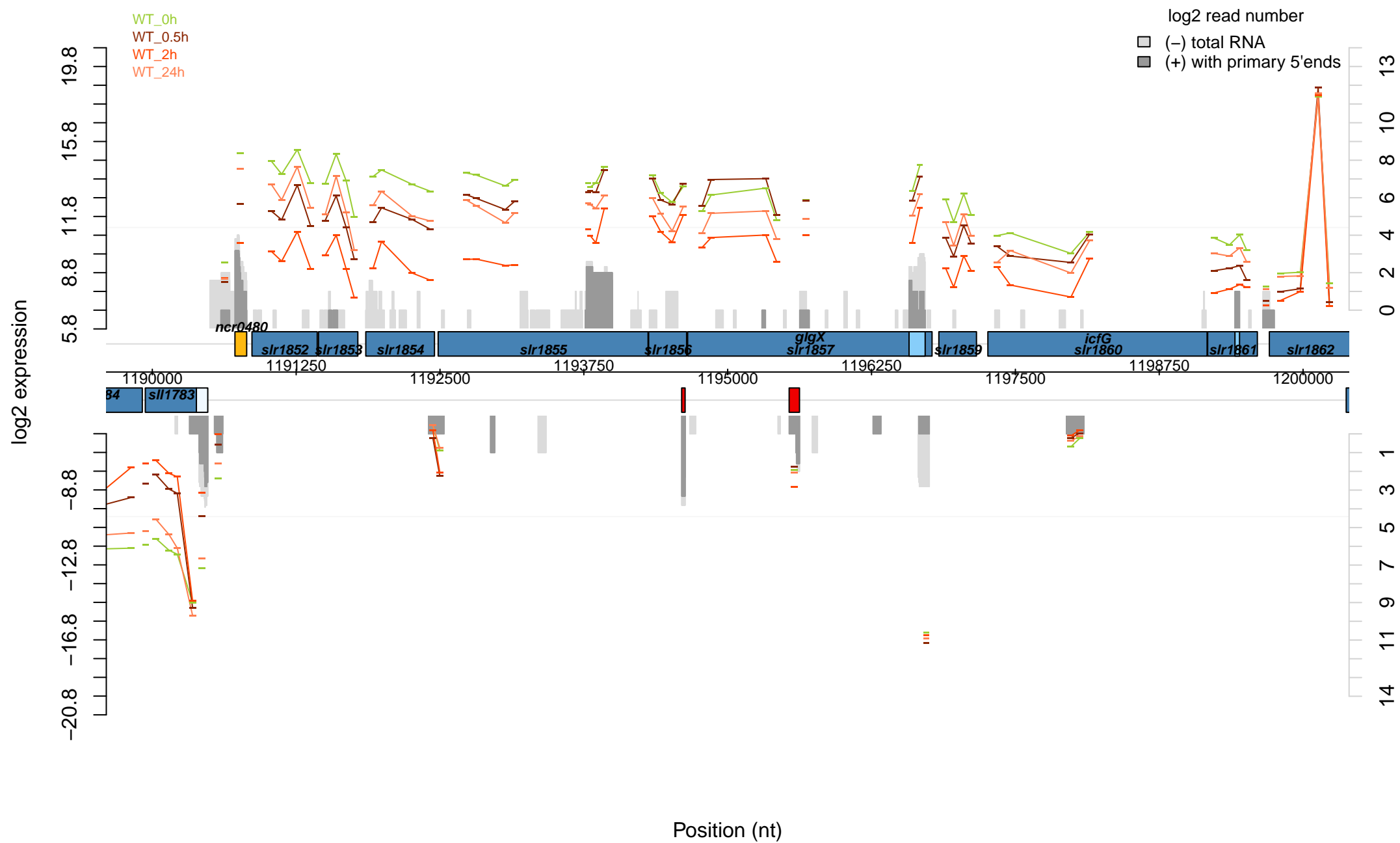

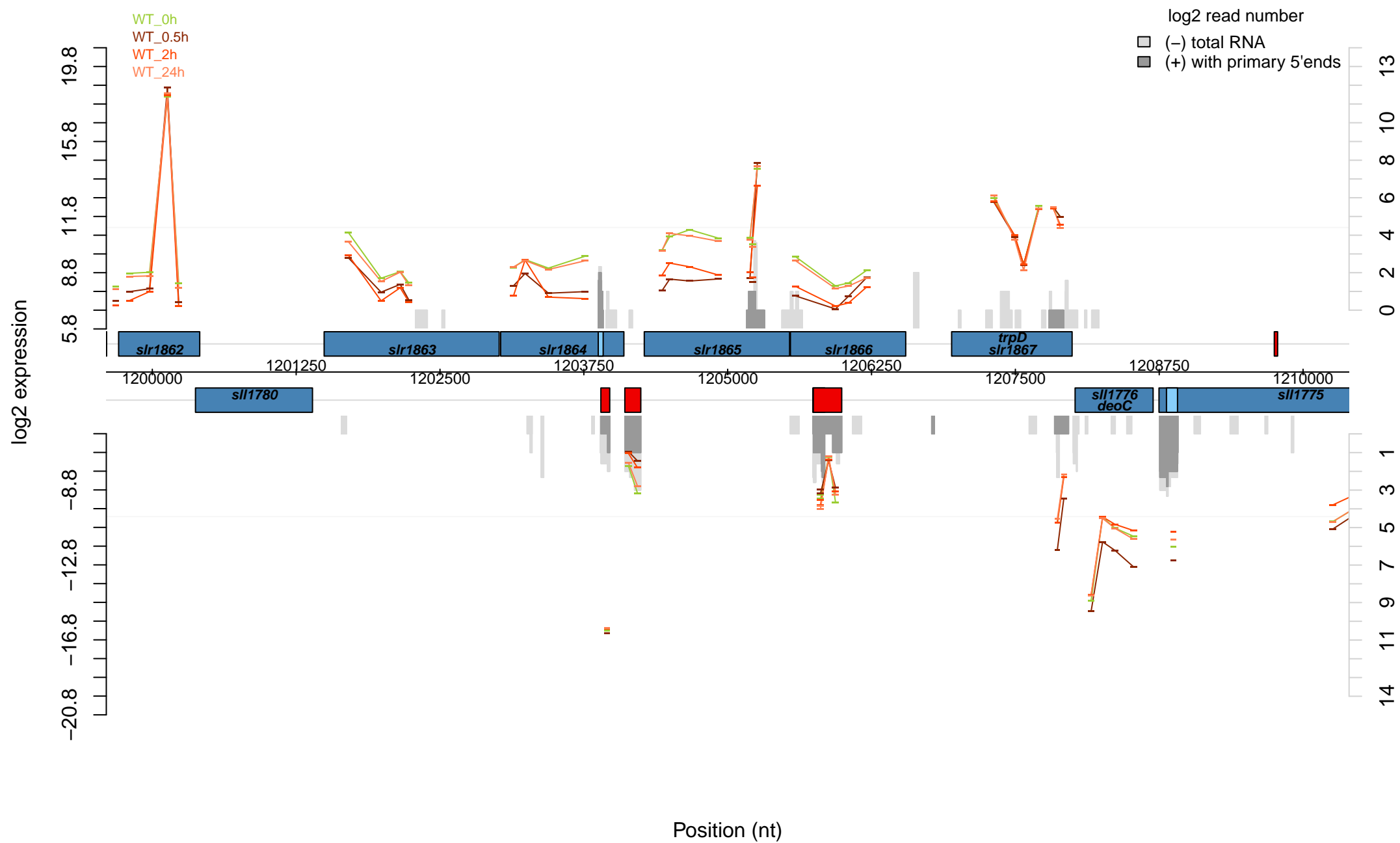

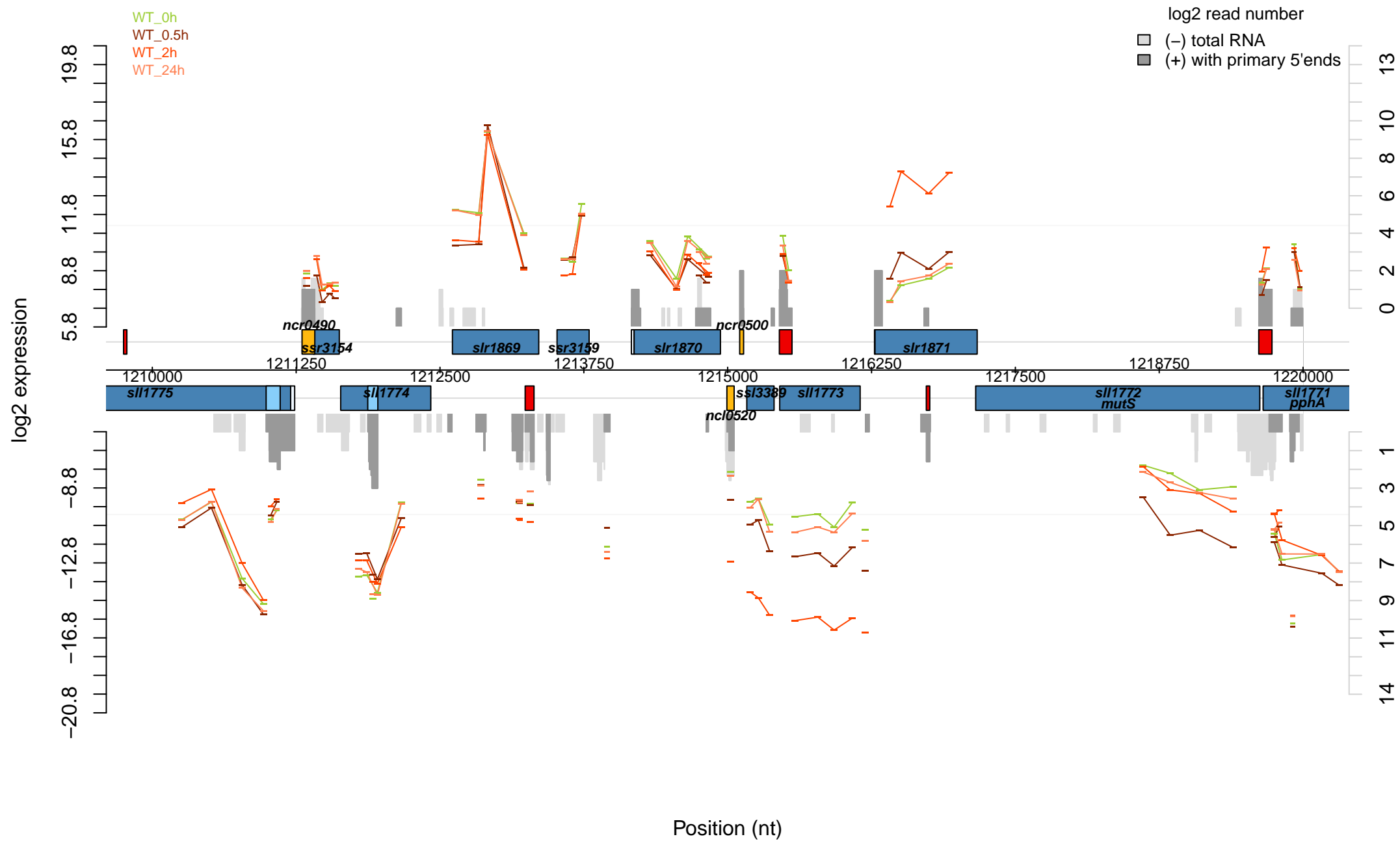

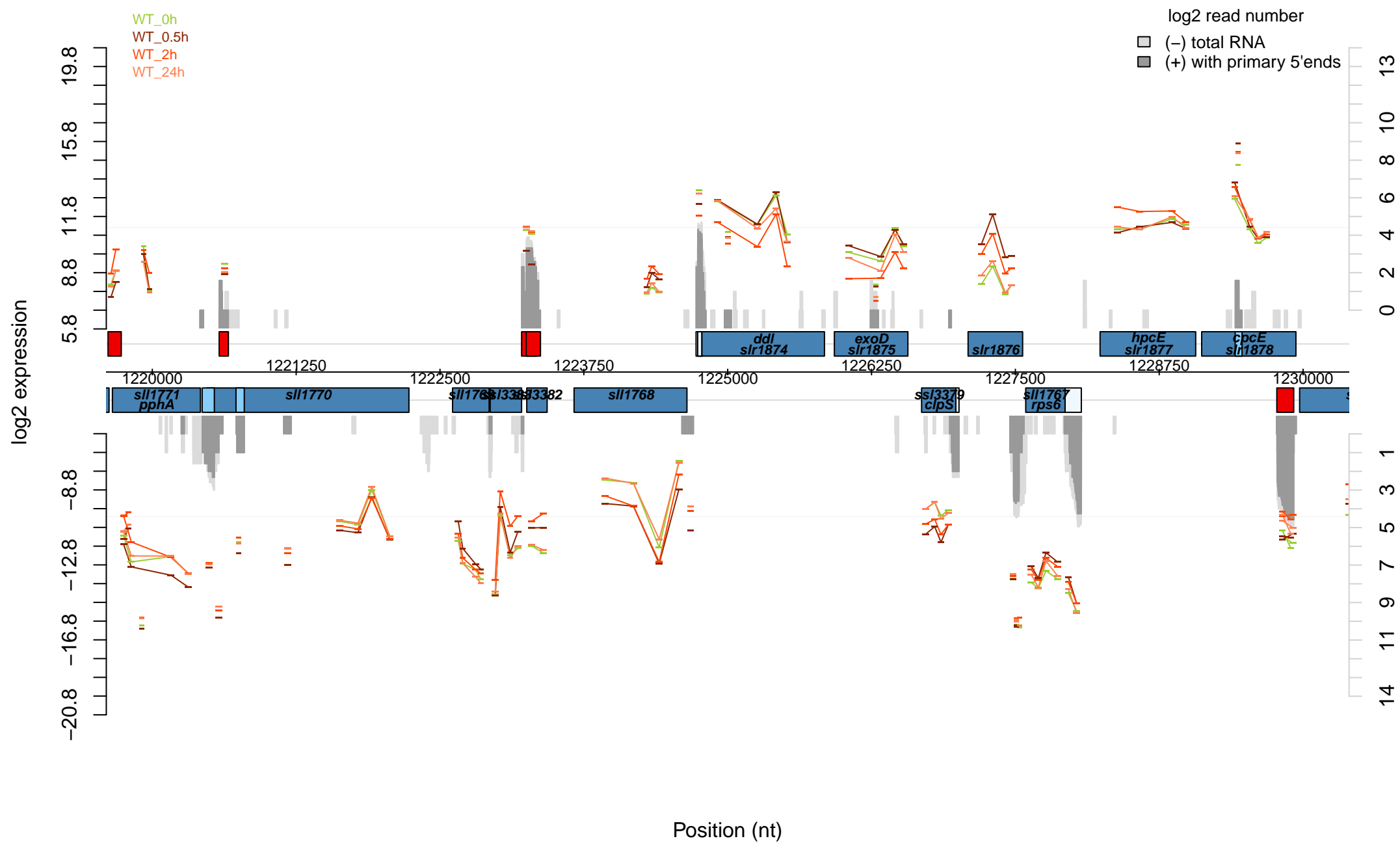

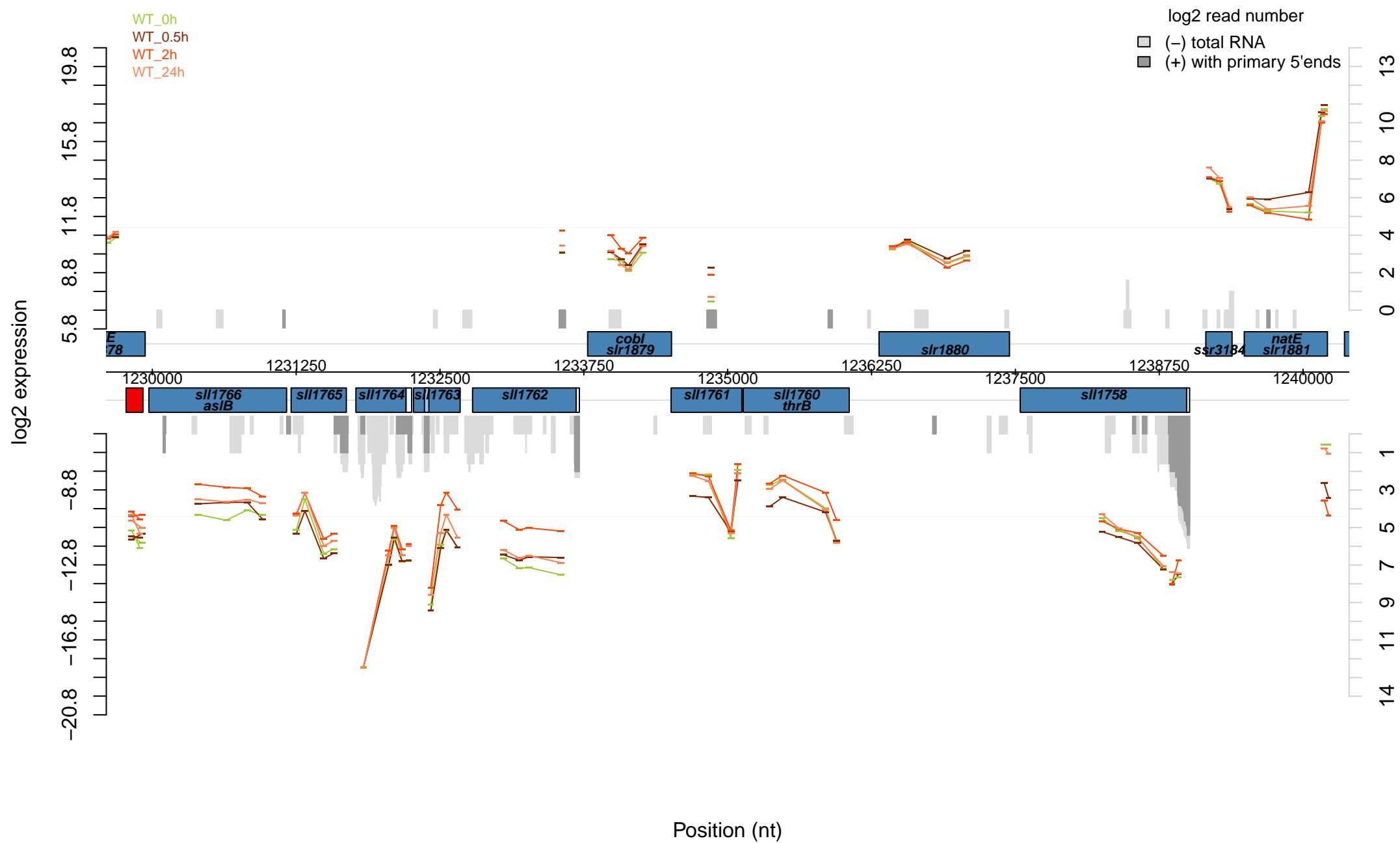

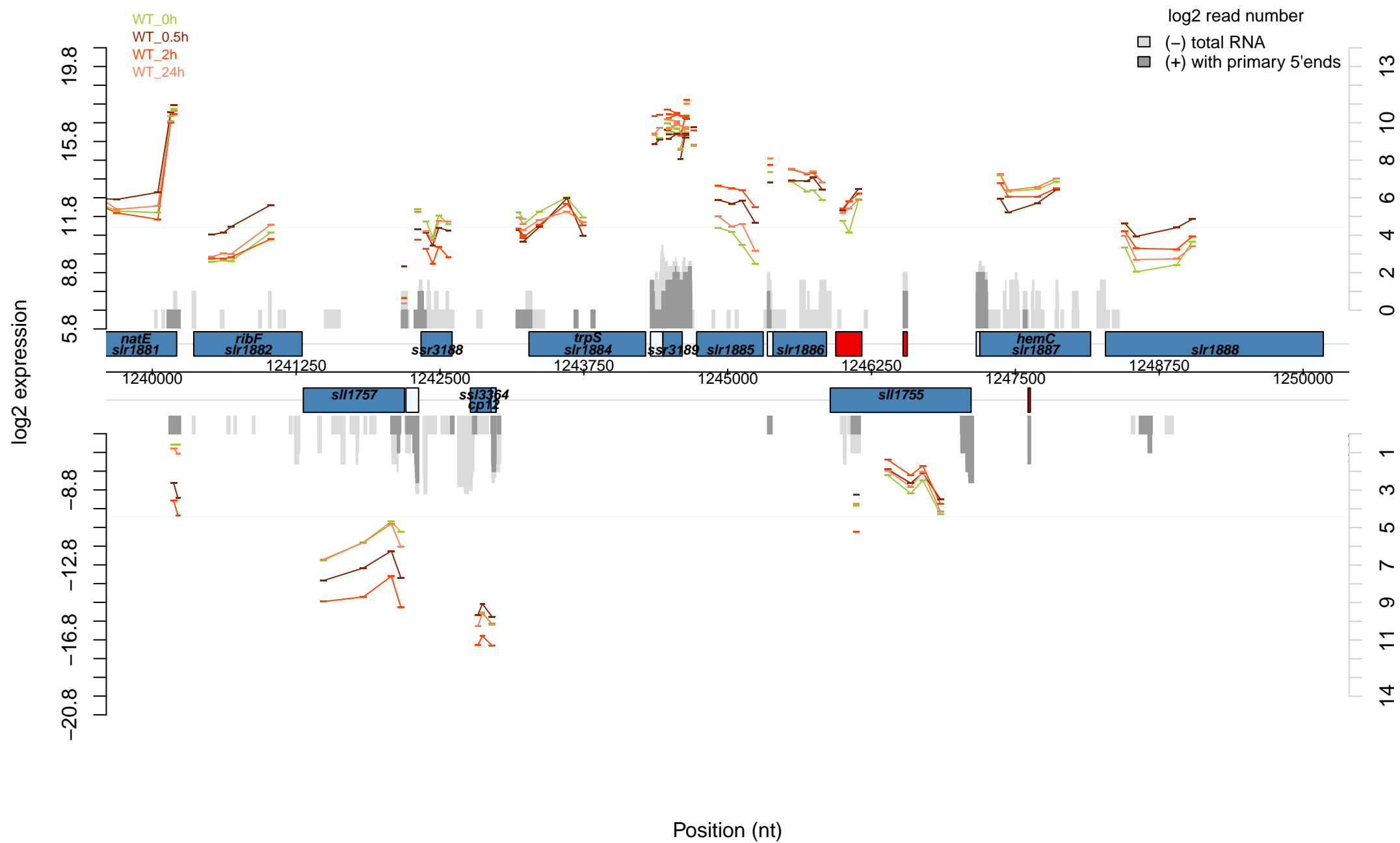

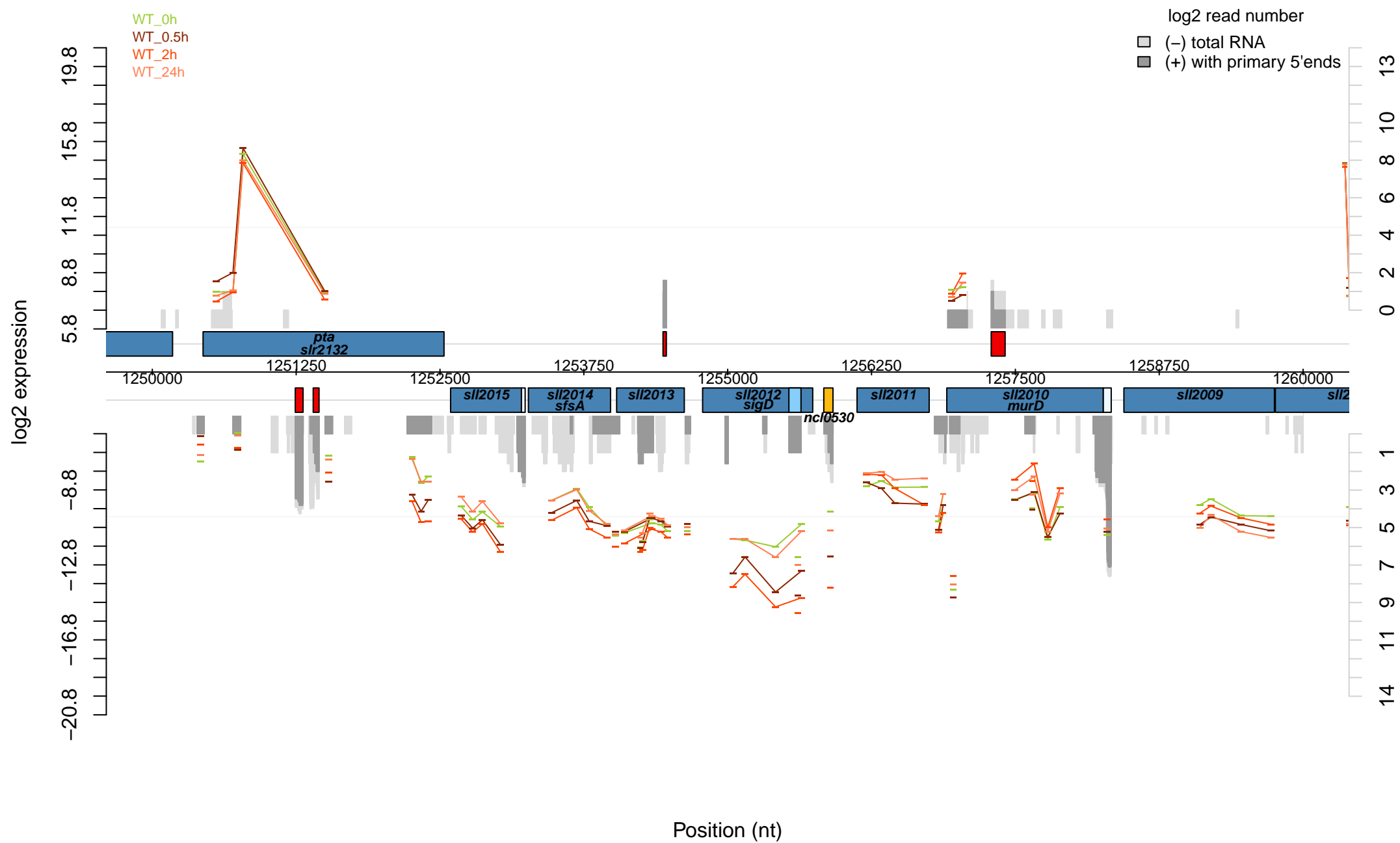

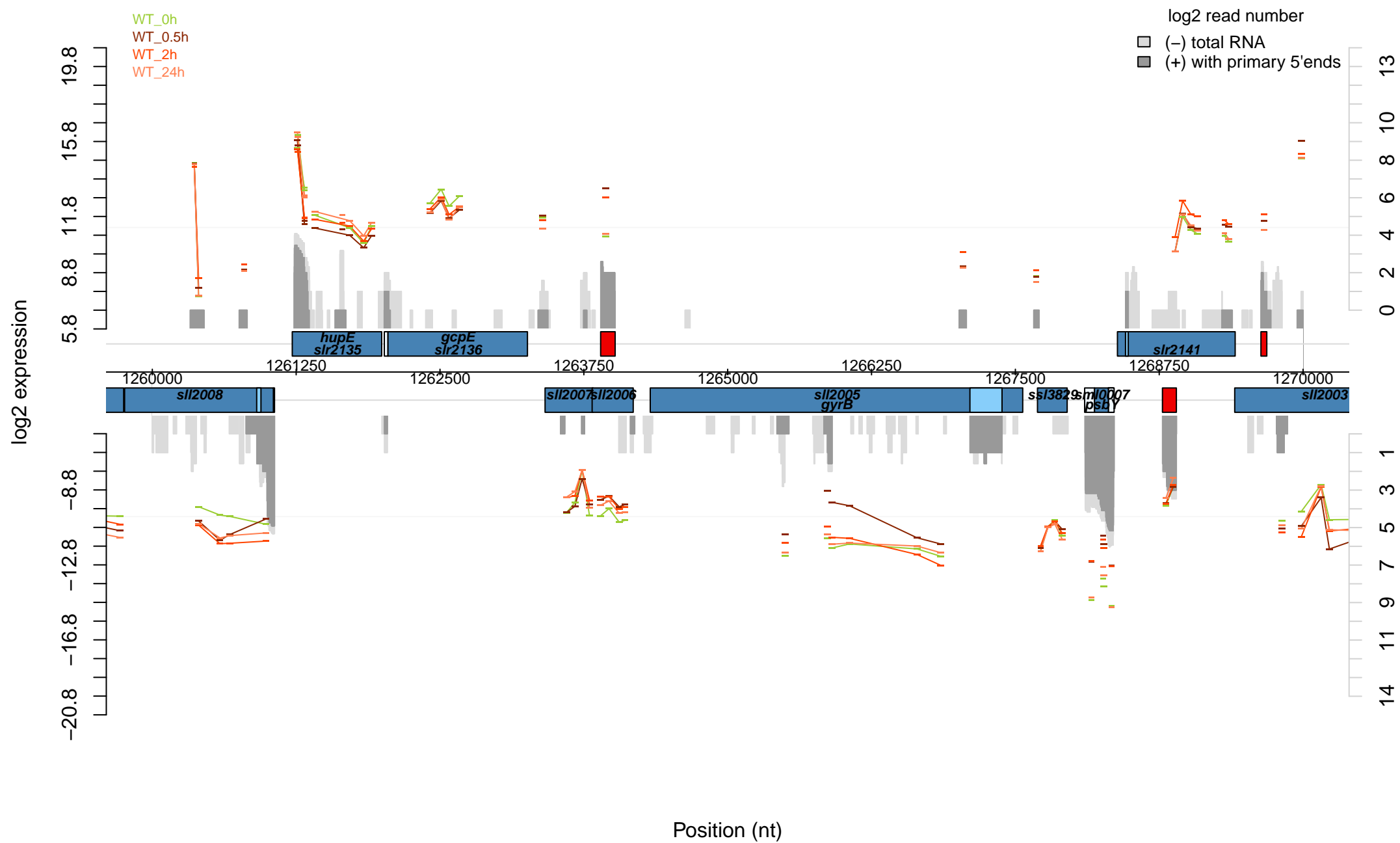

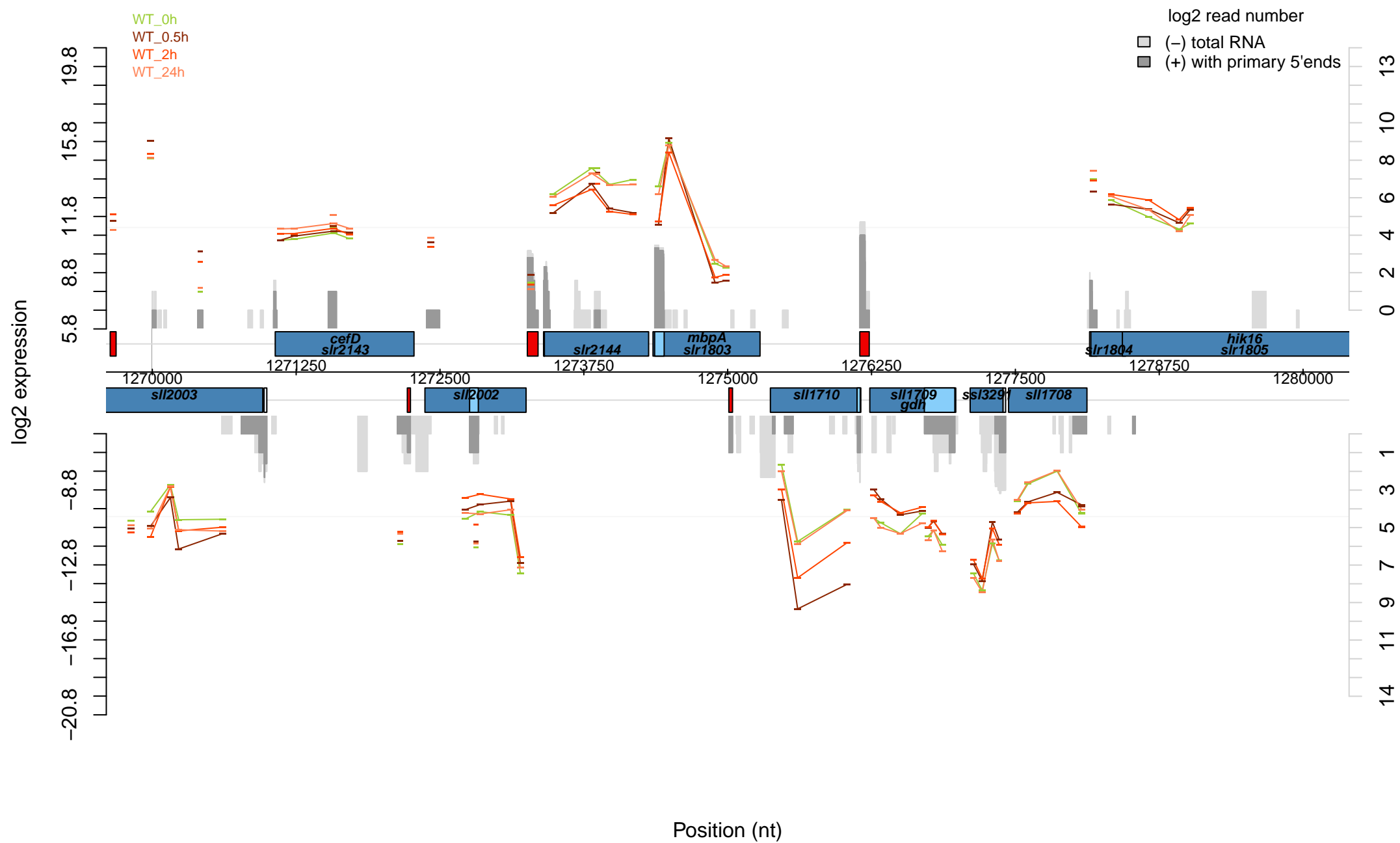

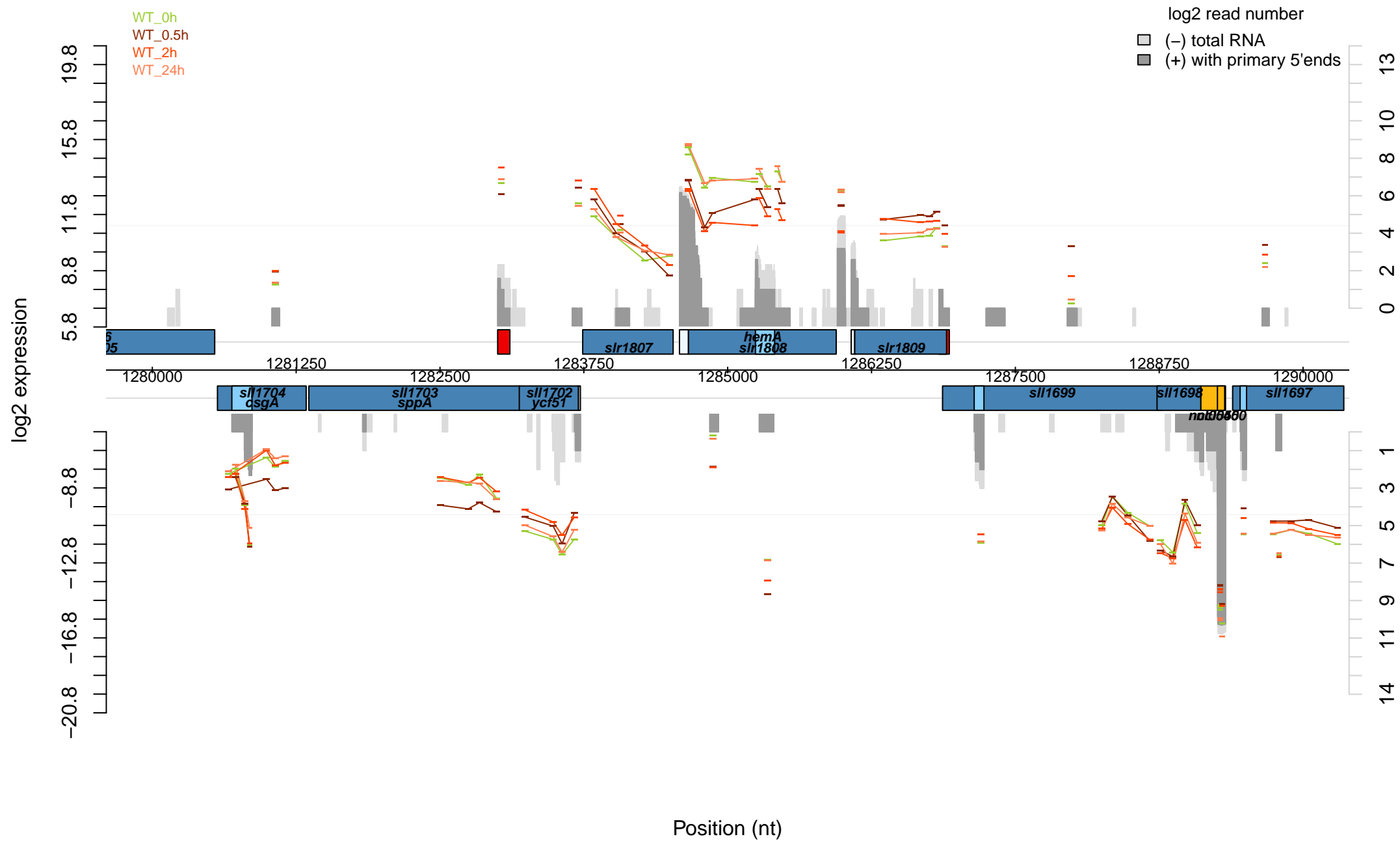

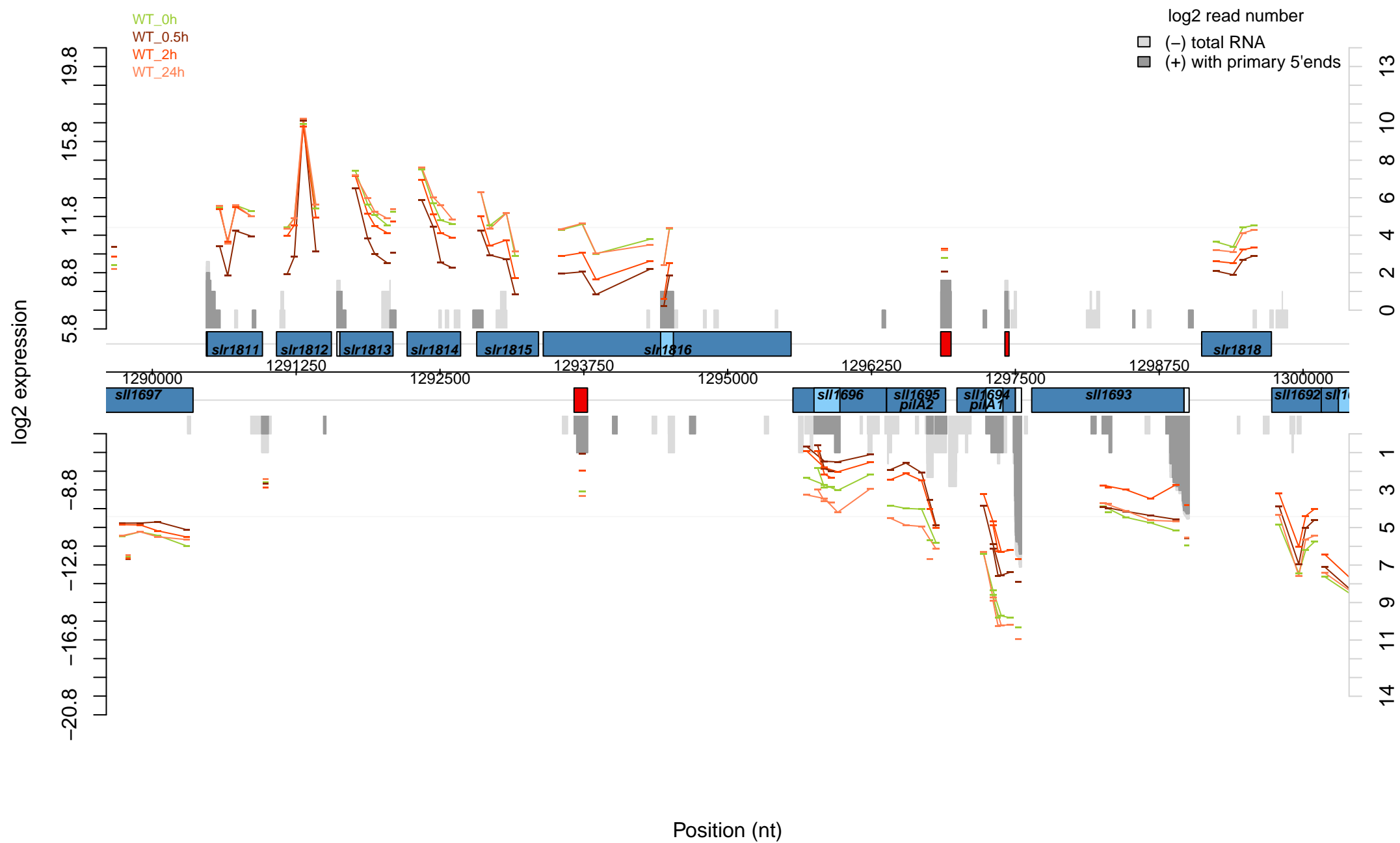

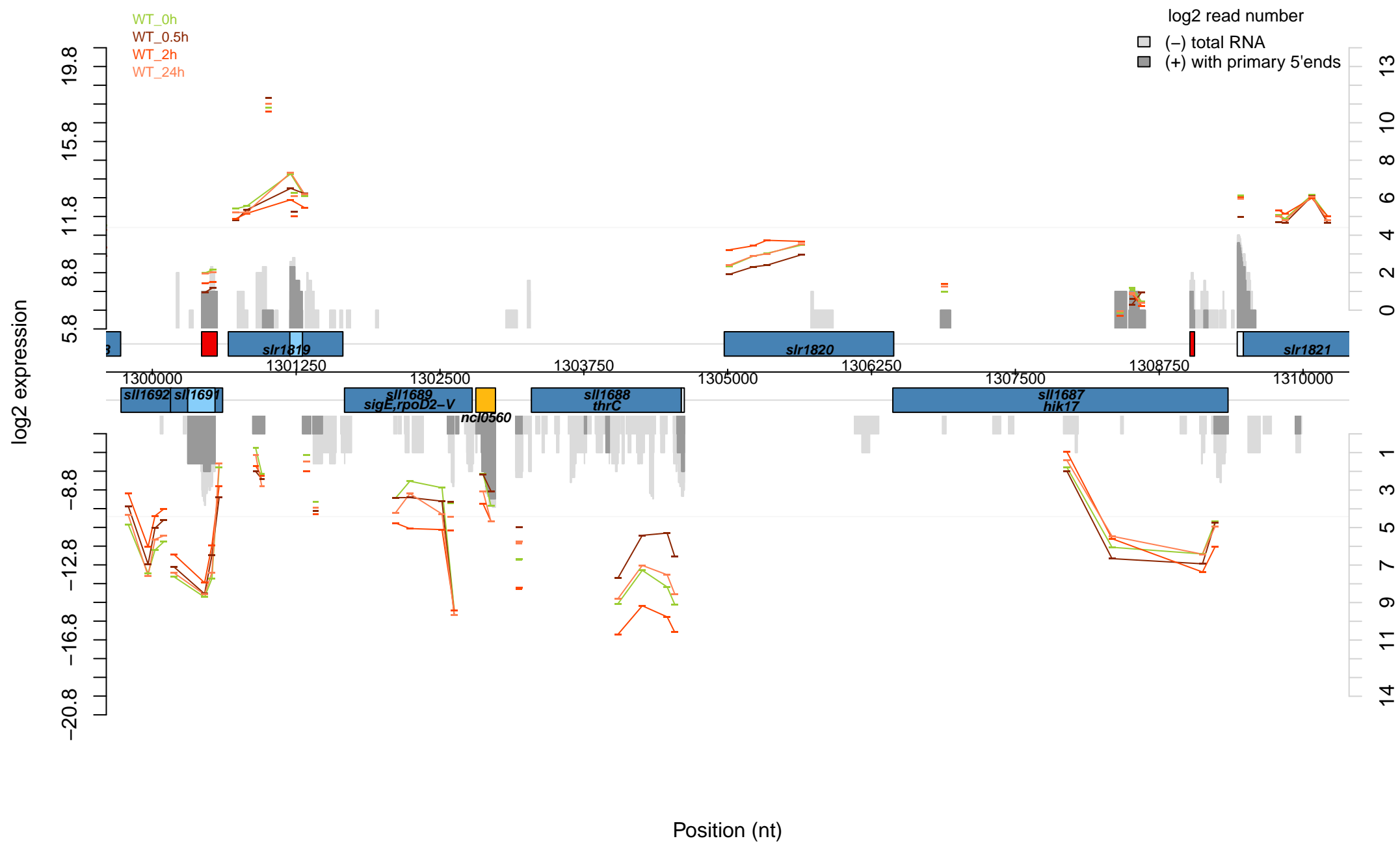

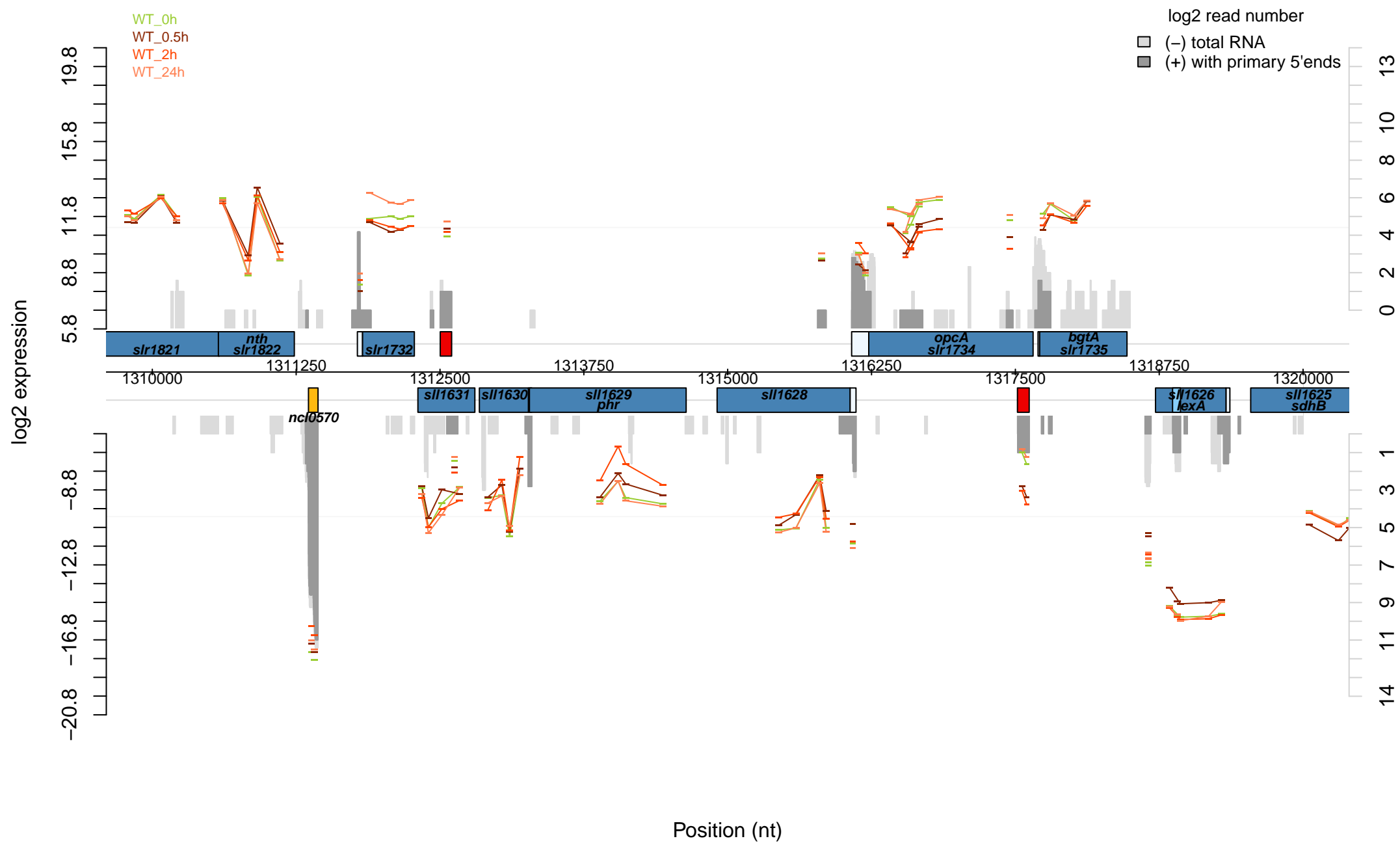

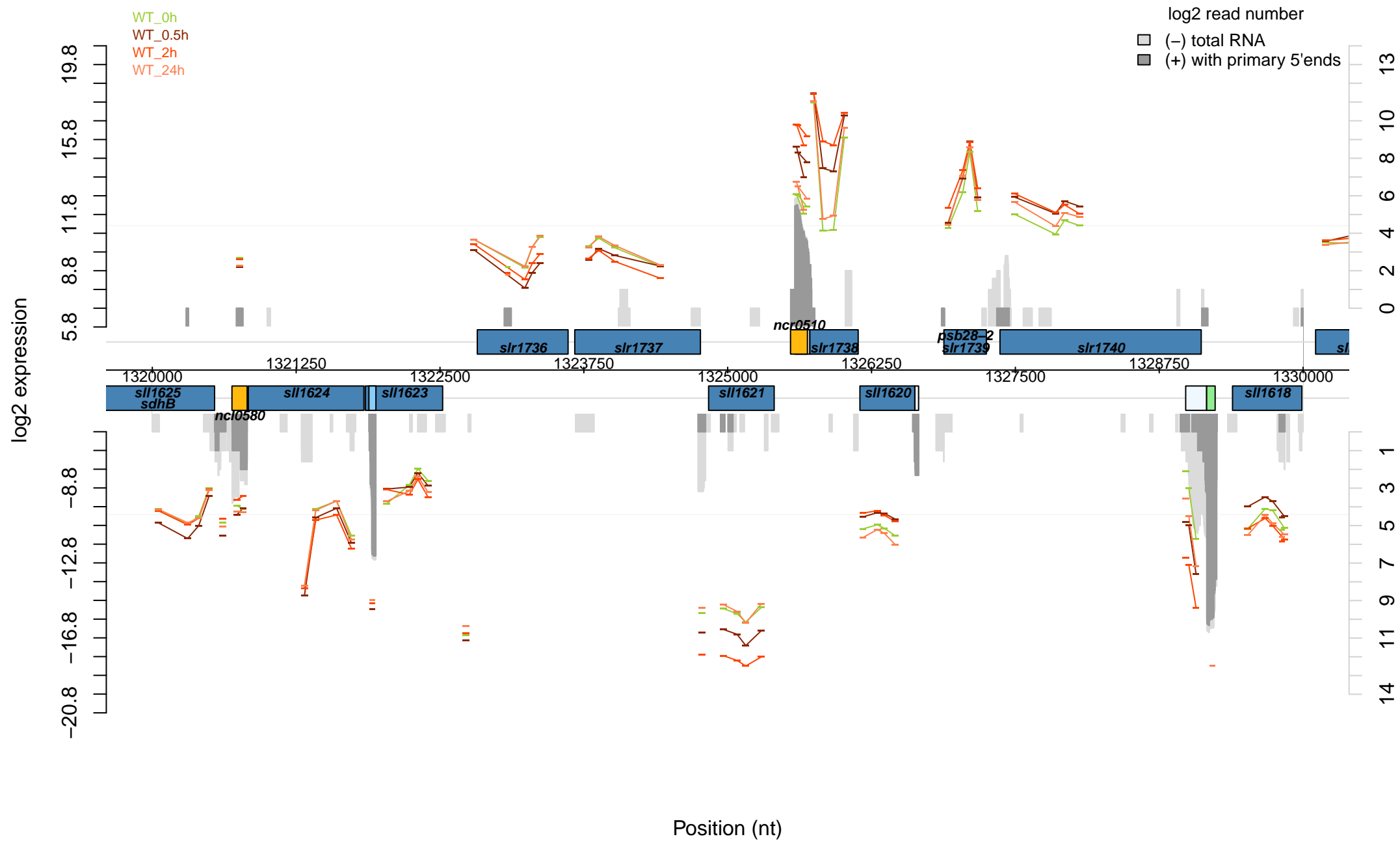

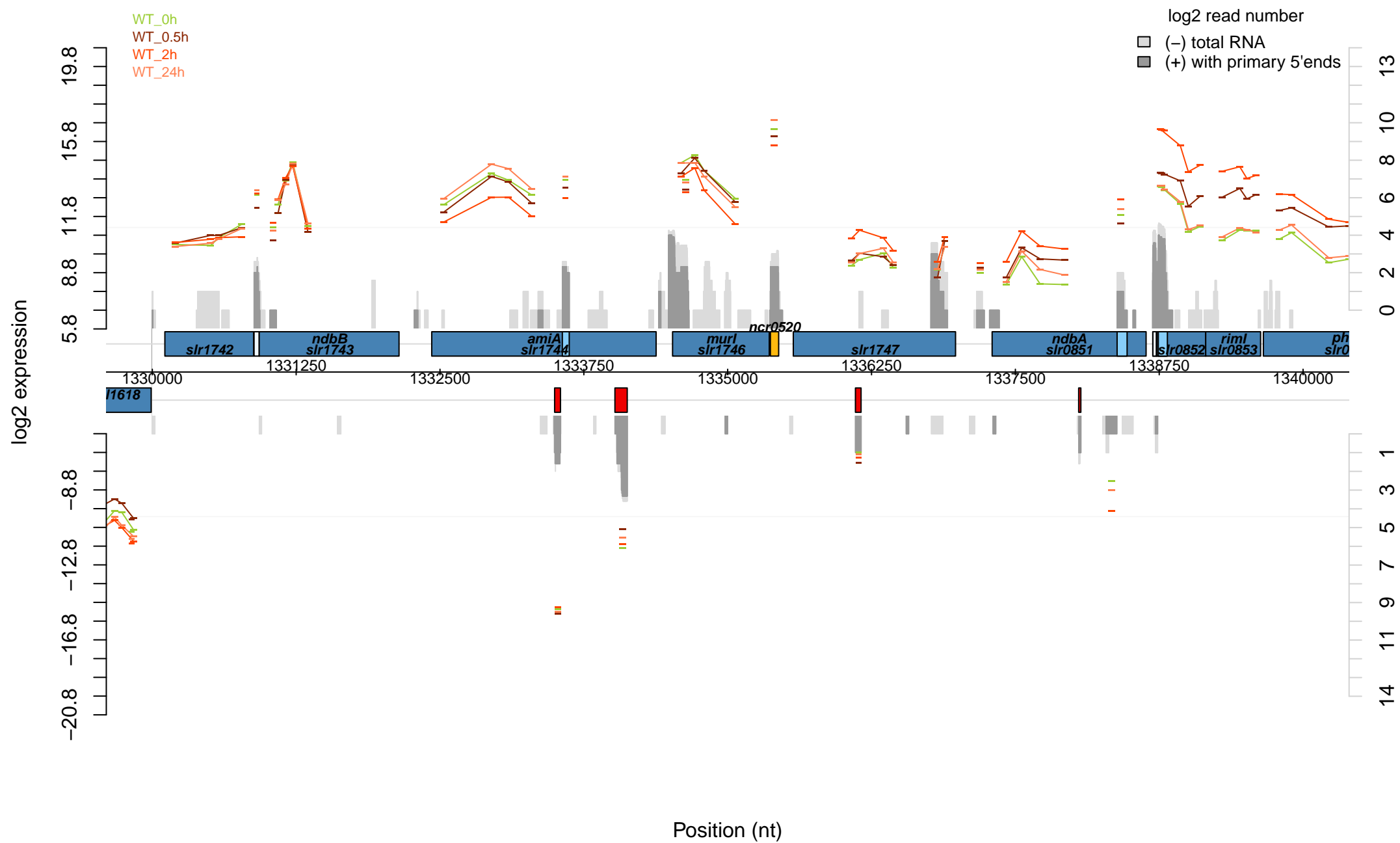

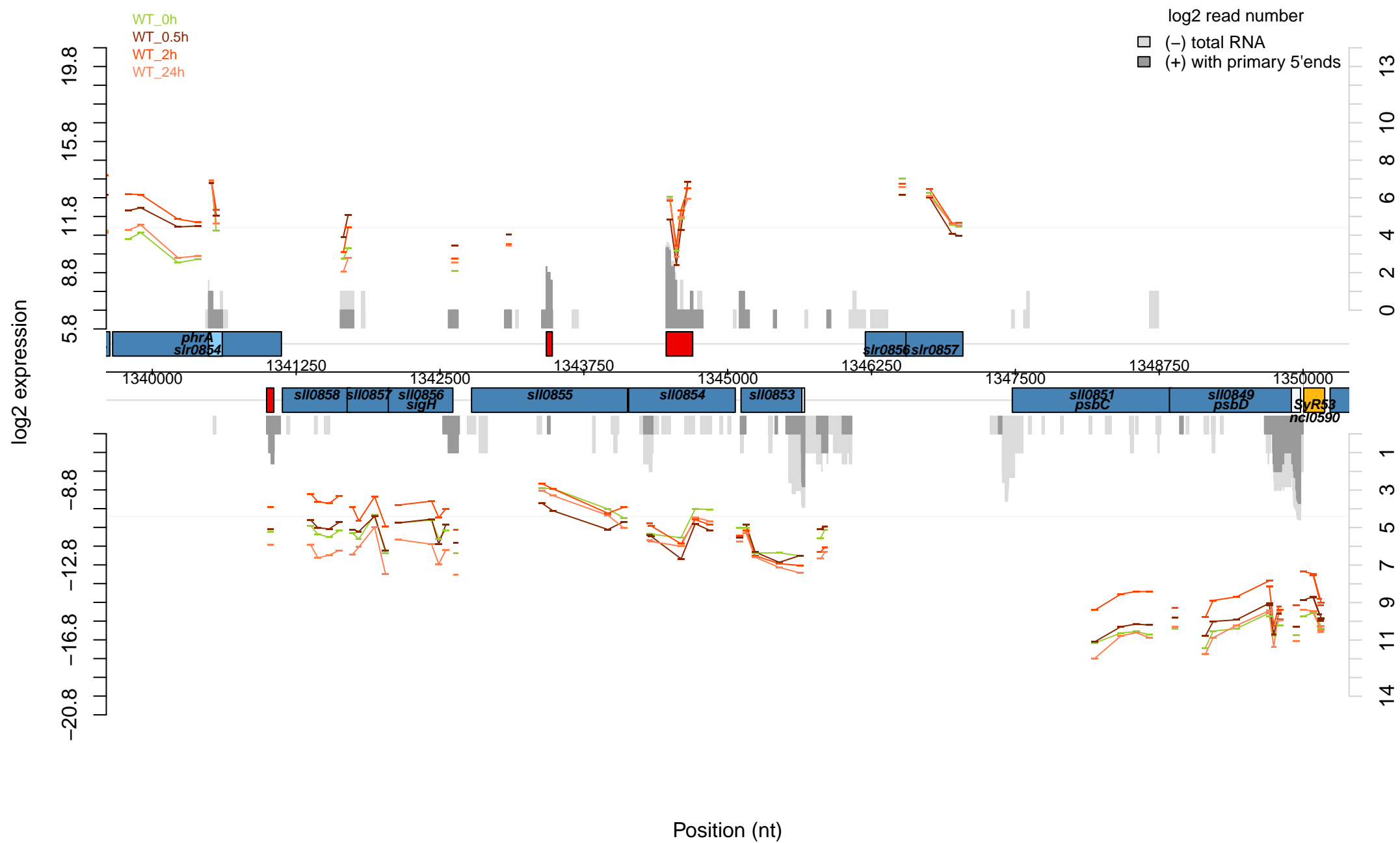

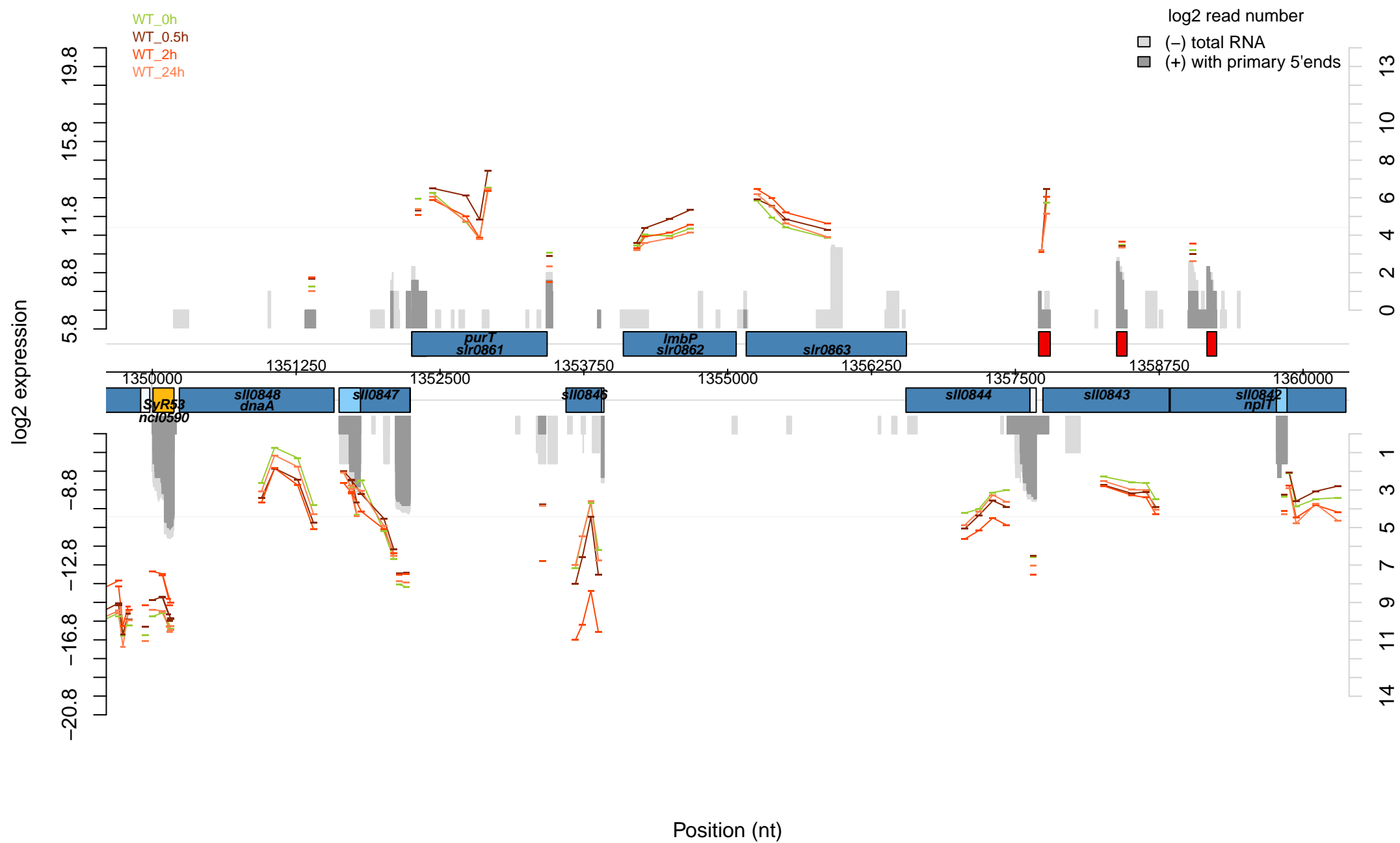

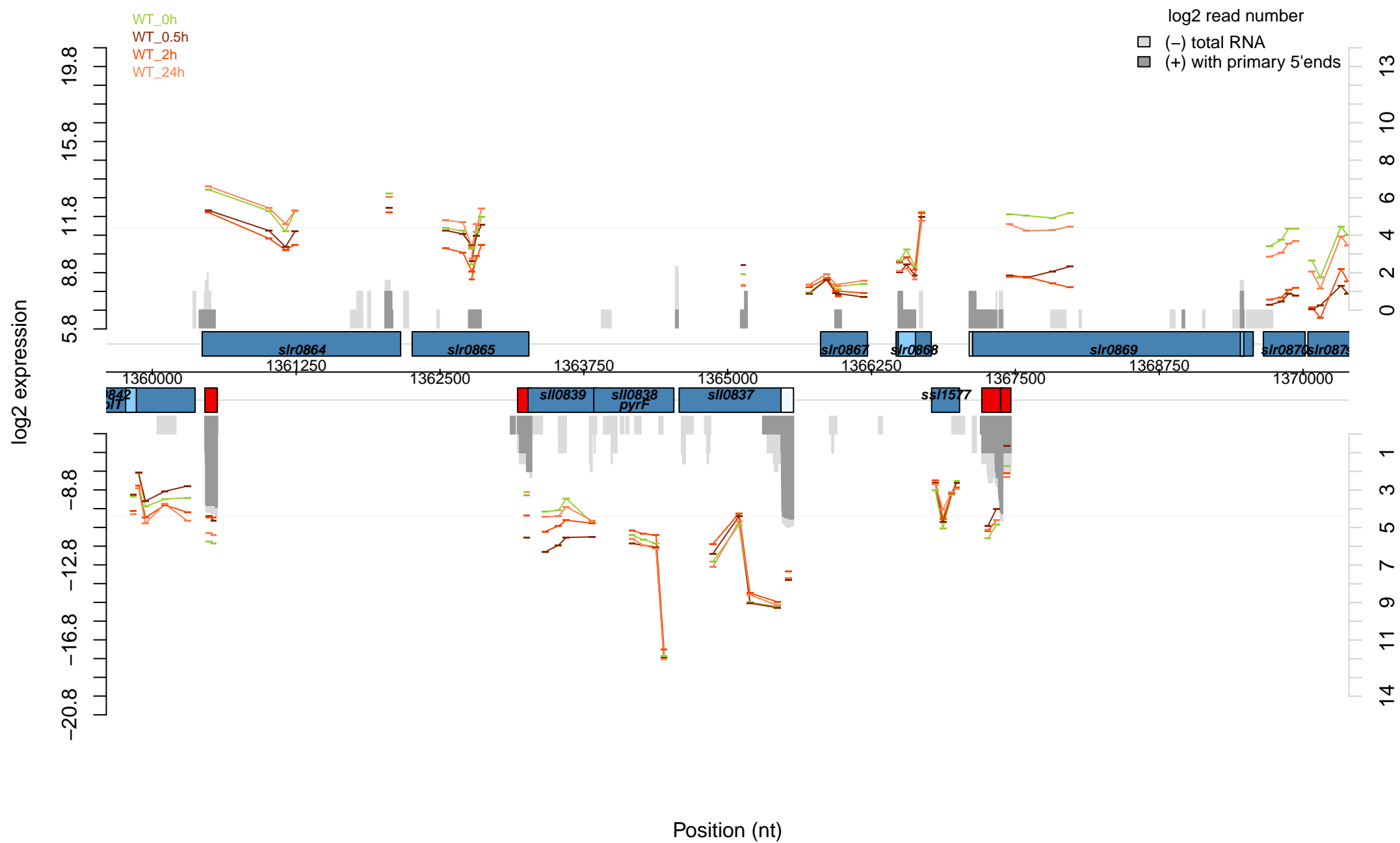

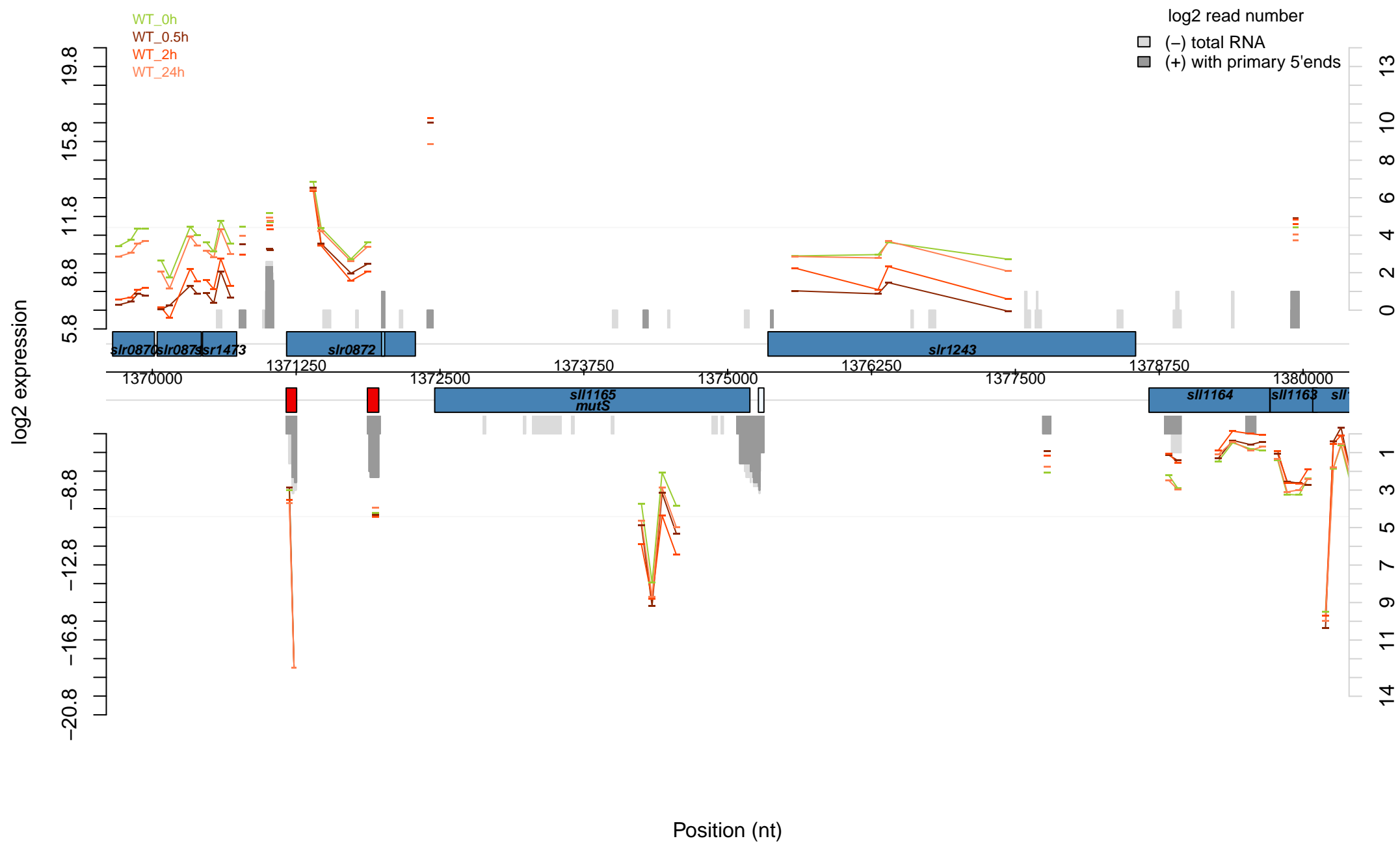

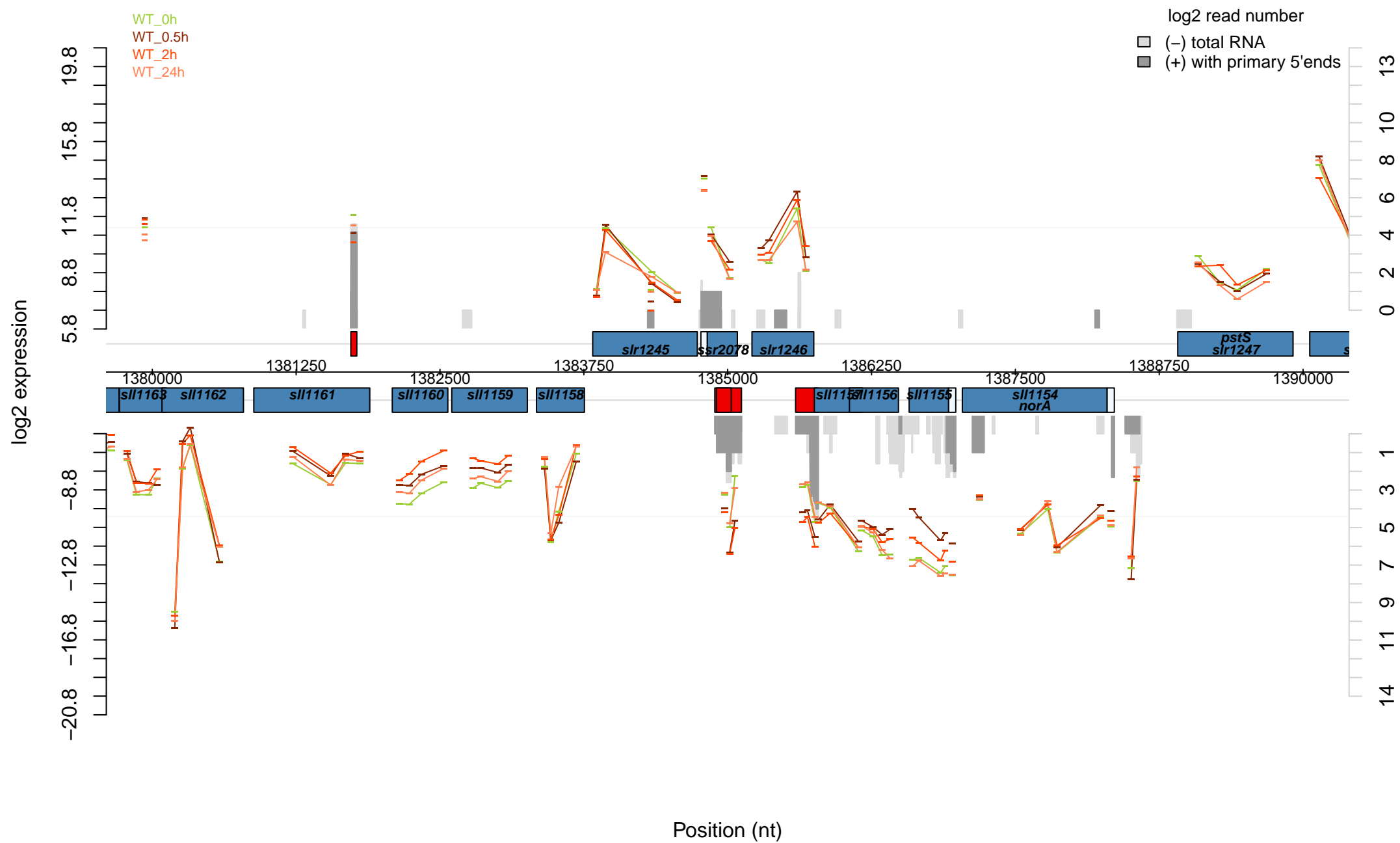

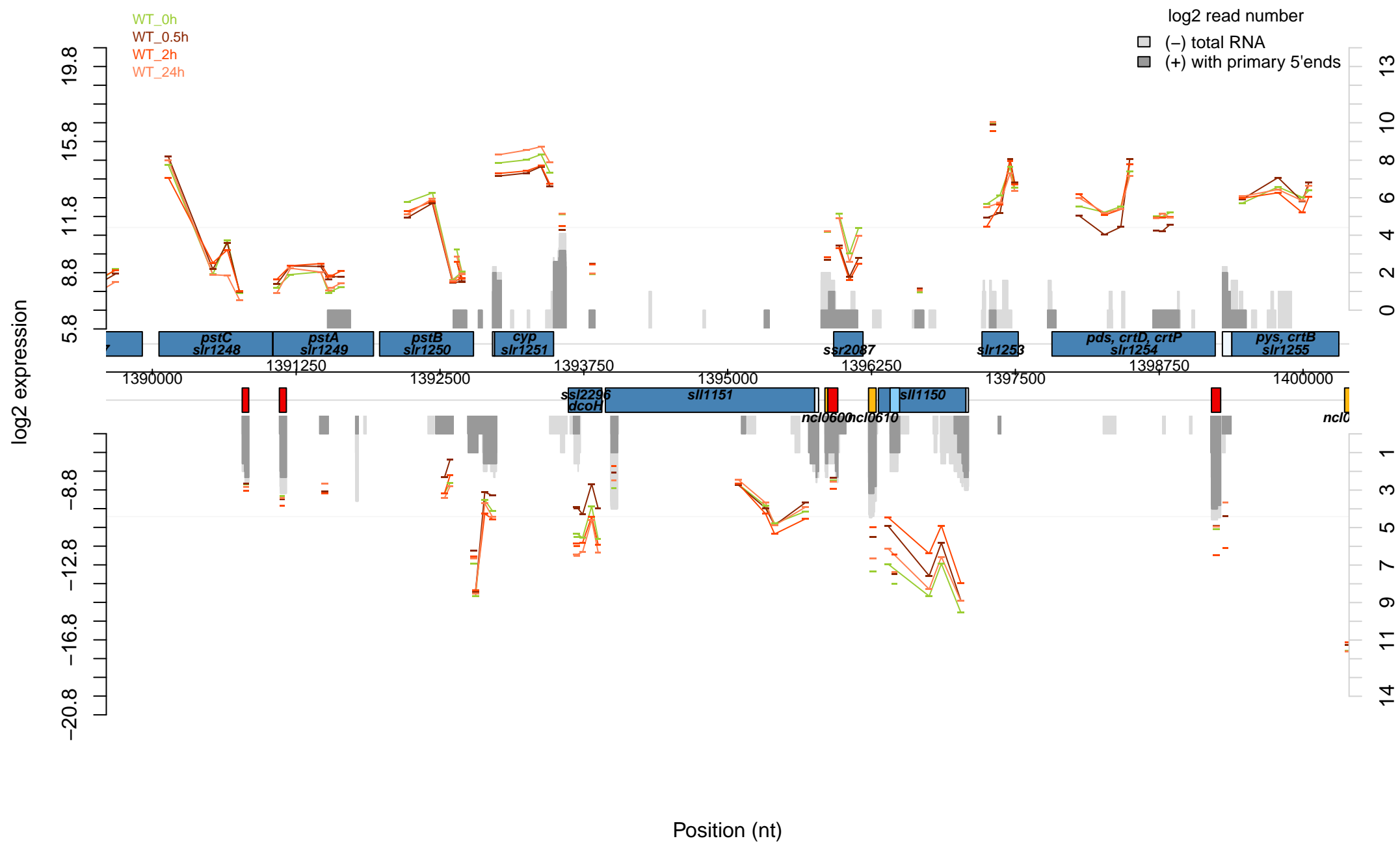

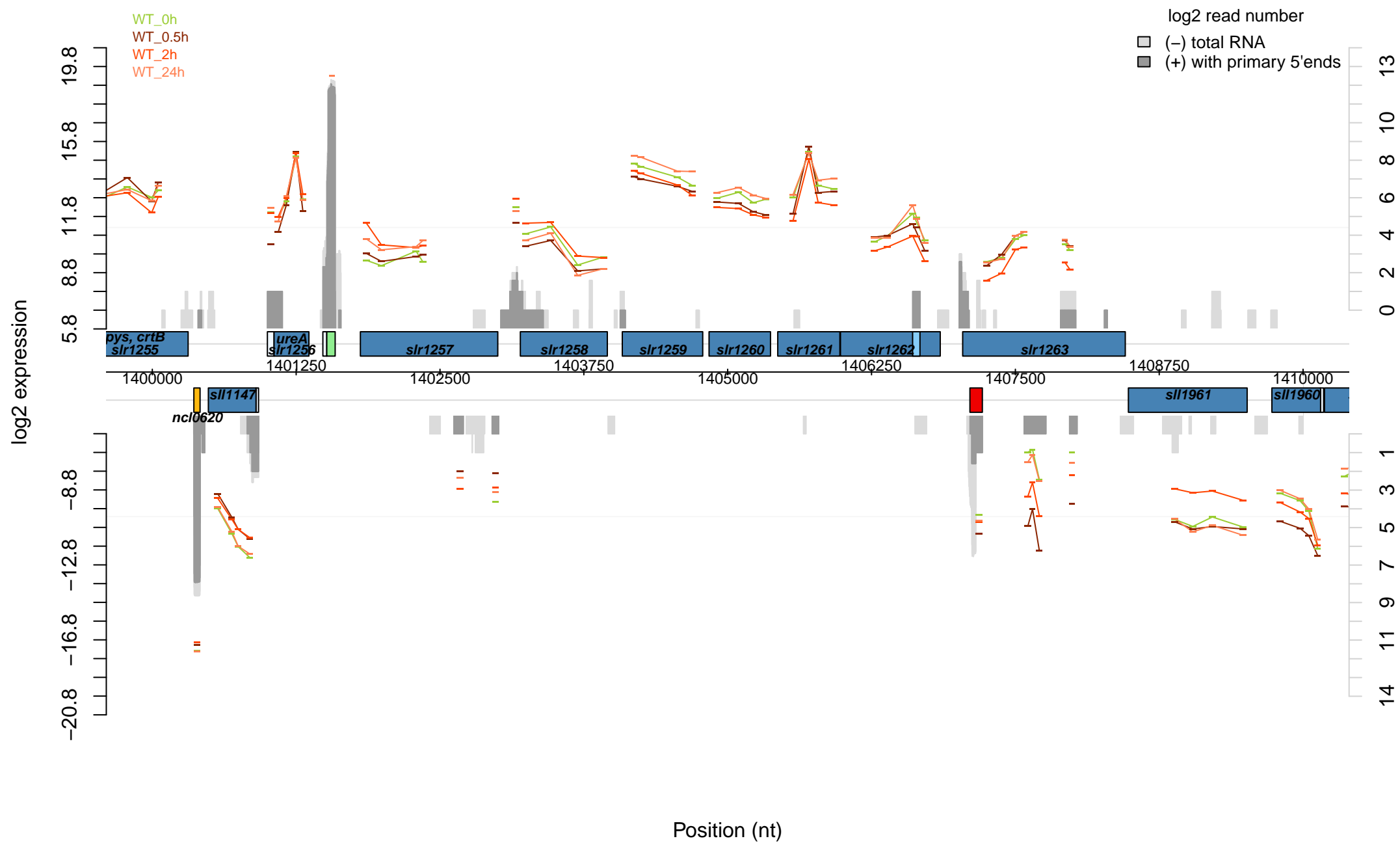

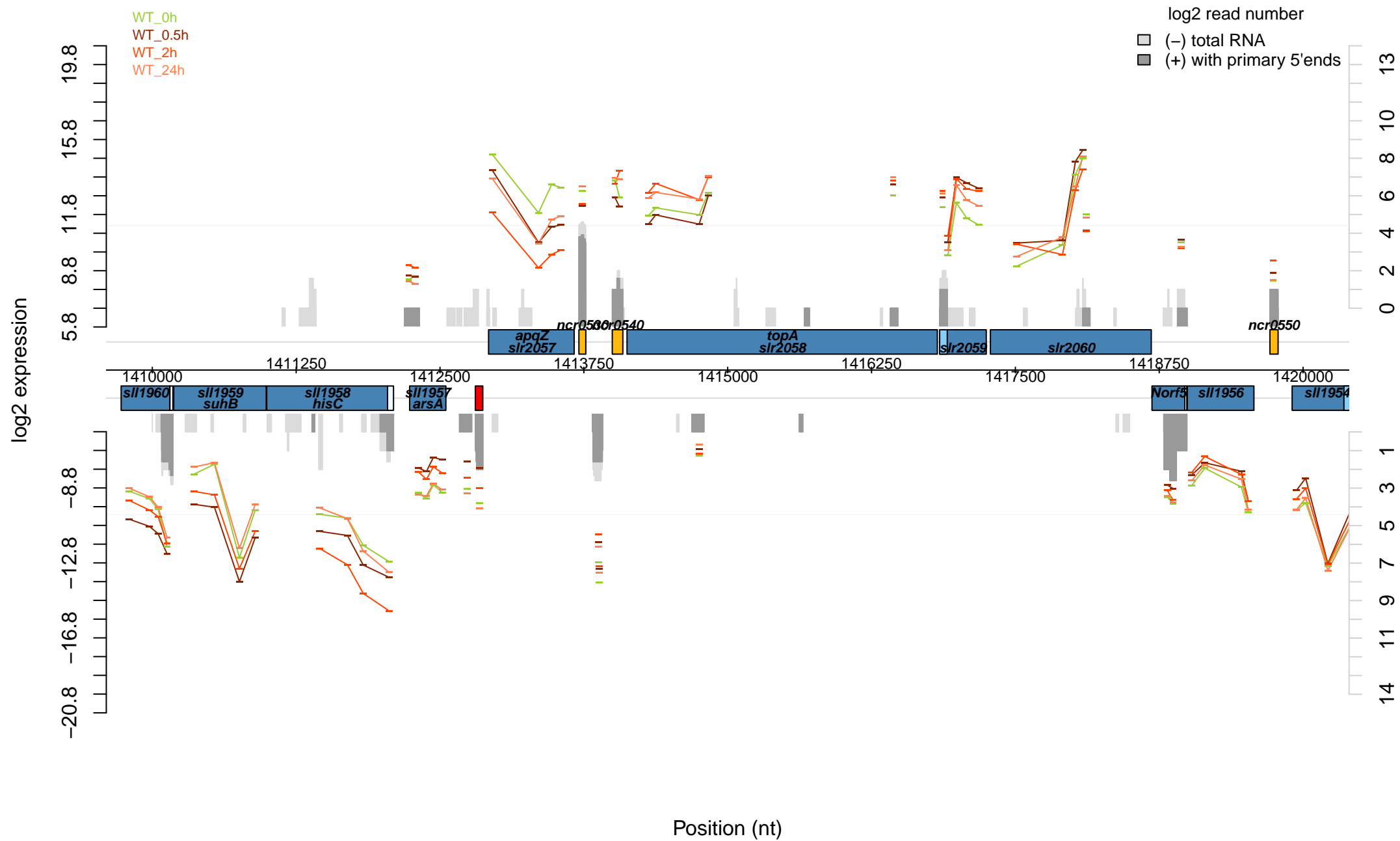

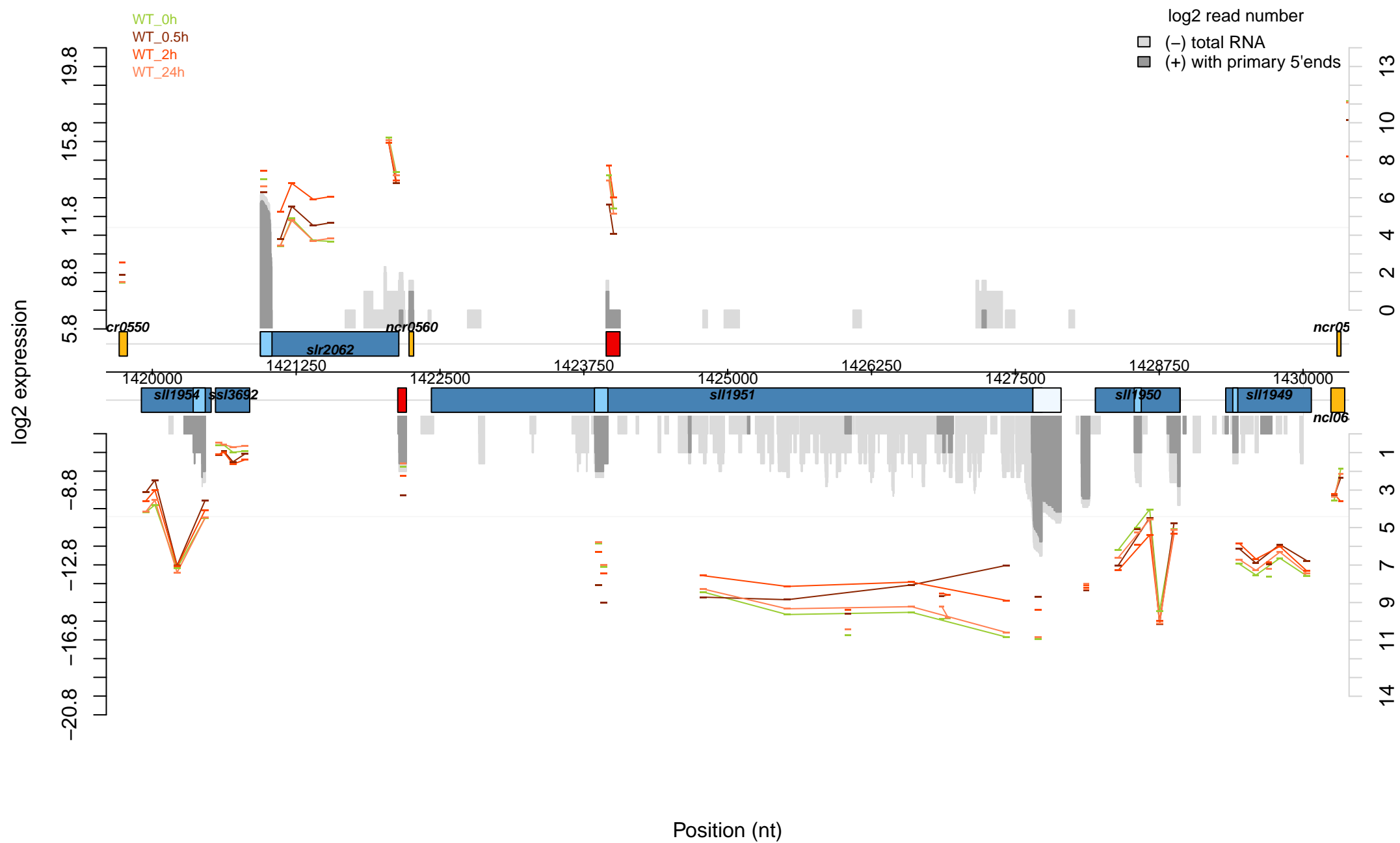

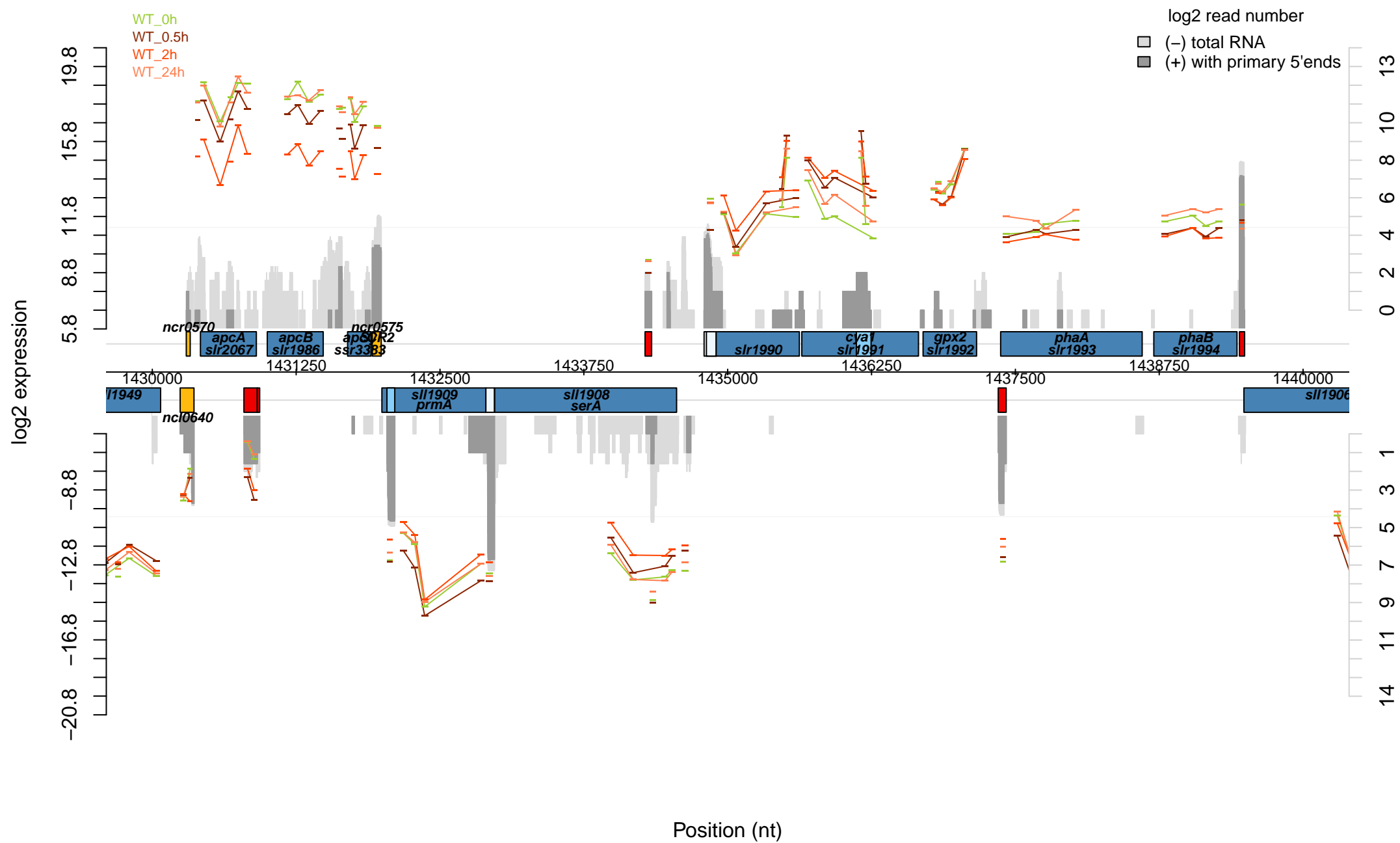

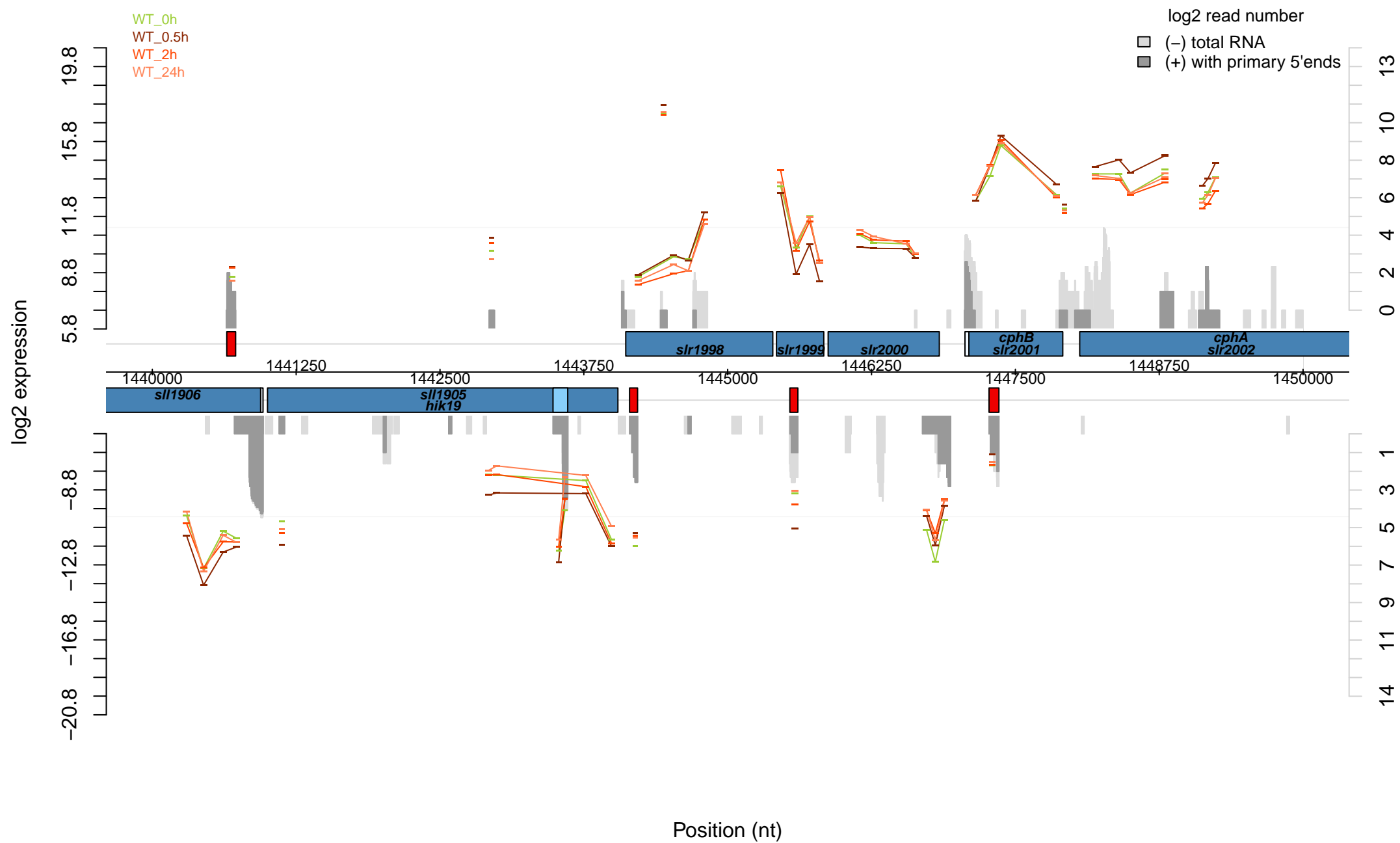

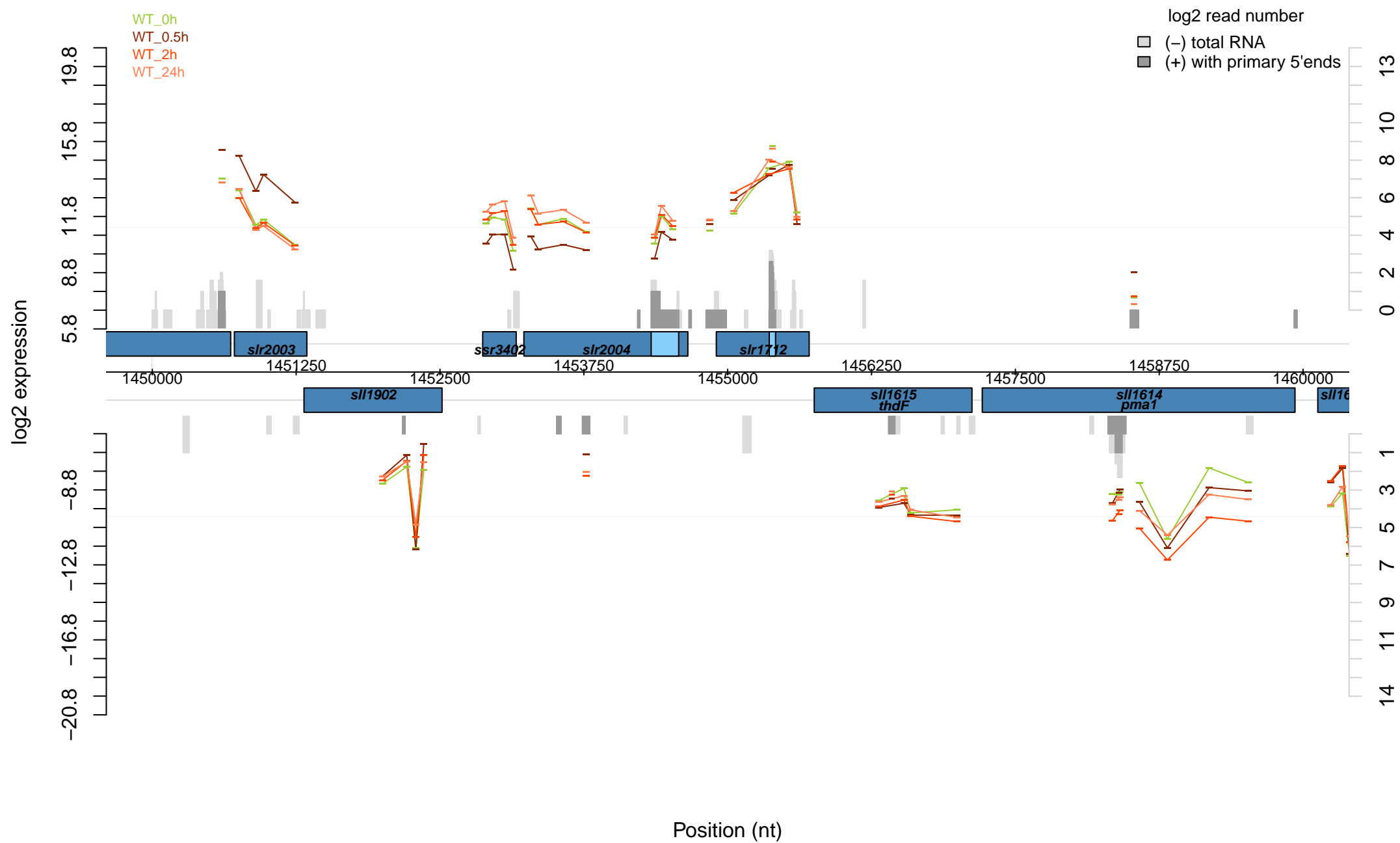

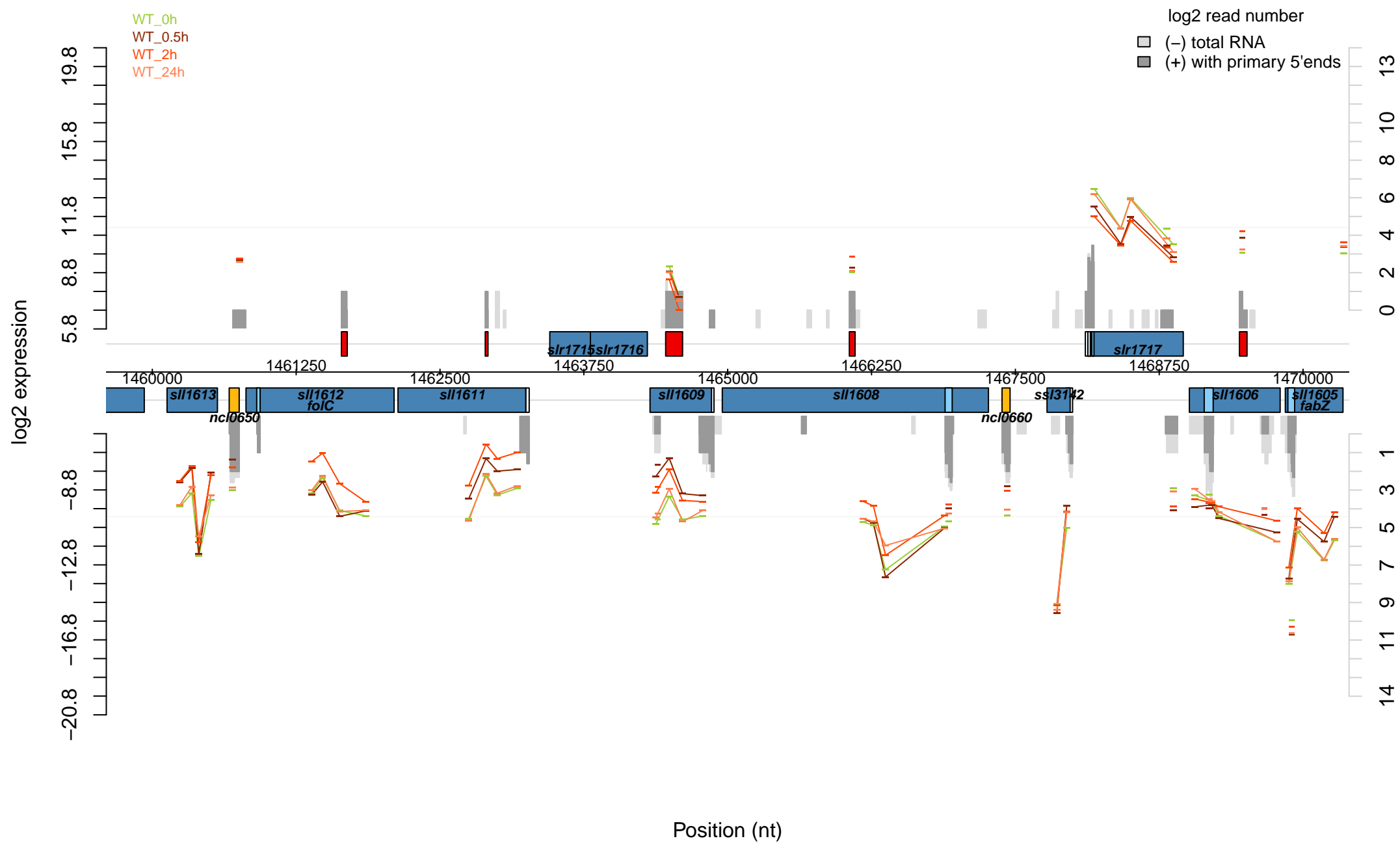

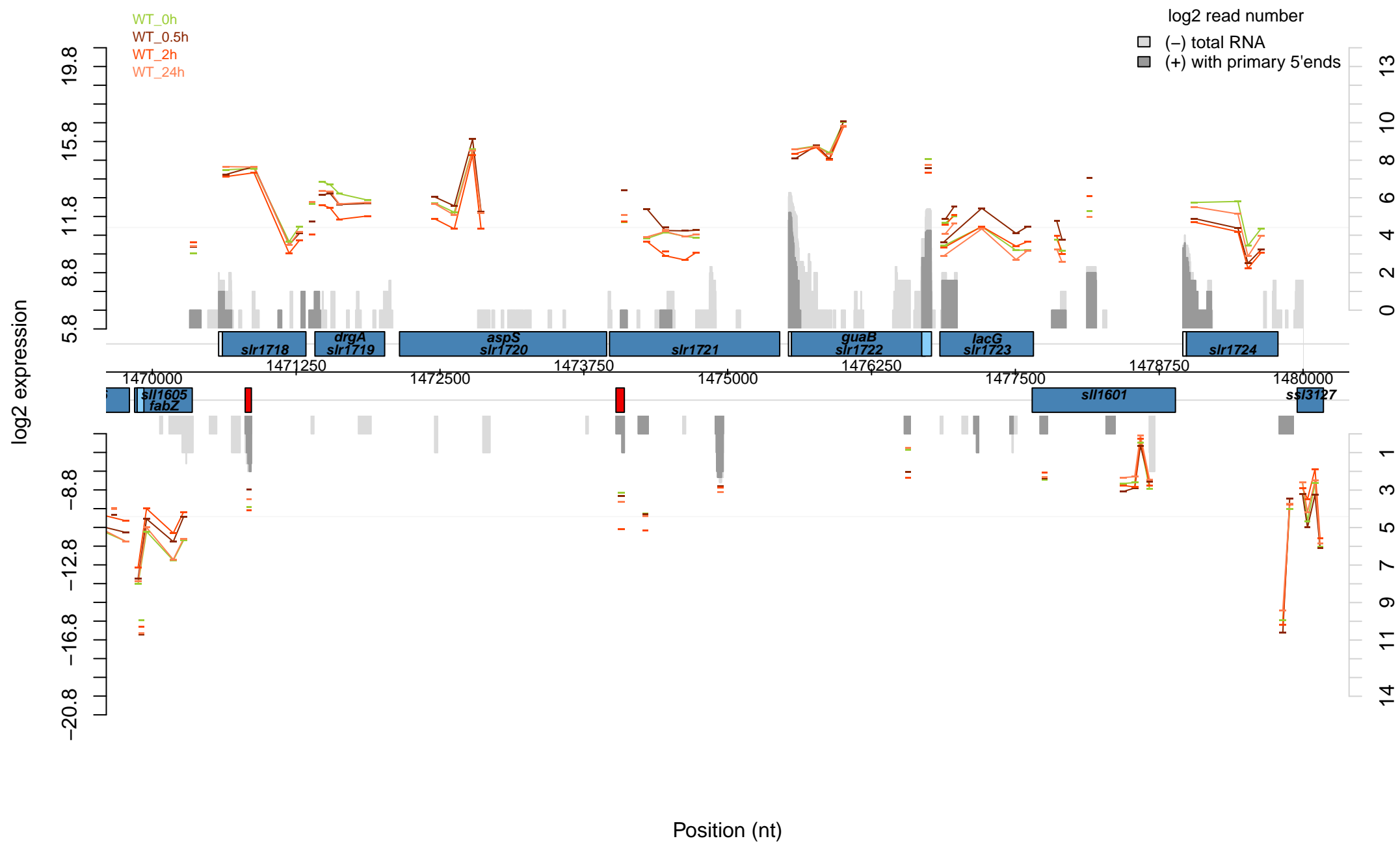

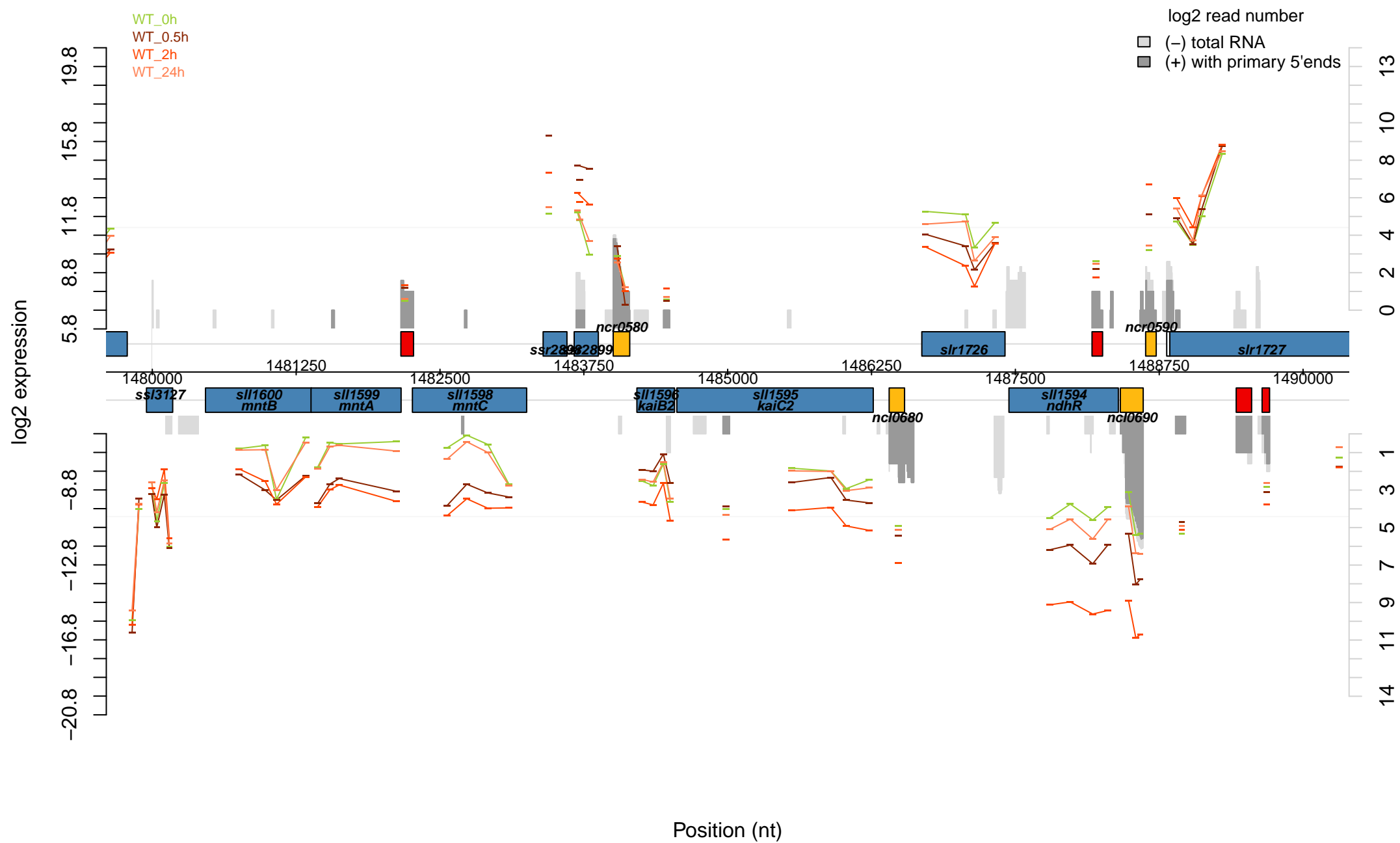

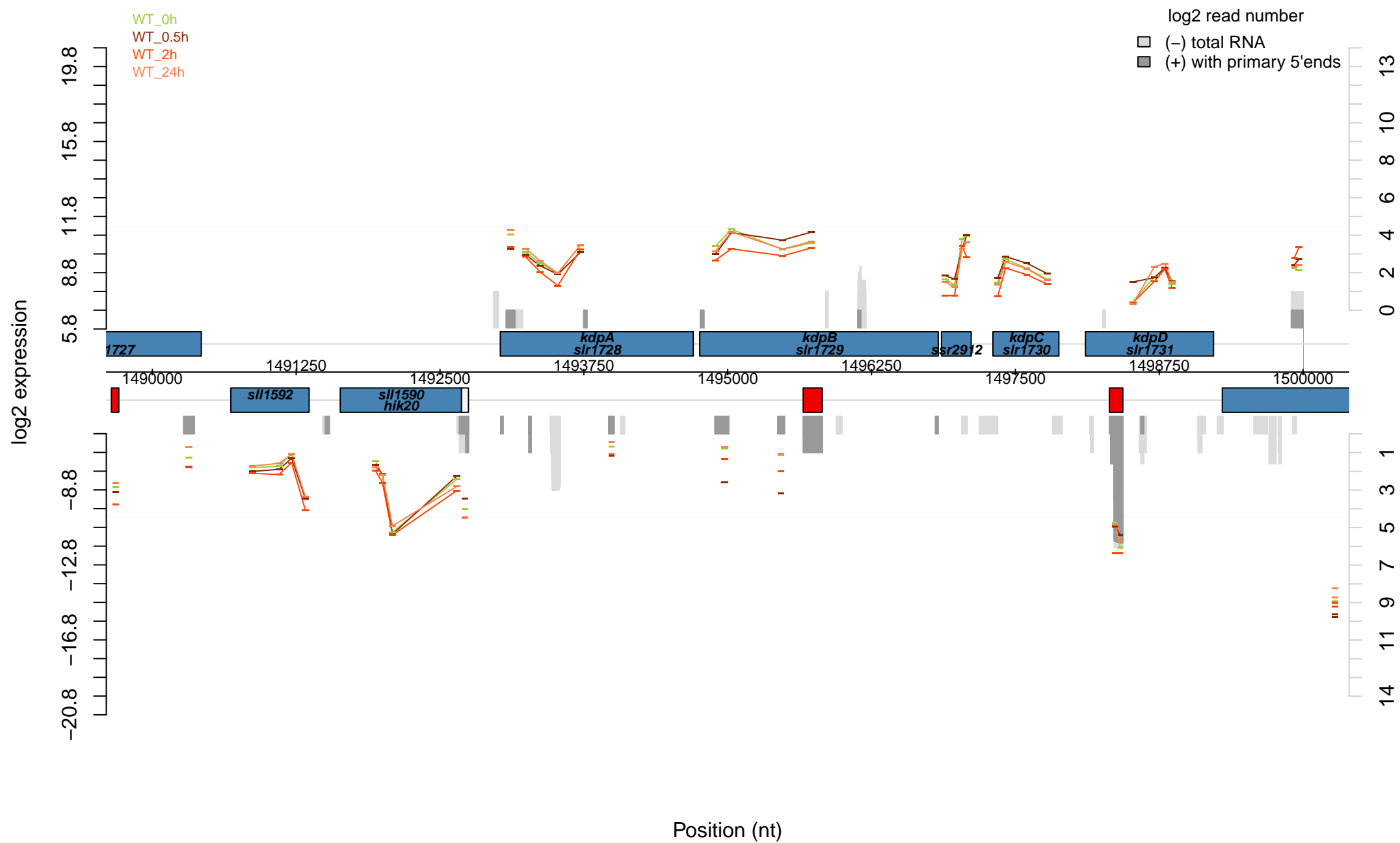

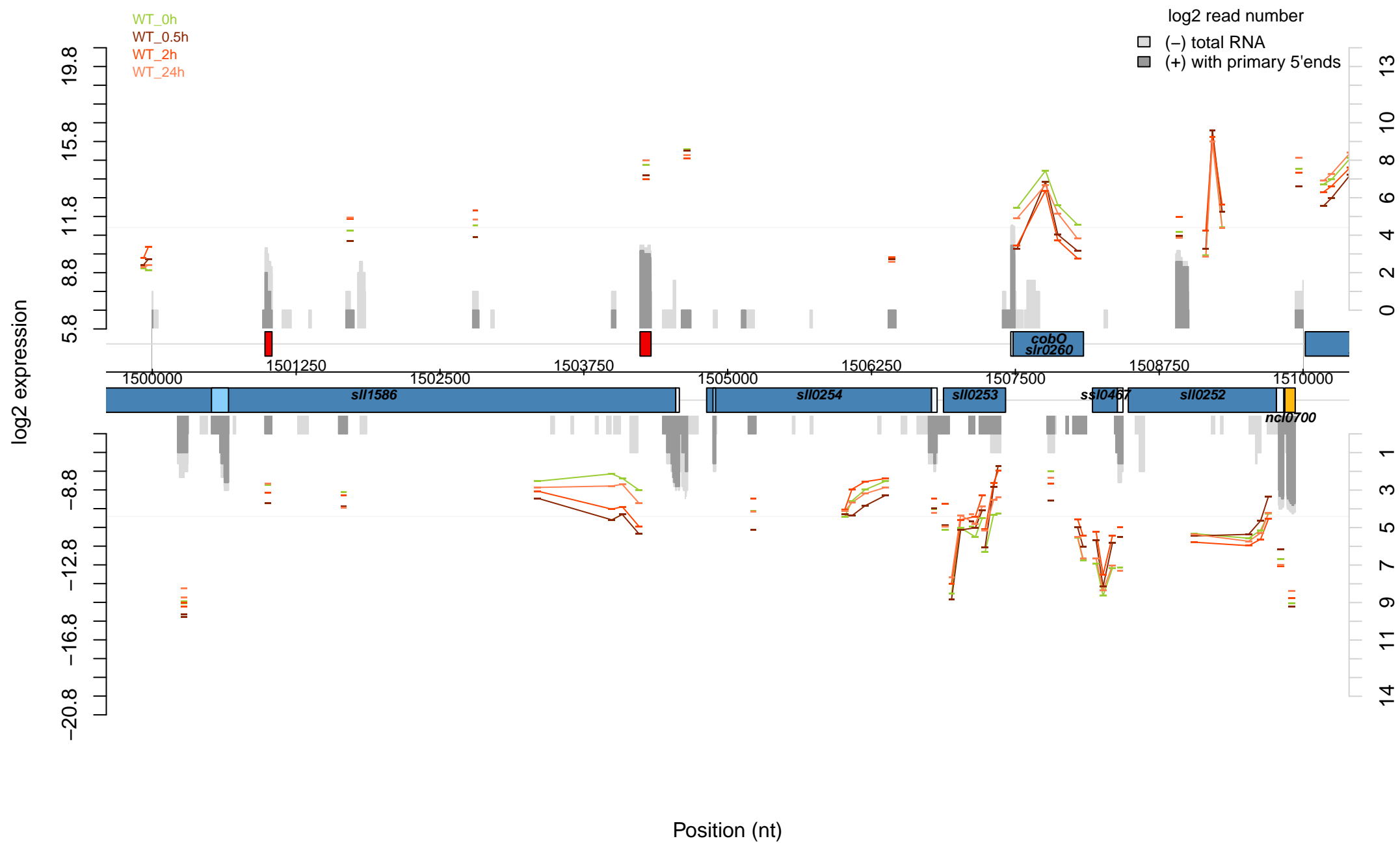

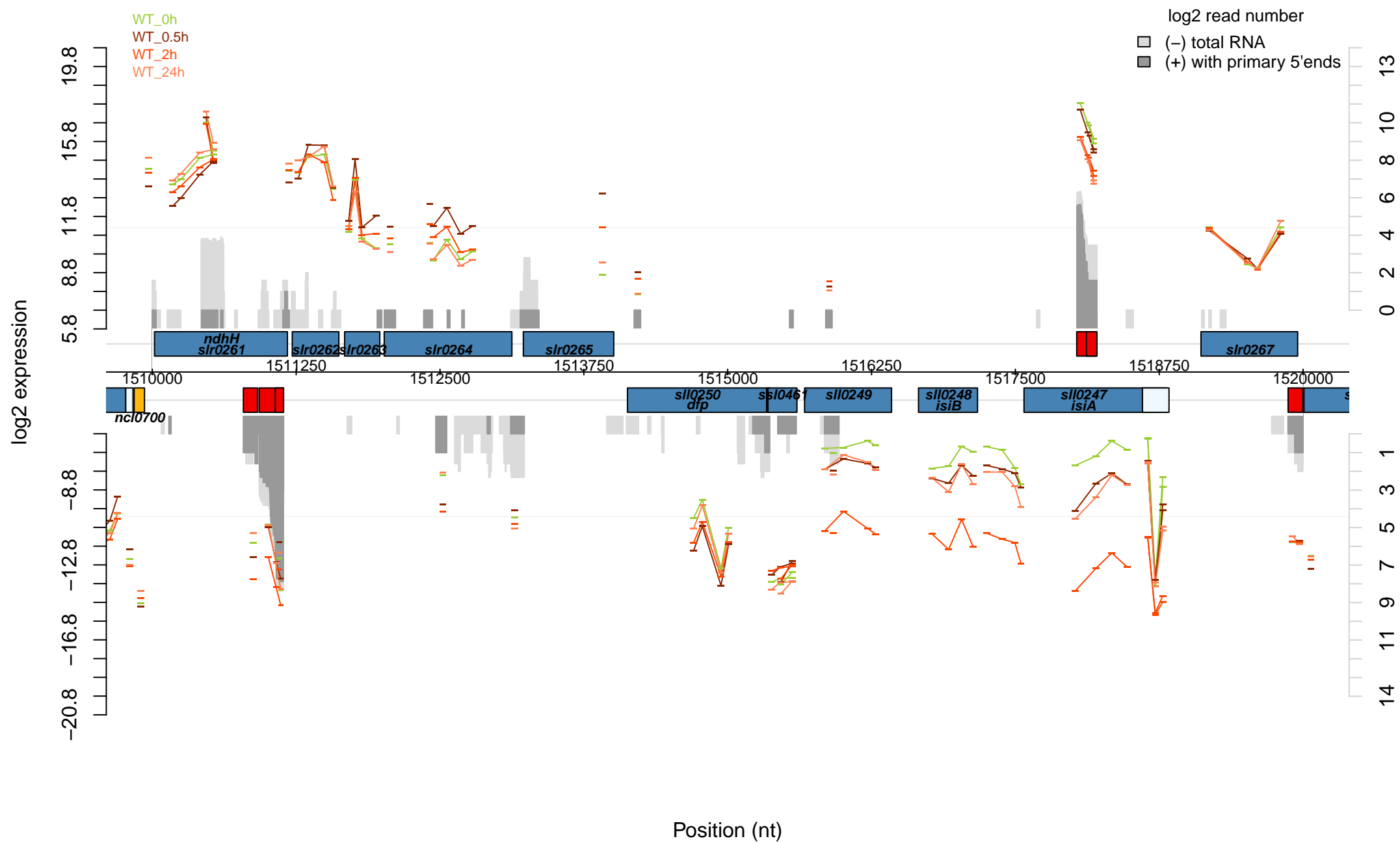

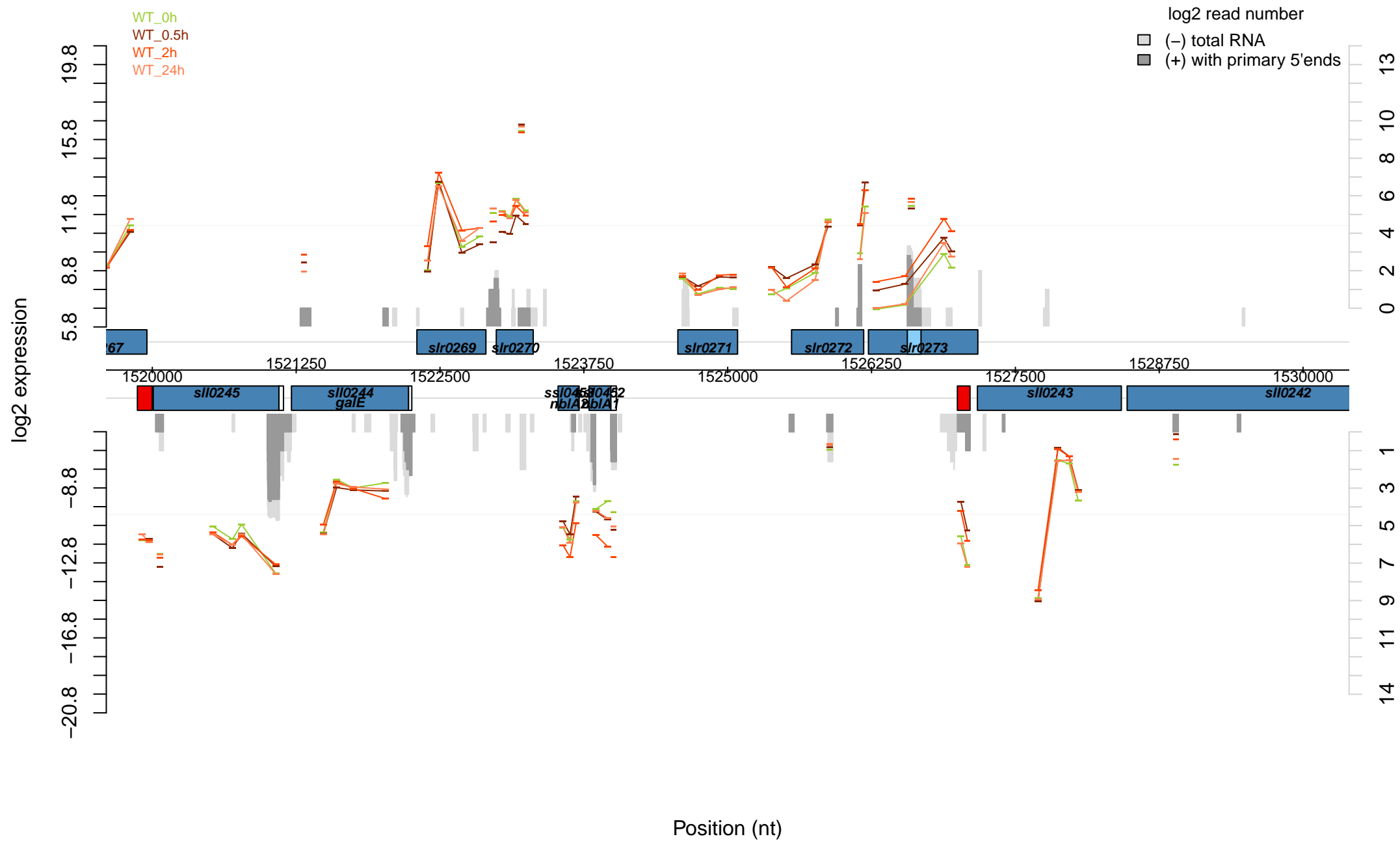

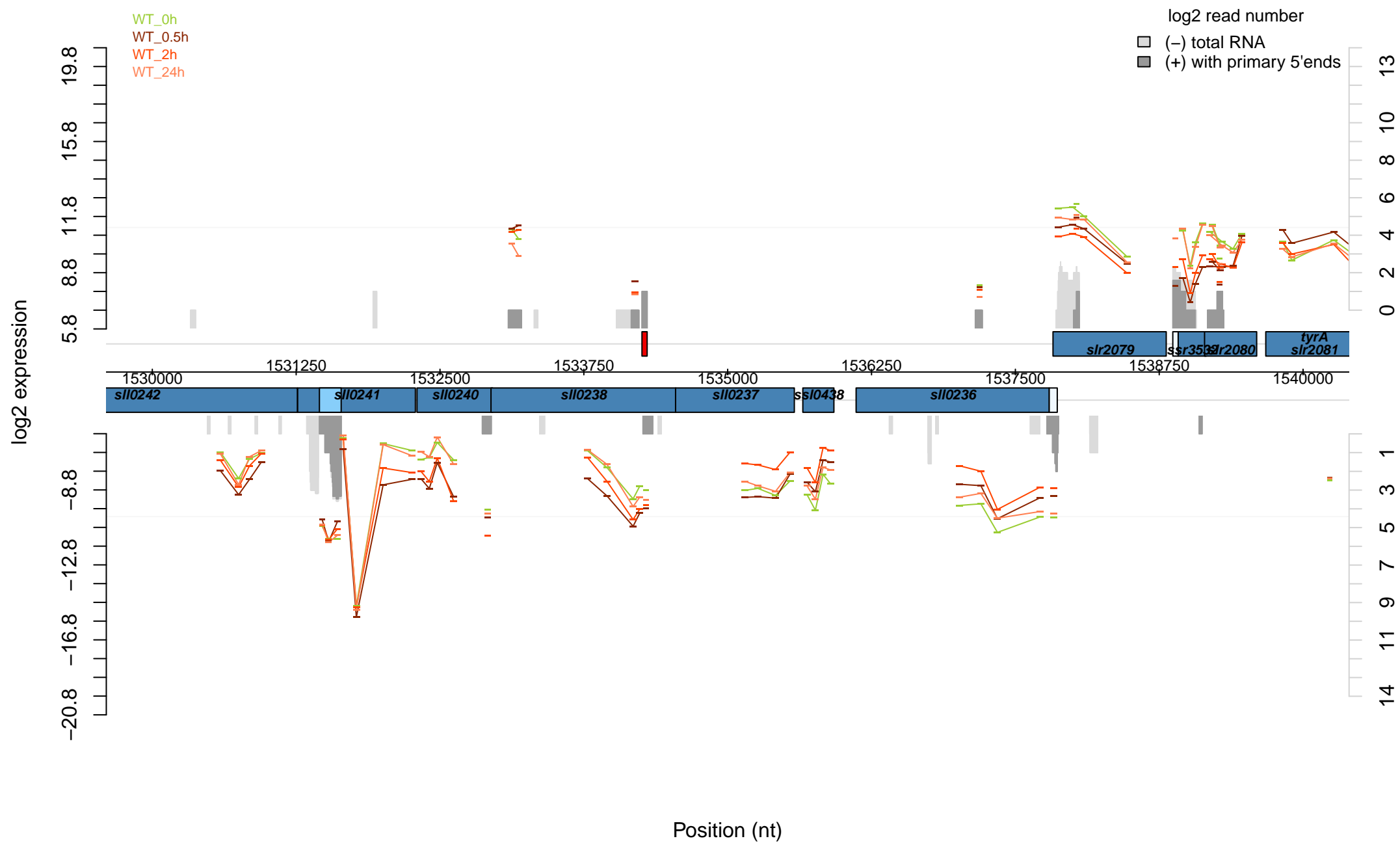

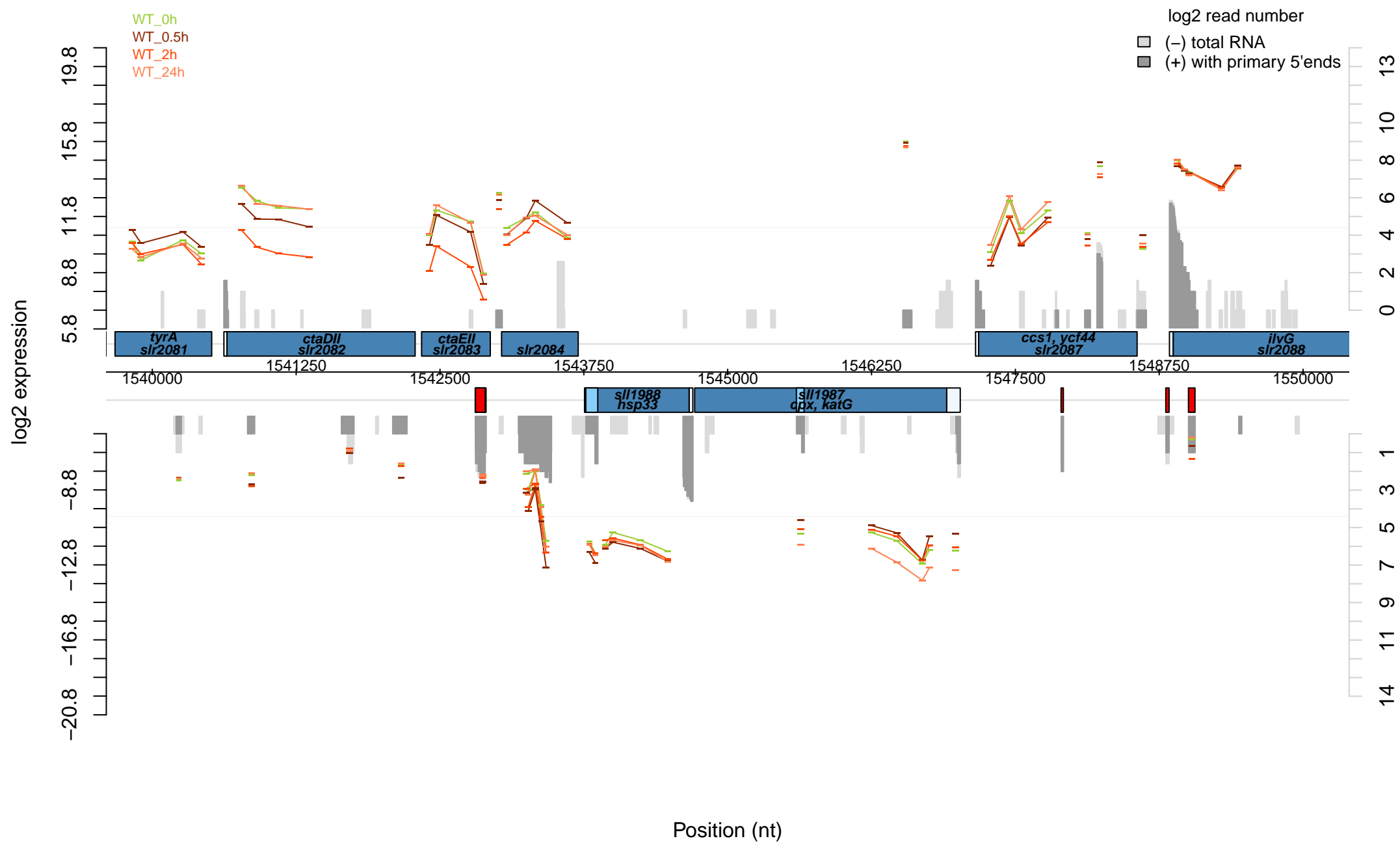

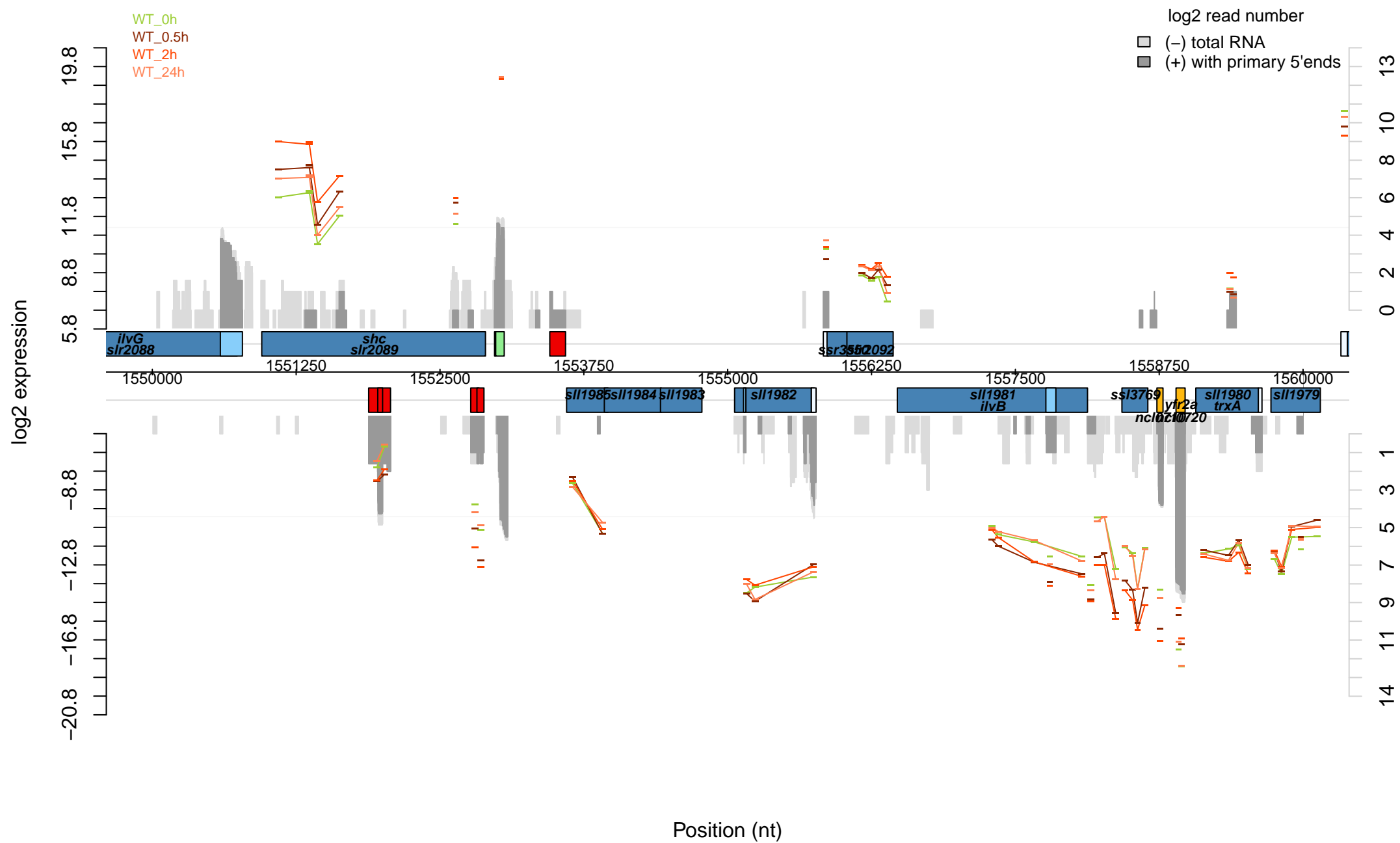

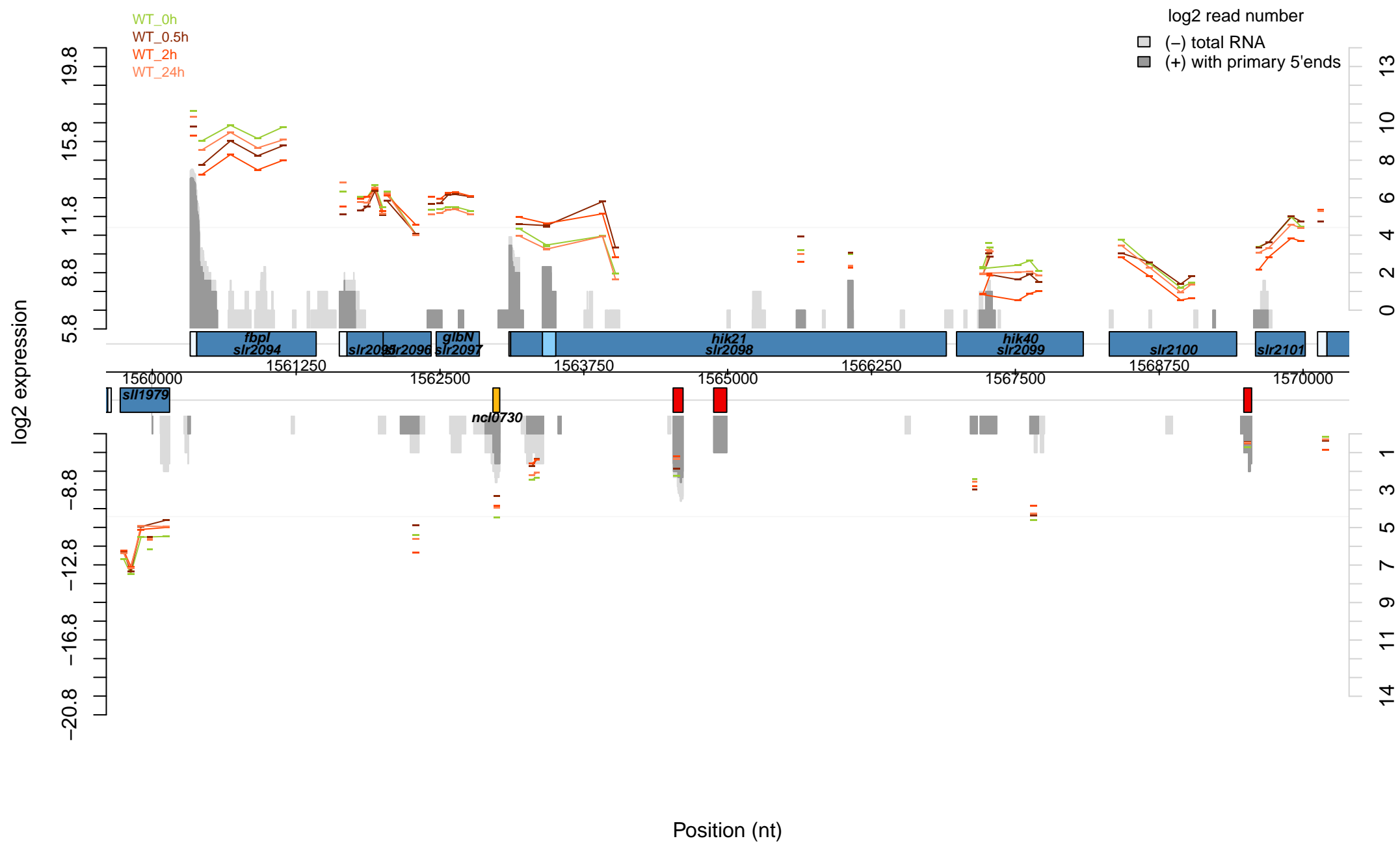

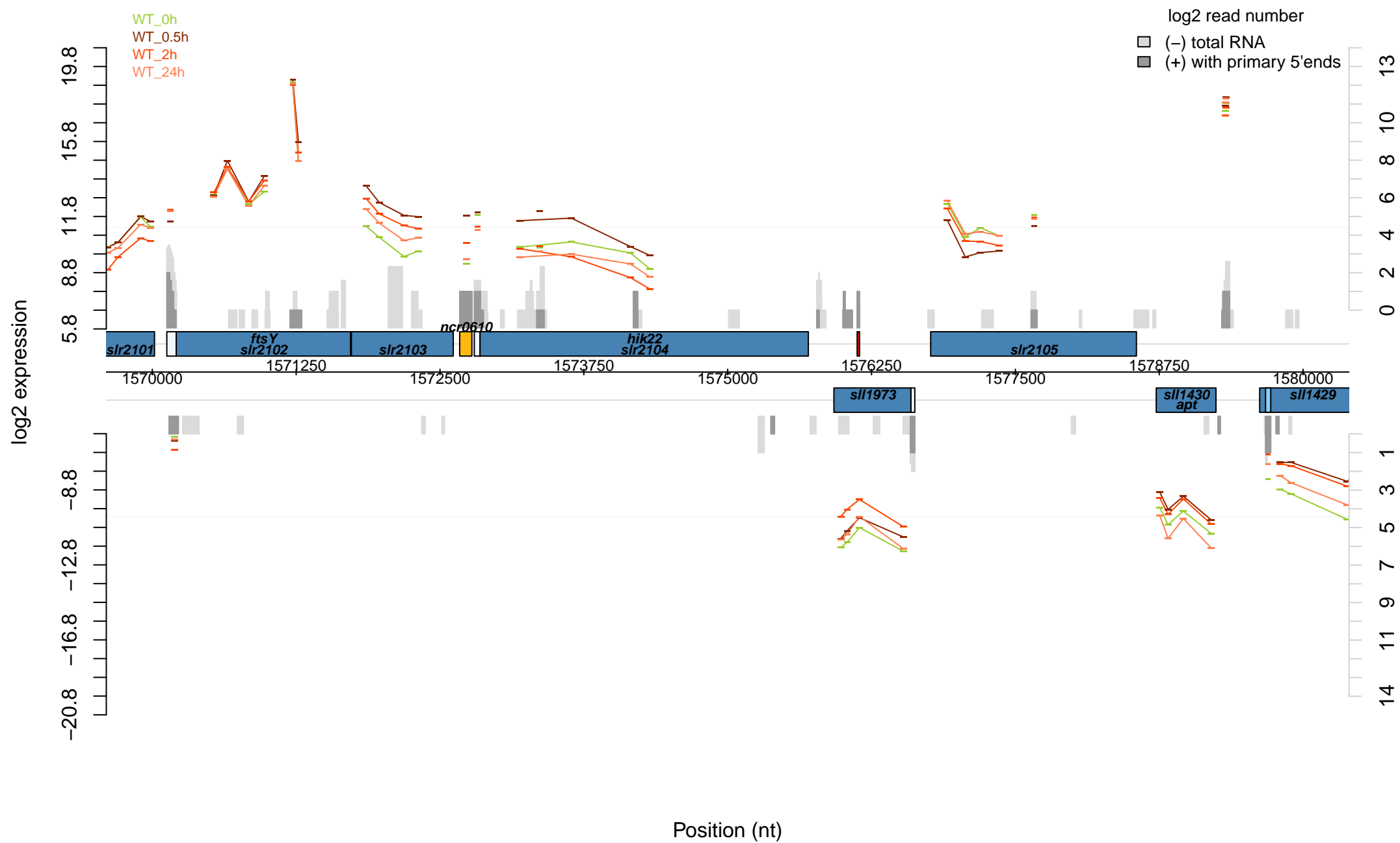

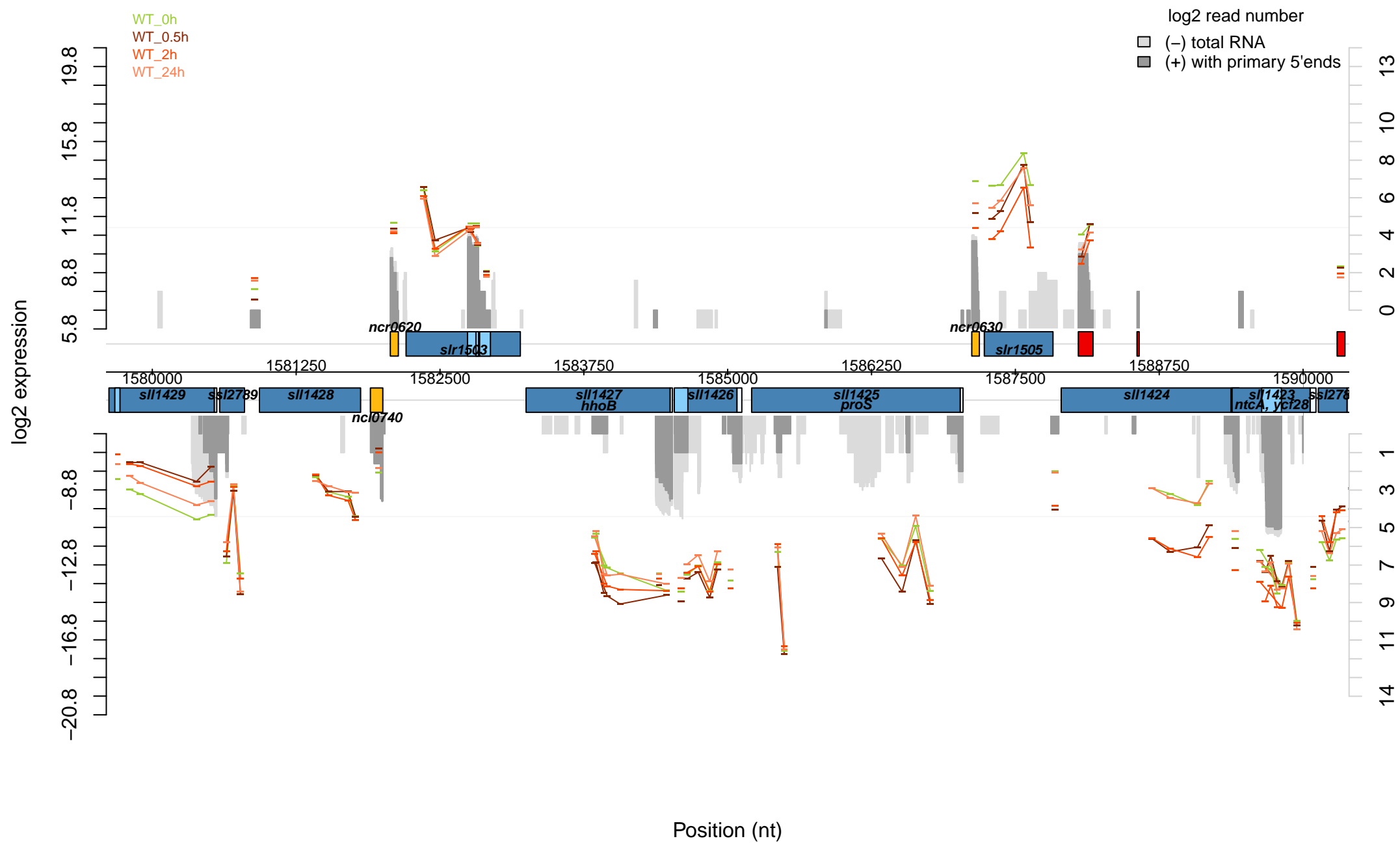

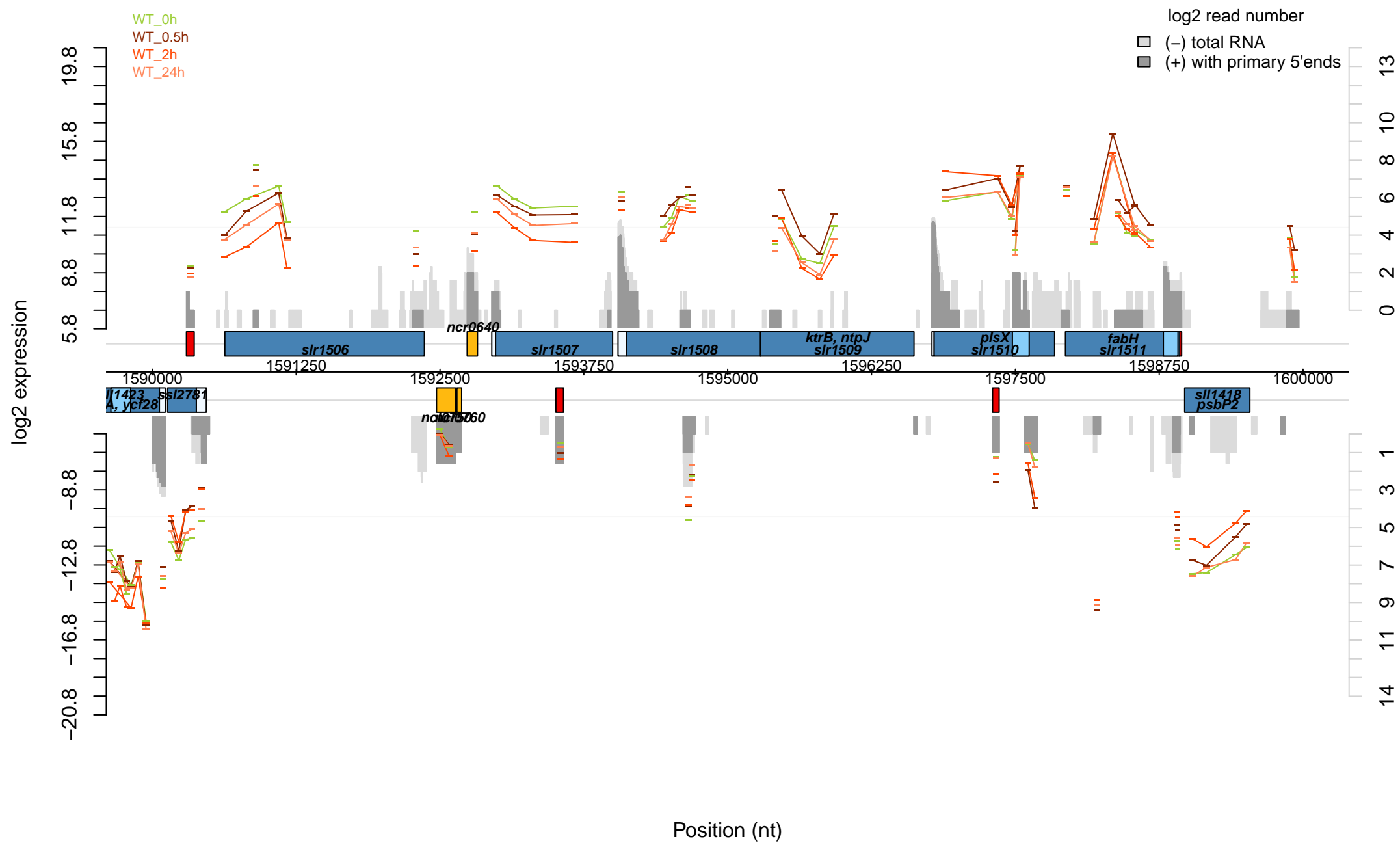

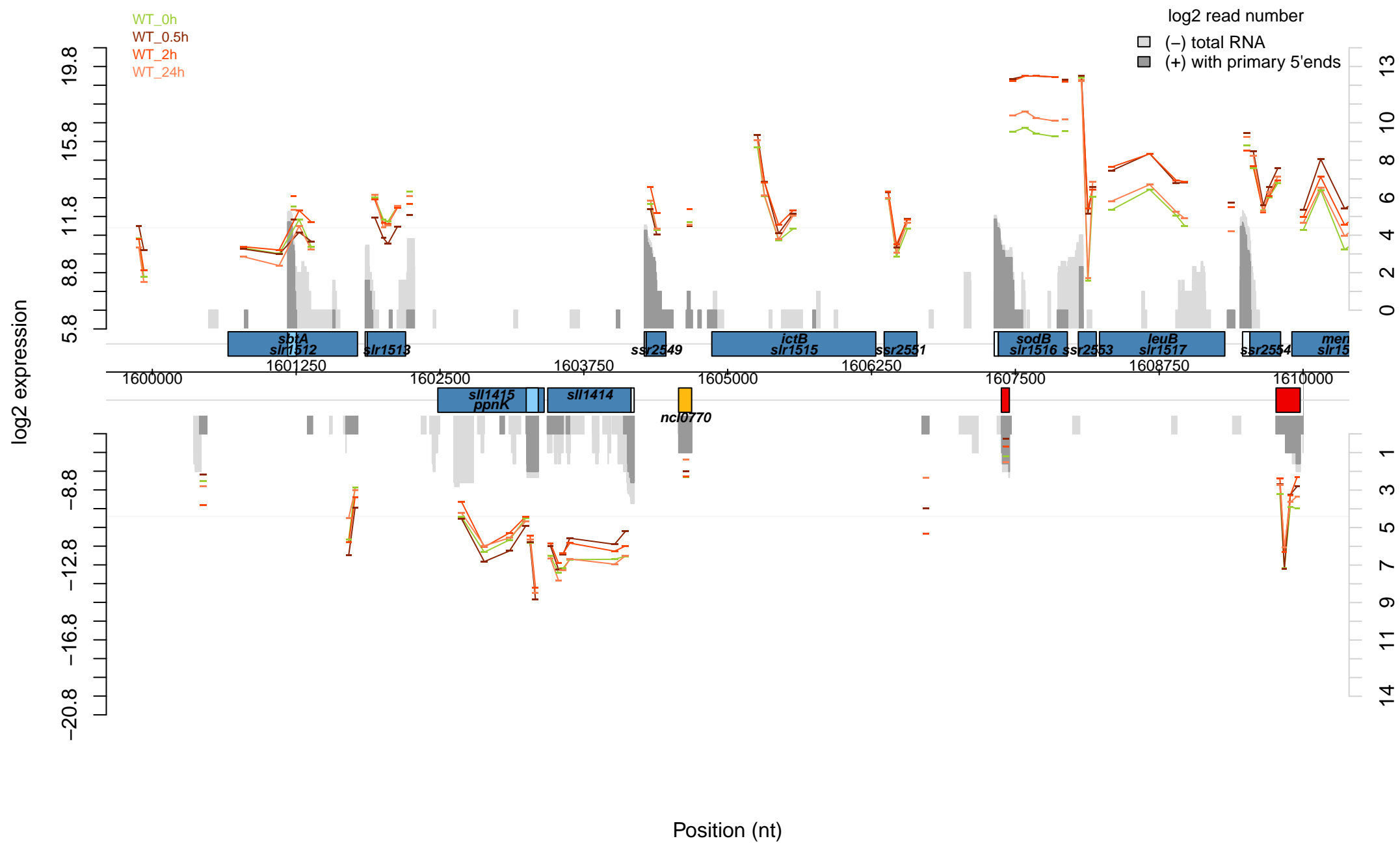

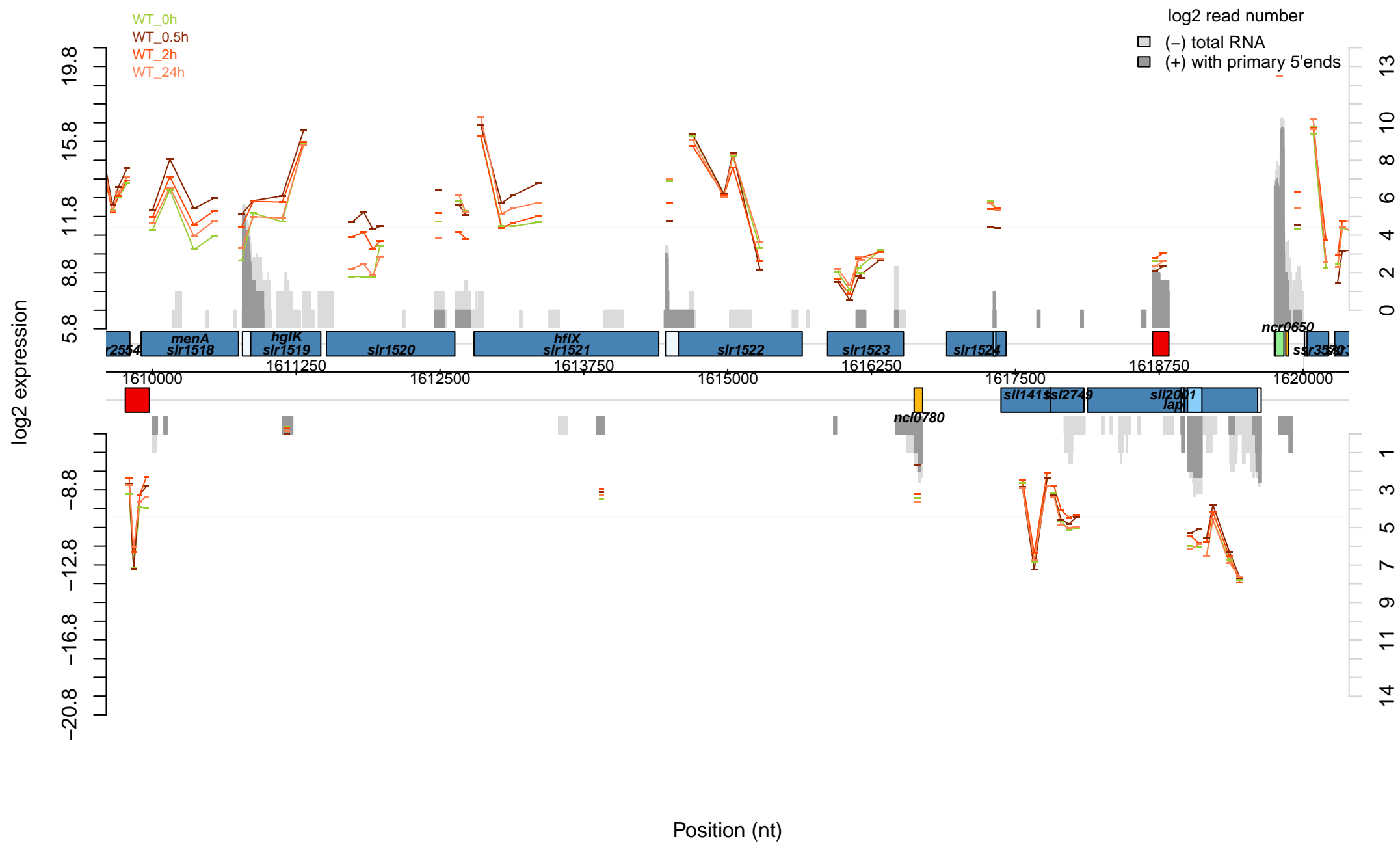

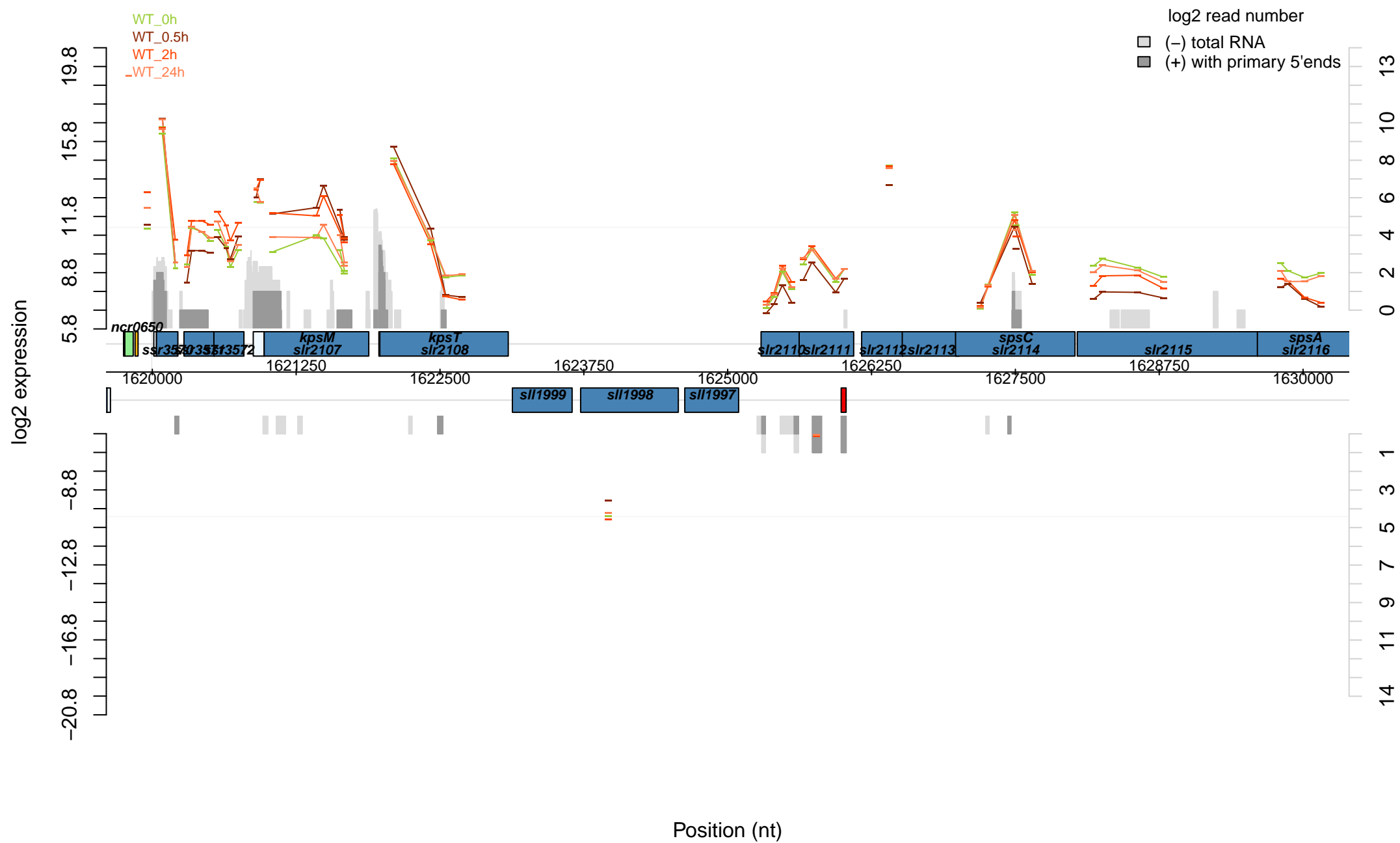

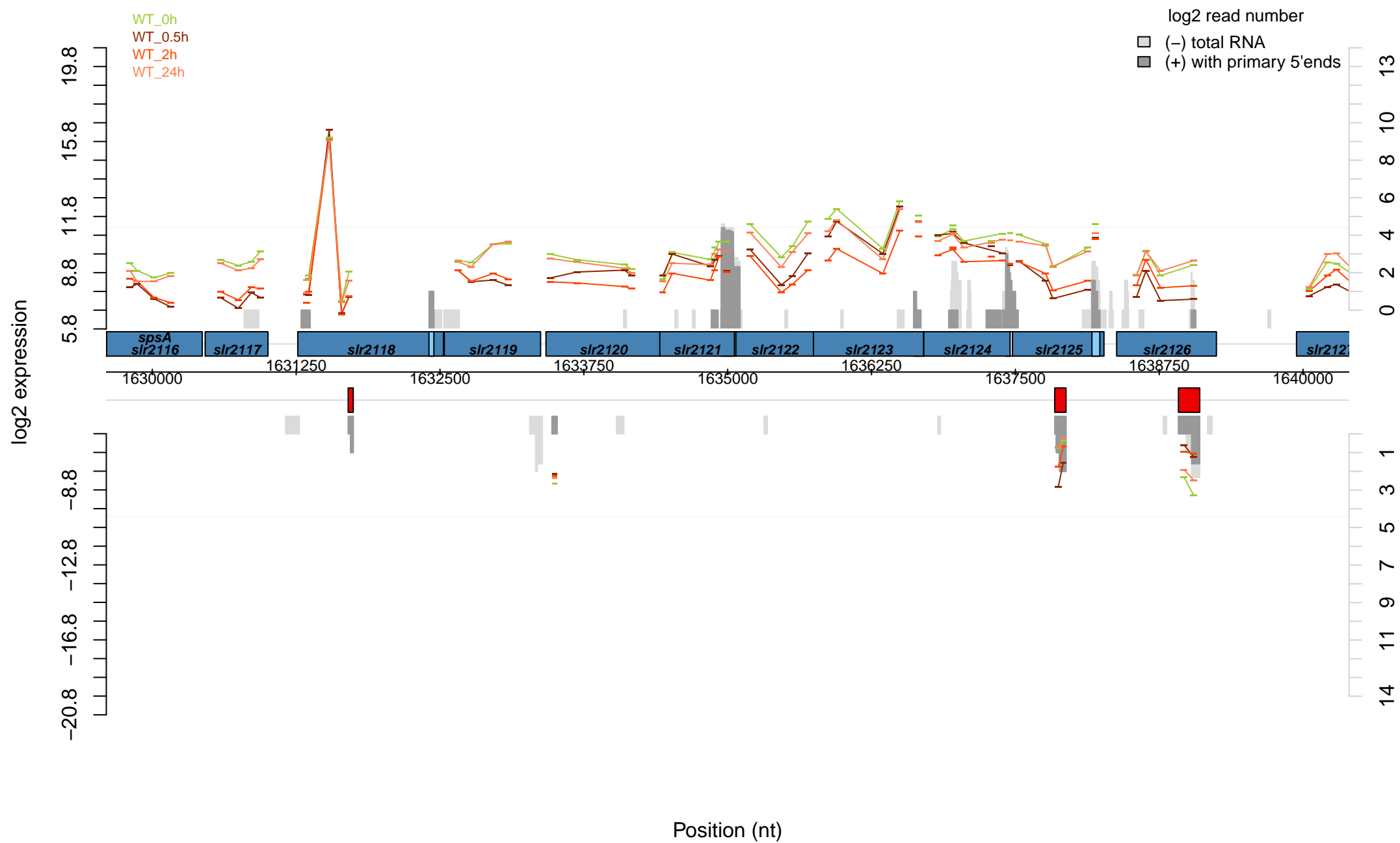

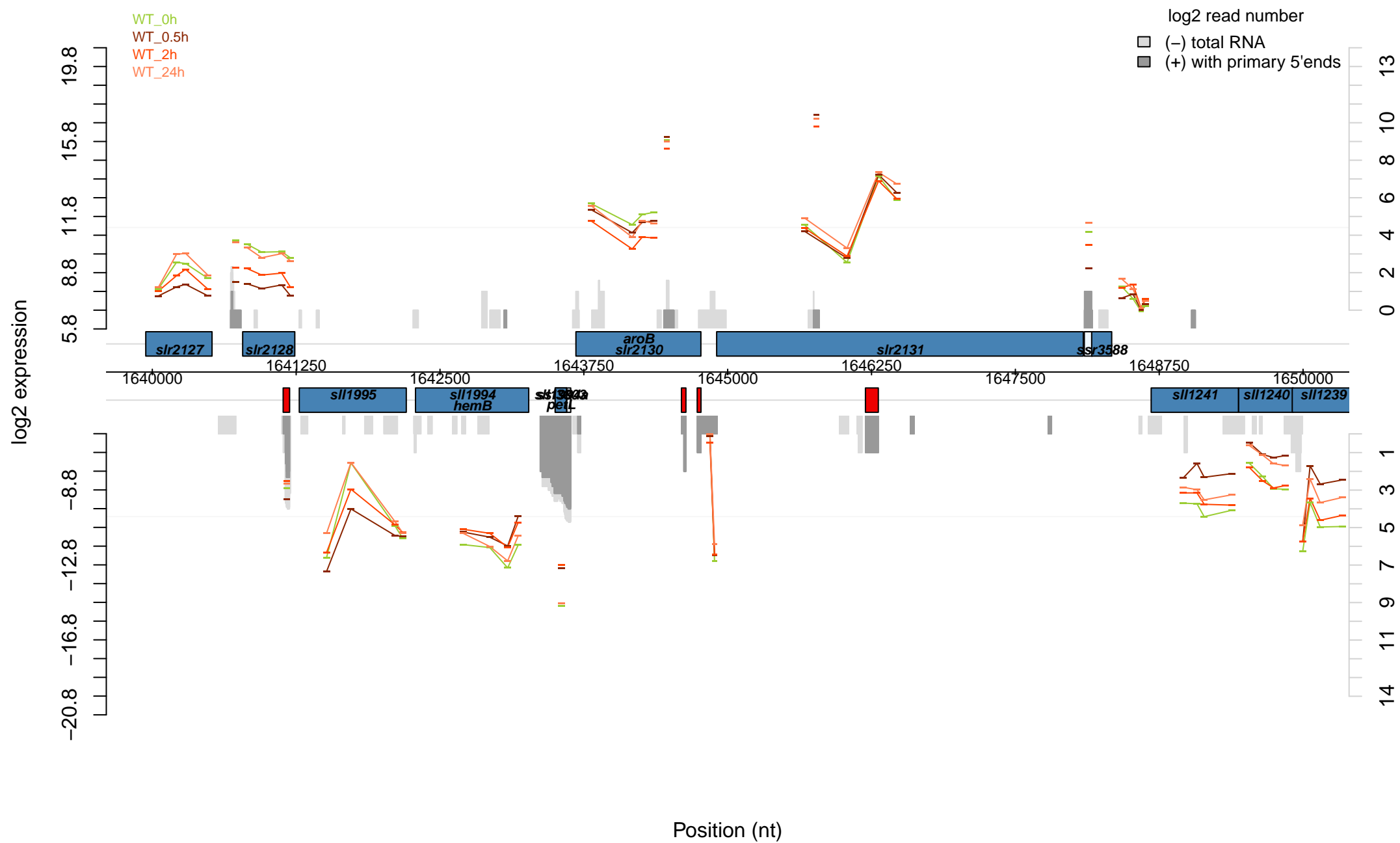

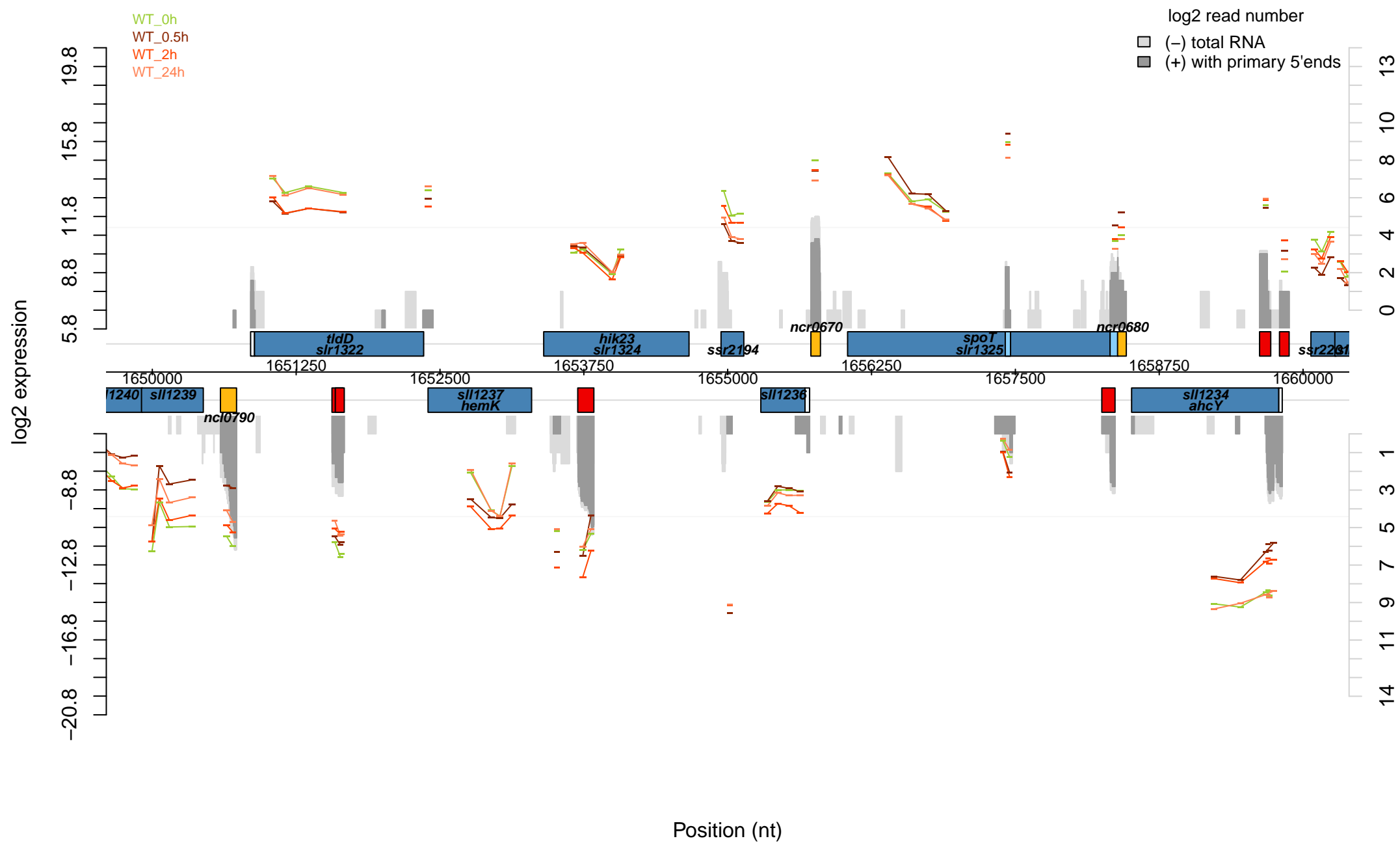

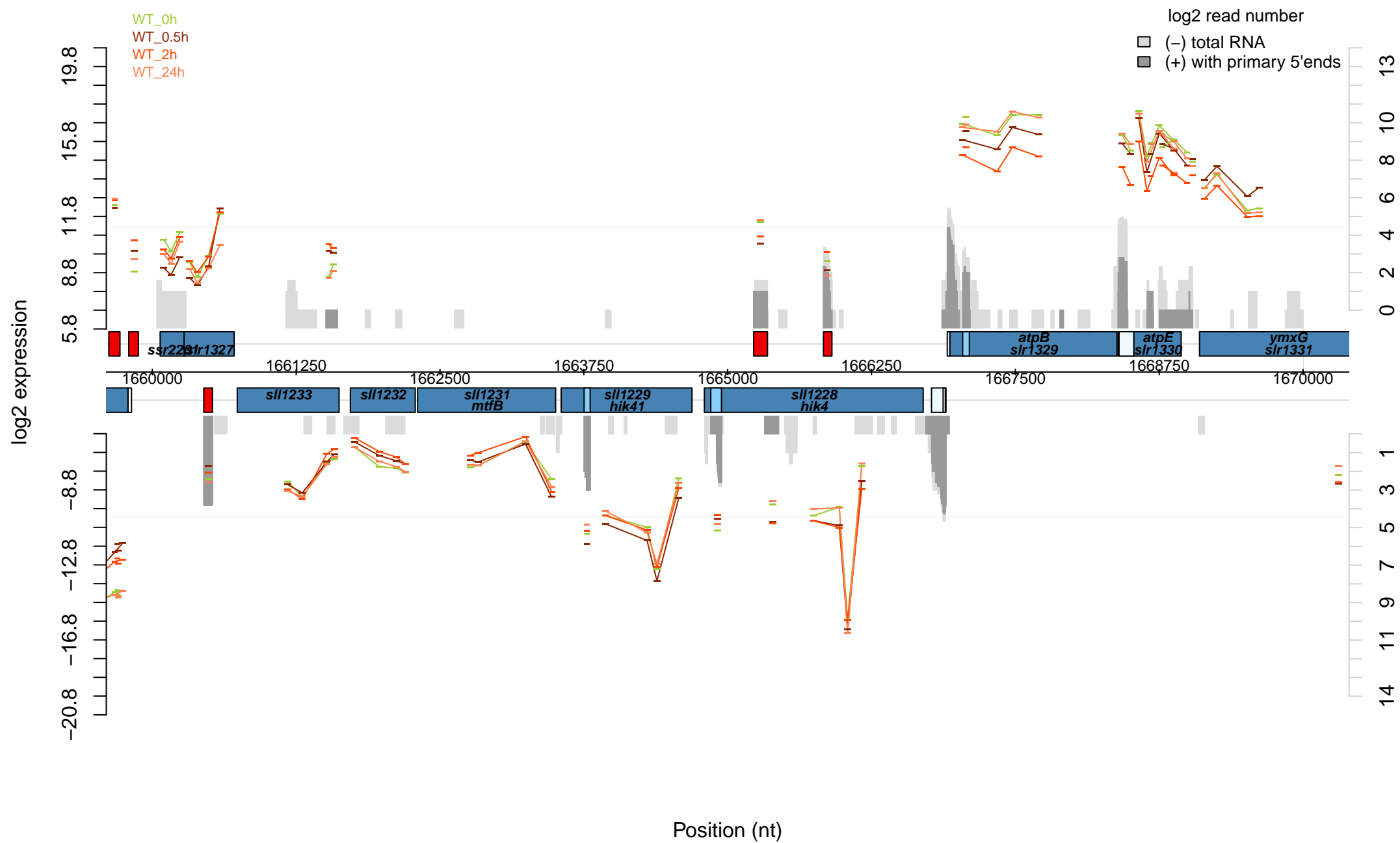

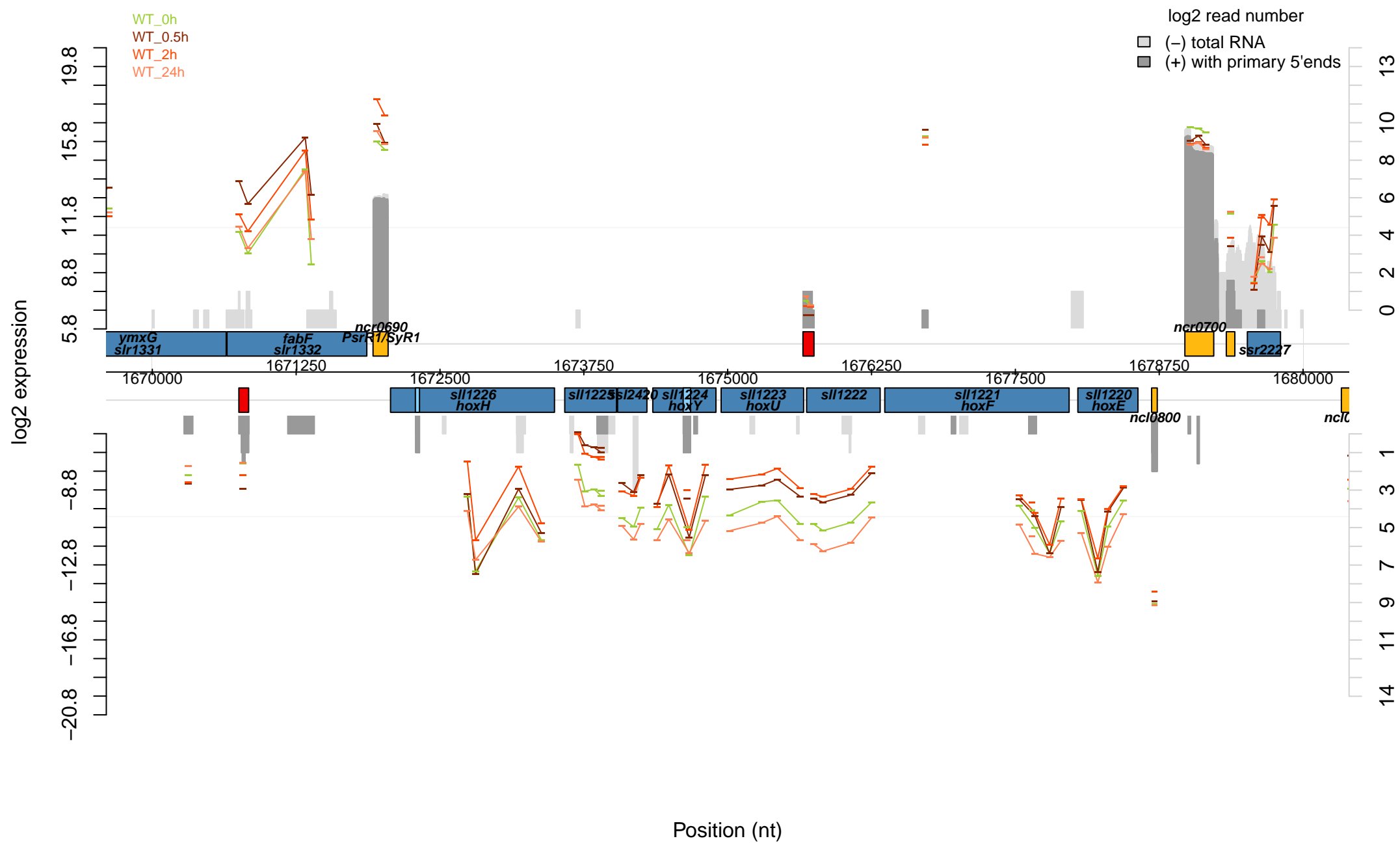

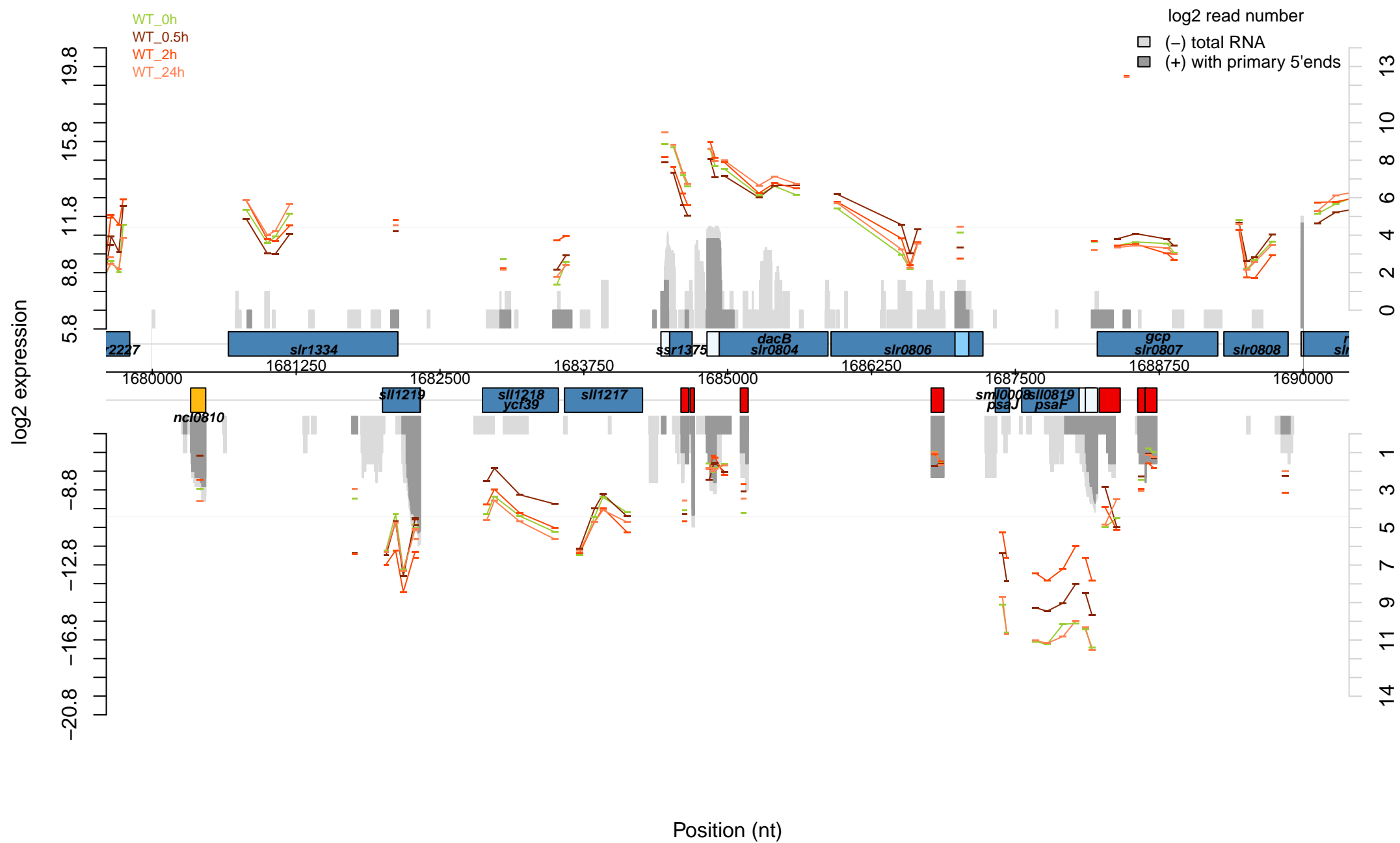

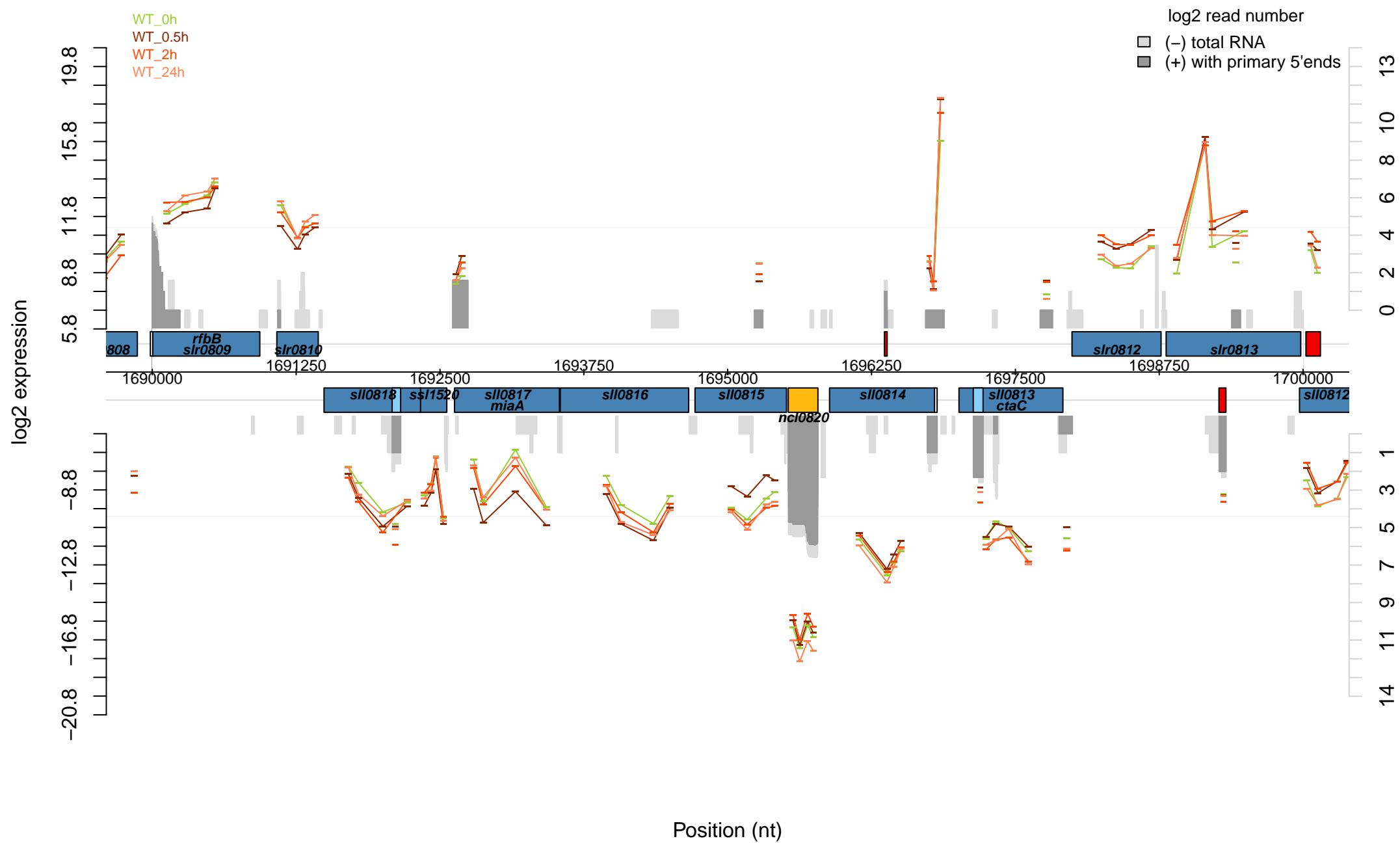

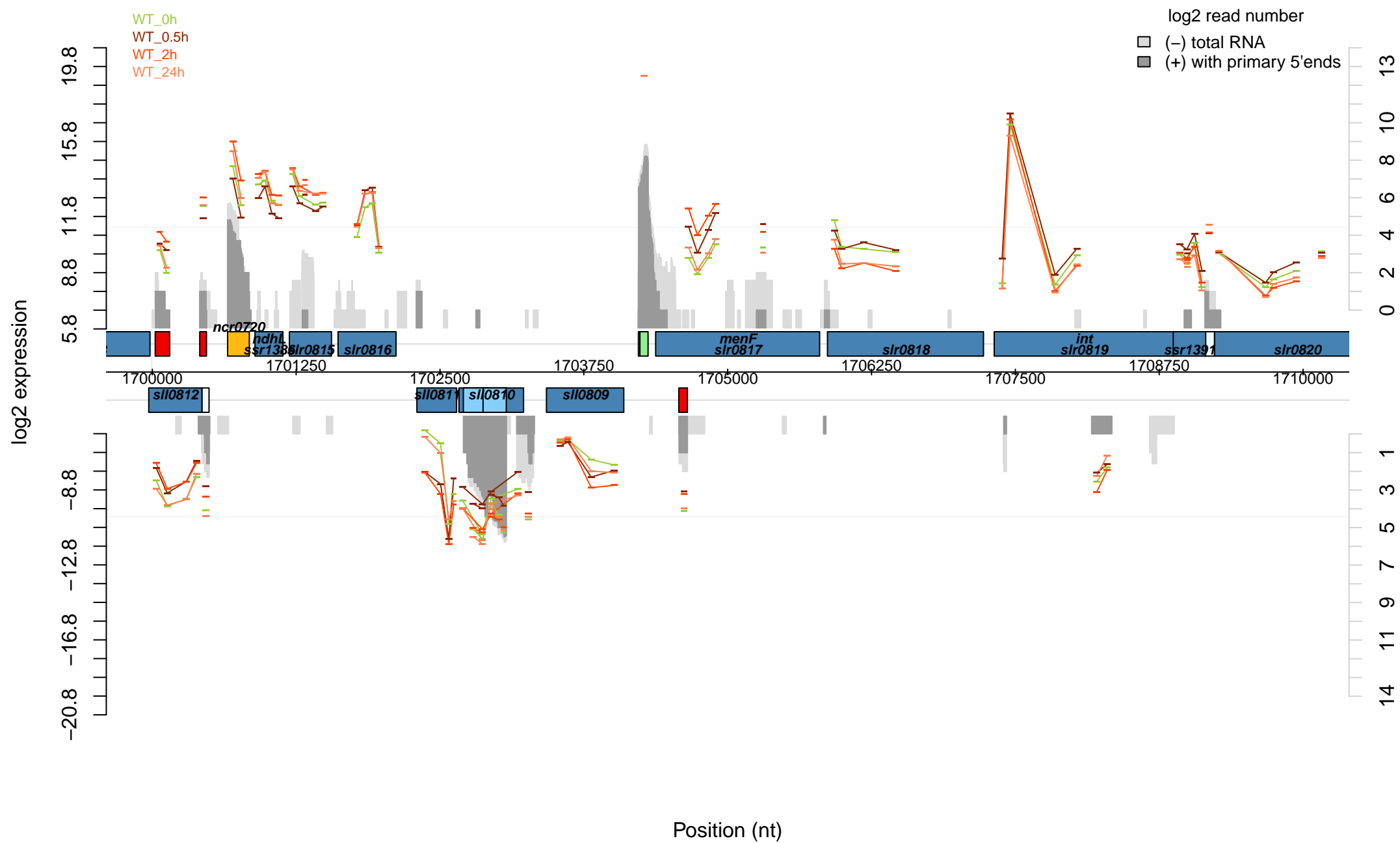

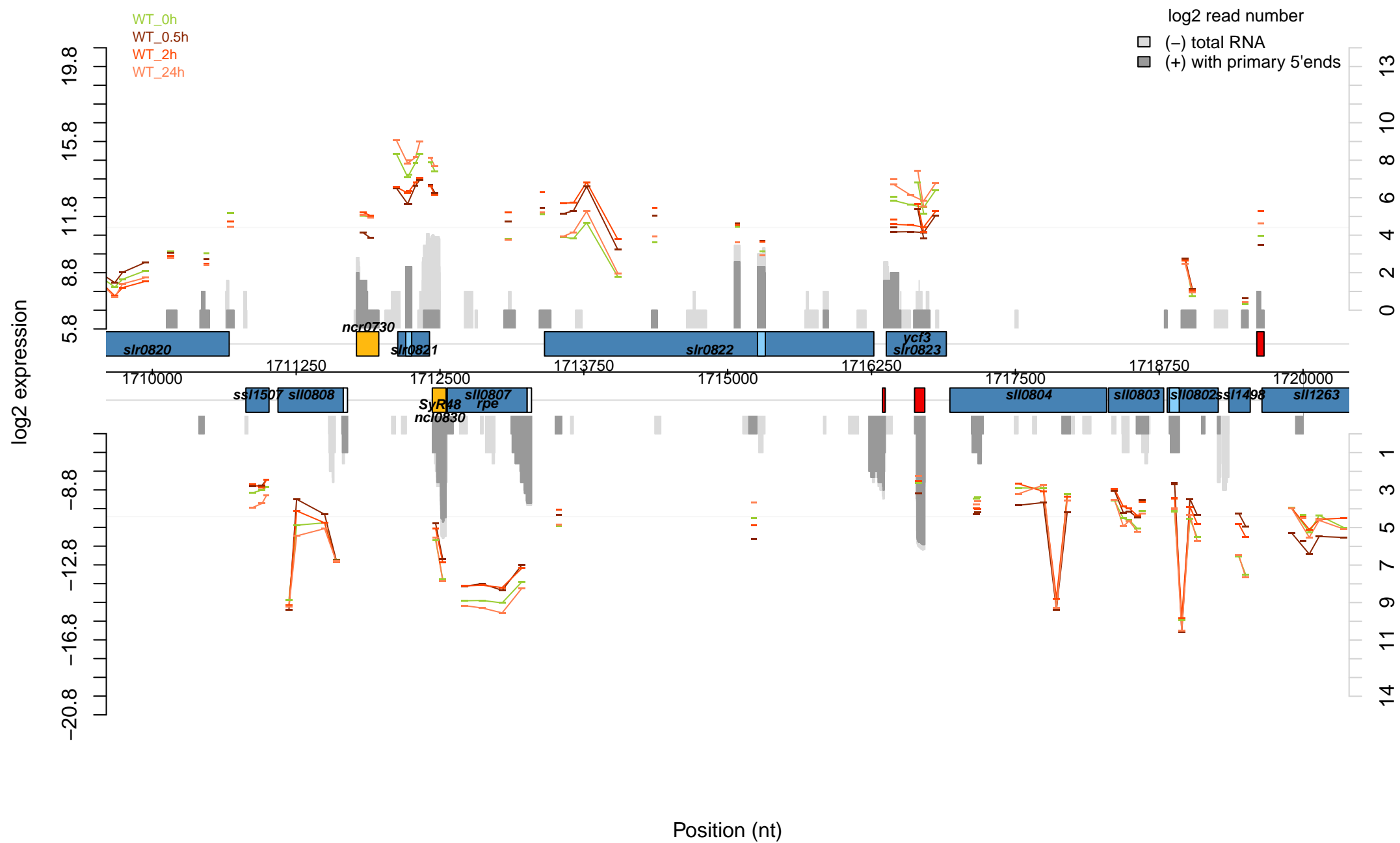

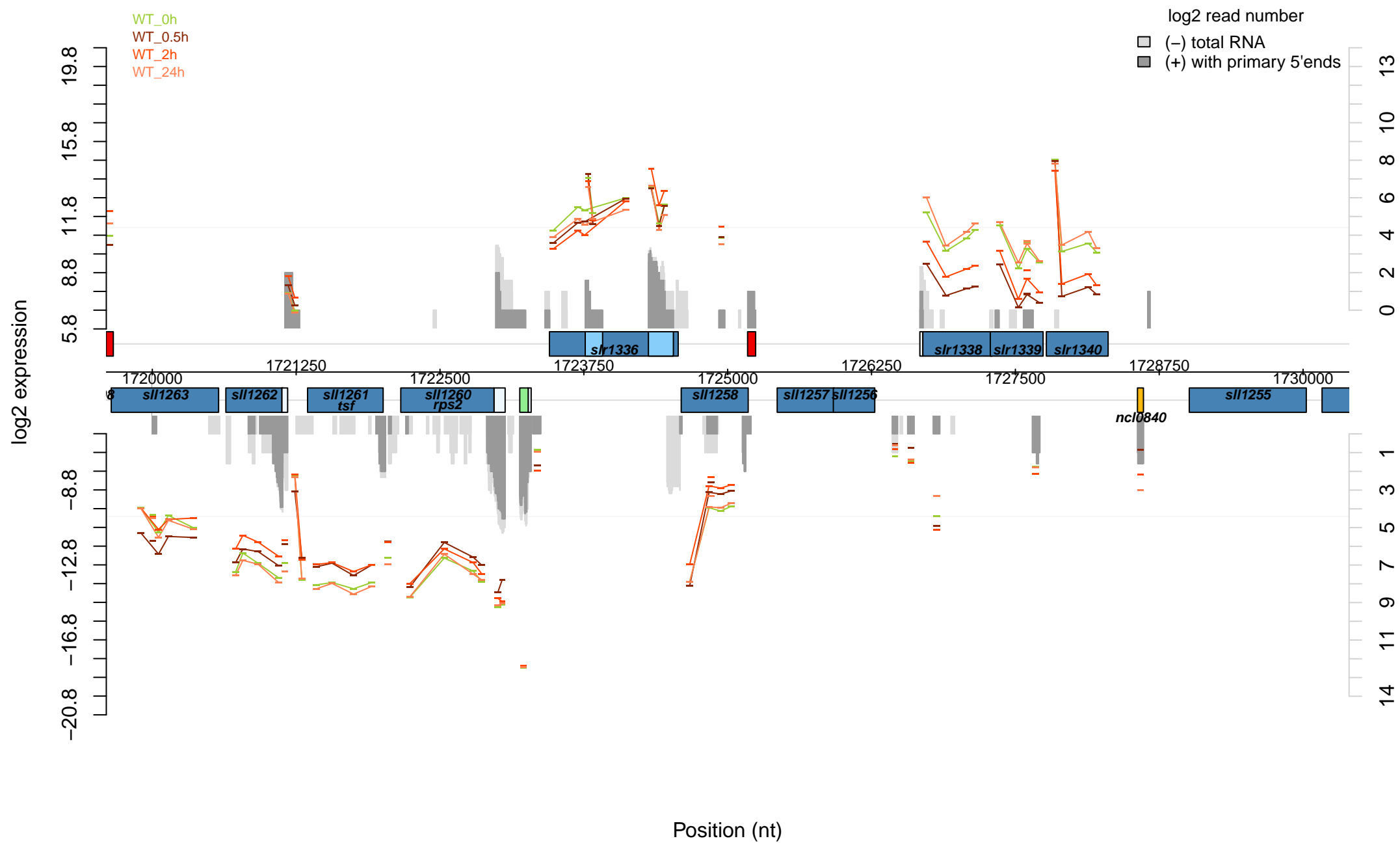

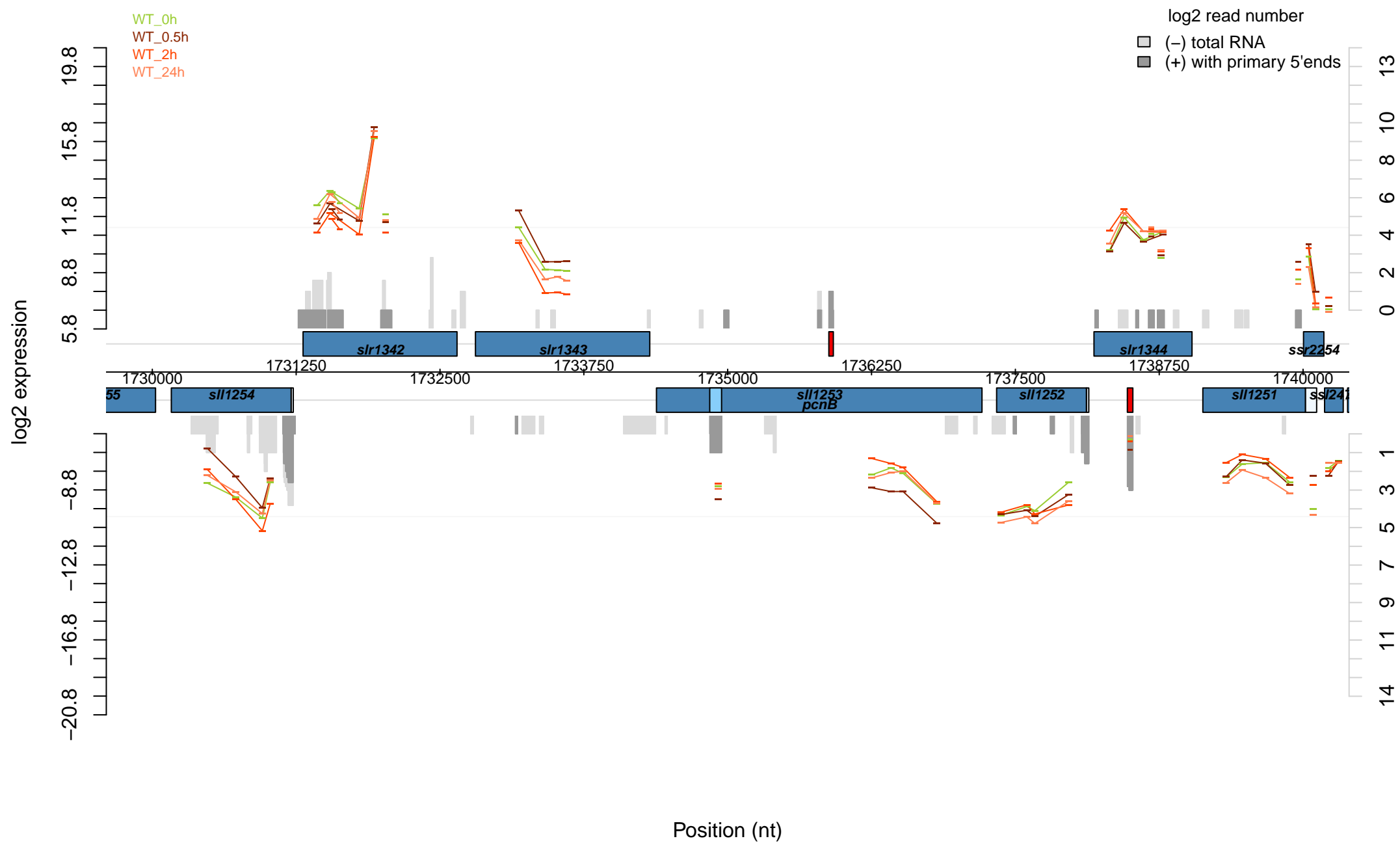

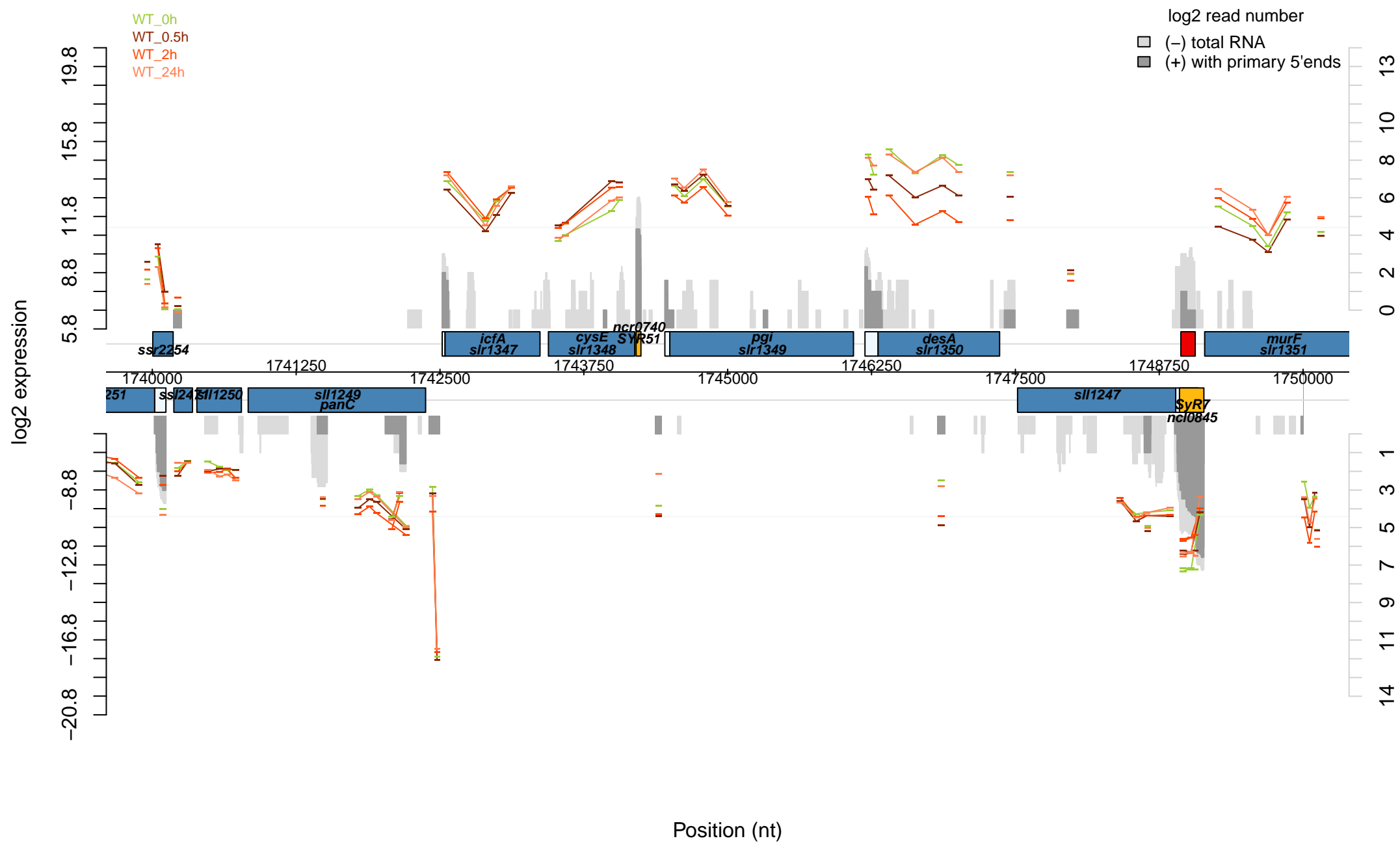

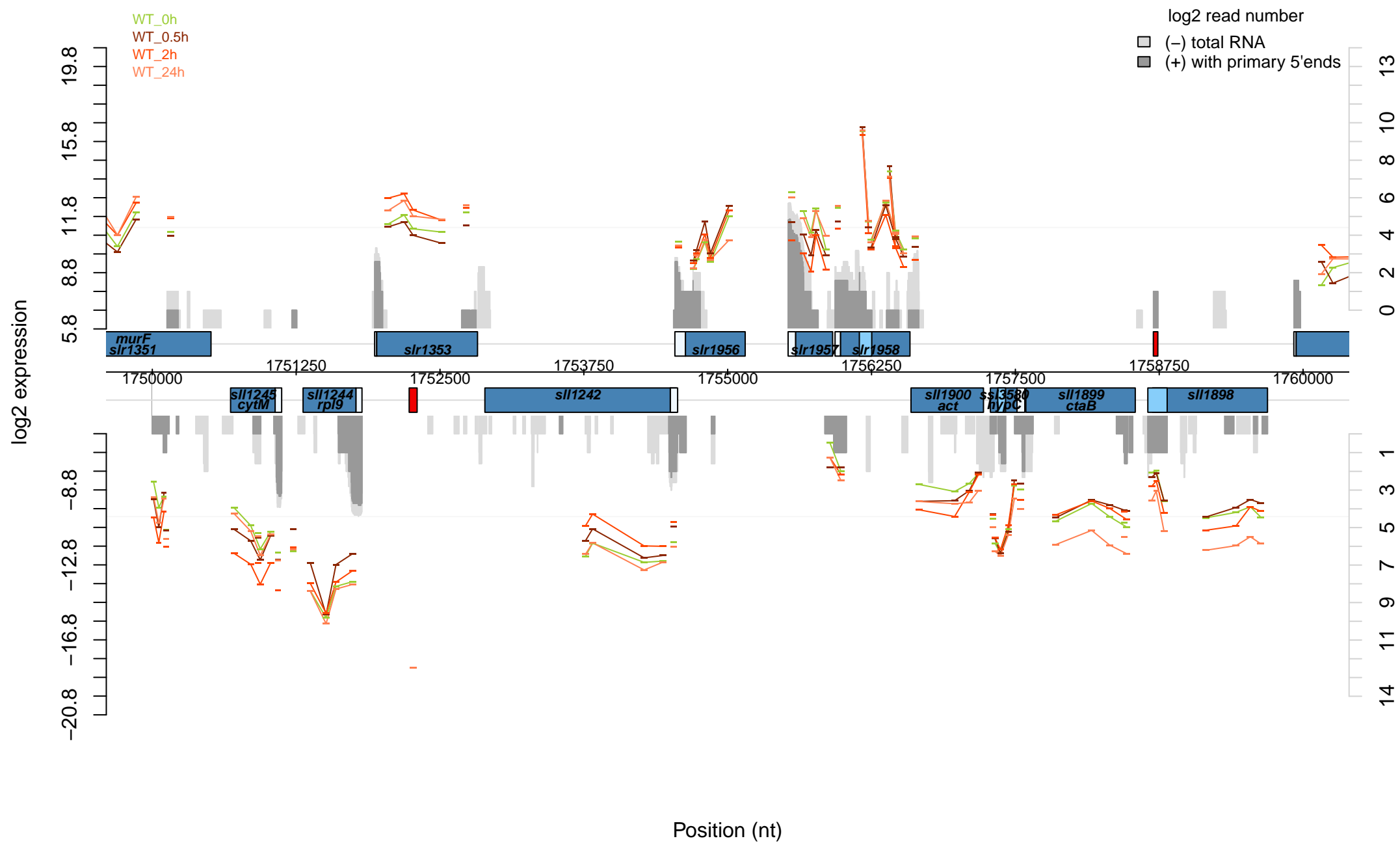

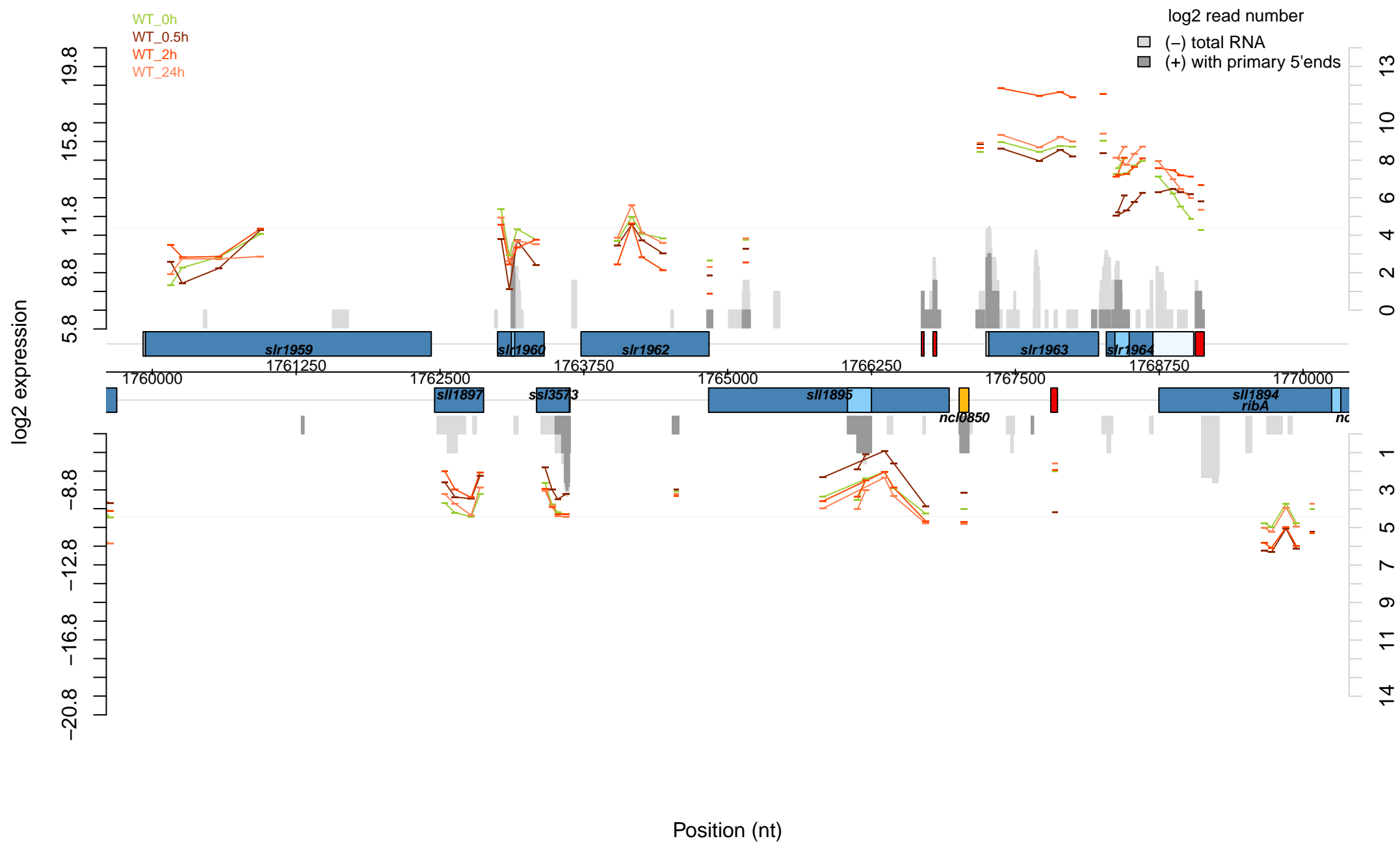

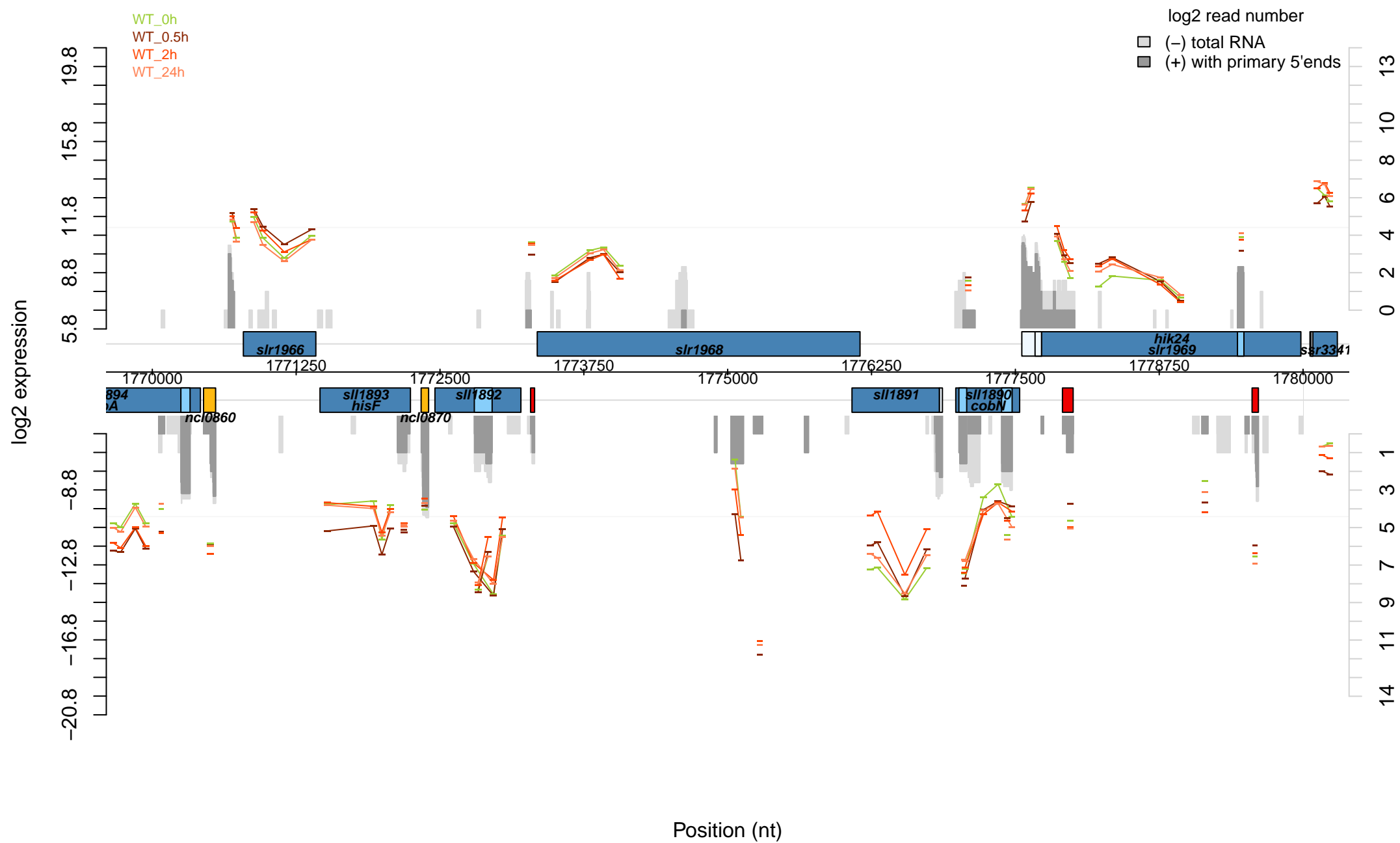

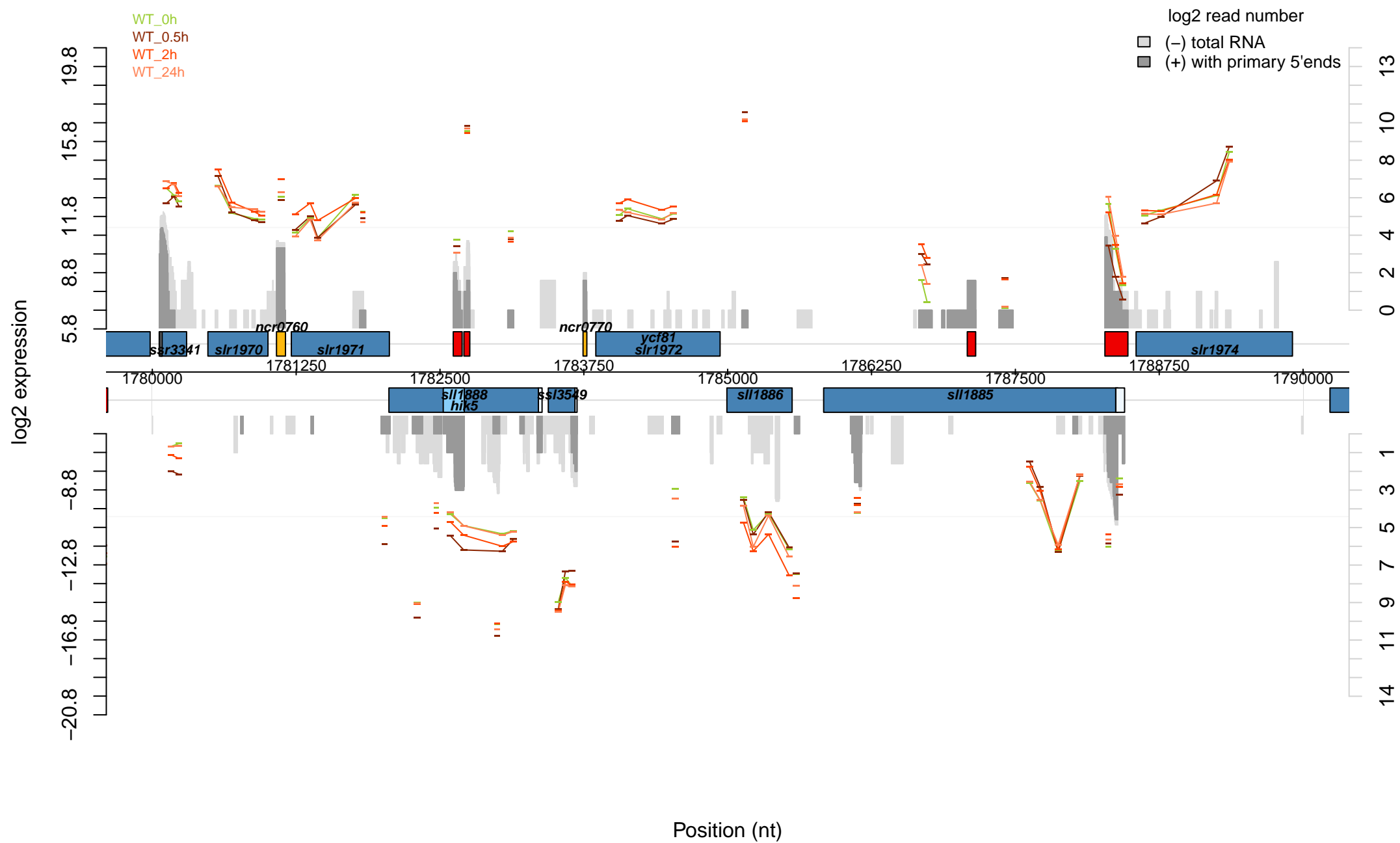

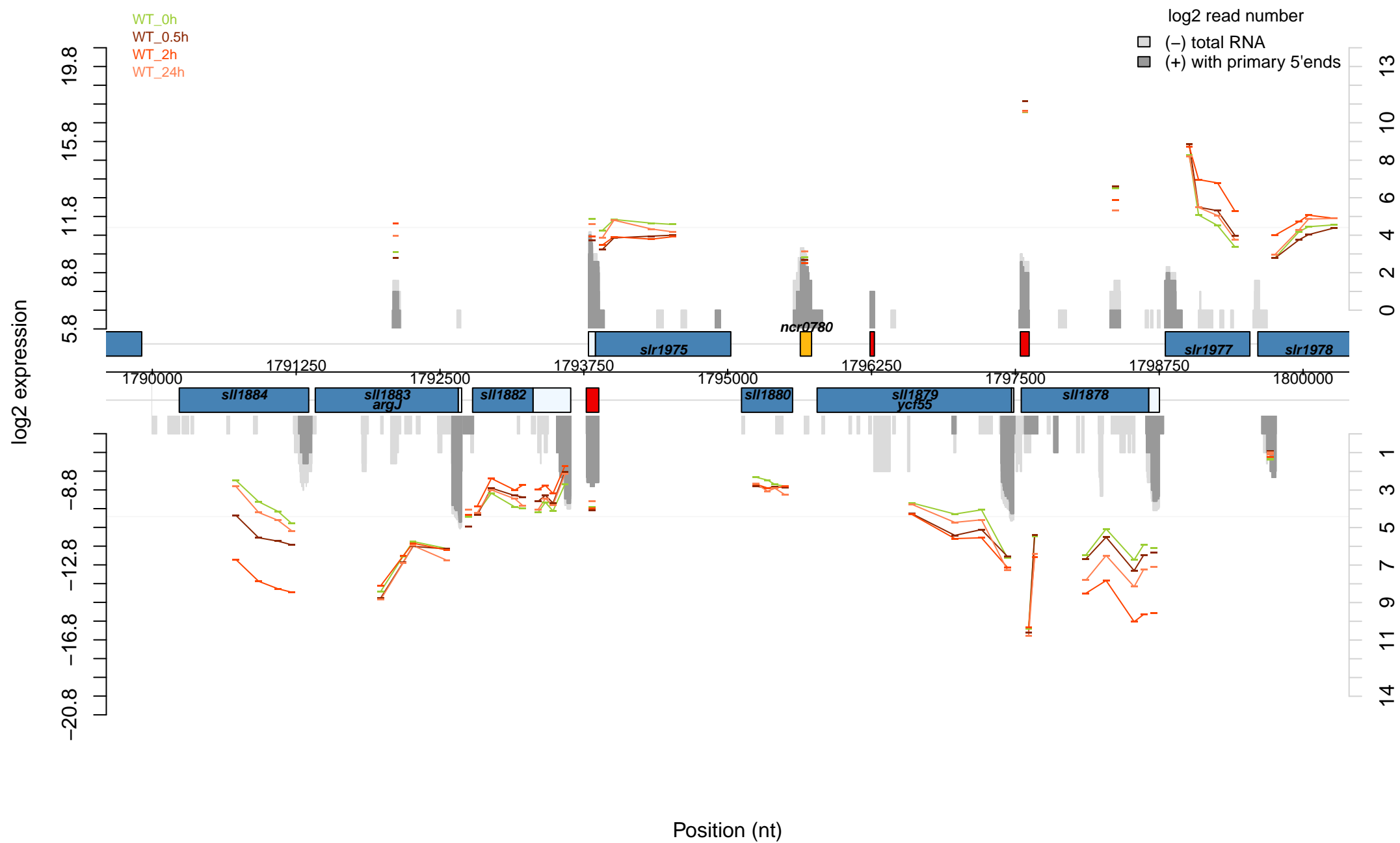

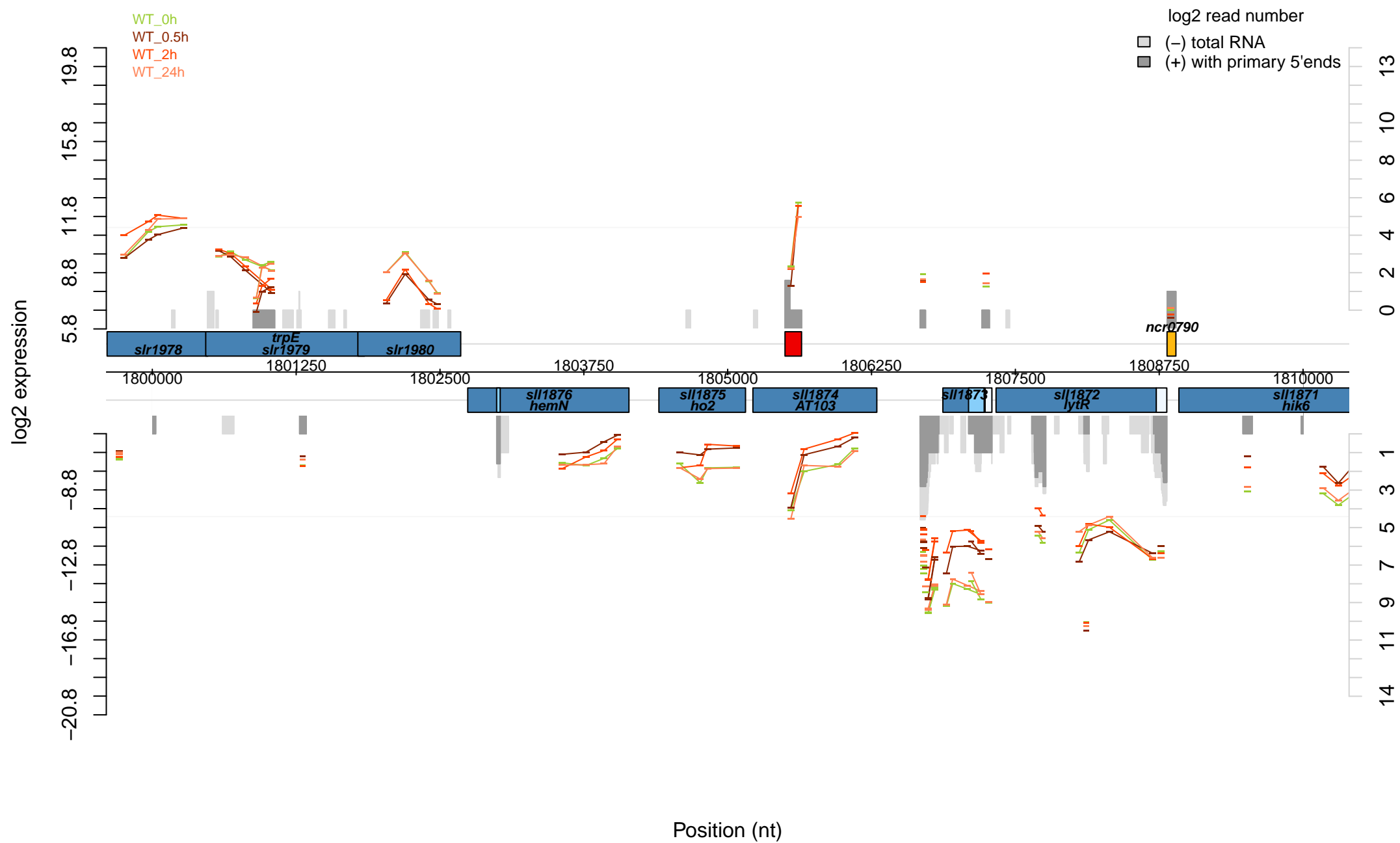

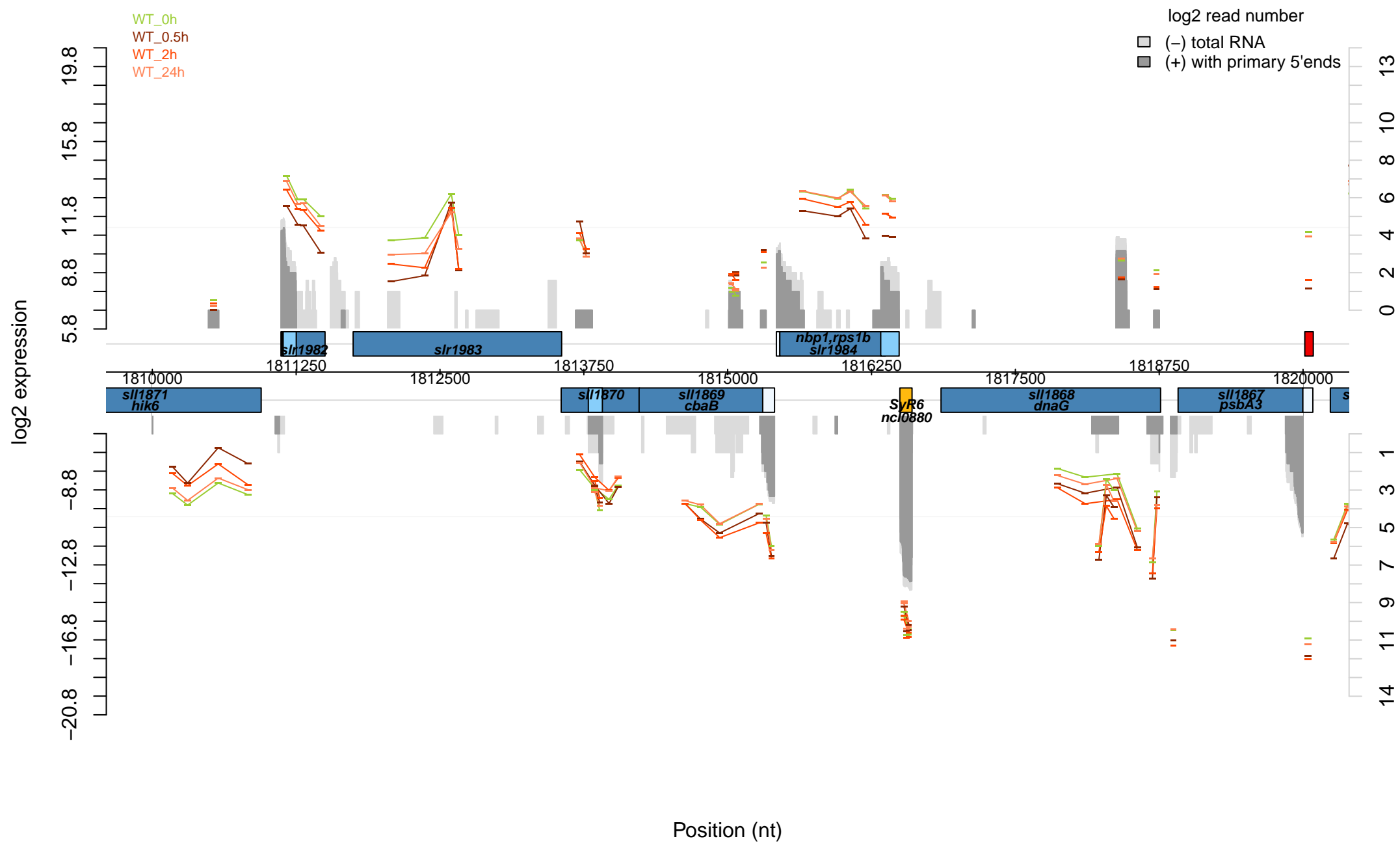

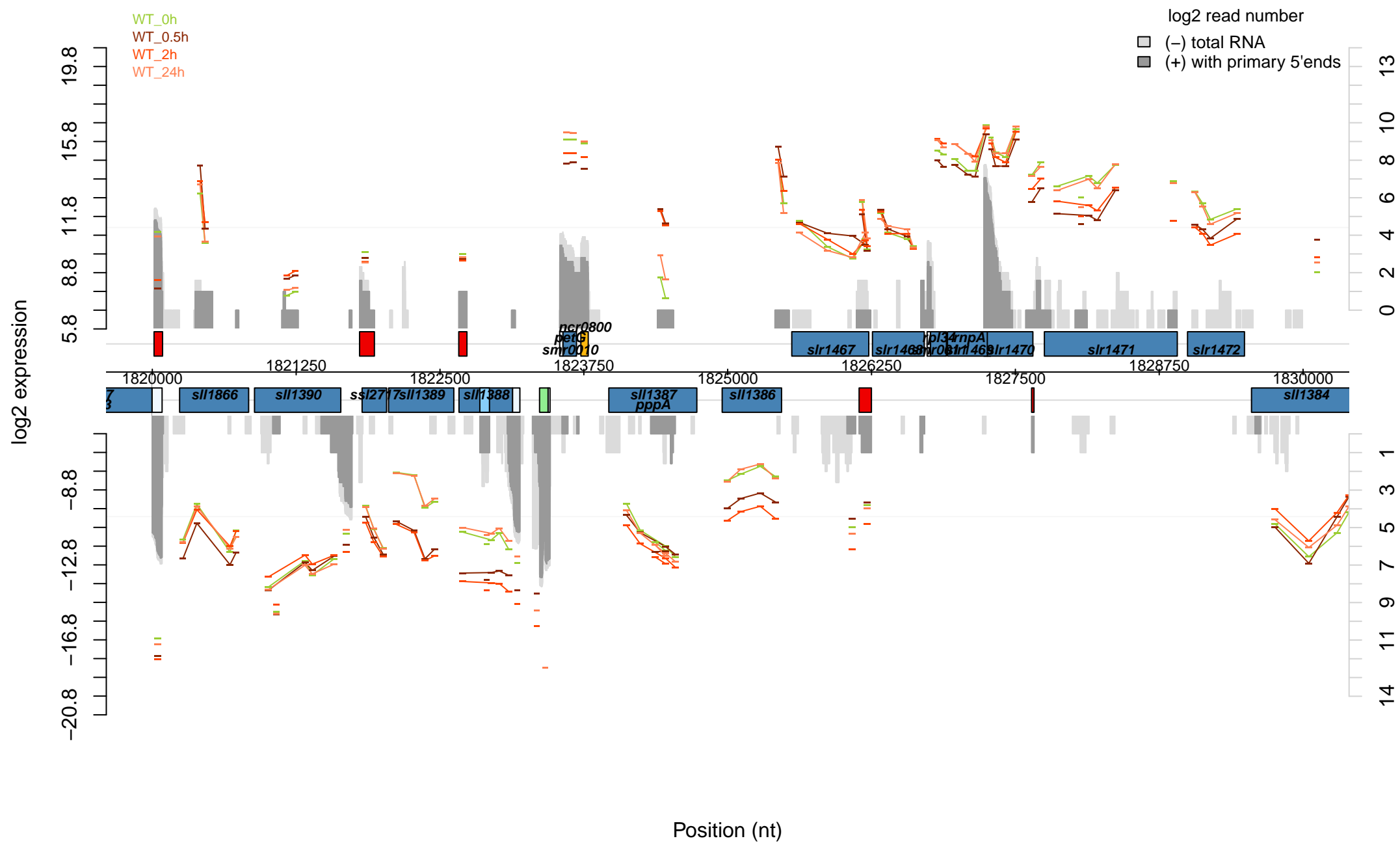

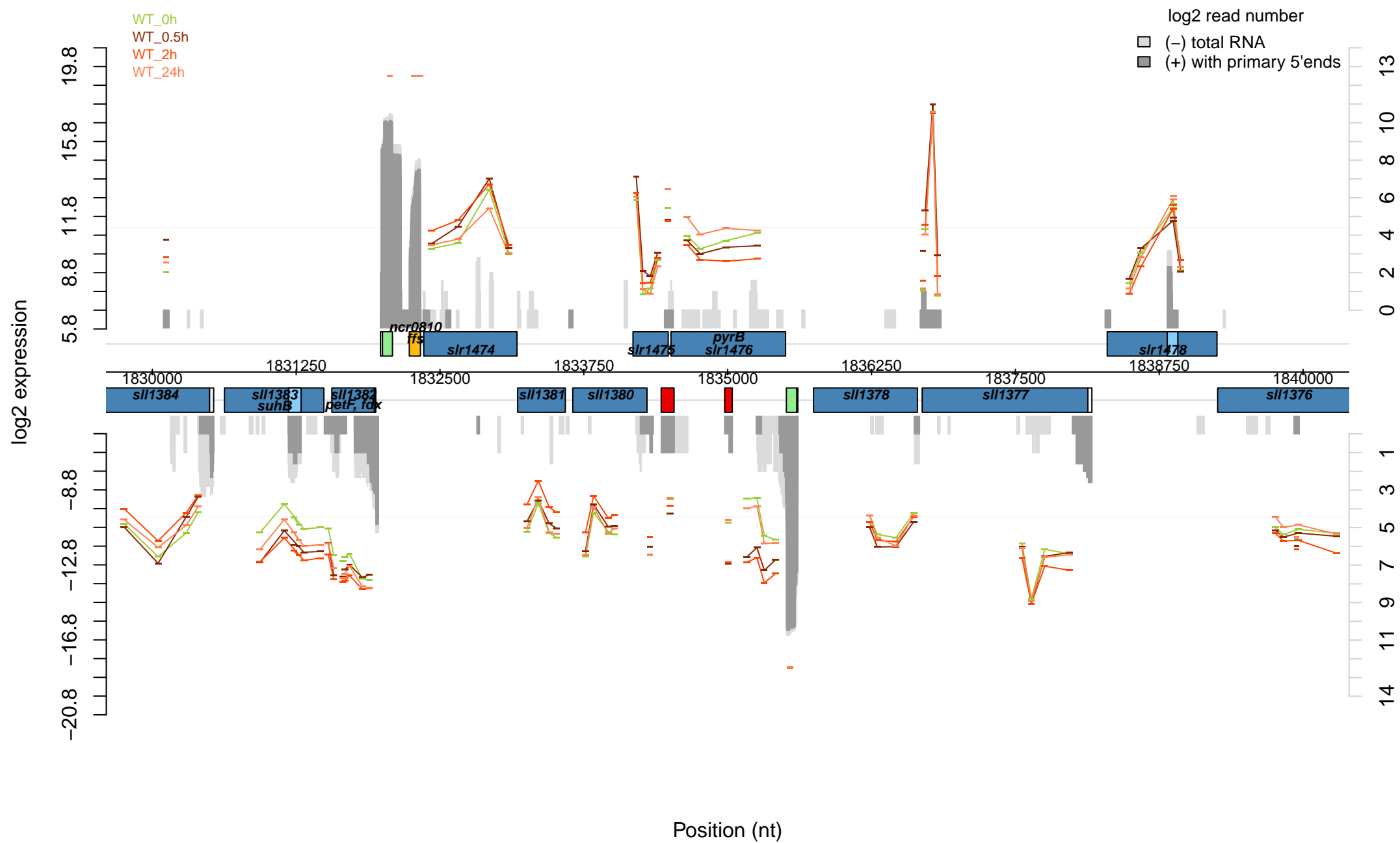

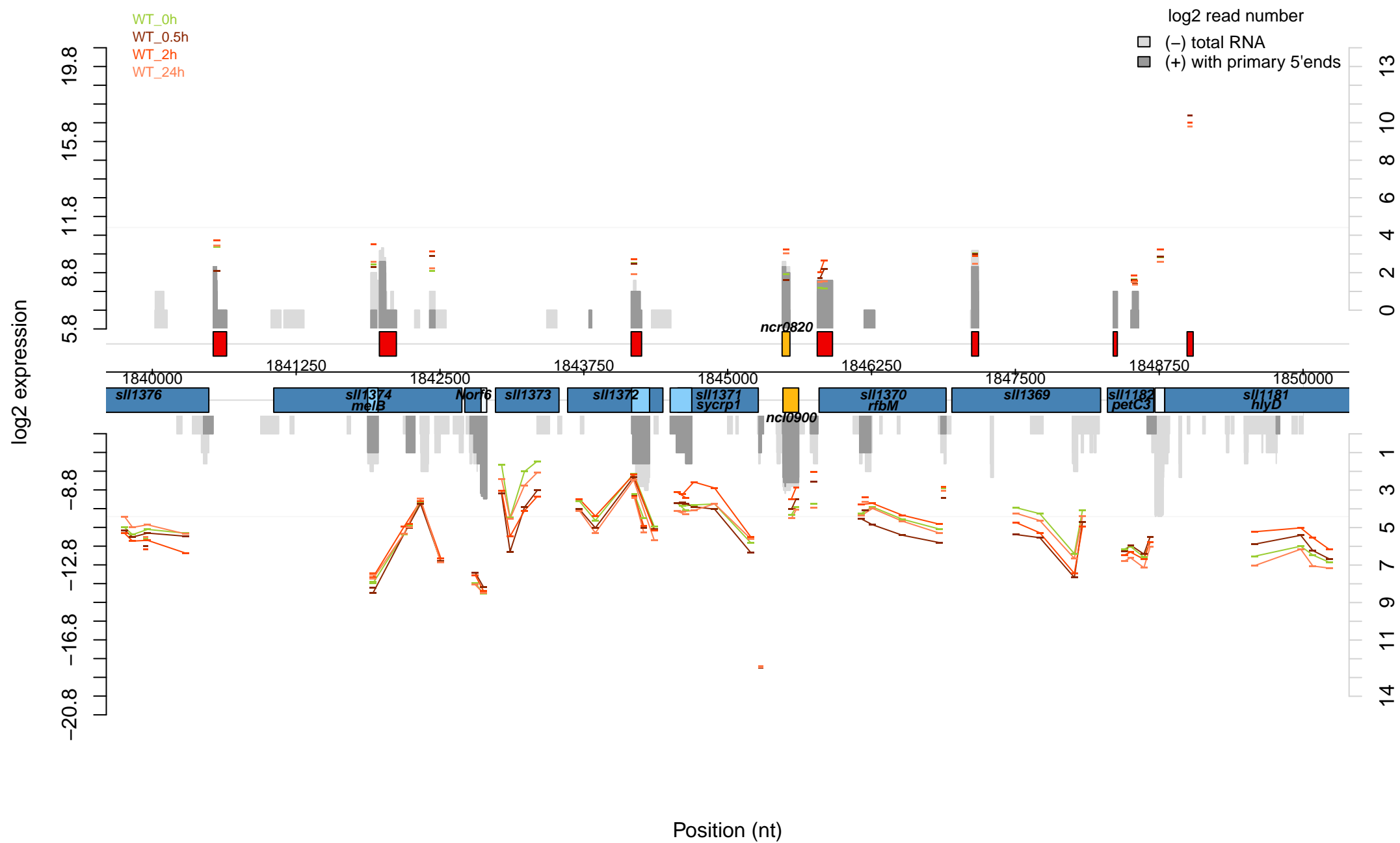

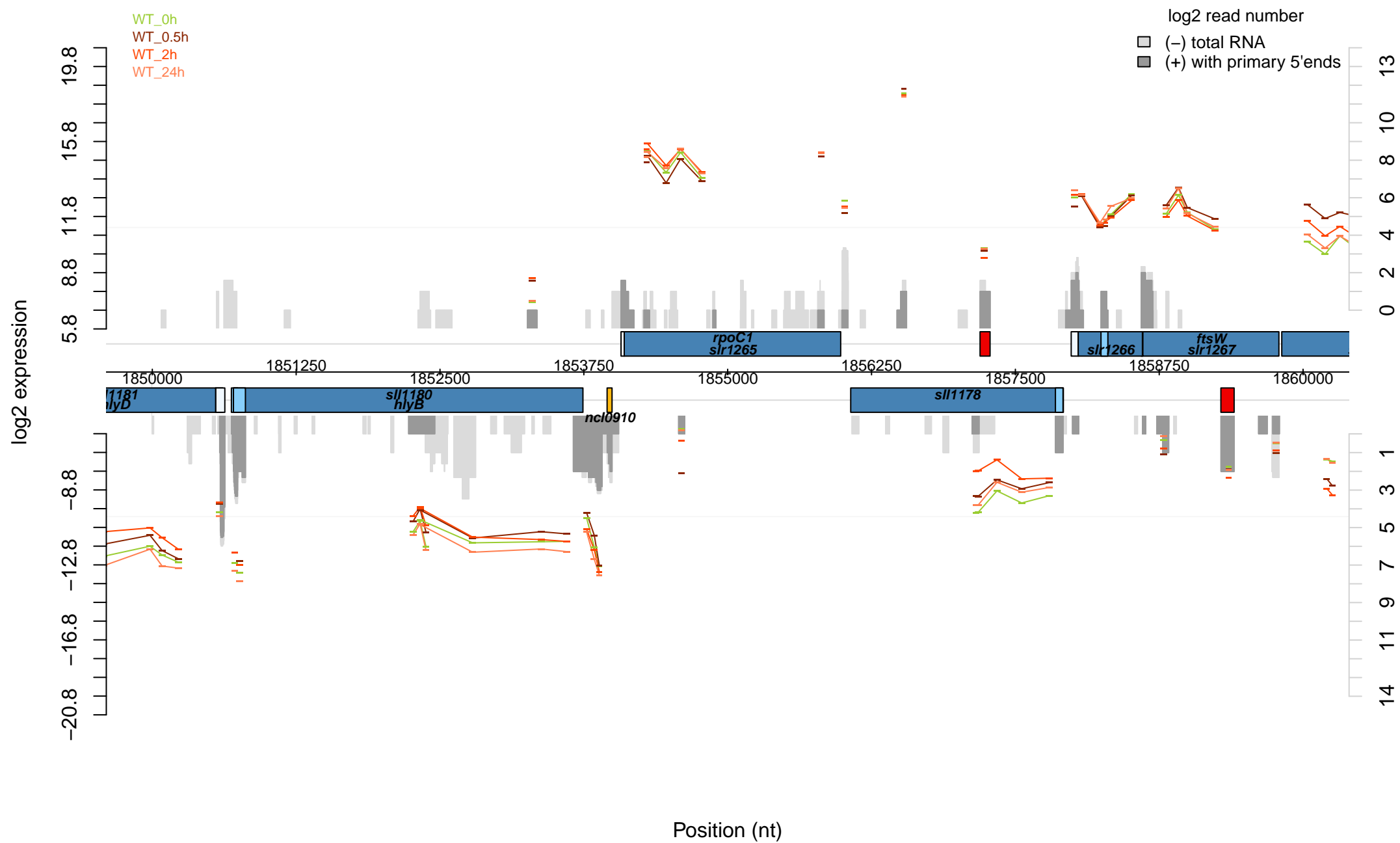

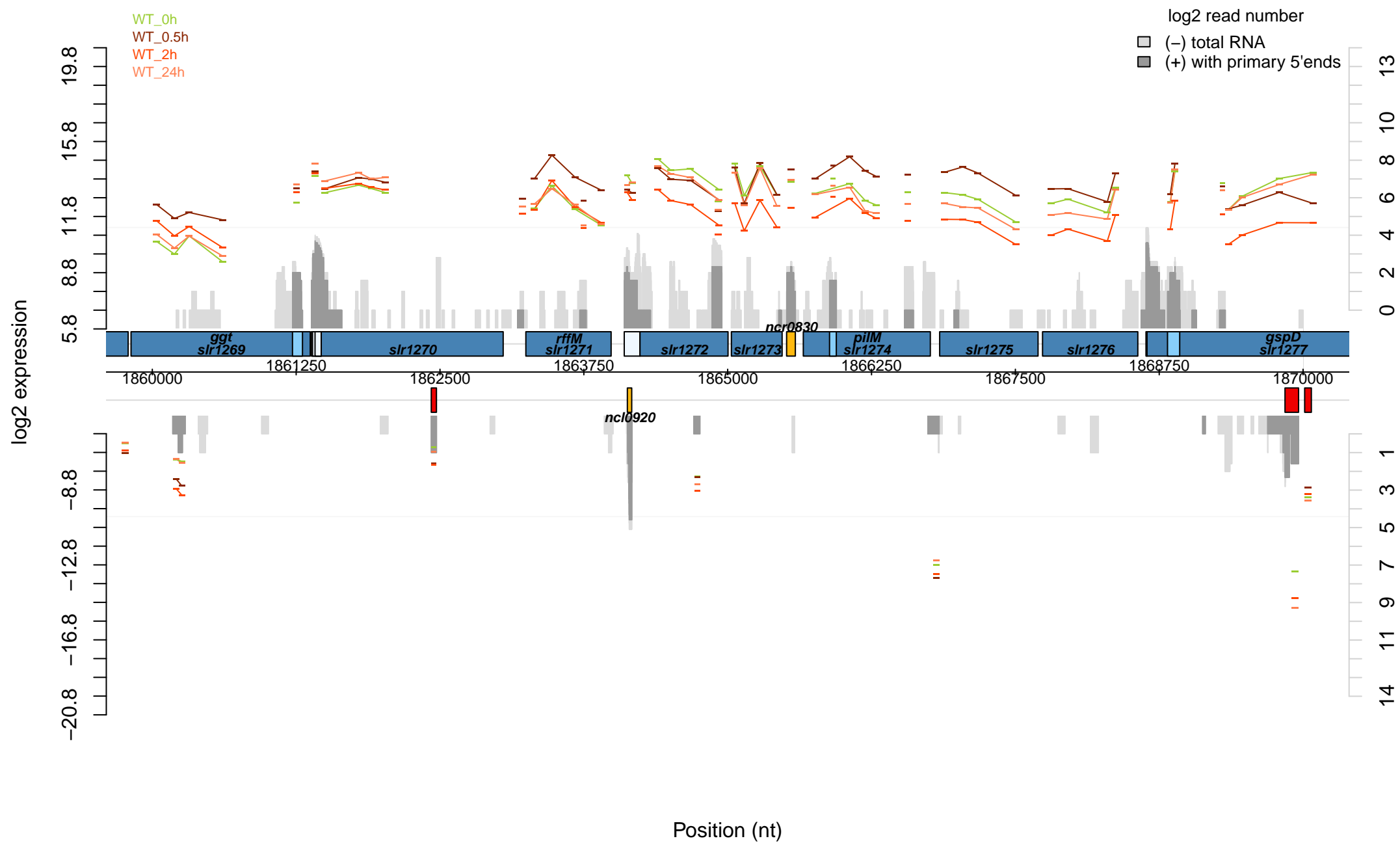

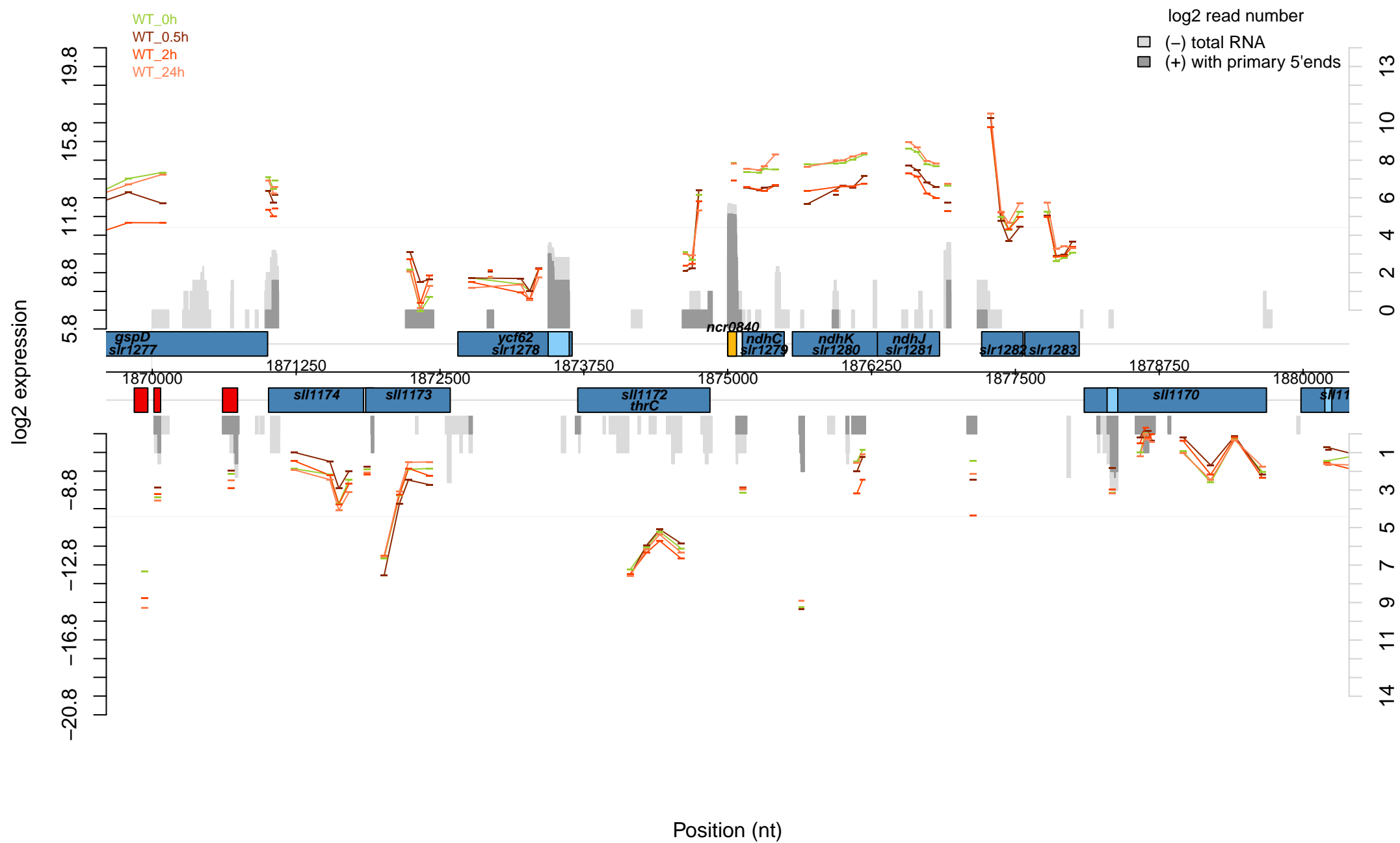

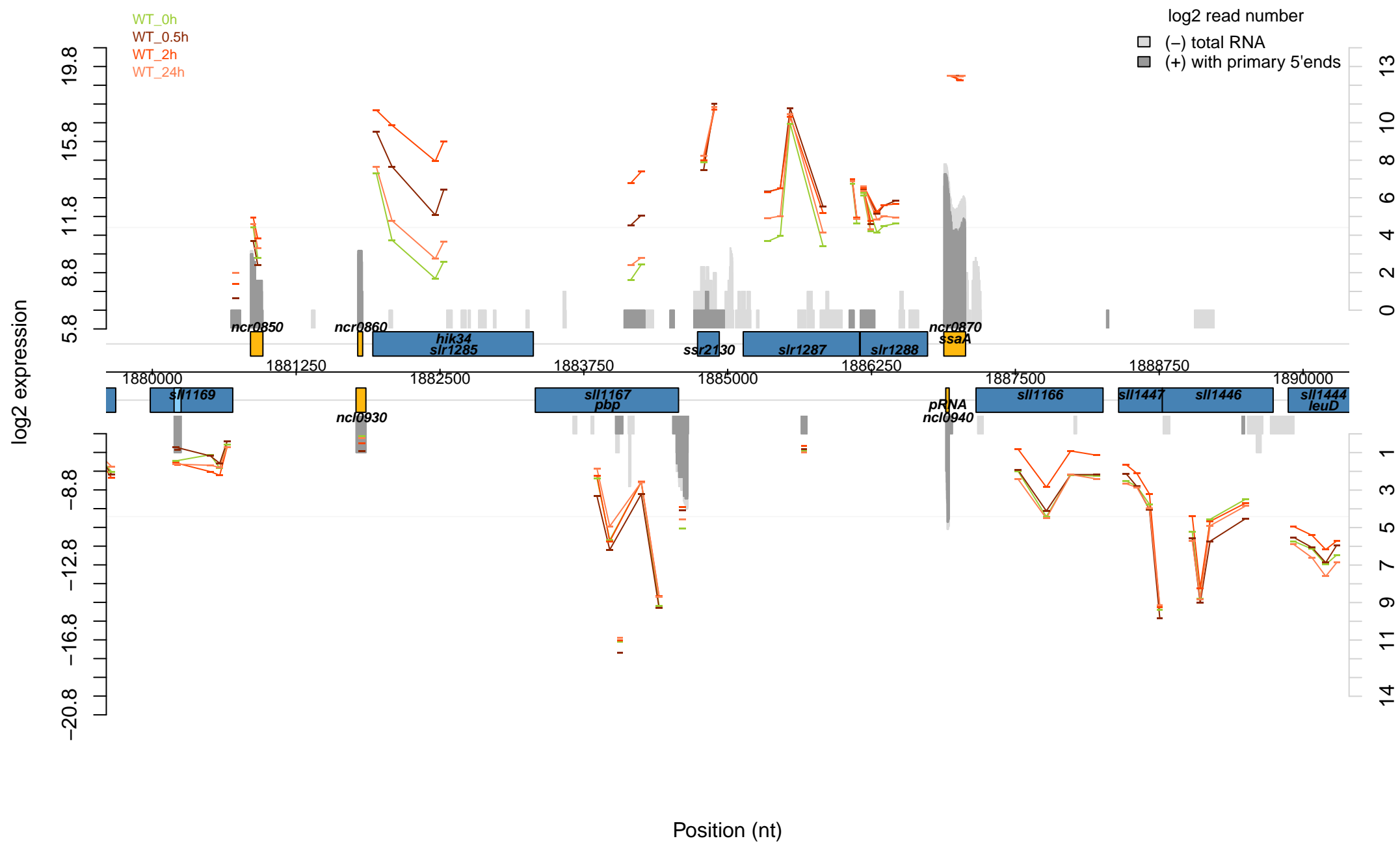

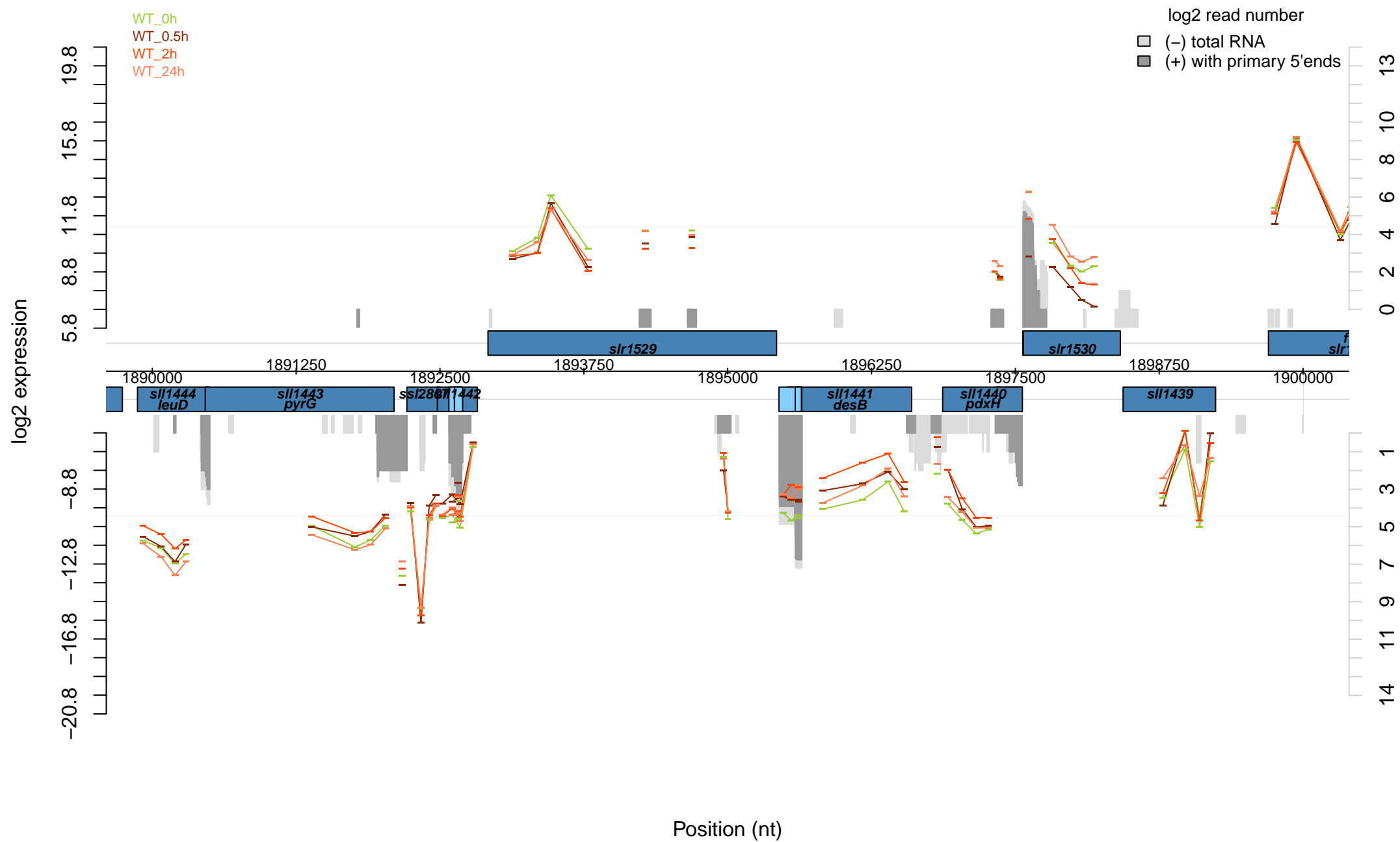

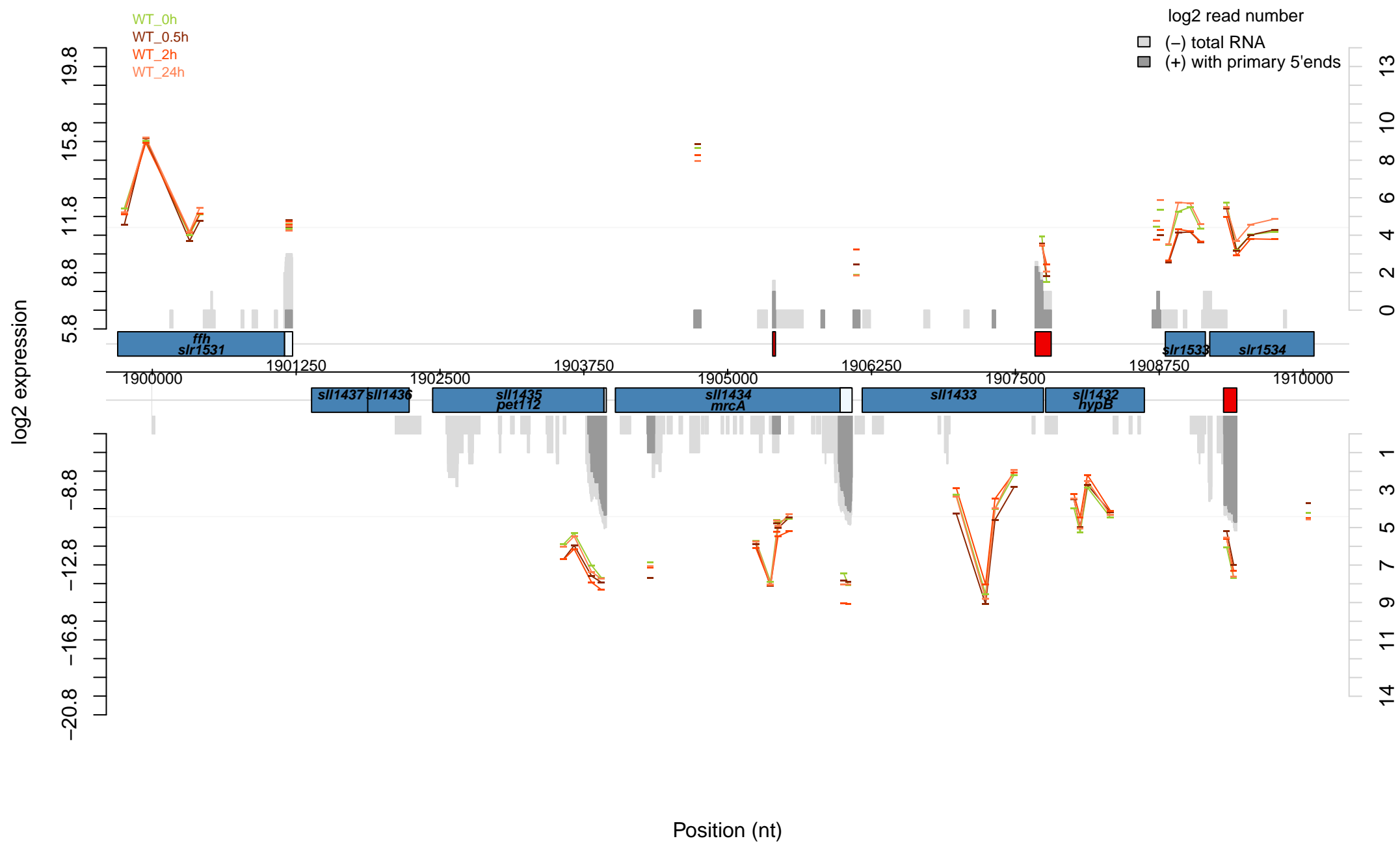

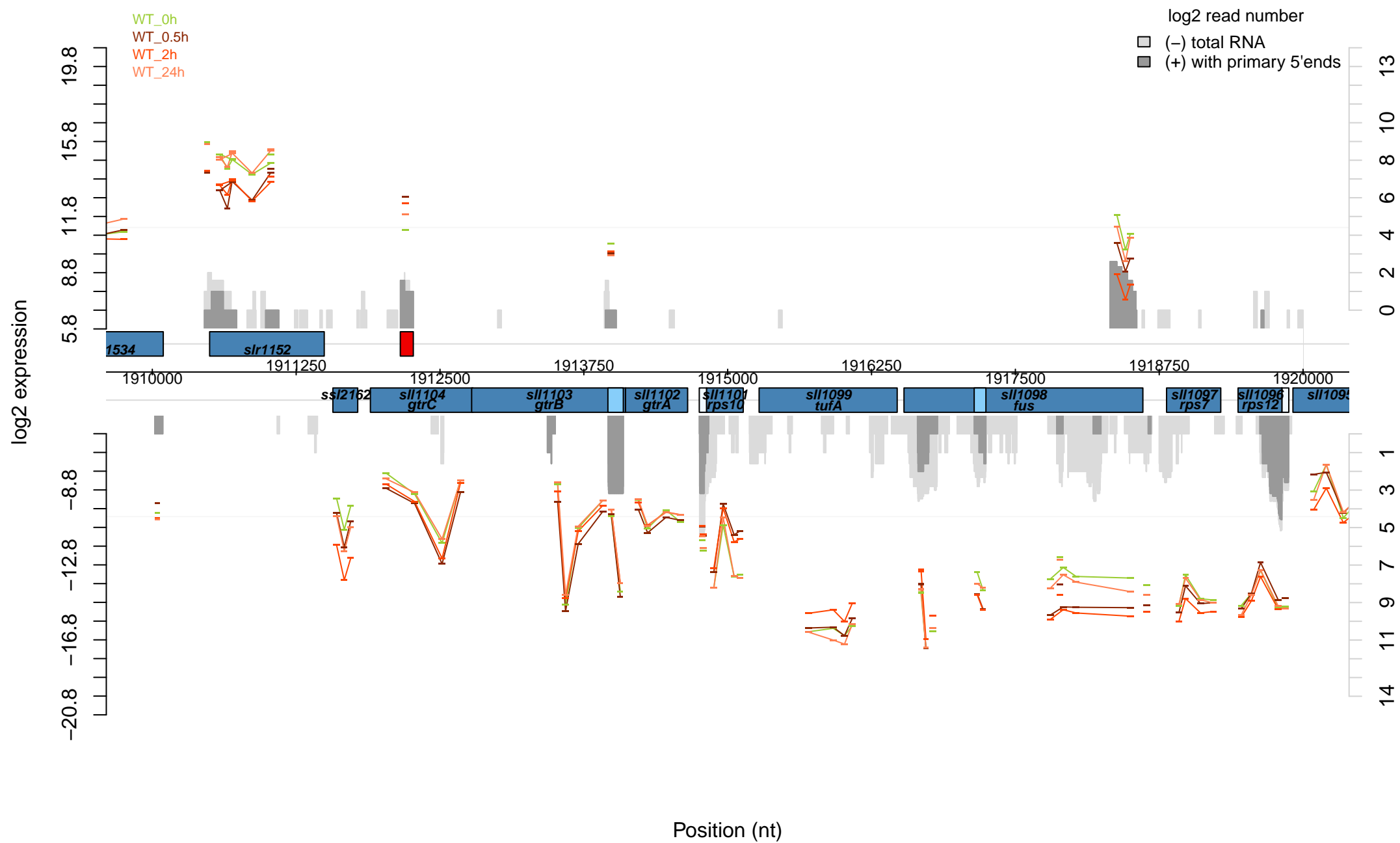

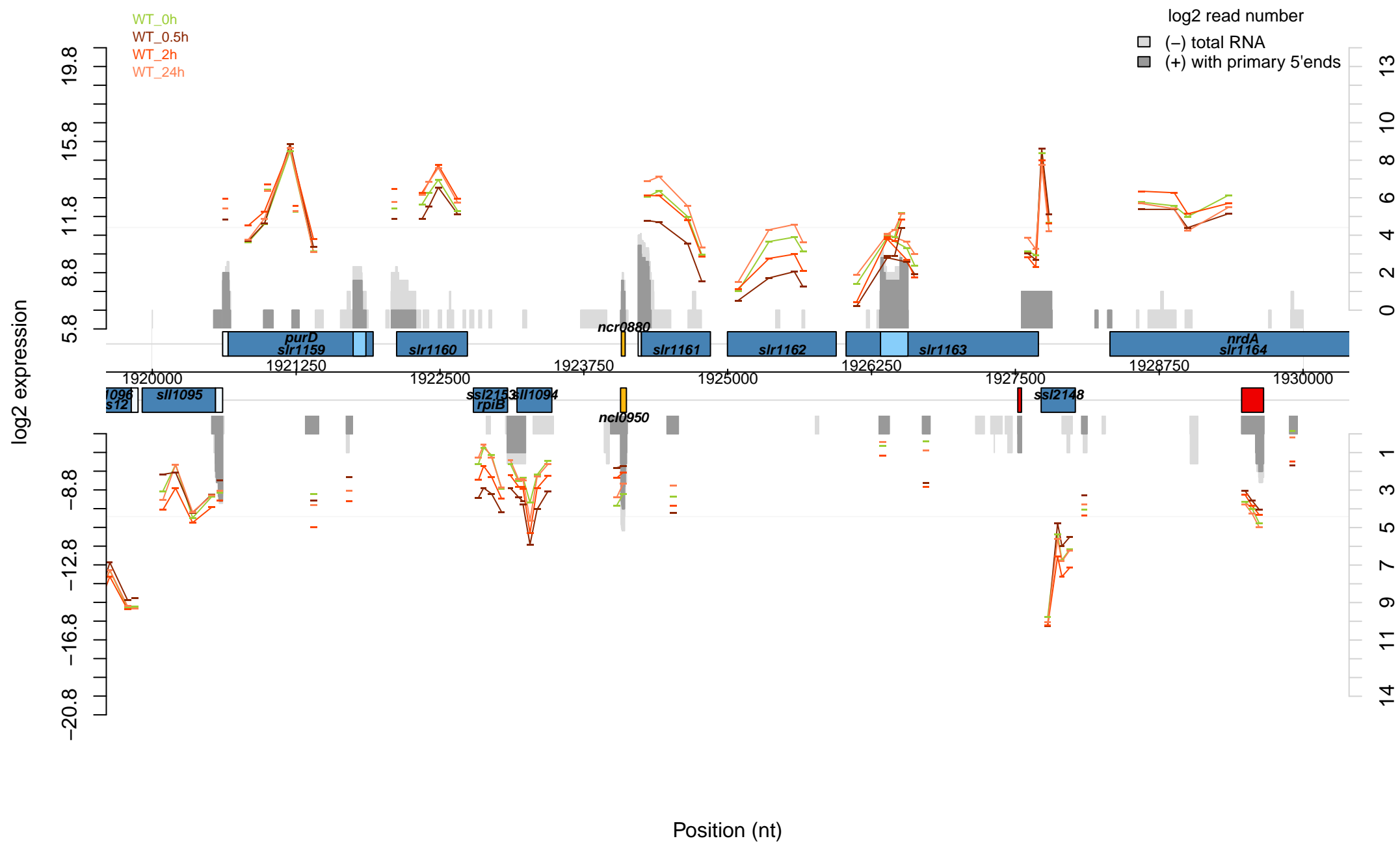

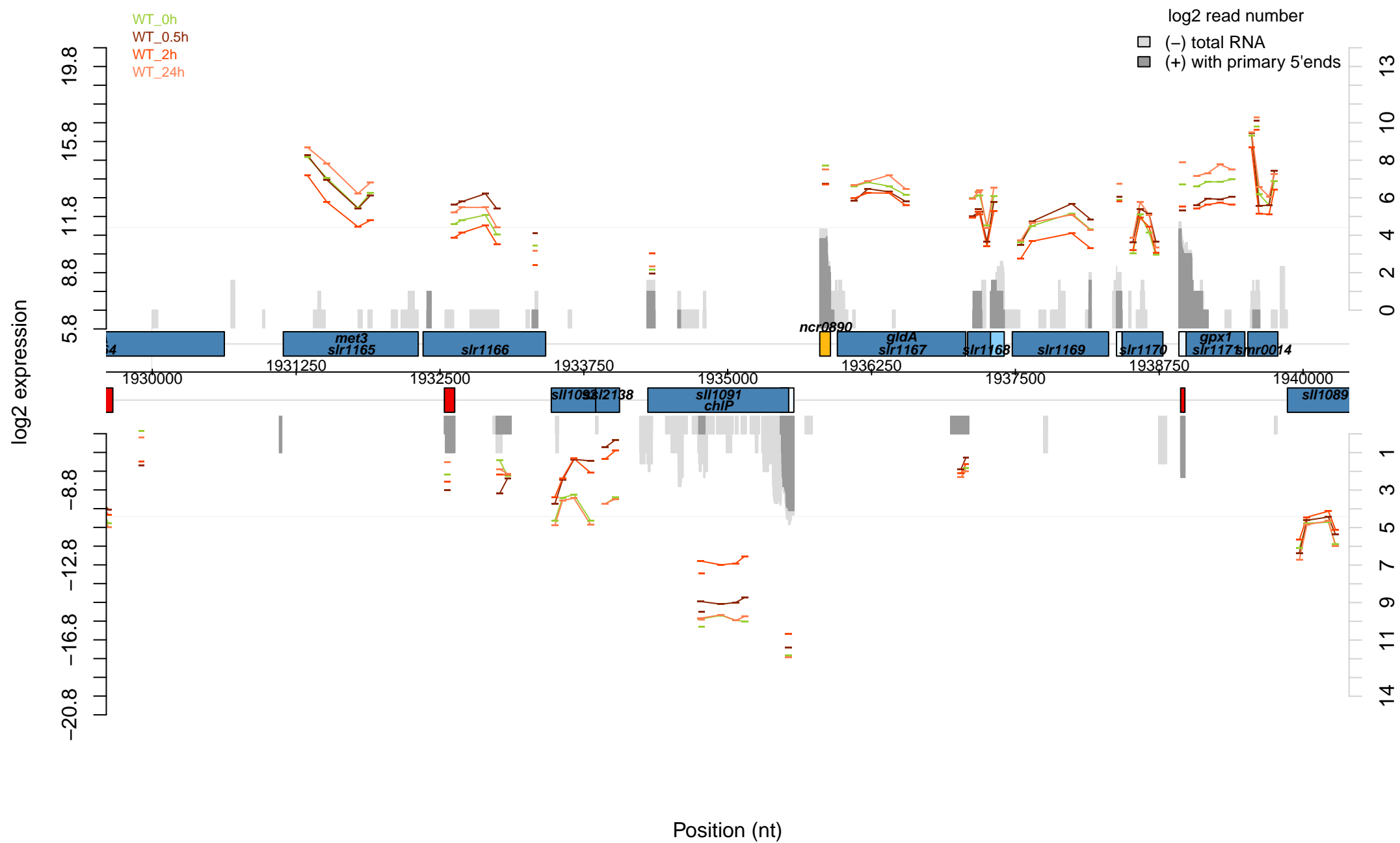

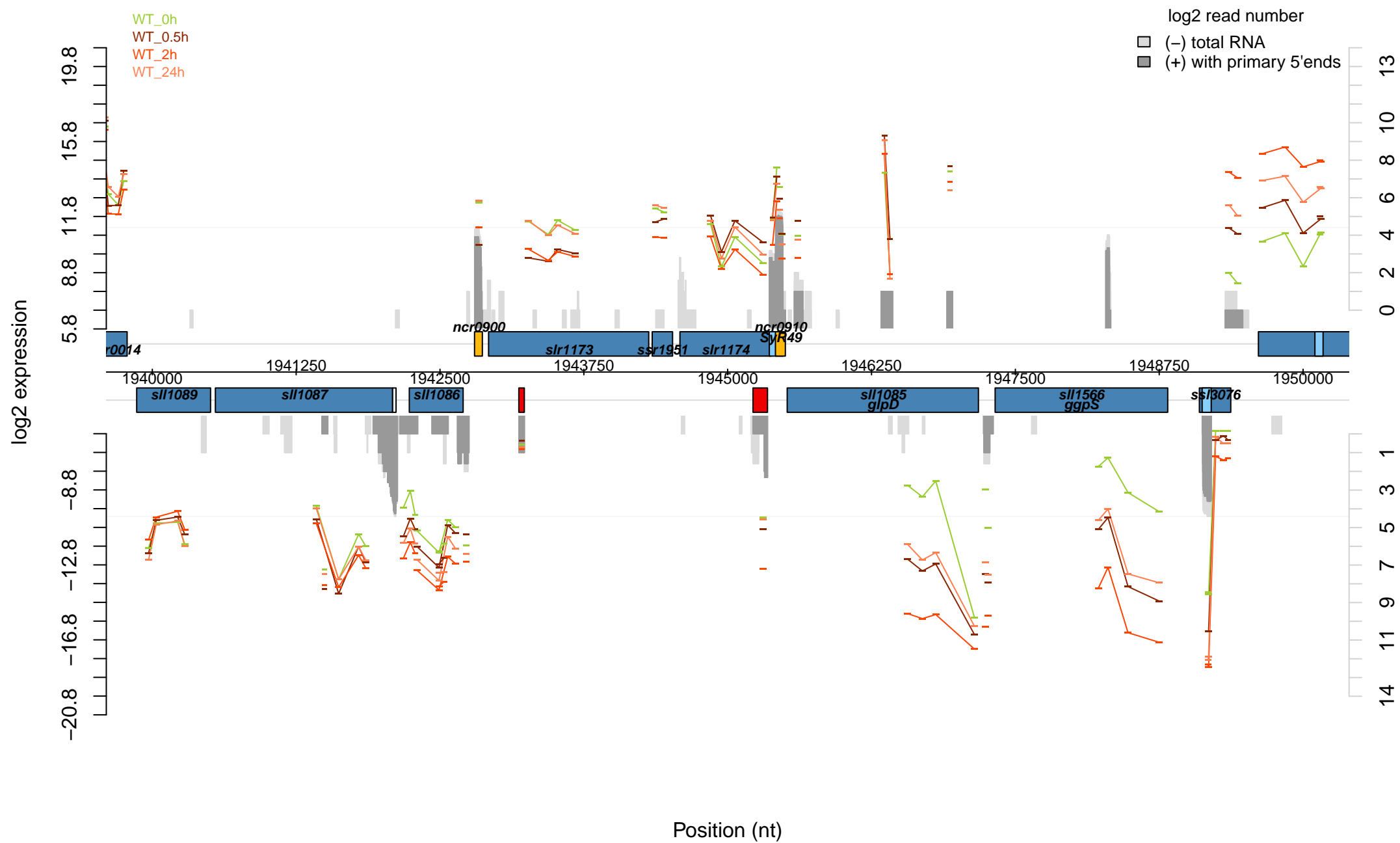

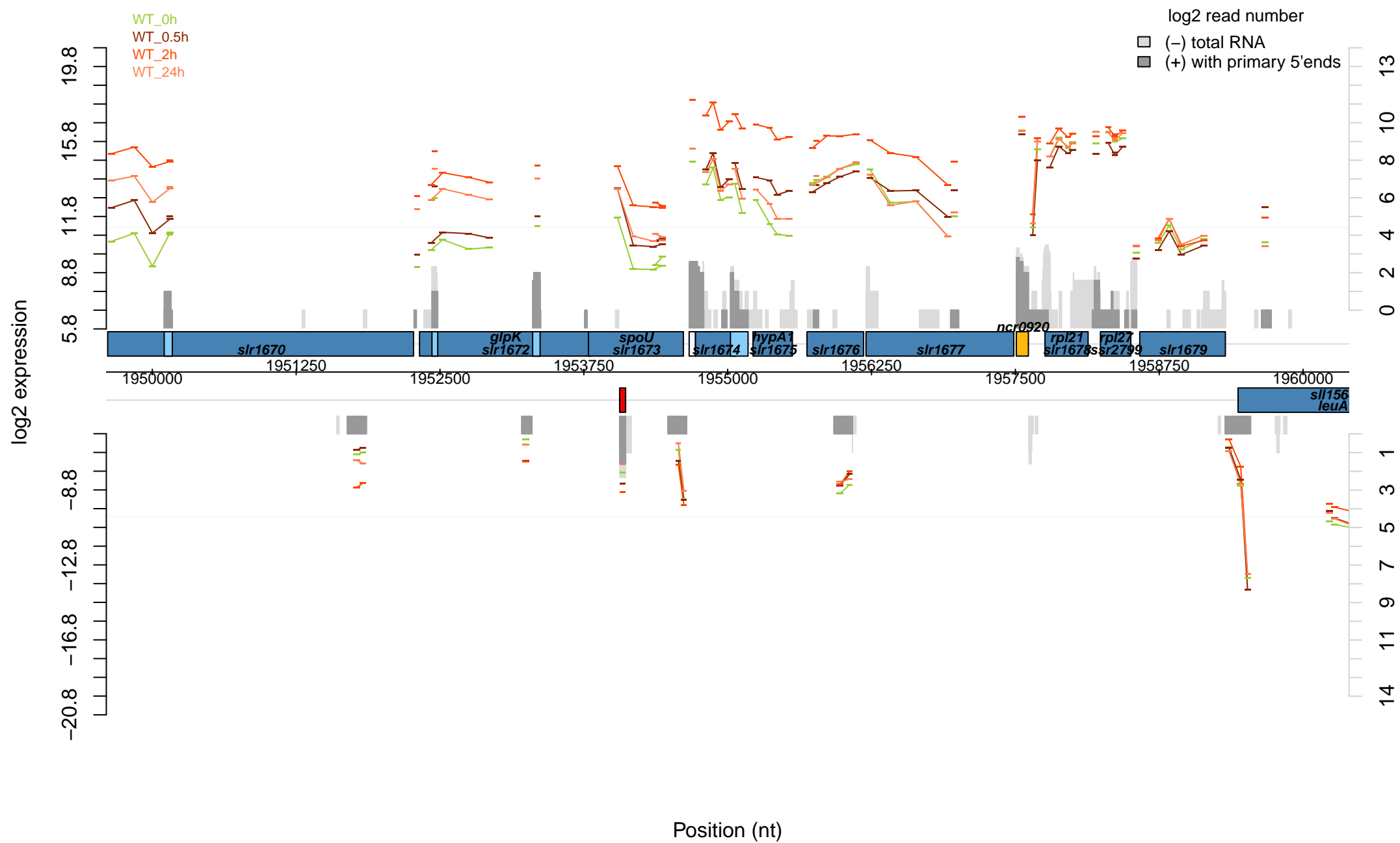

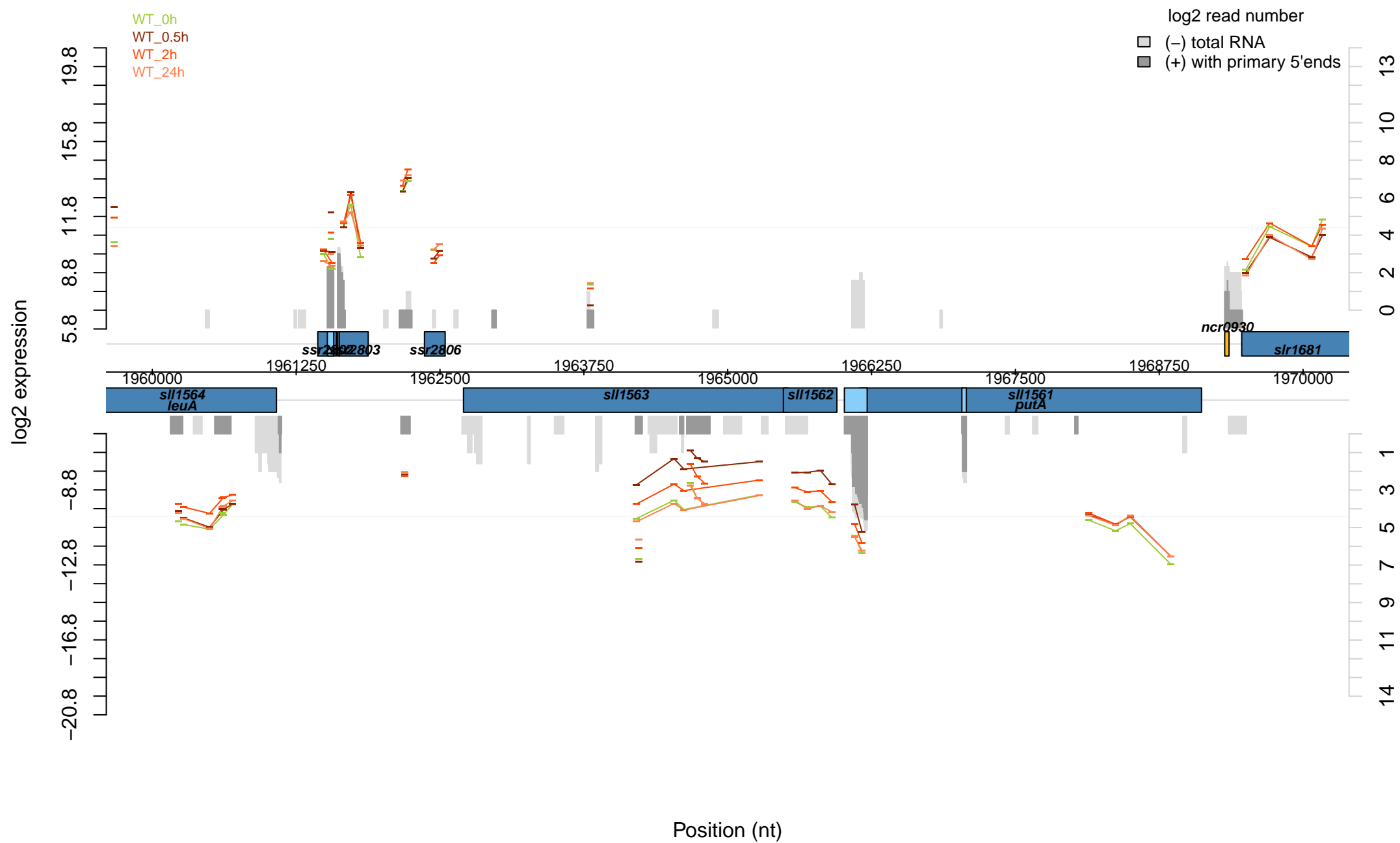

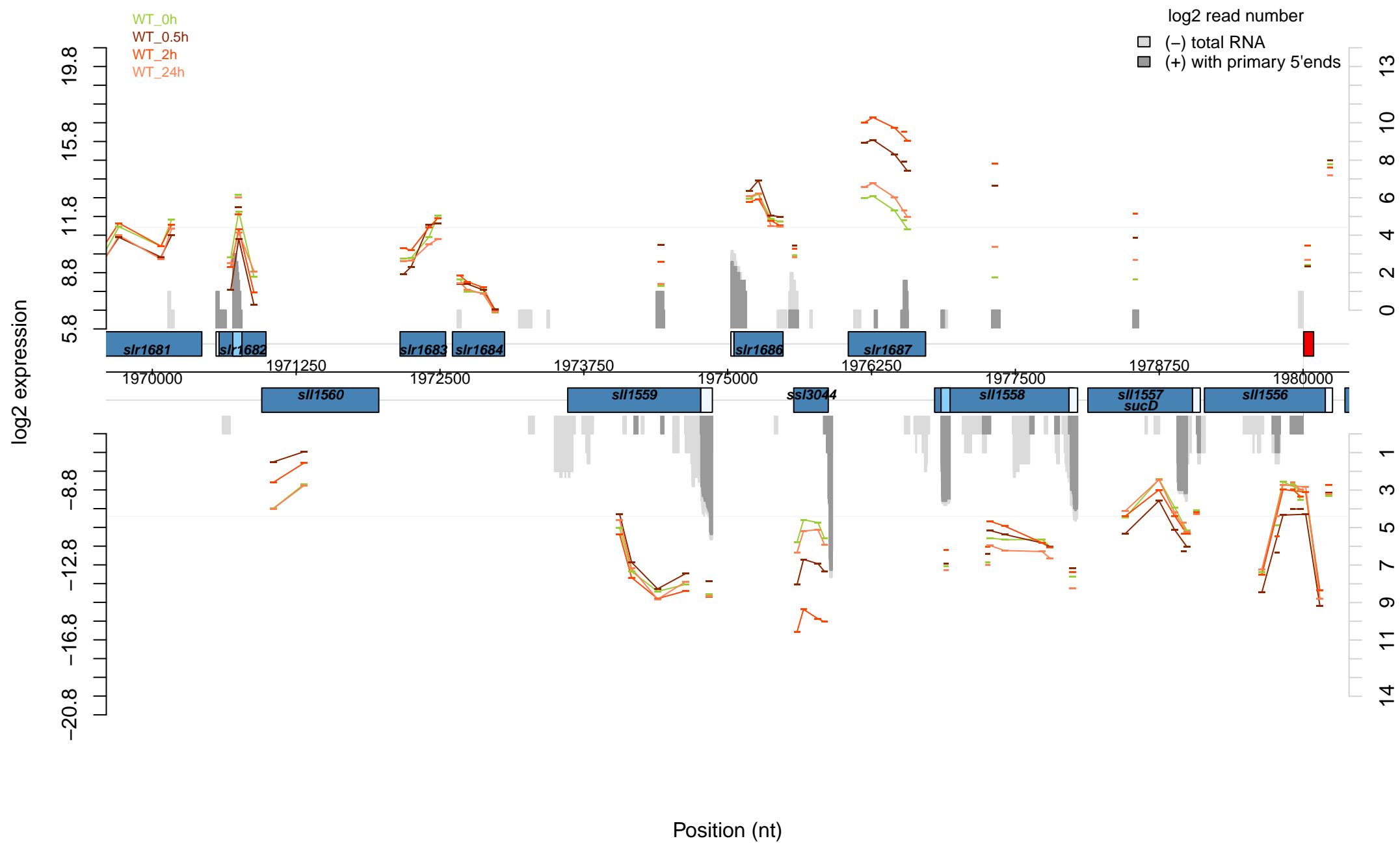

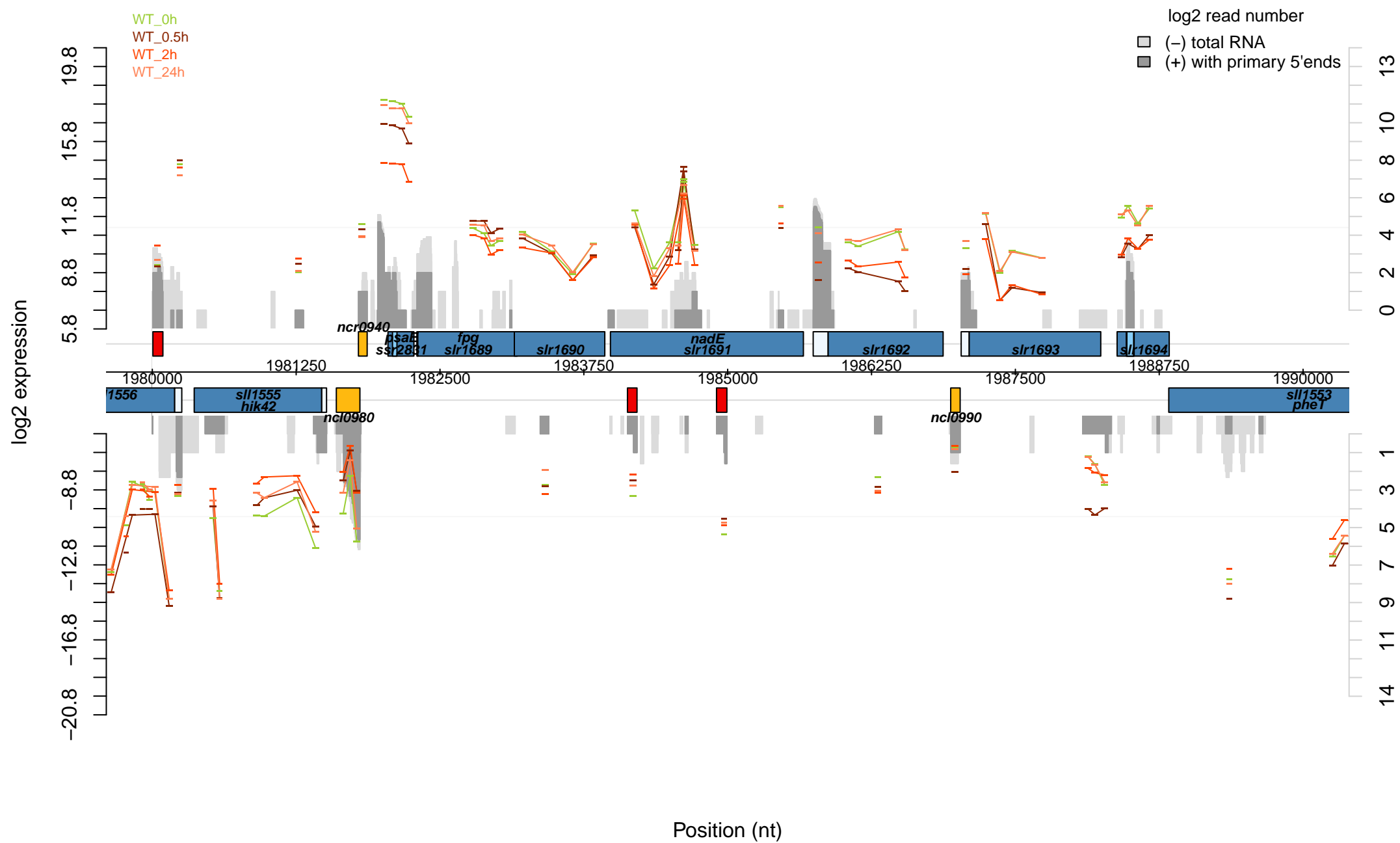

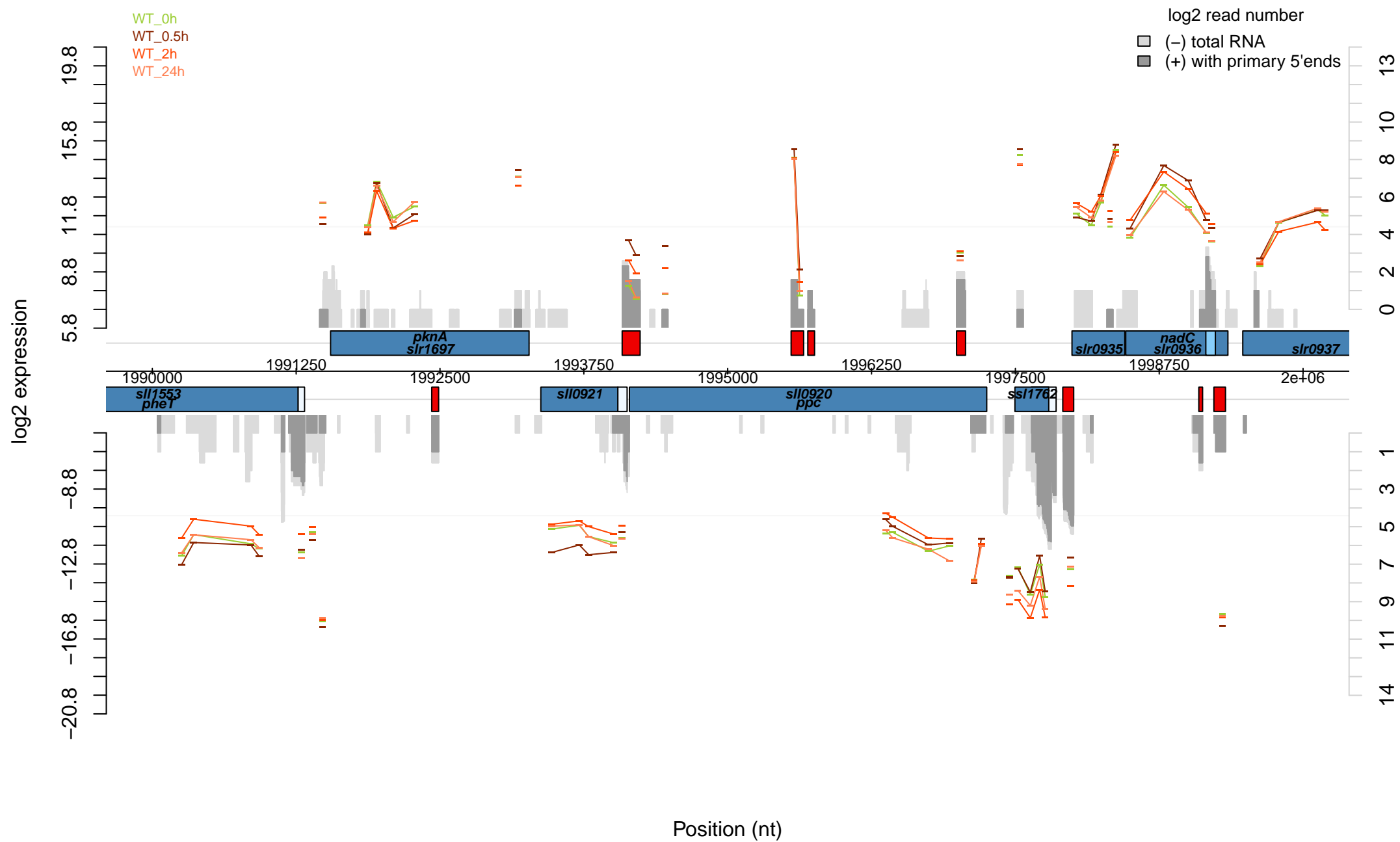

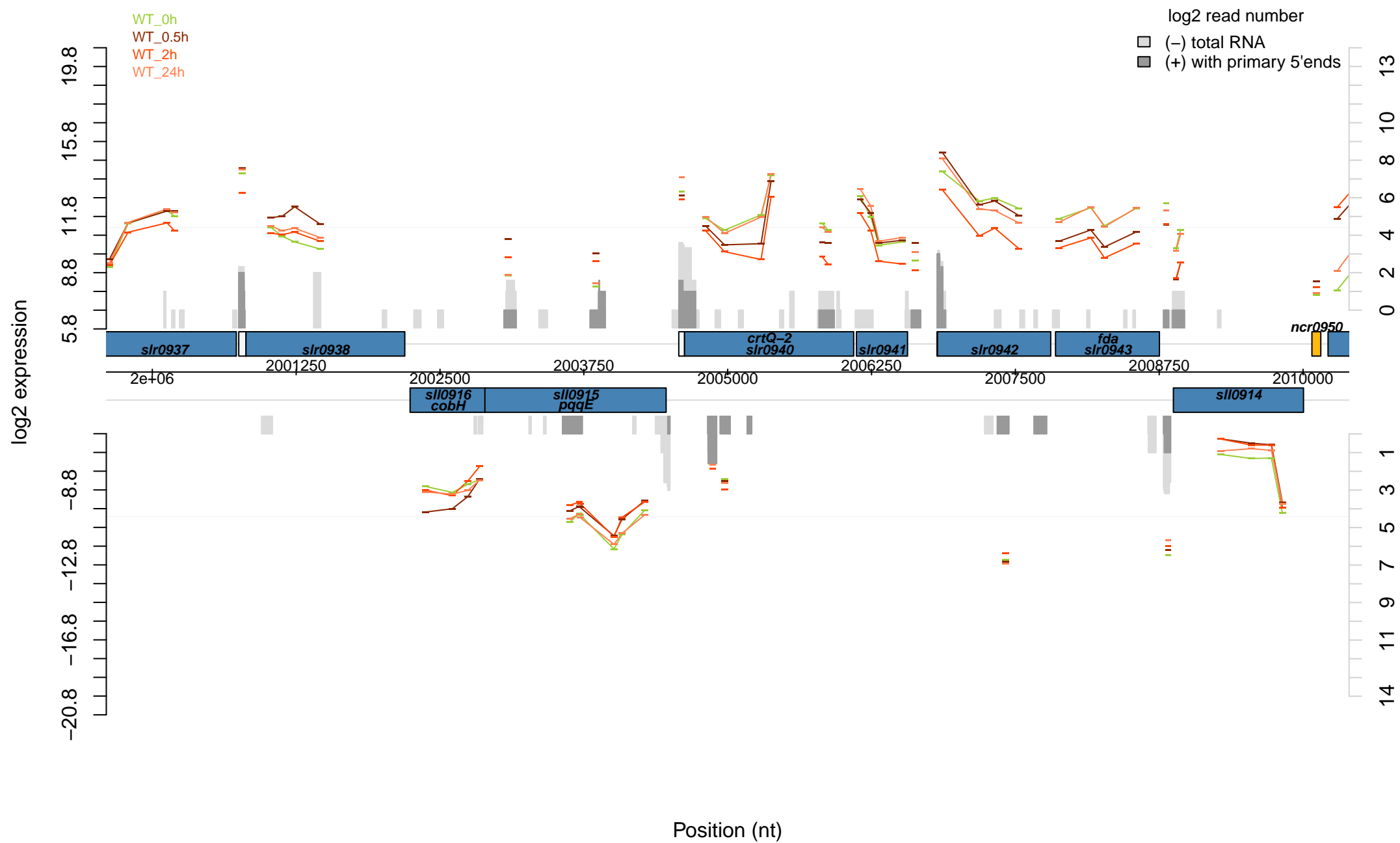

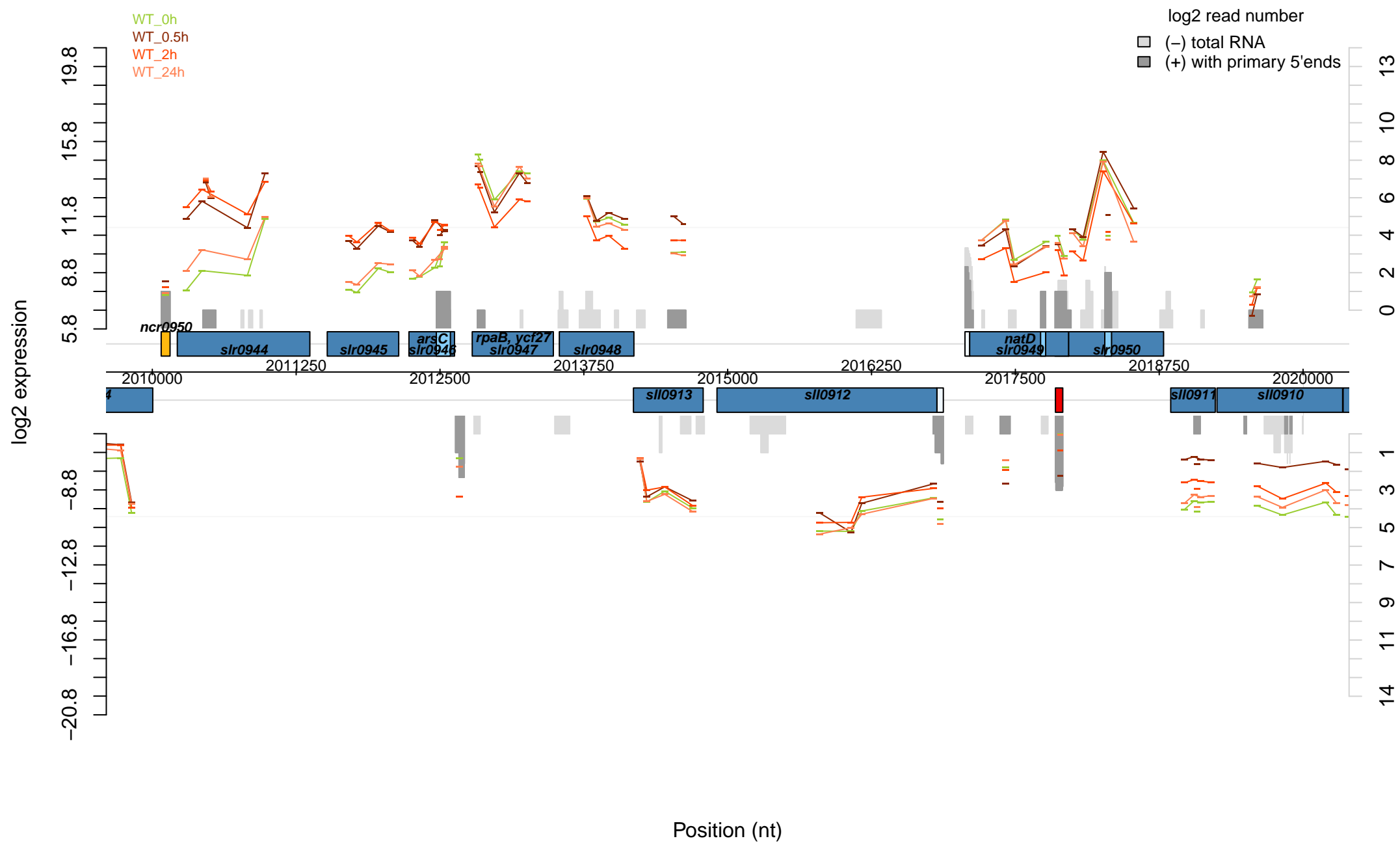

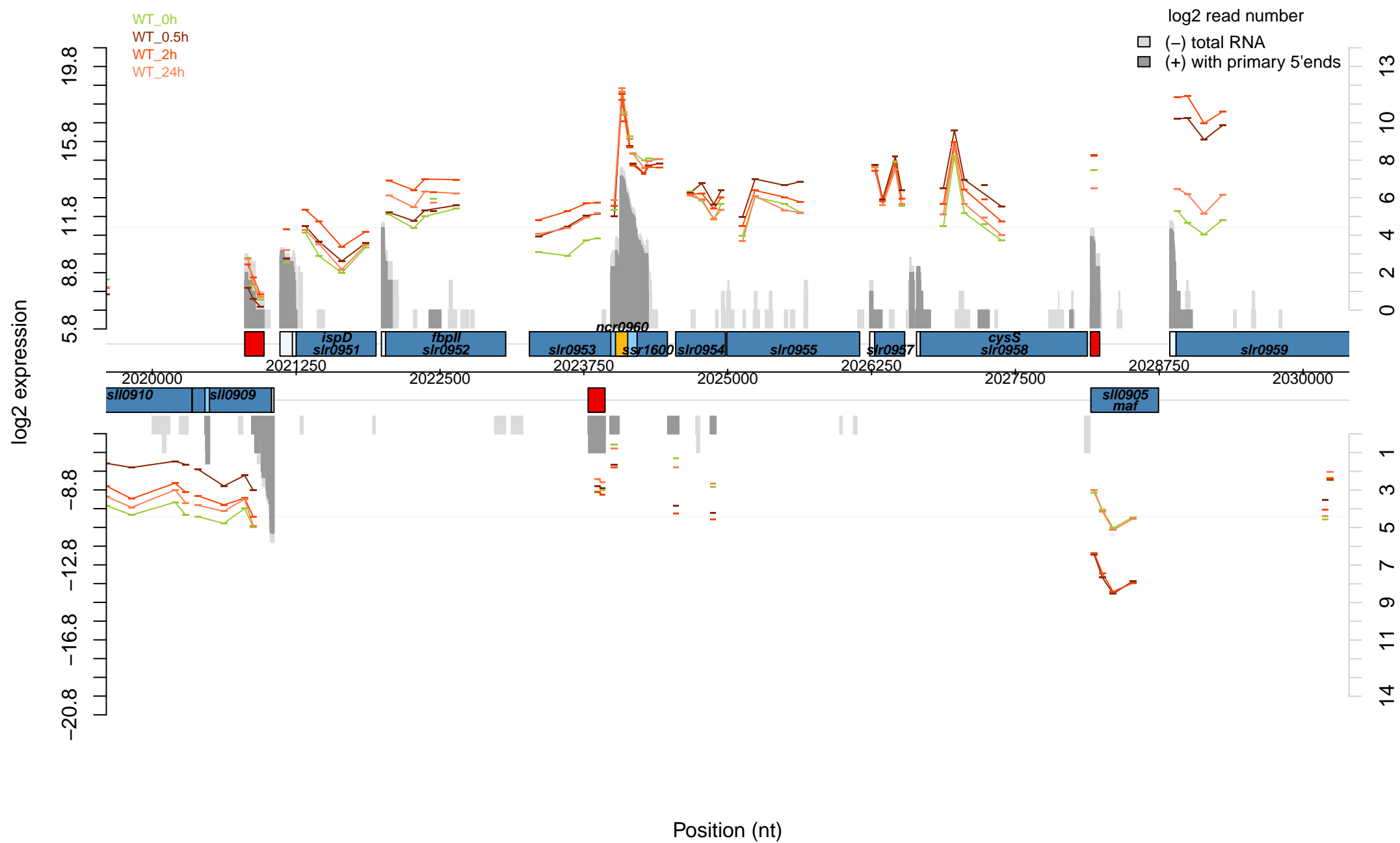

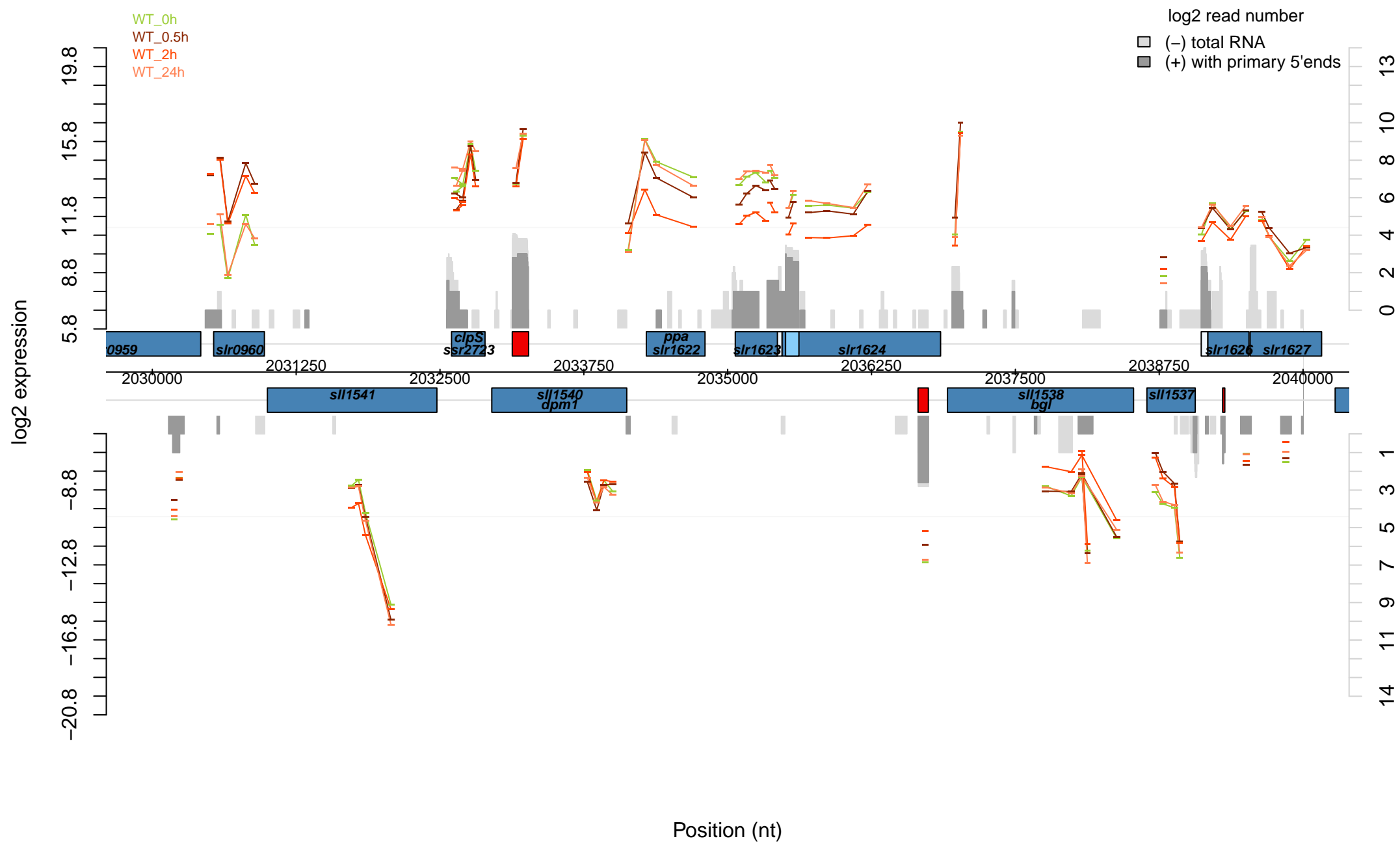

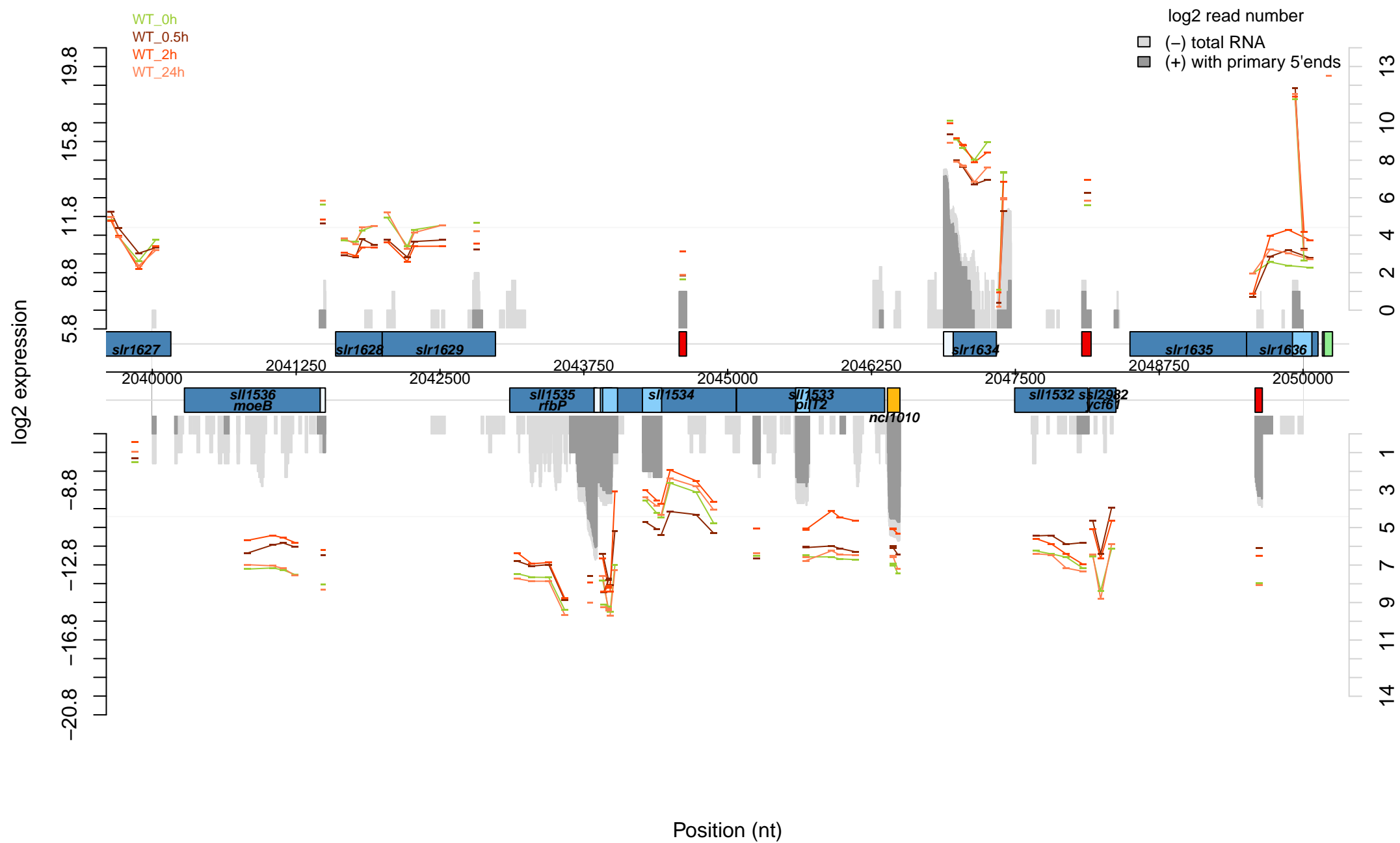

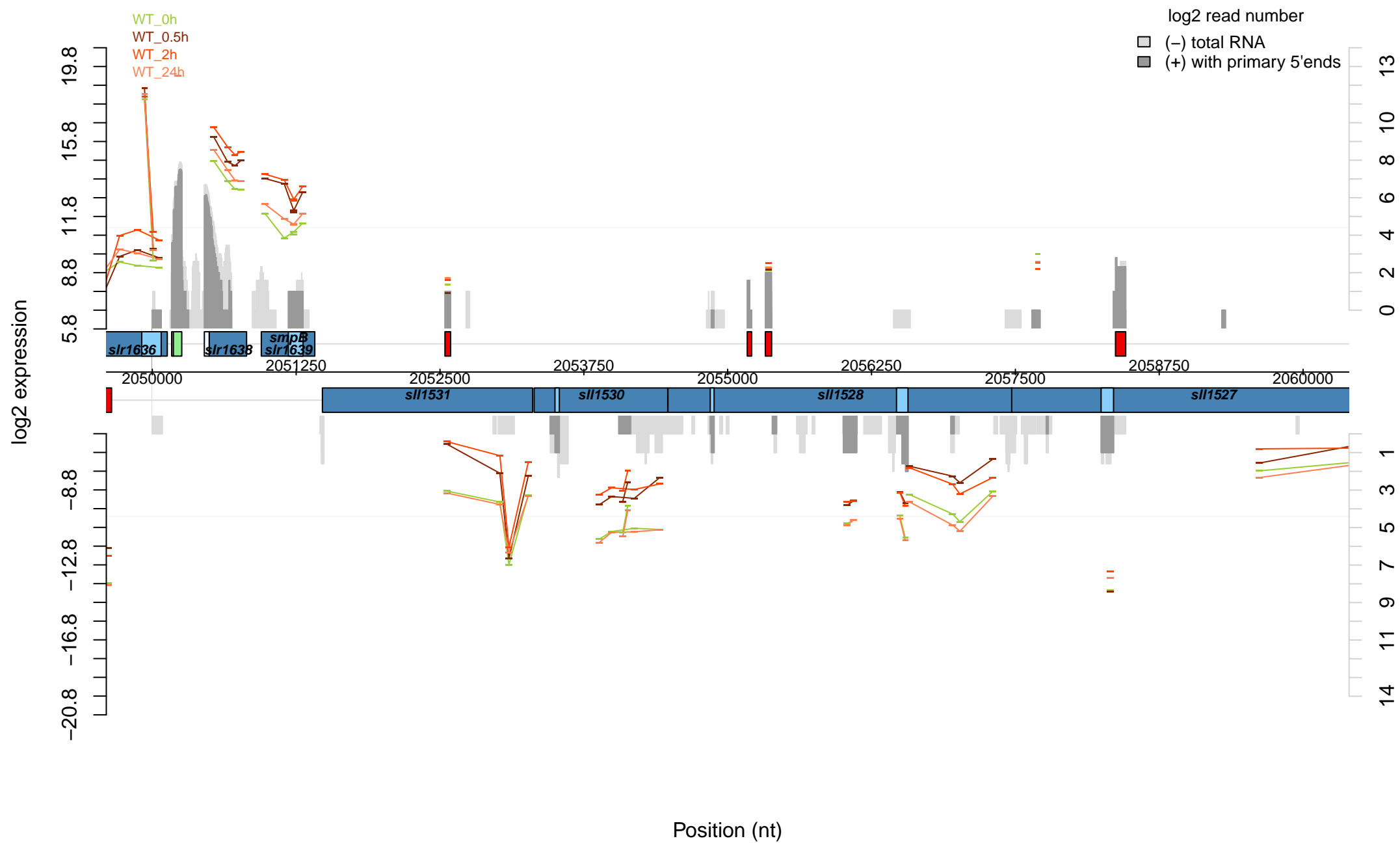

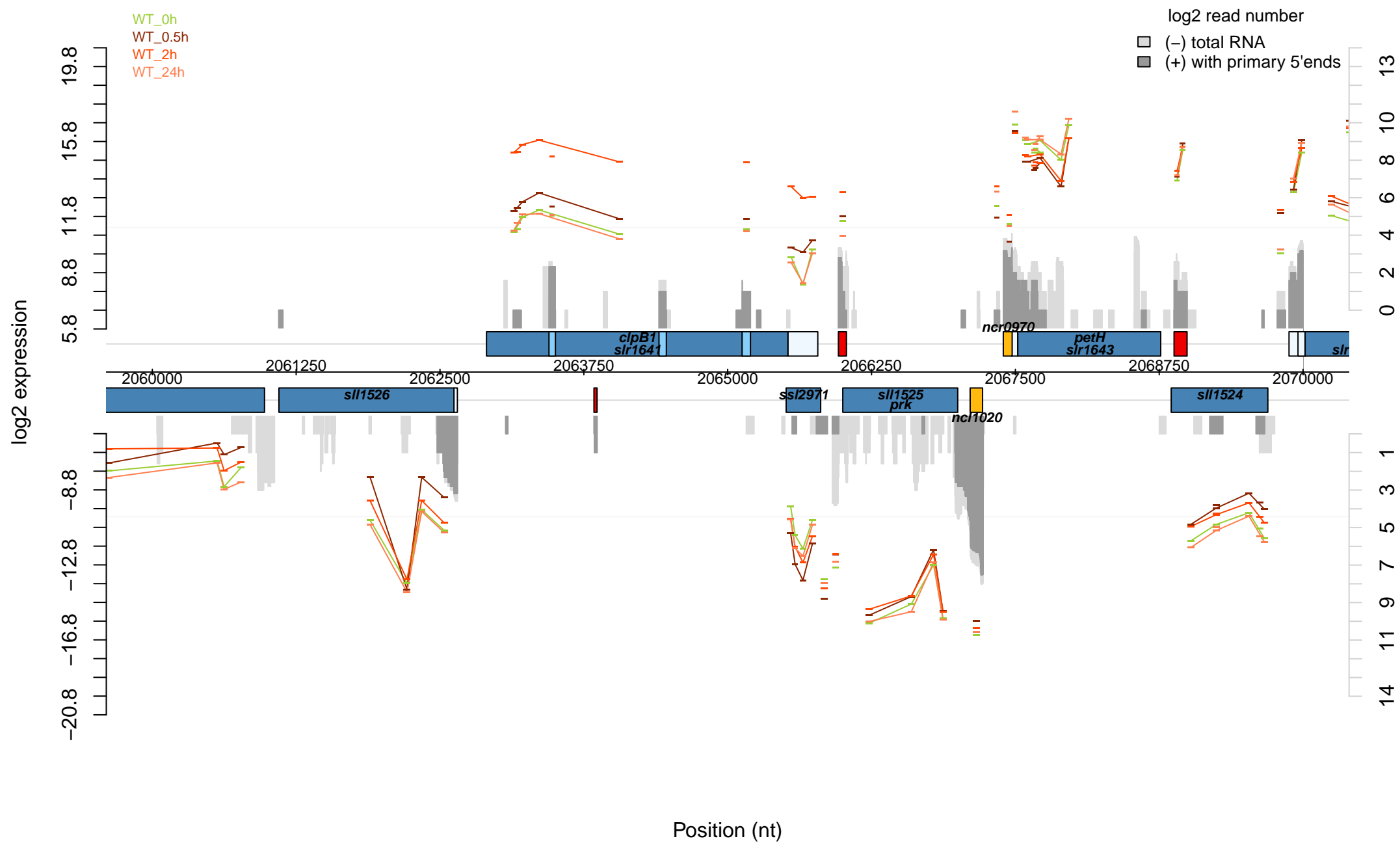

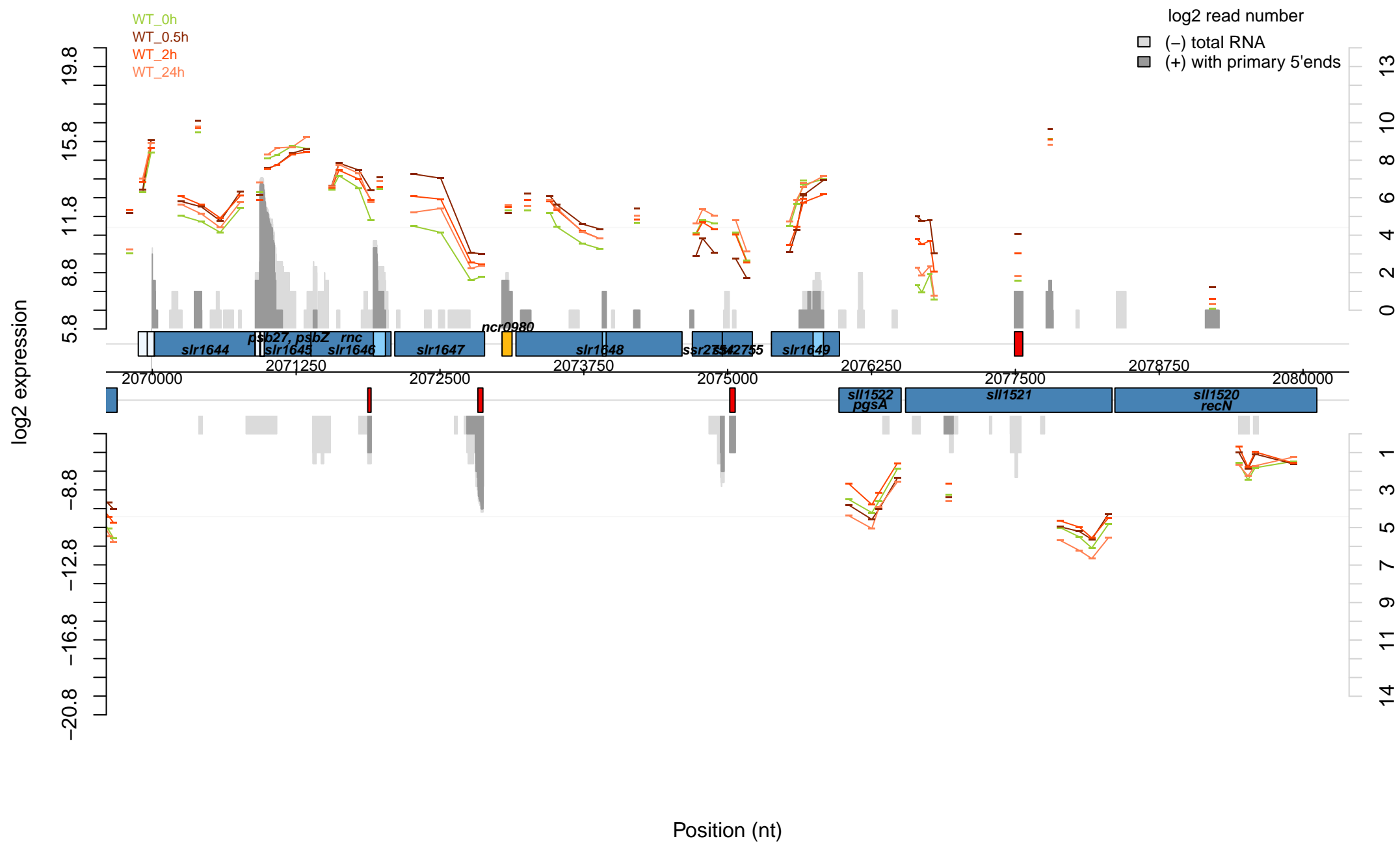

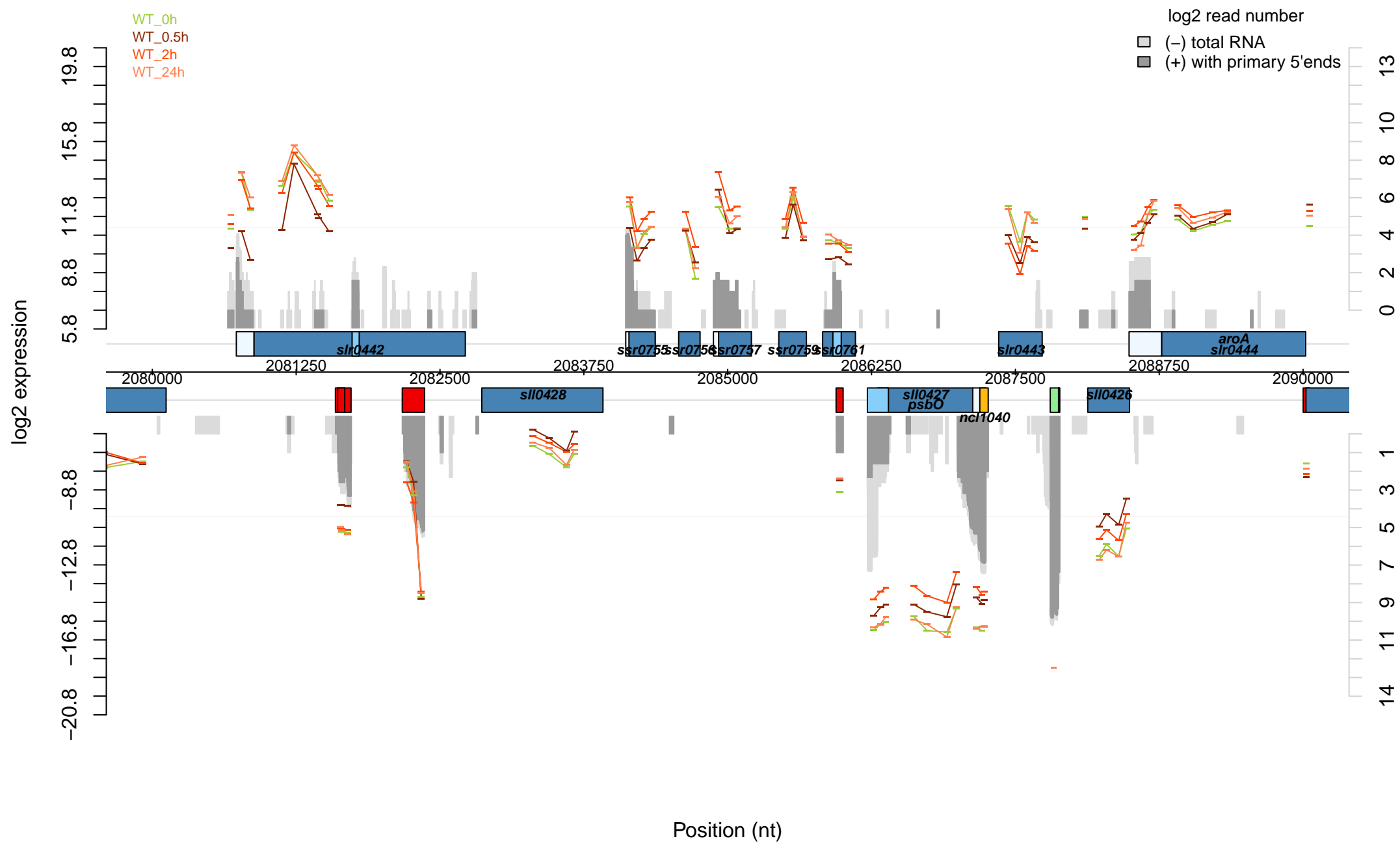

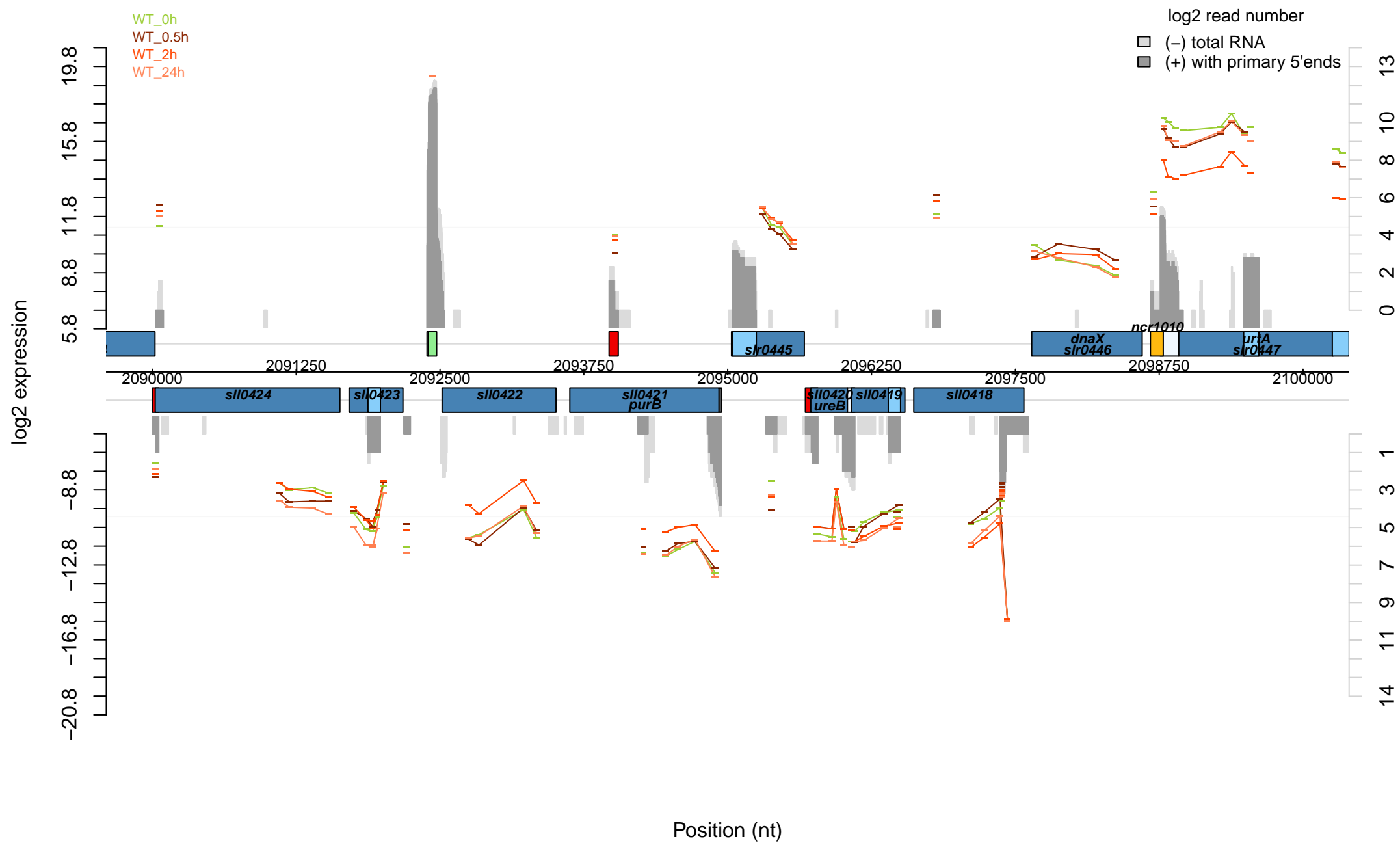

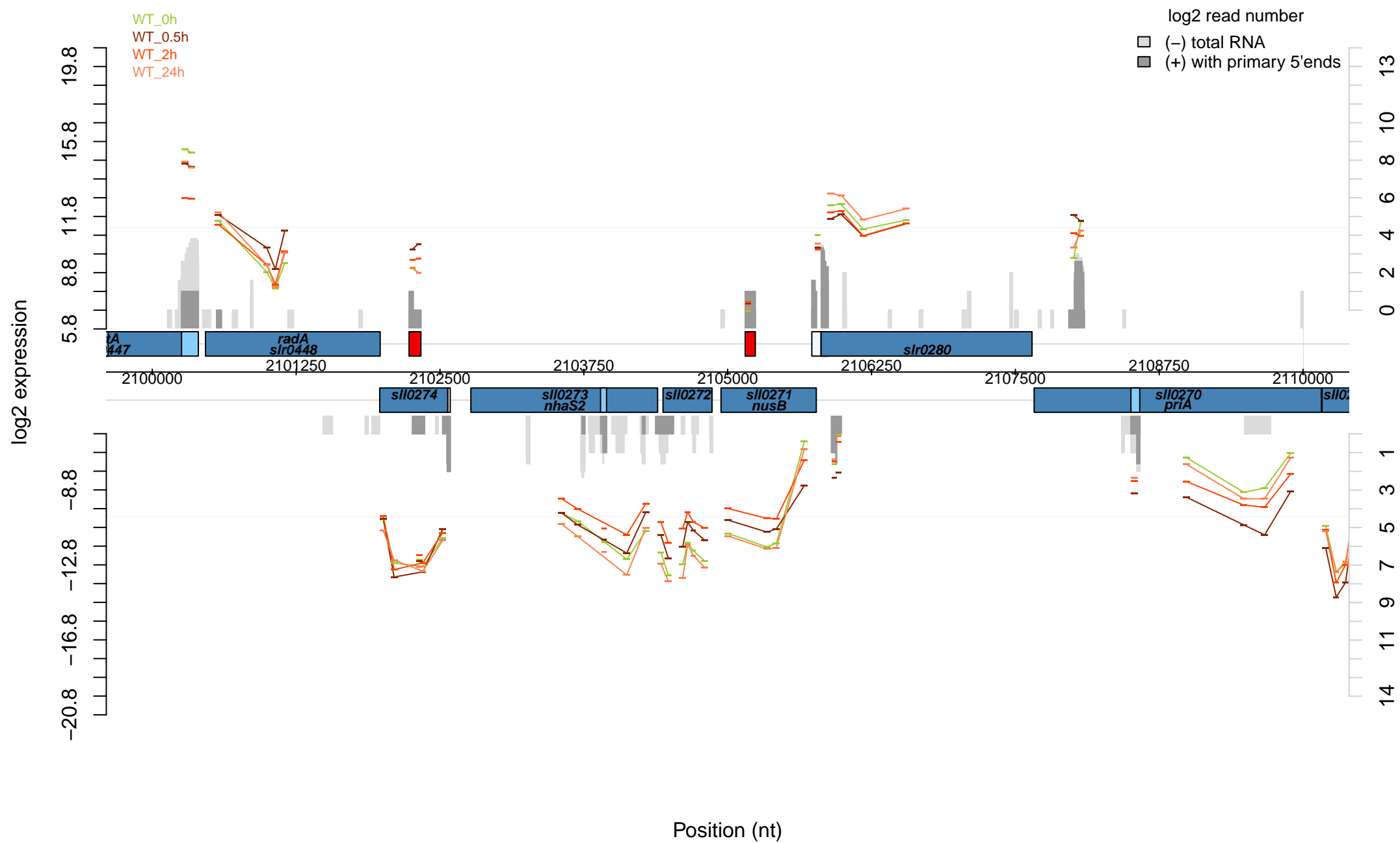

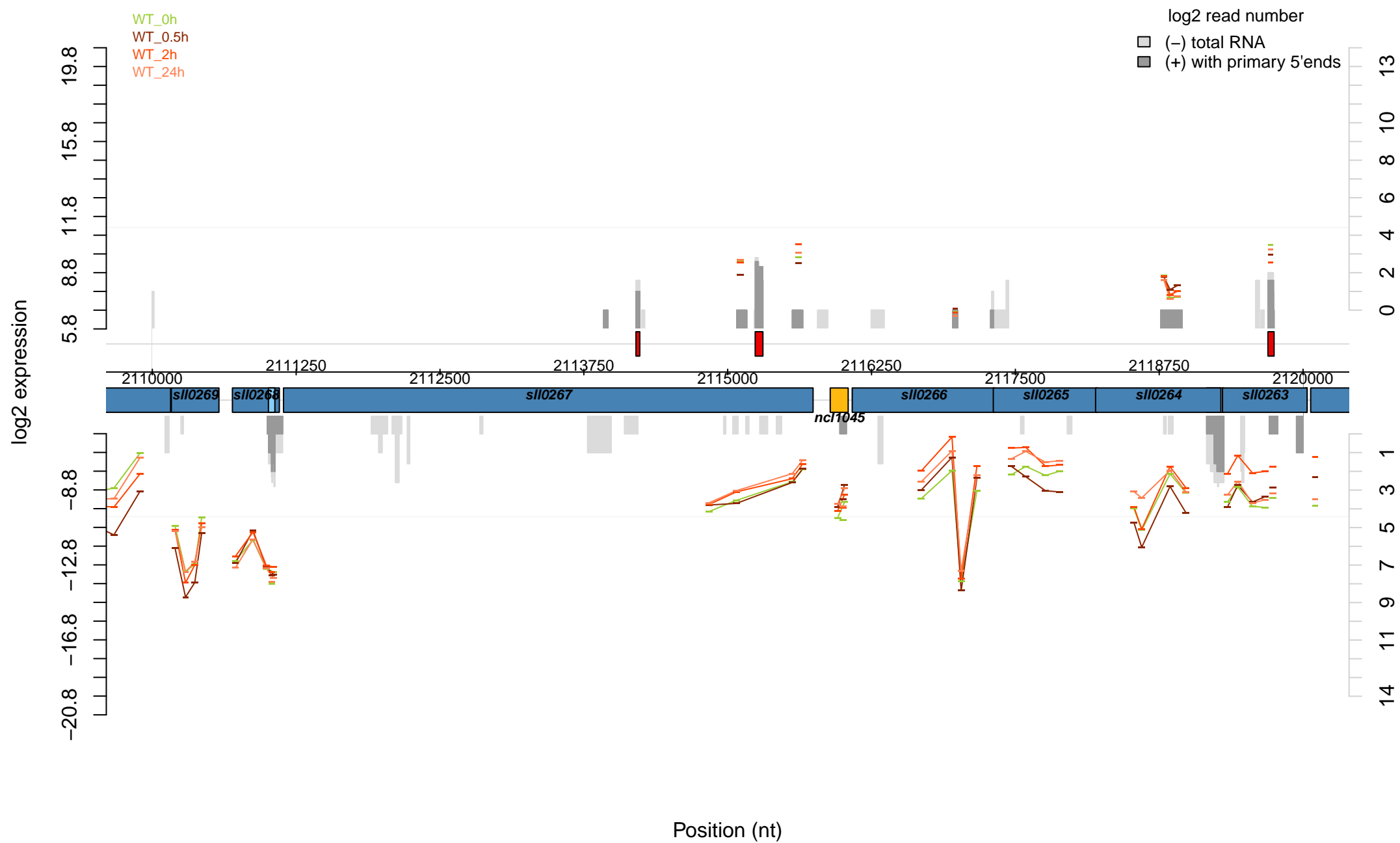

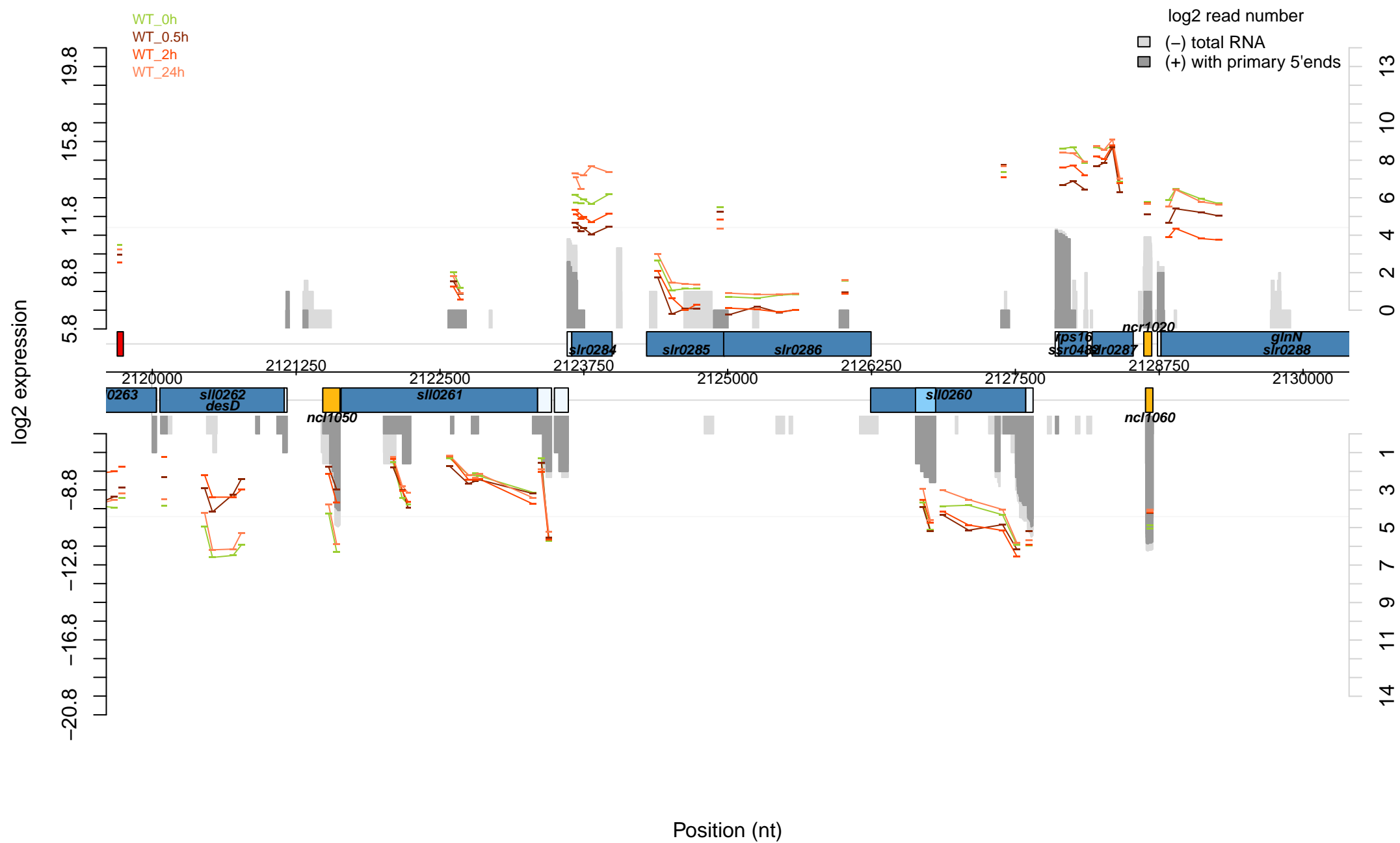

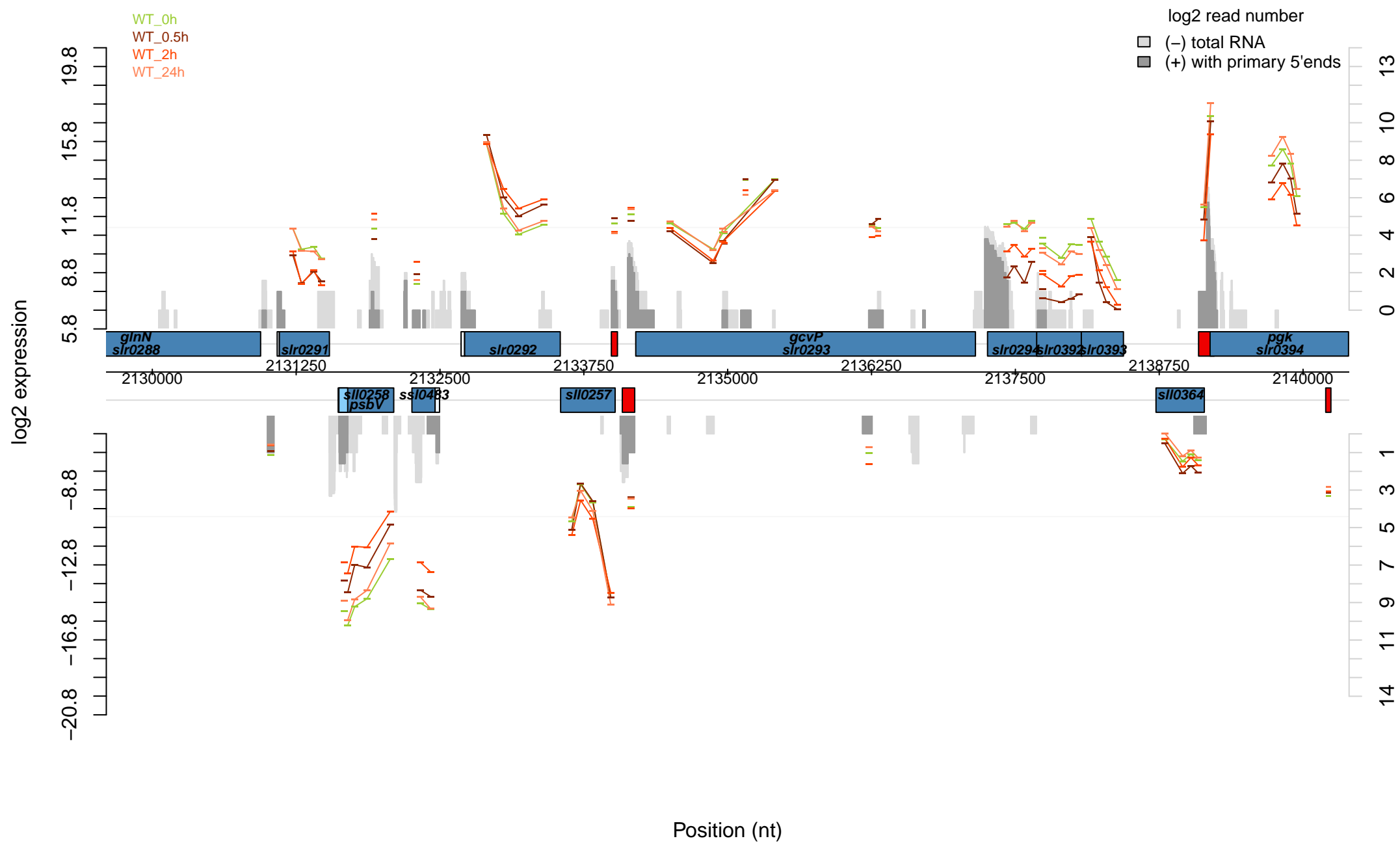

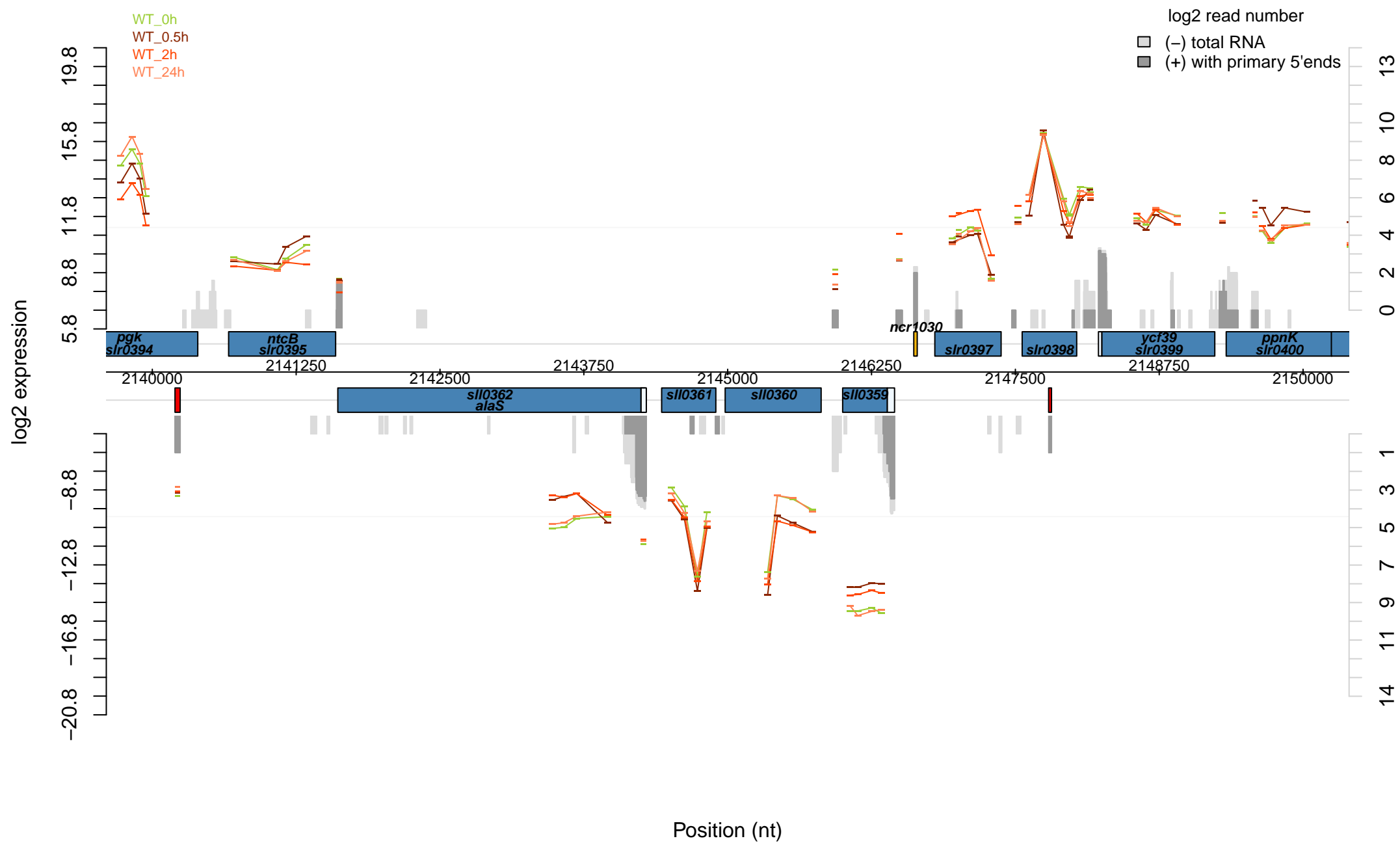

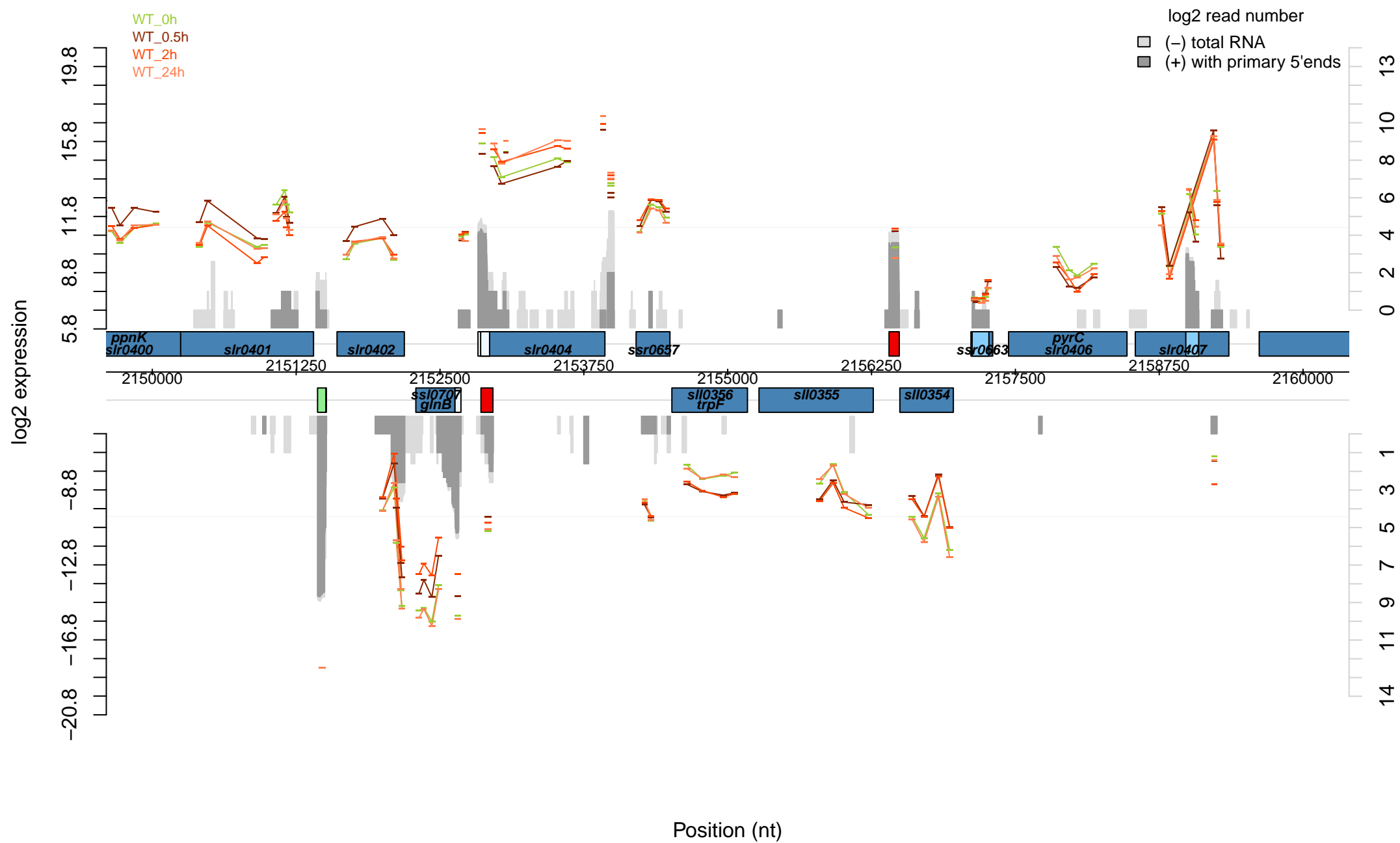

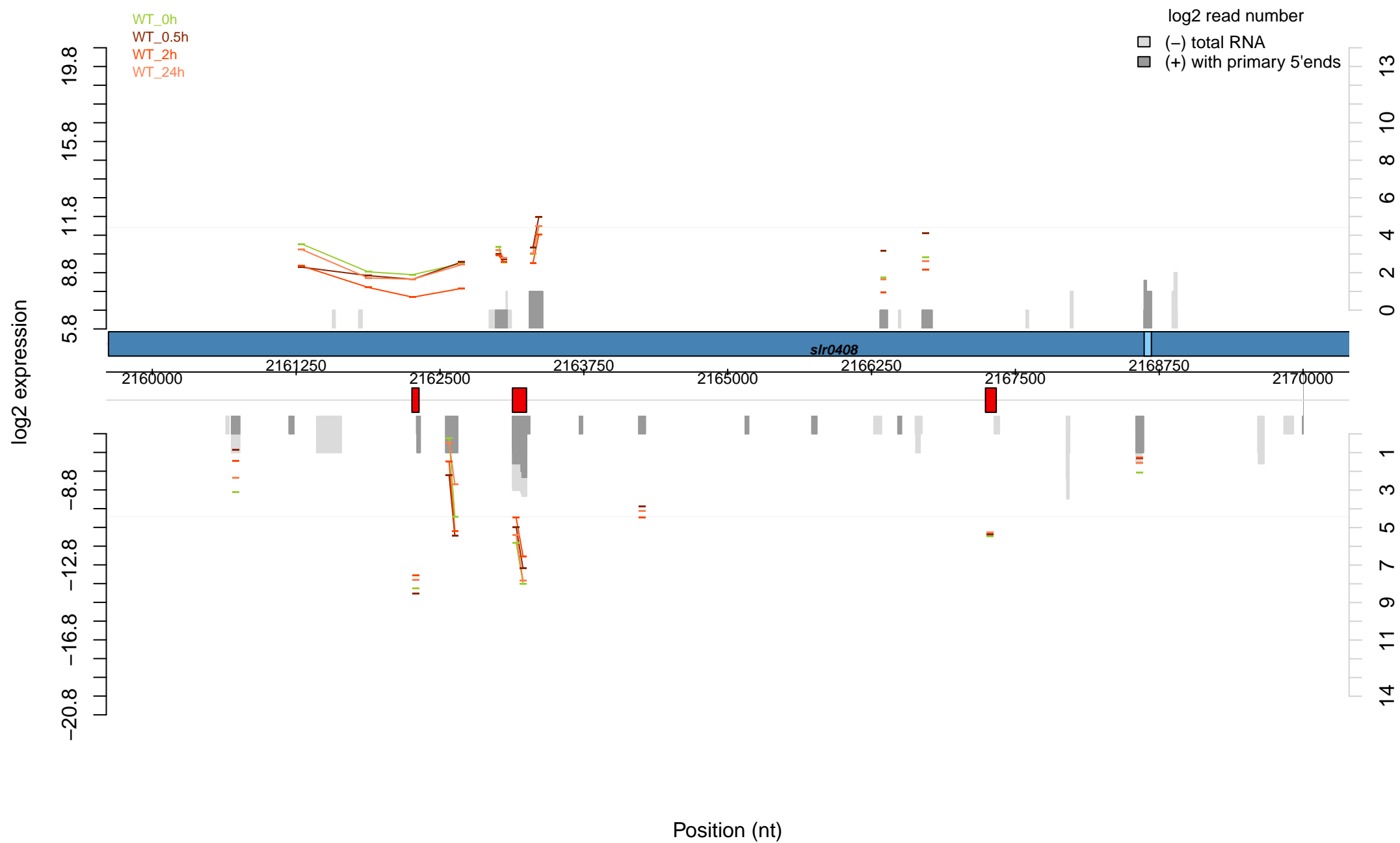

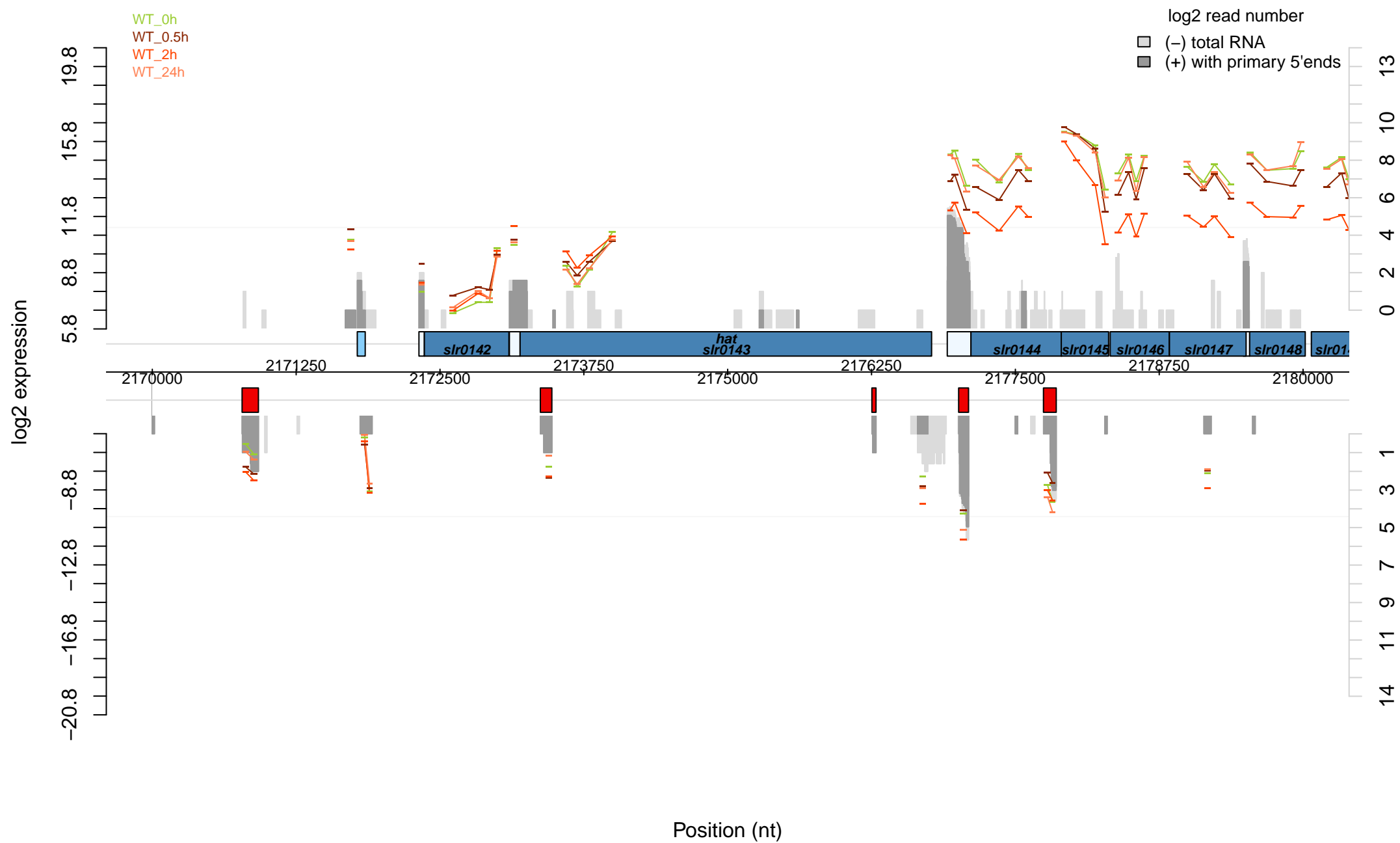

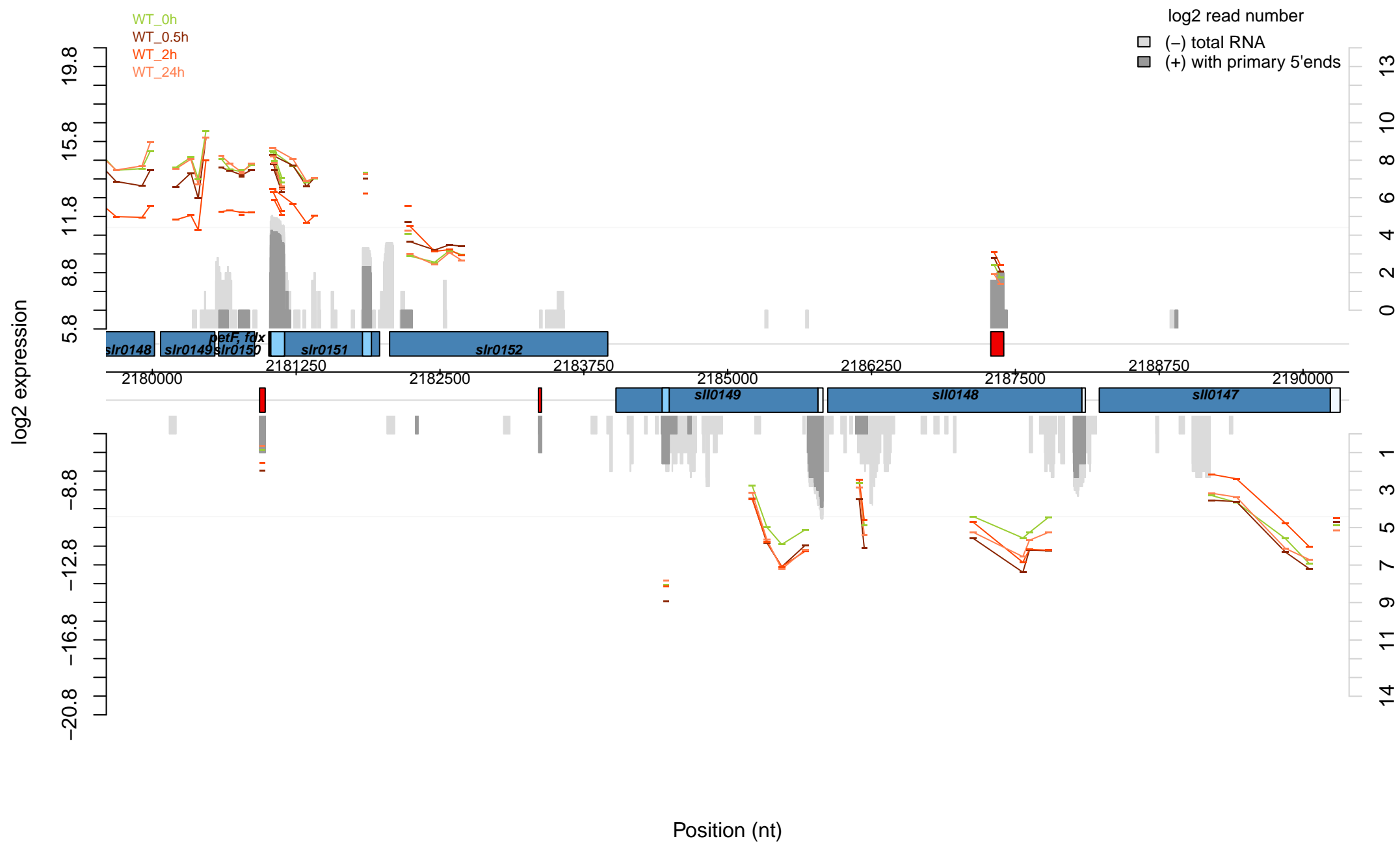

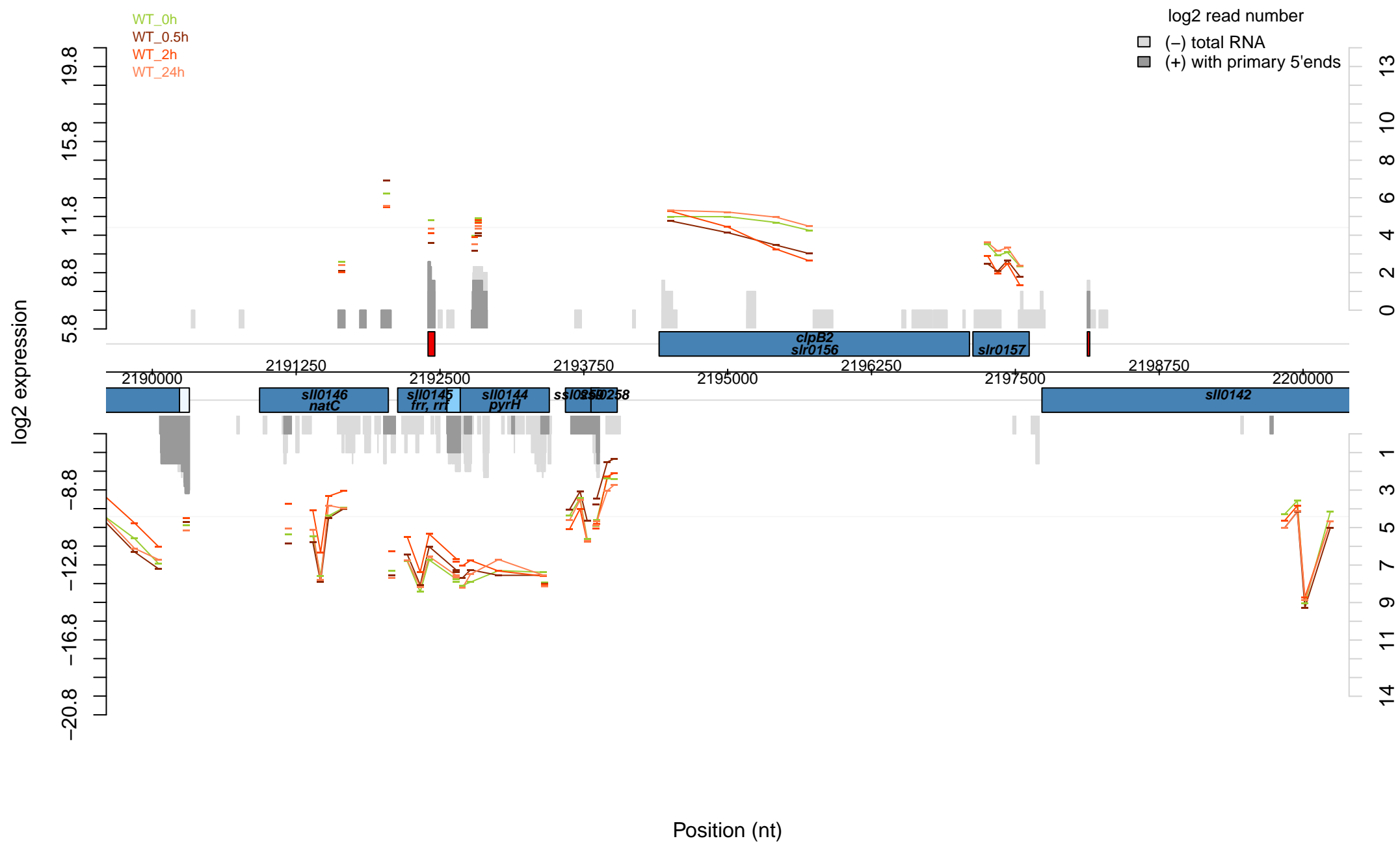

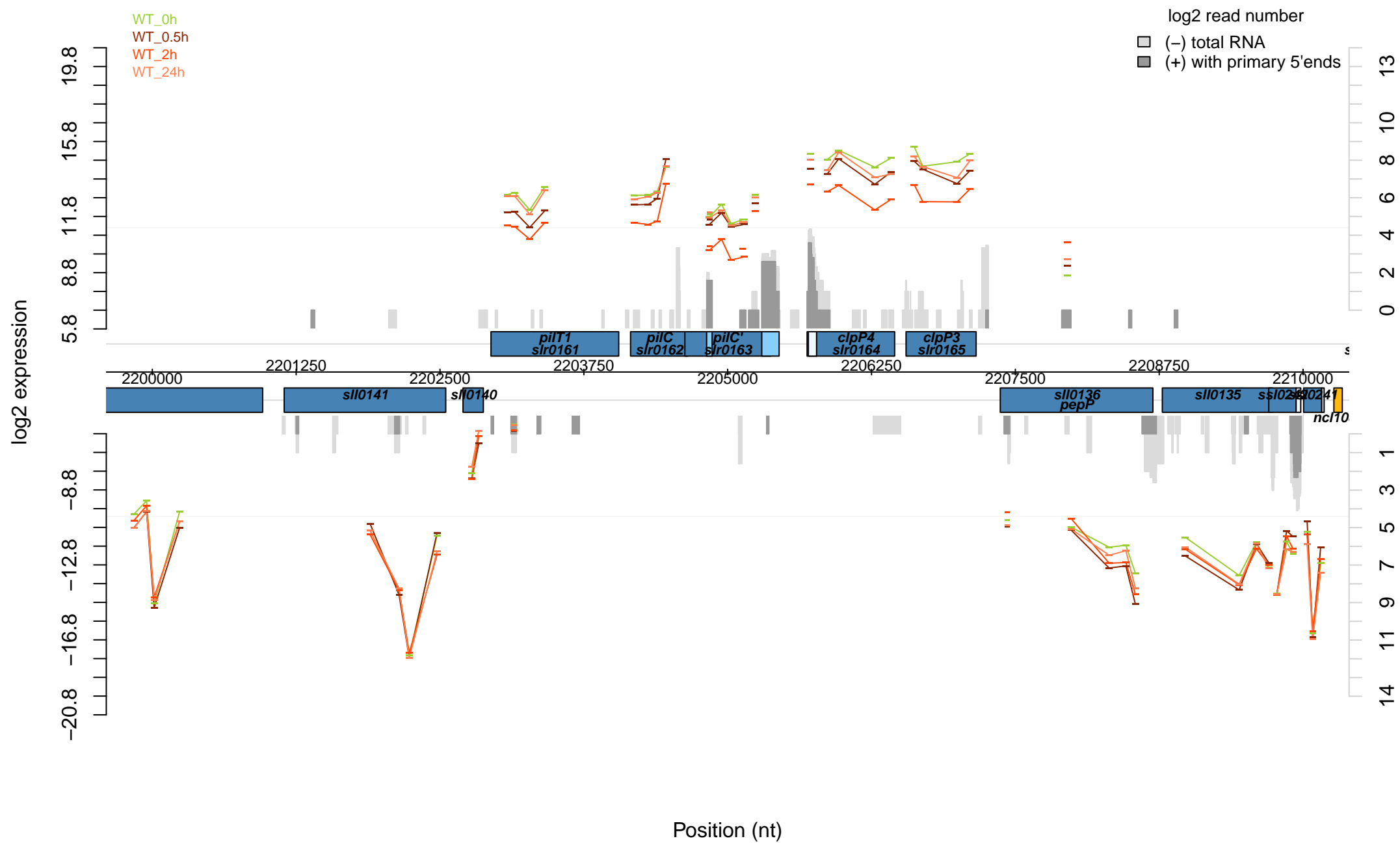

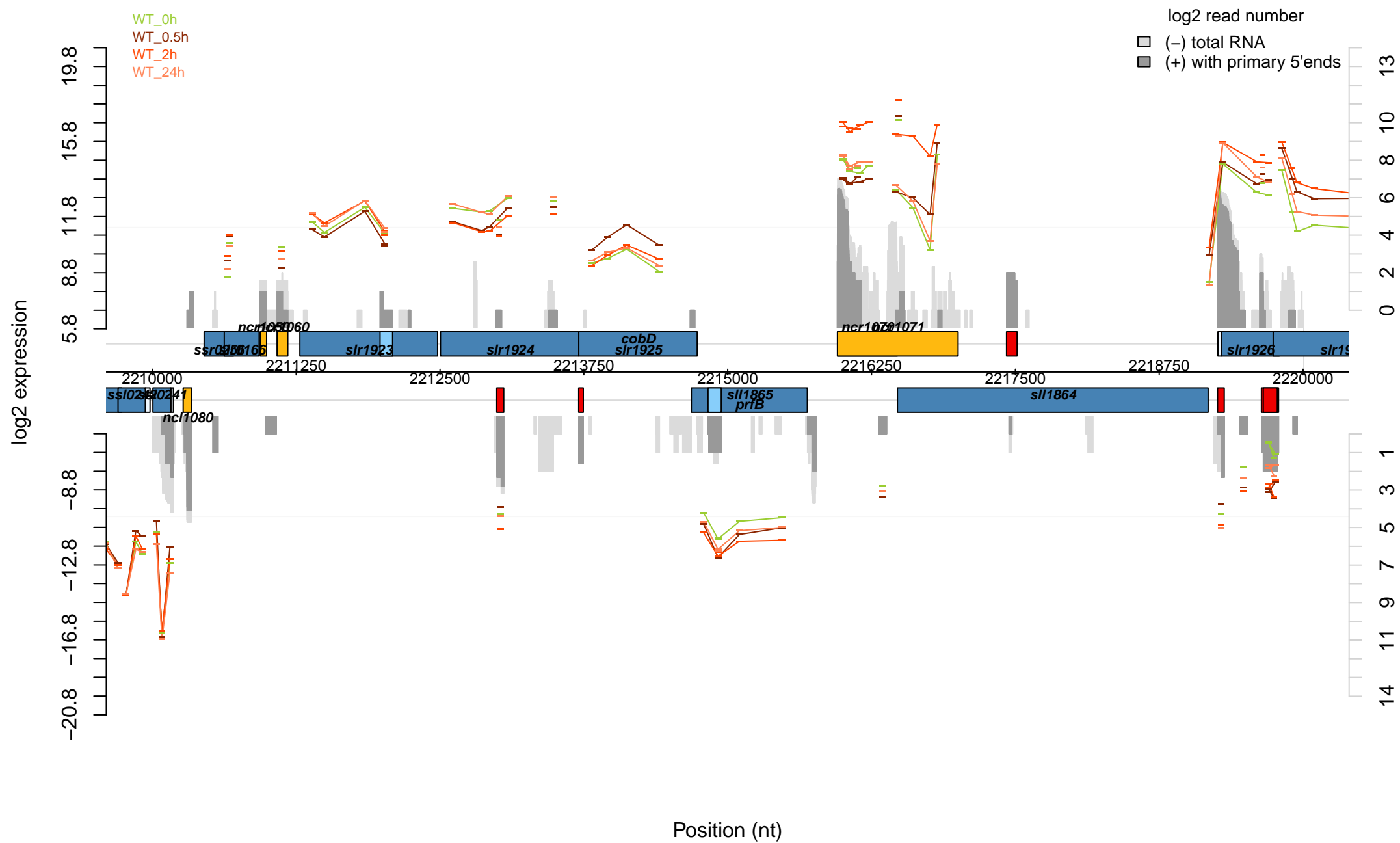

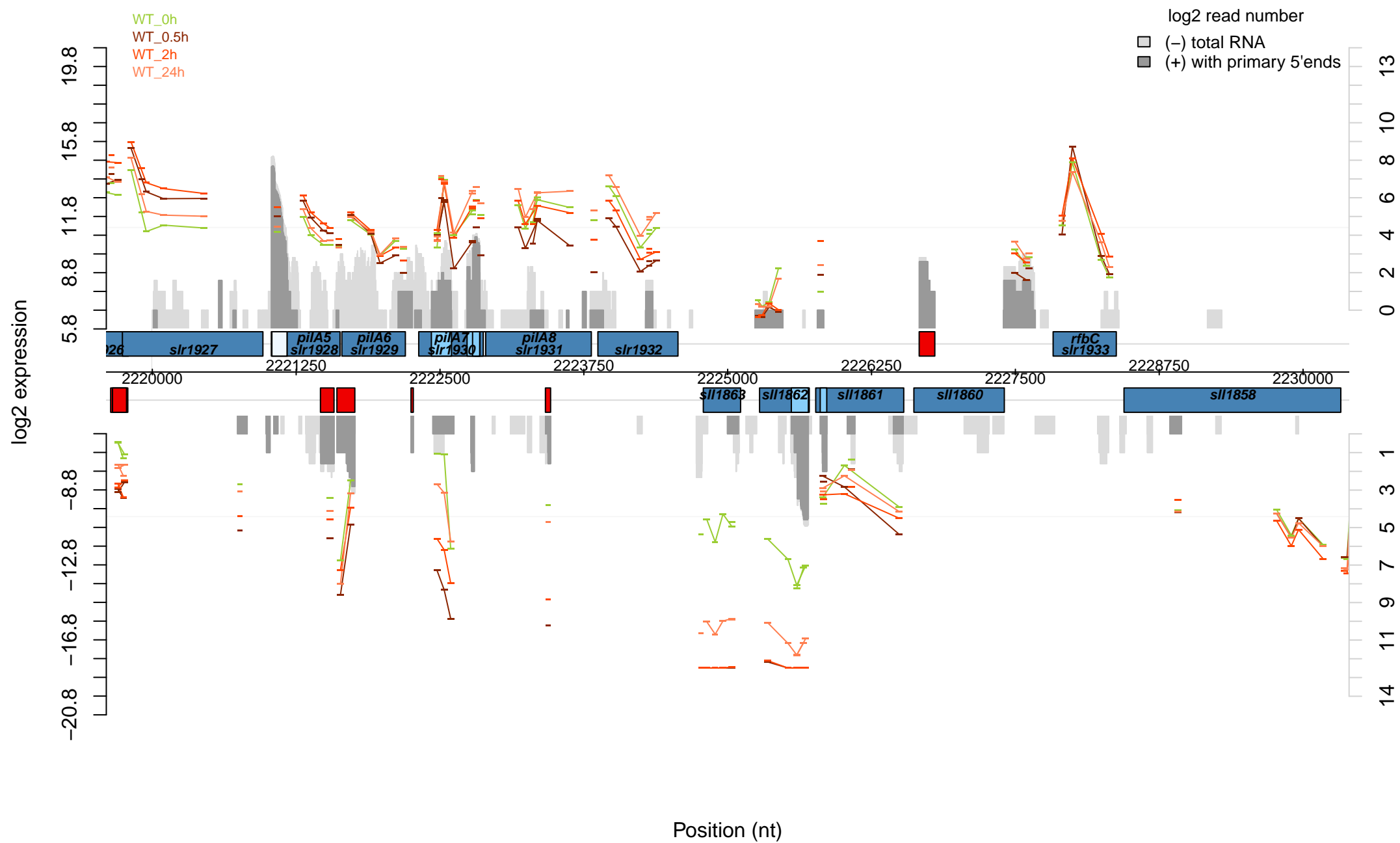

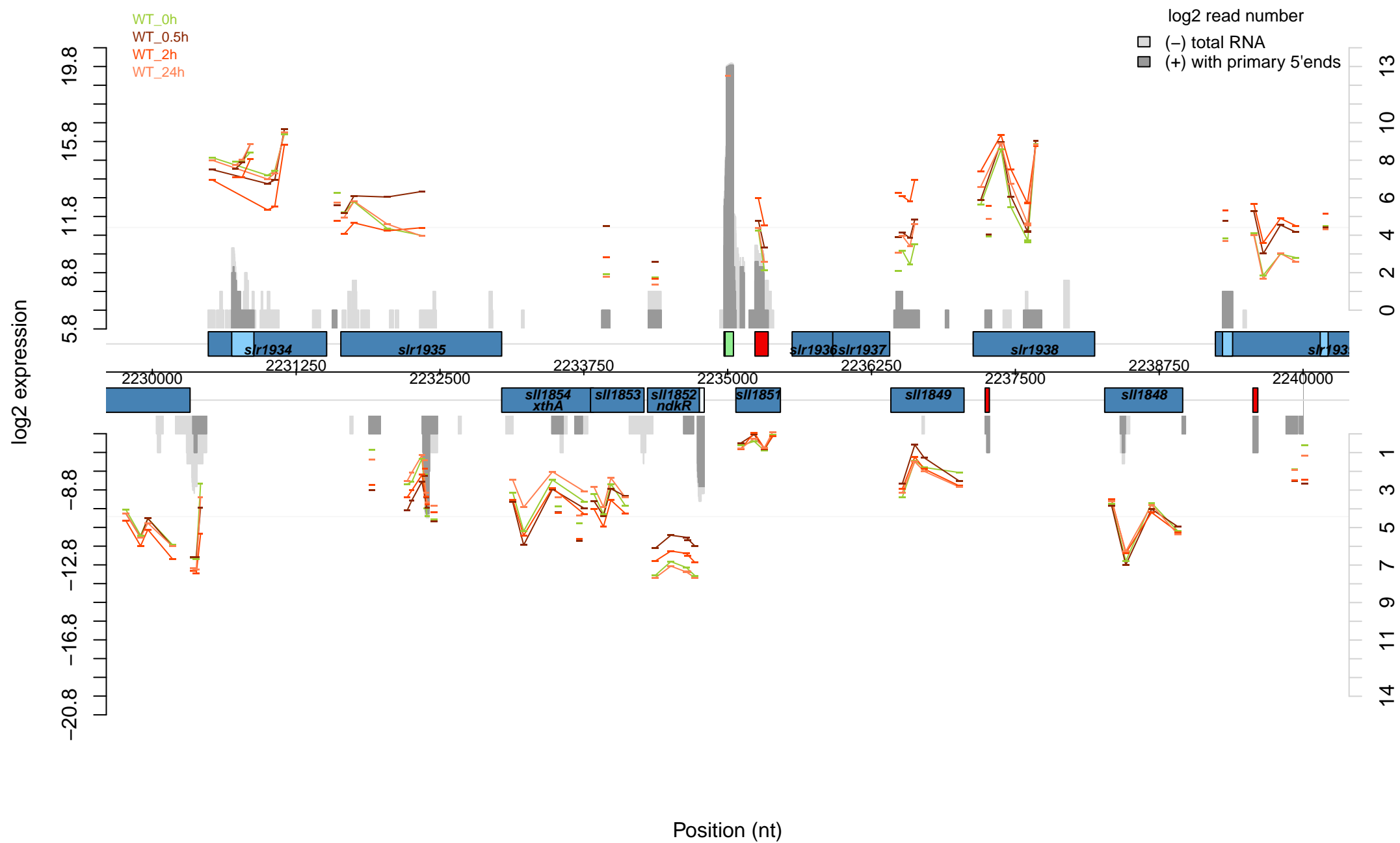

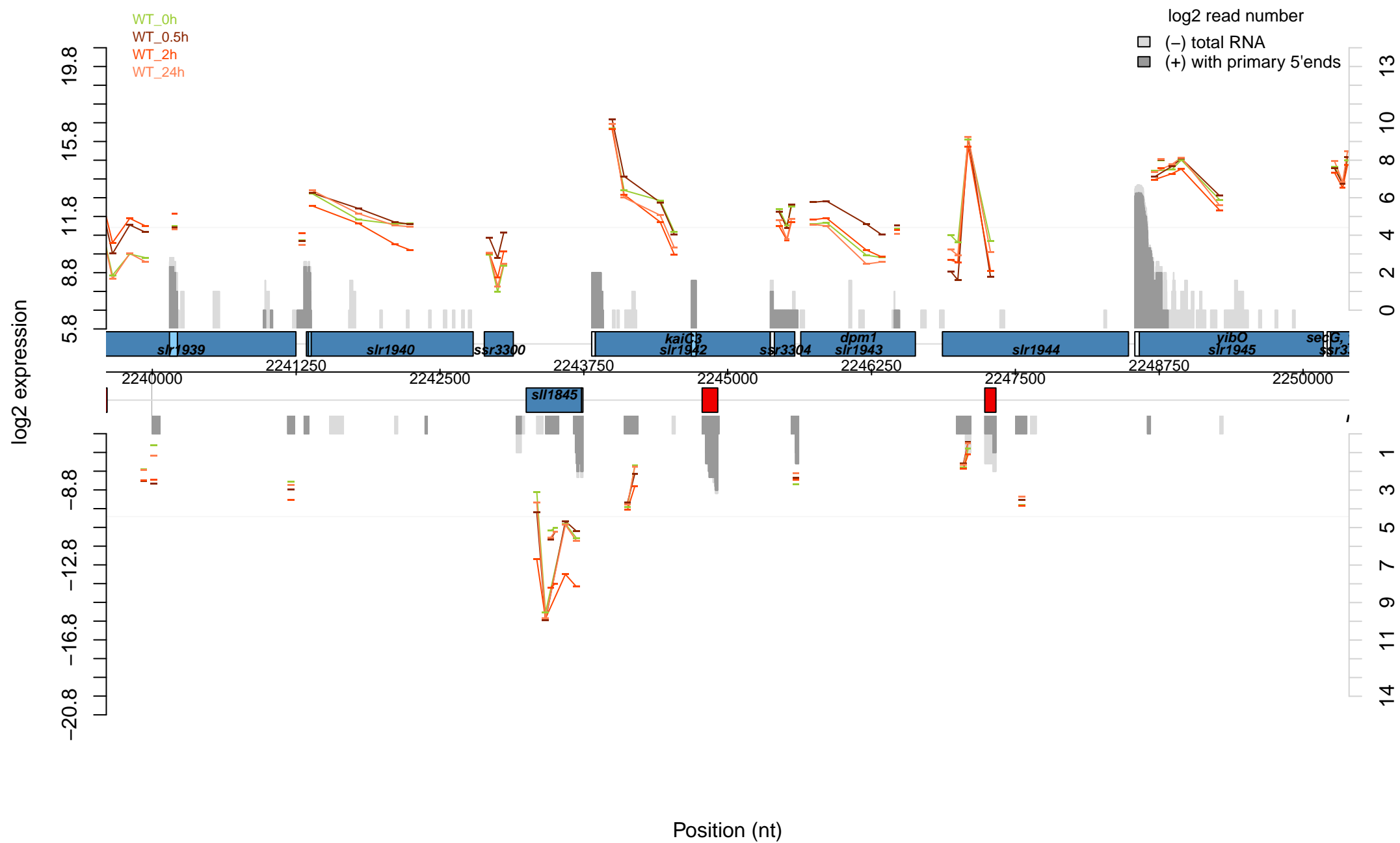

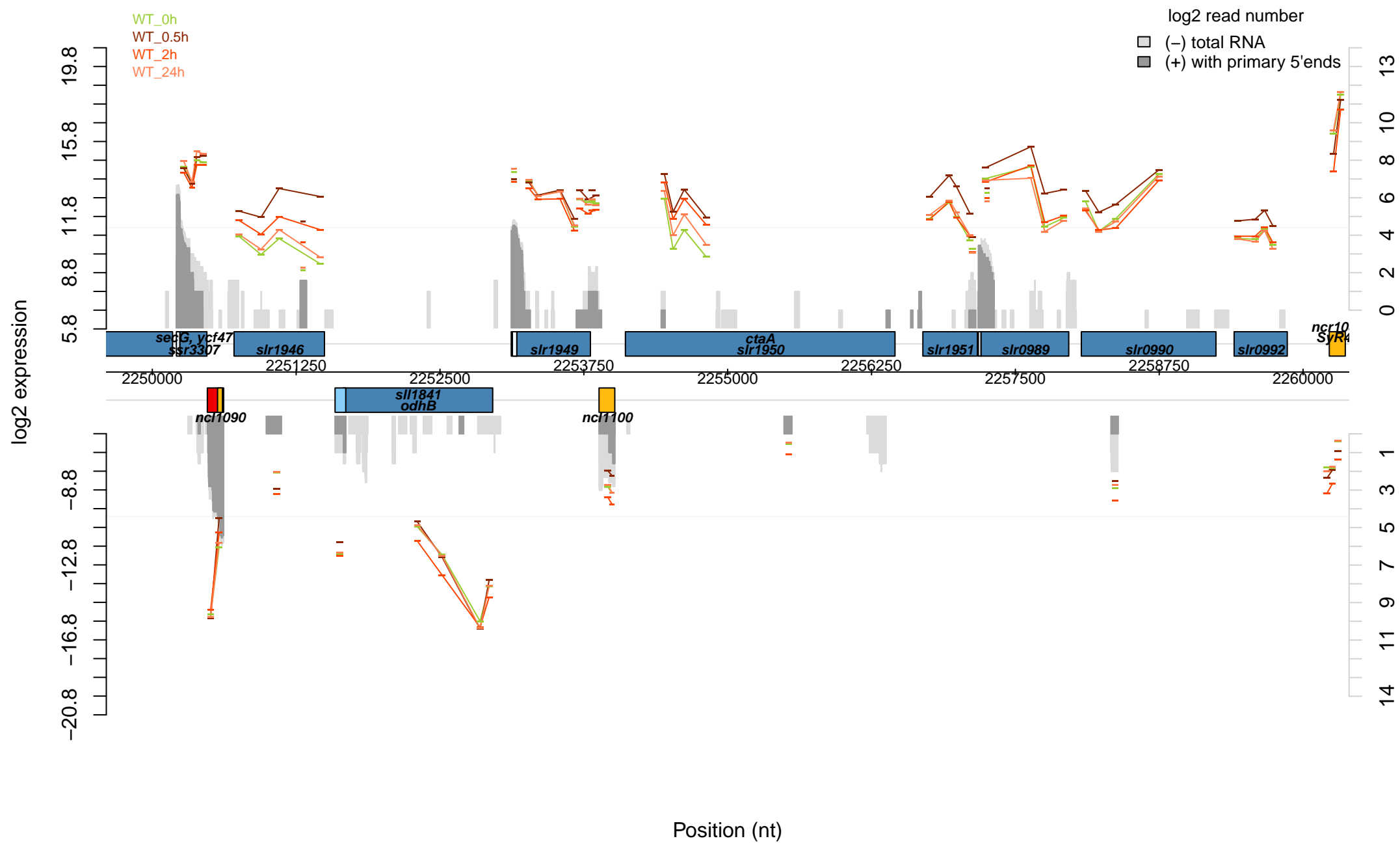

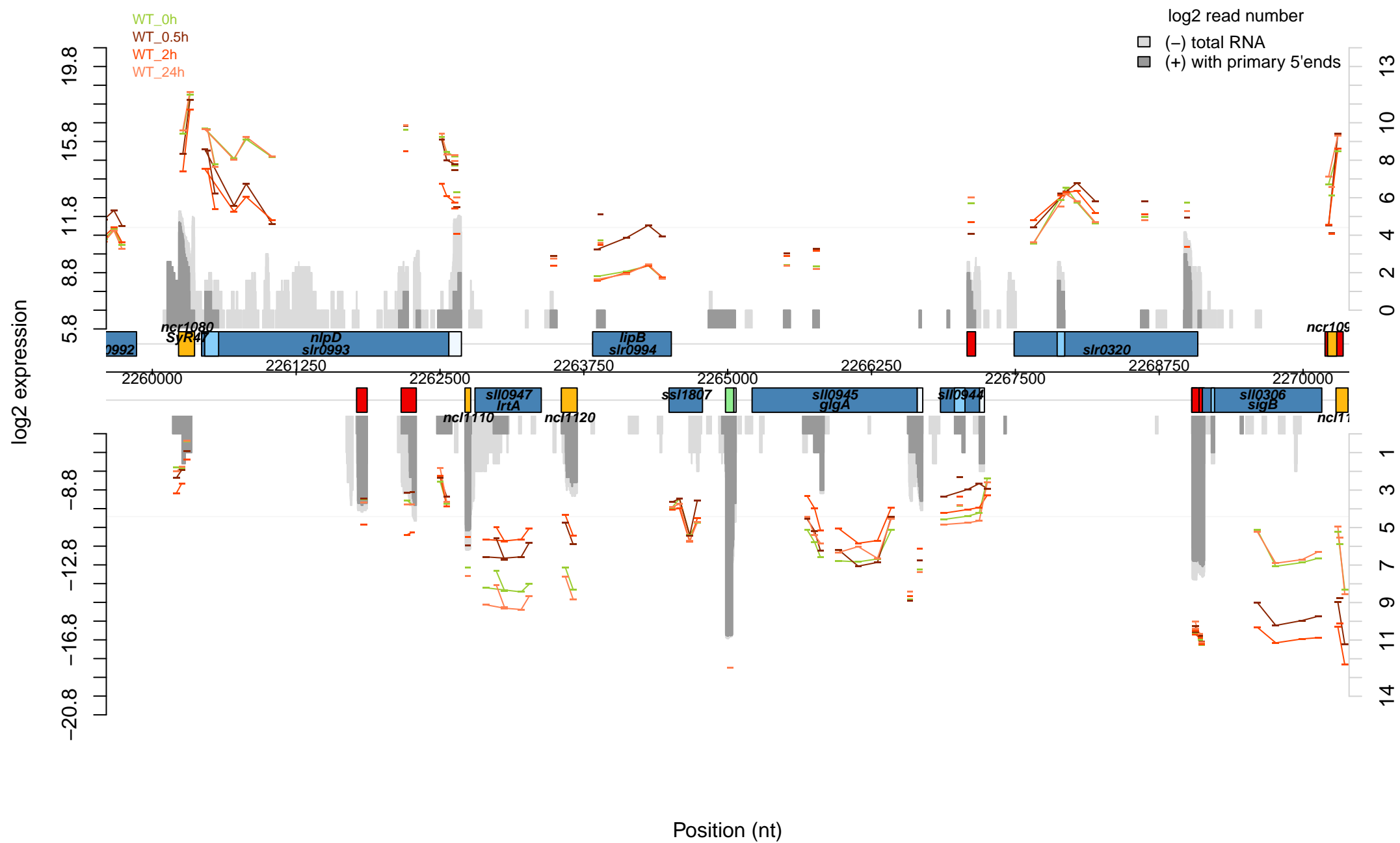

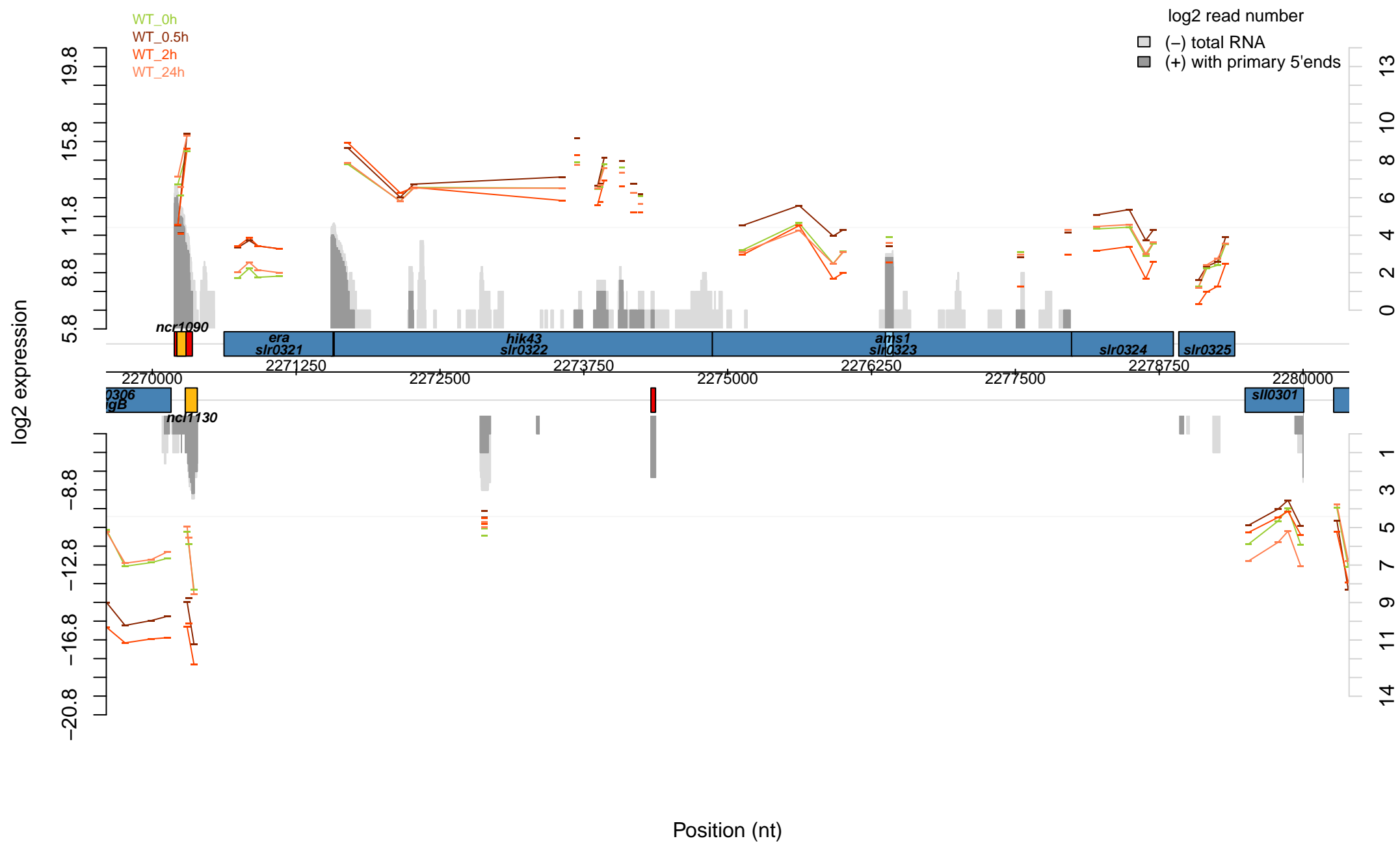

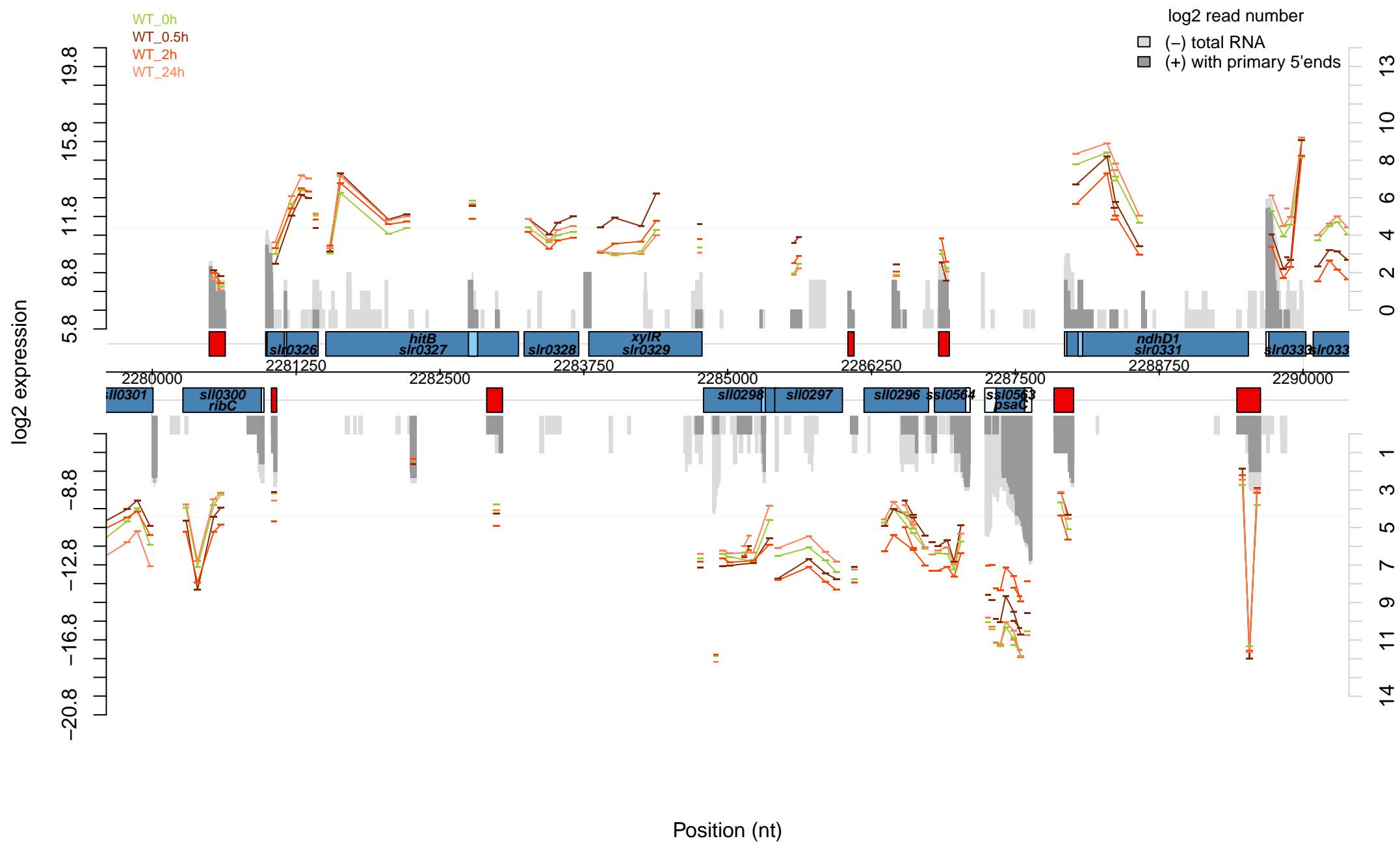

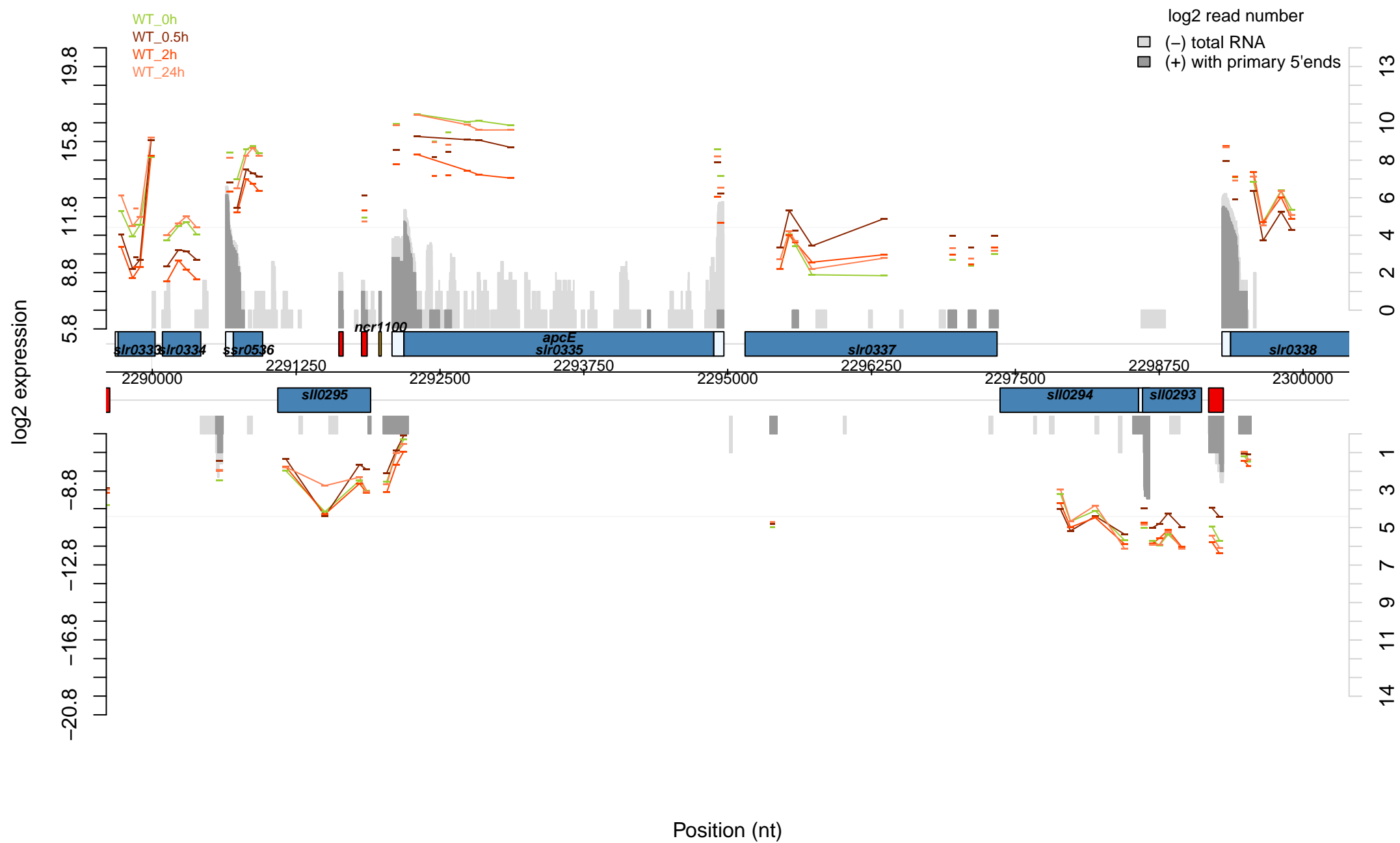

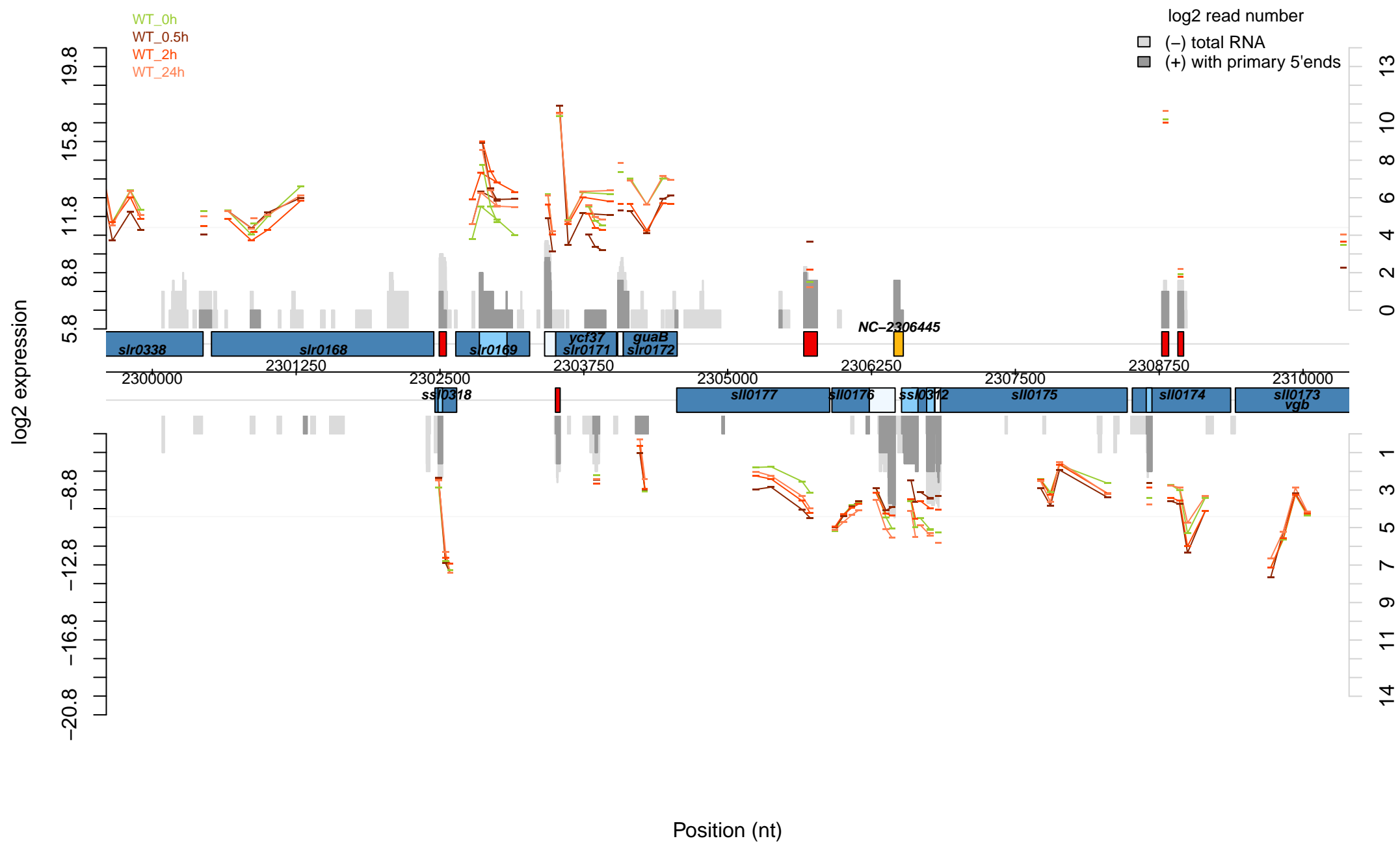

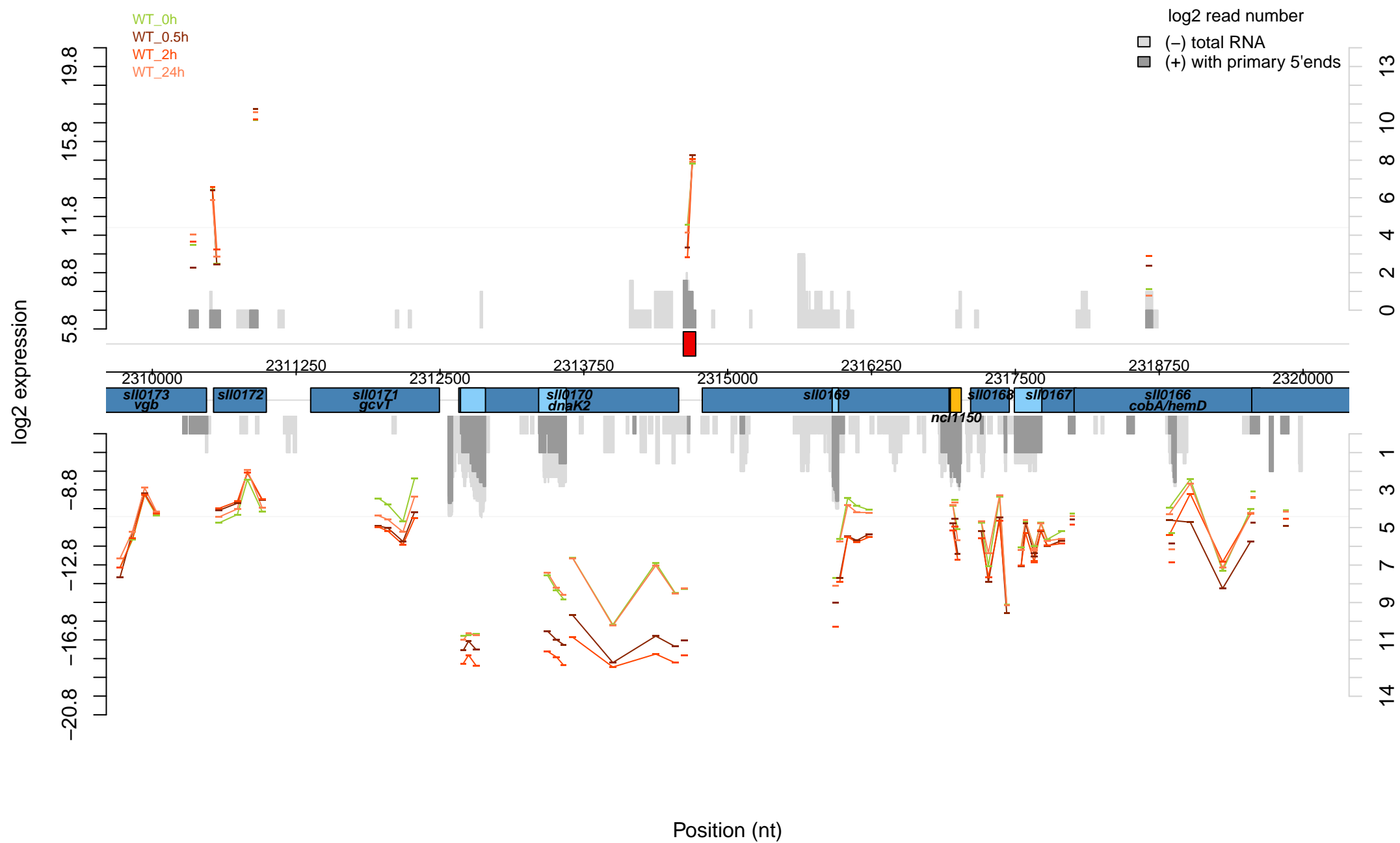

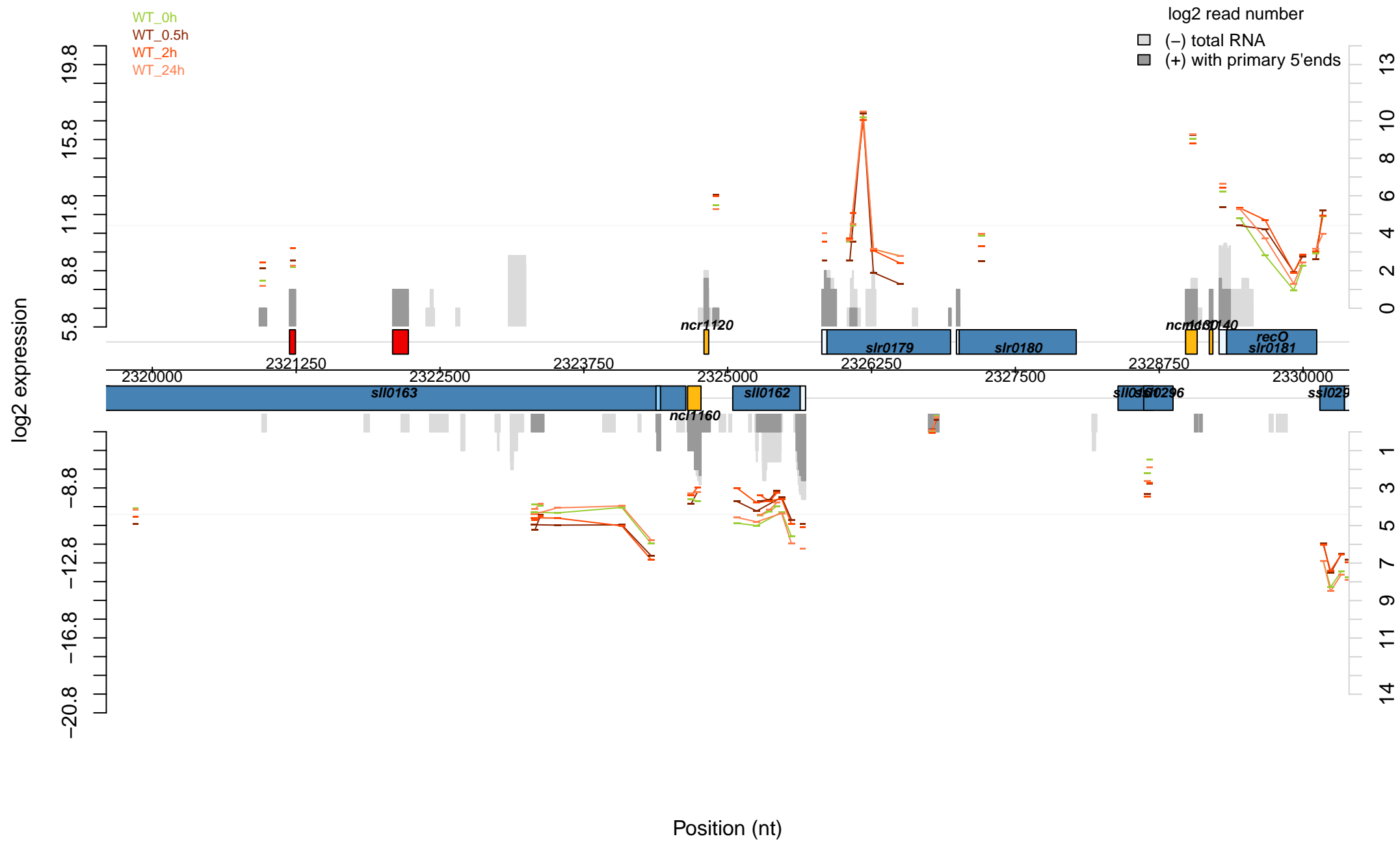

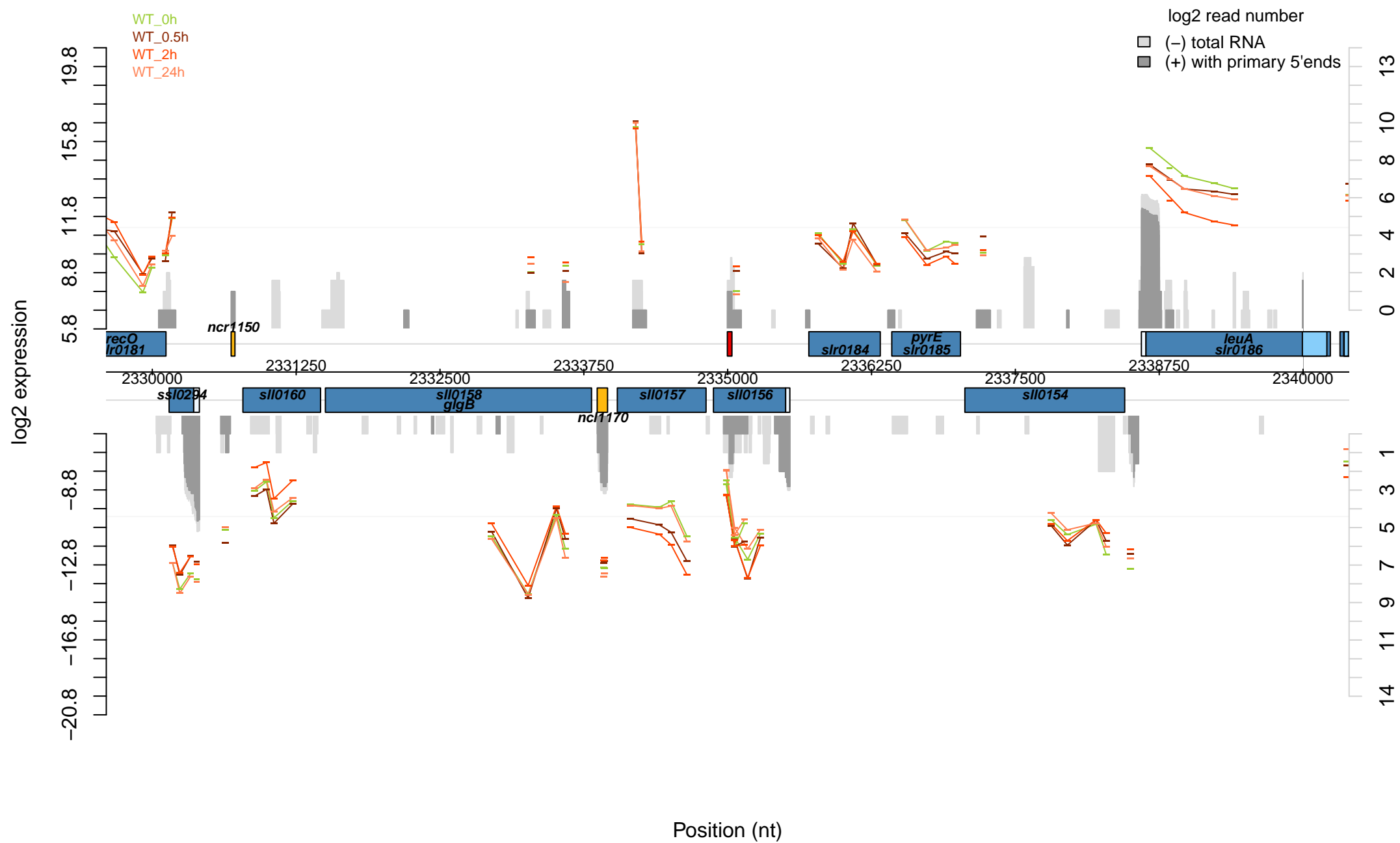

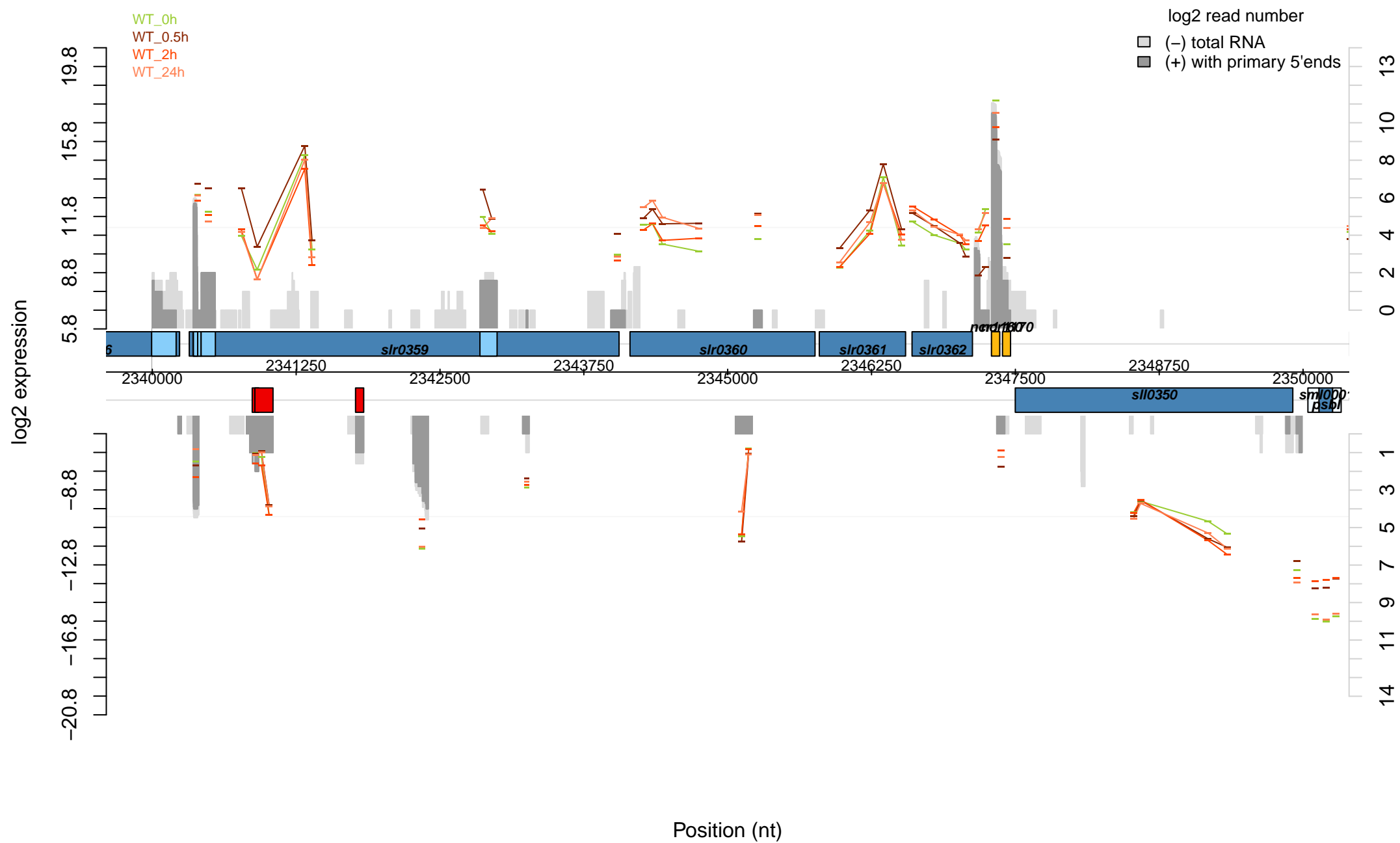

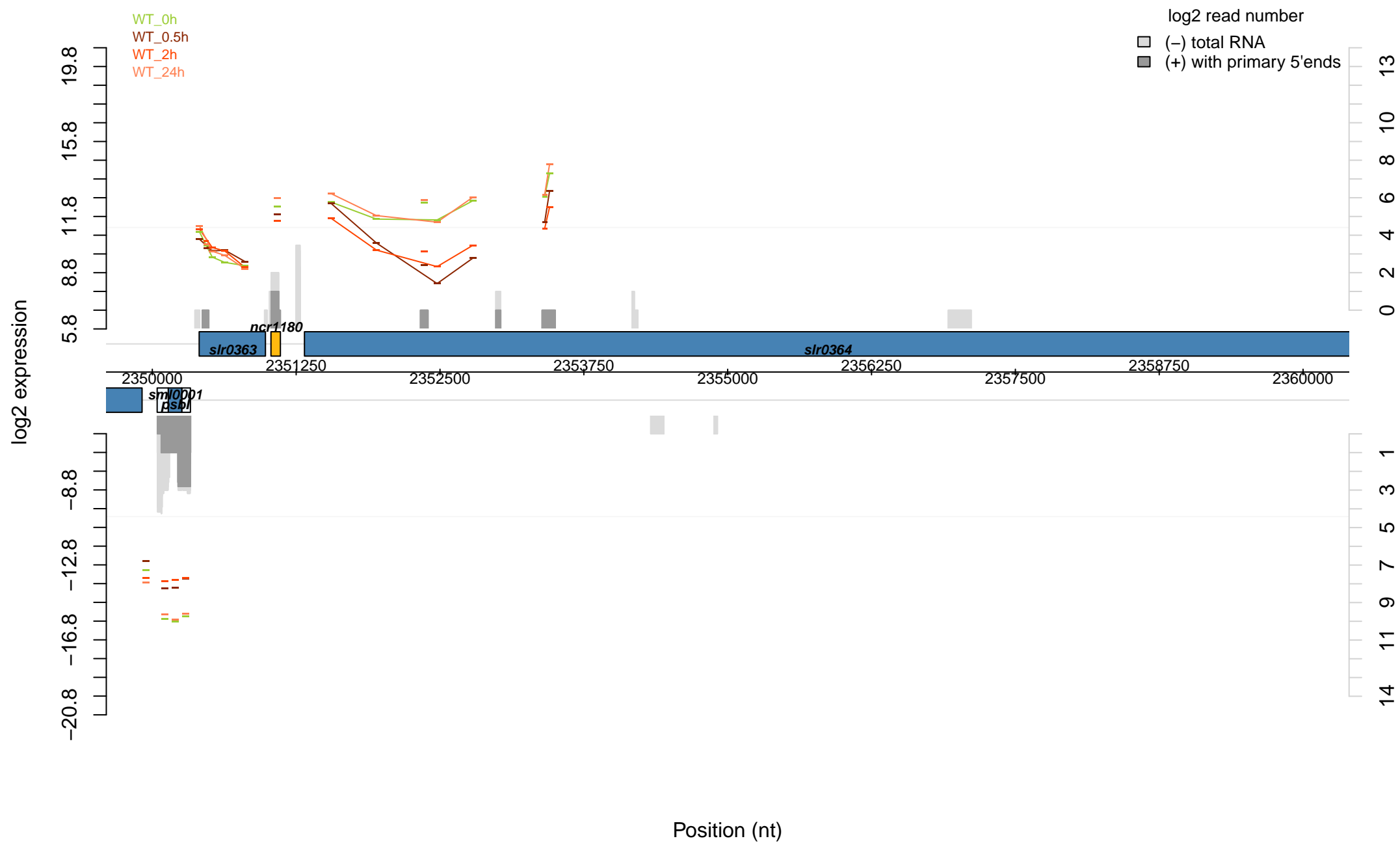

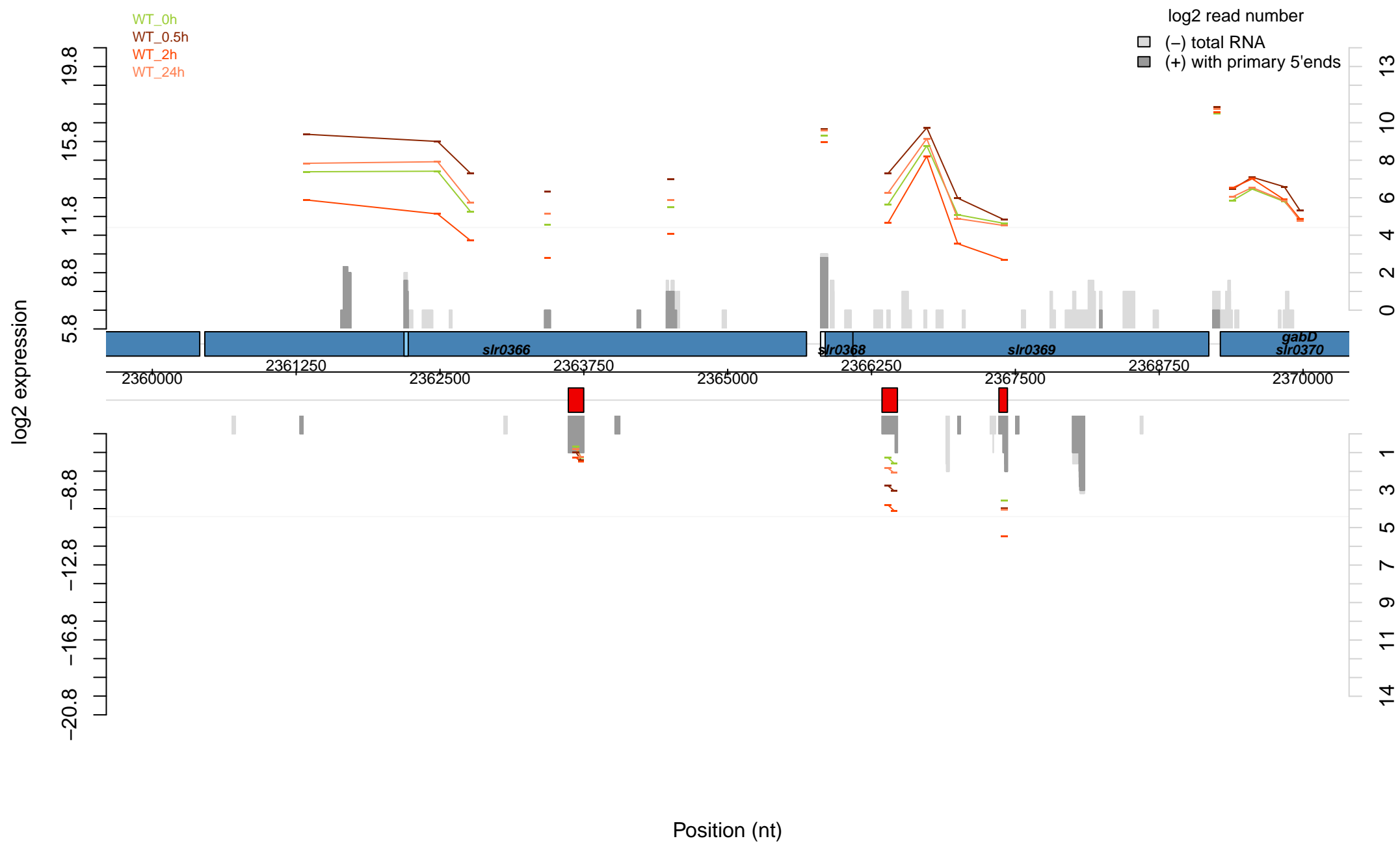

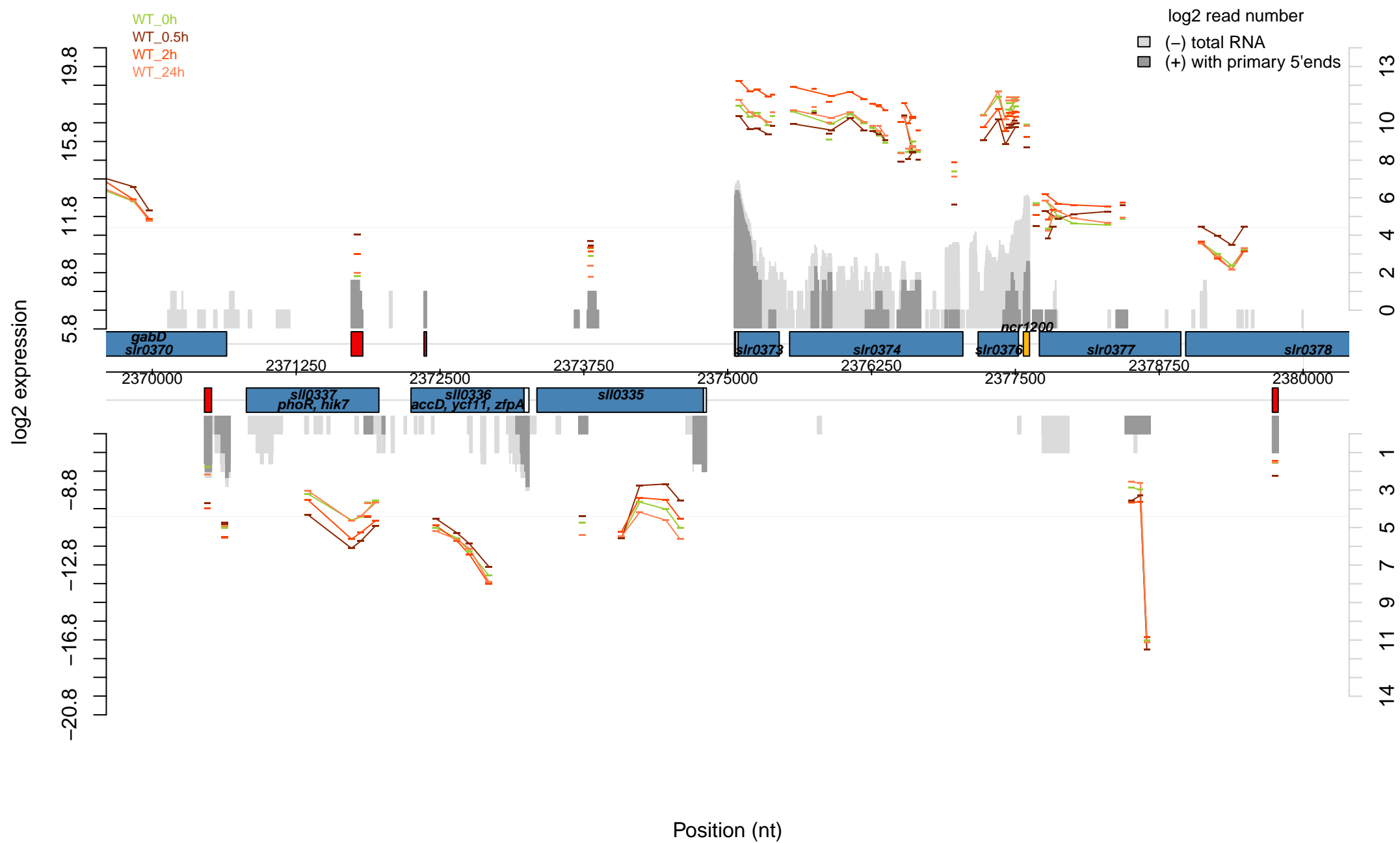

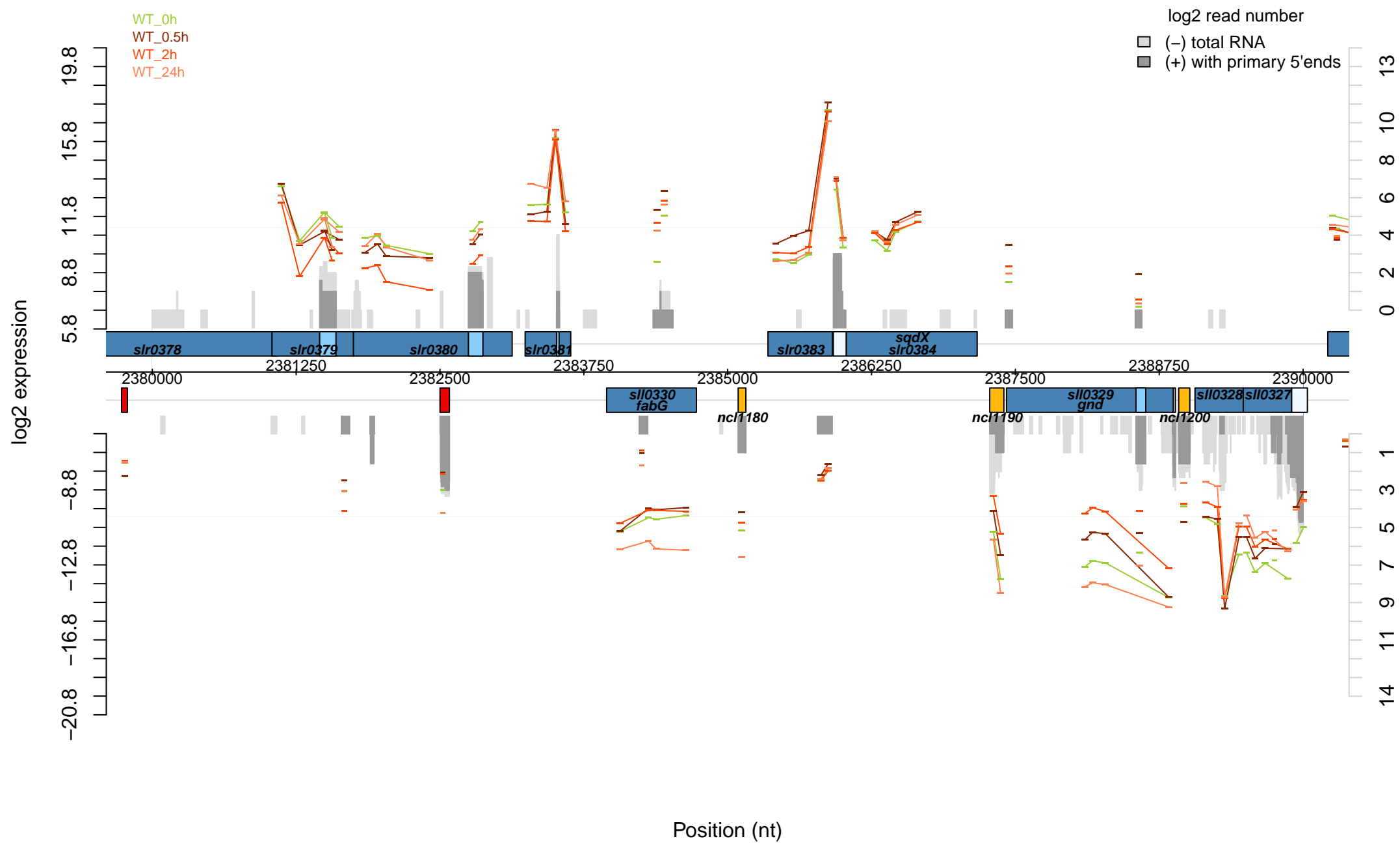

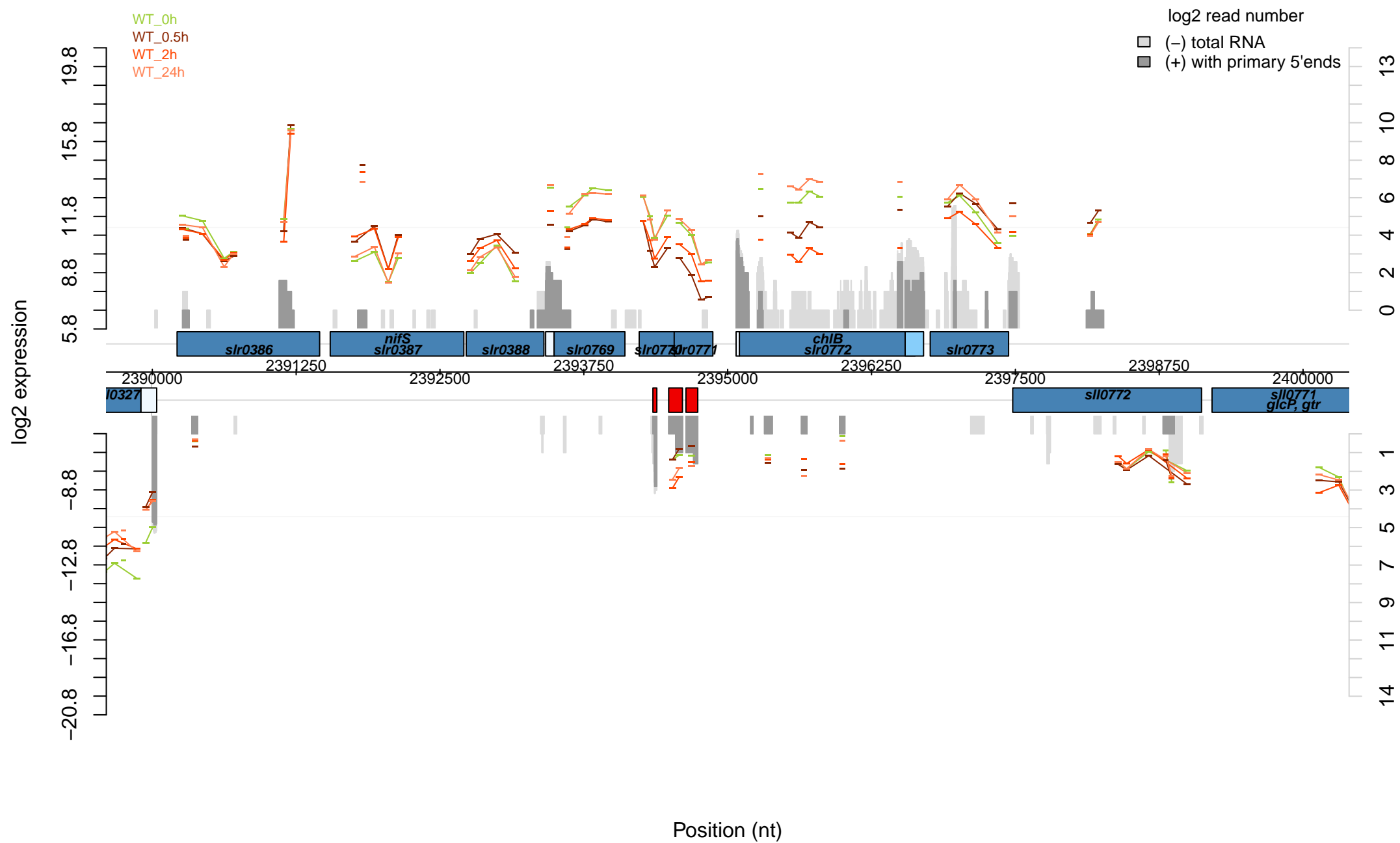

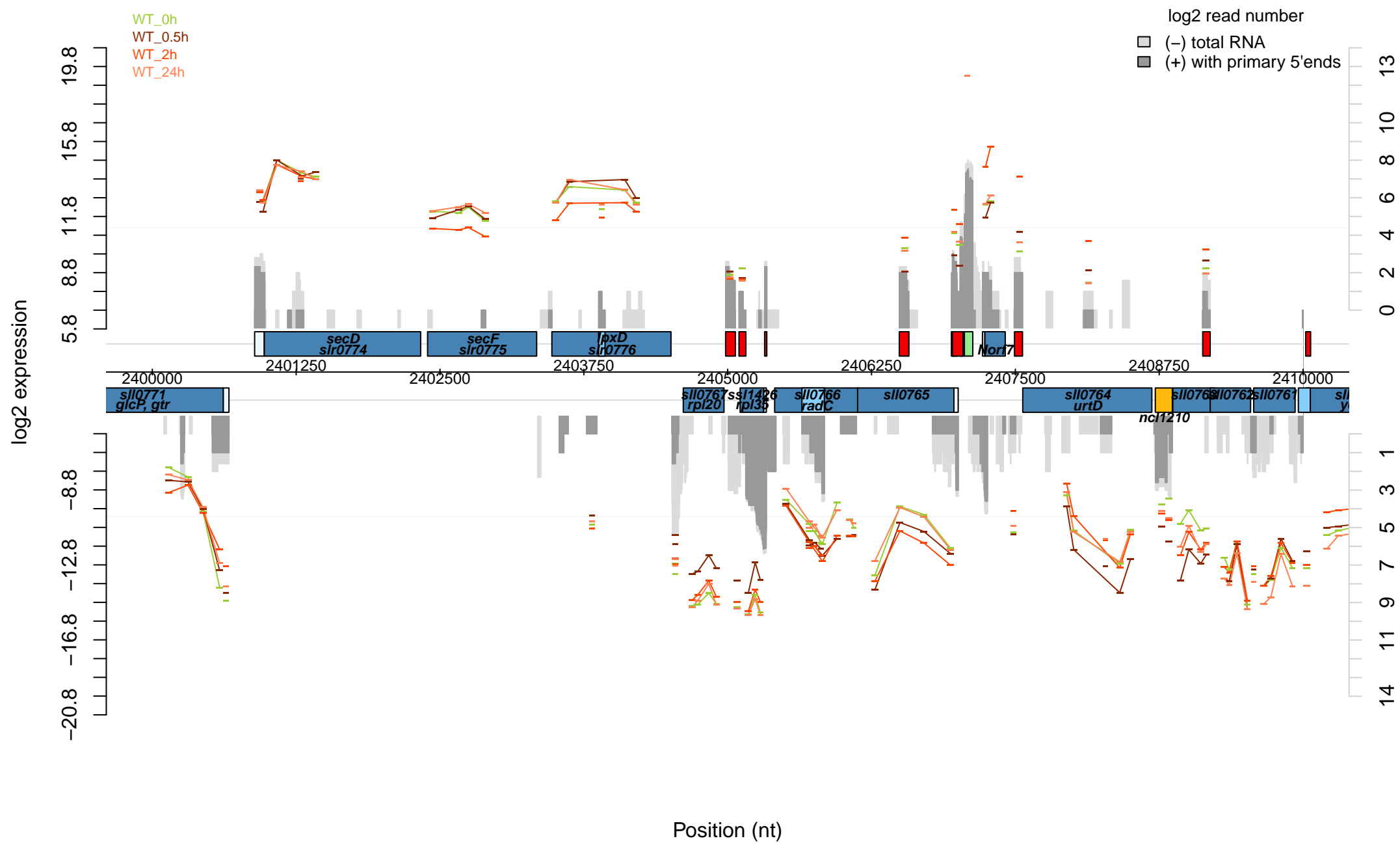

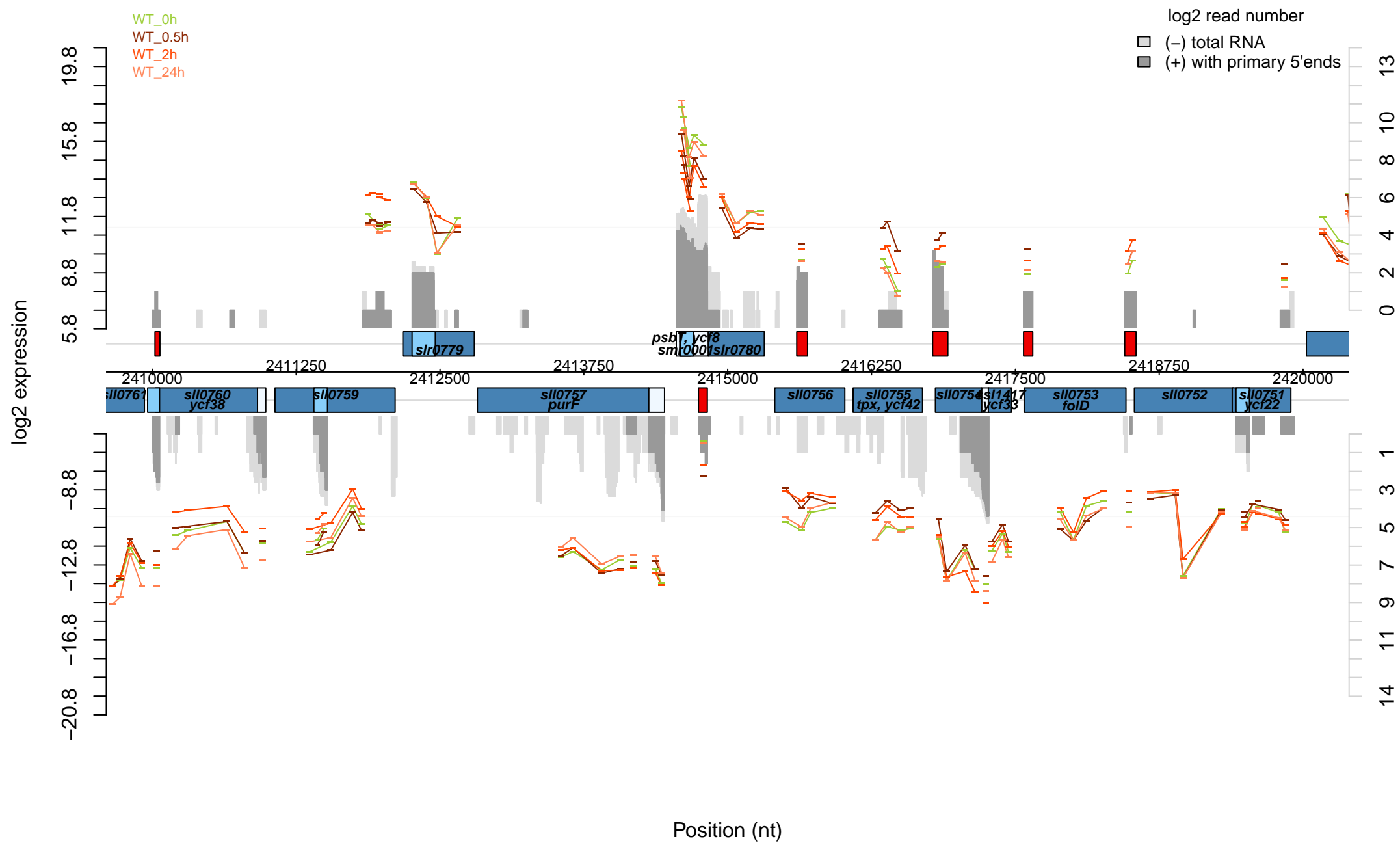

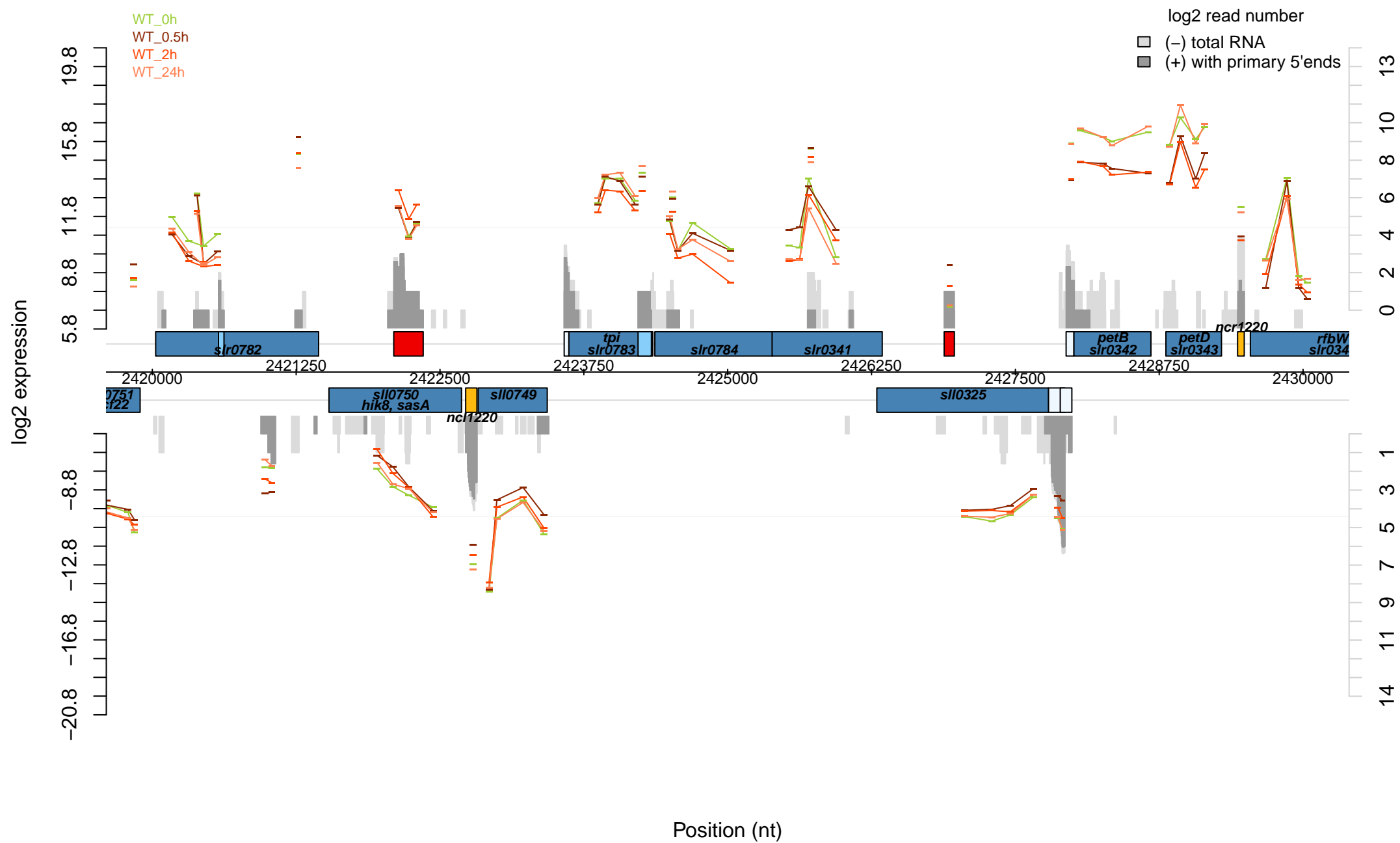

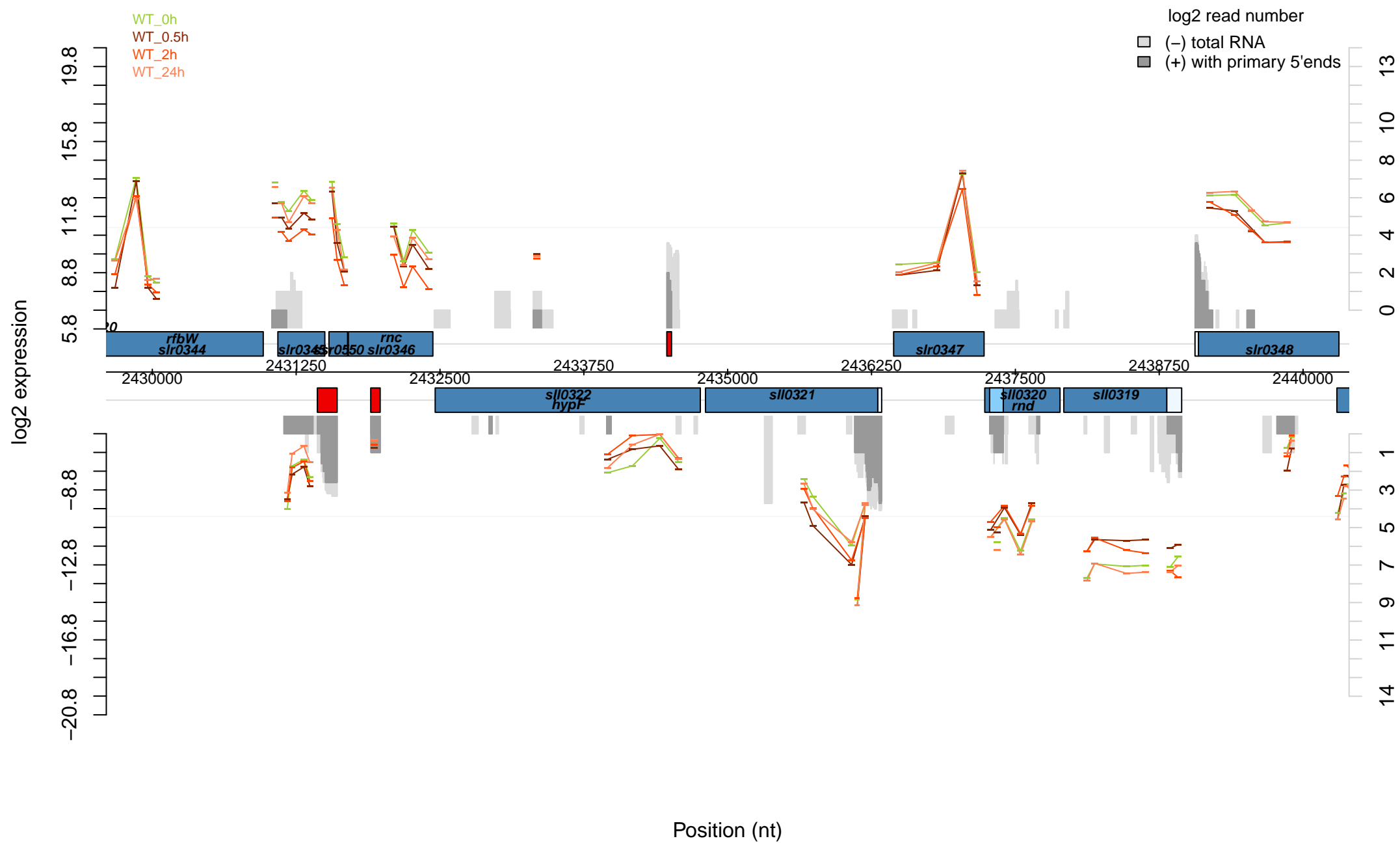

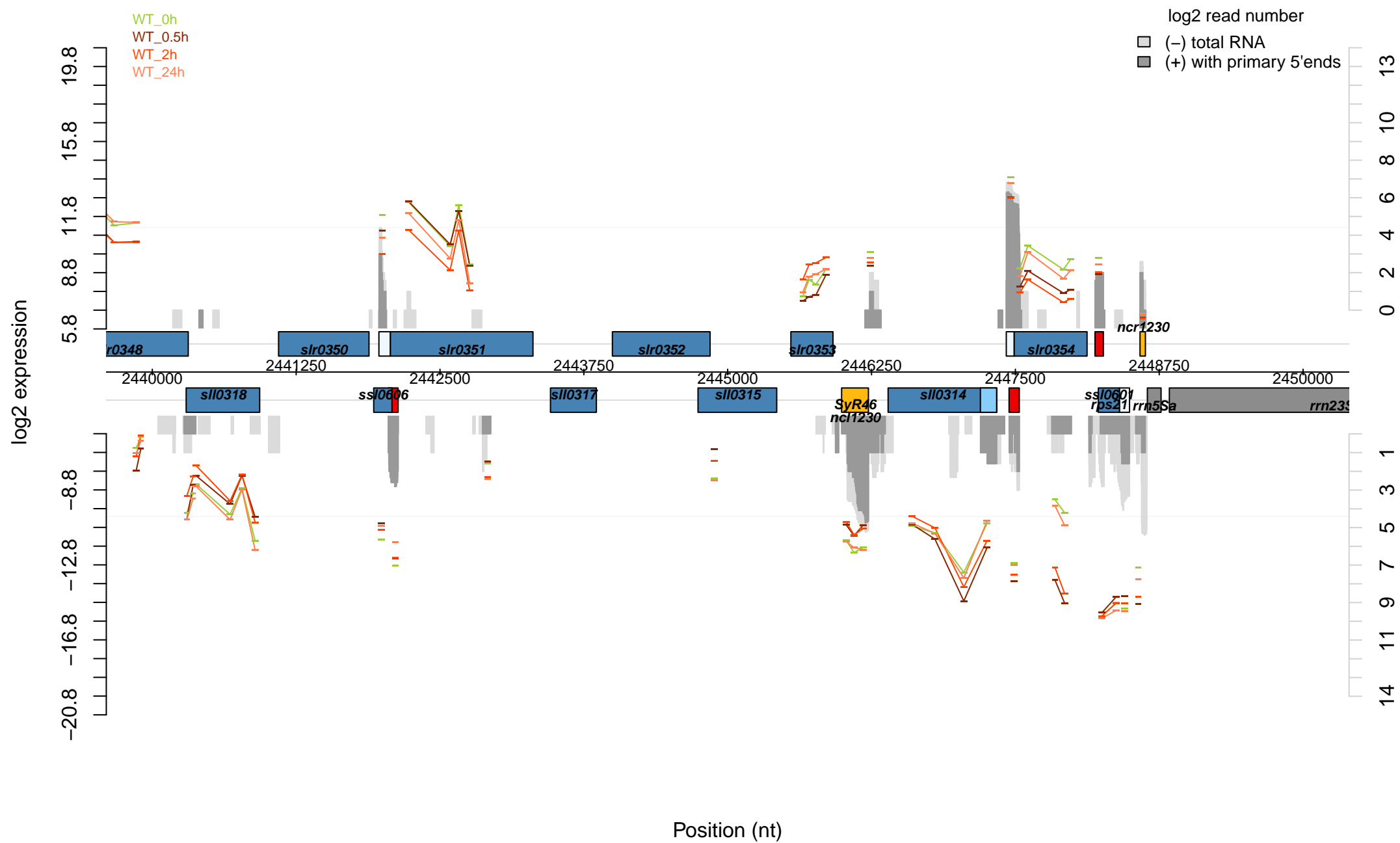

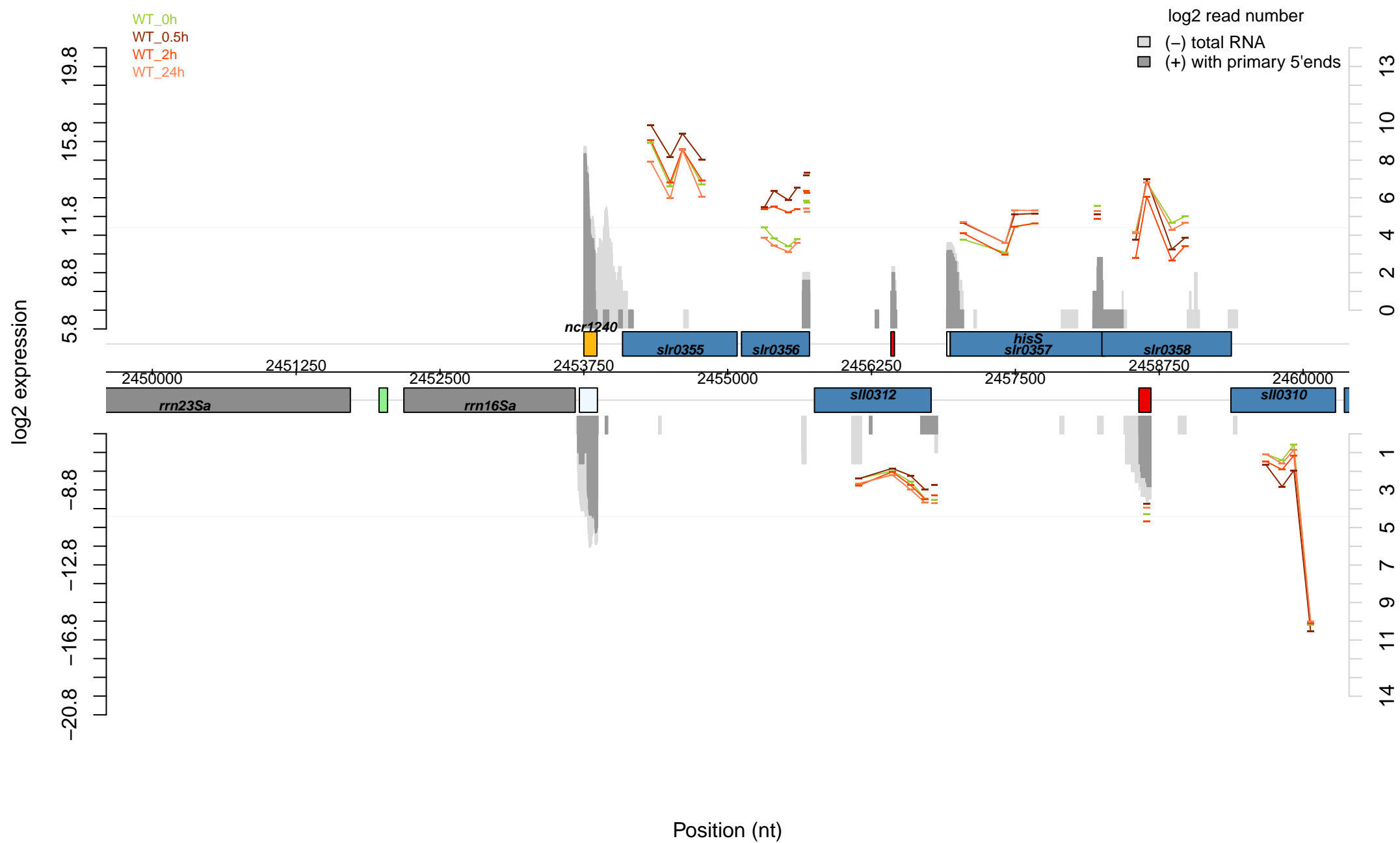

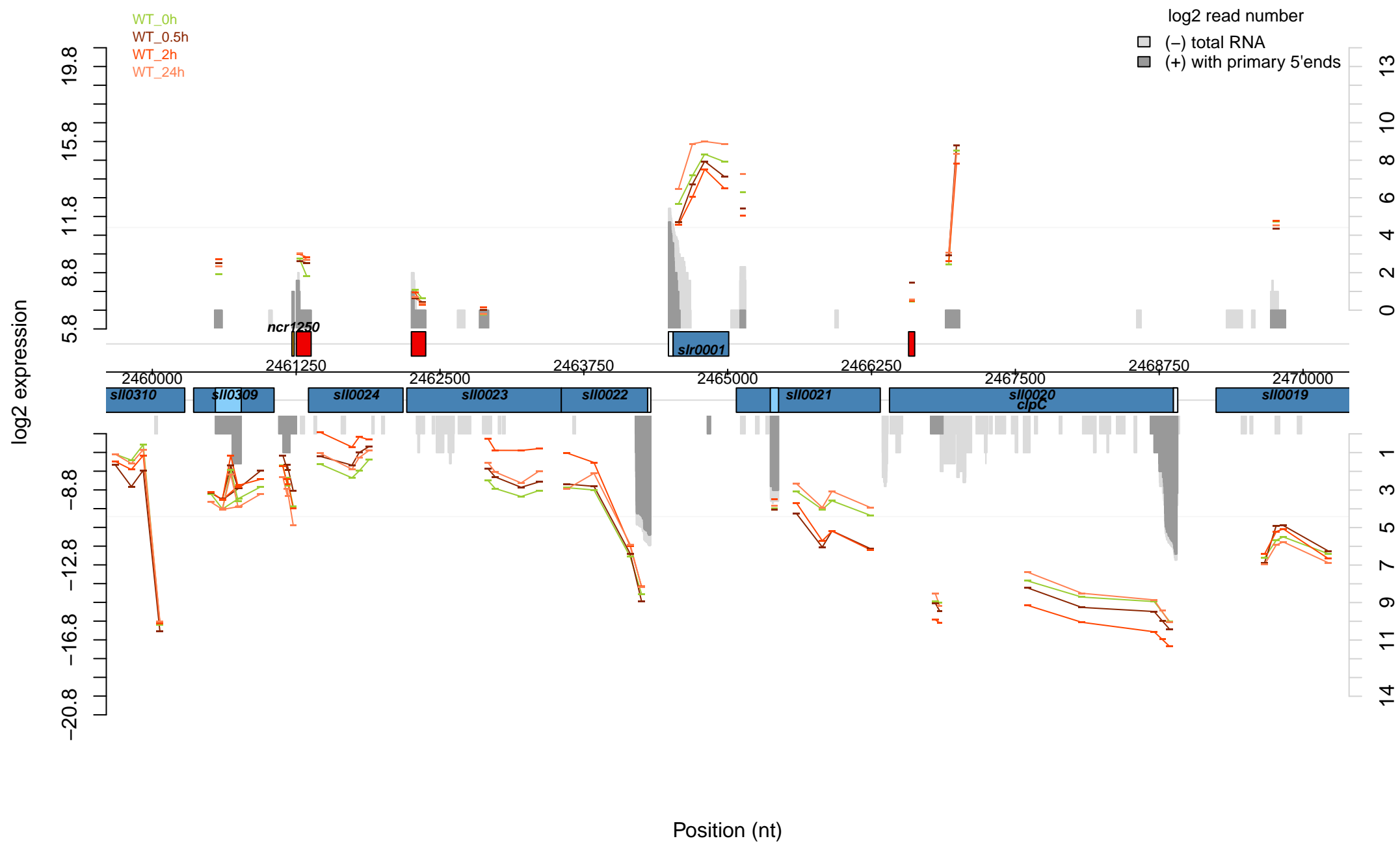

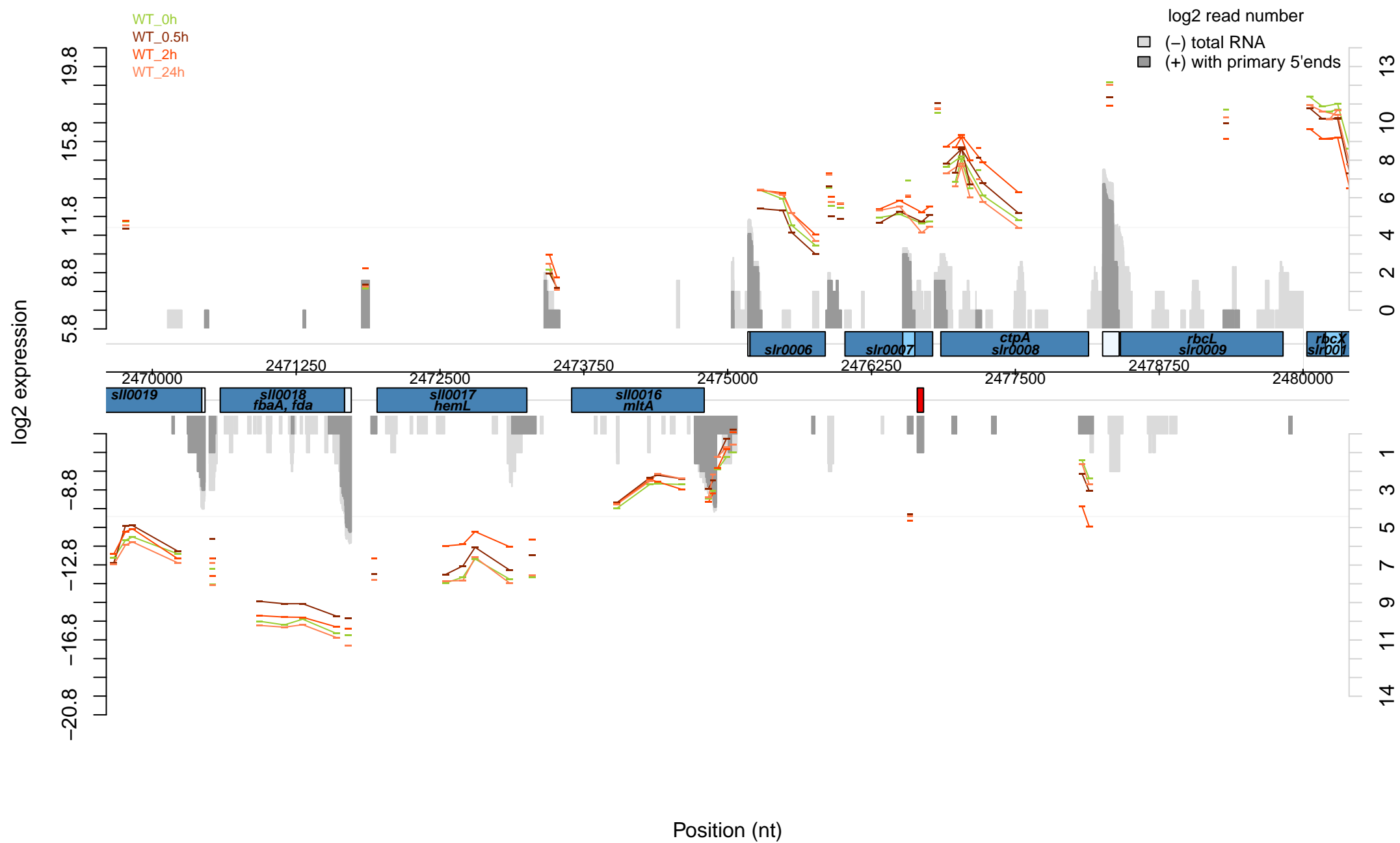

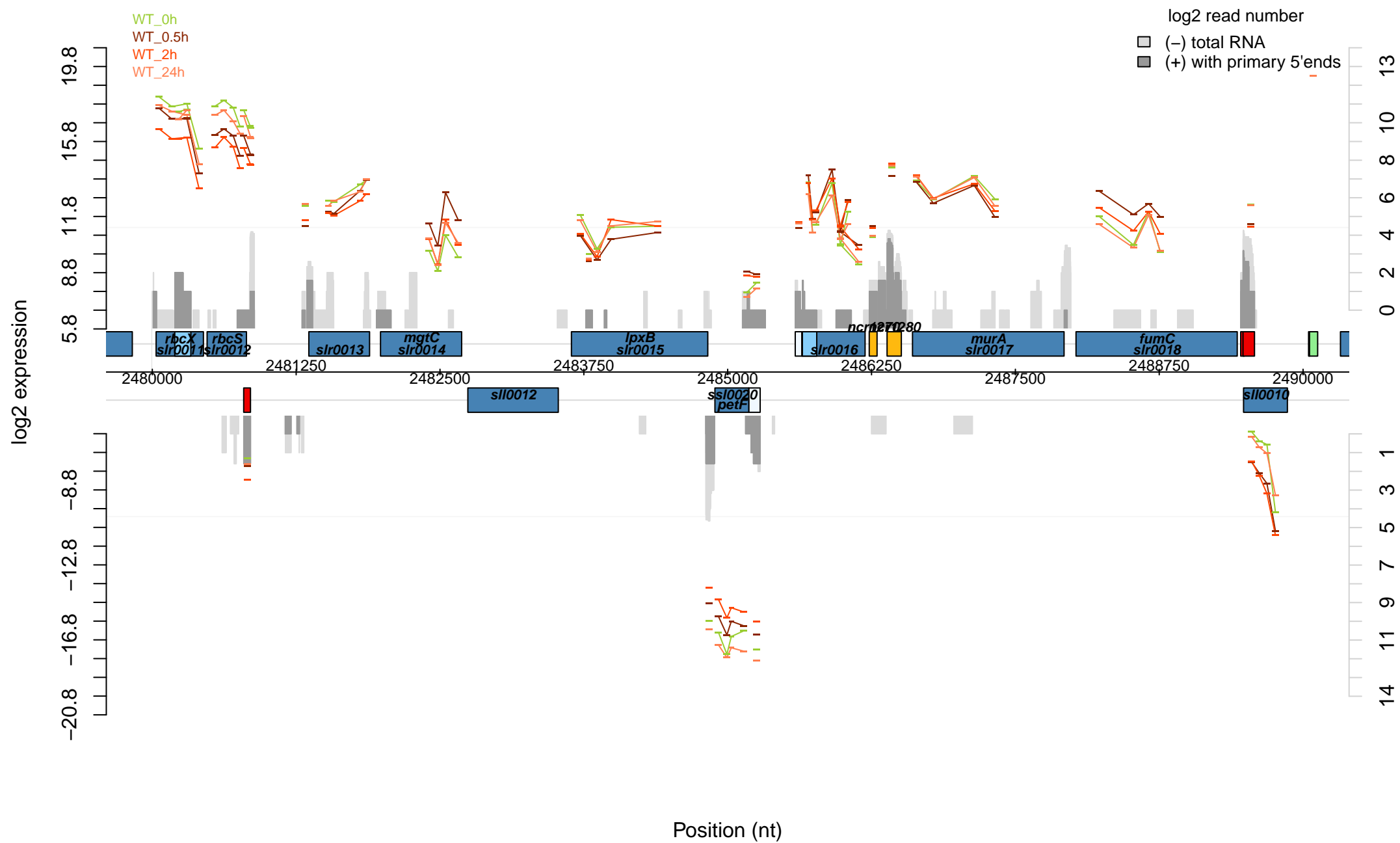

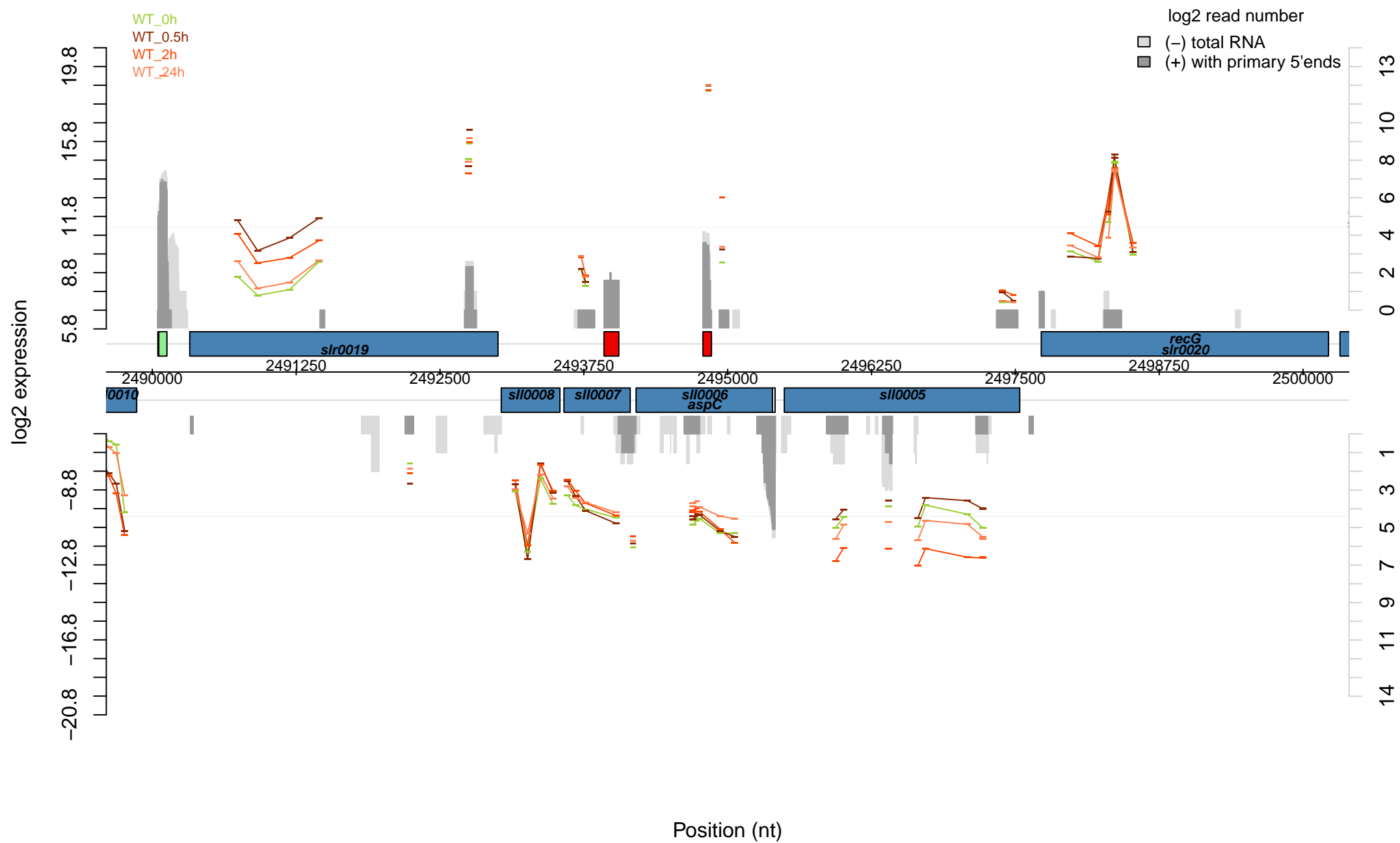

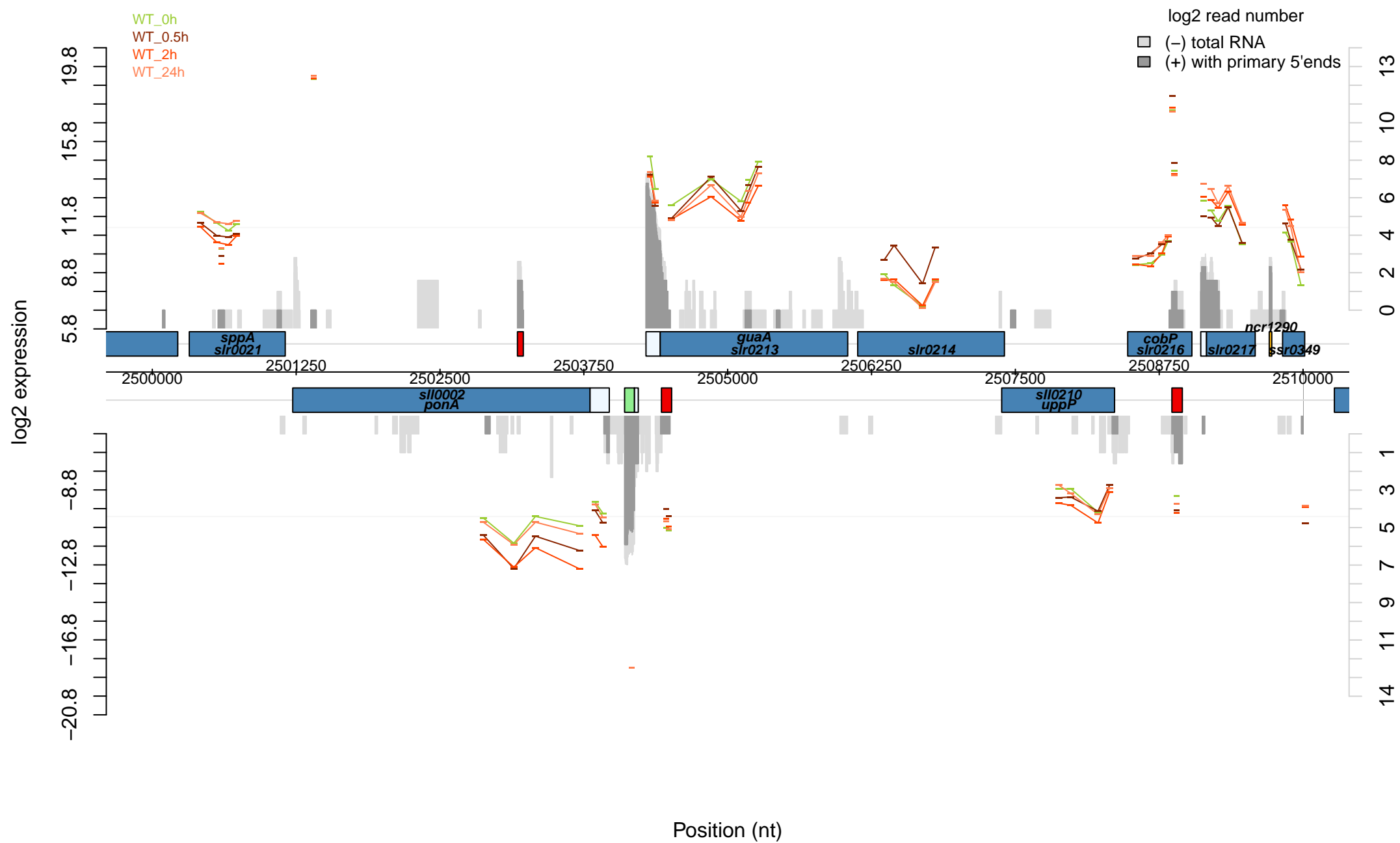

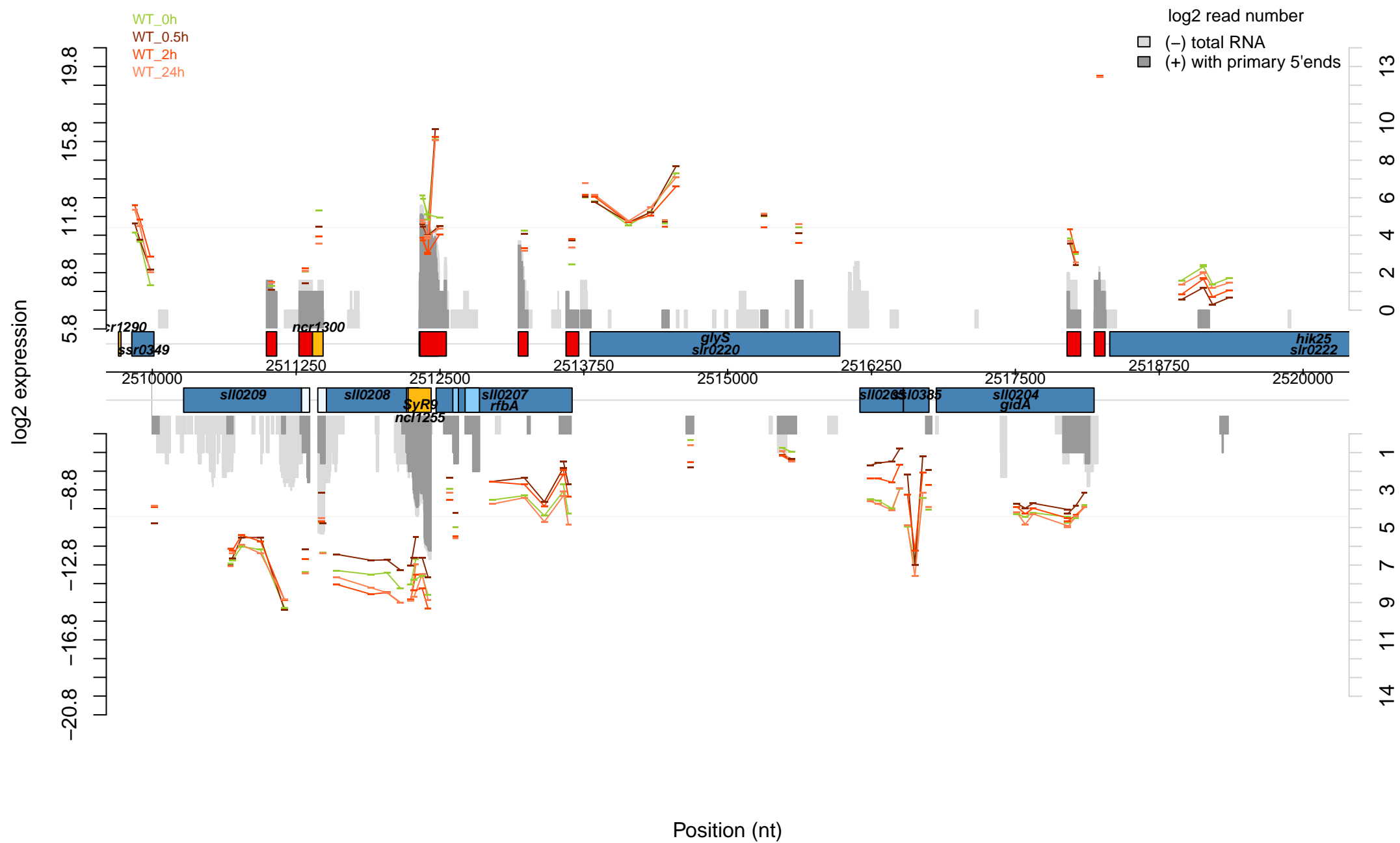

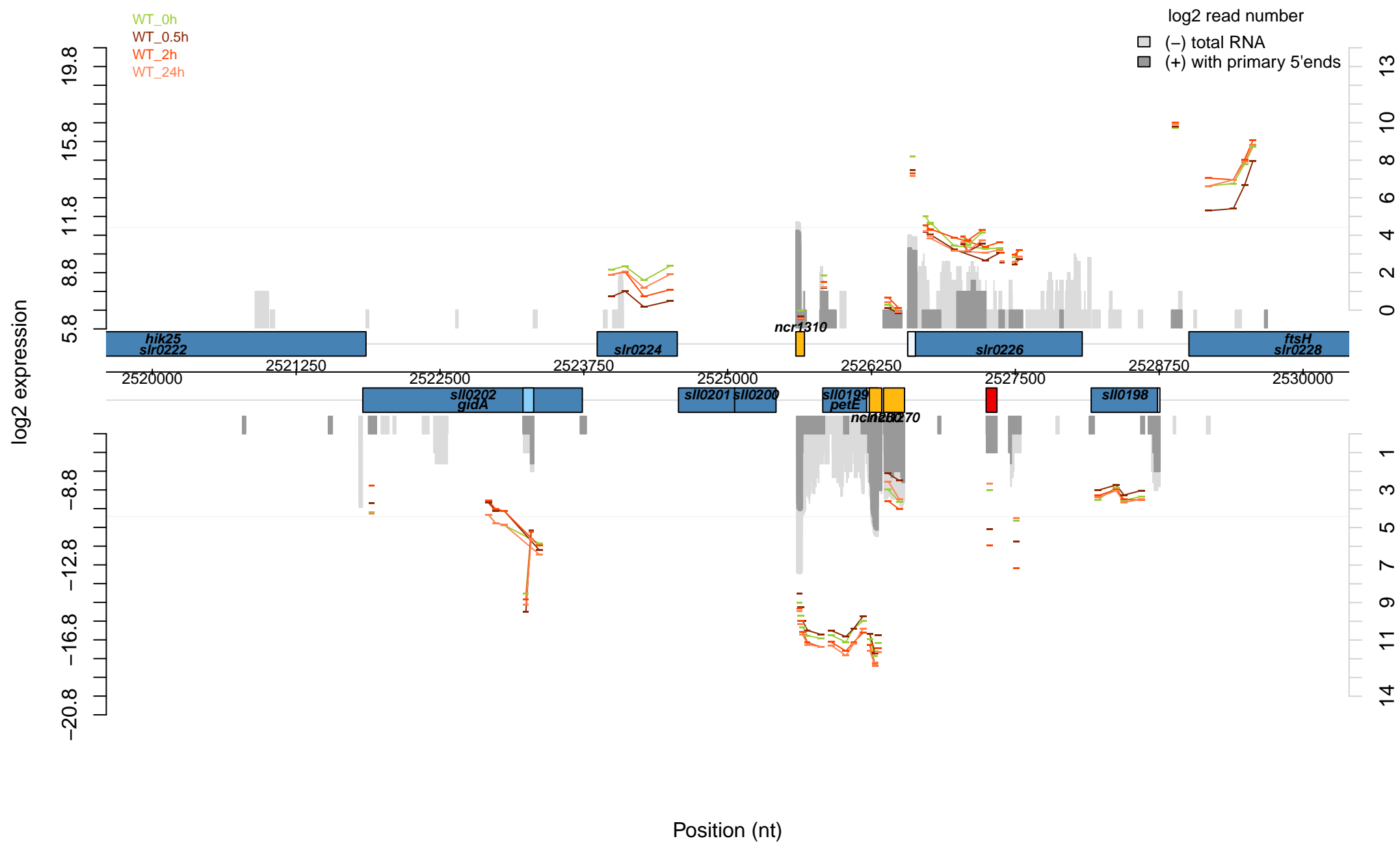

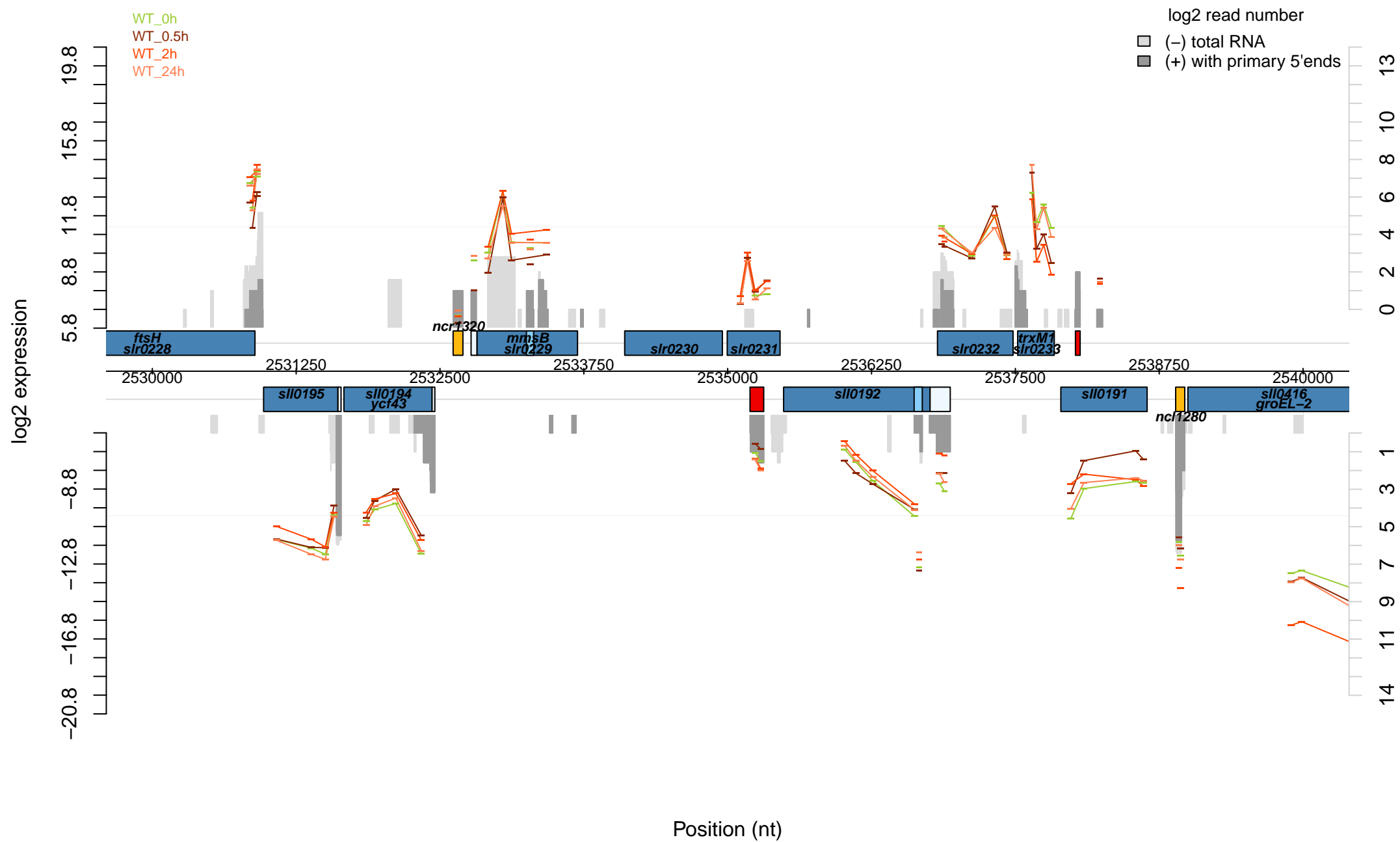

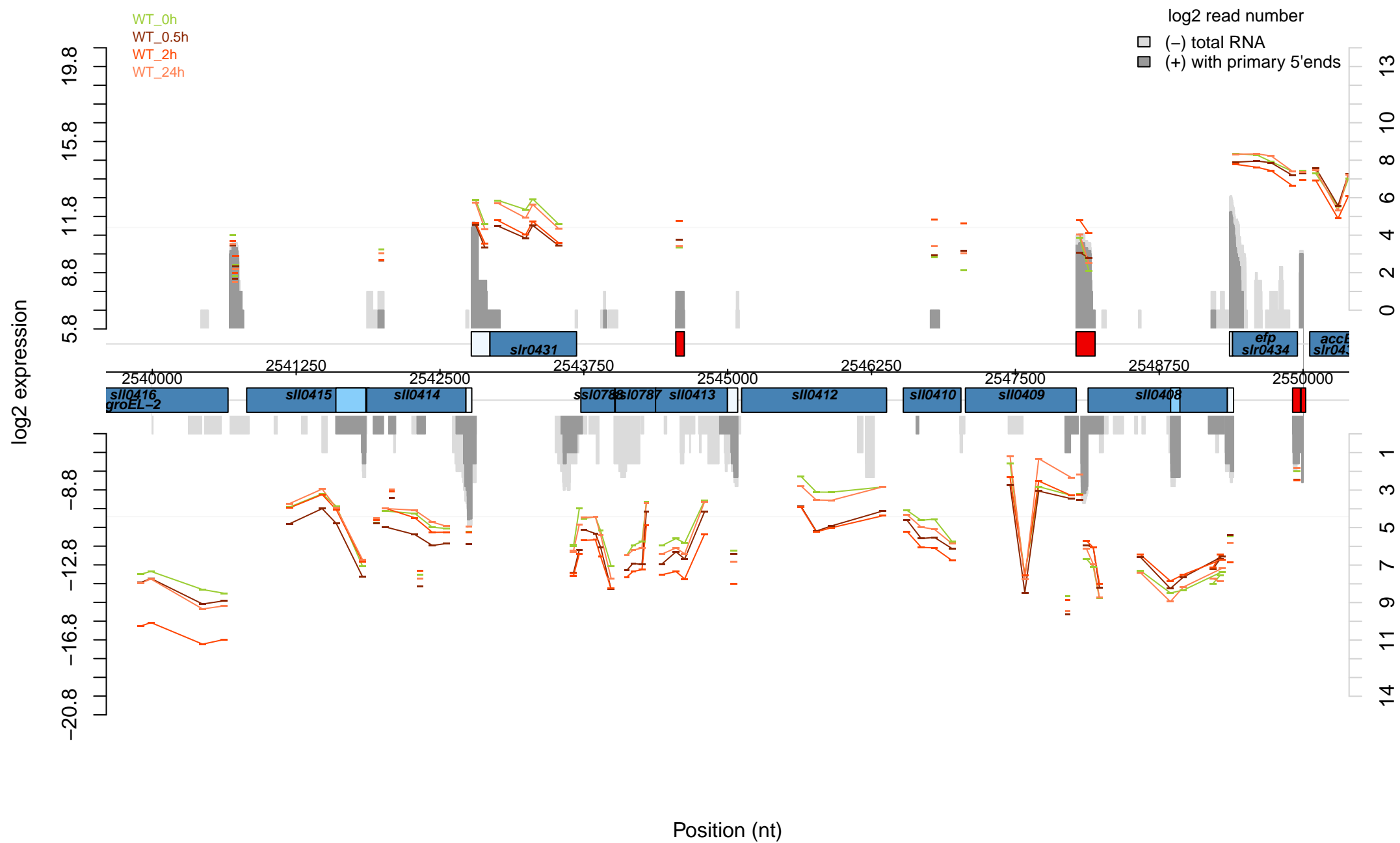

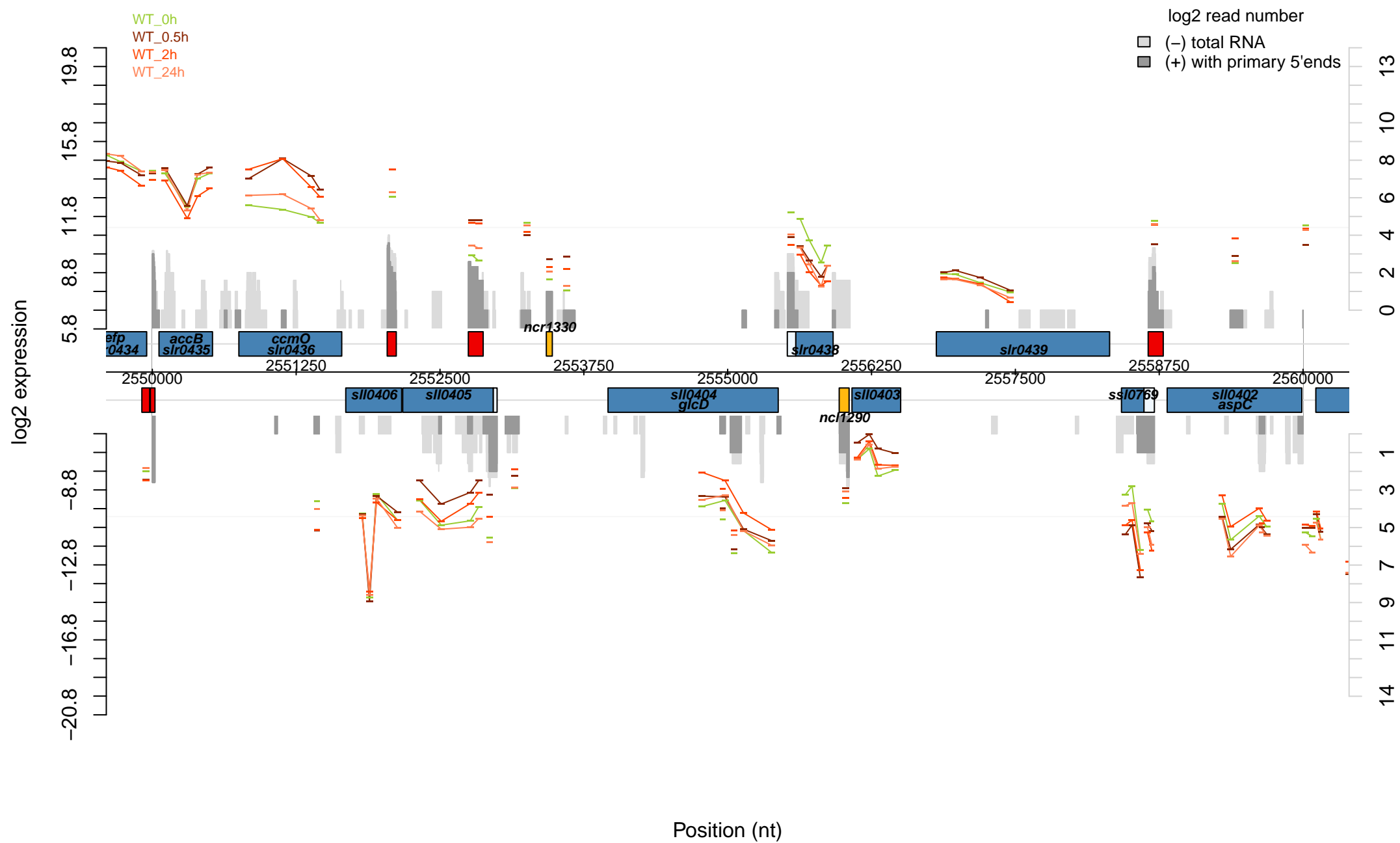

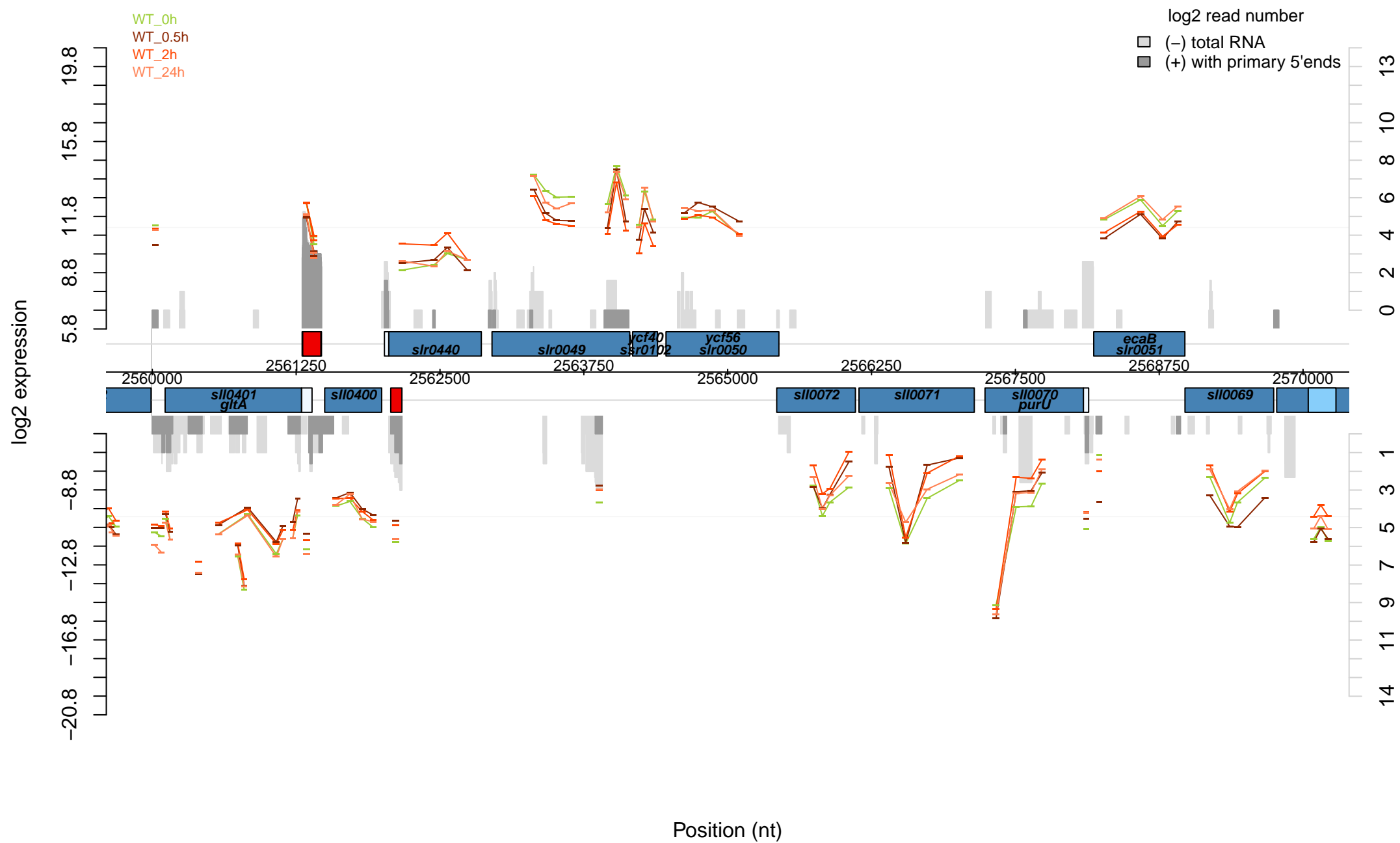

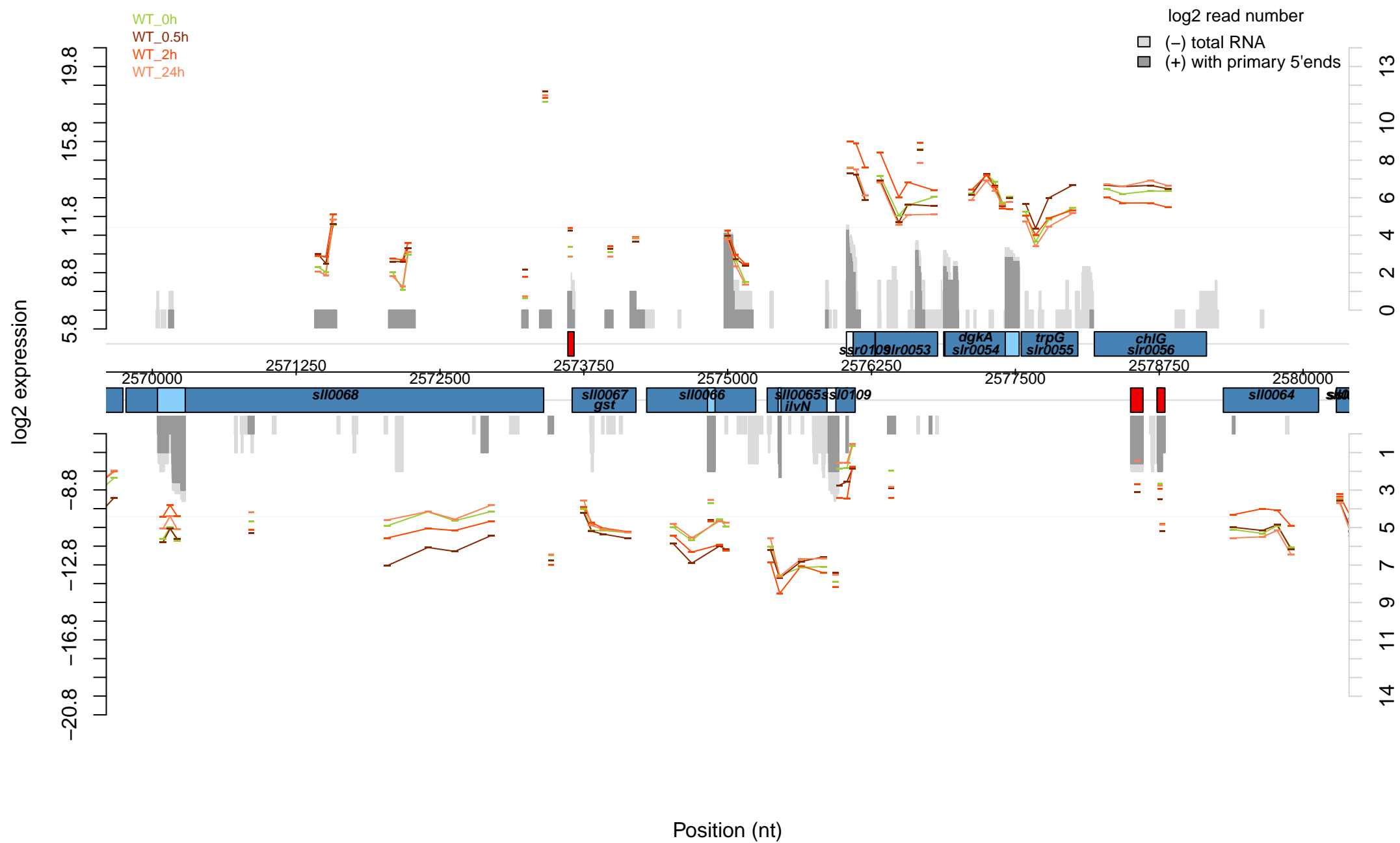

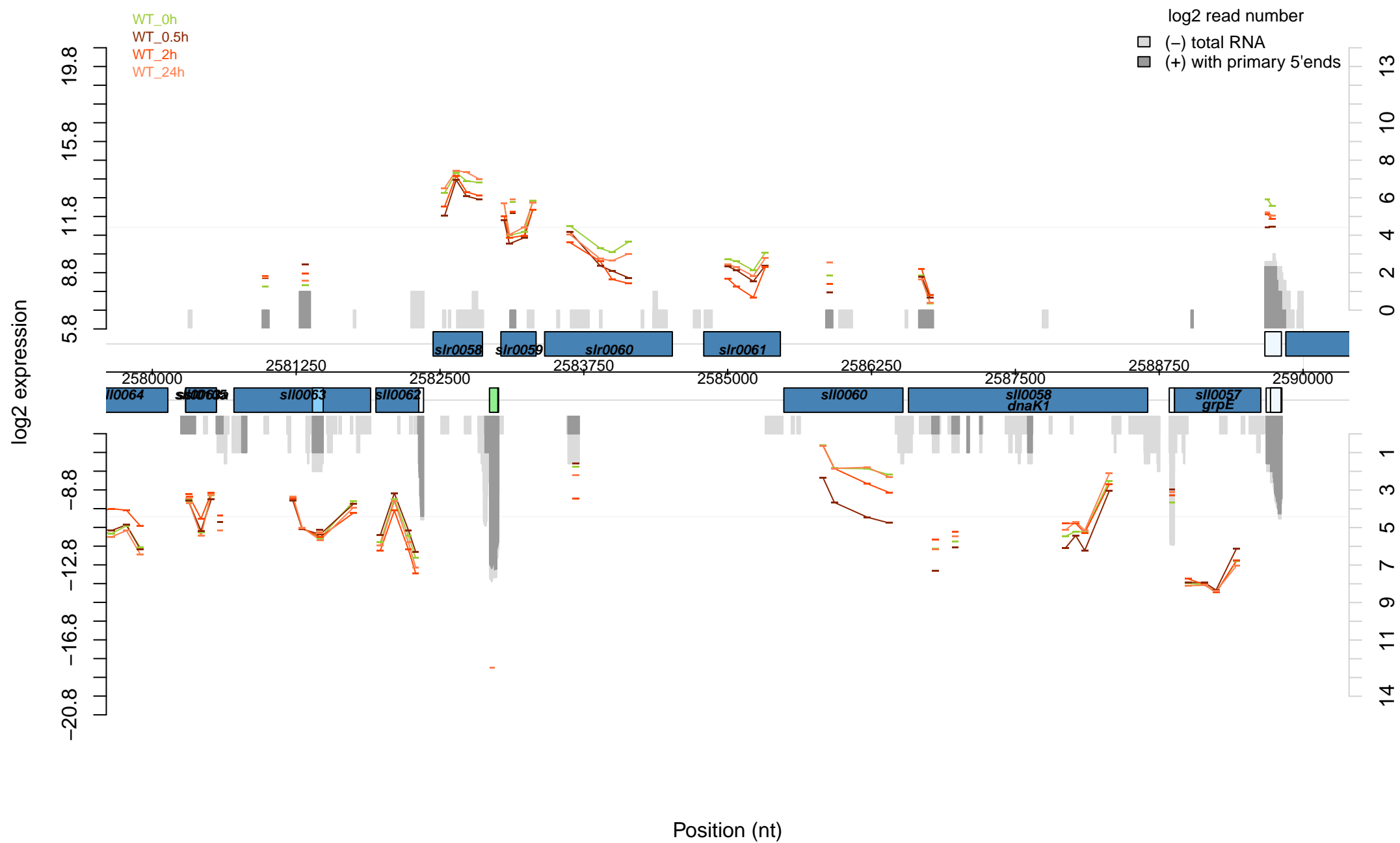

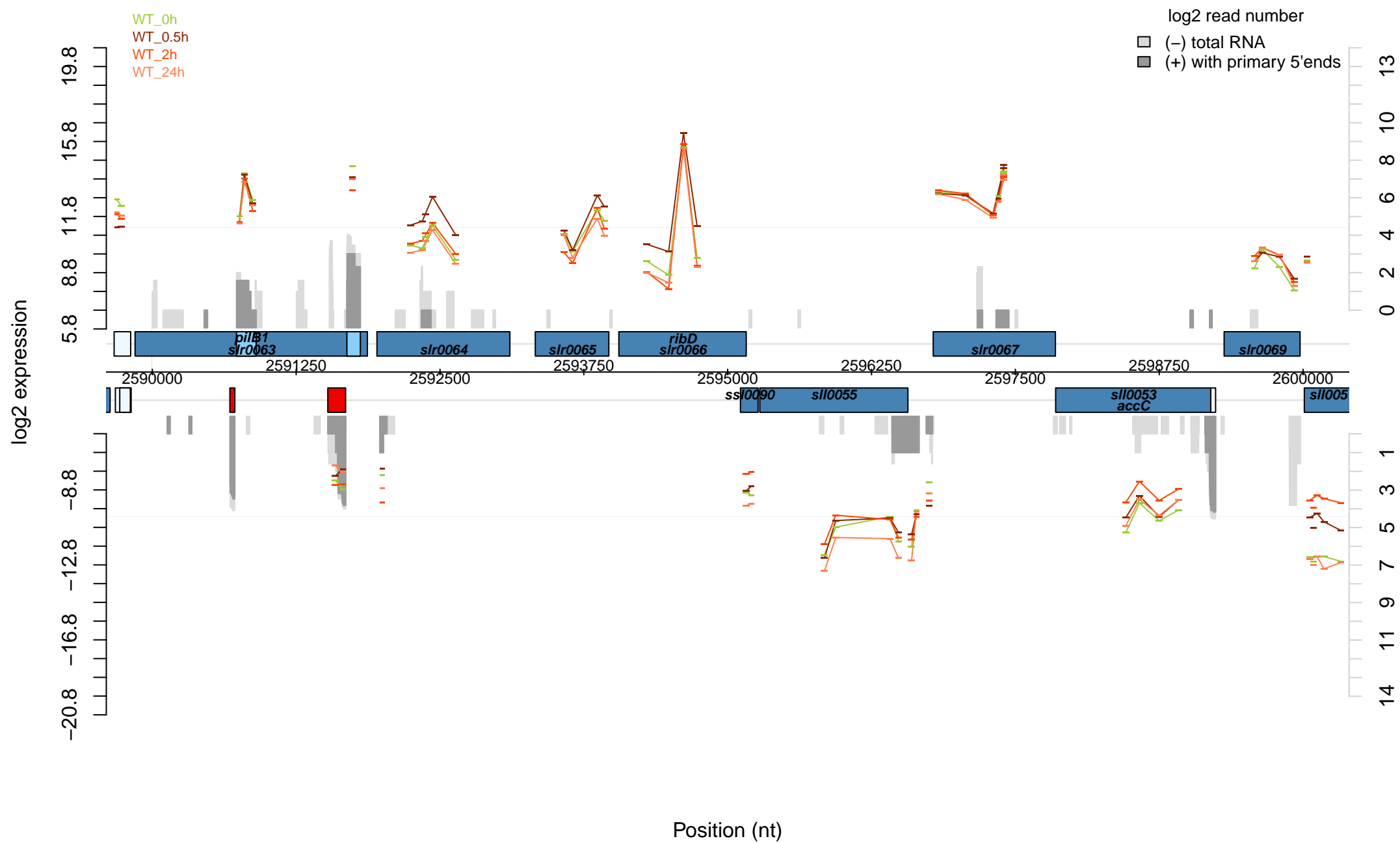

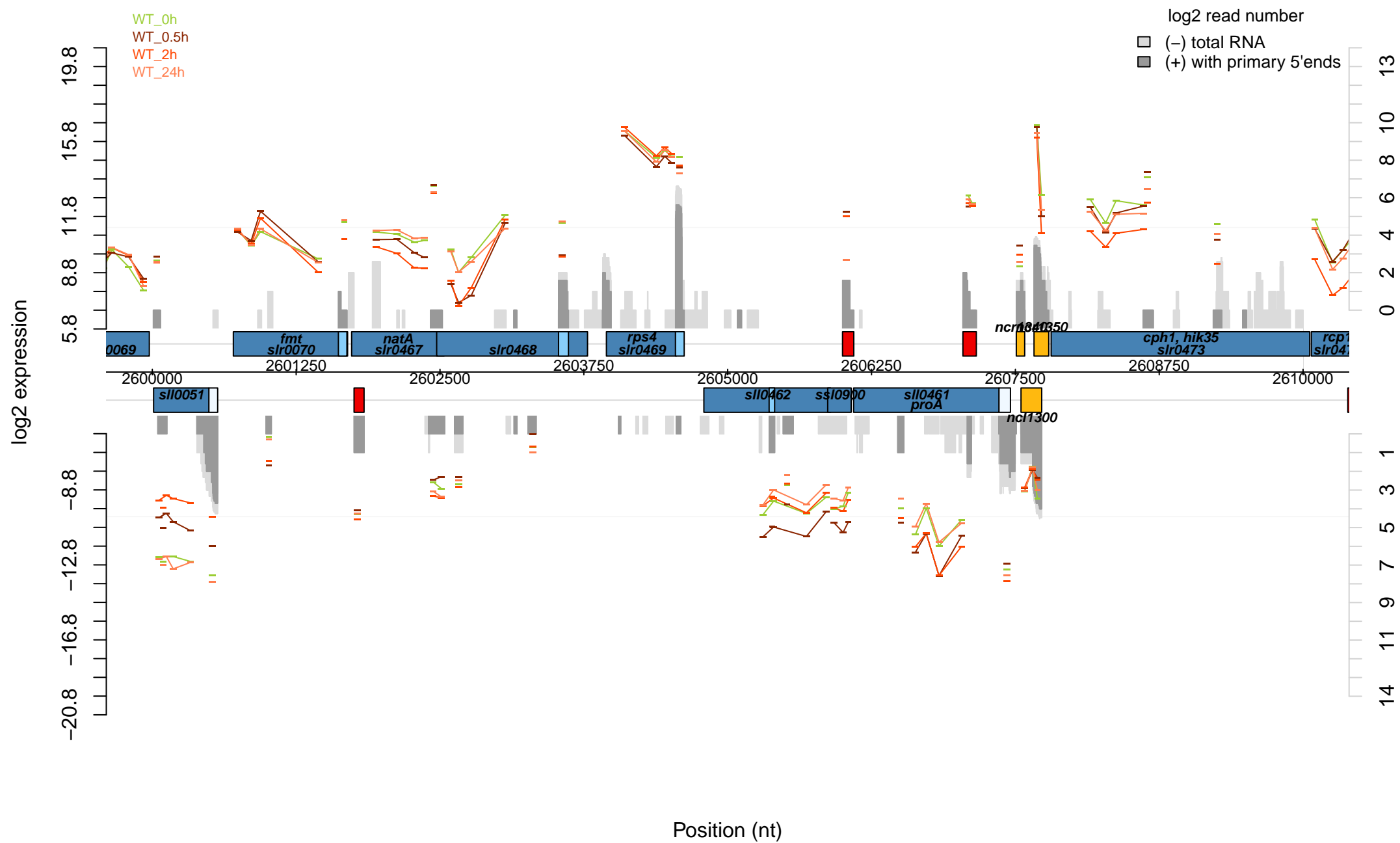

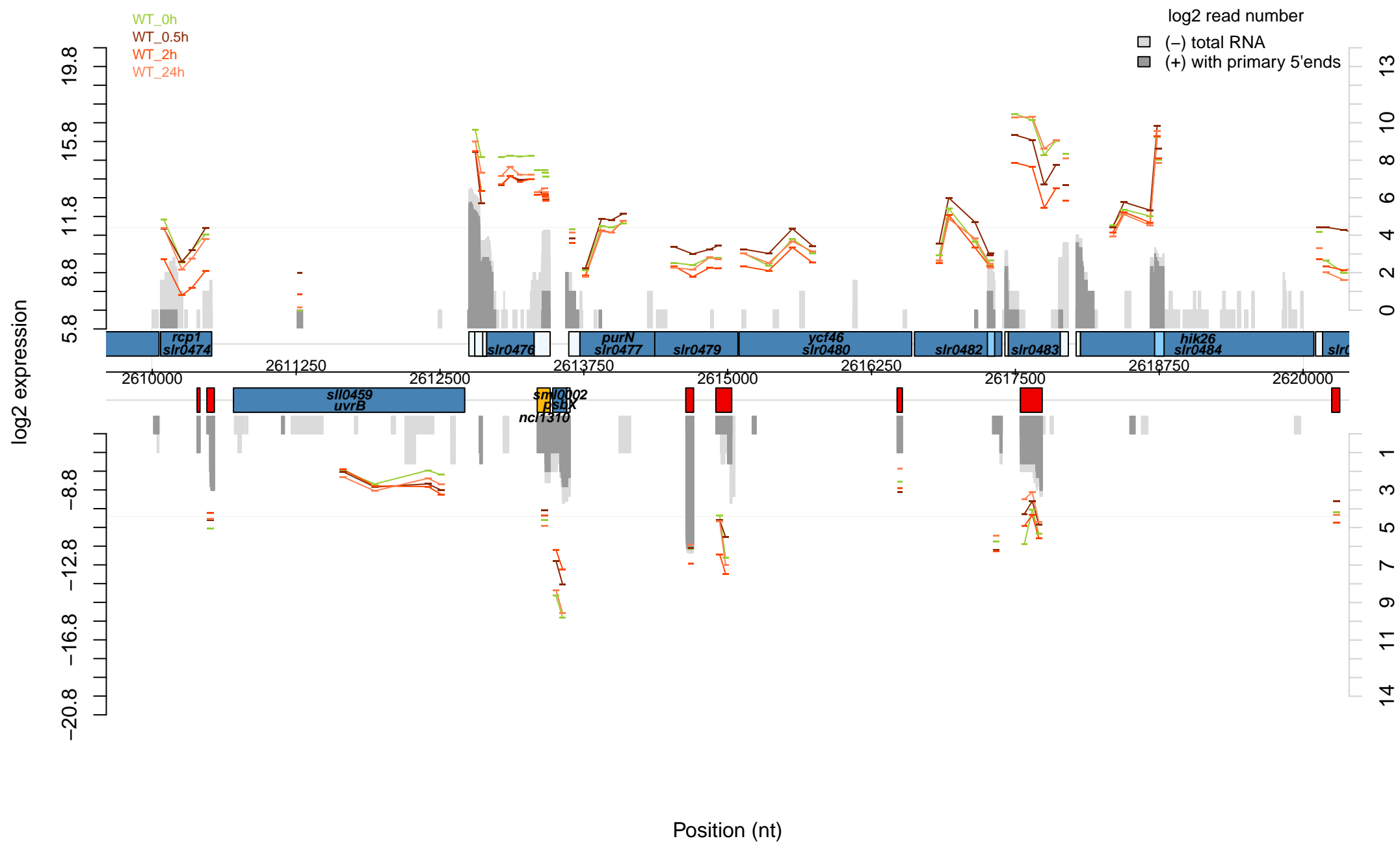

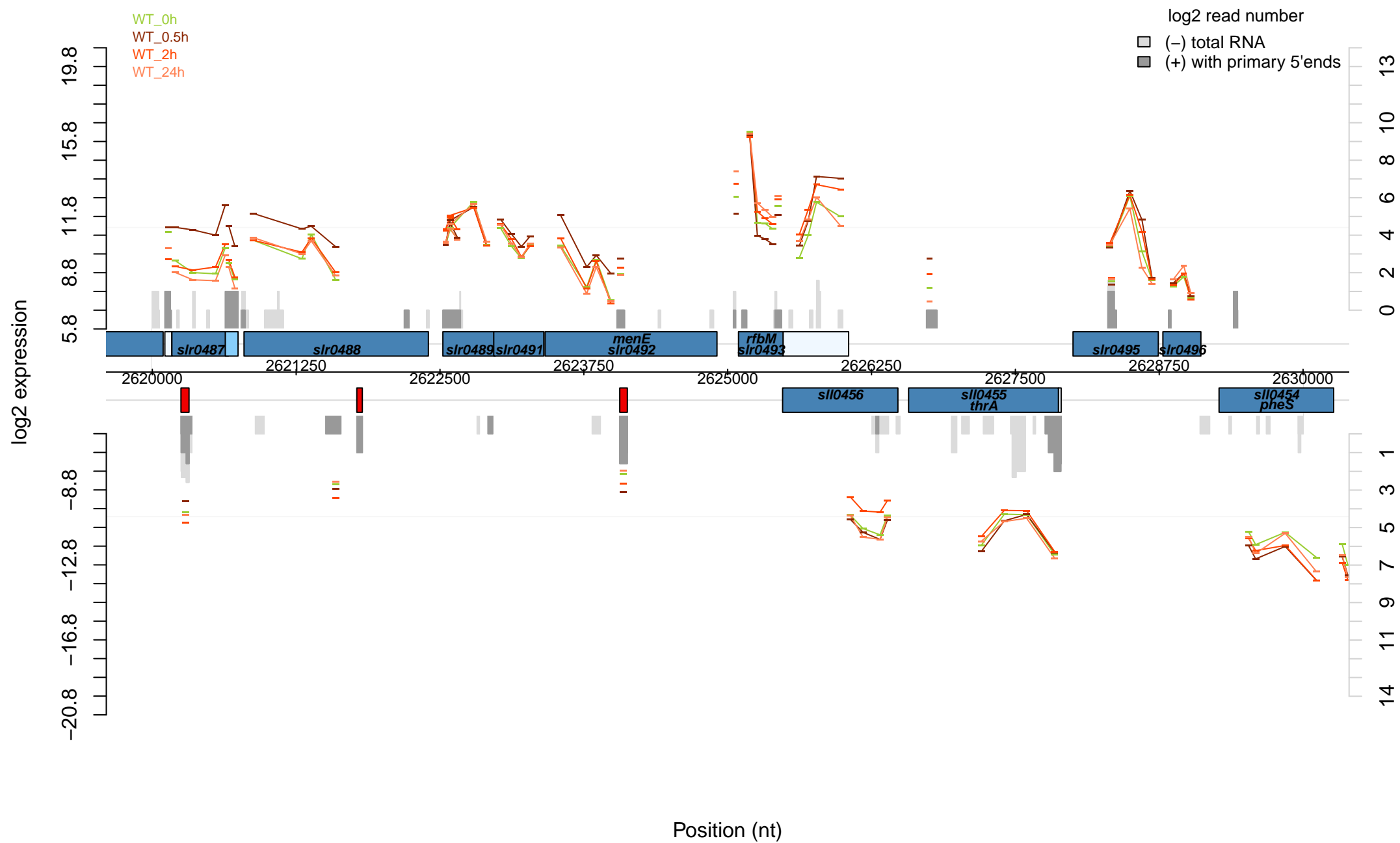

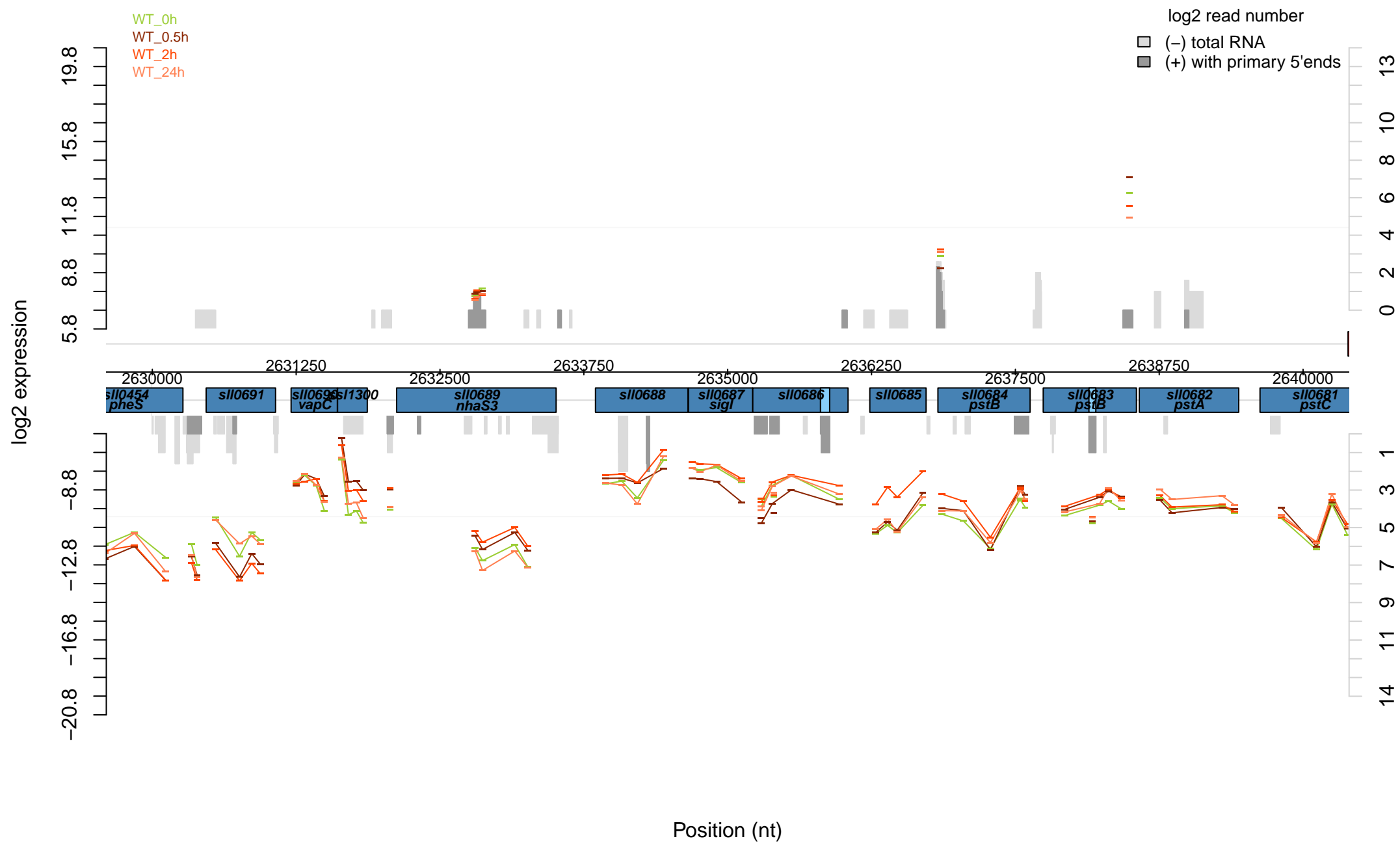

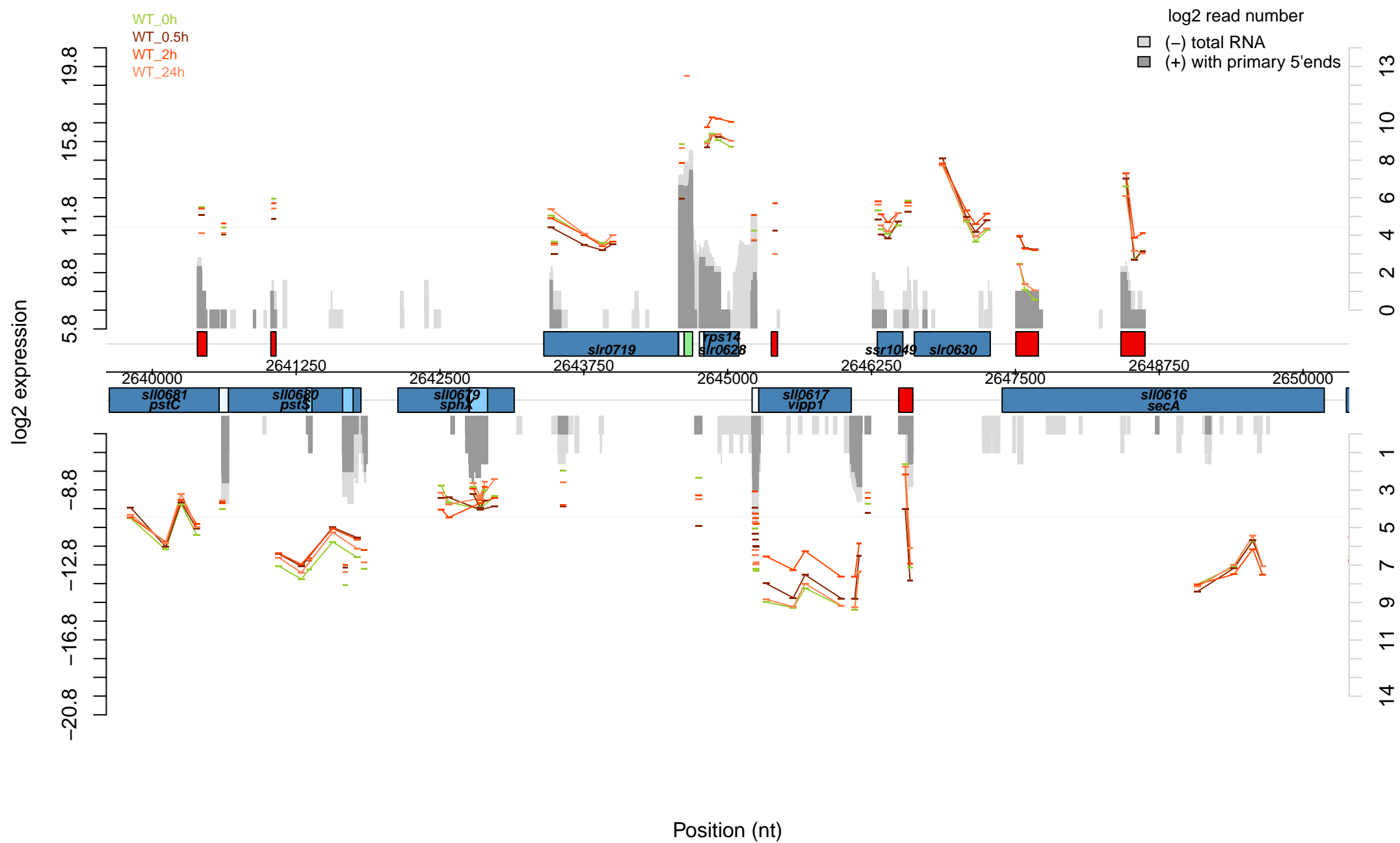

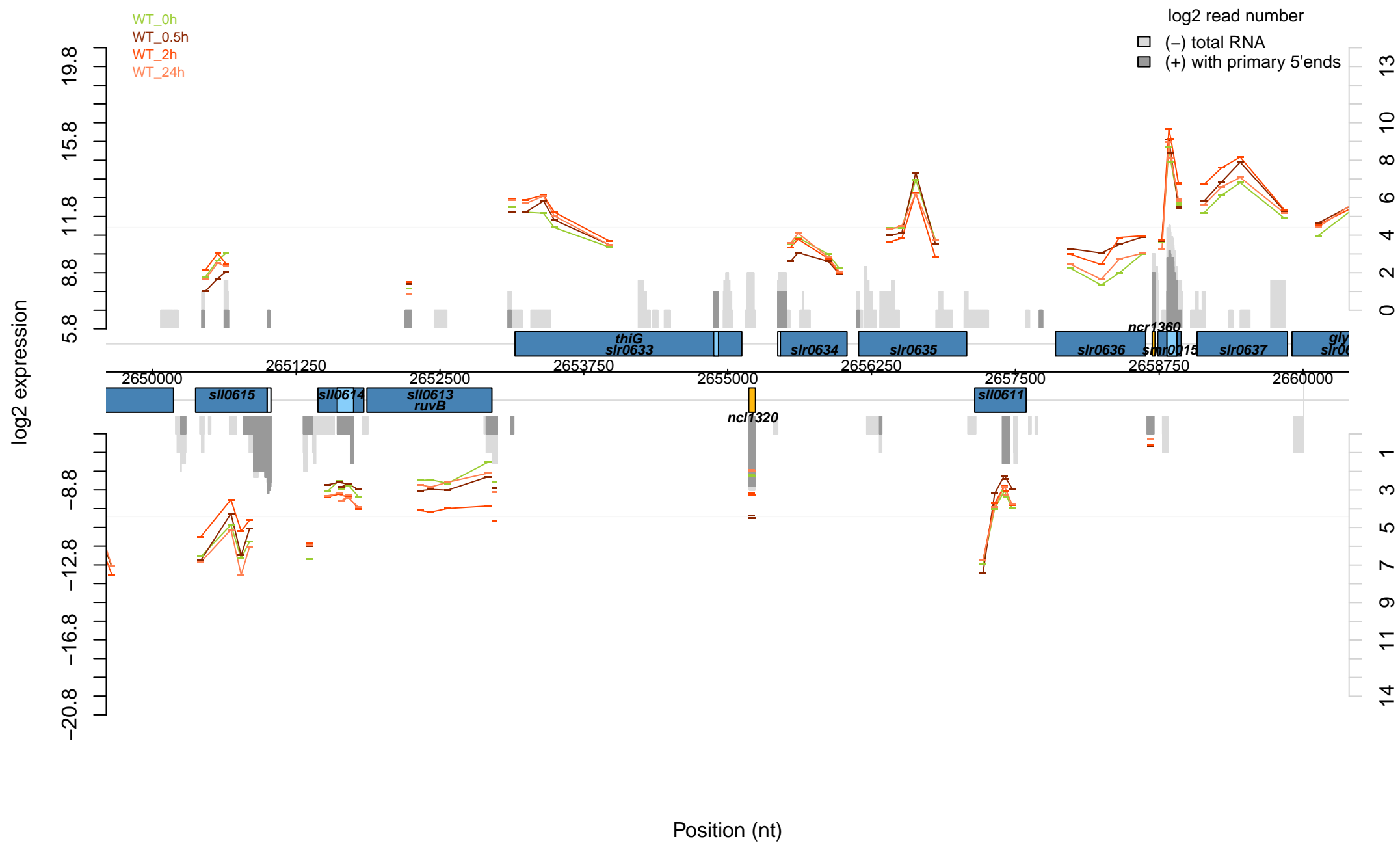

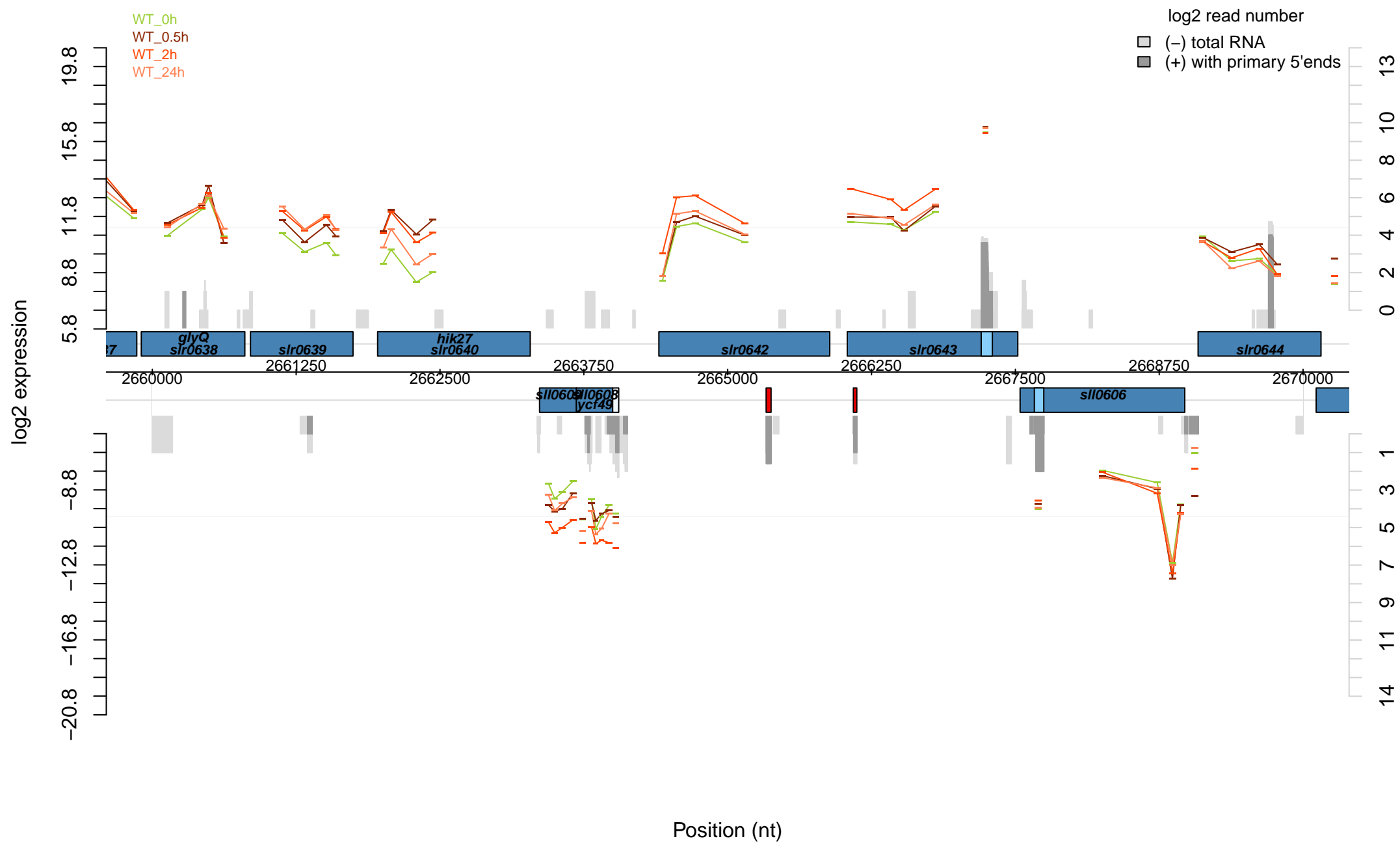

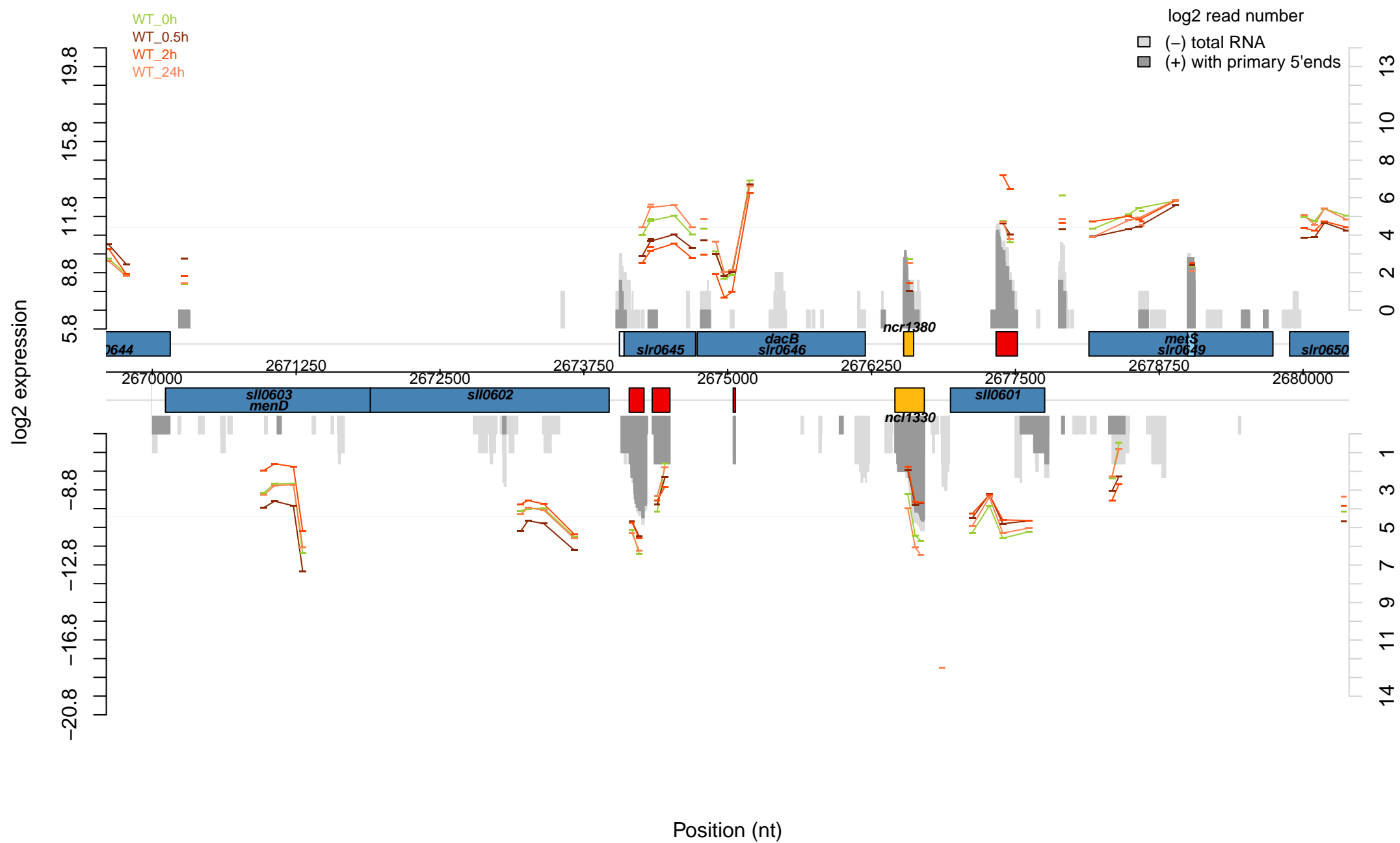

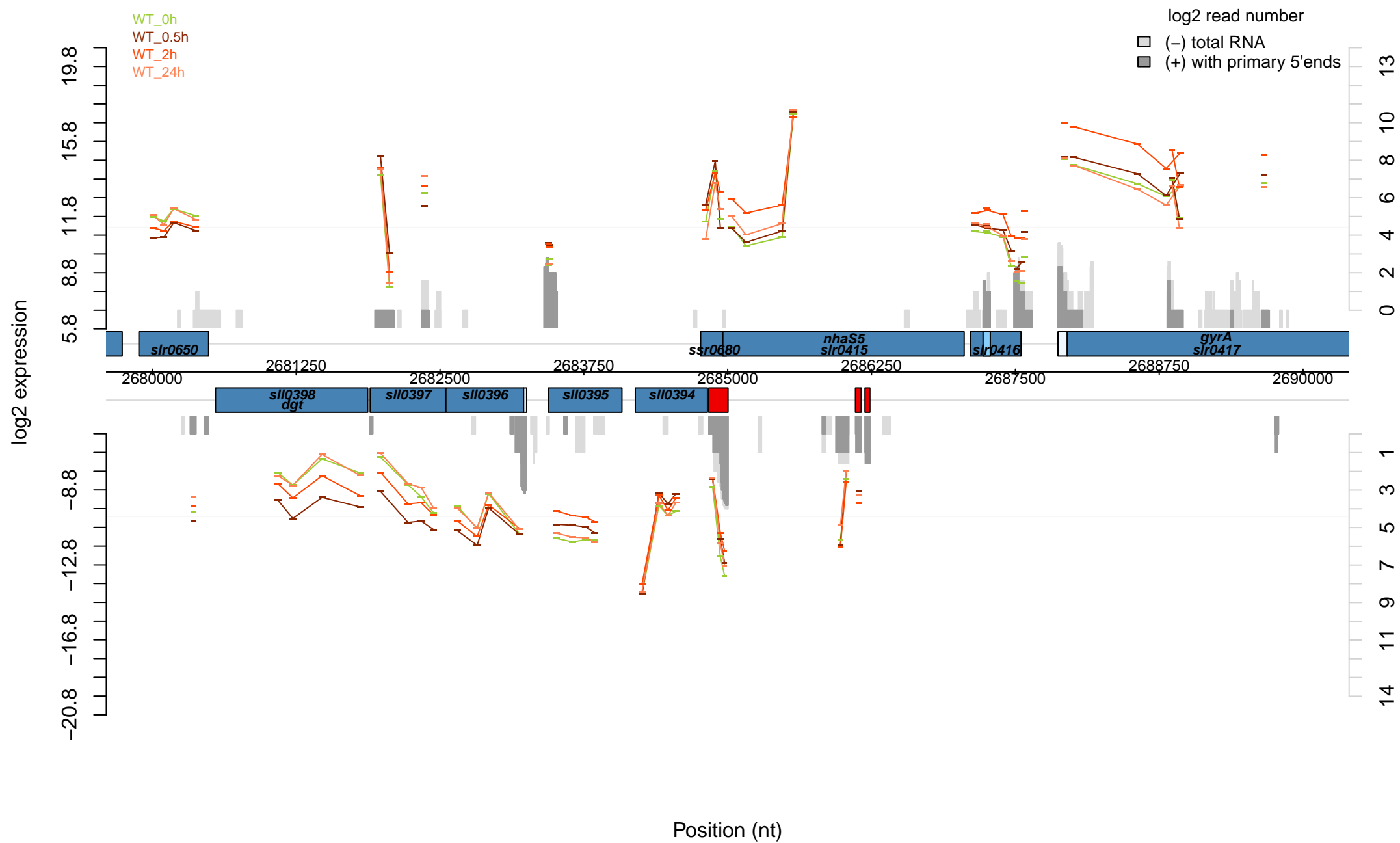

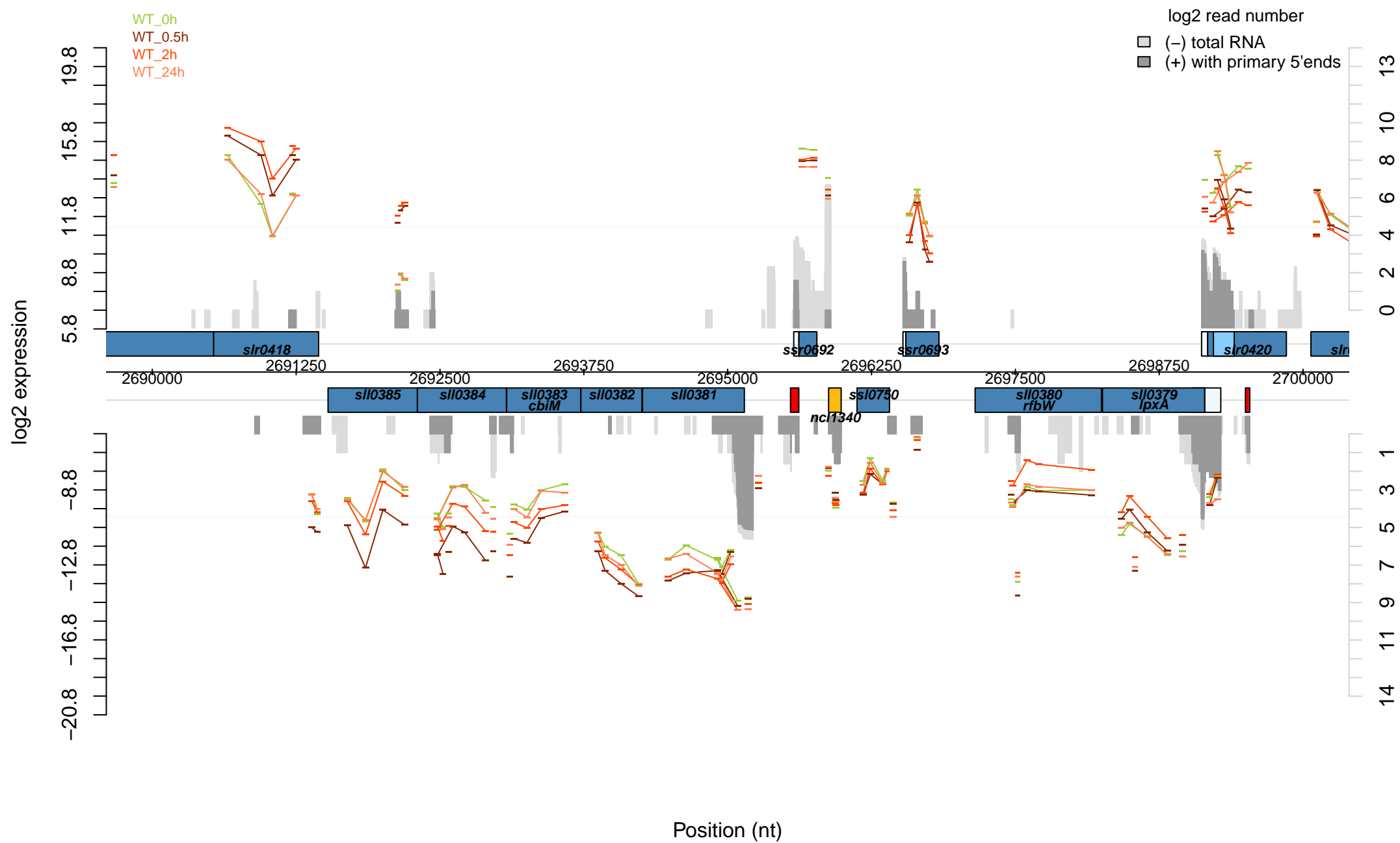

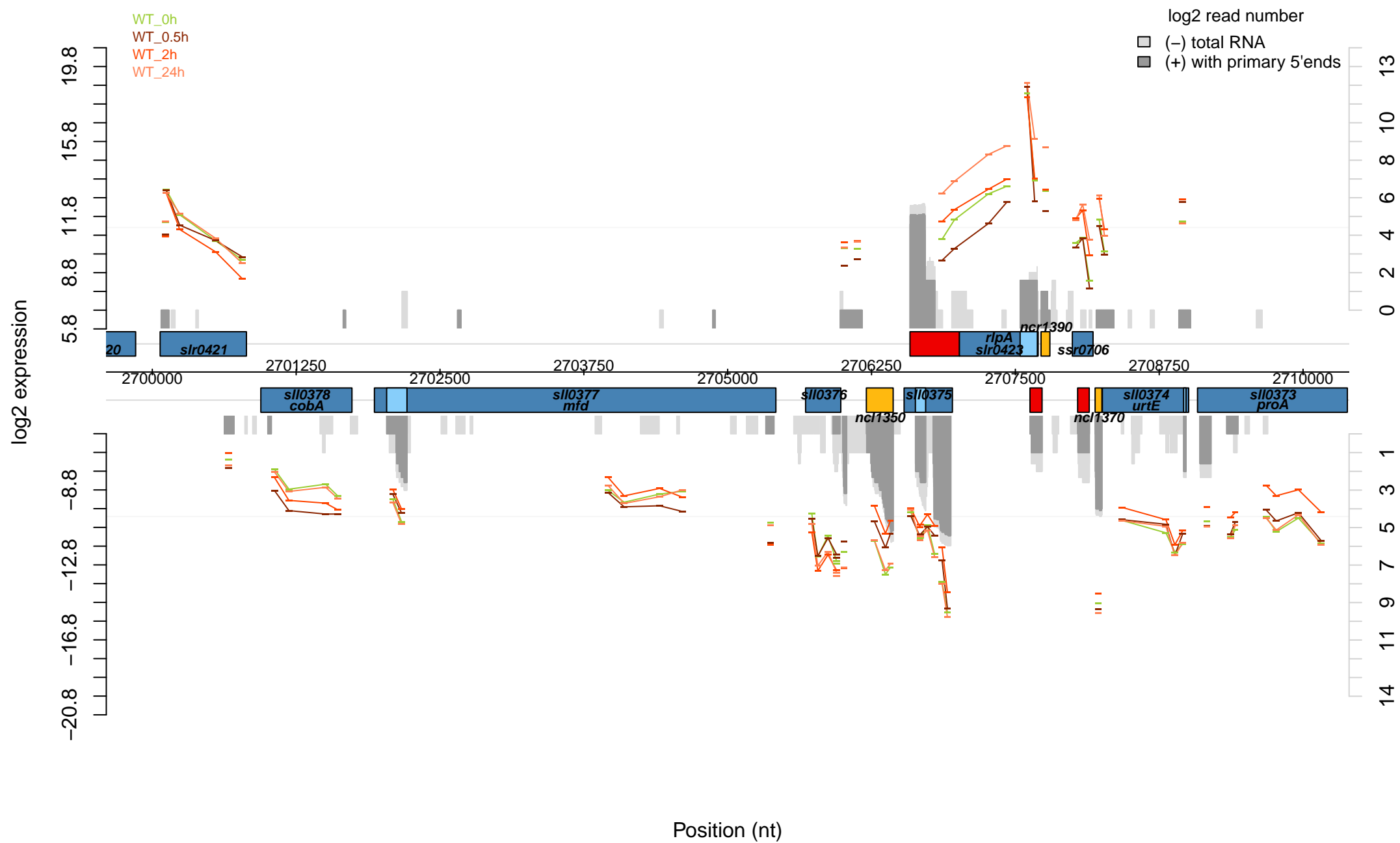

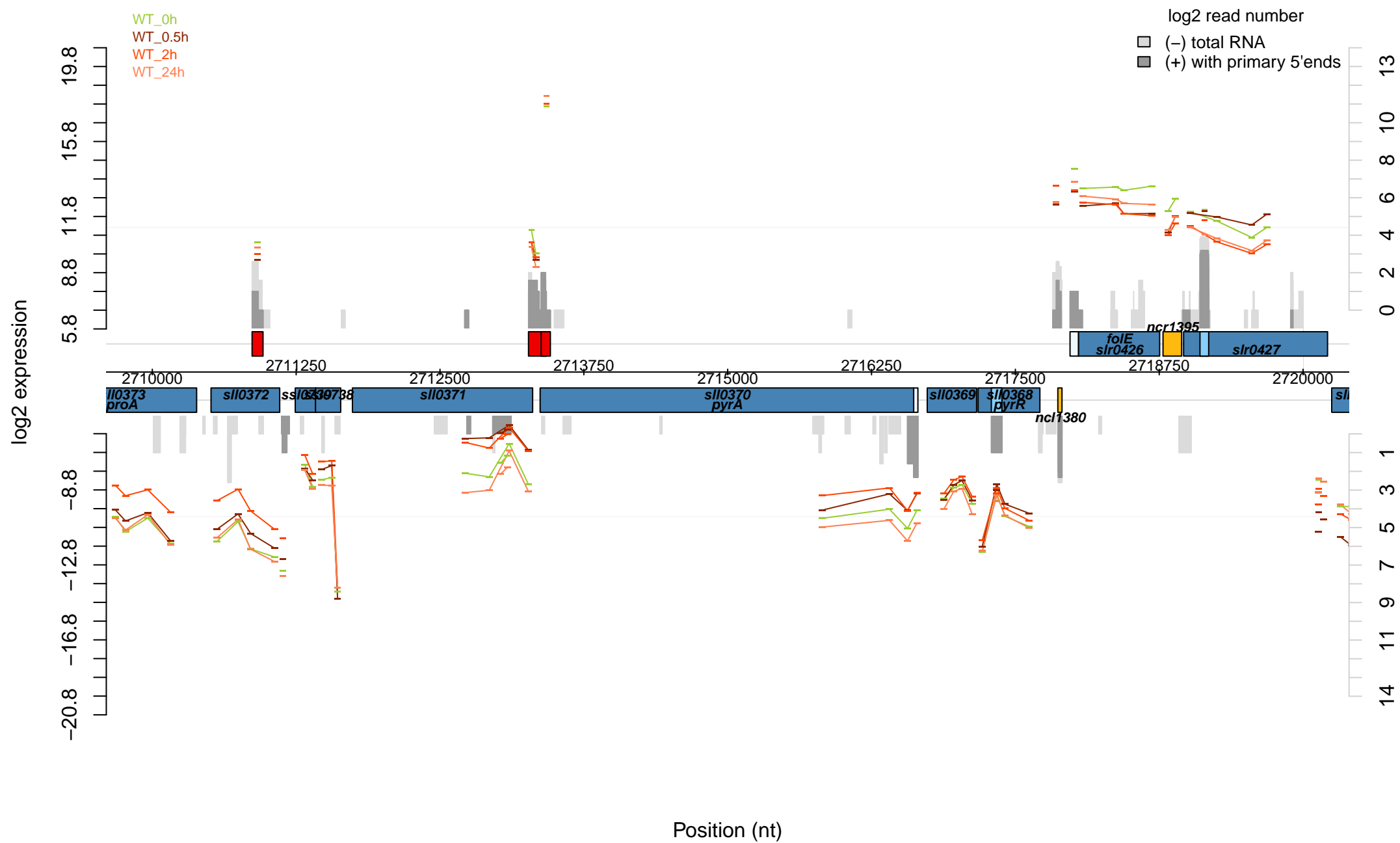

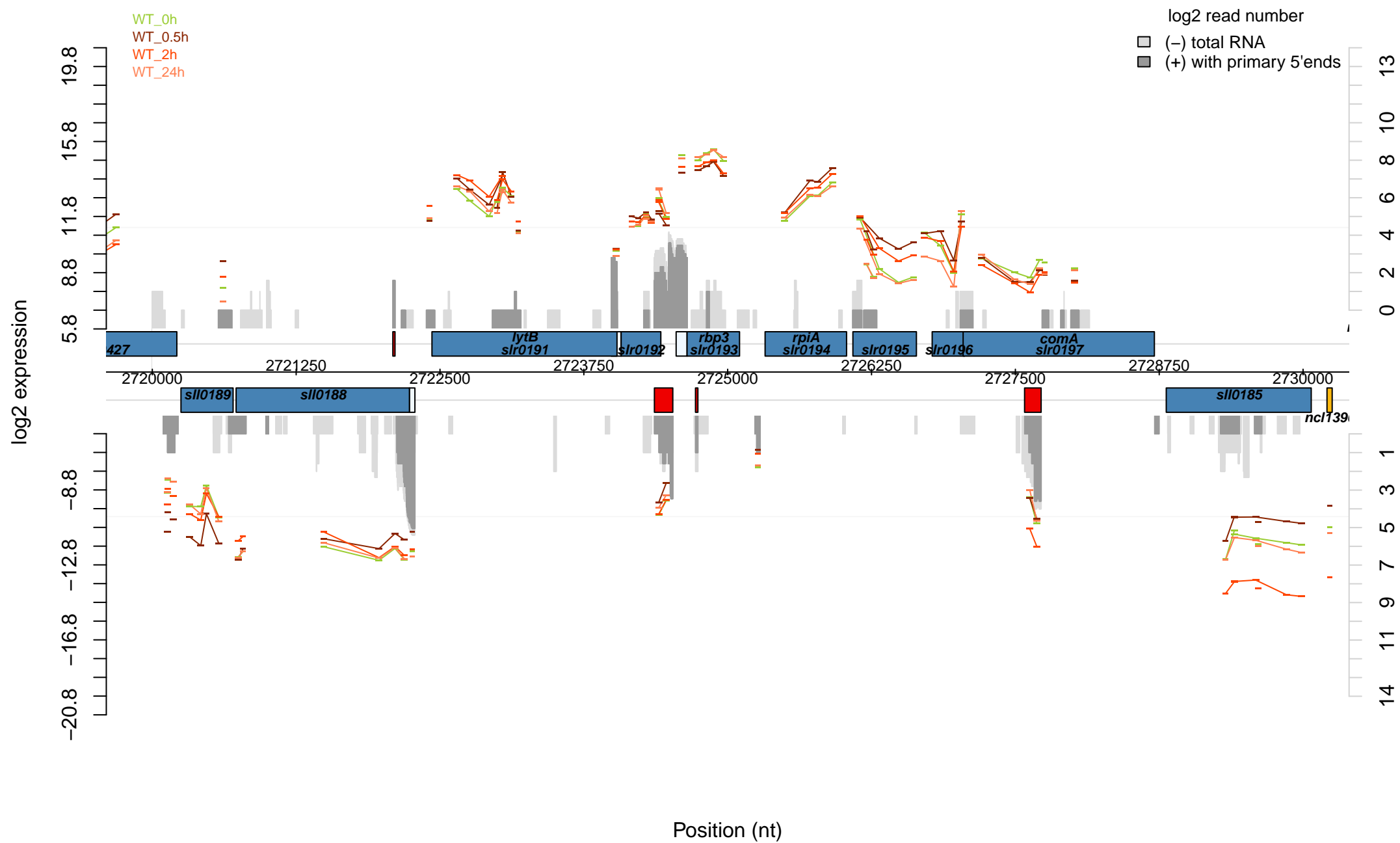

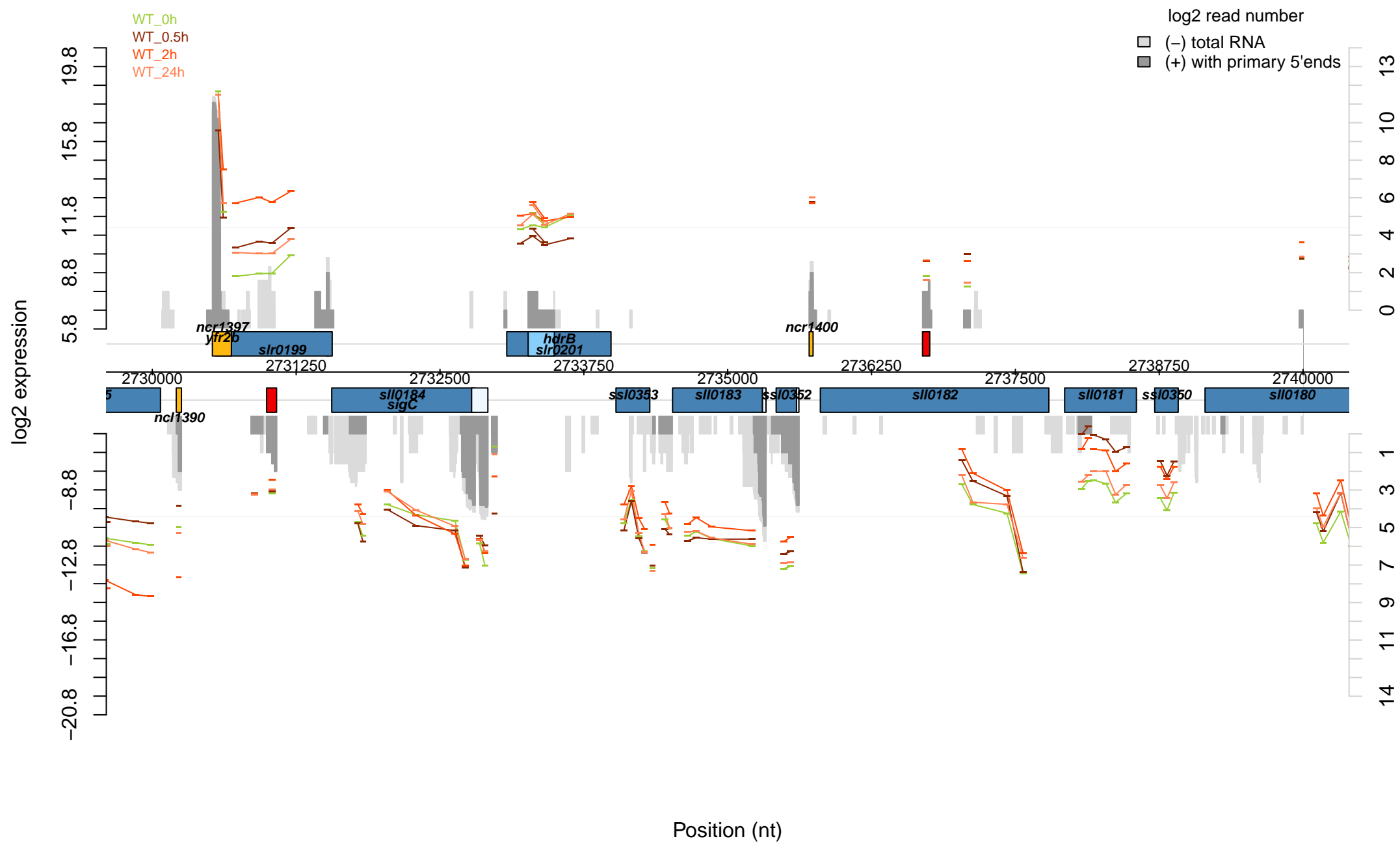

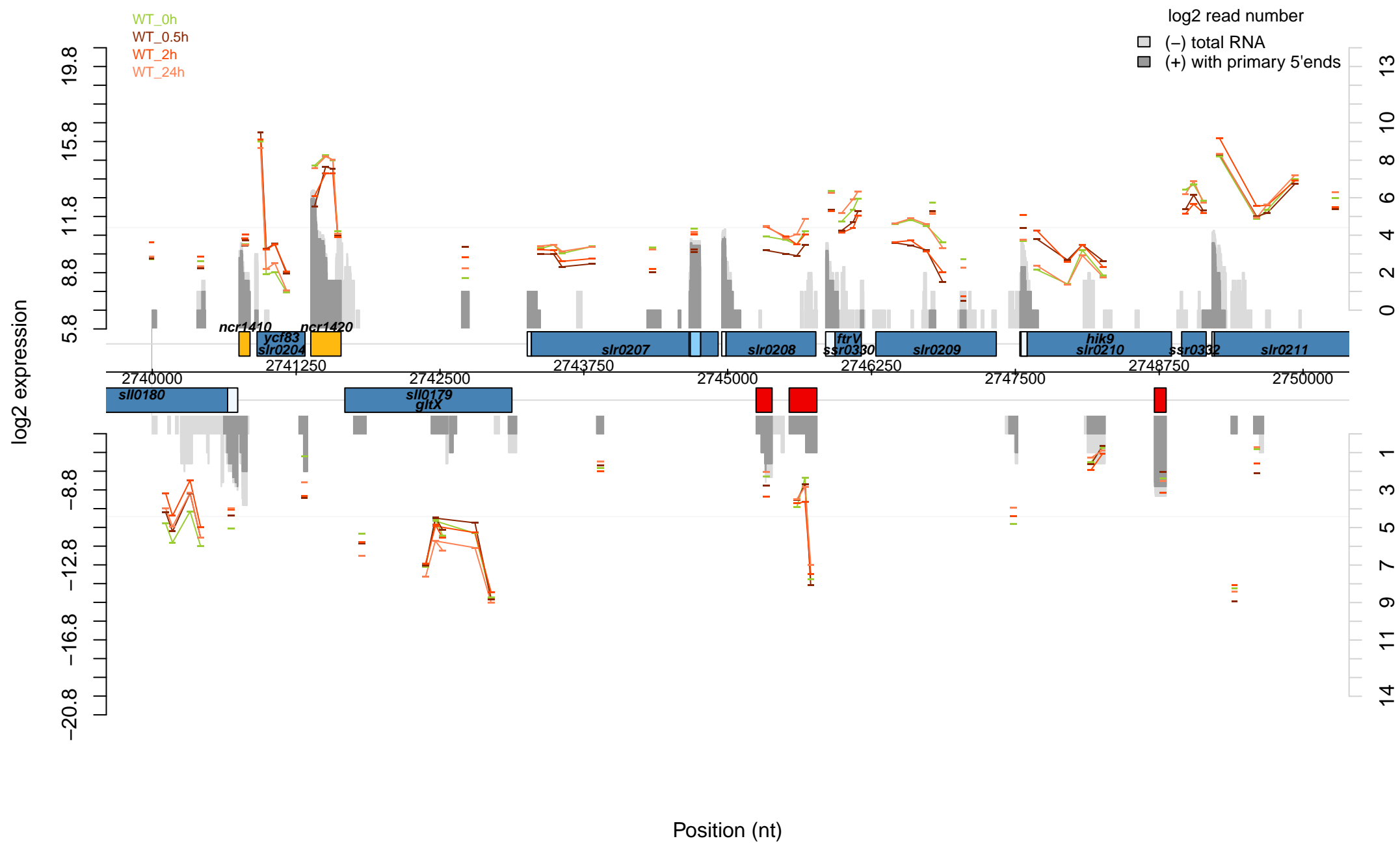

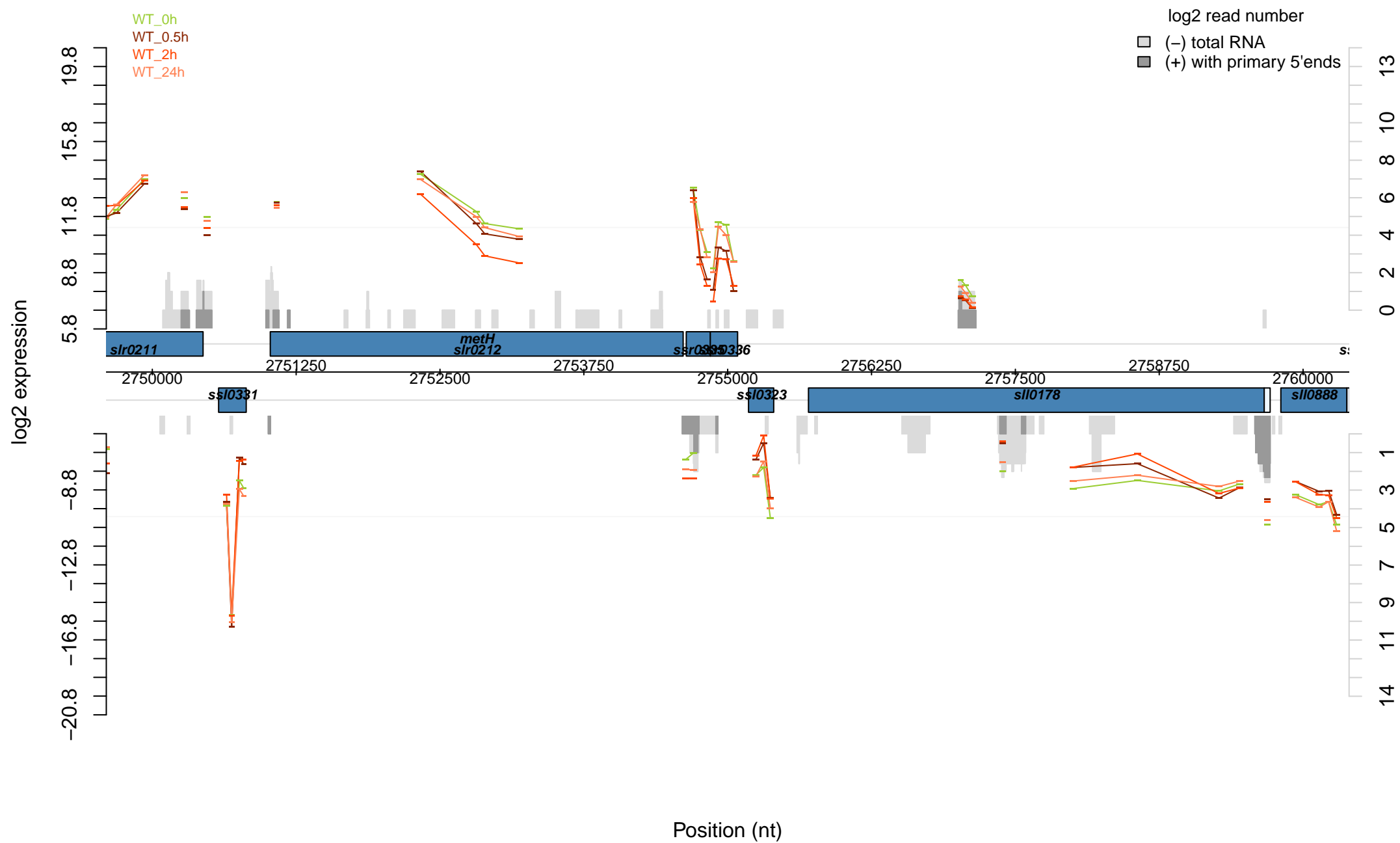

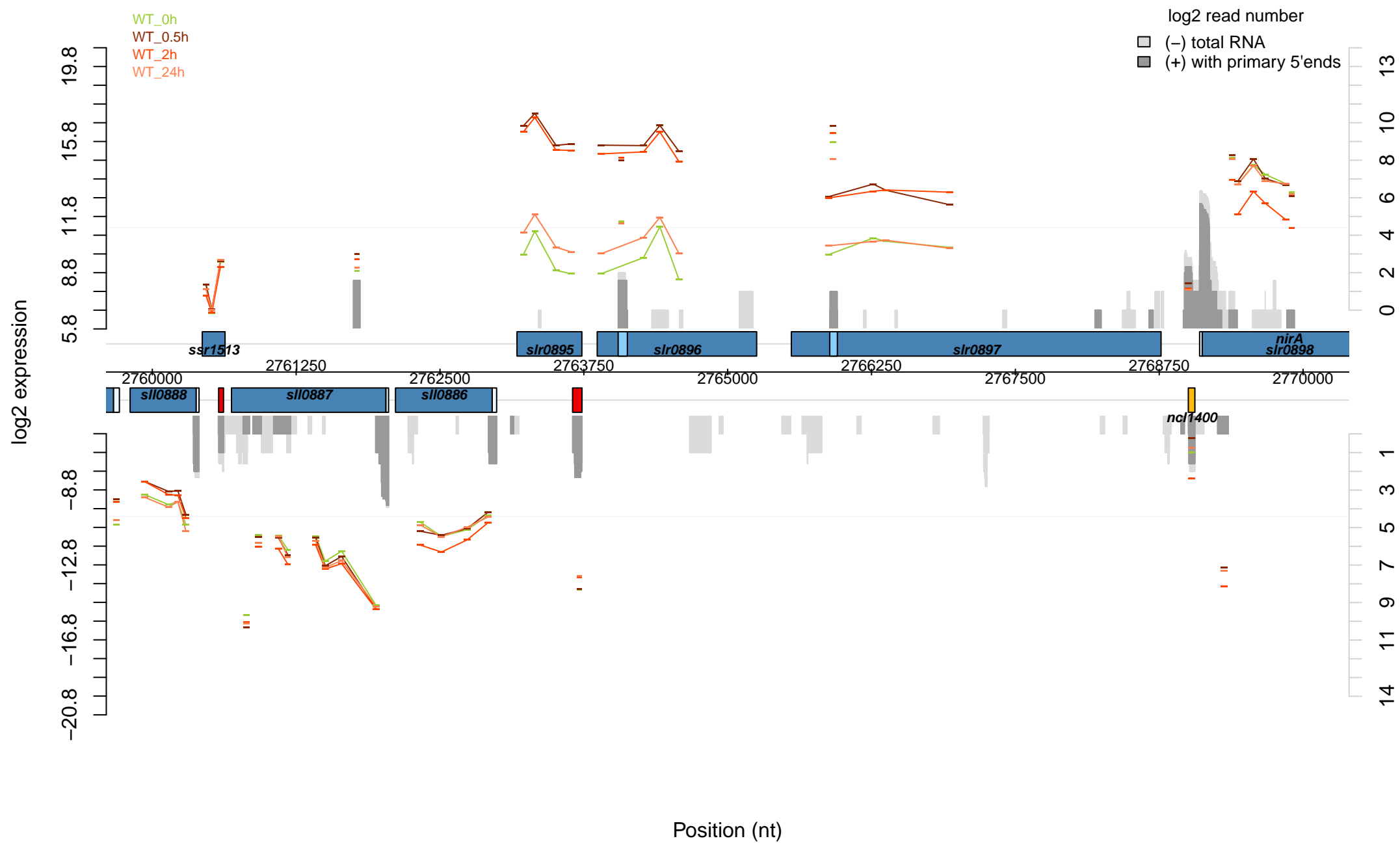

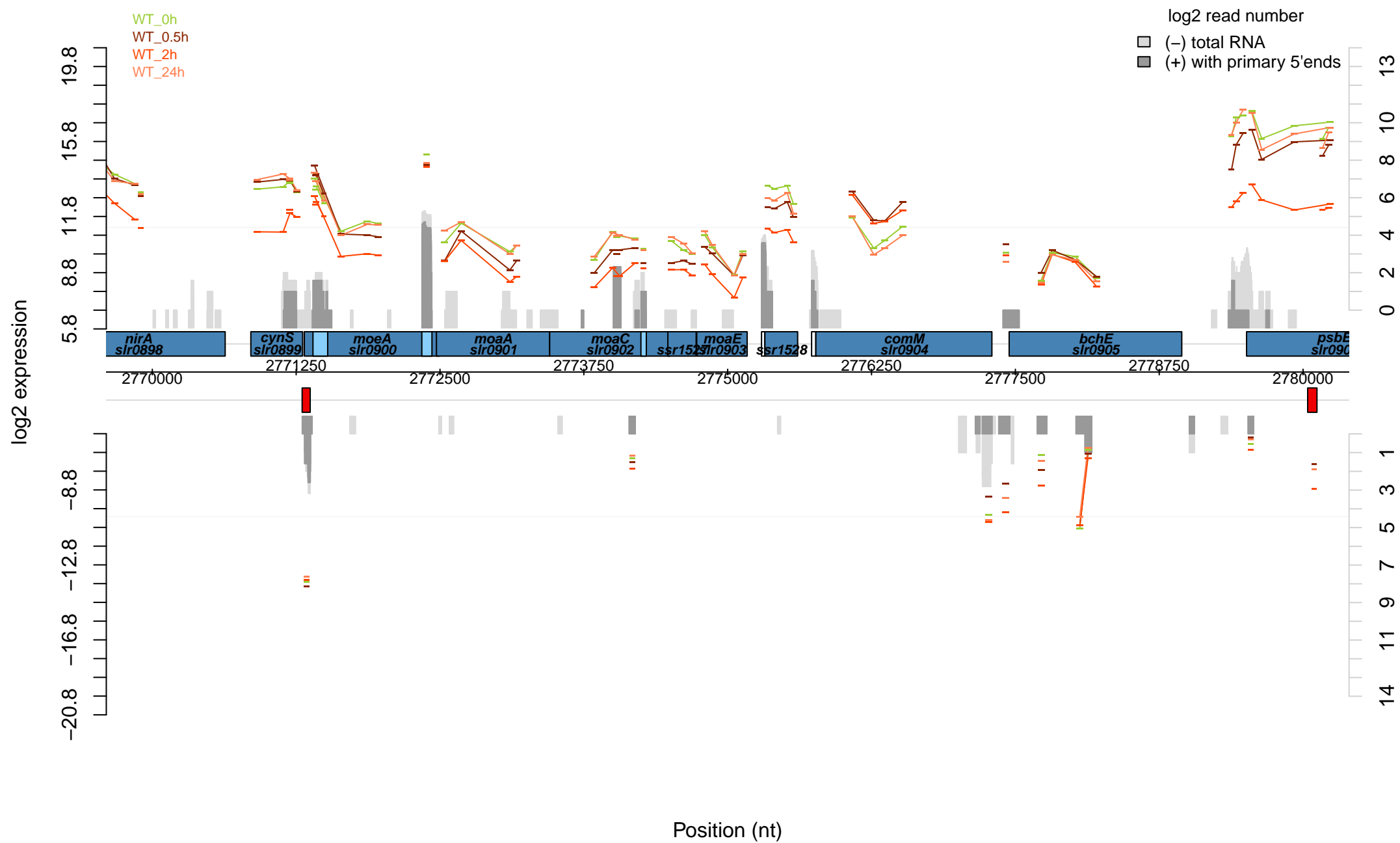

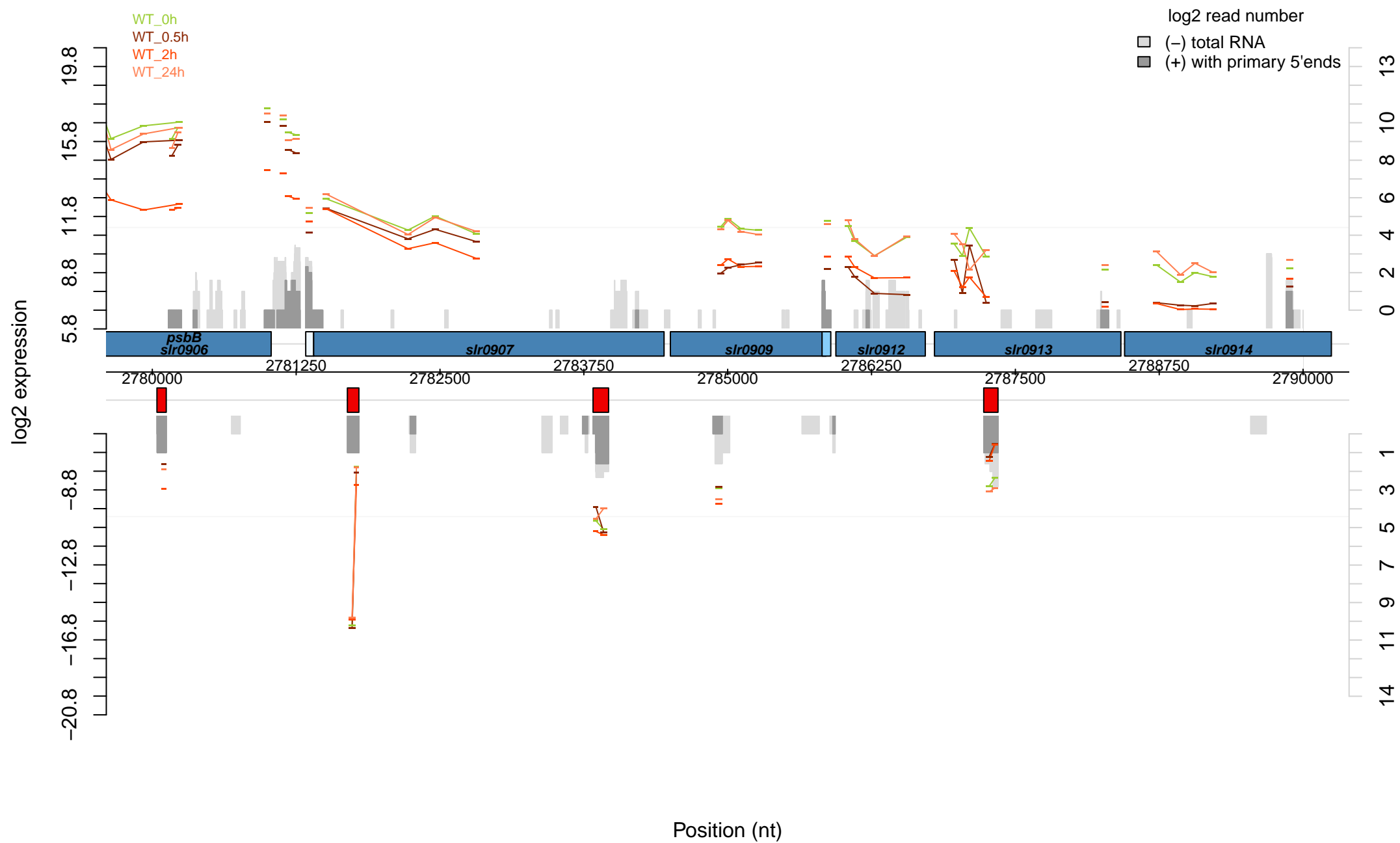

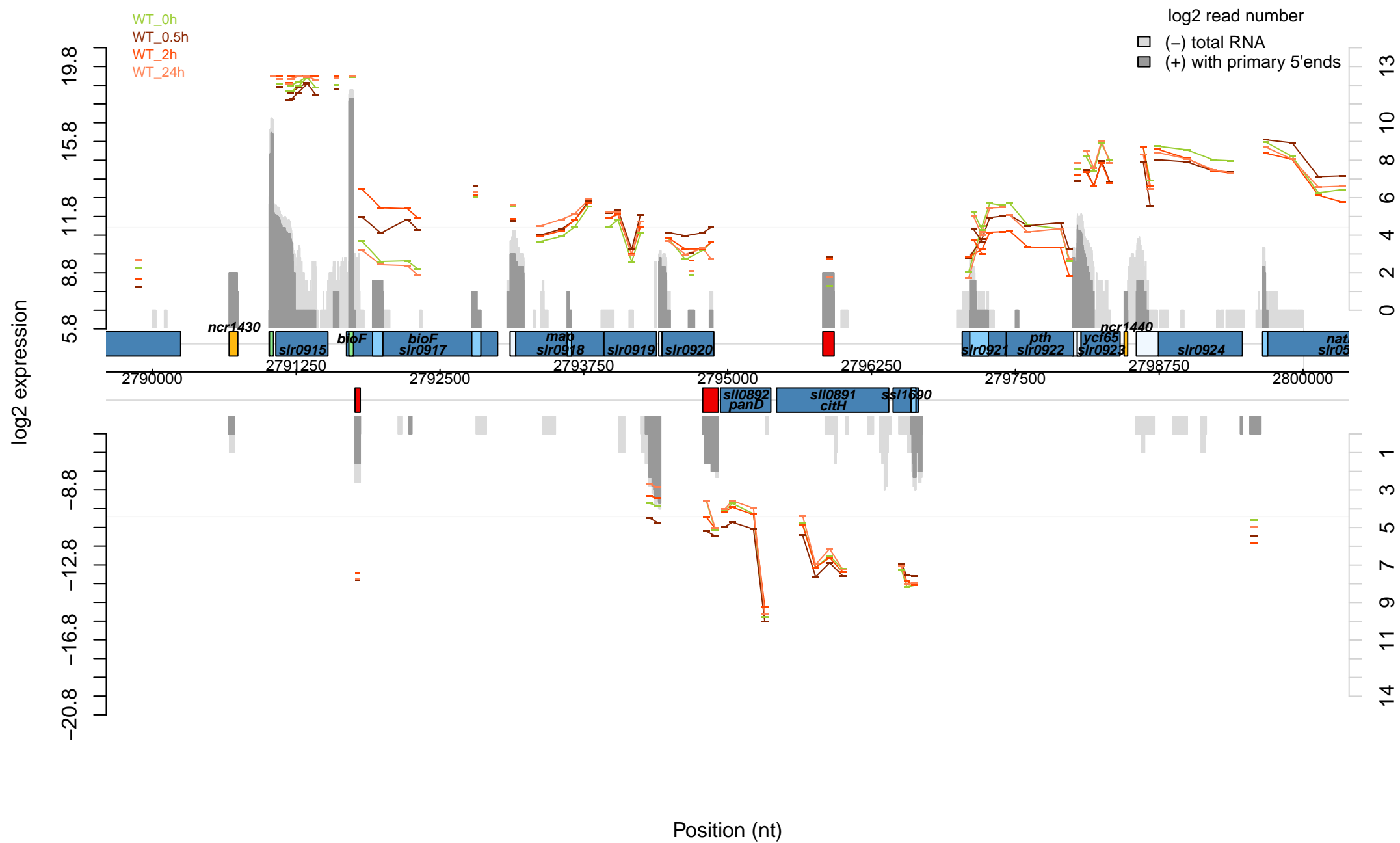

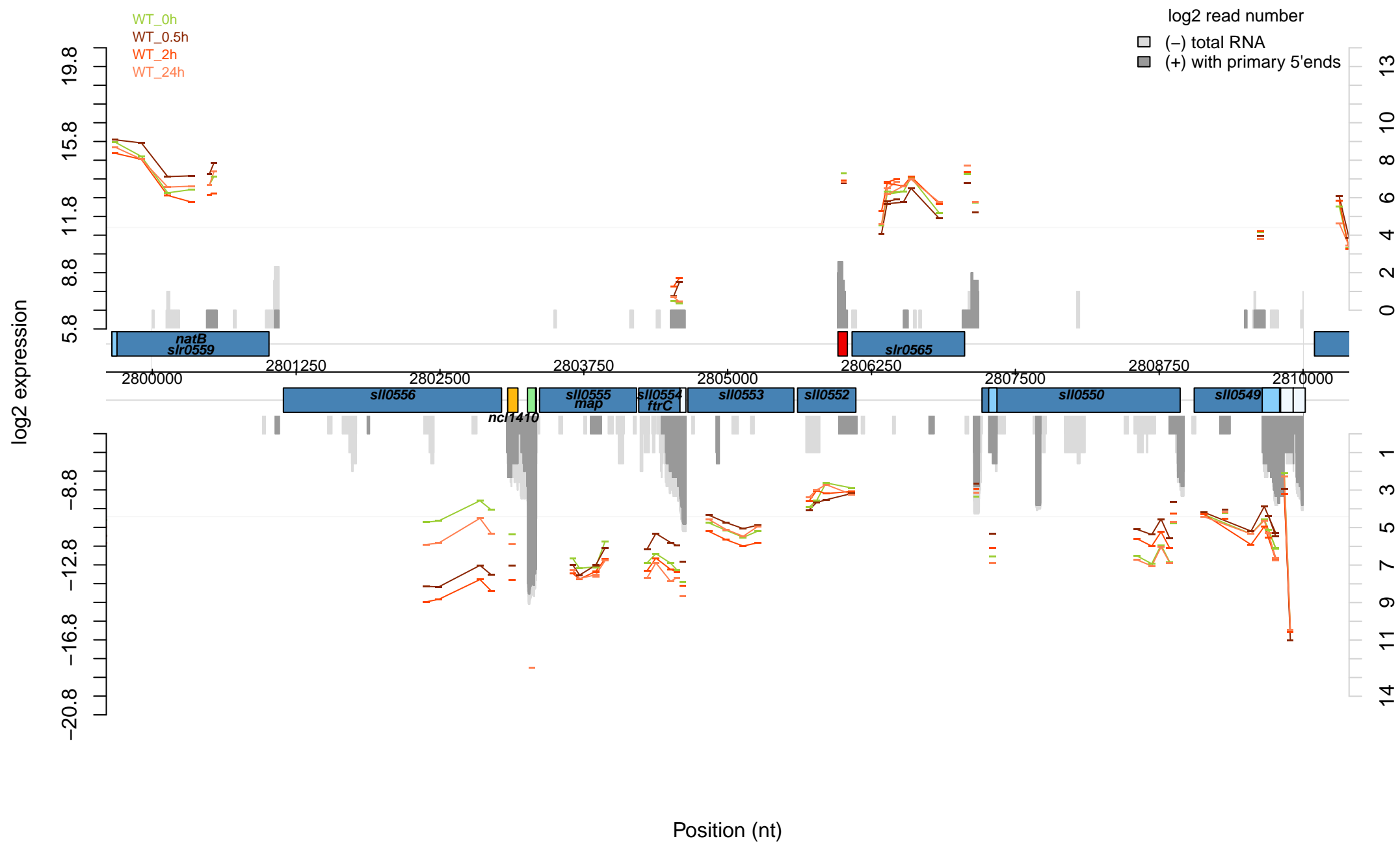

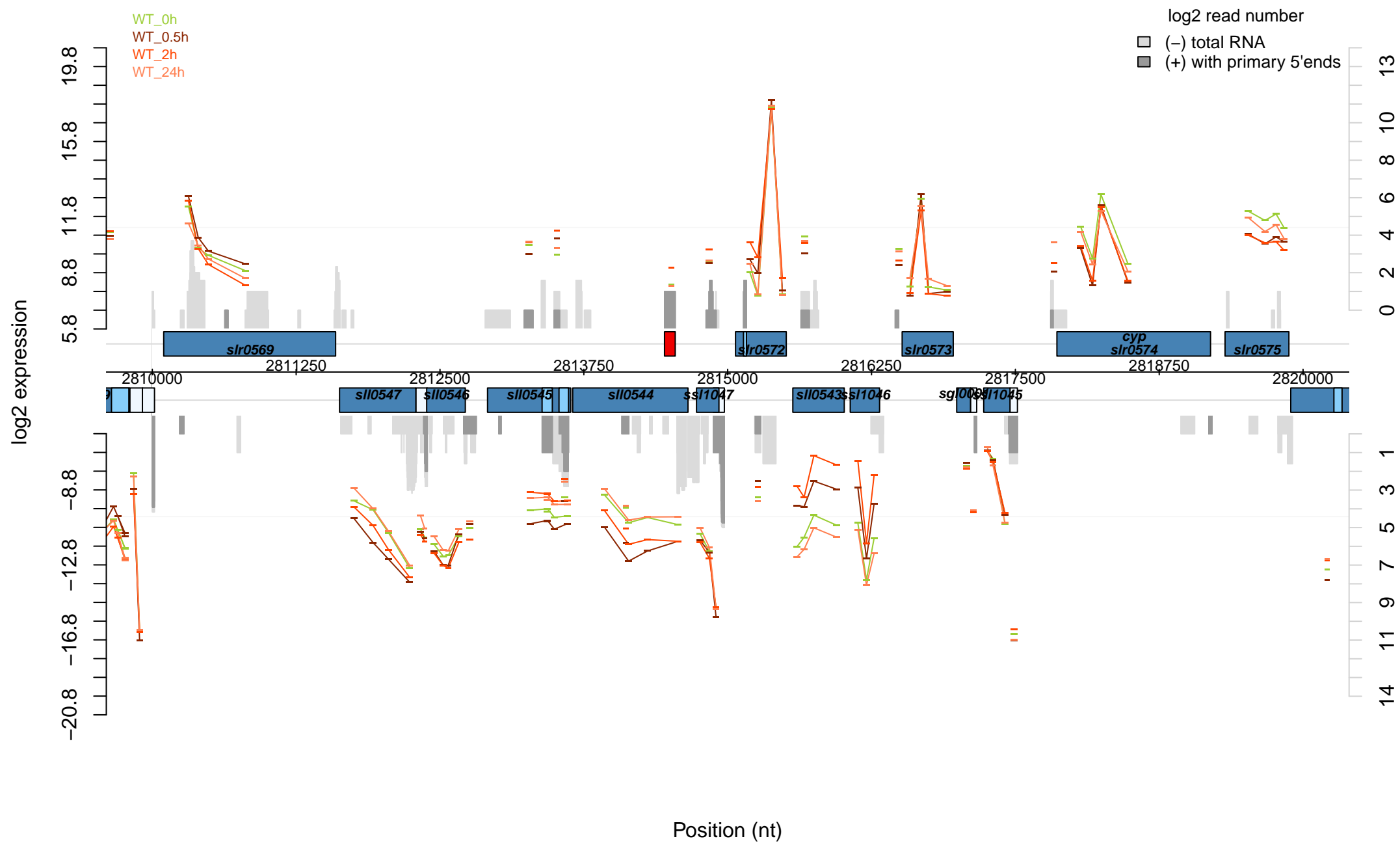

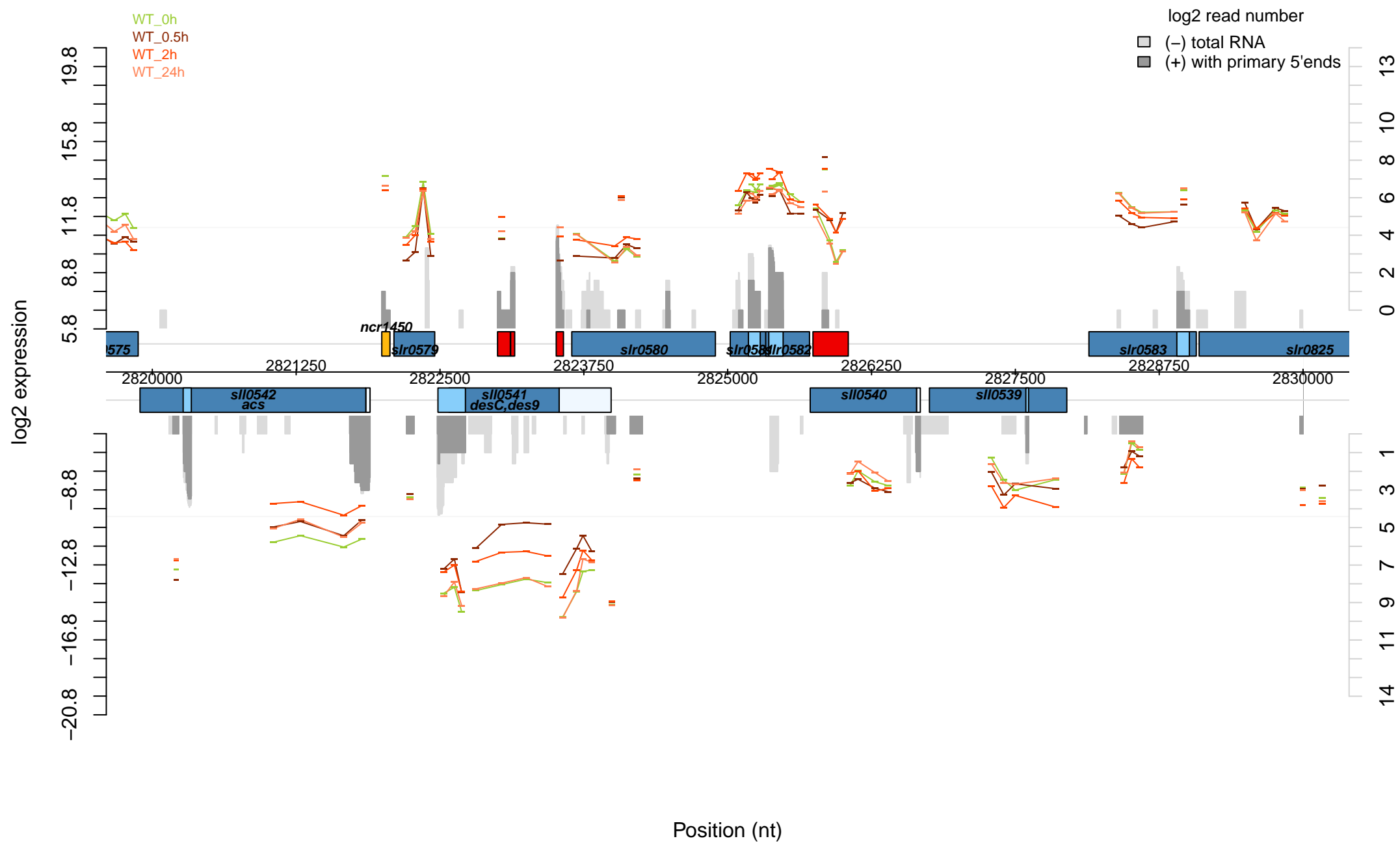

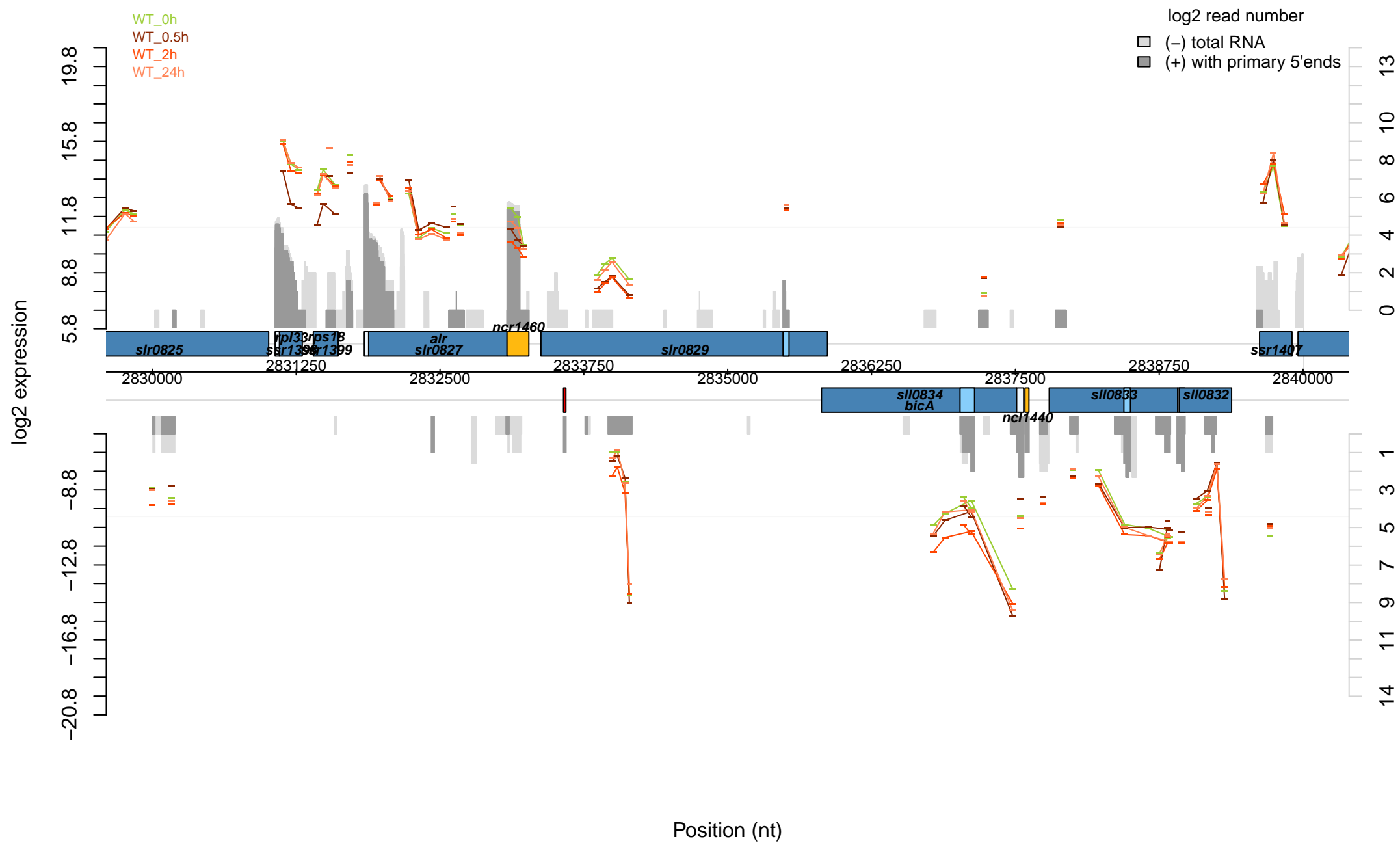

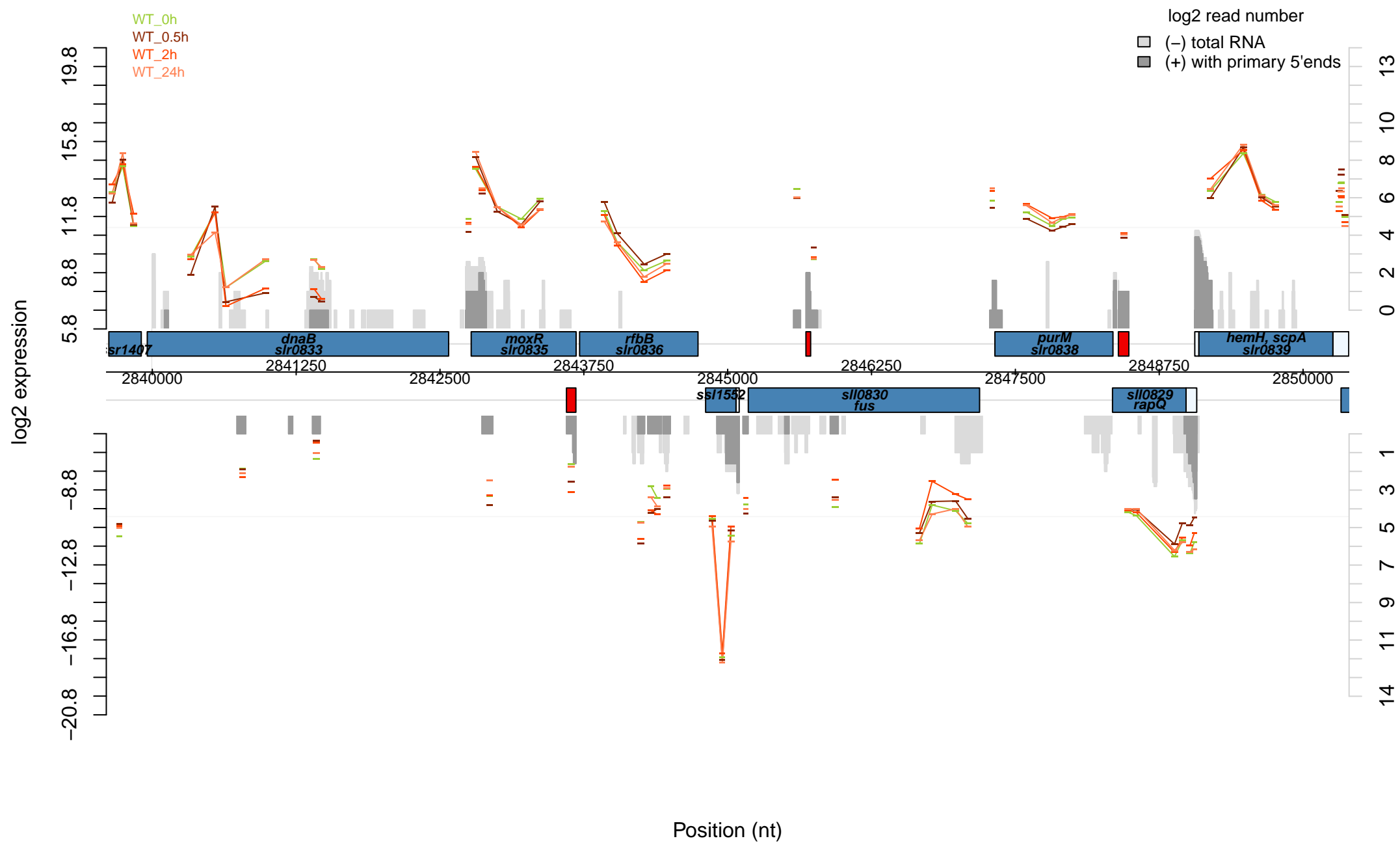

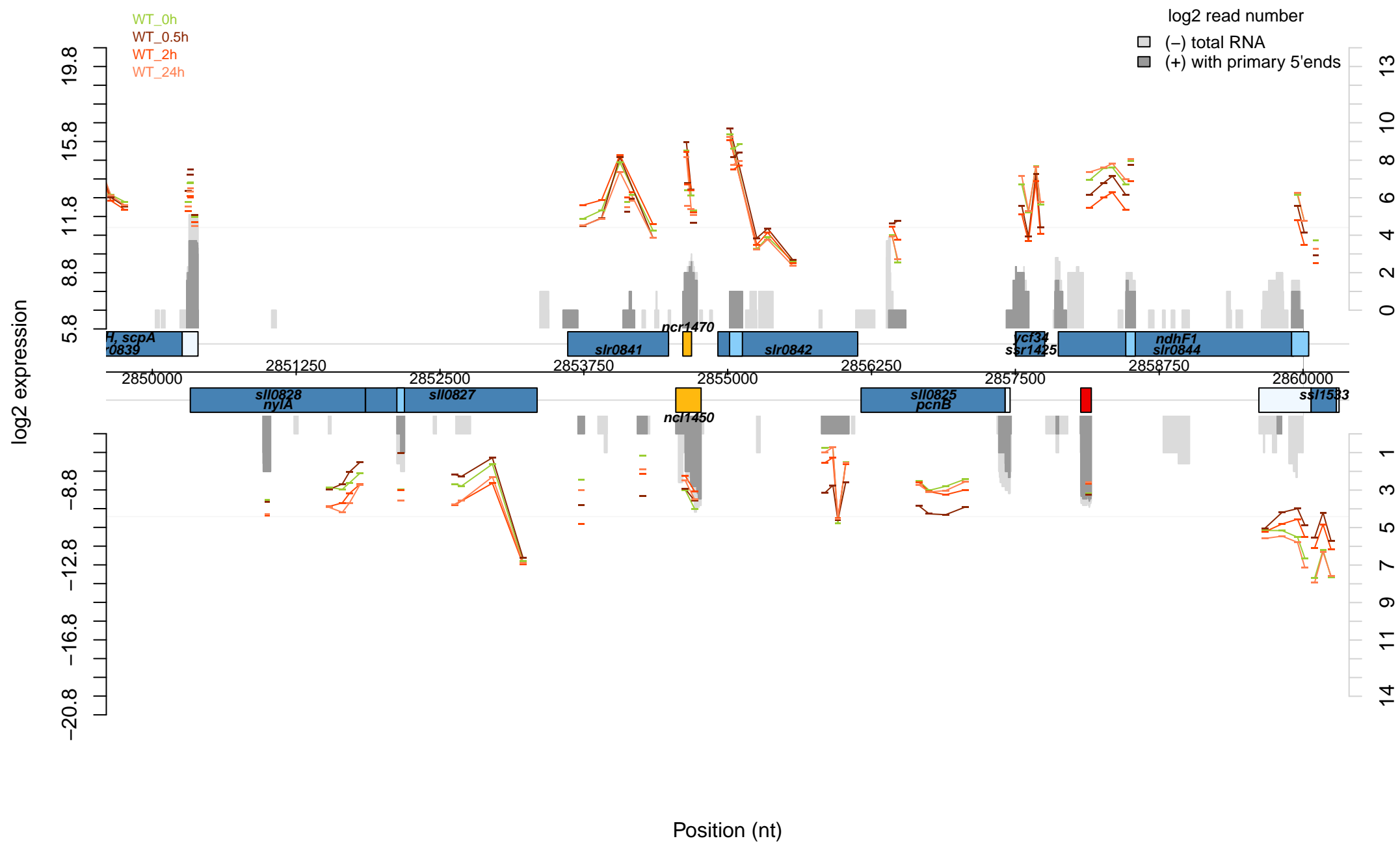

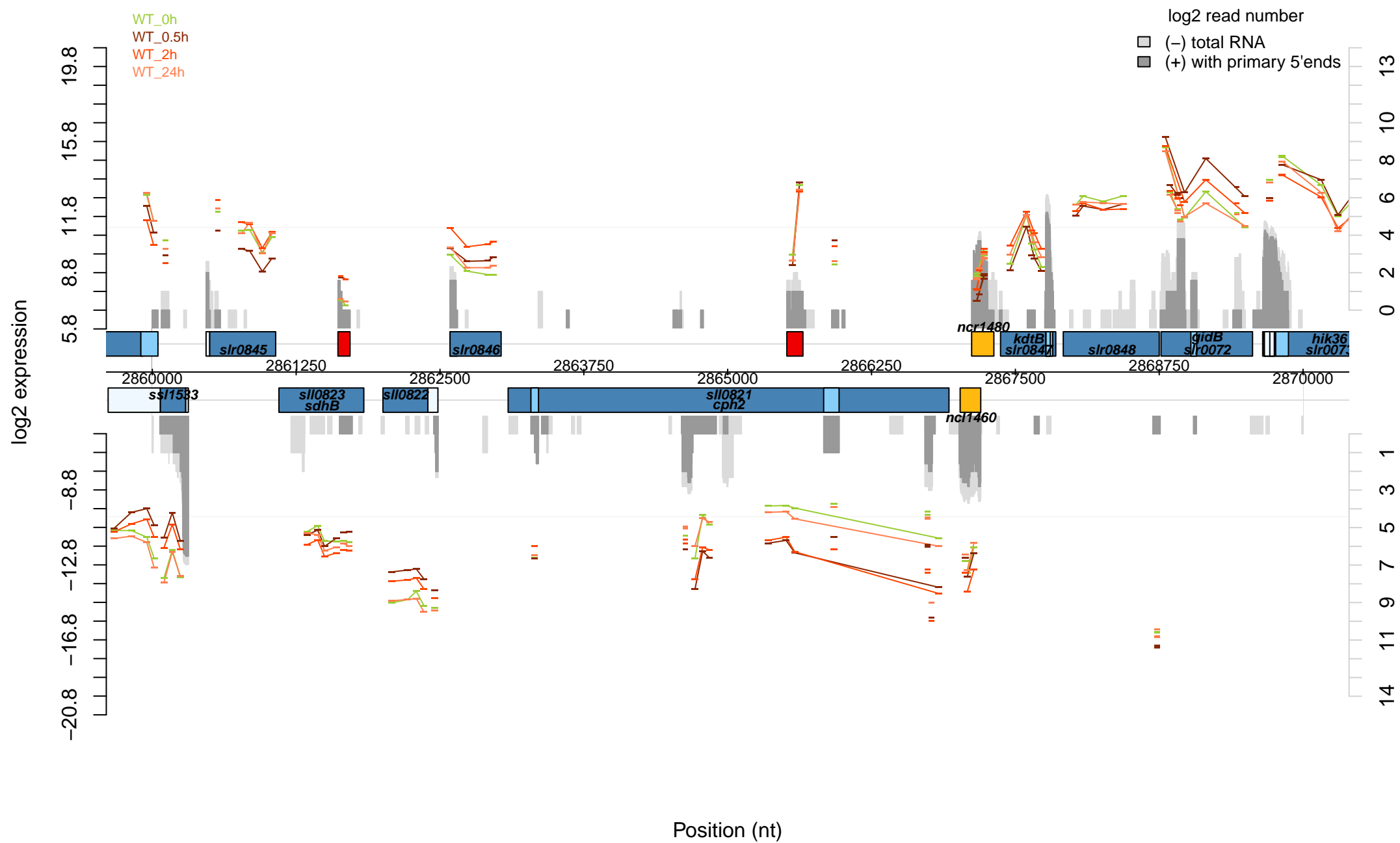

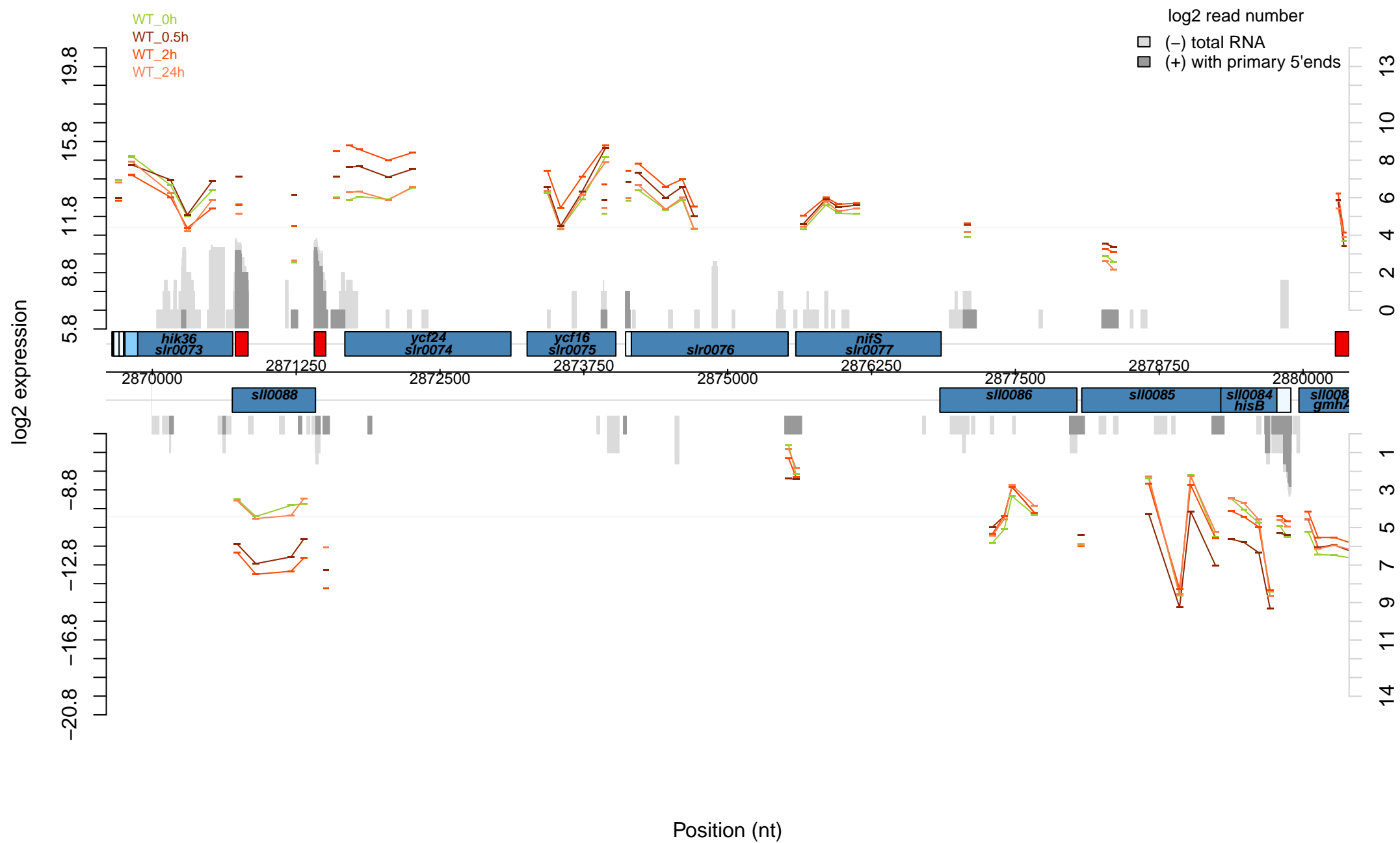

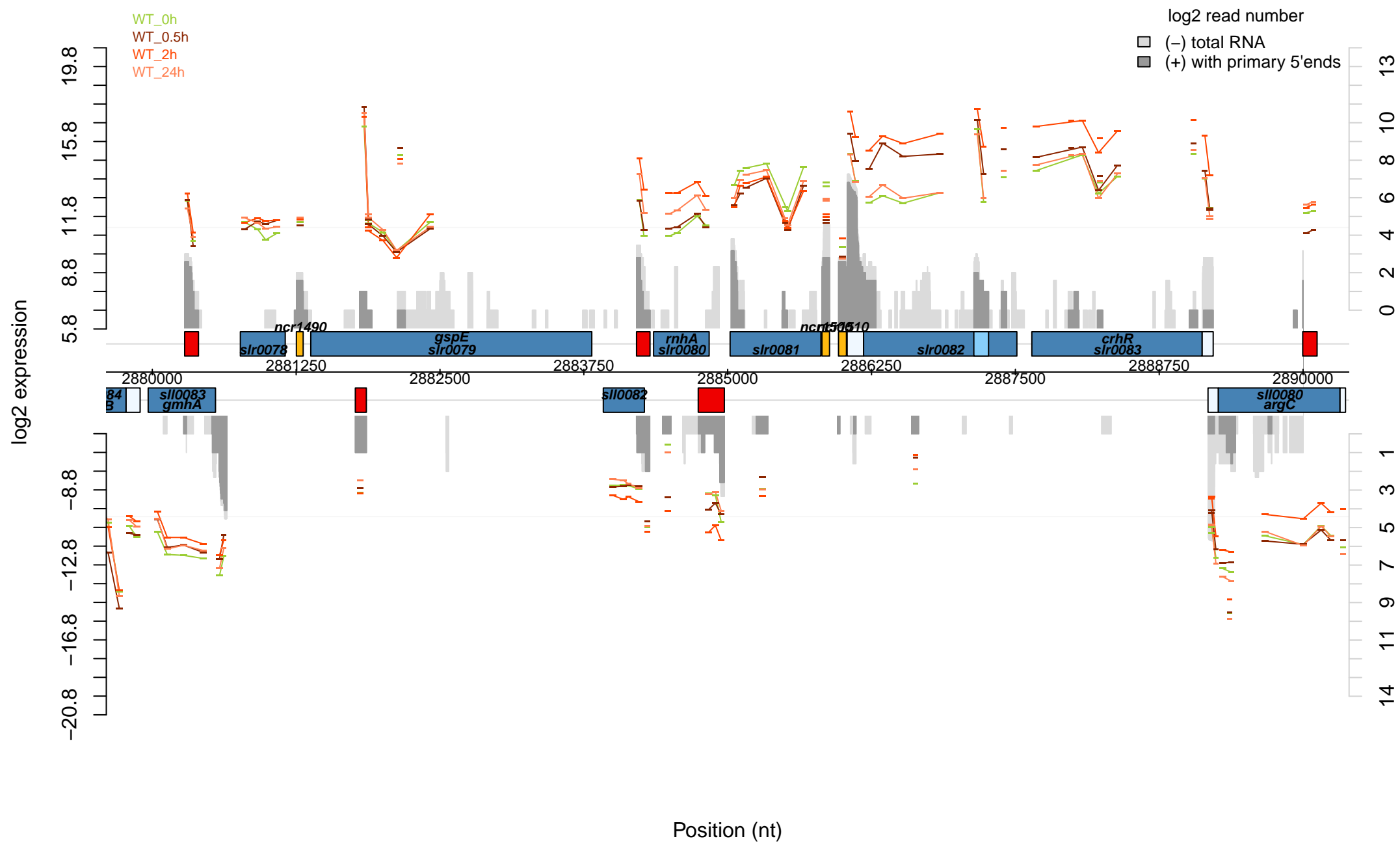

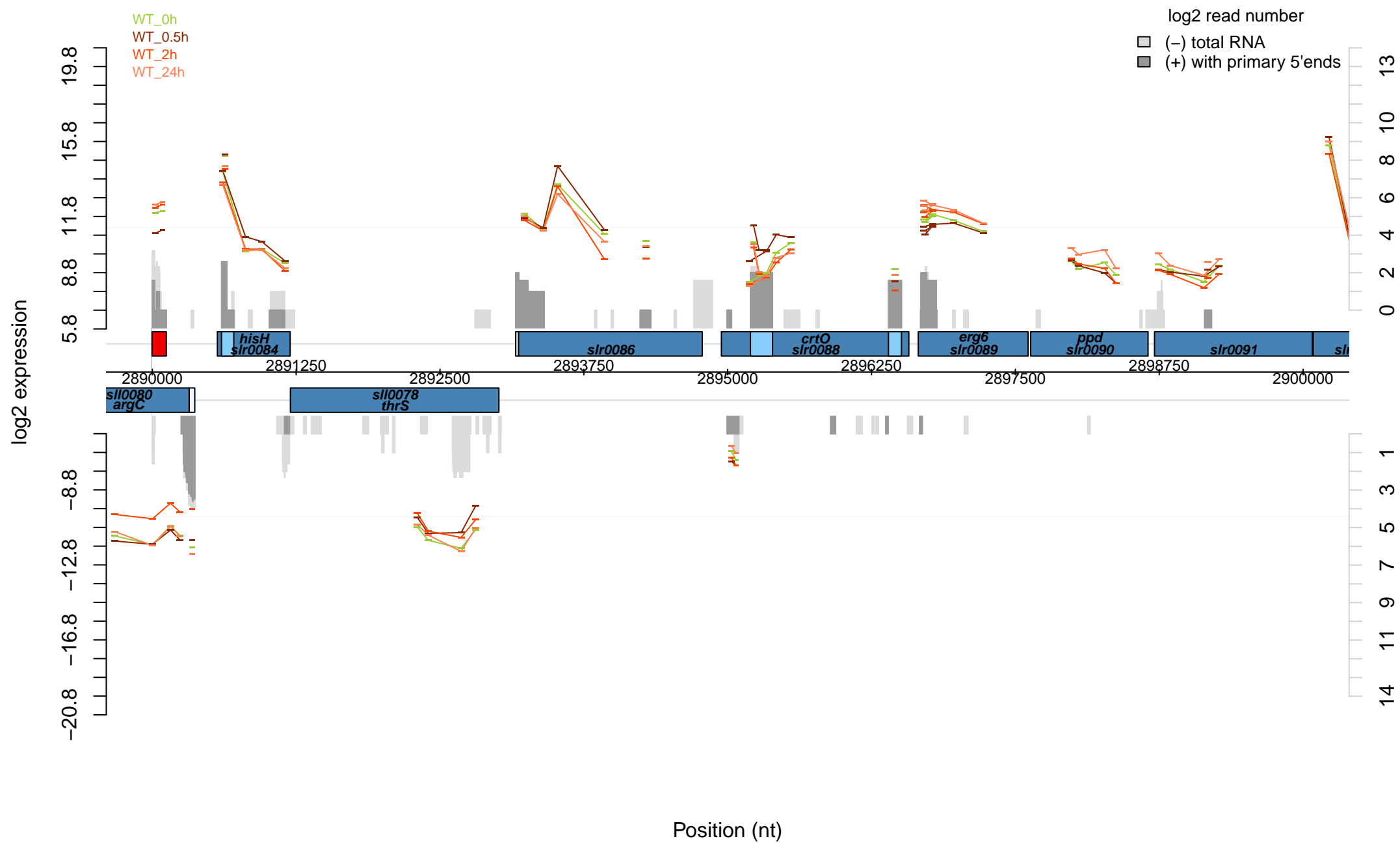

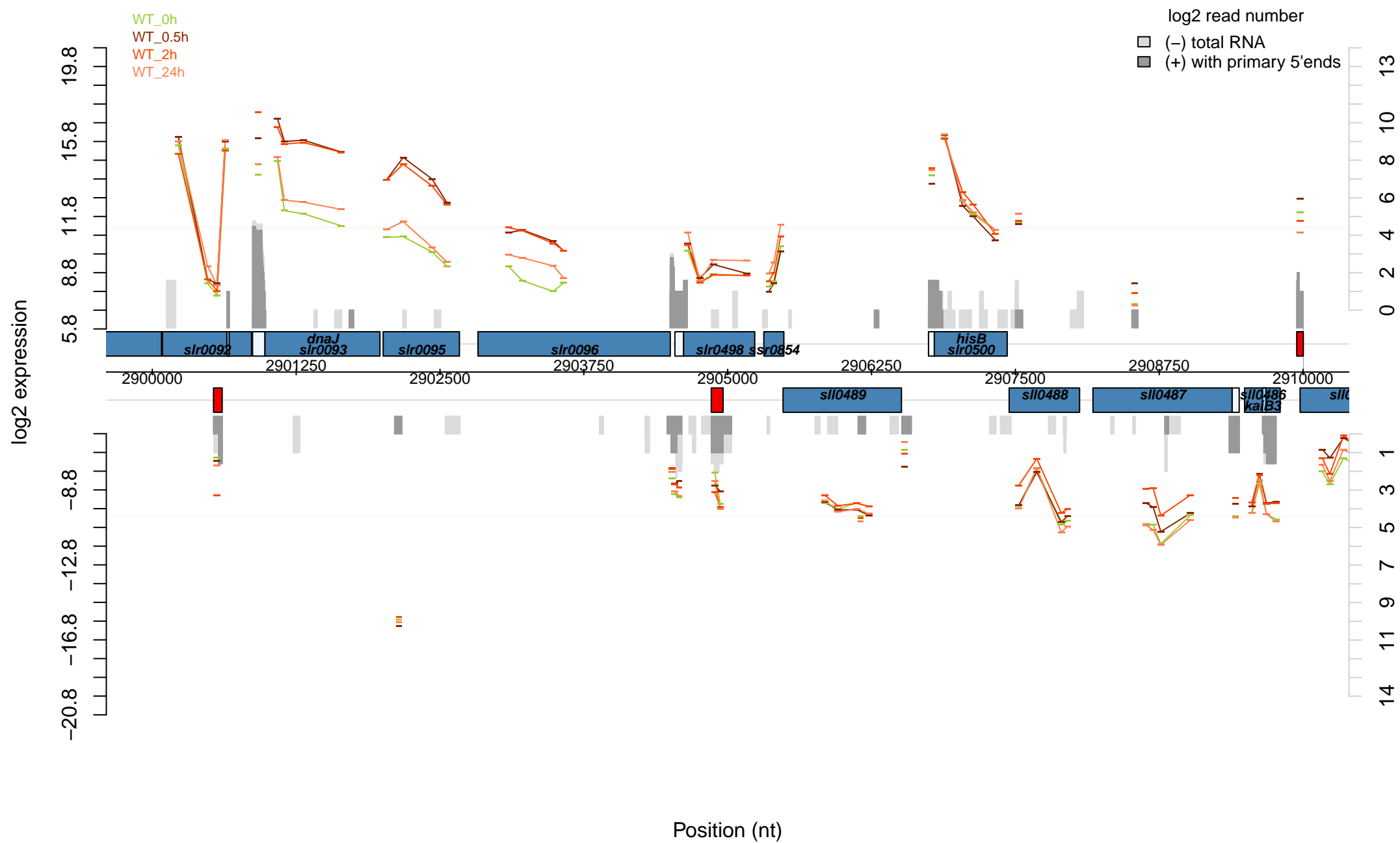

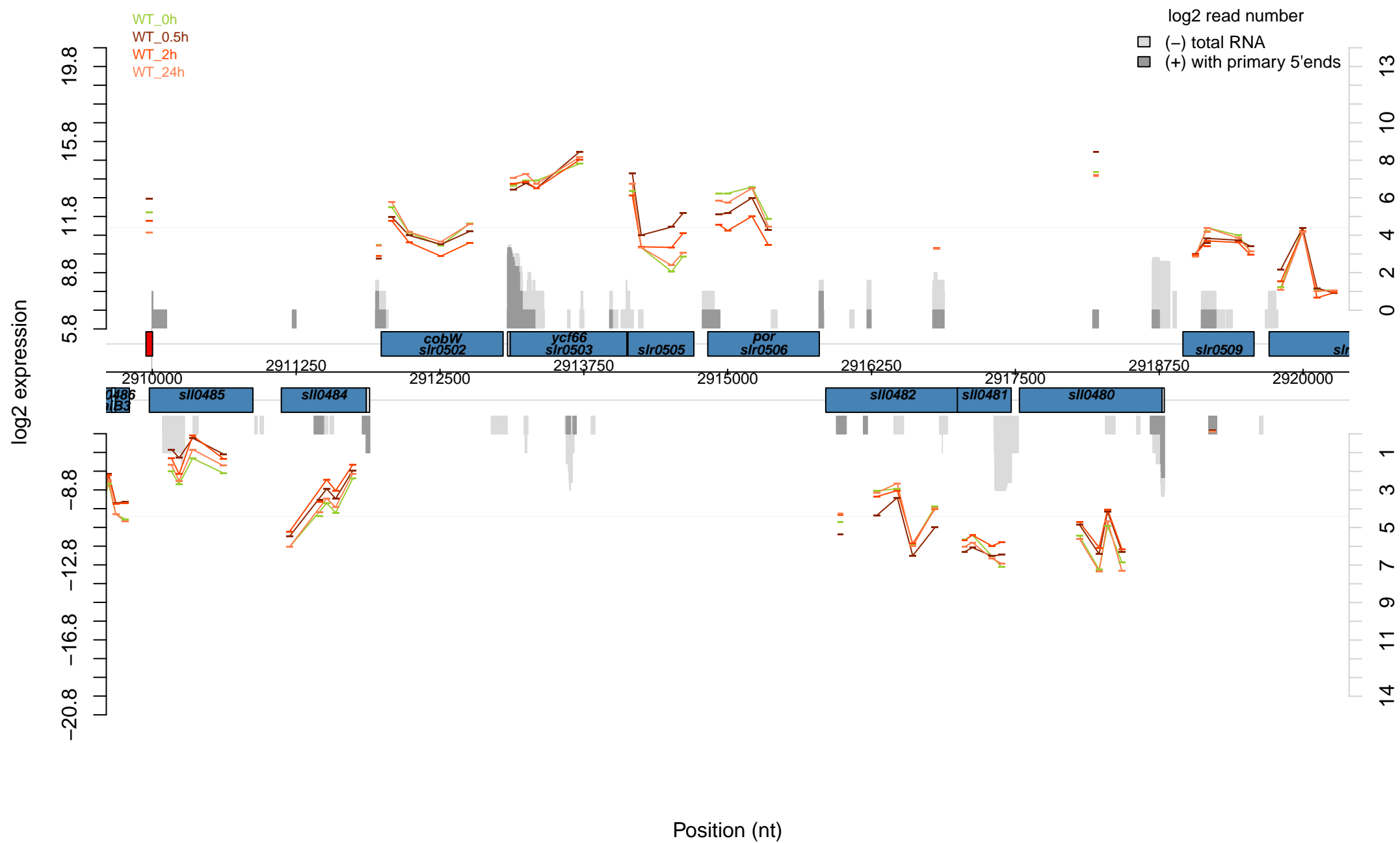

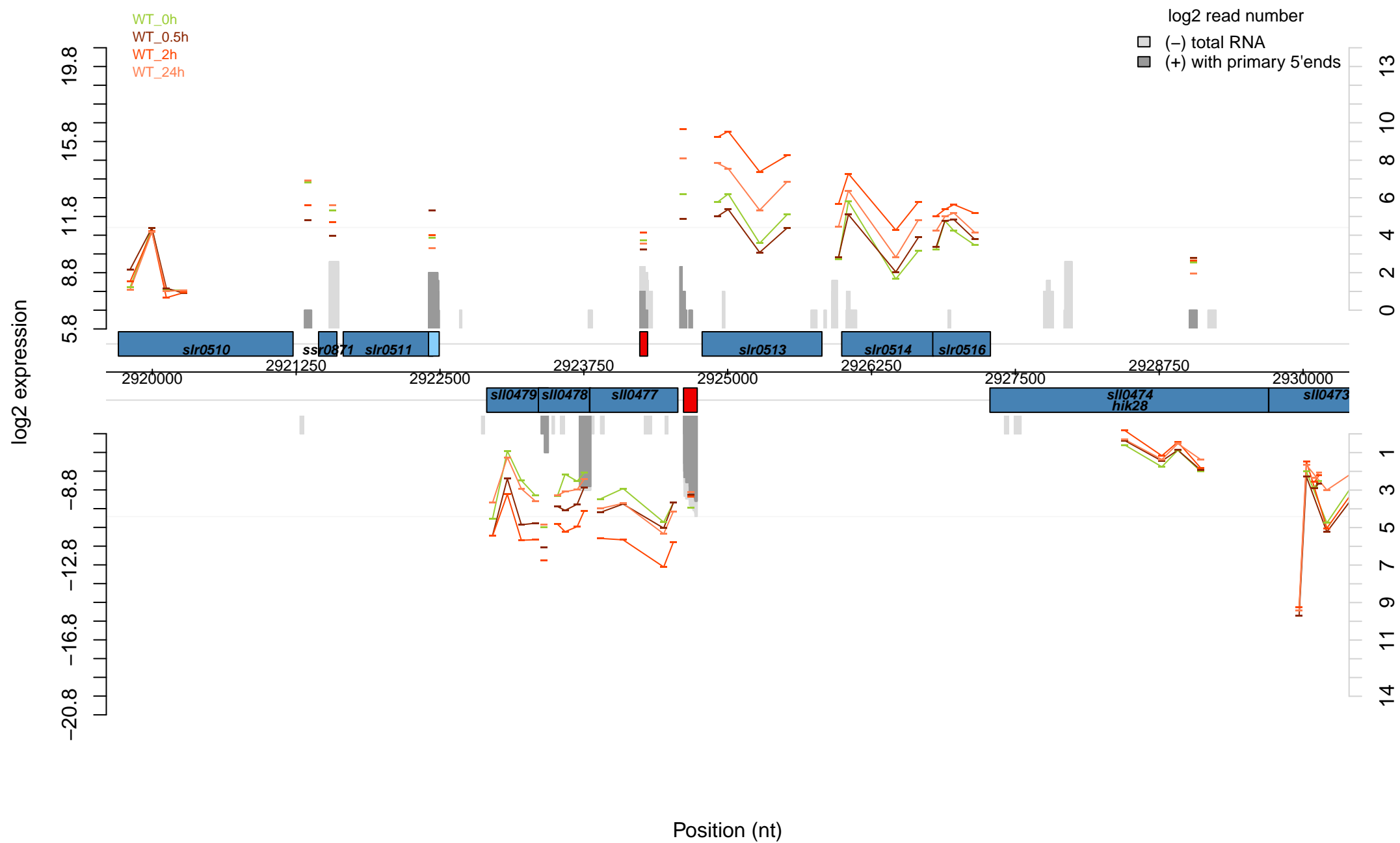

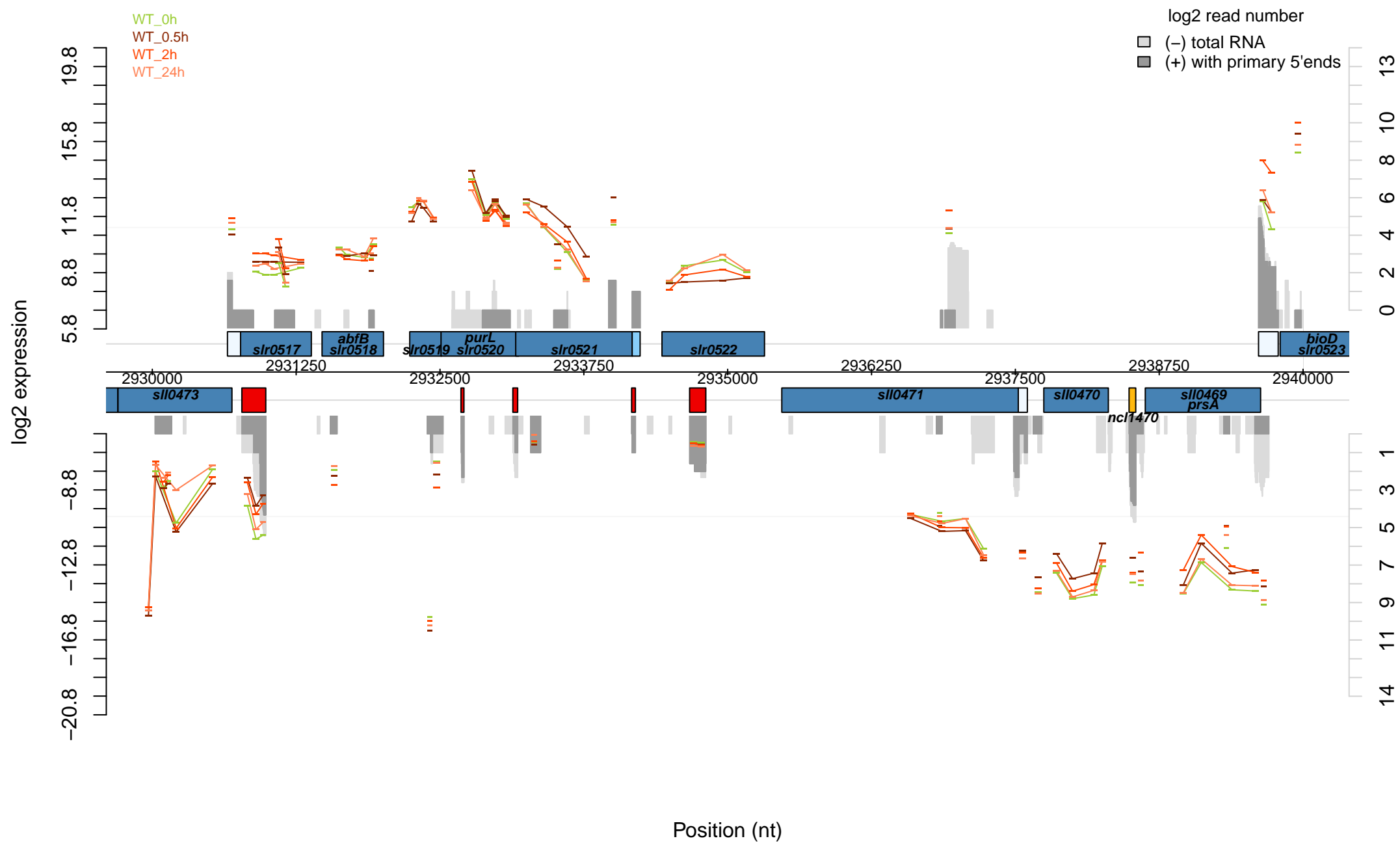

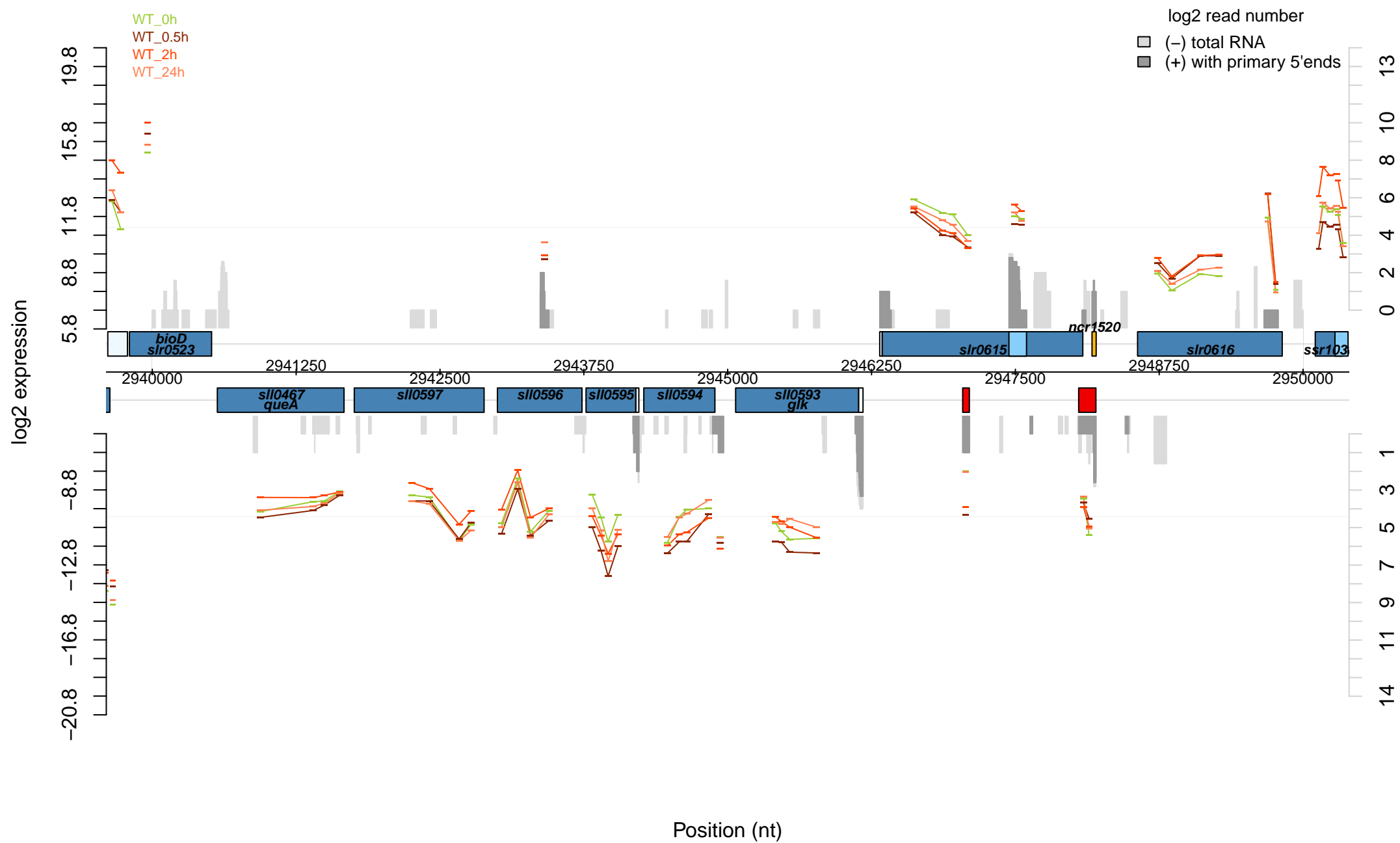

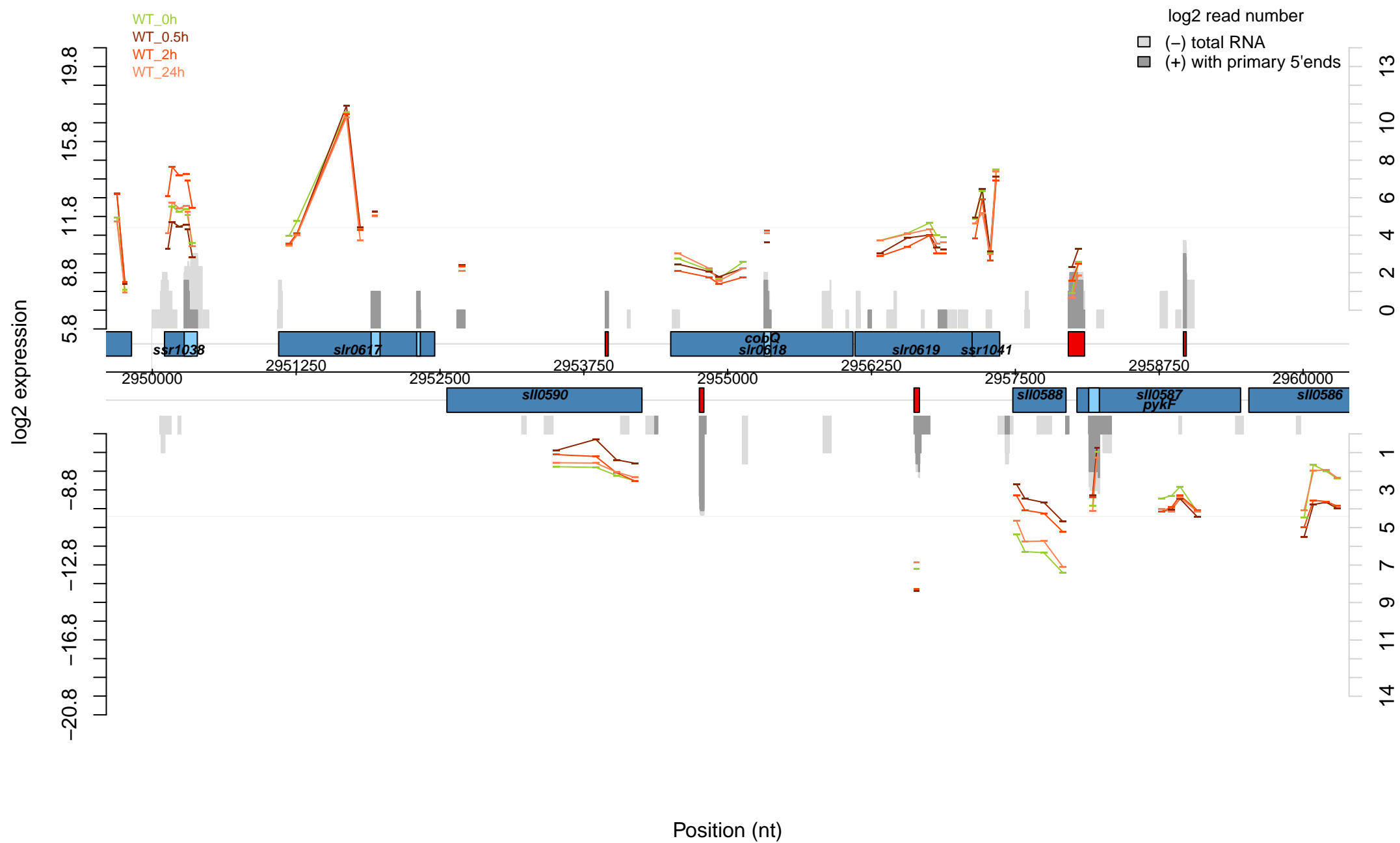

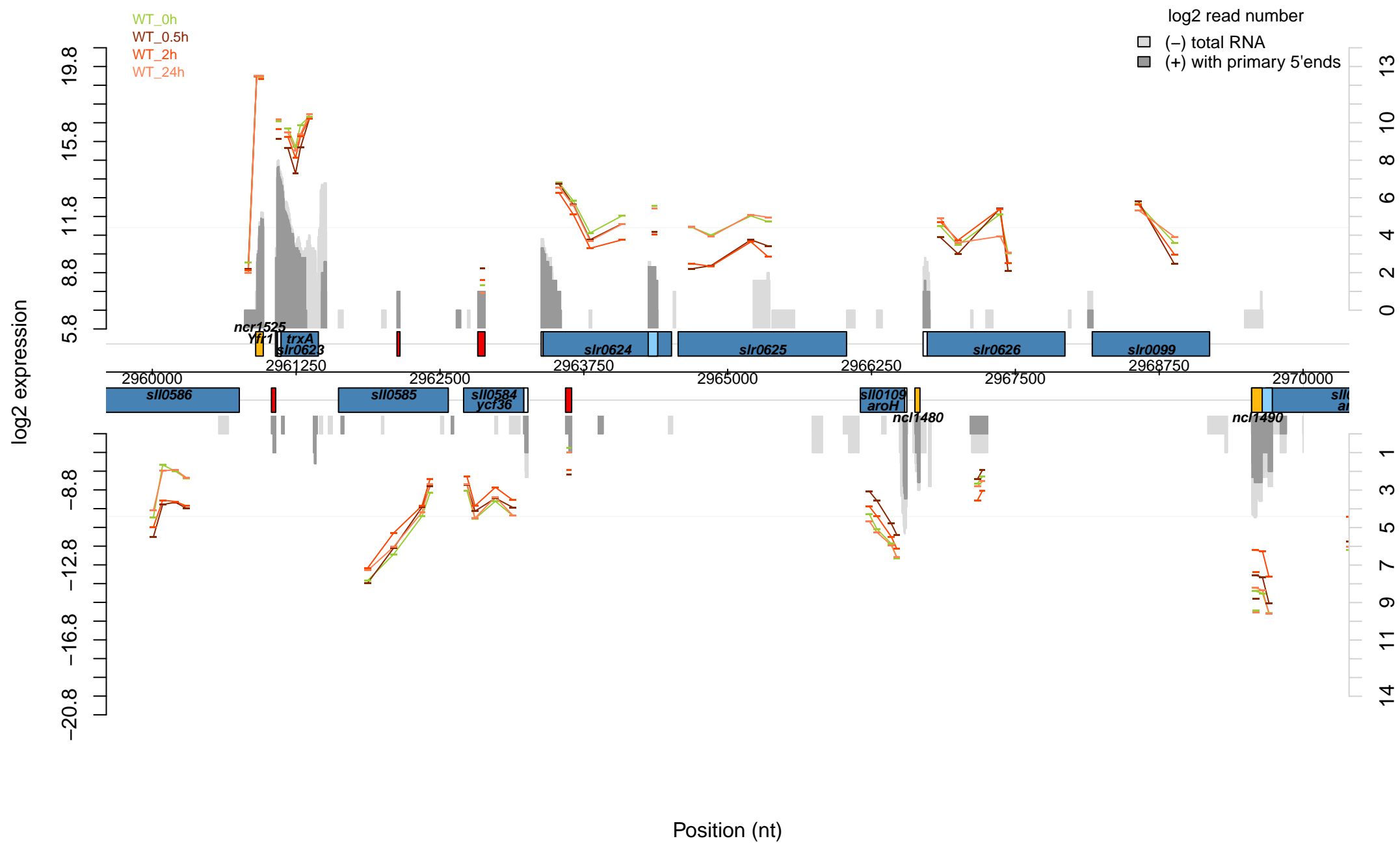

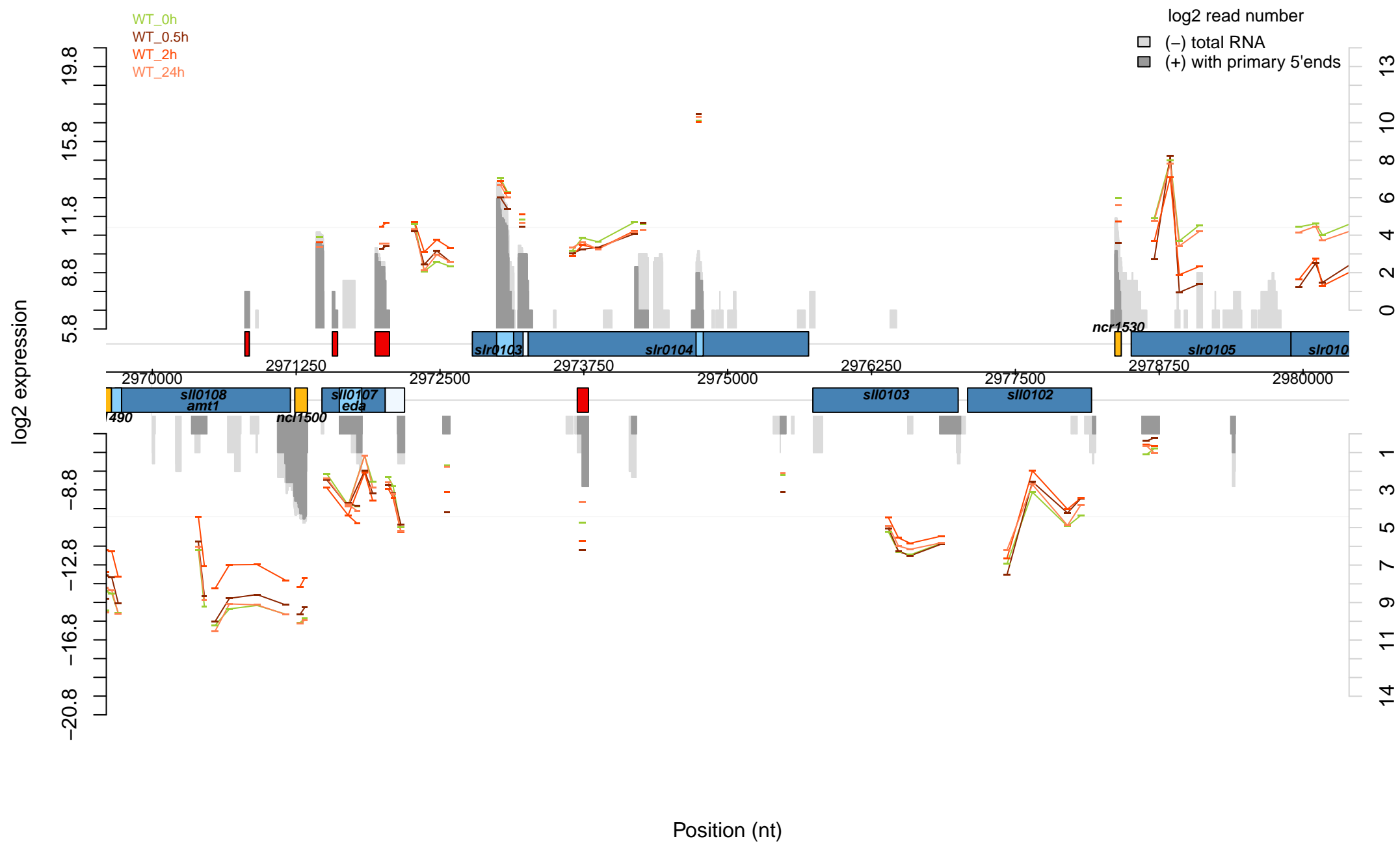

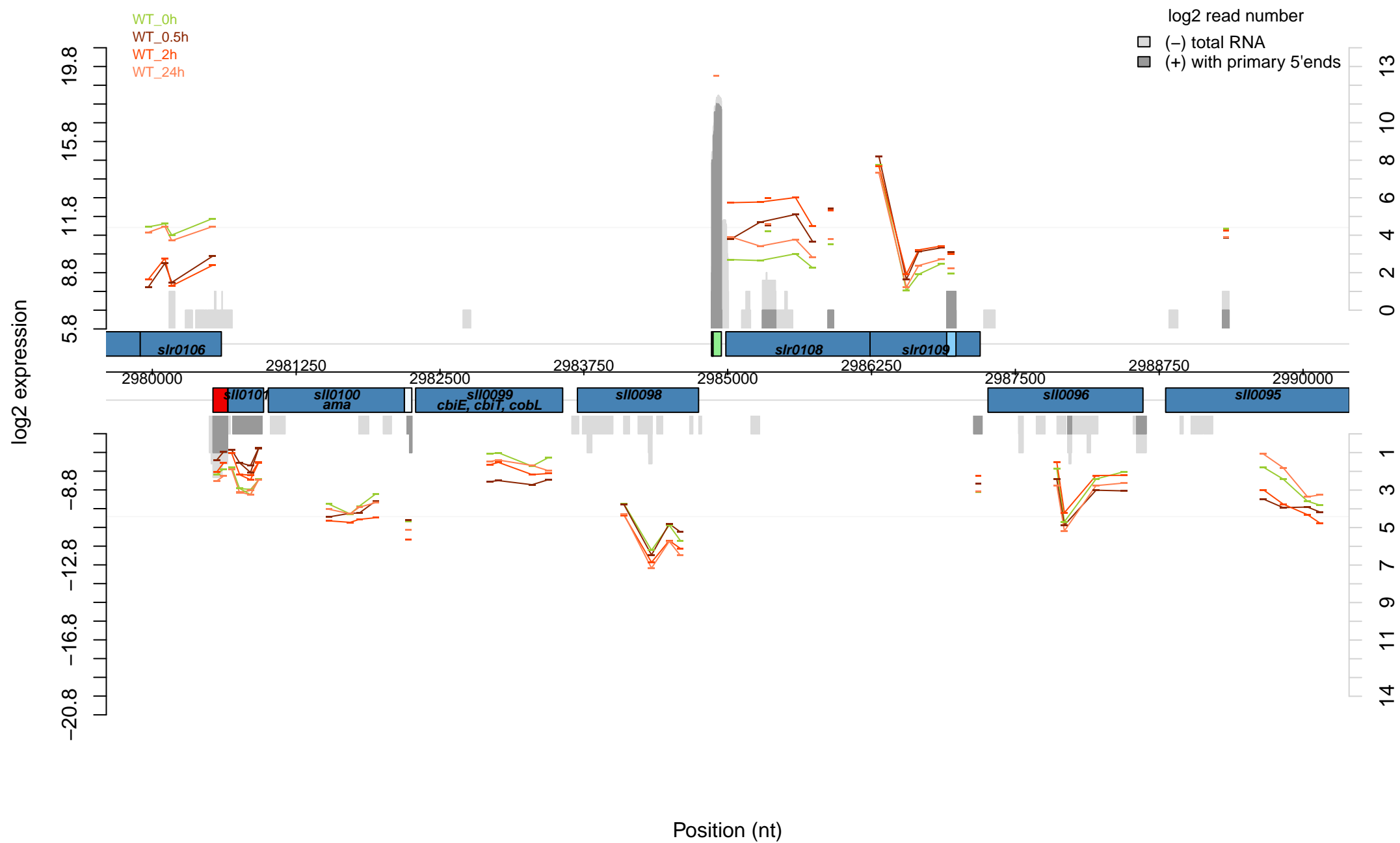

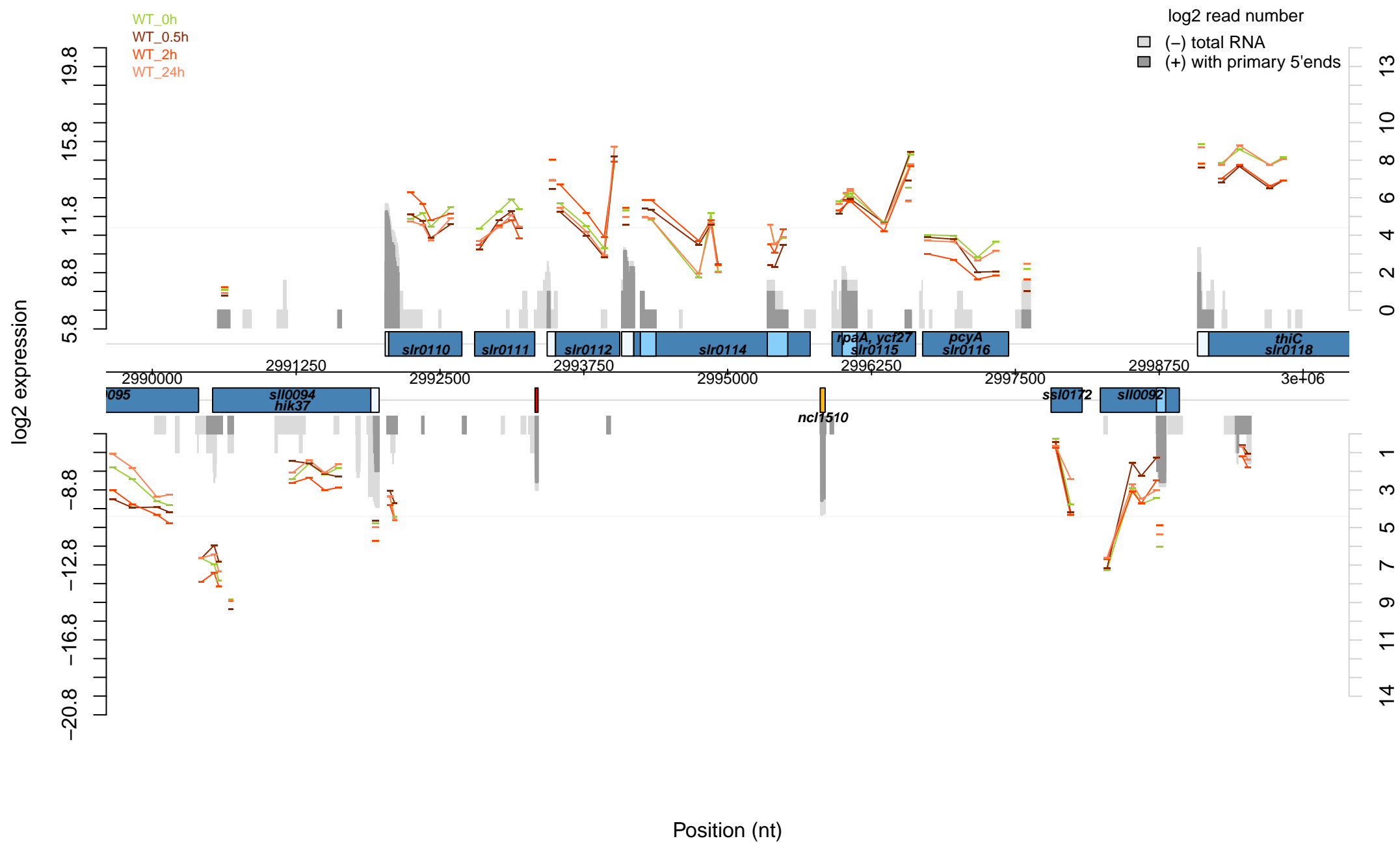

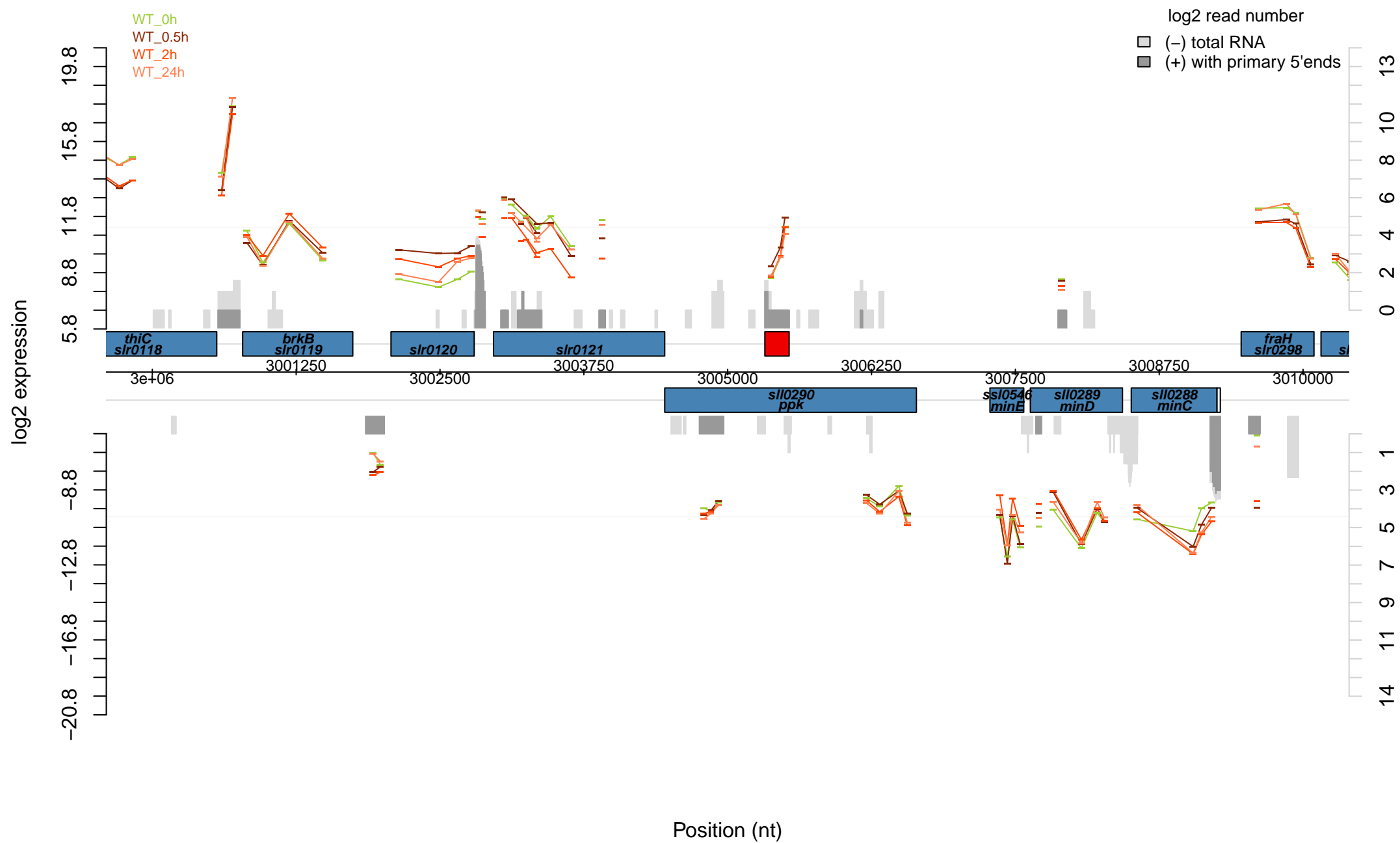

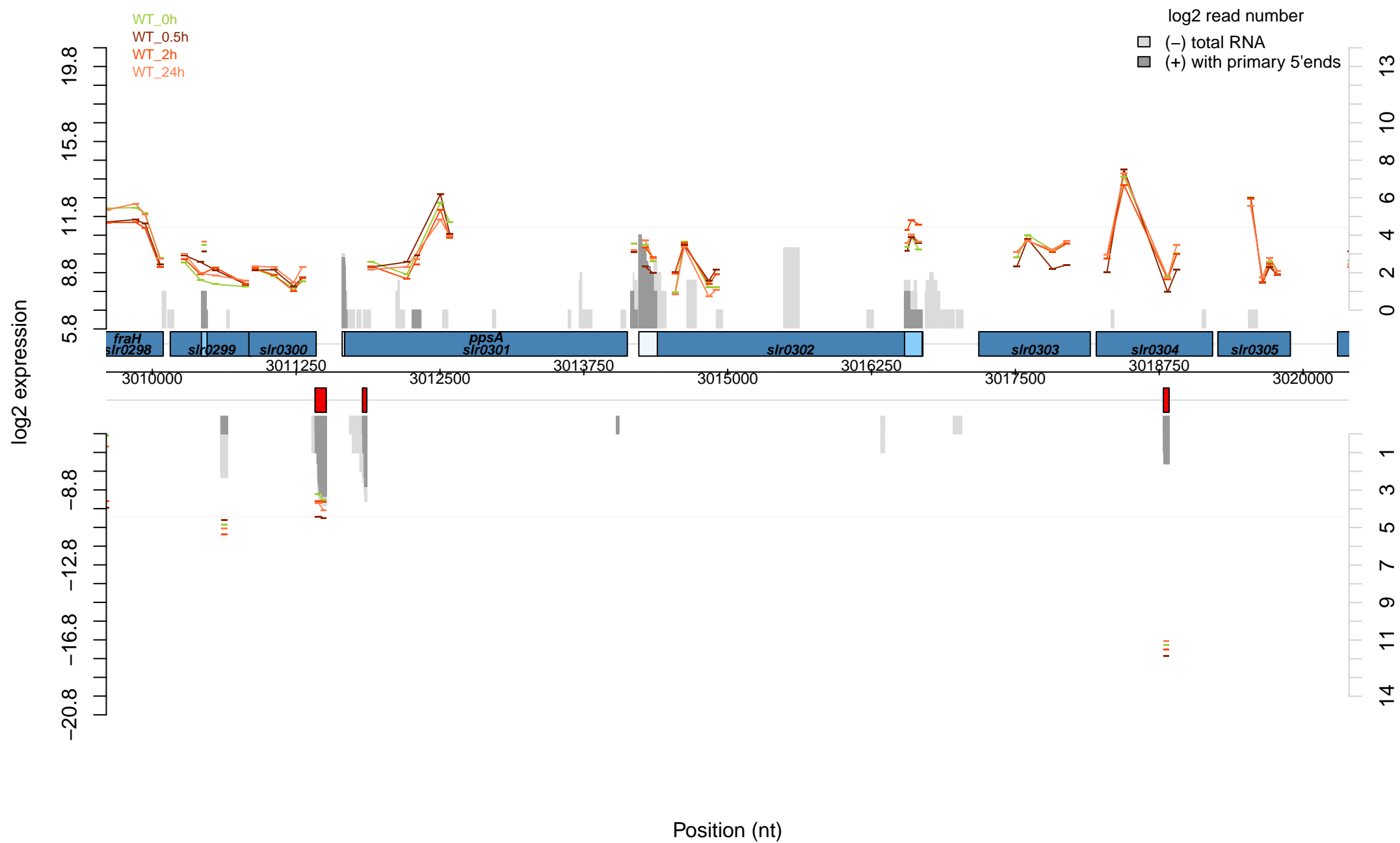

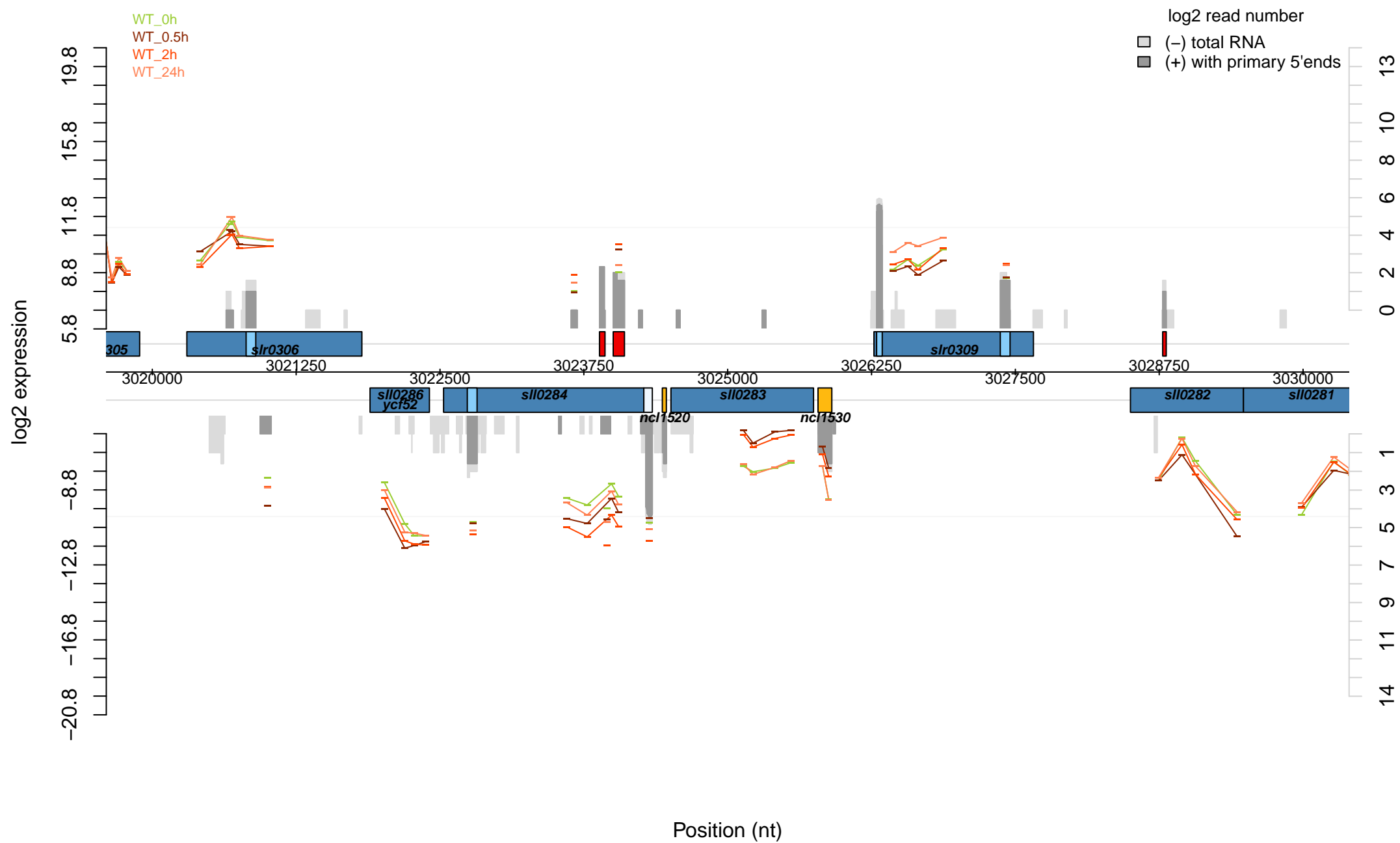

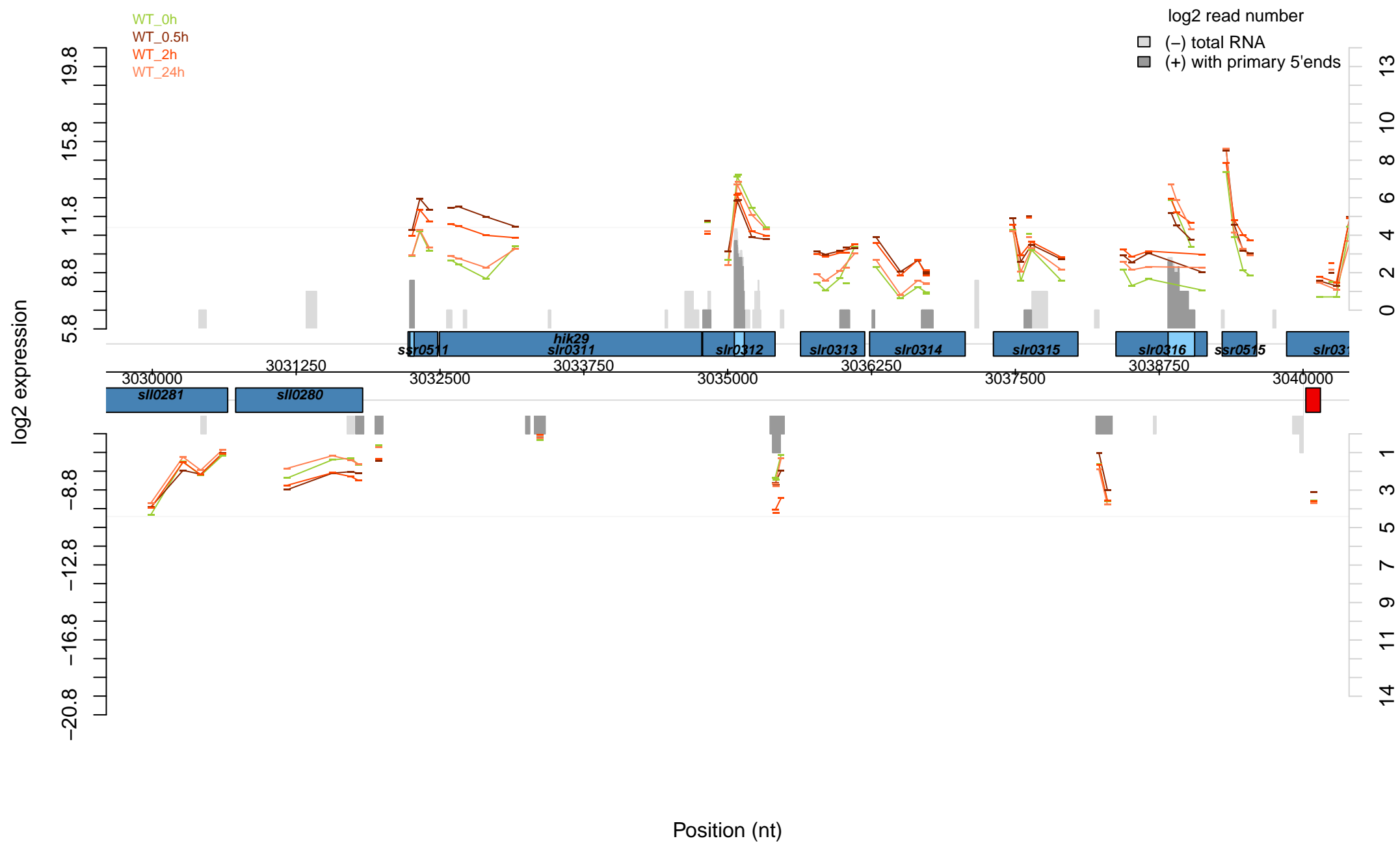

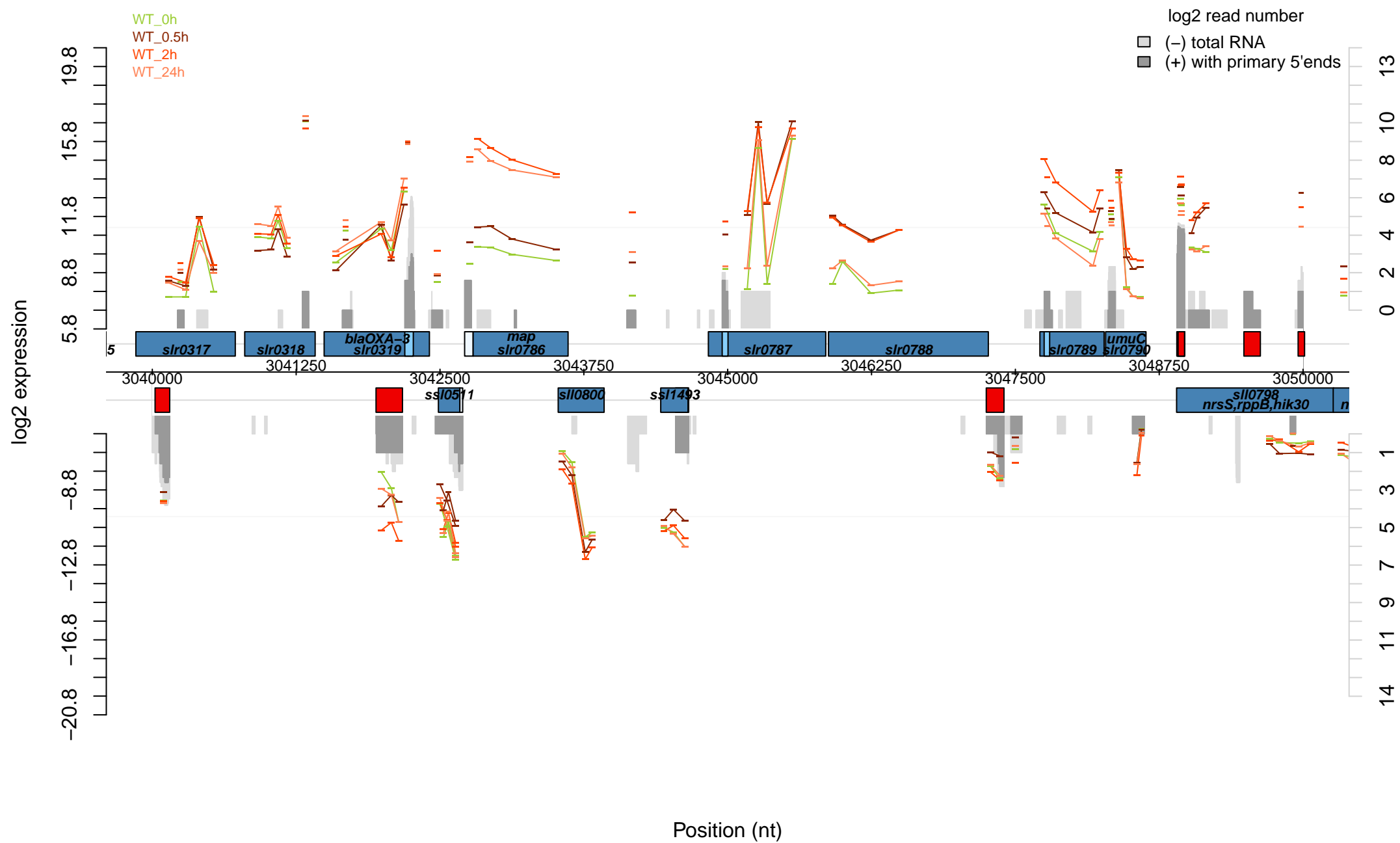

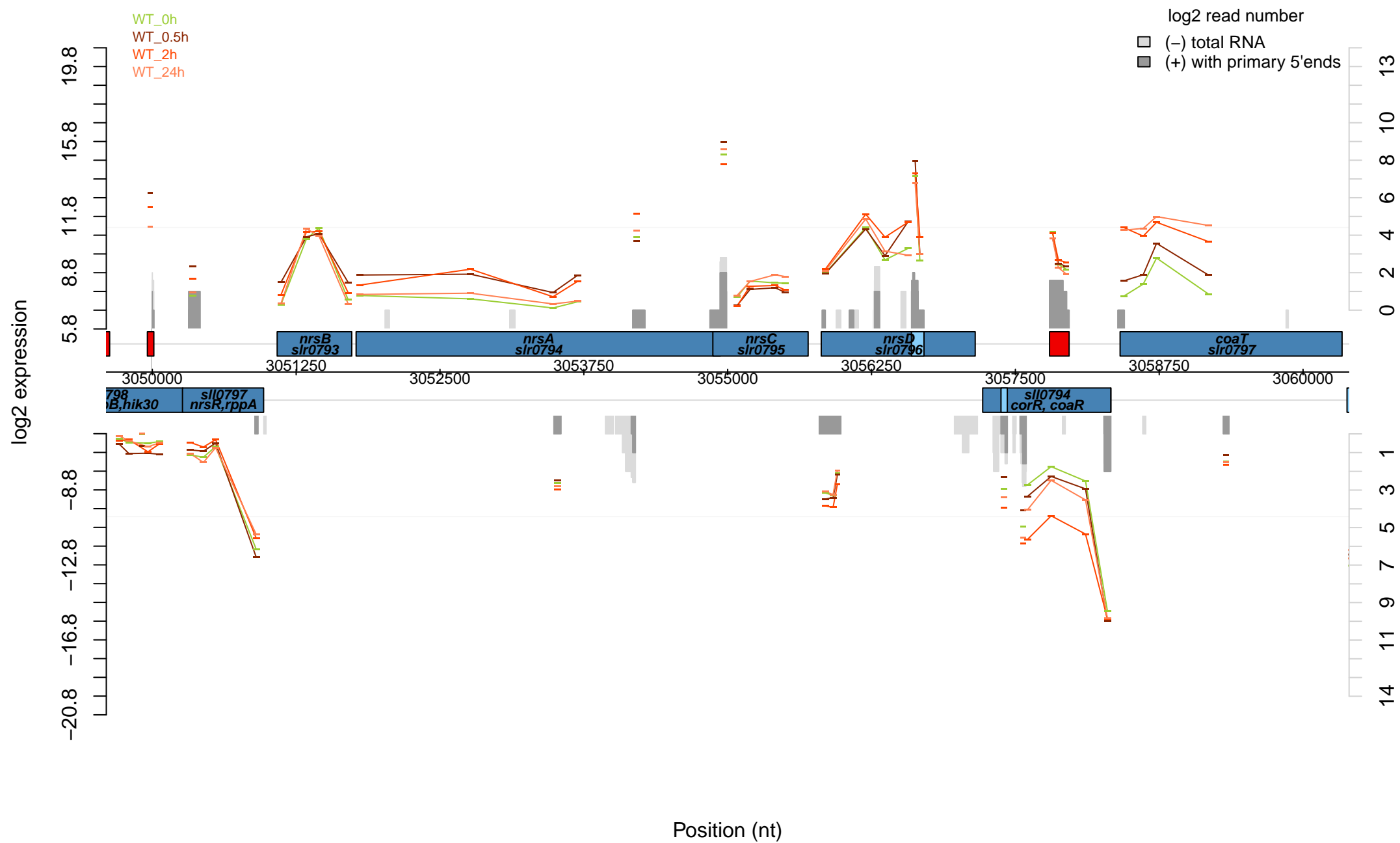

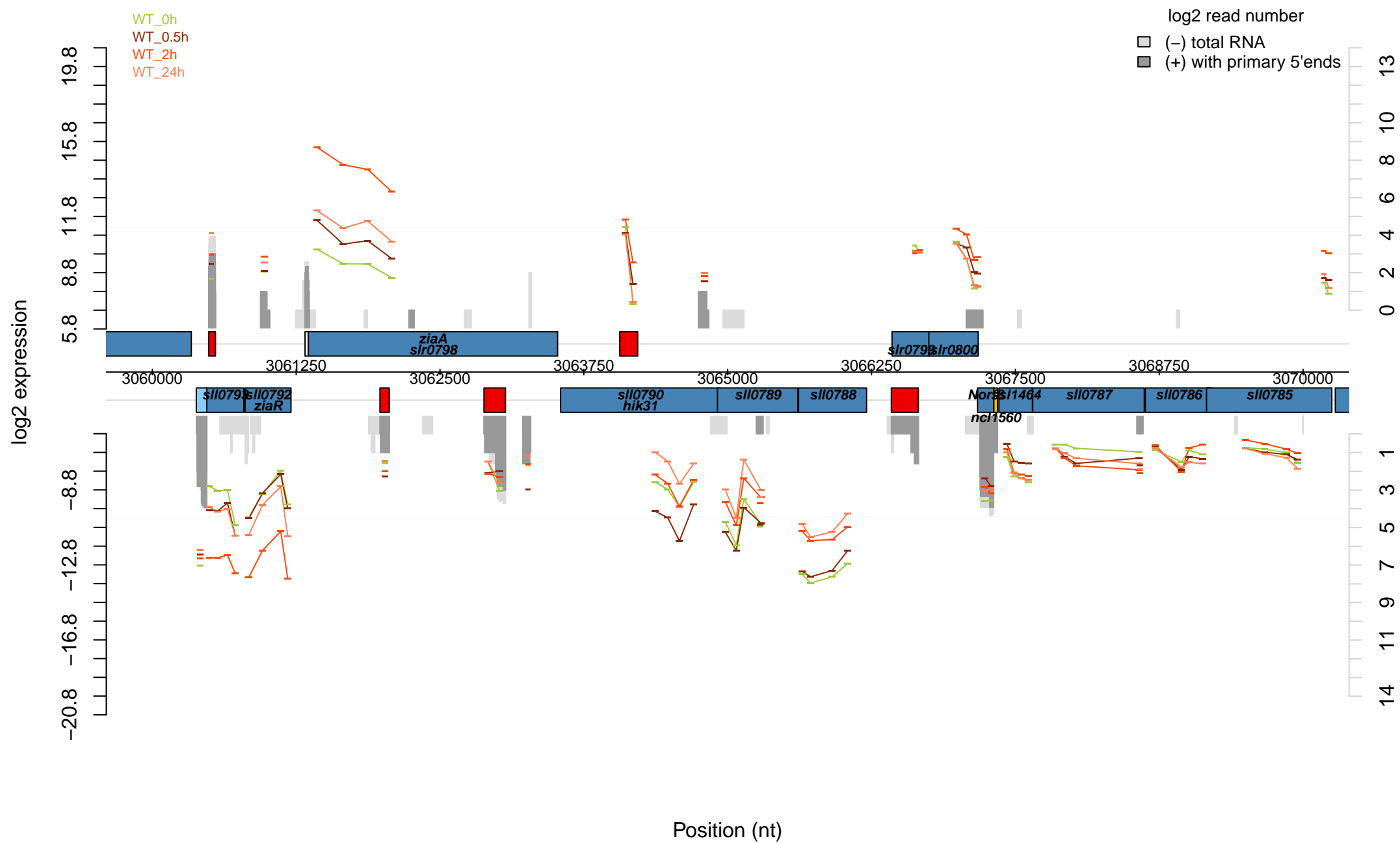

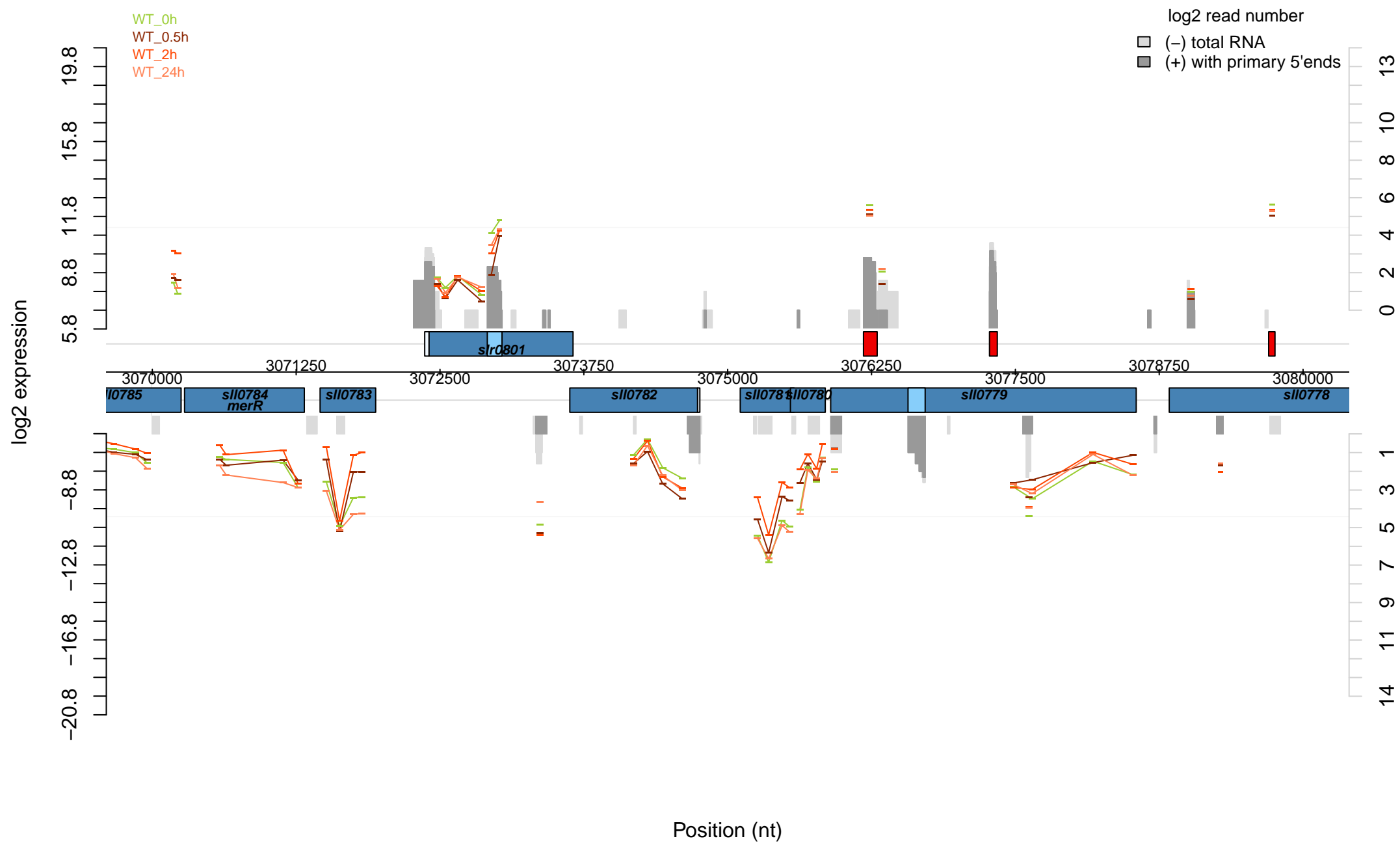

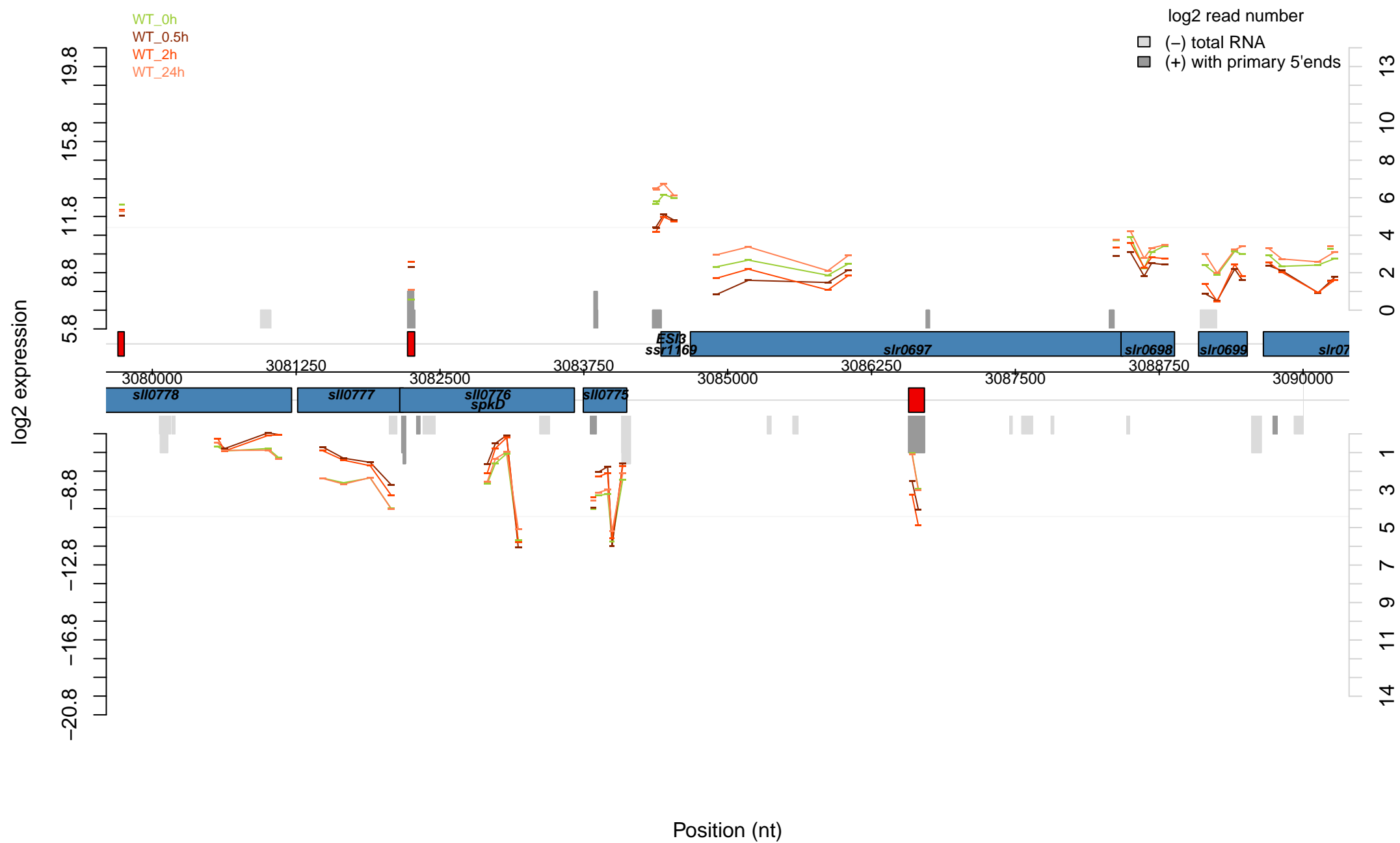

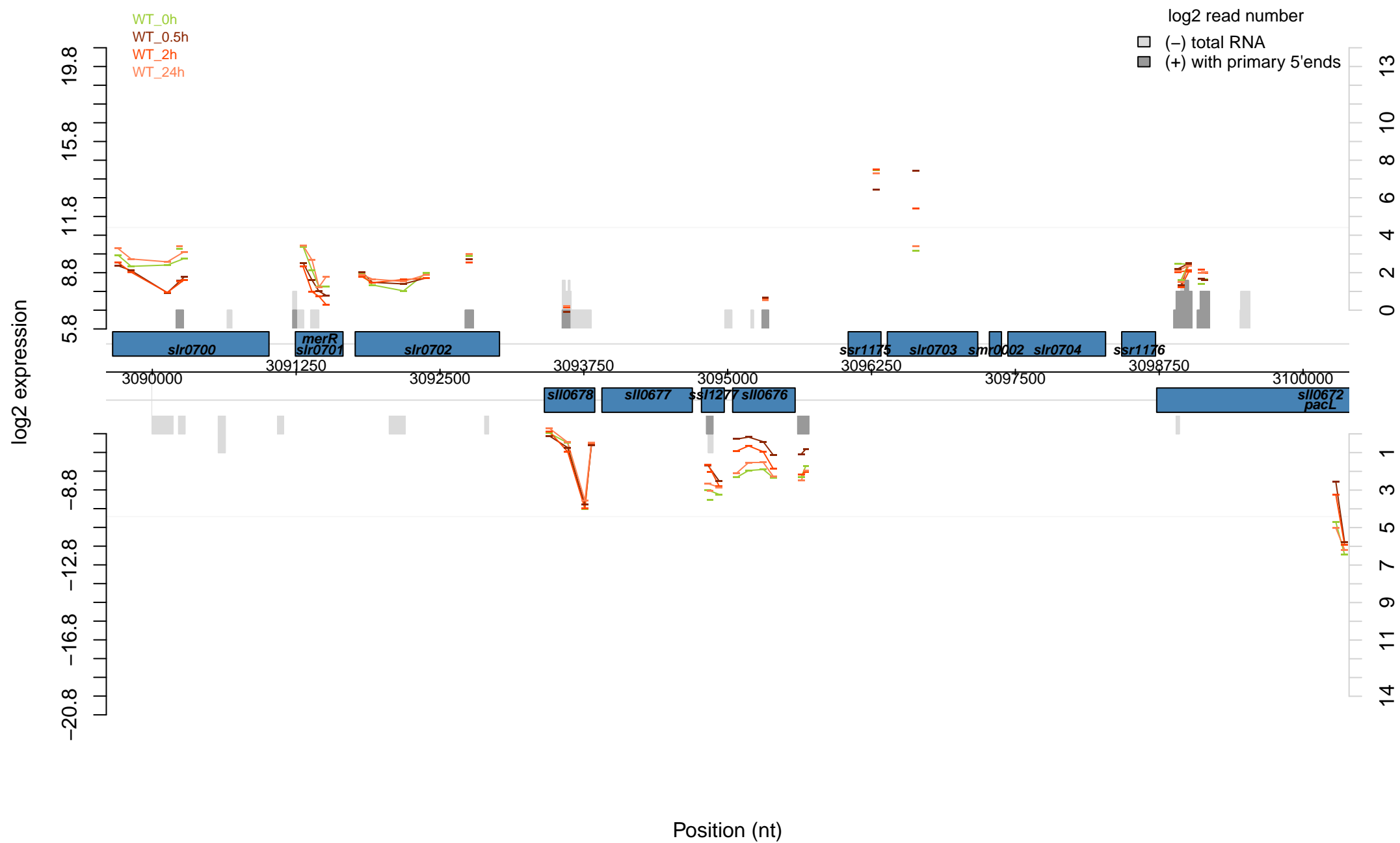

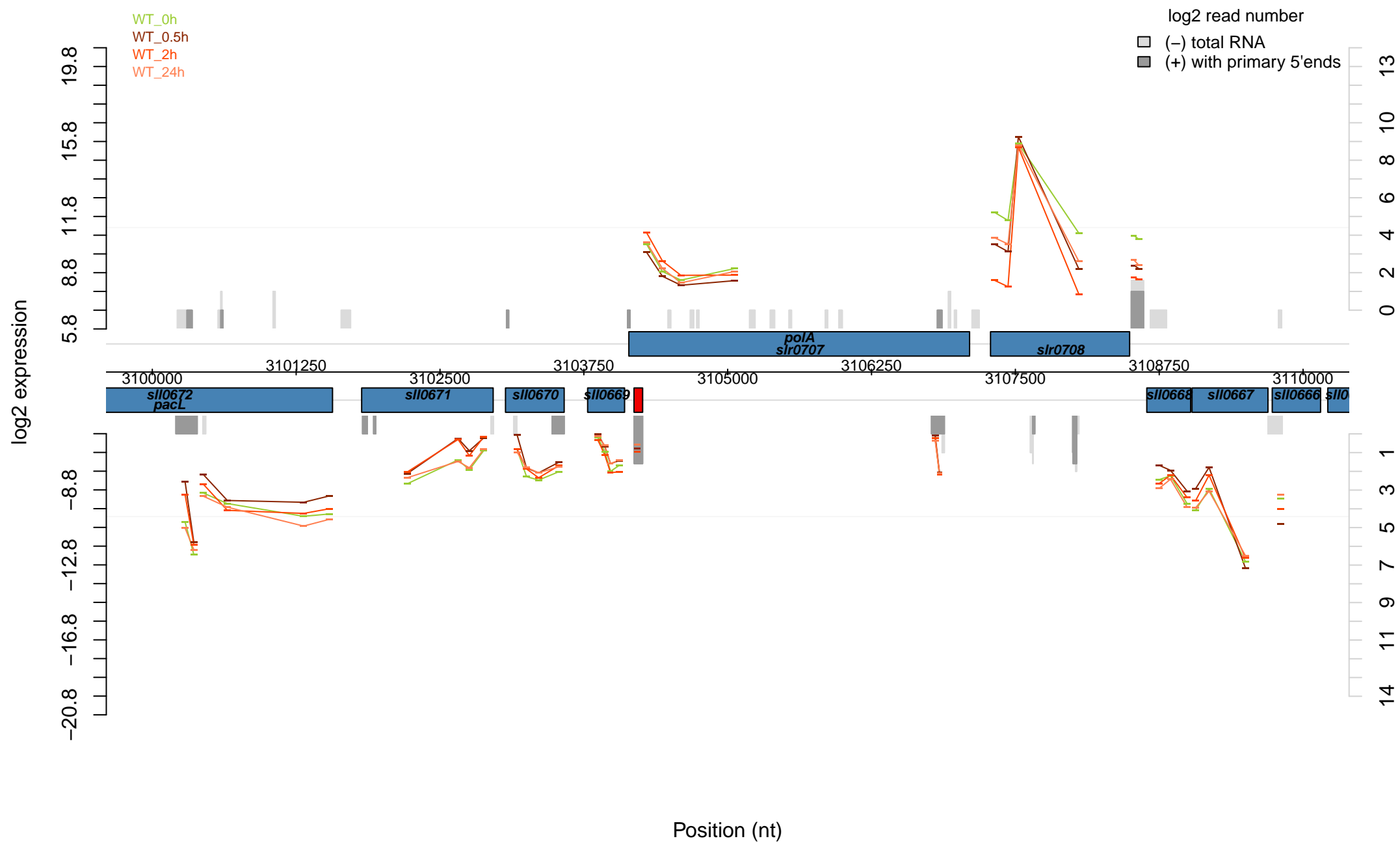

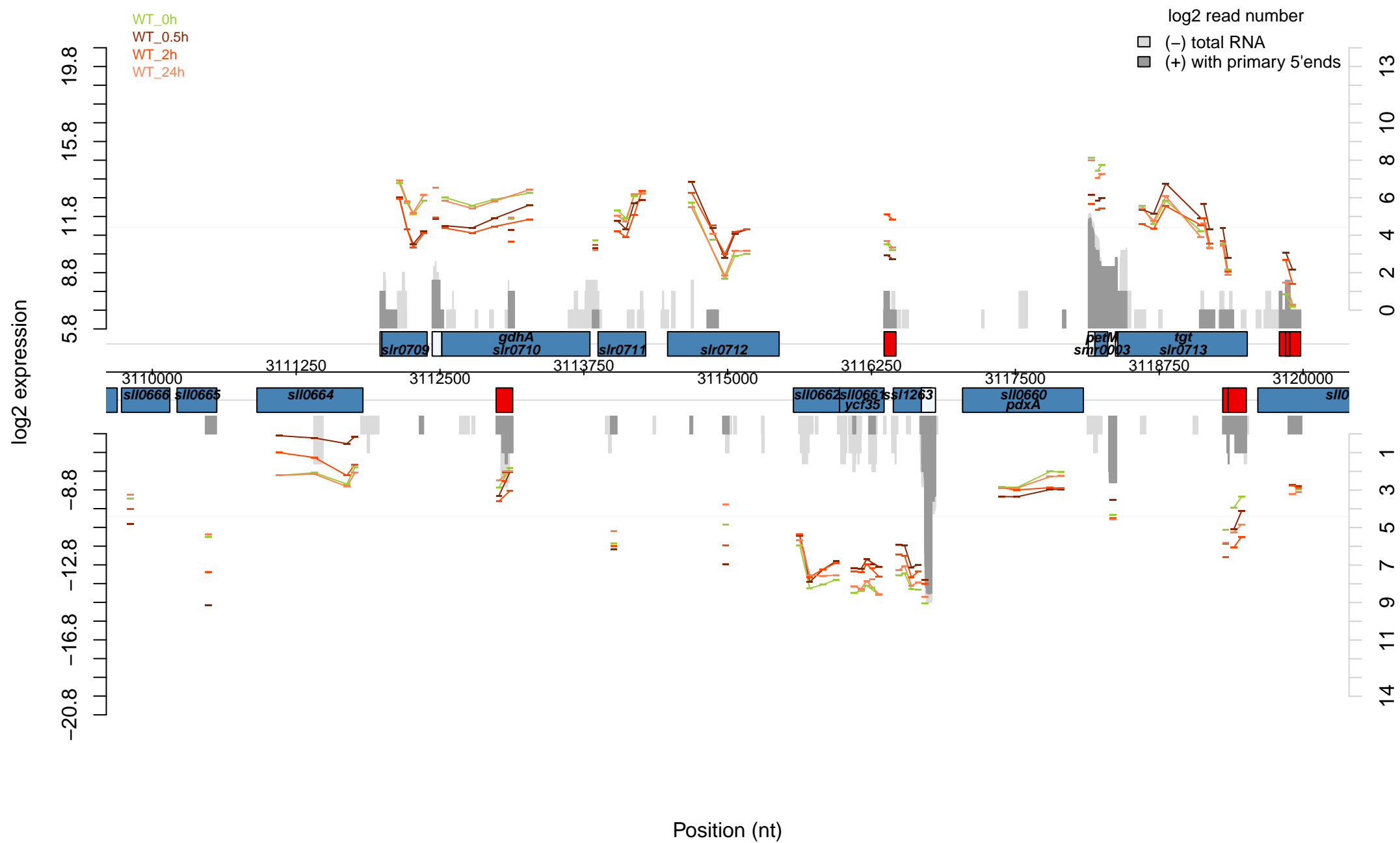

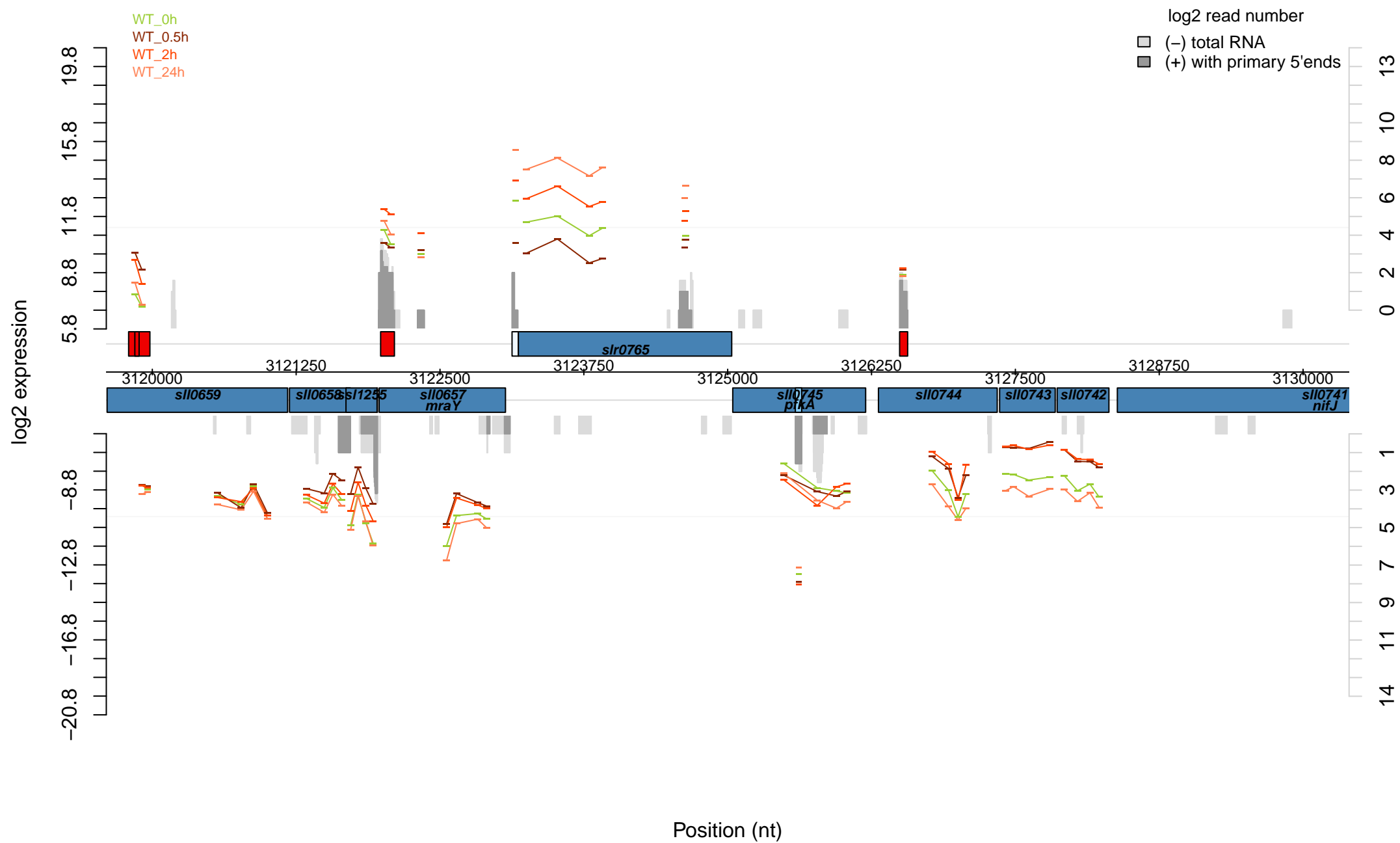

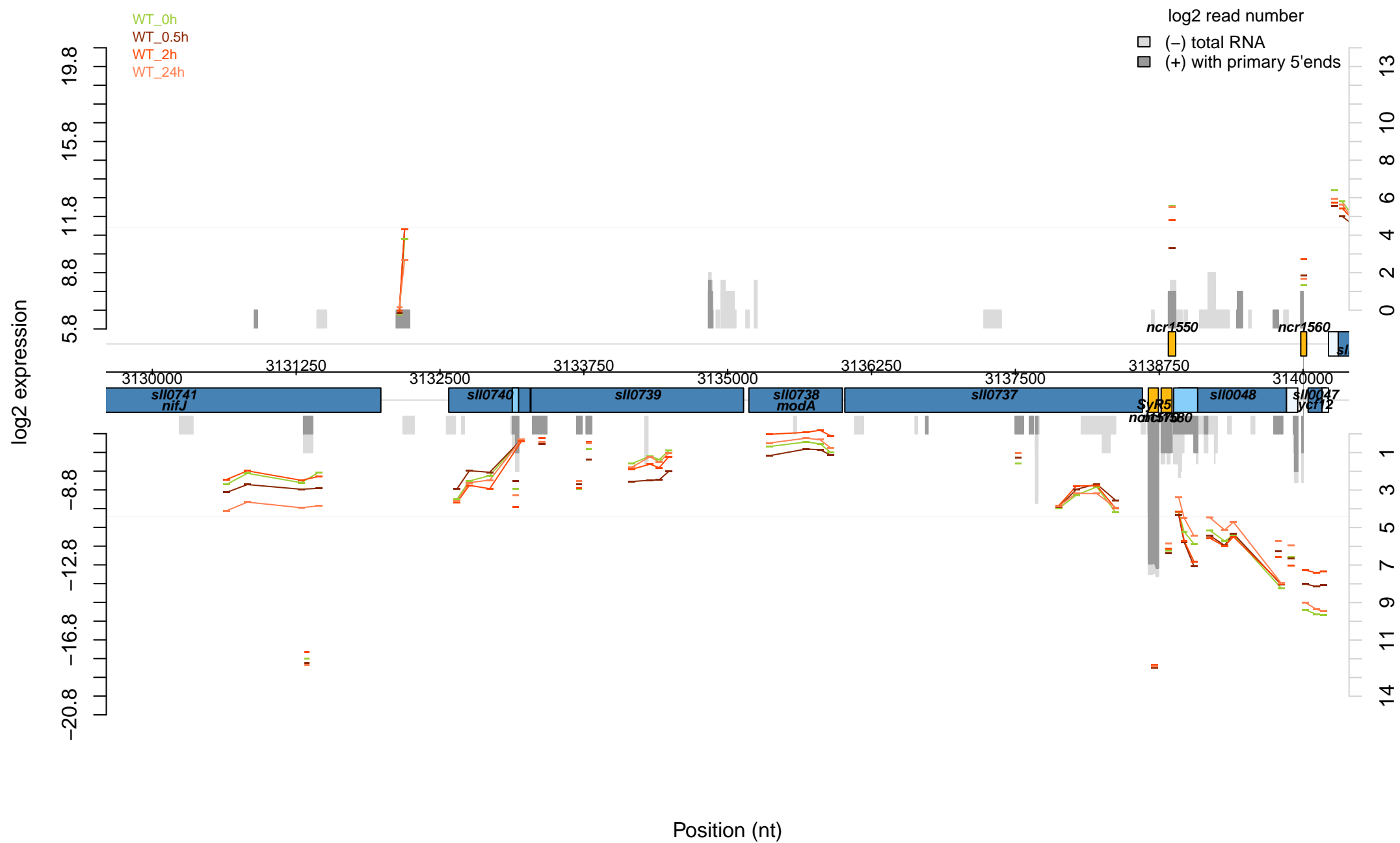

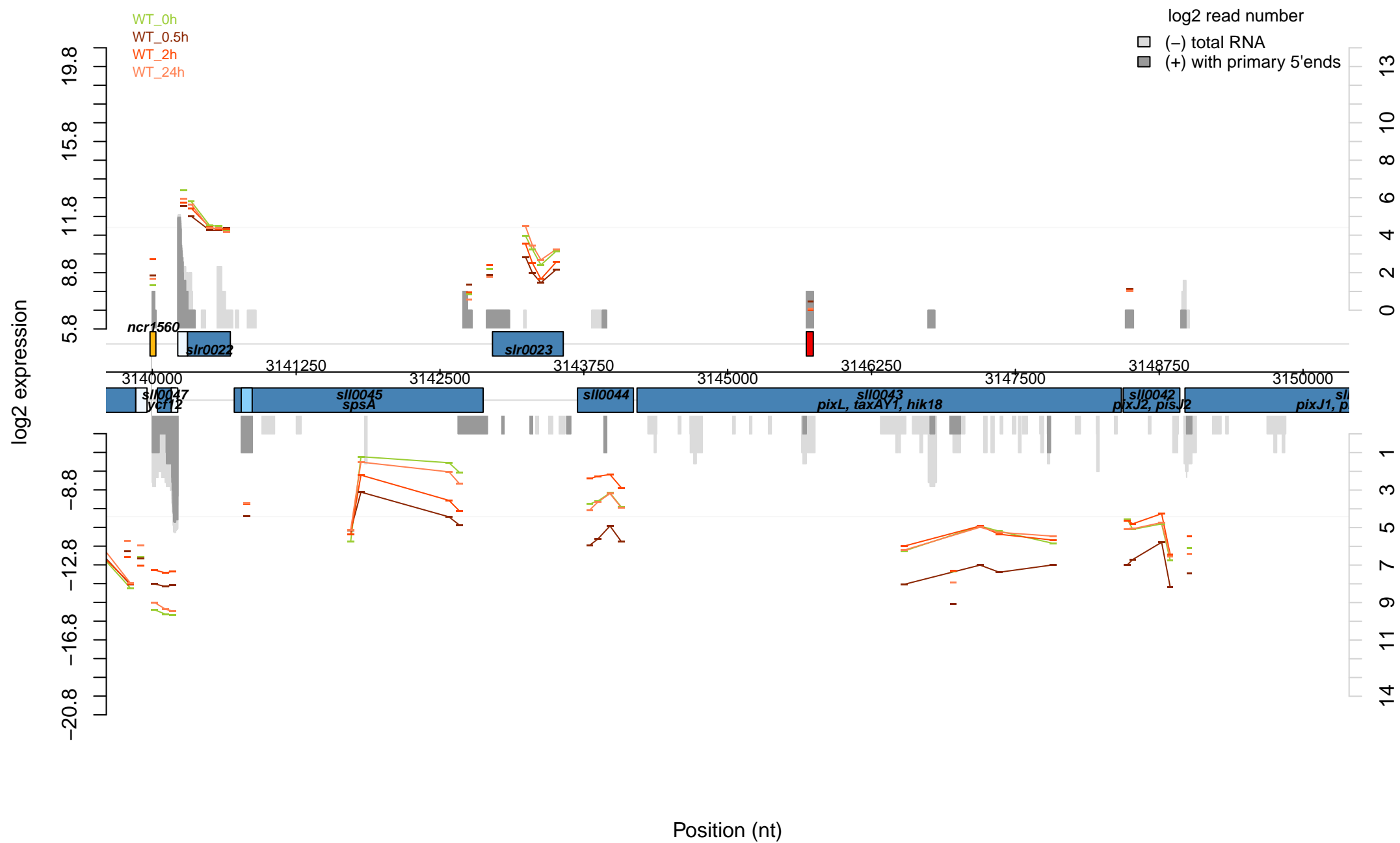

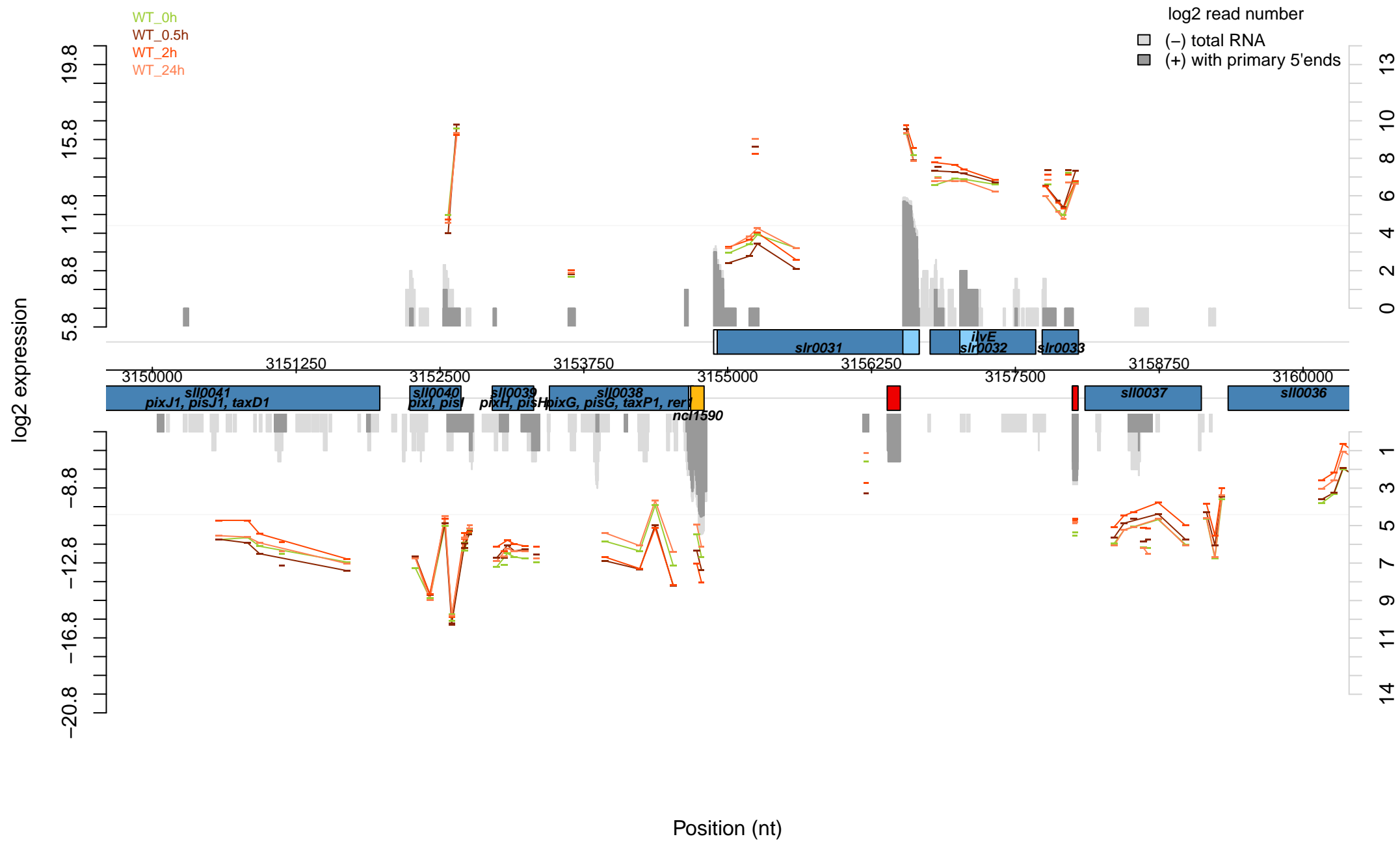

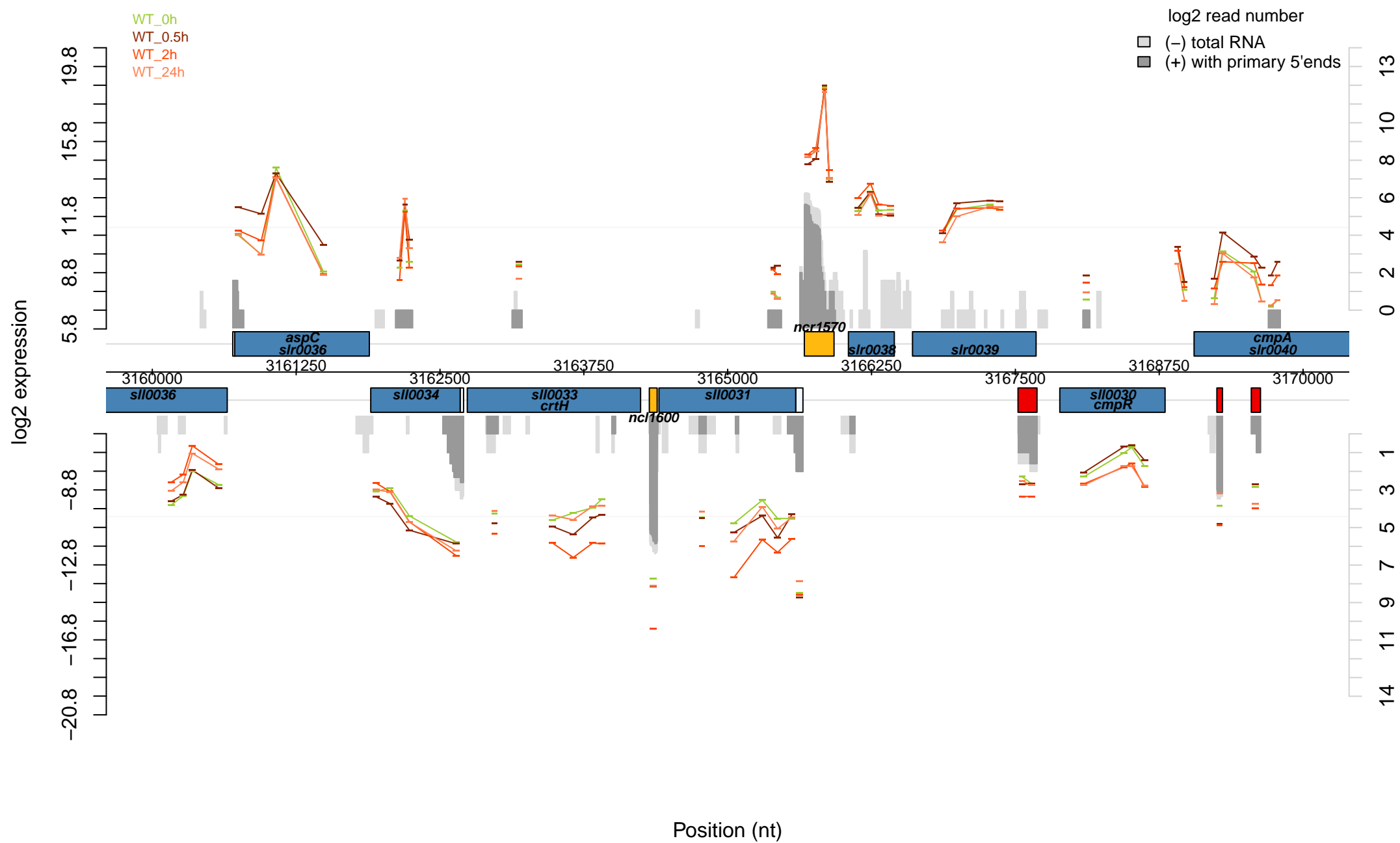

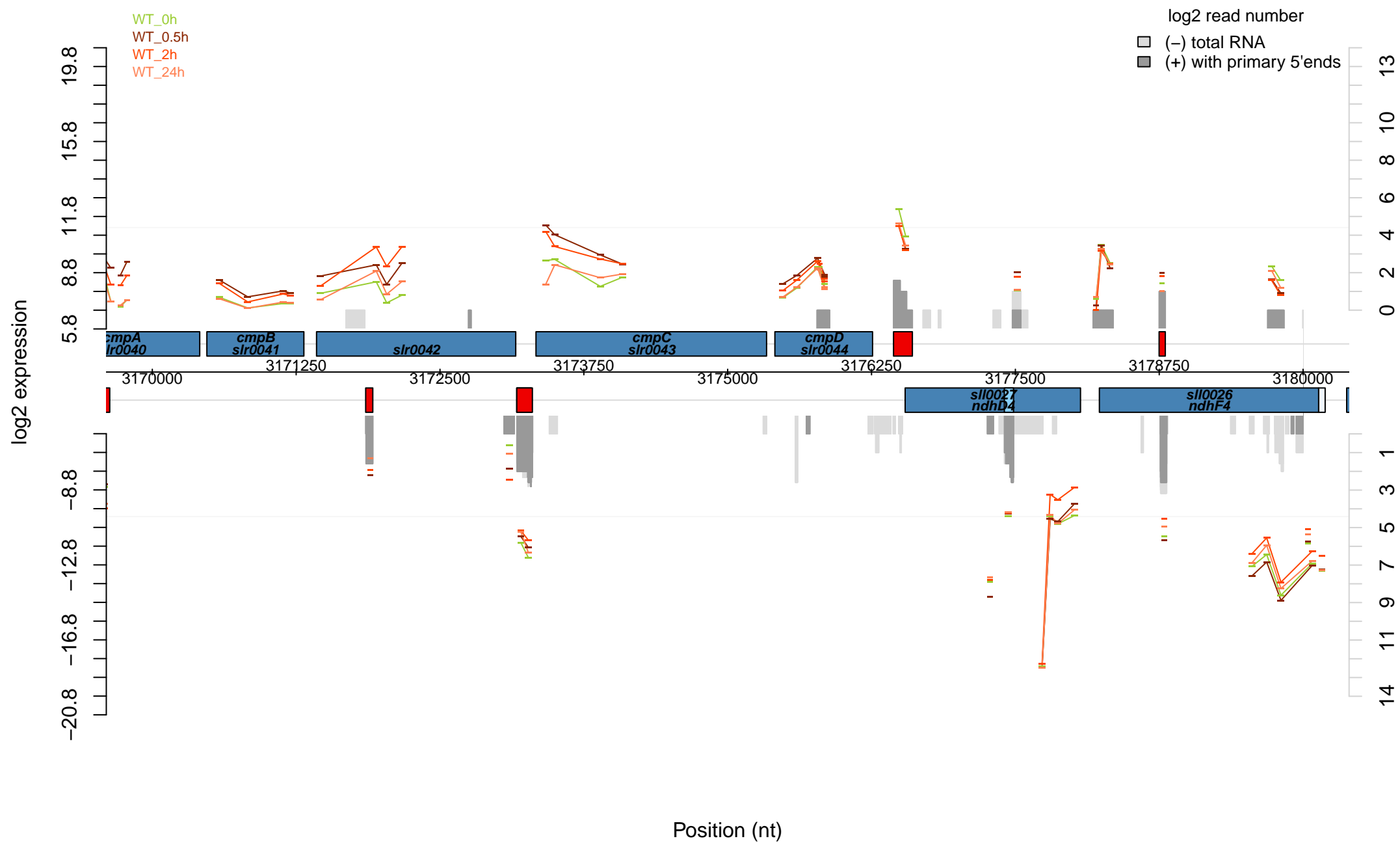

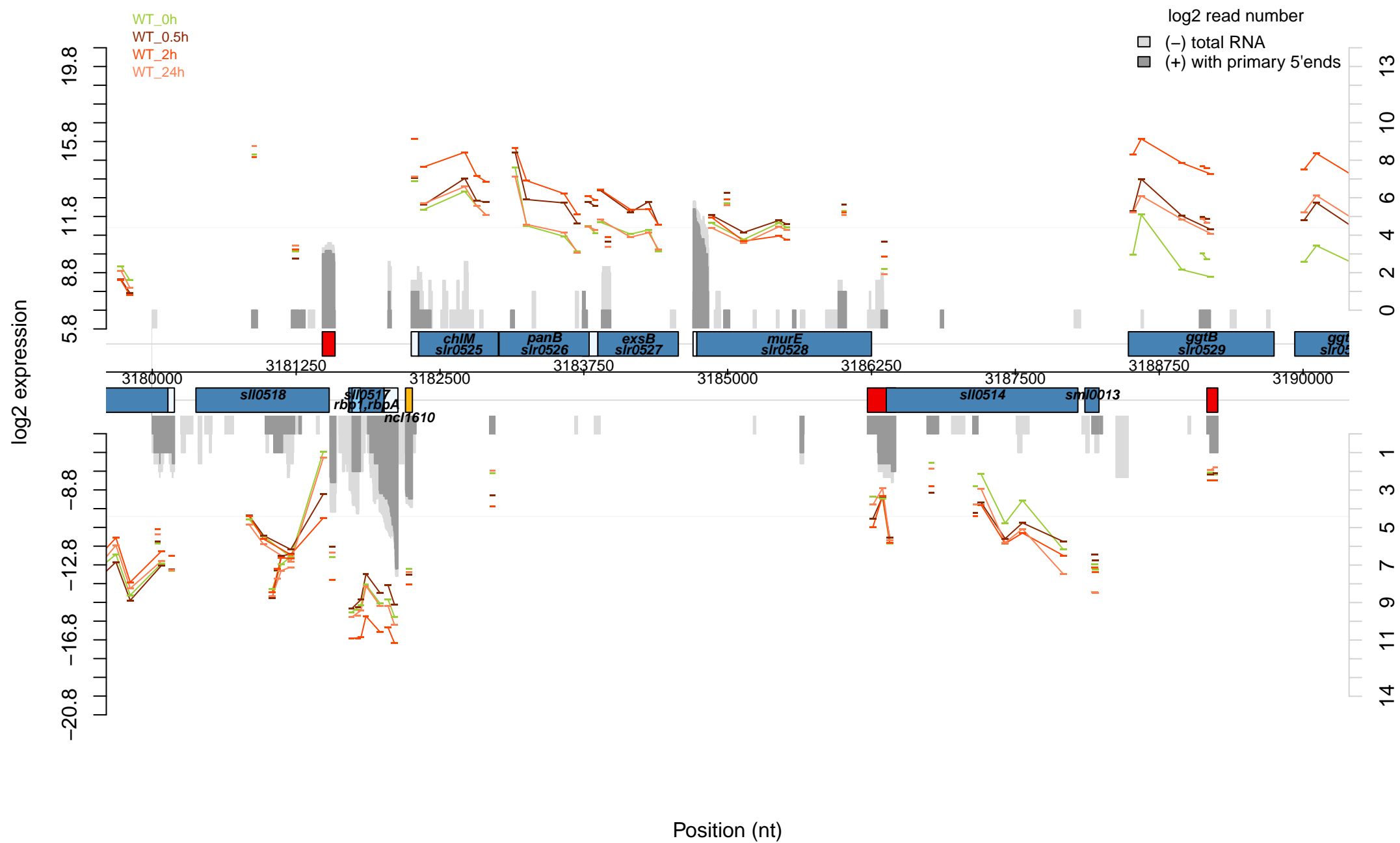

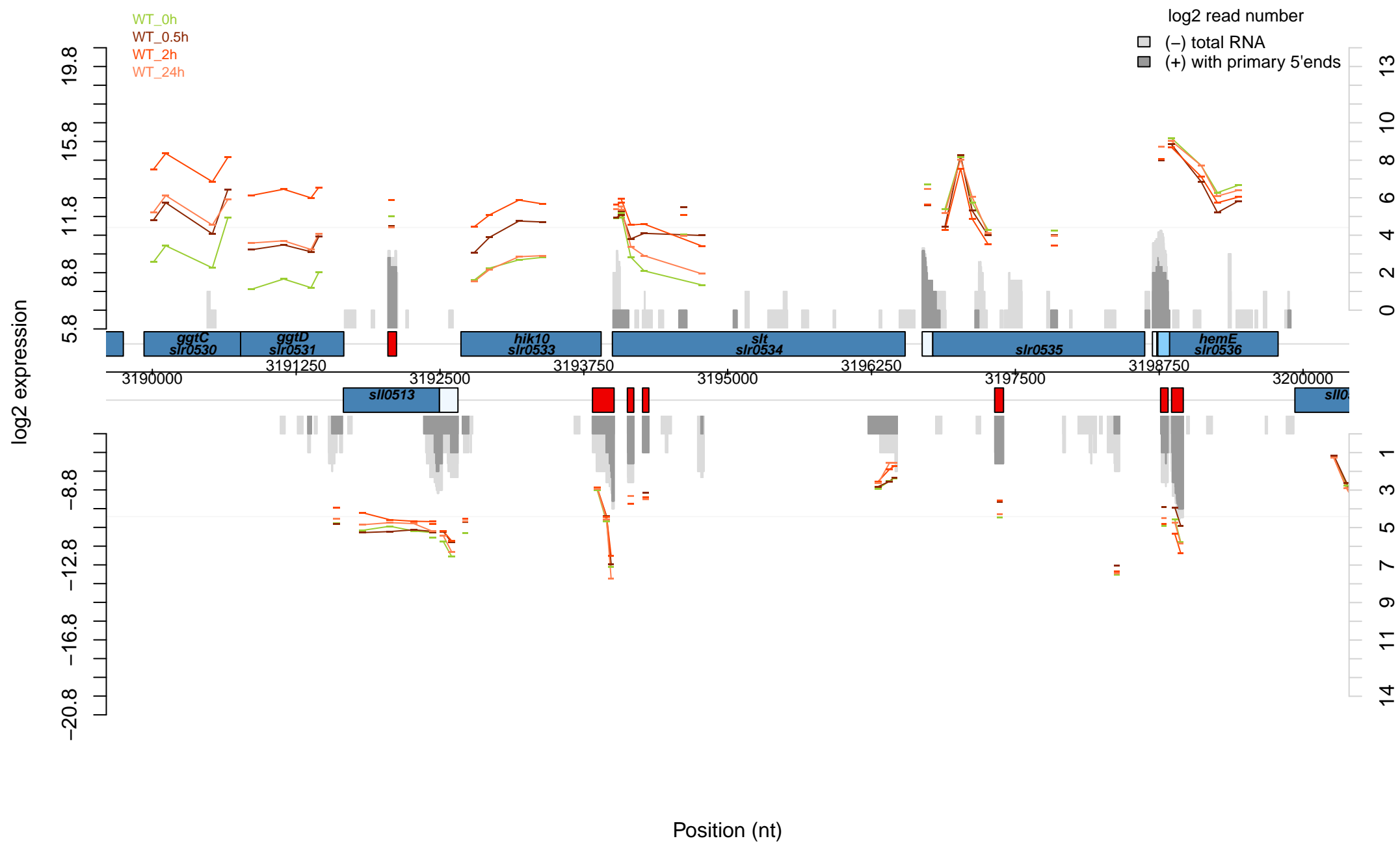

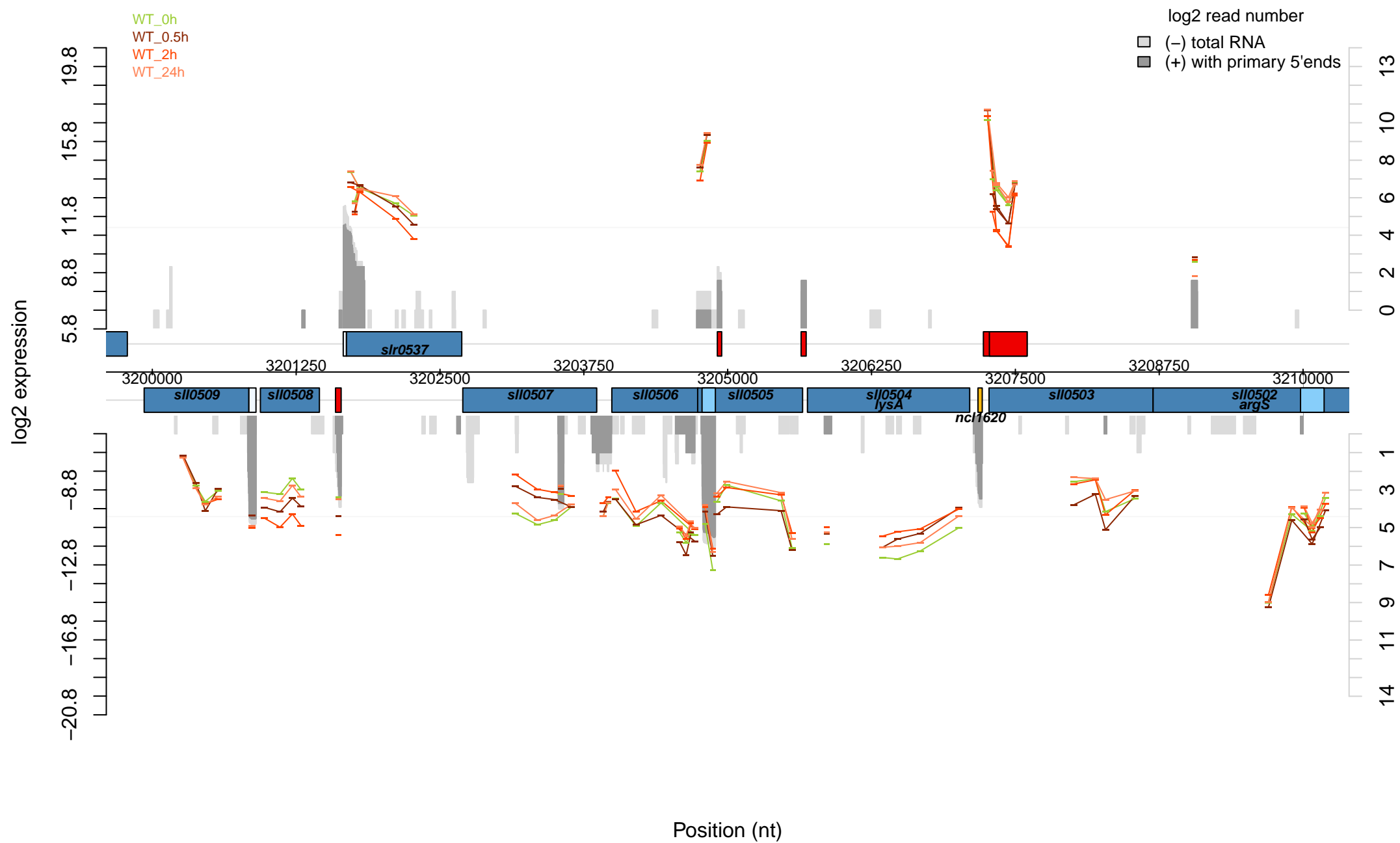

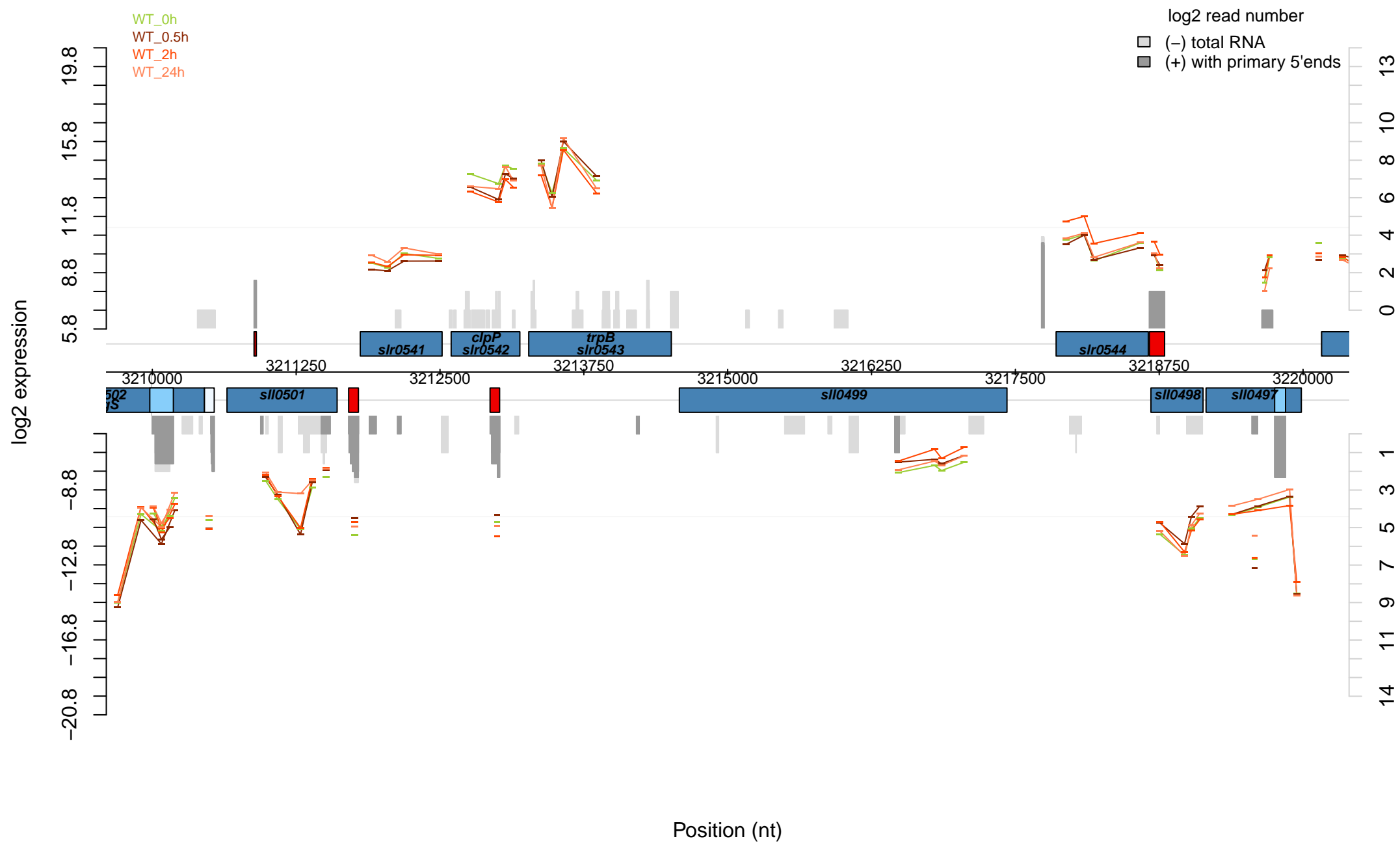

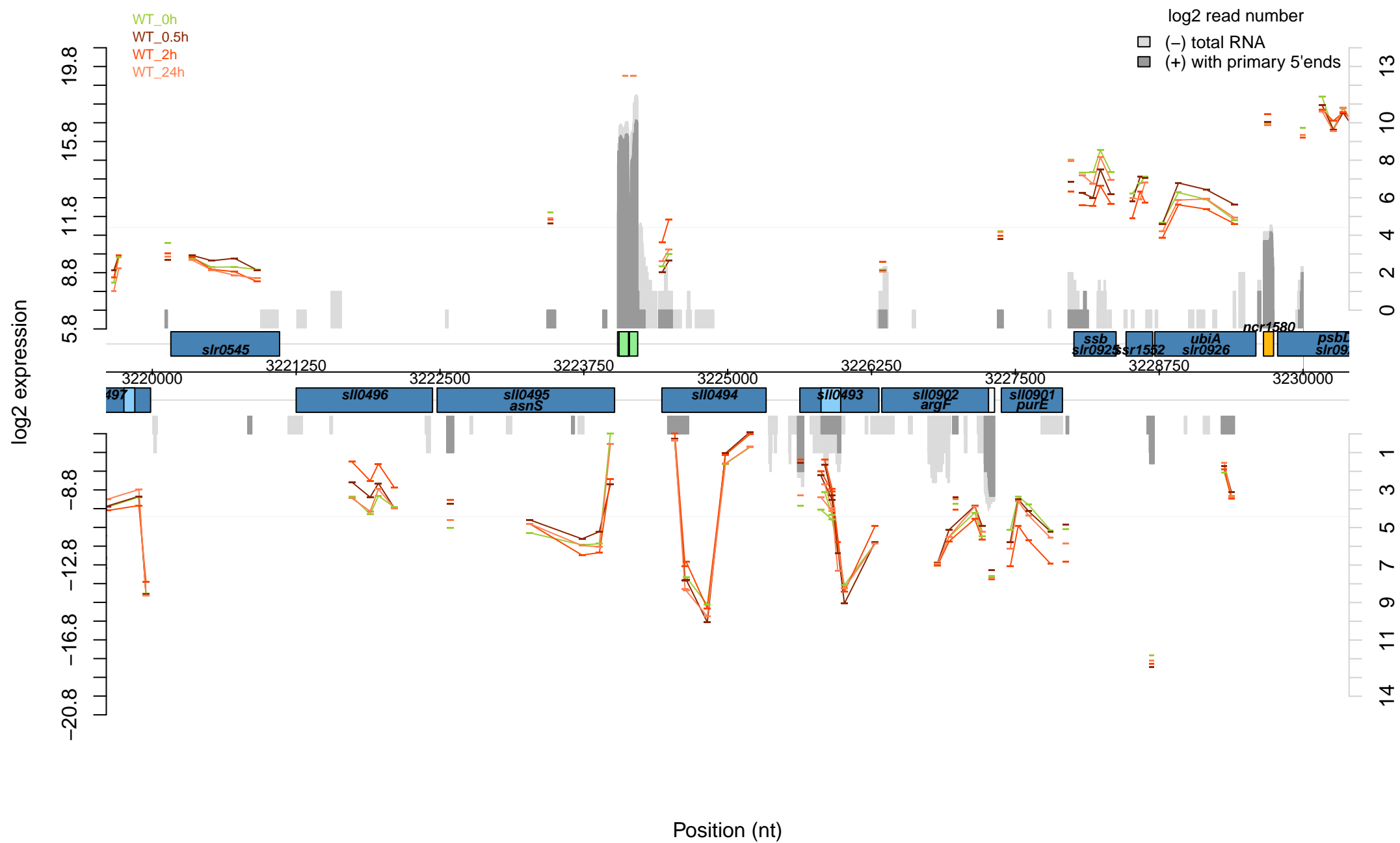

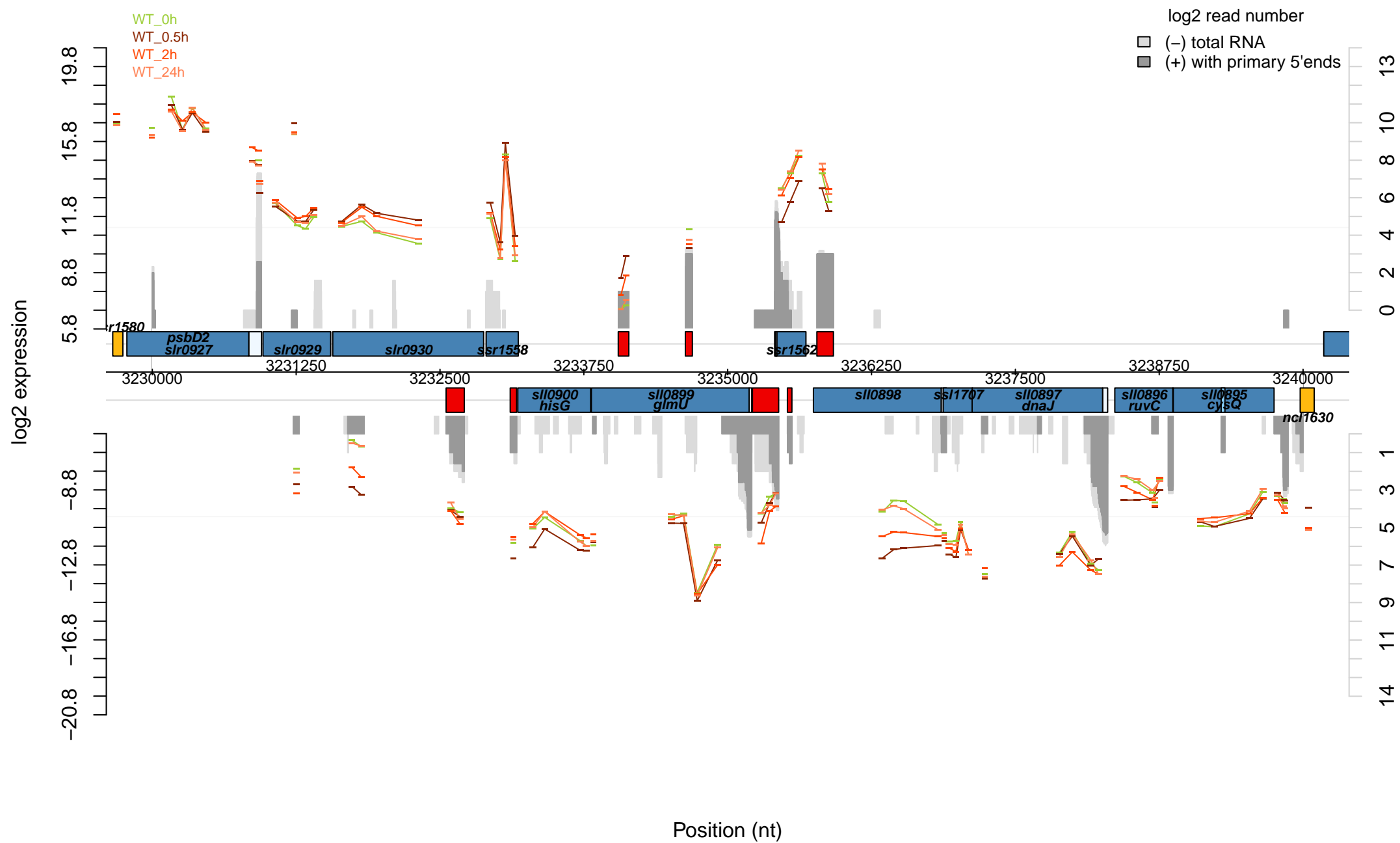

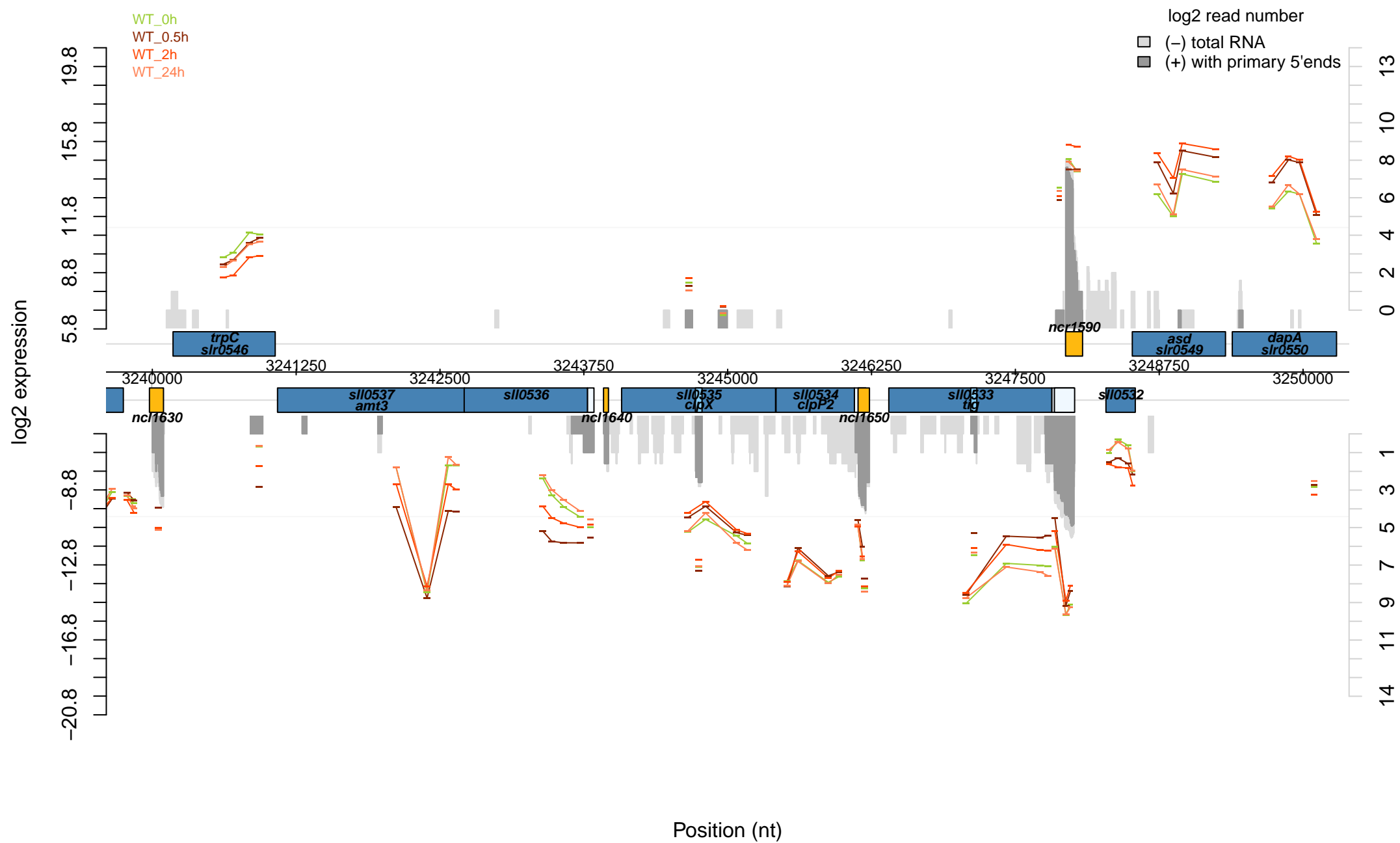

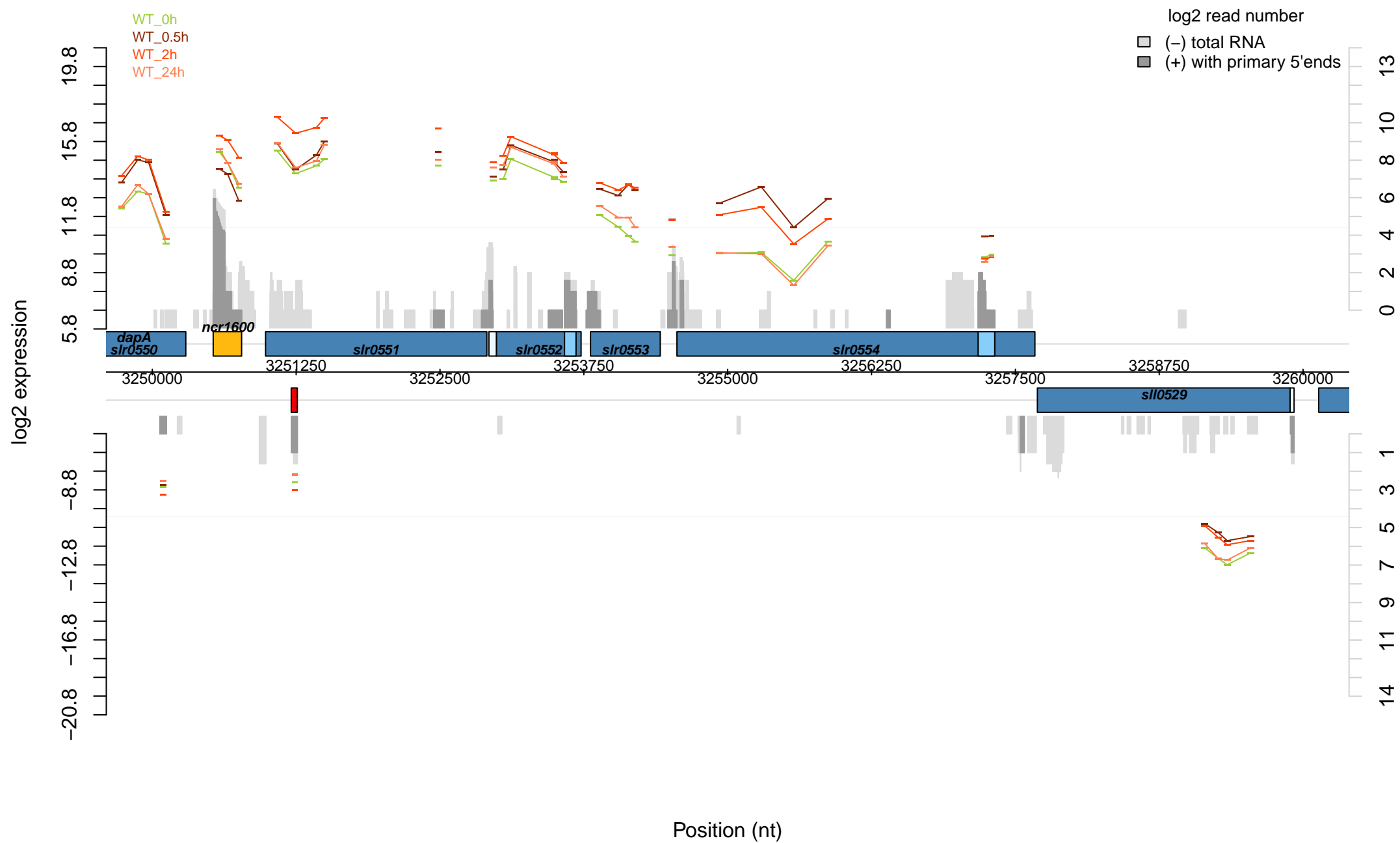

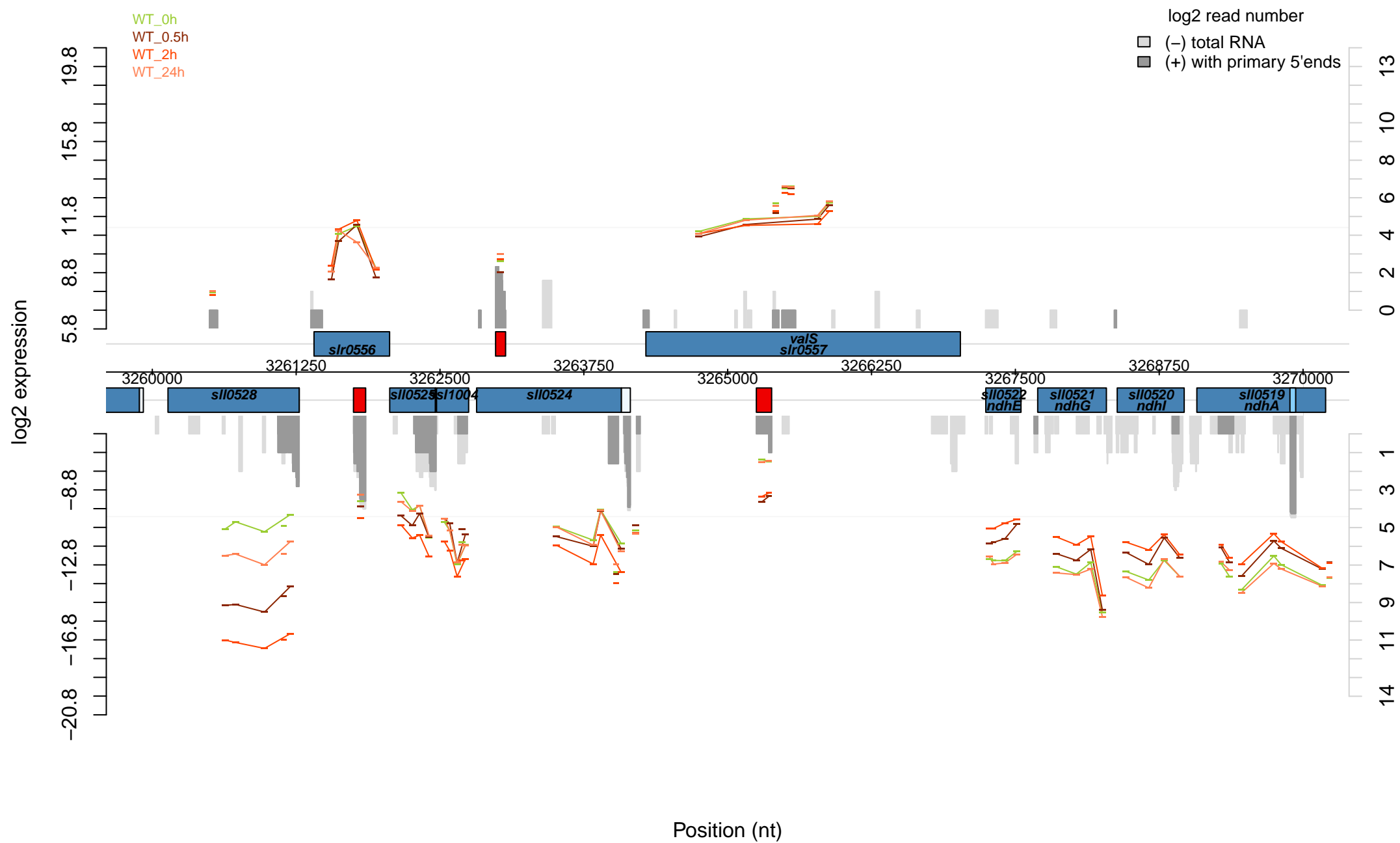

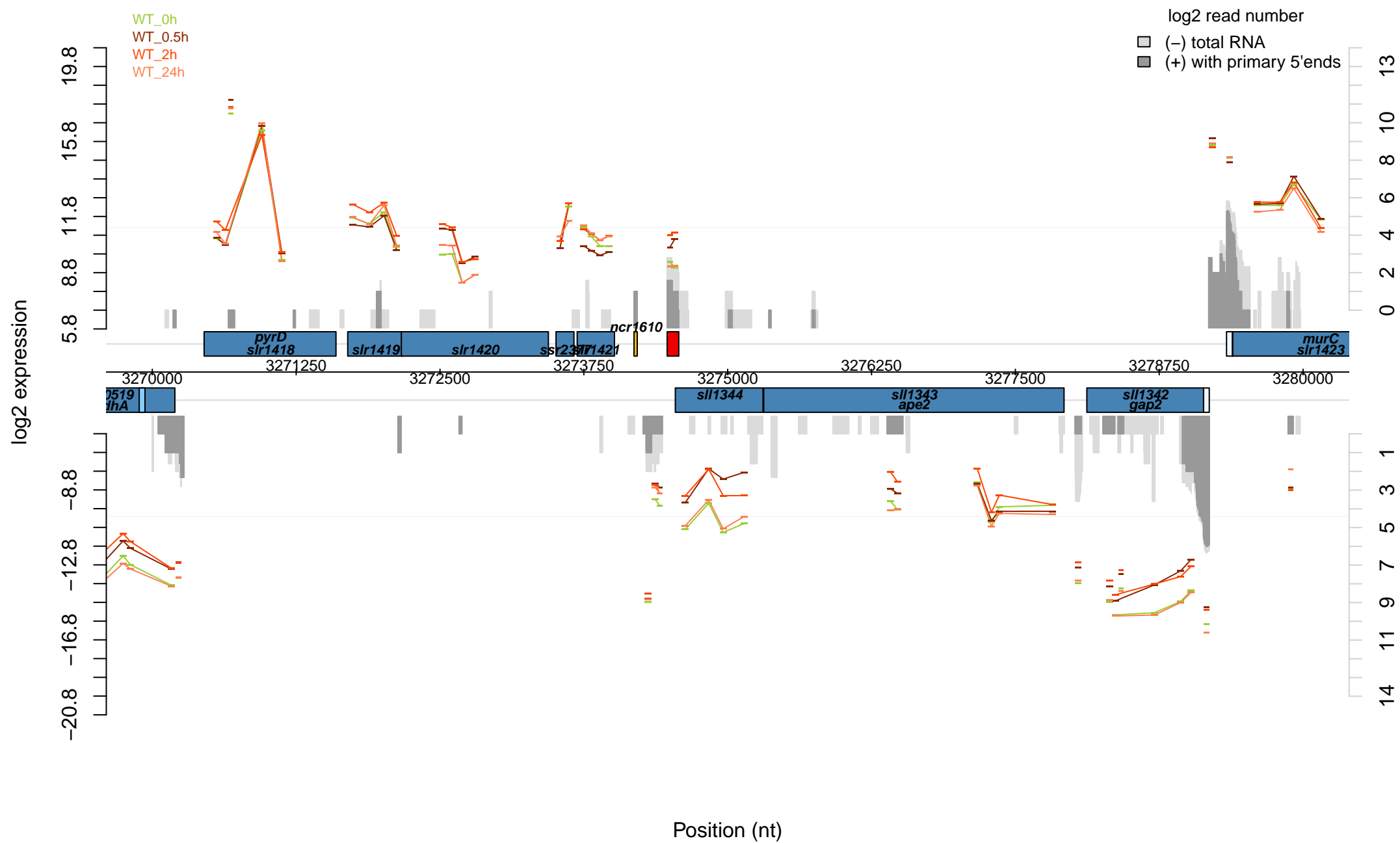

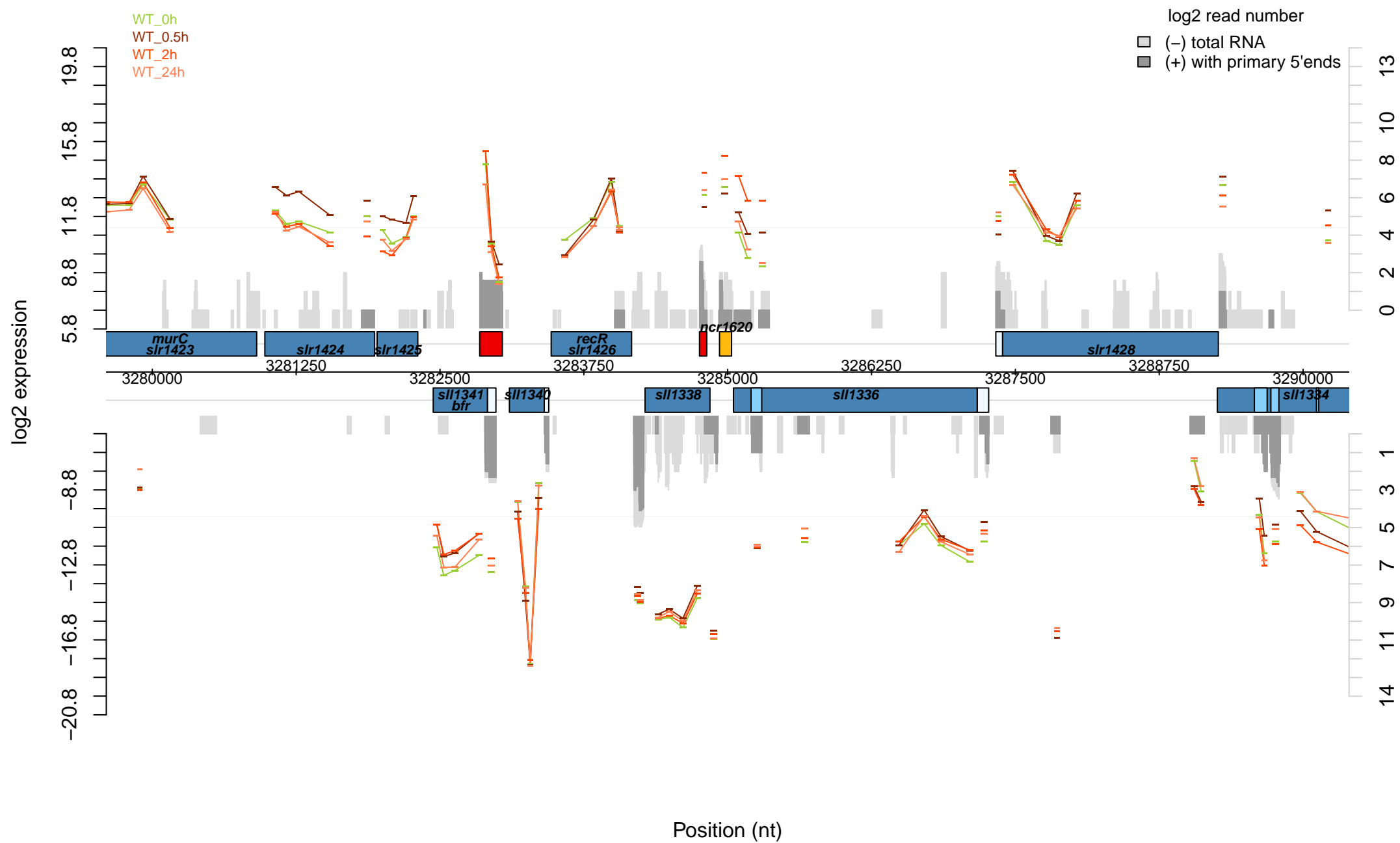

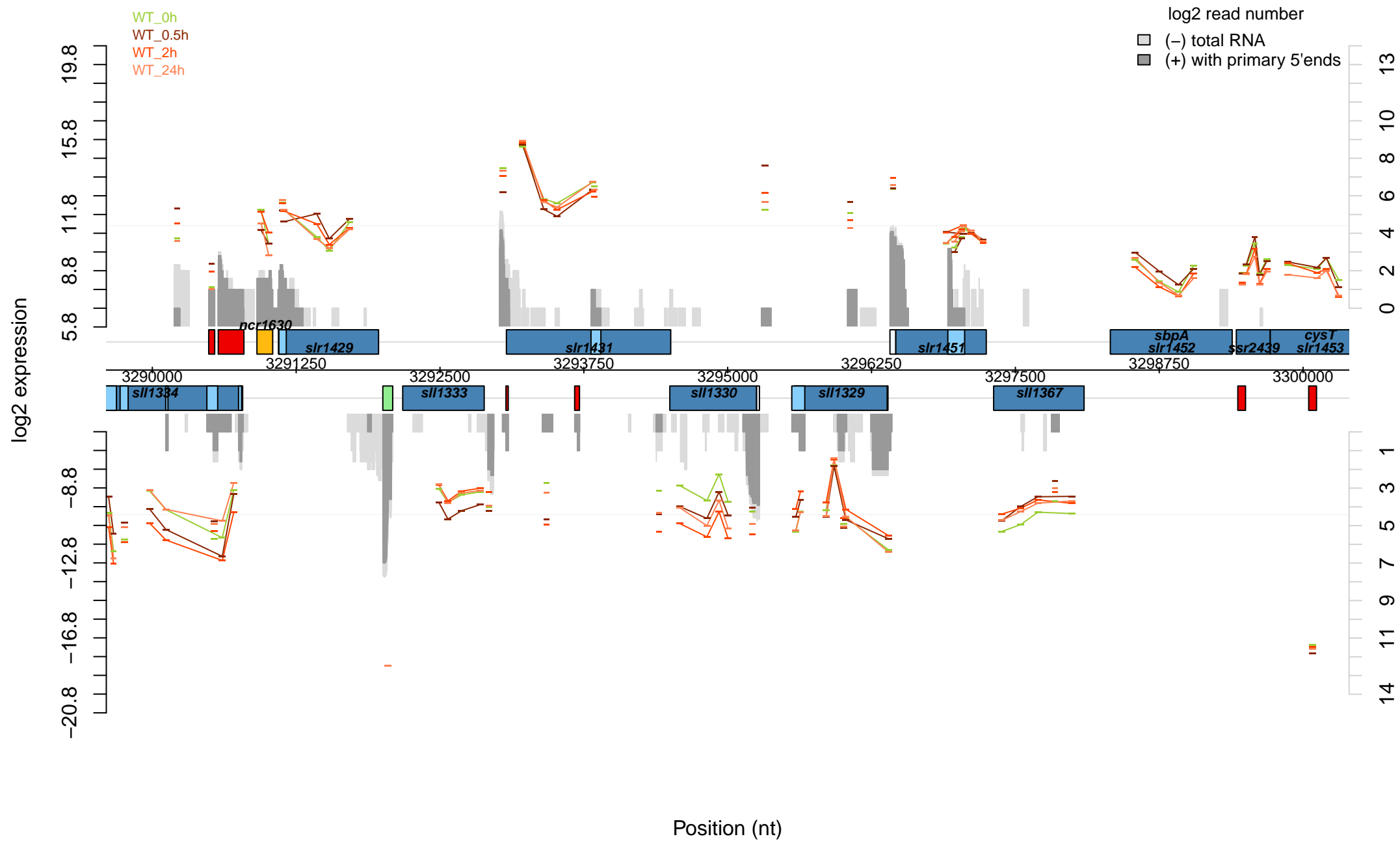

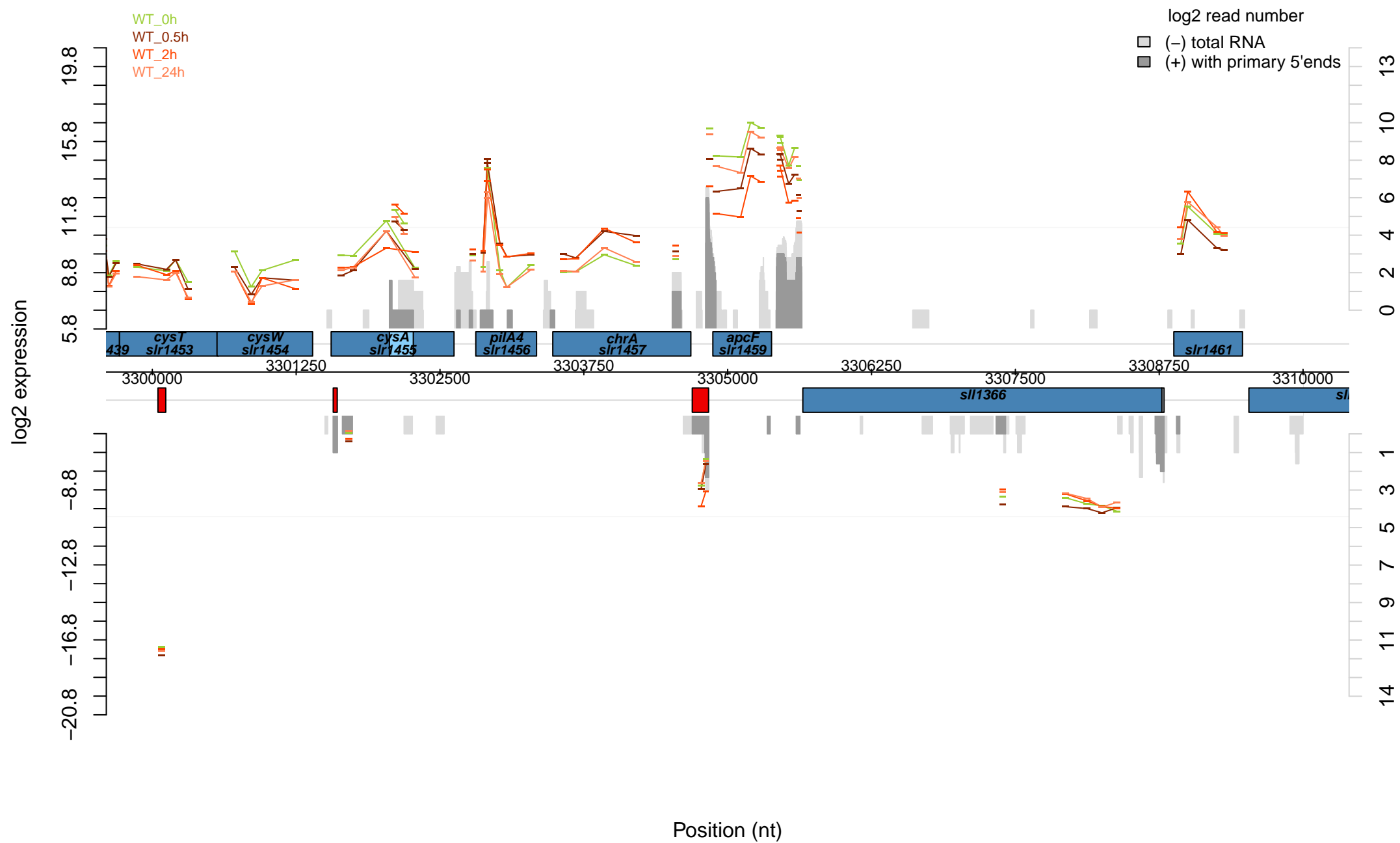

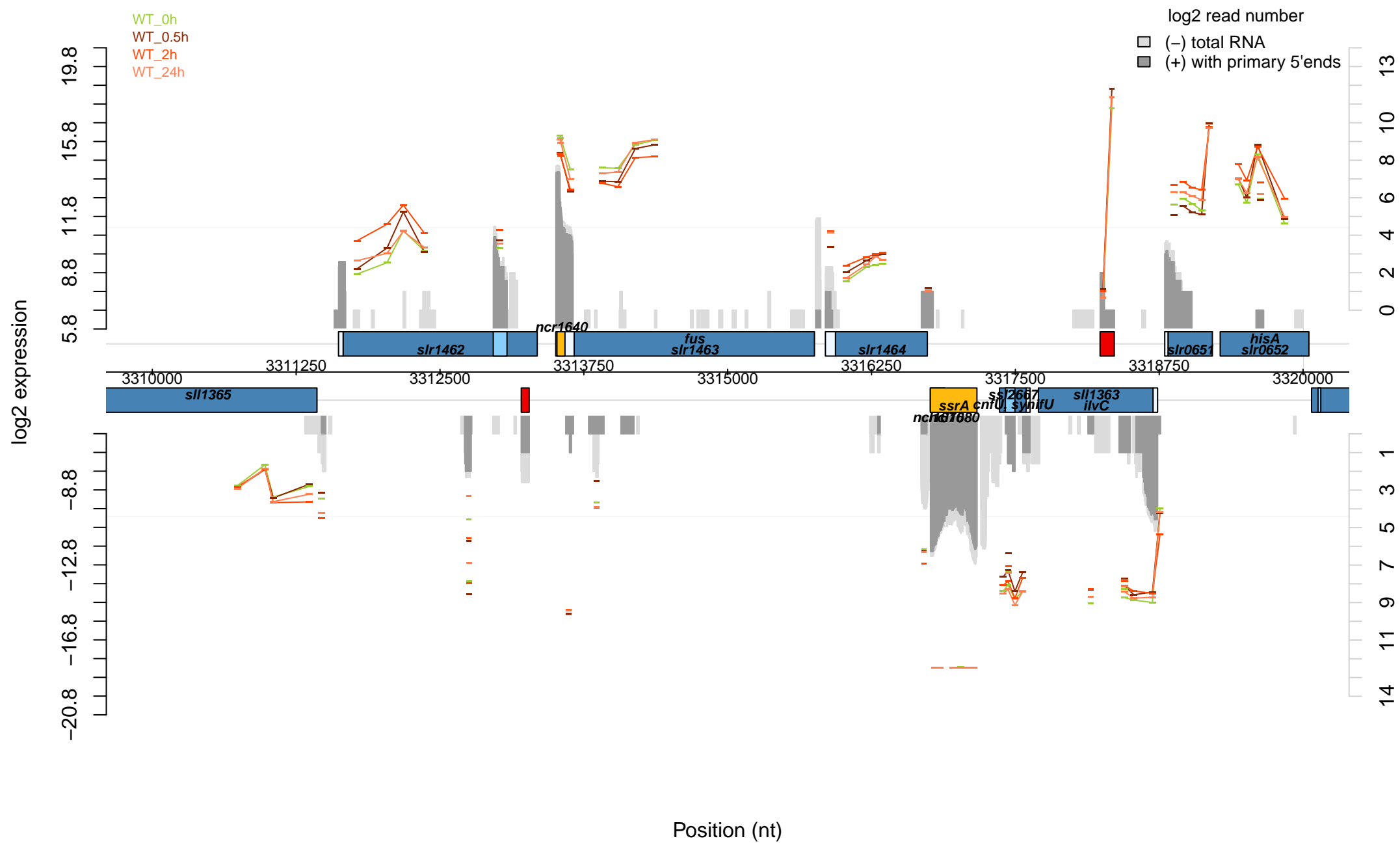

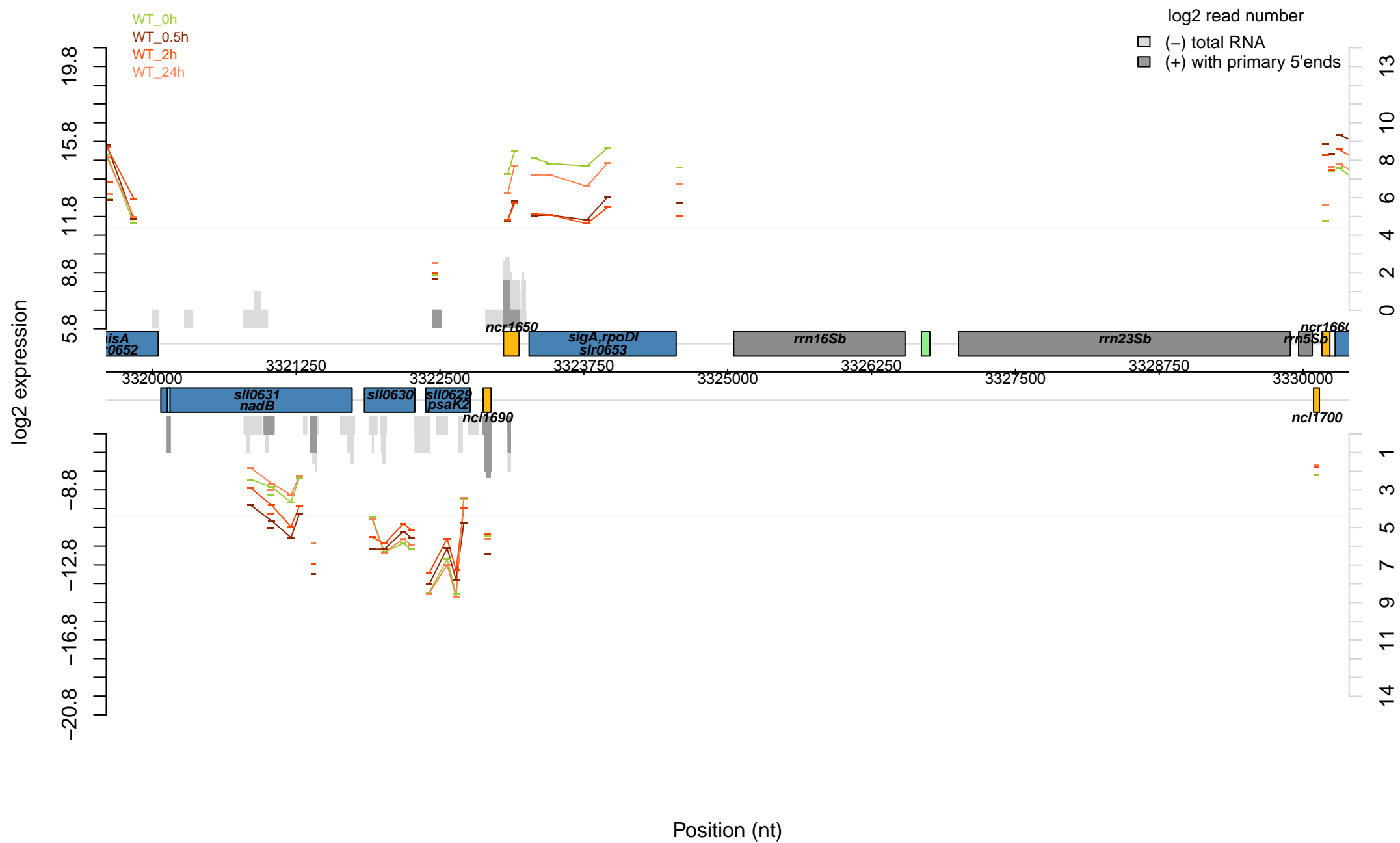

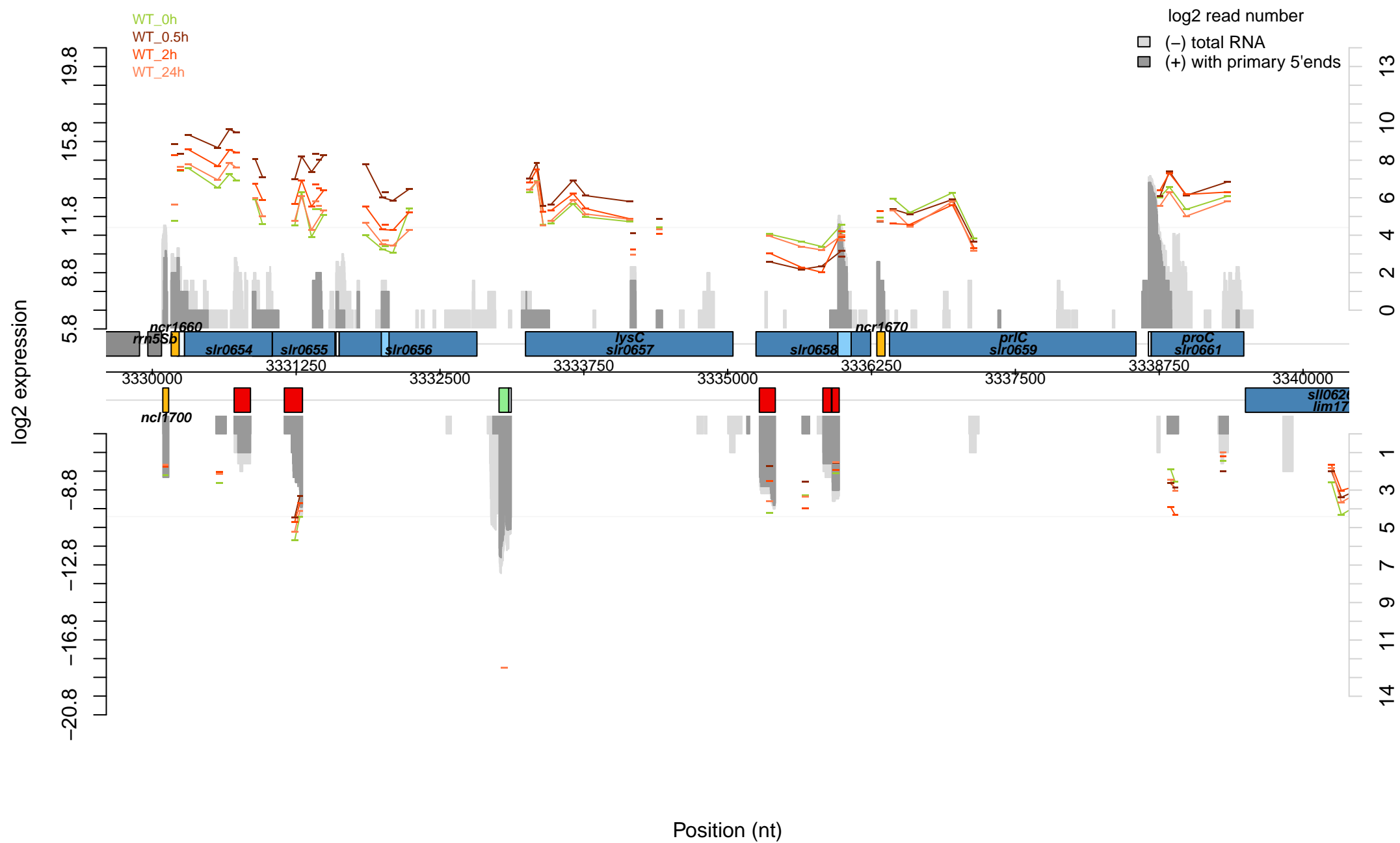

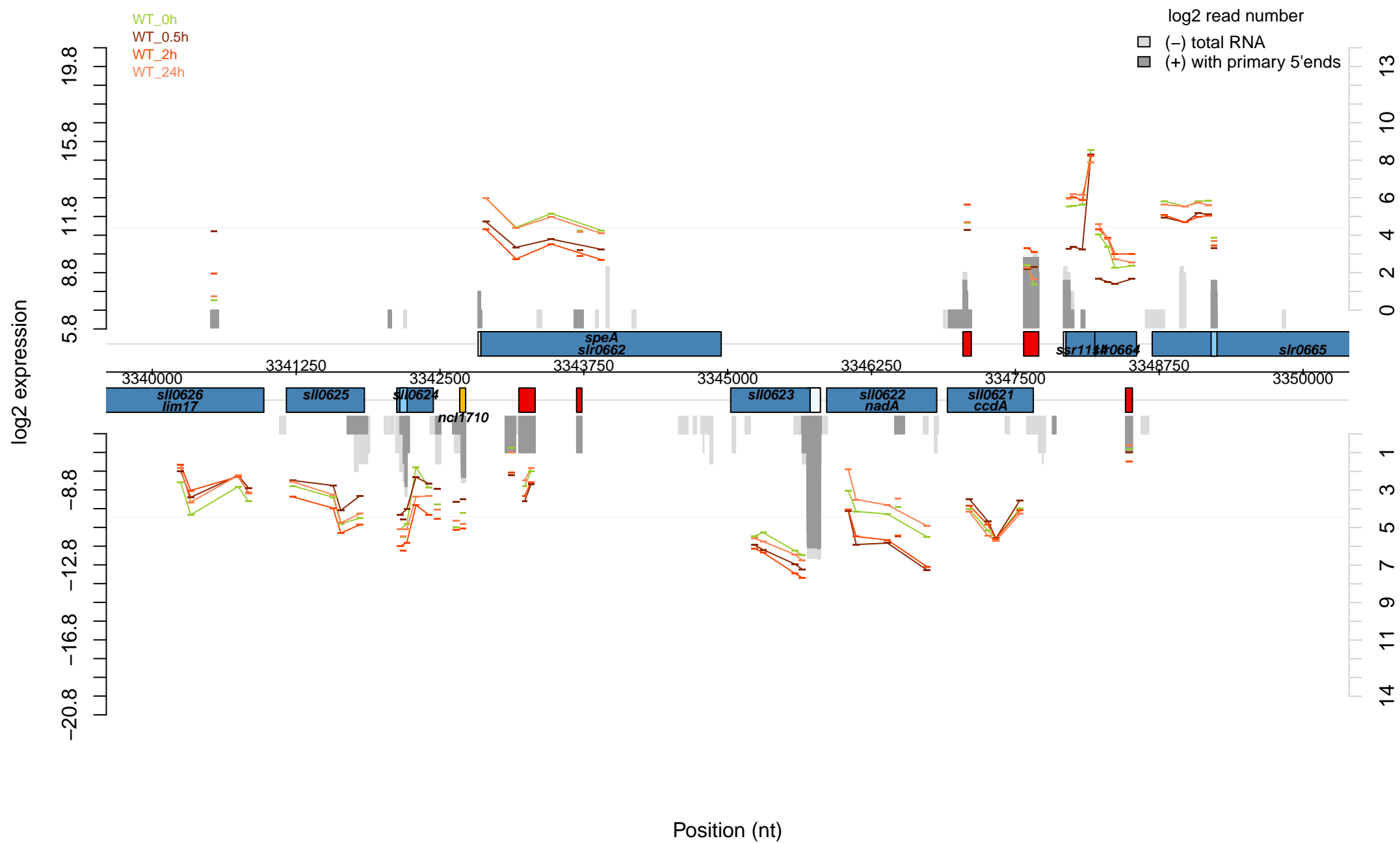

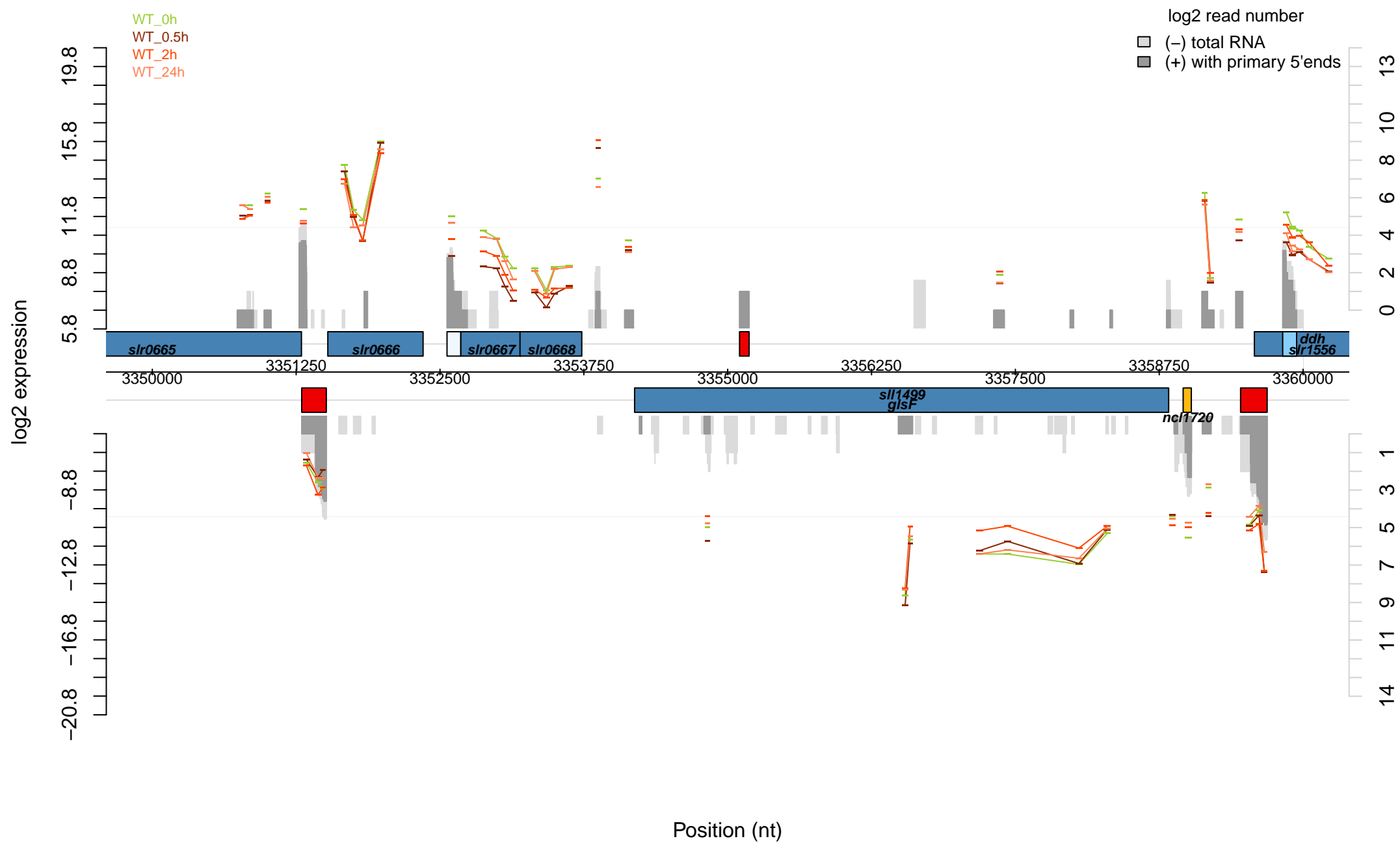

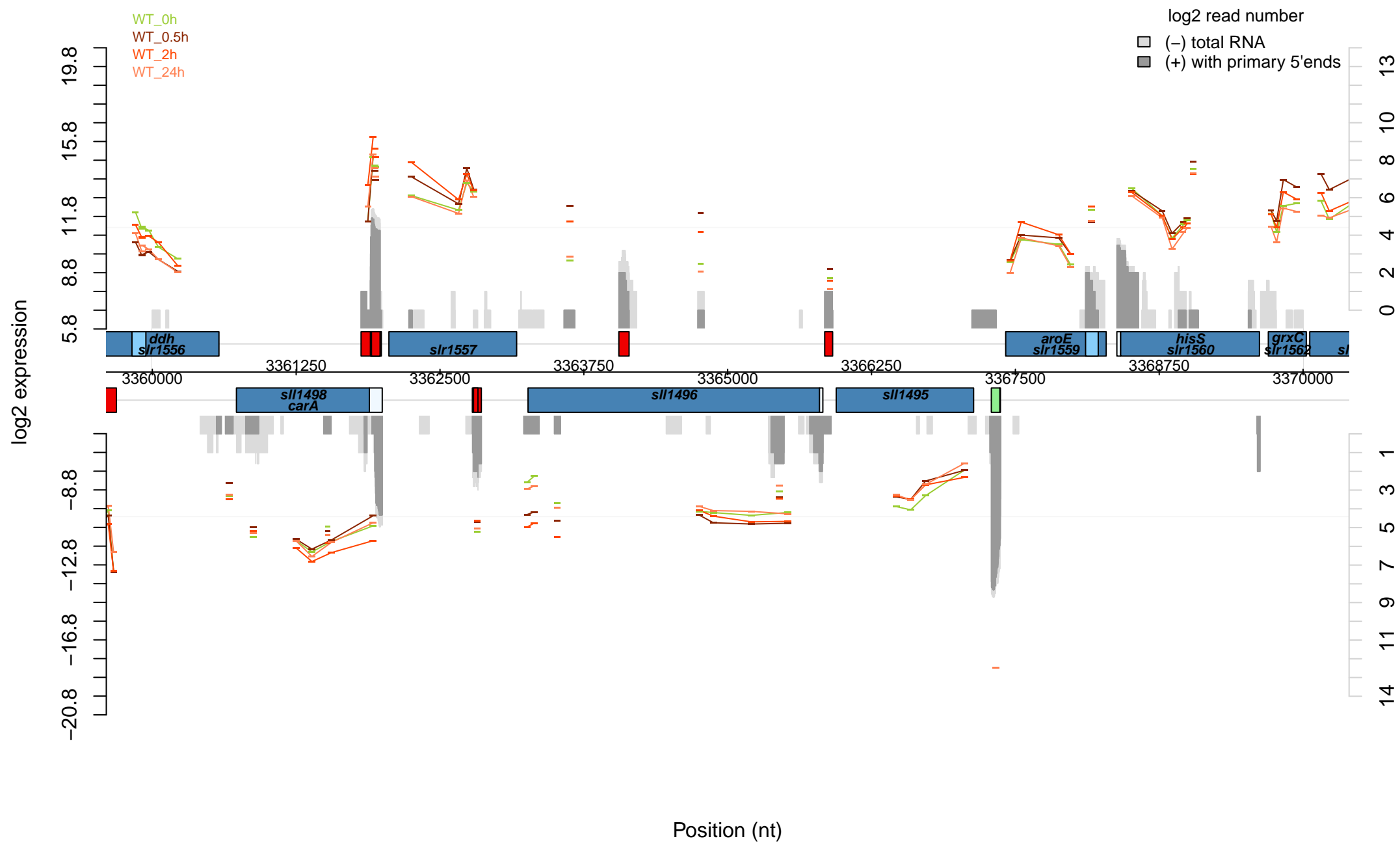

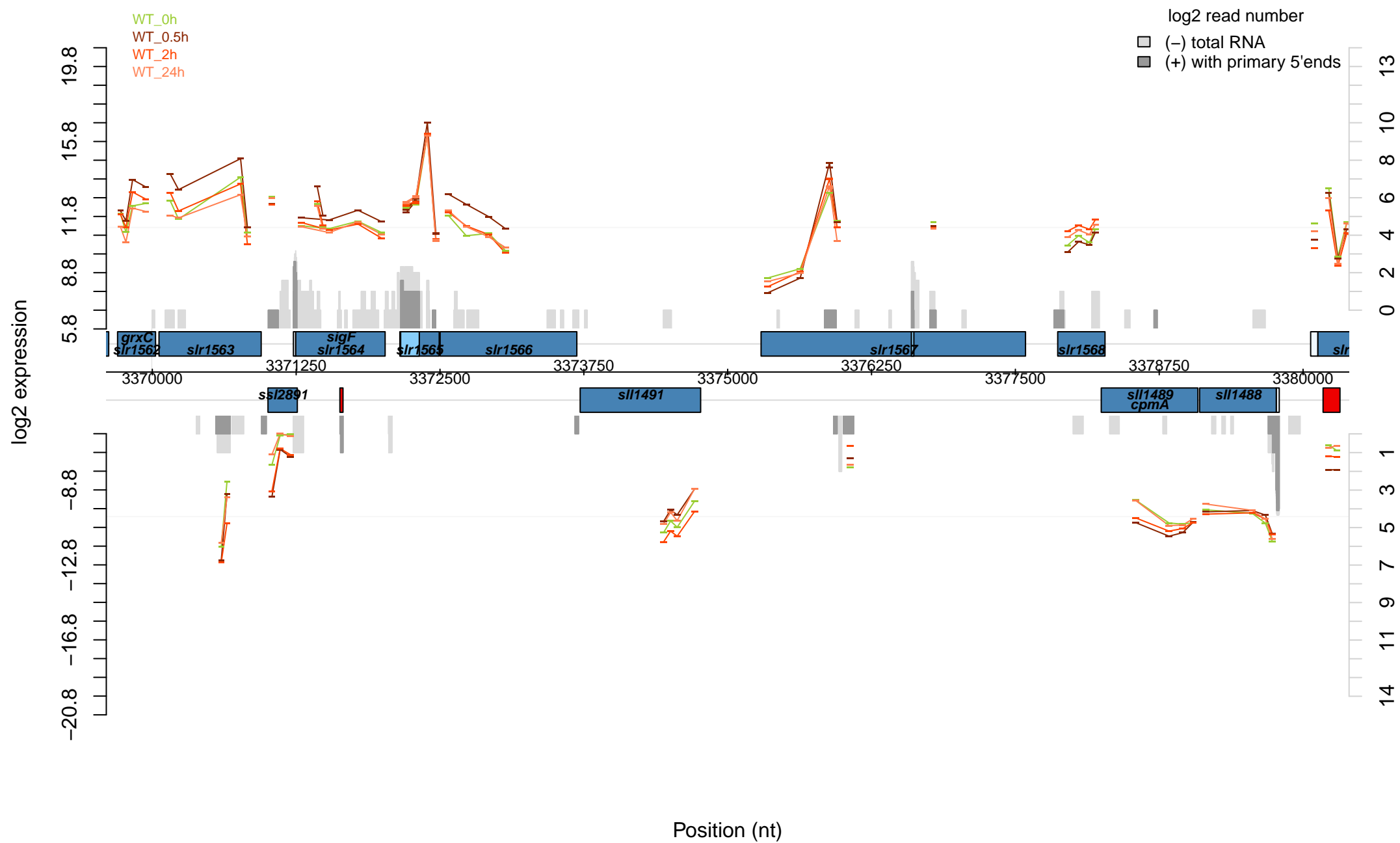

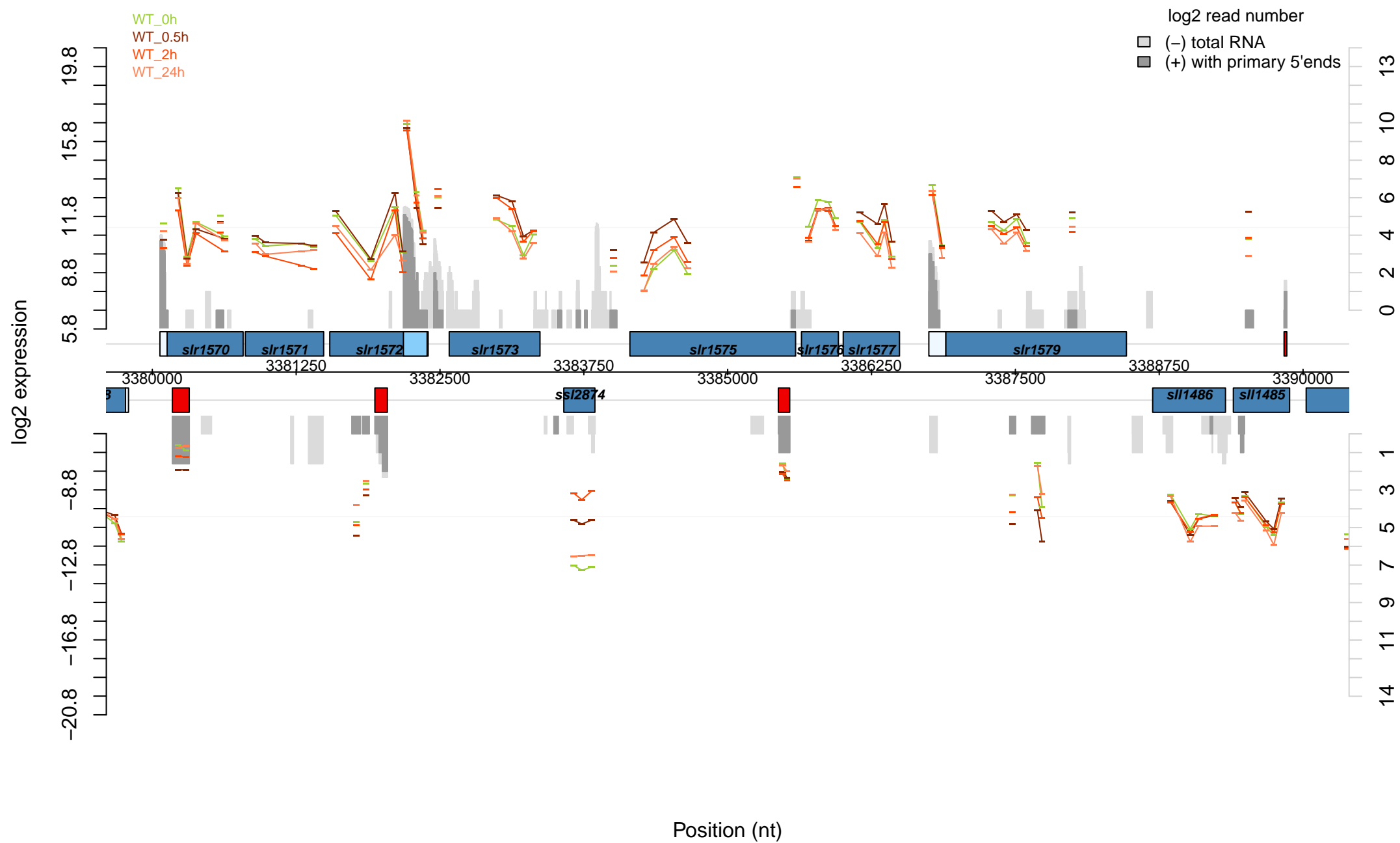

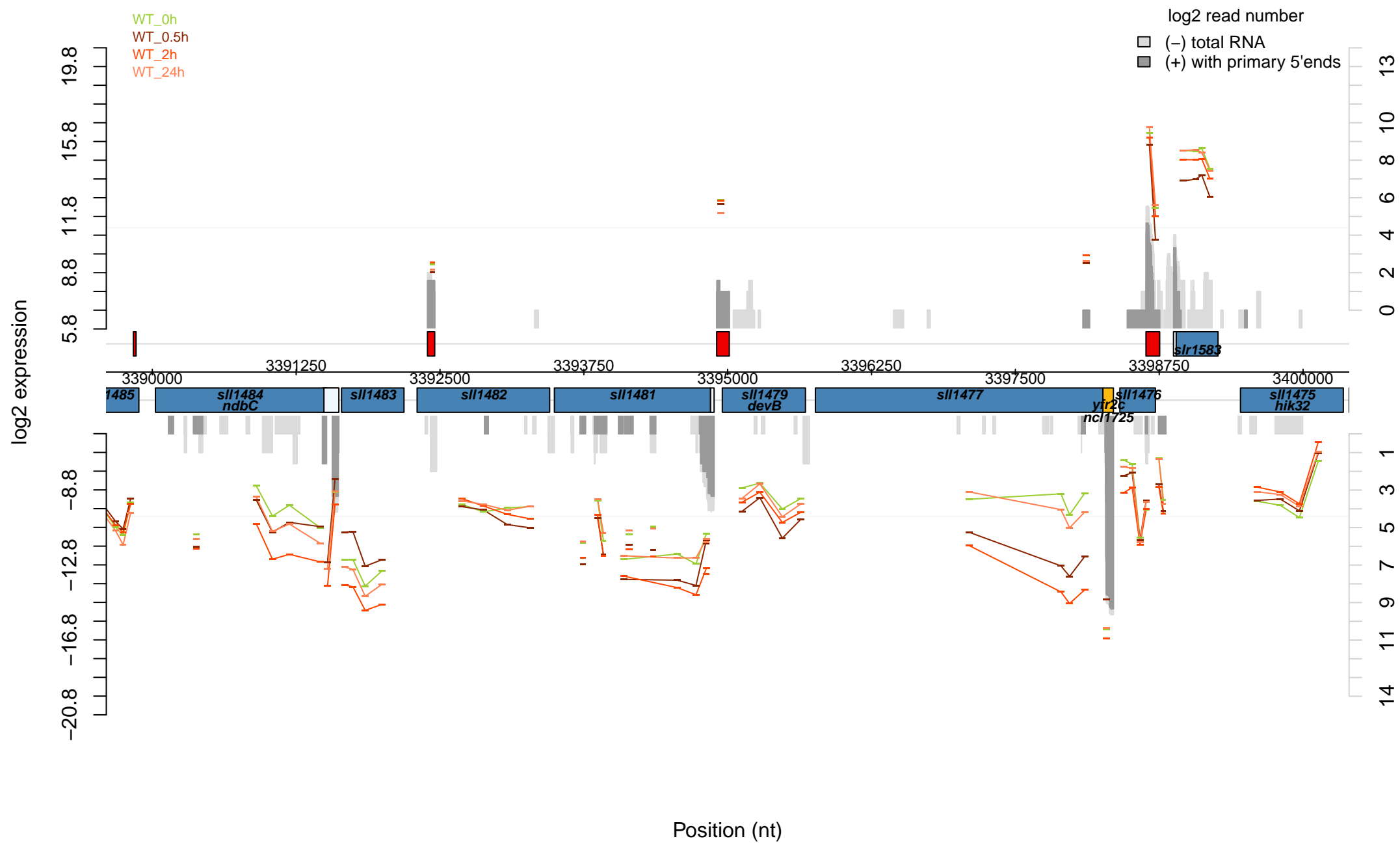

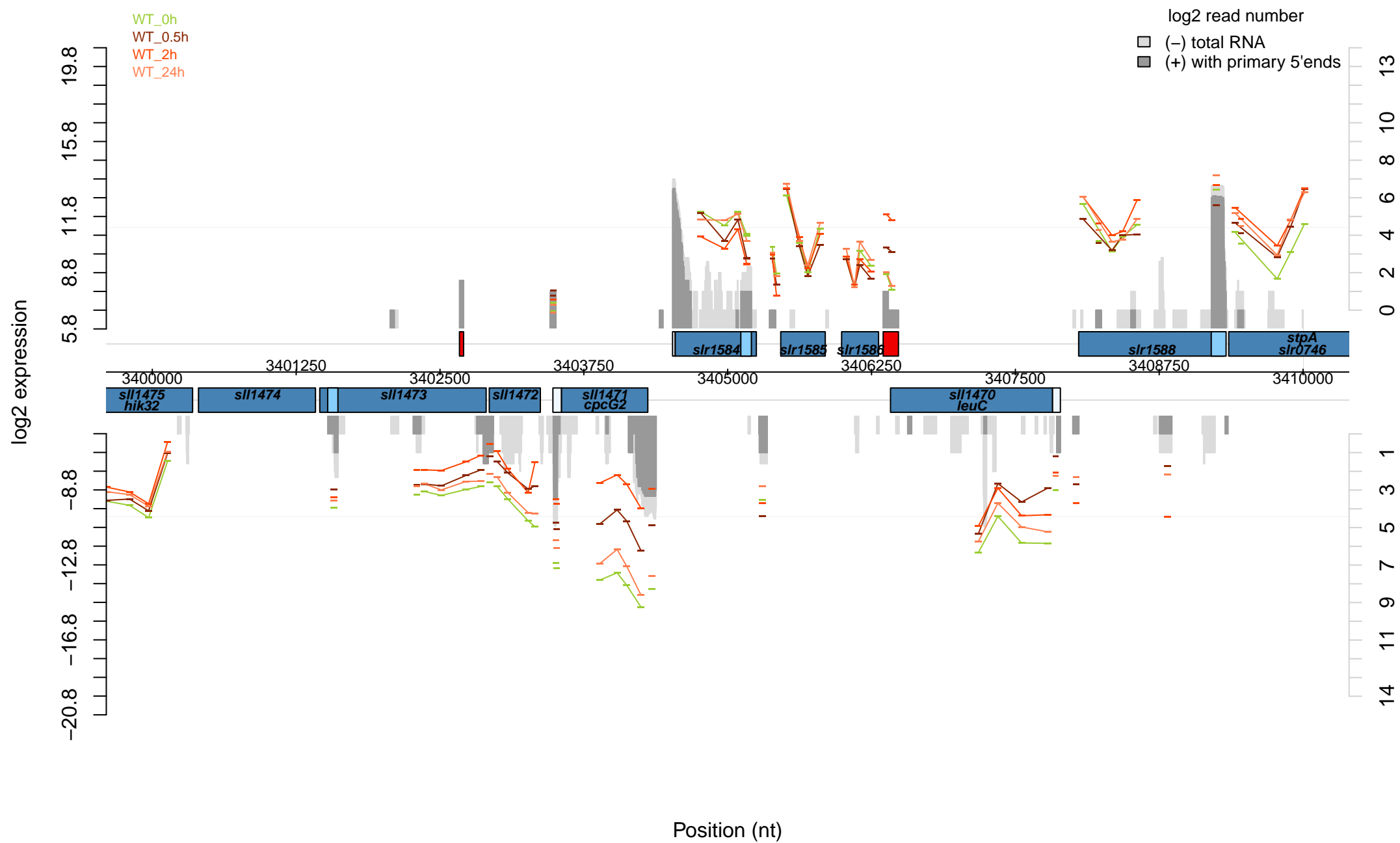

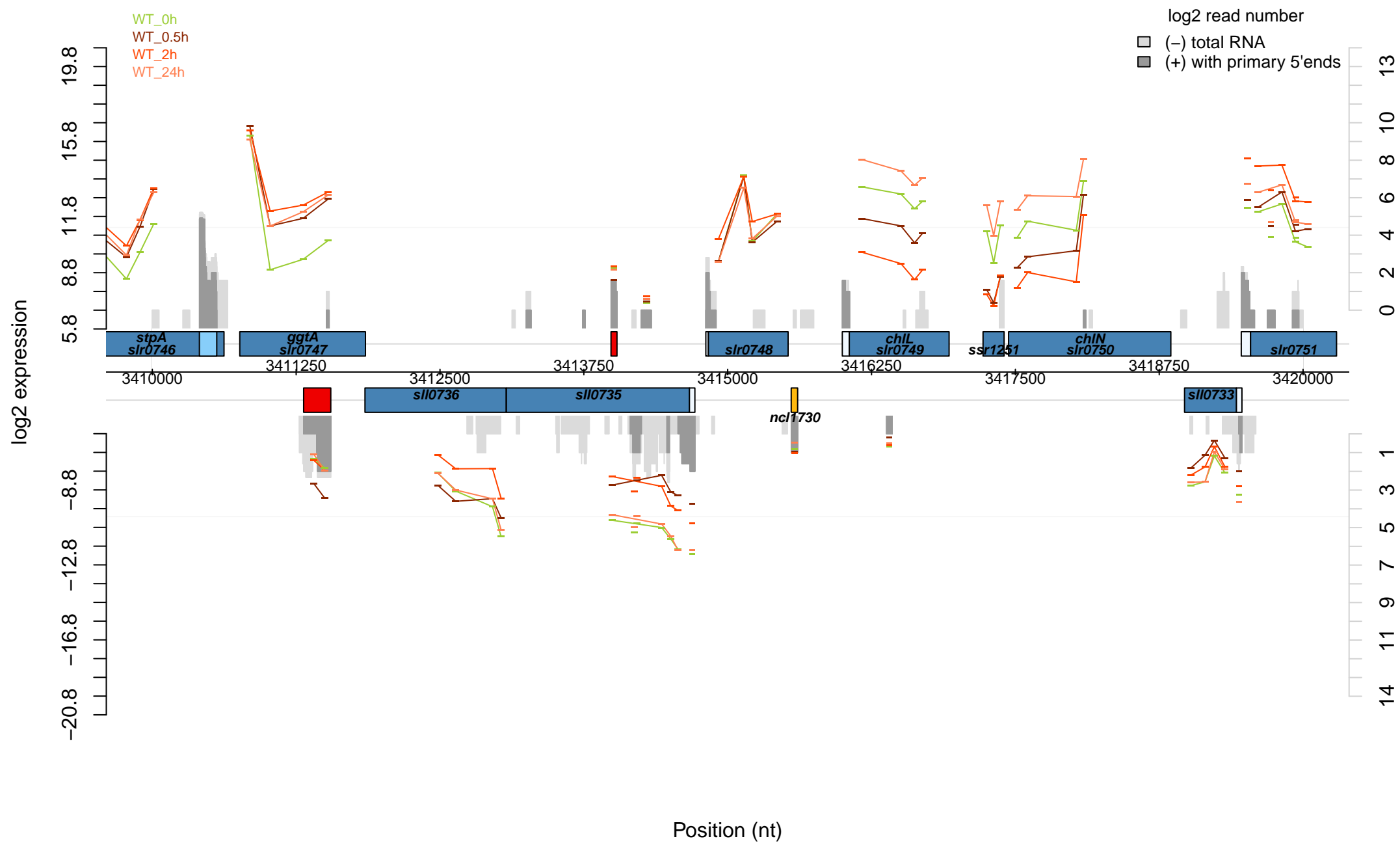

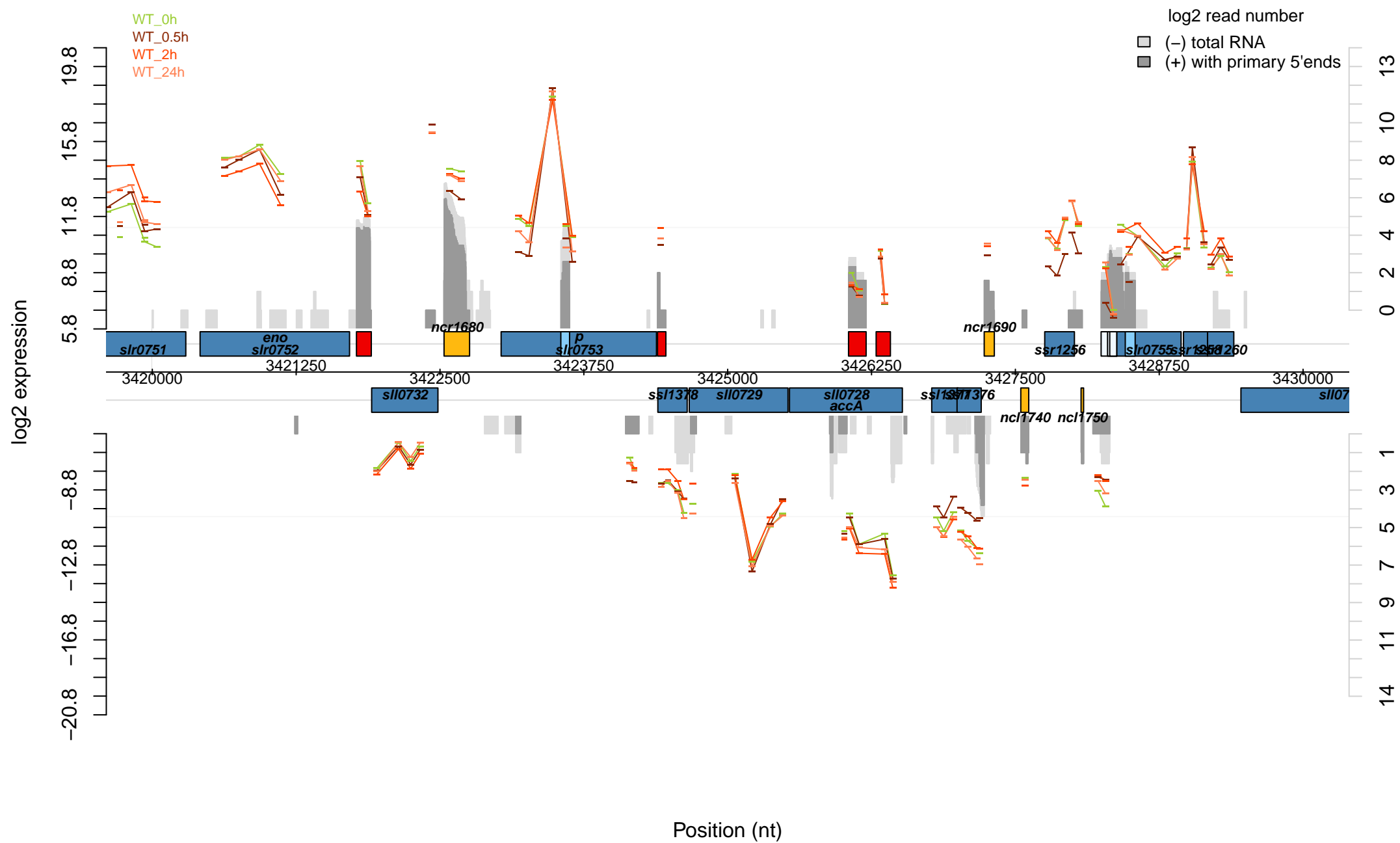

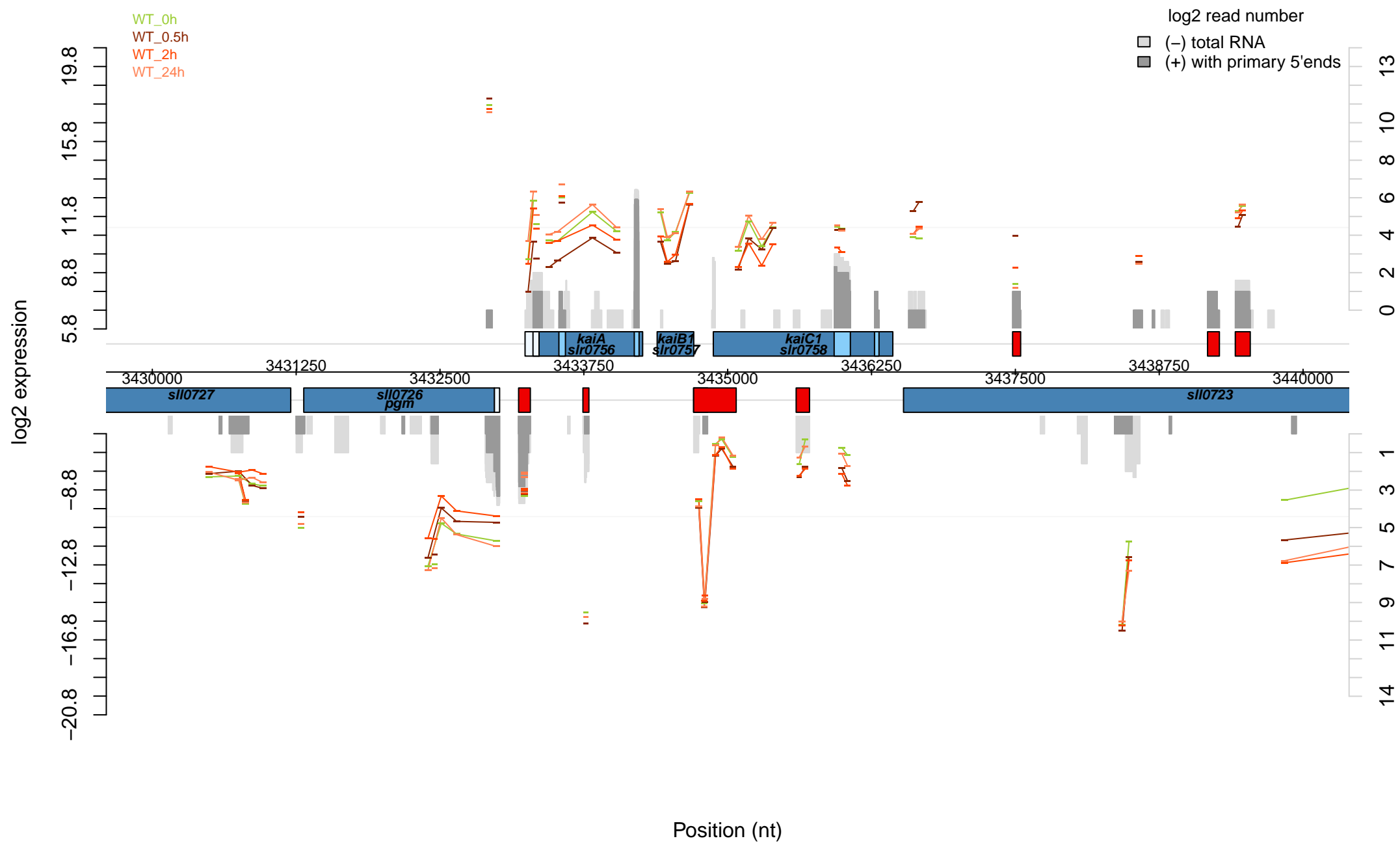

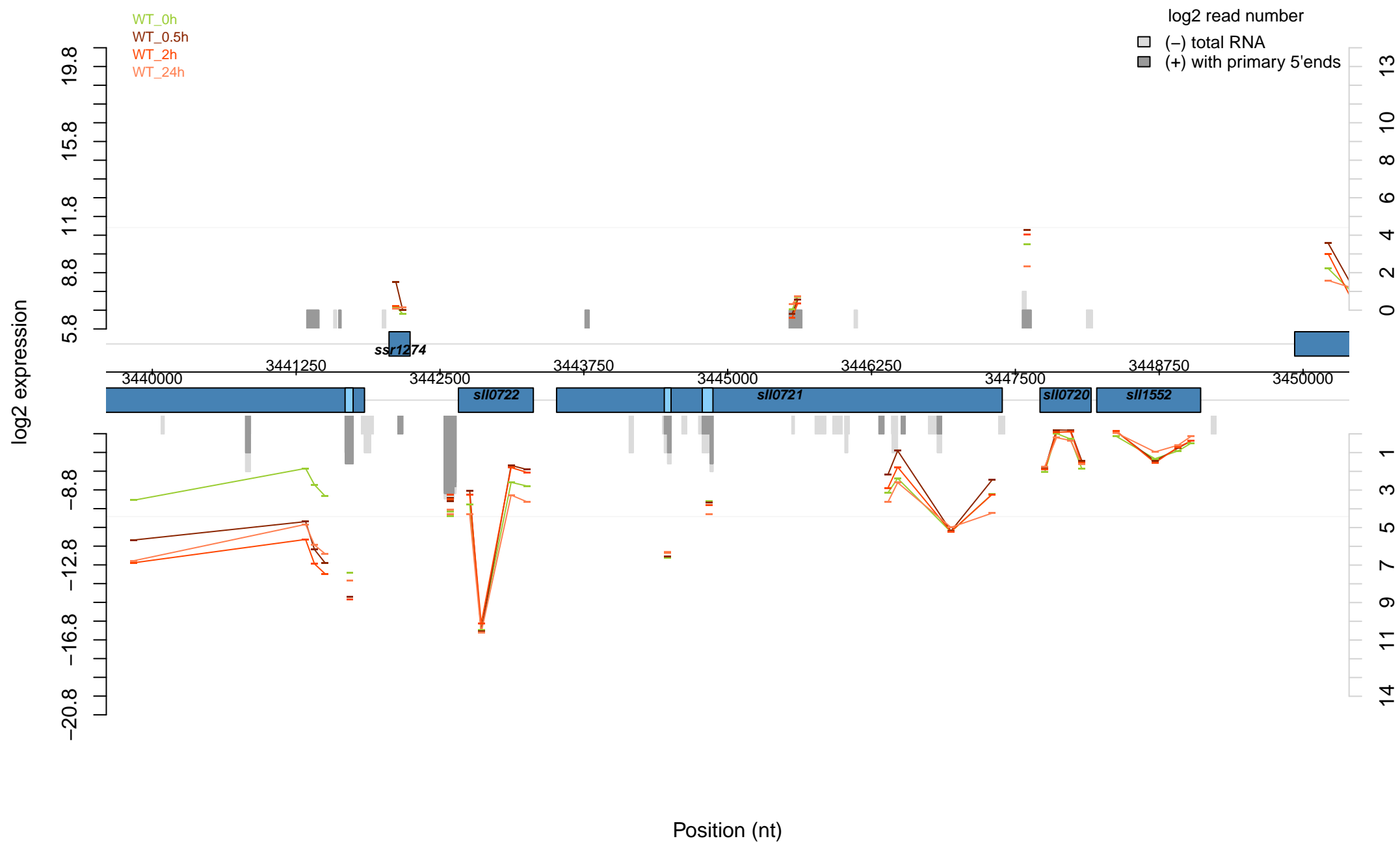

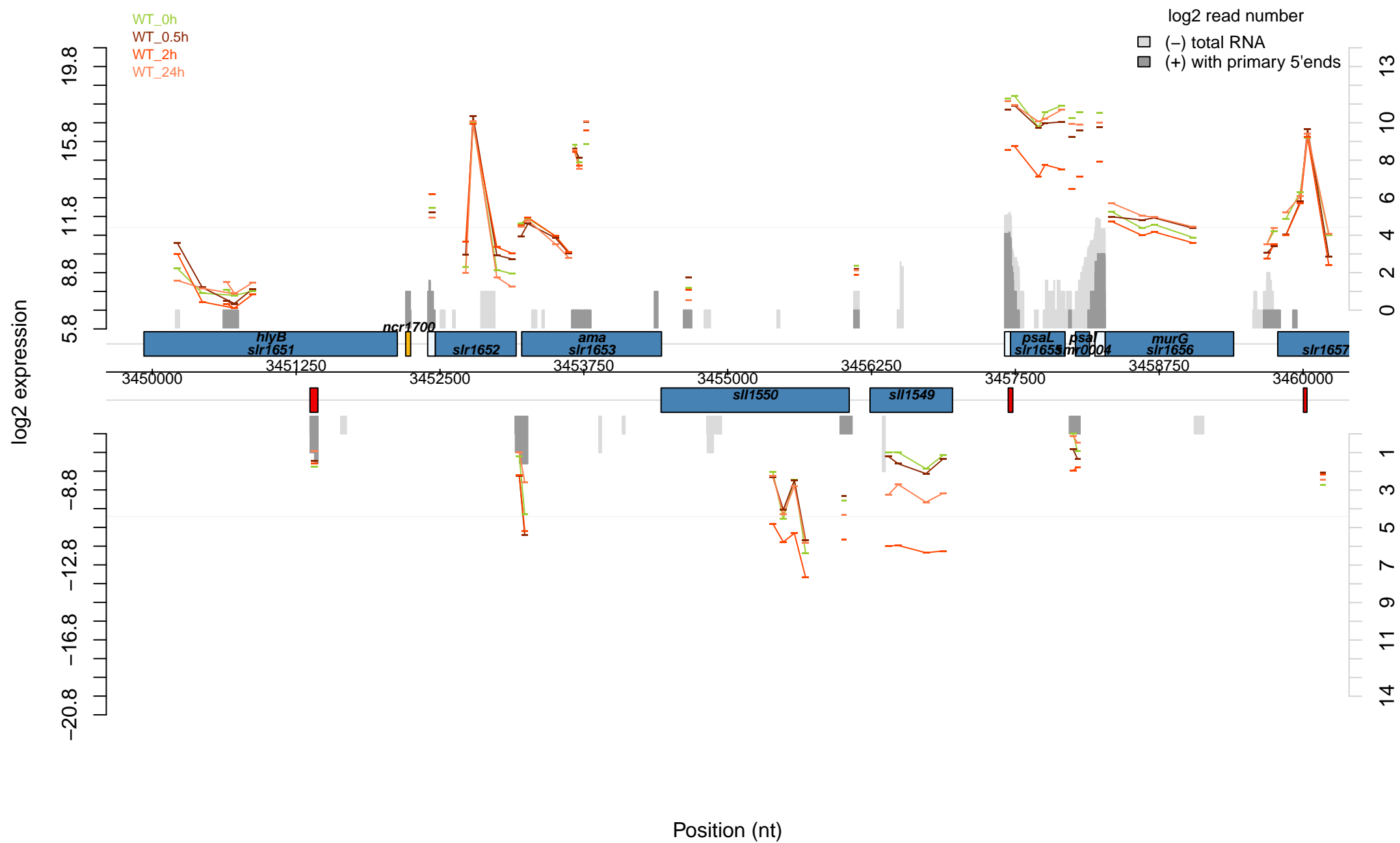

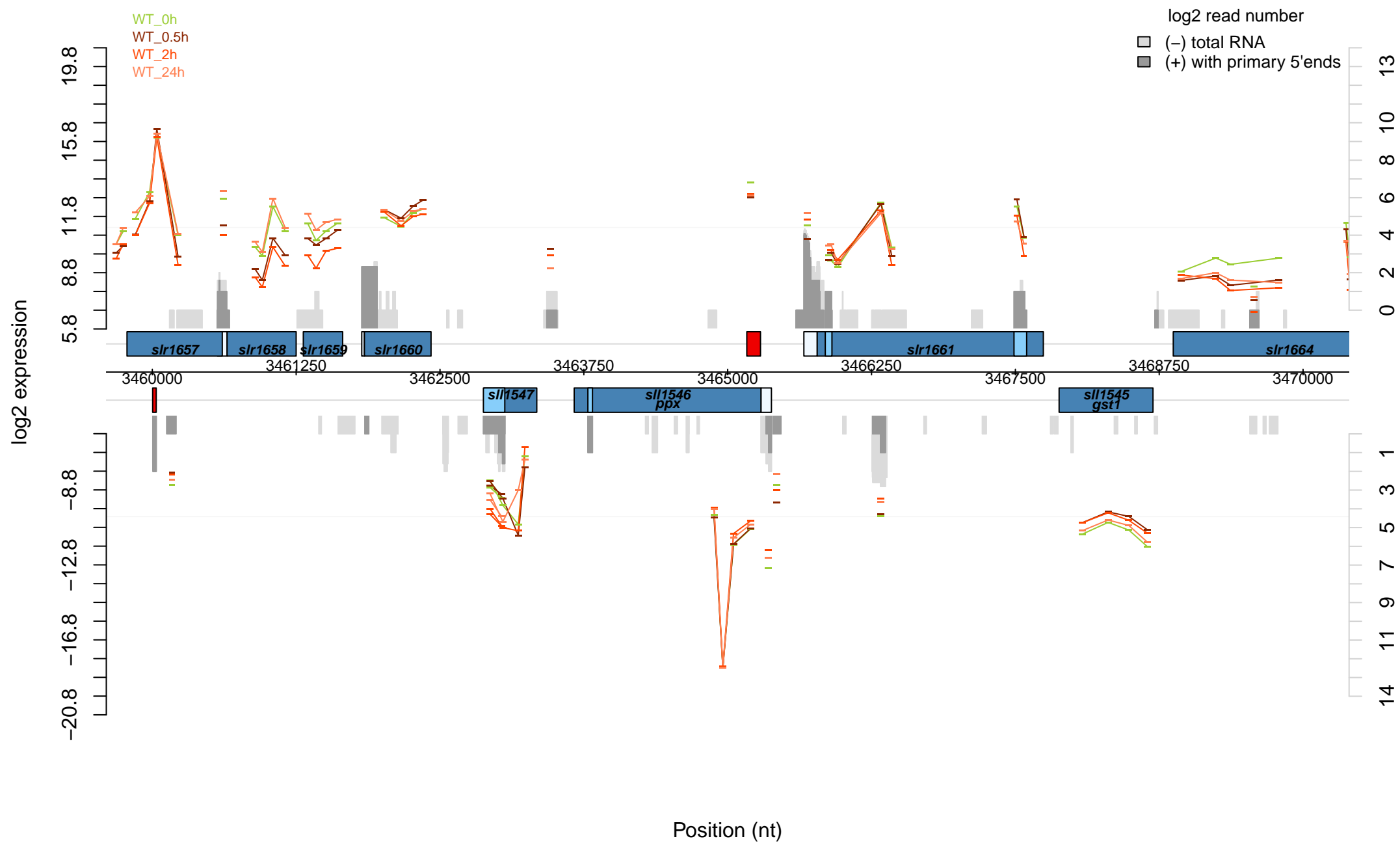

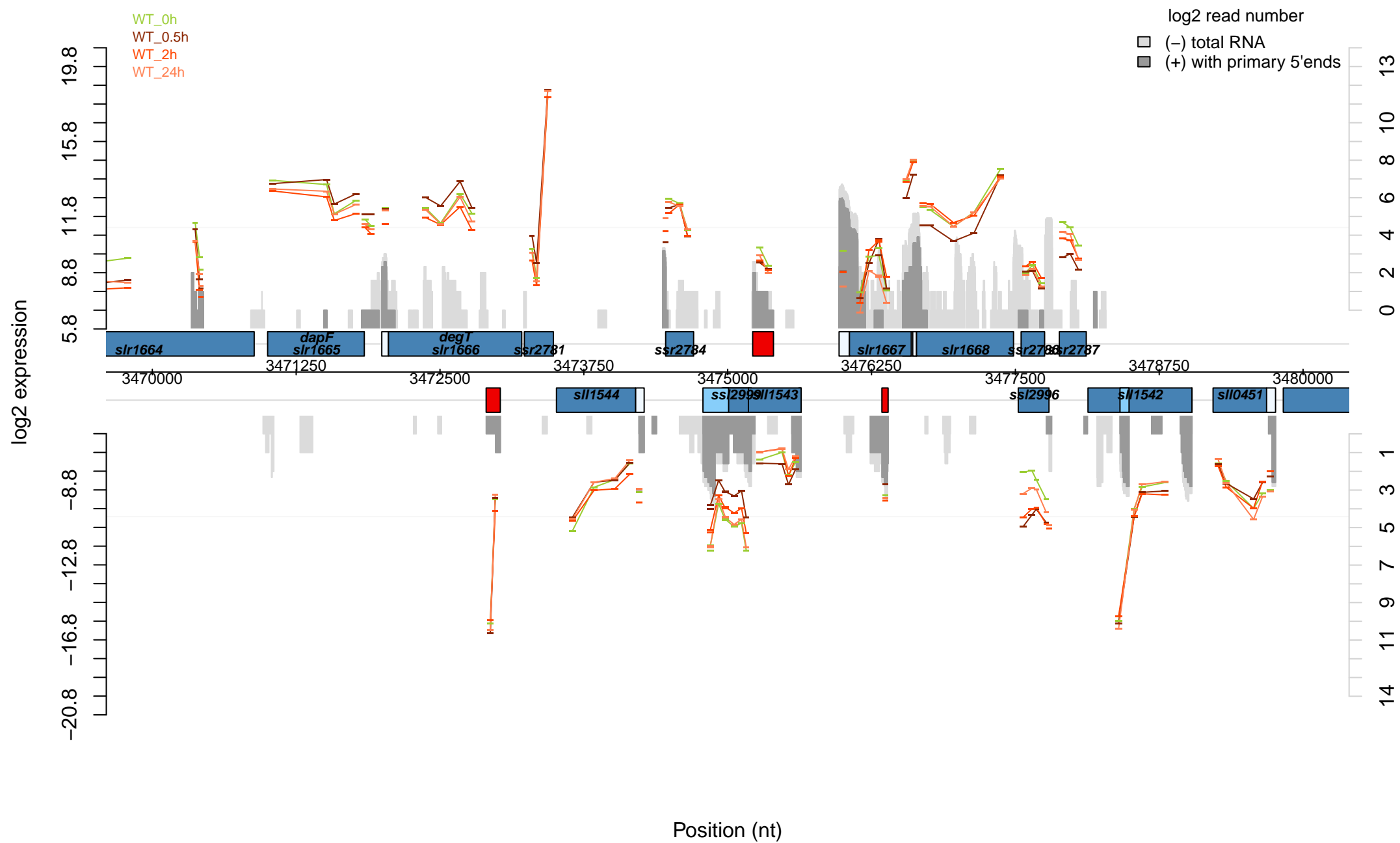

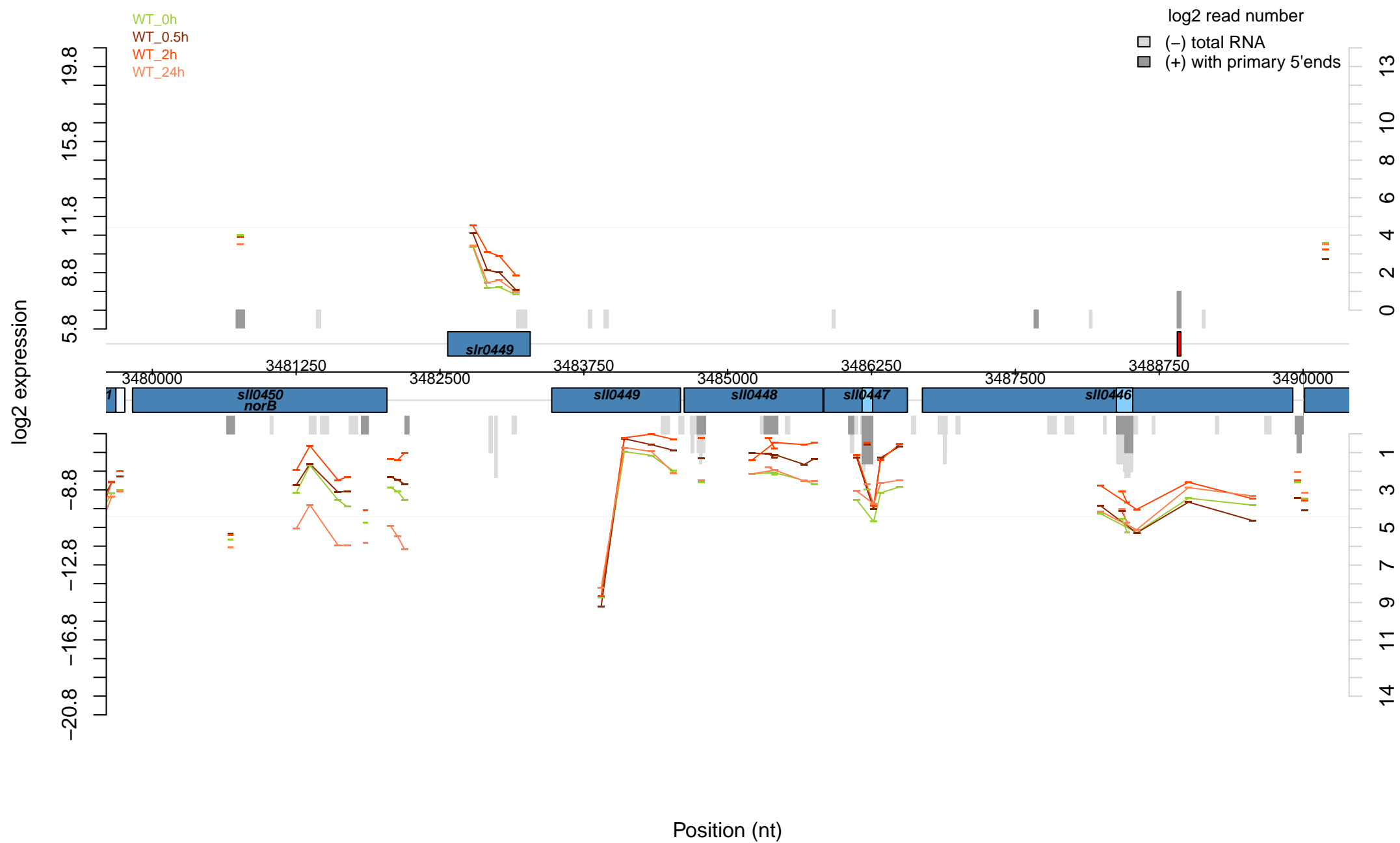

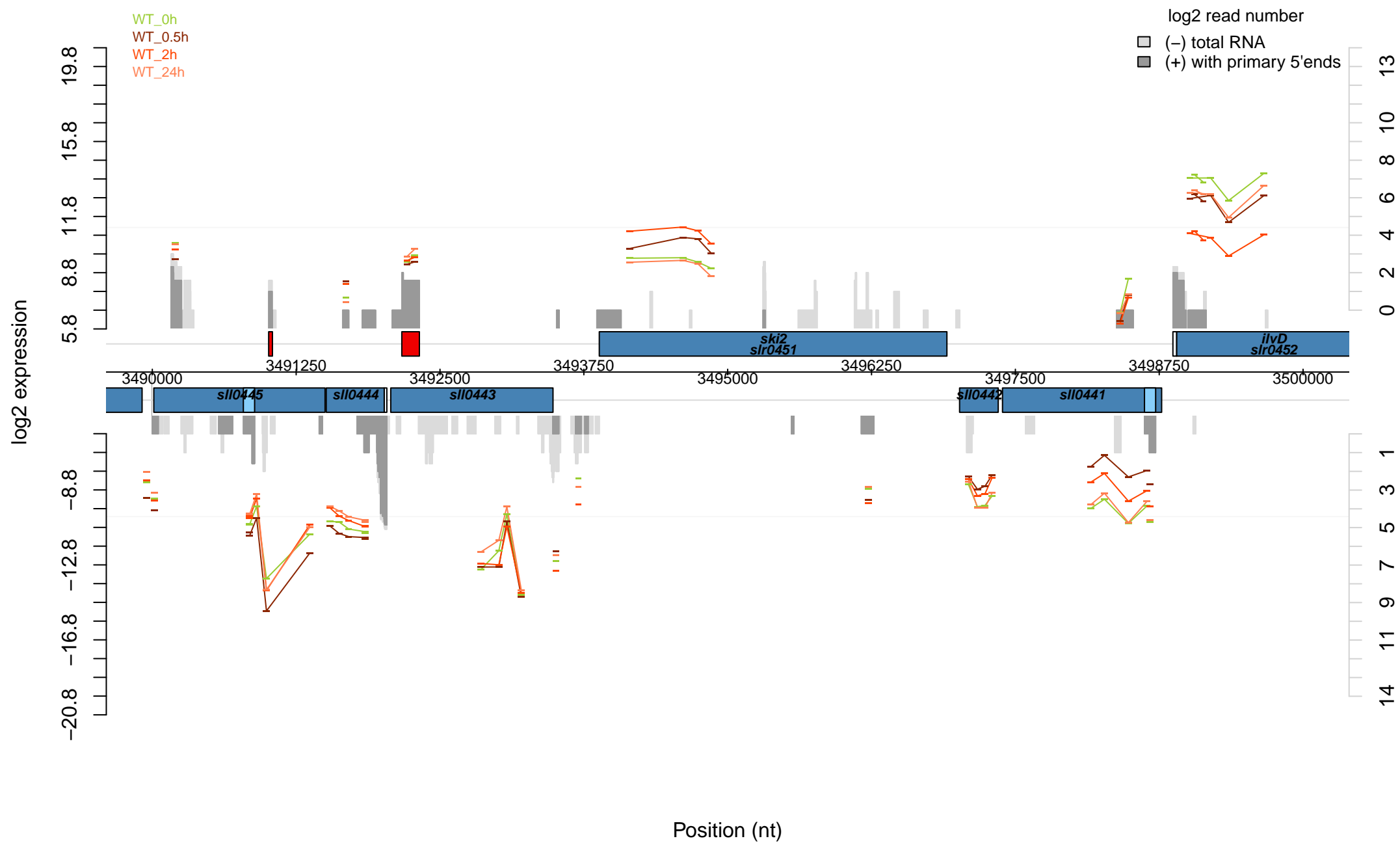

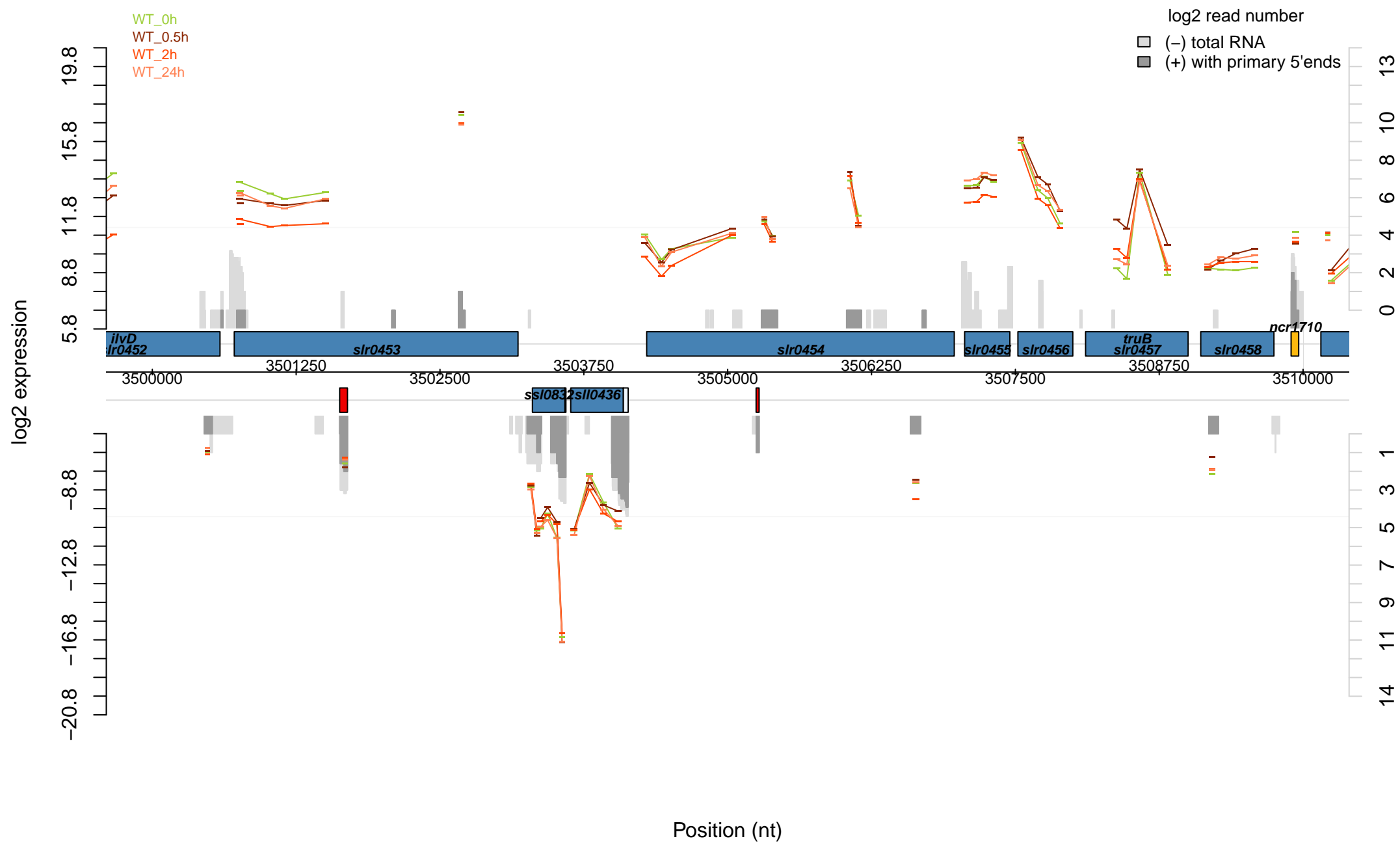

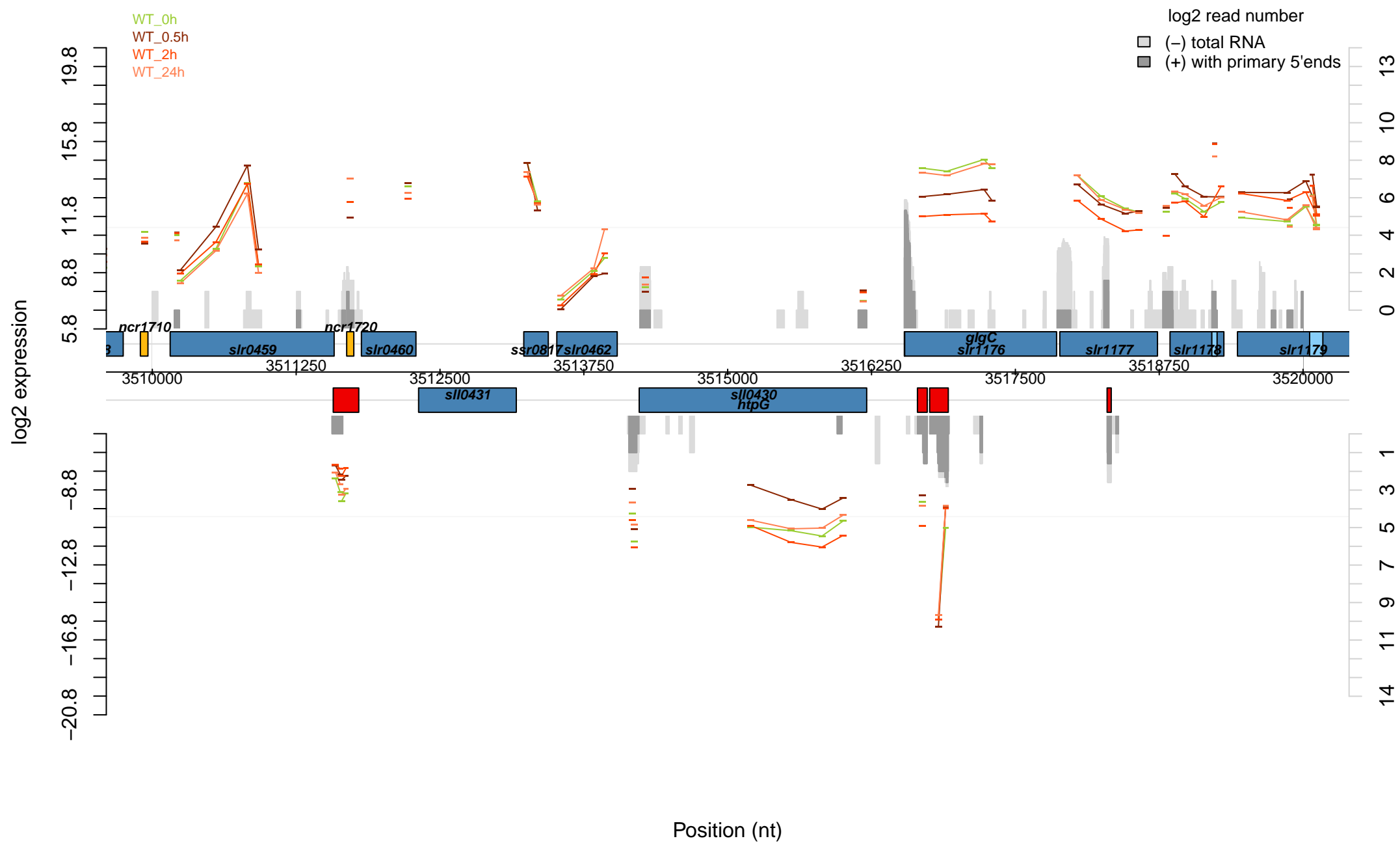

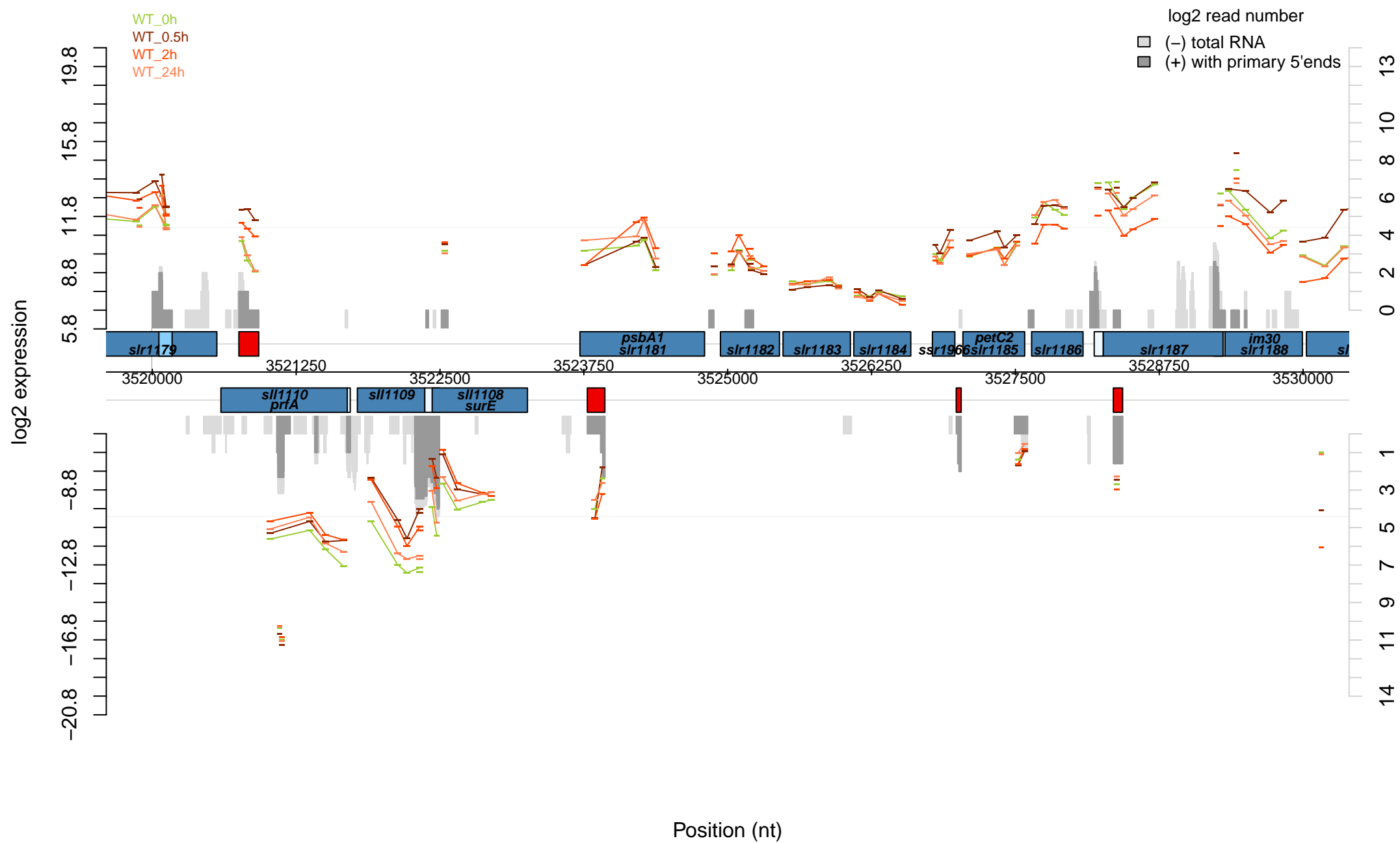

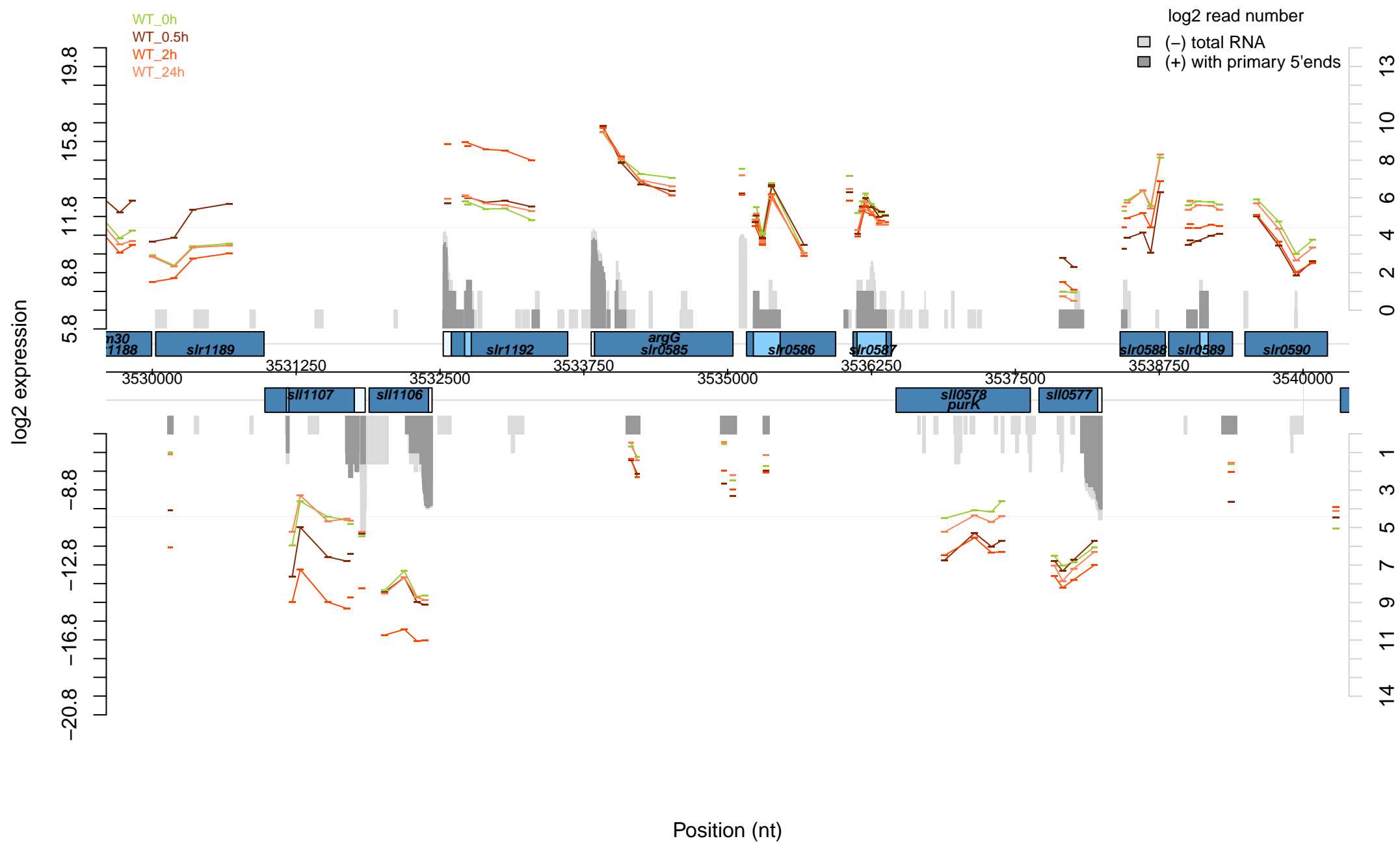

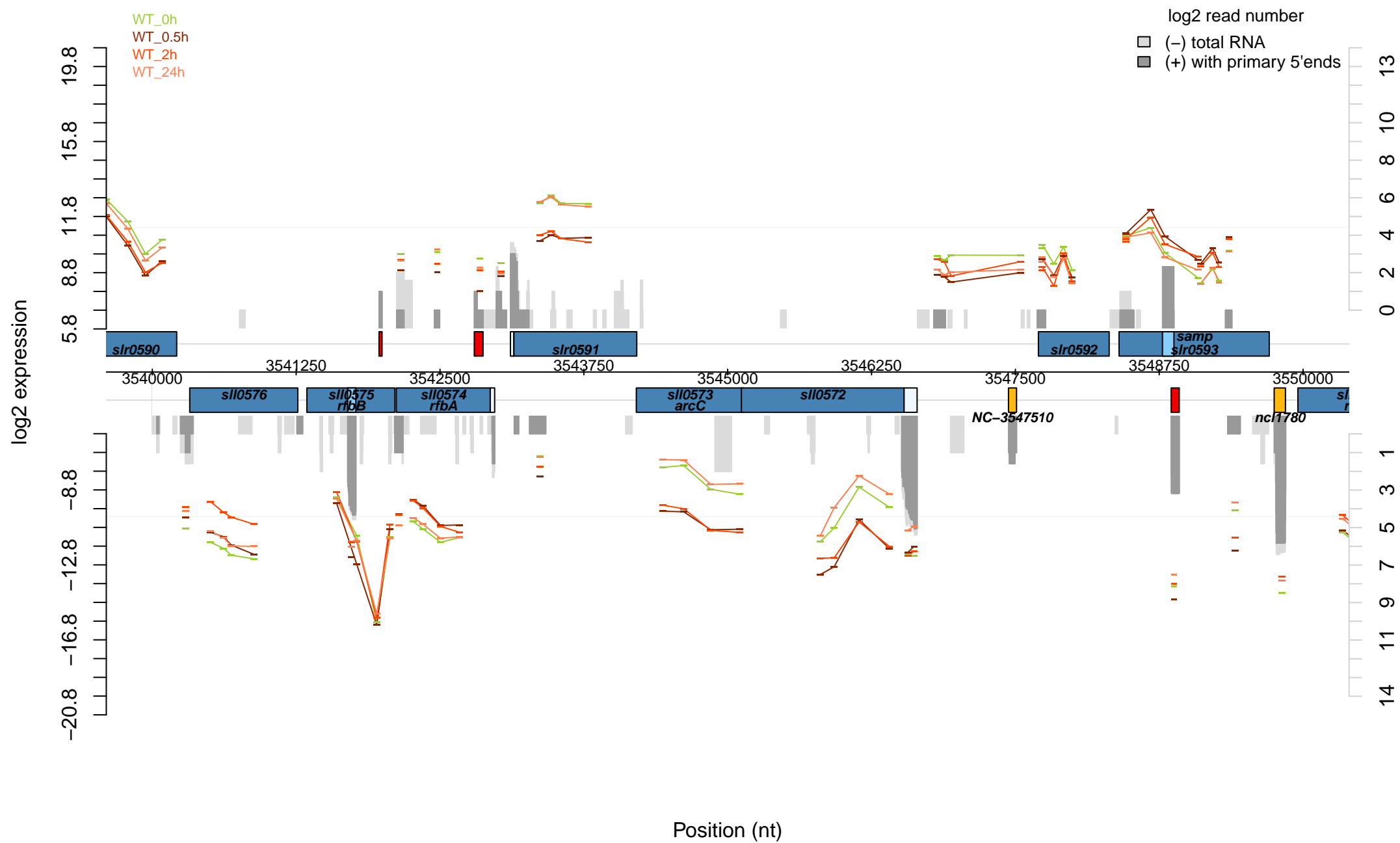

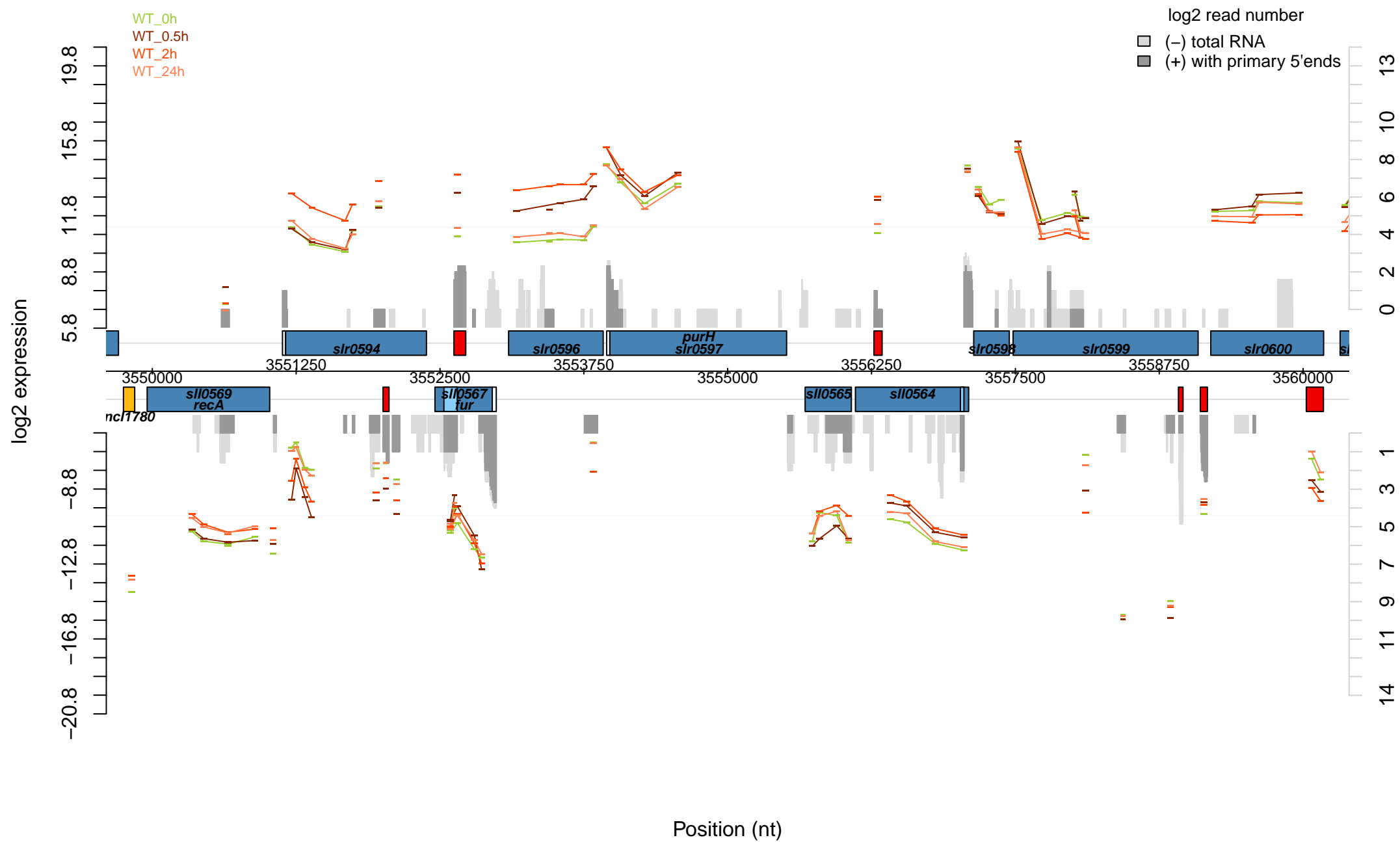

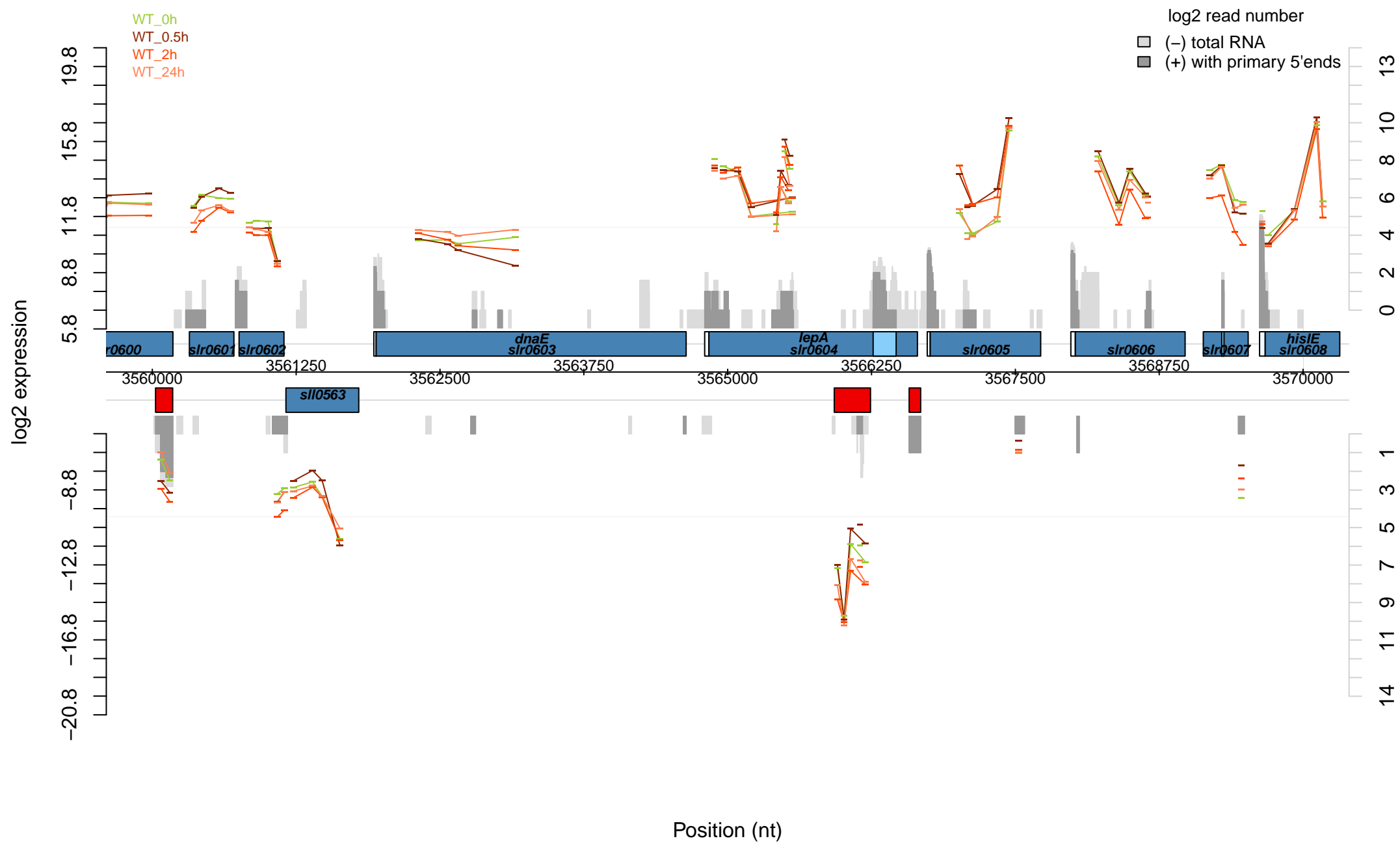

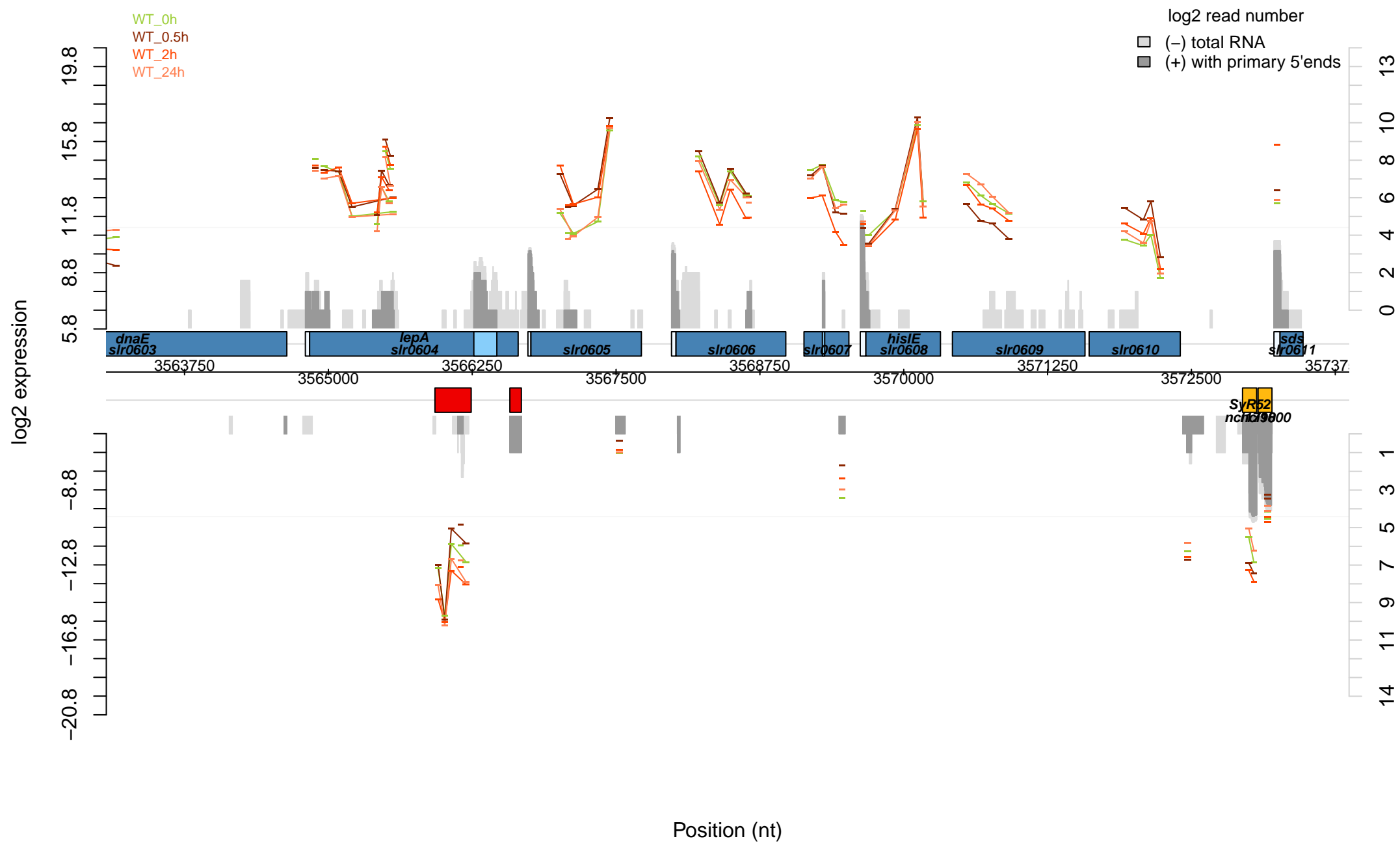

Supplement: Supplementary file 2 — Additional file 2. Genome plot of Synechocystis sp. PCC 6803 chromosome displaying the microarray data for each single probe. [file 13062_2021_316_MOESM2_ESM.pdf]
